# Mass spectrometry-based mRNA sequence mapping *via* complementary RNase digests and bespoke visualisation tools†

**DOI:** 10.1039/d5an00033e

**Published:** 2025-01-30

**Authors:** Emma N. Welbourne, Royce J. Copley, Gareth R. Owen, Caroline A. Evans, Kesler Isoko, Ken Cook, Joan Cordiner, Zoltán Kis, Peyman Z. Moghadam, Mark J. Dickman

**Affiliations:** a School of Chemical, Materials and Biological Engineering, University of Sheffield Sheffield UK m.dickman@sheffield.ac.uk; b Department of Chemical Engineering, University College London London UK; c ThermoFisher Scientific Hemel Hempstead UK; d Department of Chemical Engineering, Imperial College London London UK

## Abstract

mRNA technology has significantly changed the timeline for developing and delivering a new vaccine from years to months, as demonstrated by the development and approval of two highly efficacious vaccines based on mRNA sequences encoding for a modified version of the SARS-CoV-2 spike protein. Analytical methods are required to characterise mRNA therapeutics and underpin manufacturing development. In this study, we have developed and utilised partial RNase digests of mRNA using RNase T1 and RNase U2 in conjunction with an automated, high throughput workflow for the rapid characterisation and direct sequence mapping of mRNA therapeutics. In conjunction with this, we have developed novel software engineered to optimise and streamline the visualisation and analysis of sequence mapping of mRNA using LC-MS/MS. We show that increased mRNA sequence coverage is obtained by combining multiple partial RNase T1 digests-44% and 37% individually, 64% together-or RNase T1 and U2 partial digests-73% and 52% individually, 88% combined. The developed software automates the process of combining digests, ensuring faster and more accurate analysis. Furthermore, the software provides additional information on sequence coverage by taking into account multiple overlapping oligoribonucleotide fragments to increase the confidence of the sequence mapping. Finally, the software enables powerful and accessible visualisation capabilities by generating spiral plots to quickly analyse the sequence maps in a single output from combined multiple partial RNase digests.

## Abstract 2

Bespoke visualization tools were developed to enhance the analysis of complementary partial RNase digests *via* LC-MS. These tools enable efficient identity testing of mRNA through comprehensive sequence mapping.

## Graphical abstract

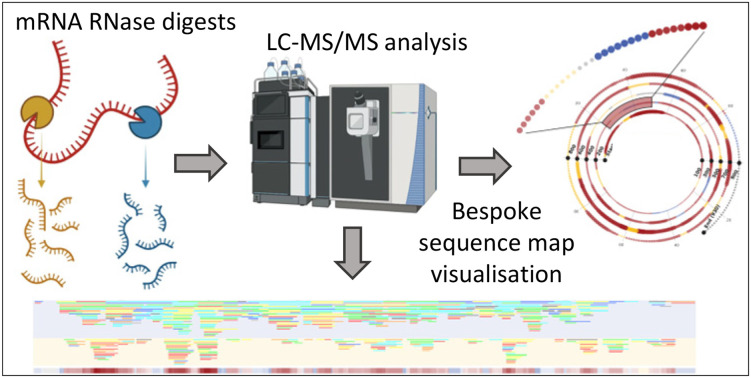

## 1. Introduction

mRNA has recently emerged as a new class of therapeutic, as demonstrated by the development and approval of two highly efficacious vaccines based on mRNA sequences encoding for a modified version of the SARS-CoV-2 spike protein.^1,2^ Furthermore, RNA-based approaches have the potential for treatments beyond vaccines and infectious diseases as therapeutics for cancer, metabolic disorders, cardiovascular conditions and autoimmune diseases.^3–5^ mRNA-based therapeutics work by translating exogenous mRNA into the target protein.^6^ mRNA drug substance is manufactured by *in vitro* transcription (IVT) using bacteriophage T7 polymerase, often incorporating N1-methylpseudouridine to form a nucleoside-modified messenger RNA (modRNA). modRNA drug substance is subsequently encapsulated with lipid nanoparticles (LNPs) for cellular delivery and protection from degradative forces. Formulated modRNA-LNPs comprise the drug product that is administered for vaccination. During the enzymatic manufacturing process of mRNA therapeutics, incomplete mRNA products can be generated in conjunction with other potential impurities such as double-stranded RNA.

Analytical methods are required to characterise mRNA therapeutics and underpin manufacturing development. Validated analytical methods are required to support the relevant phase of clinical development, regulatory submission requirements or to support ongoing quality control of the approved product. There is currently significant demand for improved analytical methods to characterise RNA therapeutics.

Liquid chromatography interfaced with tandem mass spectrometry (LC-MS/MS) has emerged as a powerful tool for the analysis and characterisation of mRNA vaccines and therapeutics. Recently a number of alternative workflows have been developed based on RNase mass mapping. mRNA sequence mapping using site-specific ribonucleases (RNases) have been developed to characterise the identity, sequence and chemical modifications of mRNA manufactured using IVT.^7–11^ Digestion of the mRNA has been performed using RNases such as RNase T1 in conjunction with alternative RNase enzymes, including MazF and human RNase 4 for RNase mass mapping approaches.^7,9,10^ The characterisation of large mRNA therapeutics/vaccines using LC–MS/MS is technically challenging and has been hindered by a lack of robust analytical and computational tools. Typical high frequency RNases including RNase T1 and RNase A generate short oligoribonucleotide fragments that do not uniquely match the mRNA sequence. In addition, enzymes such as *E. coli* interferase MazF generate large fragments that are typically difficult to confidently identify based on their MS/MS spectra. Therefore, novel mRNA sequence mapping approaches have been developed using partial T1 digests,^8^ parallel digestions using alternative RNases^7^ and alternative RNases such as human RNase 4,^9^ to overcome the above limitations and obtain high sequence coverage of the mRNA.

We have previously developed and utilised direct sequence mapping of mRNA using partial RNase T1 digests in conjunction with ion-pair reversed phase high performance liquid chromatography (IP-RP HPLC) coupled to mass spectrometry analysis.^8^ mRNA oligoribonucleotide identifications were performed using automated data analysis software, BioPharma Finder (BPF), which is able to identify oligoribonucleotides based on their accurate mass in conjunction with the MS/MS fragmentation spectra and map the corresponding oligoribonucleotide sequences to the known RNA sequence. Data analysis reveals that there are large numbers of (often overlapping), oligoribonucleotide fragments generated by the partial T1 digest that correspond to different numbers of missed cleavages. Data visualisation and sequence mapping methods have not previously taken advantage of the presence of multiple overlapping fragments, which provide additional confidence in assigning total sequence coverage in mRNA sequence mapping. Furthermore, the presence of multiple overlapping fragments may provide further insight into potential secondary/tertiary structures or reflect the accessibility of RNase to the mRNA. However, it should be noted that the primary mRNA sequence also influences the pattern and number of fragments generated with respect to the predicted theoretical fragments. In addition, previous mapping methods have not encompassed an ability to directly compare or combine complementary digests, for the purpose of developing sequencing workflows, improving sequence coverage or probing potential mRNA structure. Finally, the current manual method for data visualisation of sequence maps is laborious, time-consuming and error-prone.

Therefore, in an approach to optimise and streamline mRNA sequence mapping, whilst utilising the large numbers of multiple overlapping fragments and complementary RNase digests, novel software visualisation tools were developed to directly utilise the oligonucleotide identifications from multiple LC-MS/MS outputs. In this study, we have utilised partial RNase digests of mRNA using RNase T1 and RNase U2 in conjunction with automated, high throughput workflows for the rapid characterisation and direct sequence mapping of mRNA to improve sequence coverage, sequencing confidence and identity testing. The ability to rapidly identify, characterise and sequence map large mRNA therapeutics with high sequence coverage provides important information for identity testing, sequence validation, and impurity analysis.

## 2. Experimental

### 2.1. Chemicals

Water (UHPLC MS grade, Thermo Scientific), acetonitrile (UHPLC MS grade, Thermo Scientific), 1,1,1,3,3,3-hexafluoro-2-propanol (HFIP, >99.8% Fluka LC MS grade), Triethylamine (TEA, 99.7% extrapure Fisher Scientific). SMART Digest Bulk magnetic RNase T1 Kit, (Thermo Scientific), SMART Digest Bulk magnetic RNase U2 Kit, (Thermo Scientific).

### 2.2. IVT and purification of mRNA

mRNA synthesis *via* IVT was performed using linearized plasmid DNA (GenScript) at 2 × 10^−5^ mM, DNA-dependent RNA polymerase of T7 bacteriophage (Roche) and ATP, CTP, GTP and UTP (Roche) in an equimolar ratio at 10 mM concentration. The reaction was further supplemented with the standard reaction buffer recommended by the enzyme manufacturer. Inorganic pyrophosphatase (Roche) at 2.9 × 10^−3^ mM was added to the reaction mixture to prevent magnesium pyrophosphate precipitation. RNase inhibitor (Roche) was added at 2.1 × 10^−4^ mM to maintain an RNase-free environment in the reaction mixture. The reaction was incubated at 37 °C for 2 hours. Following IVT, template DNA was removed by the addition of DNase I and RNA was purified using silica columns as previously described.^23^ RNA concentrations were determined using a NanoDrop™ 2000c spectrophotometer (ThermoFisher Scientific) by absorbance at 260 nm normalised to a 1.0 cm (10.0 mm) path. mRNA encoding SARS-CoV-2 Spike protein (CSP) was prepared using a DNA template containing the open reading frame flanked by the 5′ and 3′ untranslated regions (UTR) and a poly(A) tail. Size and integrity of the mRNA was assessed using capillary electrophoresis (see ESI Fig. SF1†).

### 2.3. Partial RNase digests of mRNA

Partial RNase T1 digestion of CSP mRNA was performed using immobilised RNase T1 with the total reaction volume made up to 50 μL with SMART Digest buffer. The reaction was incubated at 37 °C and was terminated by the magnetic removal of the immobilised RNase. Details of mRNA quantity, RNase quantity and reaction time for different digest conditions are detailed in ESI Table ST1.†

Partial RNase U2 digestion of CSP mRNA was performed using 4 μL of immobilised RNase U2 and 40 μg of mRNA with the total reaction volume made up to 50 μL with SMART Digest buffer. The reaction was incubated at 37 °C for 30 minutes and was terminated by the magnetic removal of the immobilised RNase.

Automated RNase digests were performed using an automated robotic liquid handling system (KingFisher Duo Prime system, Thermo Scientific) using BindIt™ software (version 4.0) to control the system. A 96-deepwell plate was set up with 50 μl of SMART Digest buffer containing mRNA sample in row A and RNase T1/U2 immobilised on magnetic beads within 50 μl of SMART Digest buffer in row G. The KingFisher was programmed to transfer RNase immobilised magnetic particles to Row A to digest the RNA at 37 °C for the allotted time. Sedimentation of beads was prevented by repeated insertion of the magnetic comb using the mixing speed setting “Fast”. Immediately after incubation, the magnetic beads were collected and removed from the reaction.

### 2.4. LC-MS/MS analysis of mRNA digests

mRNA digest samples were analysed by IP-RP HPLC in conjunction with tandem mass spectrometry.

A Vanquish binary gradient UHPLC system (Thermo Fisher Scientific), using a DNAPac RP column (2.1 mm I.D. Thermo Fisher Scientific), was implemented for chromatography. Chromatograms were generated using UV detection at a wavelength of 260 nm.

The chromatographic analysis of RNase T1 and U2 digests was performed using the following conditions: buffer A 0.2% triethylamine (TEA) and 50 mM 1,1,1,3,3,3-hexafluoro-2-propanol (HFIP); buffer B 0.2% TEA, 50 mM HFIP, and 20% acetonitrile (ACN). RNA was analysed using a gradient that held at the starting percentage of buffer B for one minute, followed by a linear extension to the final percentage of buffer B. A flow rate of 0.2 mL min^−1^ and a temperature of 60 °C was used.

Chromatographically separated mRNA digests were interfaced with an Orbitrap Exploris 240 MS instrument (Thermo Fisher Scientific). Data was collected using data dependent acquisition in full scan negative mode with an MS1 resolution of 120 000 and a normalised automatic gain control (AGC) target of 200%. MS1 ions were selected for higher energy collisional dissociation (HCD). MS2 resolution was set at 30 000 with an AGC target of 100%, isolation window of 3 *m*/*z*, scan range of 150–2000 *m*/*z* and normalised stepped collision energies.

Additional details of the LC-MS/MS methods are supplied in ESI Table ST2.†

### 2.5. LC-MS/MS data analysis

Data analysis was performed in BioPharma Finder v5.2 (BPF, Thermo Fisher Scientific), using the Oligonucleotide Analysis module with ‘Enable Automatic Parameter Values’ selected for component detection. To identify large fragment ions, the maximum oligonucleotide mass was set to 30 000 Da, minimum confidence at 0.5 and mass accuracy at 10 ppm. The ribonuclease selection was set to RNase T1/RNase U2, default specificity (G-) was used for RNase T1 and “Custom Specificity” selected for RNase U2 with “A-,G-” inputted. The specificity level was set at “high”. The phosphate location was set at “none”, so 3′ OH termini was the default. Phosphorylation and cyclic phosphorylation were set as variable modifications of the 3′ terminal in the sequence manager containing the RNA sequence. Random RNA sequences of the same length and GC content were included in the sequence manager in addition to the correct RNA sequence, these were generated by the random sequence generator in the BPF sequence editor.

For data processing and review additional filters were included: “Identification” = “does not contain nonspecific”, “does not contain nonunique”; “Mod” = “does not contain None”; “Nonunique Seq” = “≤1”; “Δppm” = “≤20”, “≥−20”; “Conf. Score” = “≥90”; “Best ASR” = “≤2.0”; “ID Type” = “contains MS2”; “Mono Mass Exp.” = “>0”. All oligonucleotide identifications from BPF are shown in the ESI (Tables ST3–6†).

### 2.6. Generation of software

Automatic extraction of numerical data from sequence map images was executed *via* an in-house Python script. The script is available open source *via* GitHub at https://github.com/kesler20/sequence_matching. This script was also developed into an online software, which is available at https://wiz-app.up.railway.app/seqM.

One or two BPF sequence maps were loaded into the software in a png format, along with the total sequence length of the analysed mRNA construct. The information from the BPF images is extracted *via* image analysis. Analysis begins by reading and converting the BPF colour images into greyscale images using OpenCV. The images are then cropped to remove white margins, focusing on the relevant data sections. The software normalizes the images and converts them to a greyscale matrix, which is then processed to identify and sort fragment bars into their respective row positions.

A search algorithm detects fragment bars by examining pixel intensity patterns. Detected bars are indexed based on sequence position rather than pixel location. The colour and confidence level of each bar is extracted, with specific confidence values assigned according to predefined colour mappings. These values generate a comprehensive dataset, including the start, end, length of each fragment and its confidence level. This colour-coded information is automatically recorded, and cumulative confidence is calculated for each nucleotide in the mRNA sequence.

For a single BPF png input, a linear map and a single spiral plot are outputted. For a pair of BPF png images, a linear map is outputted alongside three spiral plots: one representing each of the individual images, and one representing their combination.

## 3. Results and discussion

### 3.1. Development of enhanced software visualisation tools for mRNA sequence mapping

**Fig. 1:**
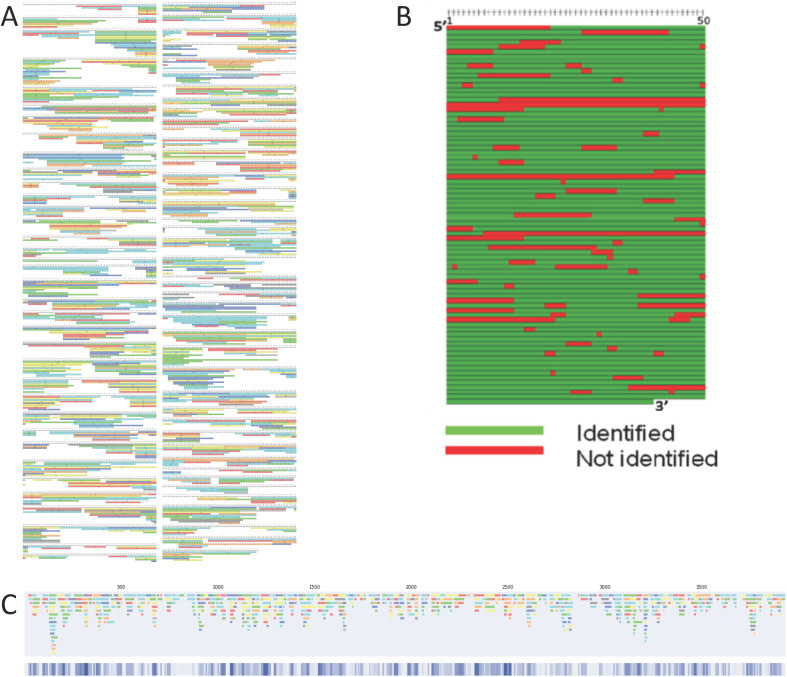
mRNA sequence maps obtained using LC-MS/MS and different visualisation tools. (A) Oligoribonucleotide identifications generated from a partial RNase T1 digest of mRNA are shown in an image map generated in BPF. (B) Manually generated sequence map using the oligoribonucleotide identifications. (C) Linear sequence map using automated software analysis directly from the BPF image.

mRNA sequence mapping using RNase digests prior to LC-MS/MS analysis and automated data analysis tools have been used in a number of different approaches.^7–11^ Commercially available software tools can be used to automatically generate images of the overlapping fragments identified by LC-MS/MS analysis from mRNA digests (see Fig. 1A). Despite providing high level information, this map style is complex, is very challenging to use in comparing RNase digests and does not allow for combining data sets to improve sequence coverage.

Corresponding mRNA sequence maps can be generated from such outputs. Fig. 1B shows the type of sequence map produced using our previous method. To generate a single map, each row of recorded sequences (from BPF) was transferred to a spreadsheet with conditional formatting. In each sequence location, where at least one oligoribonucleotide sequence (represented by one bar) is found in that position the cell is coloured green. Where no bar is found, the cell is recorded as red. This process is time-consuming and prone to error when transferring data across. Additionally, and crucially, note that the data recorded is in binary format, either bar(s) are found or they are not found. This can not easily be utilised in a high throughput fashion or where multiple data sets could be combined to generate a single sequence coverage map.

To address these issues, we have developed software tools that automate the extraction and visualisation of the oligoribonucleotide identifications from BPF software outputs, including the start, length and end of each fragment, to generate linear and spiral sequence maps. Moreover, where additional overlapping fragments are present we have used this information combined with the intensity-based confidence score provided with each oligoribonucleotide identification to generate a visual representation of the overall confidence in the sequence. Where multiple overlapping oligoribonucleotides have been identified within an mRNA sequence this is reflected in the mRNA sequence map. An example of one of our new linear sequence maps is shown in Fig. 1C. Through automation of this process, which manually can take up to an entire working day, our tool significantly reduces the analysis time to approximately 4–10 minutes per image (dependent on the number of identified oligoribonucleotides), enhancing the efficiency of RNA sequence mapping.

### 3.2. Generation of bespoke linear and spiral sequence maps

**Fig. 2:**
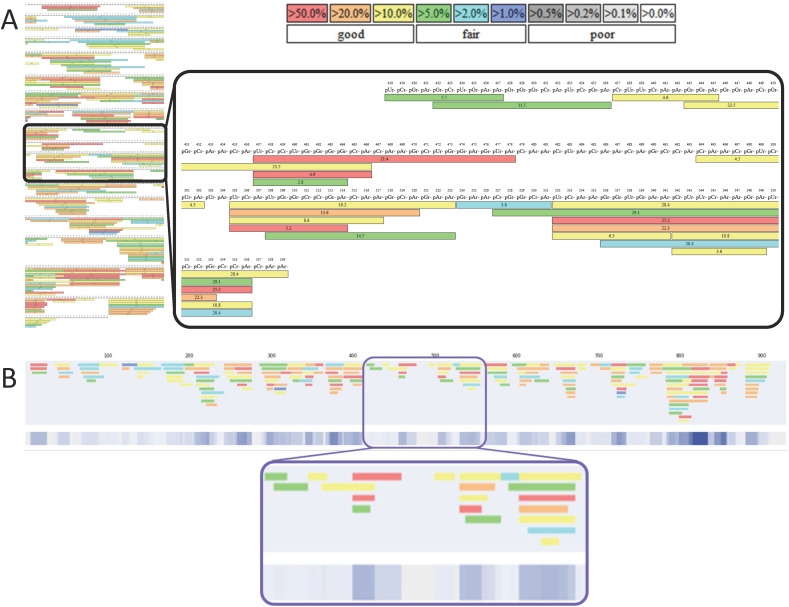
Generation of linear mRNA sequence maps using novel visualisation software. (A) mRNA sequence mapping image generated from LC-MS/MS analysis of a partial RNase T1 digest. Highlighted section shows the overlapping oligoribonucleotide fragments. The confidence colour coding used by BPF is shown in the legend. (B) Linear mRNA sequence map automatically generated from the image shown in Fig. 1A. The BPF confidence score based colour coding is translated into our linear map. A colourmetric representation of the overlapped fragments is shown beneath. Here, the intensity of the blue colour represents a combined confidence score, encompassing both the number of overlapping fragments and their individual confidences from the original BPF oligo identification. The same section of sequence highlighted in (A) is highlighted in (B).

Our first bespoke software tool generates linear maps by automatically extracting and presenting the identified oligoribonucleotide fragments from one or a set of complementary BPF sequence mapping images. In Fig. 2, we demonstrate how the software converts the BPF images into linear maps. The start, end, length and colour code/confidence level of each BPF identified fragment are all captured in the conversion. The linearised versions of the sequence maps are shown alongside the colourmetric representation of the overlapping fragments, which produces a single linear output, where colour intensity relates to the summation of the confidence scores of the overlapping fragments found for each nucleotide.

**Fig. 3:**
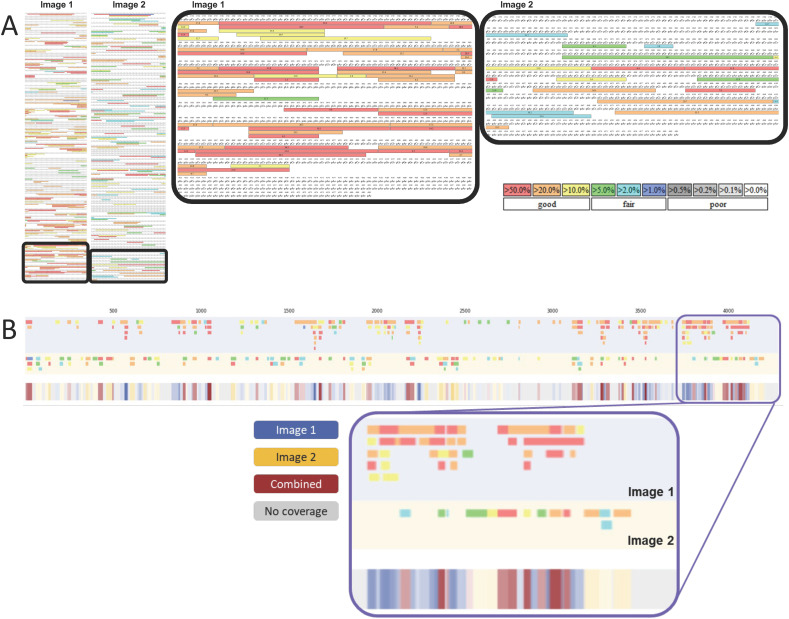
Generation of linear mRNA sequence maps in conjunction with combined LC-MS/MS analysis of complimentary RNase T1 digests. (A) Images show oligoribonucleotide identifications from the different RNase T1 partial digests. Highlighted sections are shown on the right. (B) mRNA sequence maps were generated directly from the BPF images using our automated extraction and visualisation software. Oligoribonucleotide fragments from image 1 are represented by blue, those from image 2 are represented by yellow and red represents regions with overlap between the two images. Grey is used to represent regions that are not mapped by any fragments. The same section of sequence highlighted in (A) is highlighted in (B).

When comparing and combining multiple RNase digests of the same mRNA, different colours are used to distinguish these, as shown in Fig. 3. A third colour is implemented into the colourmetric representation to highlight the sections of the sequence where oligoribonucleotide fragments are mapped in both of the digests. In this case, grey is also used for nucleotides that are not covered by either of the digests.

**Fig. 4:**
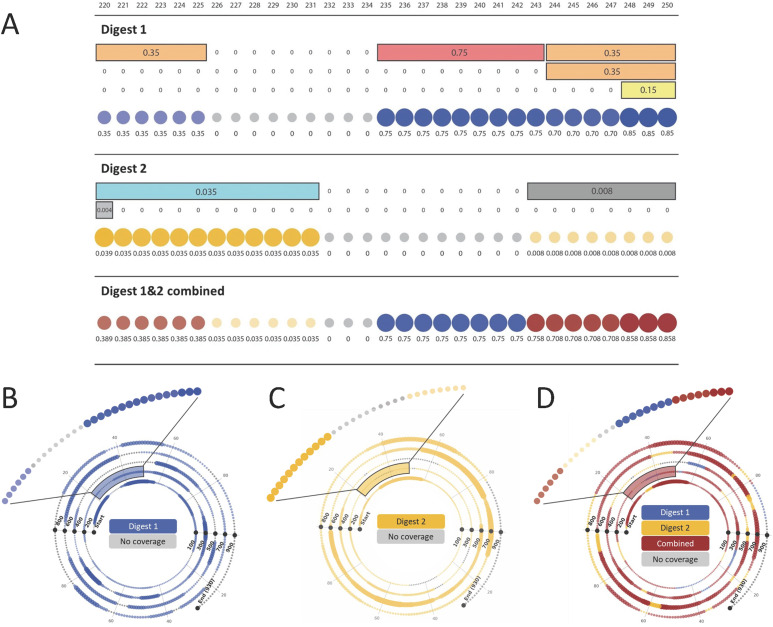
Spiral mRNA sequence coverage maps. (A) Synchronisation of BPF identified digest fragments between nucleotides 220 and 250, with each nucleotide being represented by a dot. The confidence of each identified fragment in the BPF output relates to the colour of the bar, the value is shown here. These are summed and shown beneath the sequence of circles, the size and opacity of which is related to this value. Red points show mutual coverage, blue points show digest 1 only, yellow points show digest 2 only and grey show no coverage. Spiral plots demonstrating sequence coverage of (B) digest 1, (C) digest 2 and (D) the combined output.

A complementary software visualisation tool was created to generate advanced spiral plots that display sequence maps both continuously and concisely, improving ease of interpretation and visualisation, and enabling superior presentation of the mRNA sequence maps. Fig. 4 shows how the sequence mapping data is visualised. Firstly, as with the linear maps, colour is used to distinguish each individual LC-MS/MS data set and their combination. However, in this case, each individual nucleotide in the mRNA sequence is represented by a dot. The confidence of each fragment that maps onto a specific nucleotide in the sequence is summed up, across either individual or multiple data sets, with the resulting value being represented by the size and opacity of the dot. Additionally, the percentage coverage for the combination of the sequence maps is automatically calculated and displayed.

### 3.3. Combining complementary RNase T1 partial digests for mRNA sequence mapping

Following the development of the novel software, which has been engineered to optimise and streamline visualisation and analysis of mRNA sequence mapping, we utilised the new software to rapidly combine data sets from multiple partial RNase T1 digestions into a single, consolidated mRNA sequence map.

**Fig. 5:**
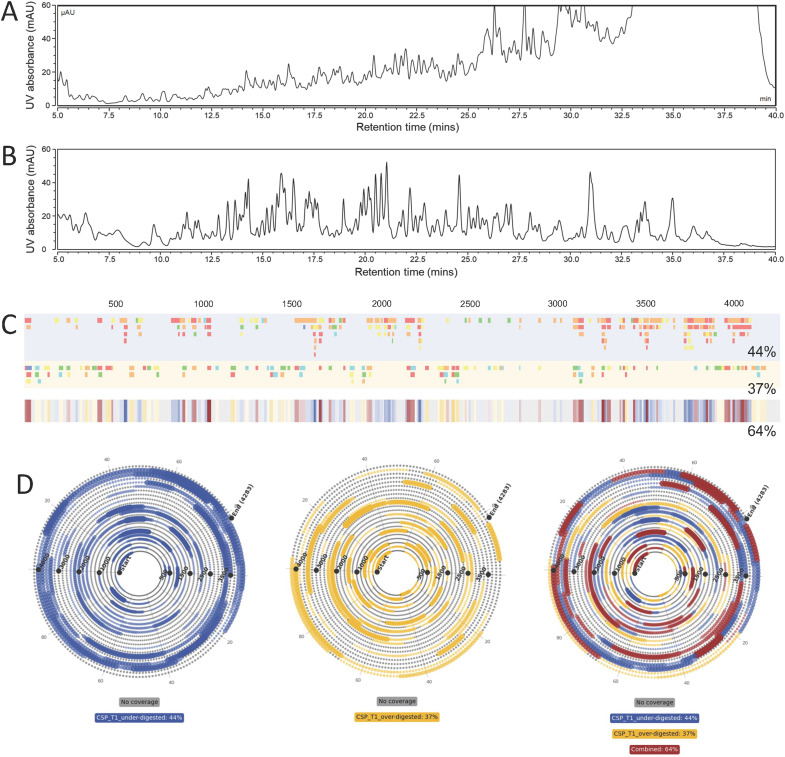
LC-MS/MS analysis of combined RNase T1 partial digests of mRNA. UV traces of RNase T1 partial digests of CSP mRNA: (A) under digested (1.25 μl immobilised T1, 10 minutes incubation) and (B) over digested (2.5 μl immobilised T1, 60 minutes incubation). (C) Linear mRNA sequence maps generated for the combined RNase T1 digests of CSP mRNA. The blue map contains fragments from the under digested sample and the yellow map contains fragments from the over digested sample. The percentage coverage of the mapped fragments is indicated for the individual digests and their combination. (D) Spiral mRNA sequence maps generated from the combined mRNA digests. Fragments from ‘under’ digested CSP mRNA are mapped in blue, ‘over’ digested fragments are mapped in yellow and overlapping fragments are mapped in red.

LC-MS/MS analysis of multiple partial RNase T1 digests of CSP mRNA (mRNA encoding SARS-CoV-2 Spike protein) was performed using varied digestion conditions (see Fig. 5). An application of this approach is demonstrated by the ability to rapidly combine data sets from multiple partial RNase T1 digestions into a single combined mRNA sequence map. To demonstrate proof of principle, two partial RNase T1 digests were performed under conditions that under-digested and over-digested a sample of CSP mRNA compared to an optimal partial RNase T1 digest (details in section 2.3). Following LC-MS/MS analysis (see Fig. 5A and B) it was evident that the digests produced a significant variation in oligoribonucleotide fragments from the chromatograms produced; the over-digested sample produced a spectrum of fragments of different sizes, leading to peaks across the range of retention time, whereas the under-digested sample shows a majority of fragments that elute towards the end of the gradient.

The linear sequence maps generated for the pair of partial RNase T1 digests are shown in Fig. 5C. The advantages of combining multiple partial RNase digests to enhance mRNA sequence coverage are clear, with unique oligoribonucleotide fragments mapped to the mRNA sequence identified in different partial RNase T1 digests. The results also demonstrate the increased confidence in coverage utilising multiple overlapping fragments generated from the complementary digests. Furthermore, the data visualisation tool enables simple identification of RNA fragments identified in each individual mRNA digest and the combined data sets.

In Fig. 5D we present the spiral plots produced for the partial RNase T1 digests of CSP mRNA. Despite the extra fragment information that is accessible from the linear maps, it is clear that the spiral plots offer a distinct advantage in data visualisation: they provide quick and easy access to an overview of the sequence coverage of individual and combined digests.

Both types of plot demonstrate that combining the different partial RNase digests significantly increases the overall sequence coverage and reduces regions of the mRNA where no oligonucleotides were identified. Individually the digests produce 44% and 37% sequence coverage, whereas in combination this is increased to 64%.

Further application of the combined partial RNase digests in conjunction with the sequence mapping visualisation tools was used for the analysis of chemically modified CSP mRNA. The combined partial RNase digests and sequence mapping of CSP mRNA containing N1-methylpseudouridine is shown in ESI Fig. SF2.† Increased sequence coverage from the combined sequence mapping was observed, consistent with previous analysis.

### 3.4. Combining RNase U2 and RNase T1 partial digests for mRNA sequence mapping

We have previously utilised and developed direct sequence mapping of mRNA using partial RNase T1 digests in conjunction with LC-MS/MS analysis where >80% sequence coverage was obtained based on only unique oligoribonucleotide fragments.^8^ It has been demonstrated that particular regions on the mRNA sequence generate large numbers of overlapping fragments whilst other regions are often only identified by a single oligoribonucleotide, due to the primary sequence of the mRNA (guanosine distribution) and the ability to produce oligoribonucleotide fragments of the ideal size for MS/MS identification. Furthermore, the secondary/tertiary structure is also likely to impact RNase digestion during partial RNase digests. Previous work showed that using long RNA with significant secondary structure elements resulted in a lower % sequence coverage.^8^ Therefore, further work was performed to develop a parallel, complementary partial RNase digest in order to map more challenging regions of mRNAs, particularly those that are larger and will have inherently great secondary structure, such as CSP mRNA. In this study, we chose to implement RNase U2, which is selective for purine nucleosides in mRNA, as our complementary enzyme for mRNA digestion.

**Fig. 6:**
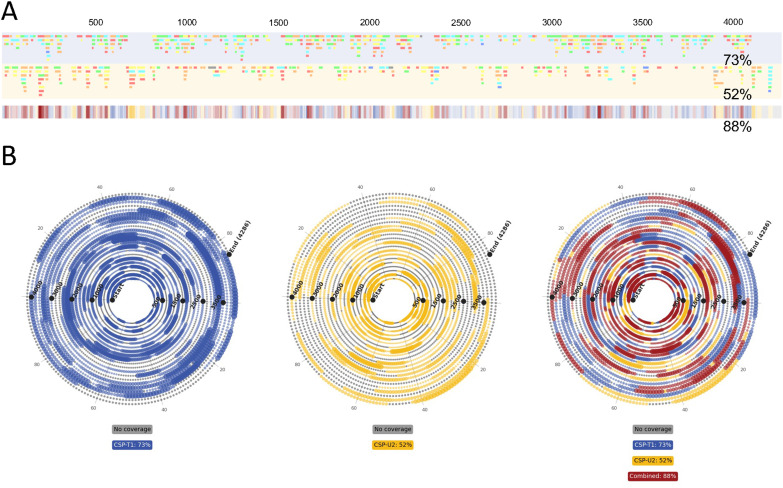
LC-MS/MS analysis of combined partial RNase T1 and RNase U2 digests of mRNA. (A) Linear sequence maps generated from the identified oligoribonucleotide fragments from these partial RNase T1 (blue) and partial RNase U2 (yellow) digests. (B) Spiral mRNA sequence maps generated from combined BPF outputs from the two CSP digests. Fragments generated by RNase T1 are mapped in blue, fragments generated by RNase U2 are mapped in yellow and overlapping fragments are mapped in red. In all, areas with no coverage are marked in grey.

The results of LC-MS/MS analysis of partial RNase T1 and RNase U2 digestion of CSP mRNA are shown in ESI Fig. SF3A and B,† respectively. The differences in the chromatograms produced by these digests demonstrate the differences in the oligoribonucleotide fragments generated by the RNases. The results of the differences in unique oligoribonucleotide identifications are even more evident in the linear sequence maps shown in Fig. 6A. Furthermore, the combined RNase sequence maps show that both digests map large portions of the mRNA sequence. However, the results show that gaps in the sequence coverage generated from the individual digests have been filled in the combined plot. This has led to significantly greater sequence coverage, based on unique oligoribonucleotide identifications, compared with the individual coverages (73% and 52% *versus* 88%).

In Fig. 6B we show the spiral plots produced for the partial RNase T1 and U2 digests of CSP mRNA. Consistent with previous analysis, the spiral plots and automatic sequence coverage calculations further demonstrates the benefit of combining multiple digests. Individual sequence coverages of 73% and 52% were found for the T1 and U2 digests respectively, whilst their combined sequence coverage was 88%.

## 4. Conclusions

In this study, we have utilised partial RNase digests of mRNA using RNase T1 and RNase U2 in conjunction with automated, high throughput LC-MS/MS workflows for the rapid characterisation and direct sequence mapping of mRNA vaccines and therapeutics. Moreover, we have developed a bespoke software engineered to optimise and streamline the visualisation and data analysis generated from the sequence mapping of mRNA using LC-MS/MS. The analytical workflow can be completed within 1.5–2.5 hours and data visualisation takes only 4–10 minutes per data set.

To achieve high sequence coverage of the mRNA based on only unique oligoribonucleotide identifications, novel data visualisation methods were developed that combine the sequence maps from multiple samples or combinations of multiple enzymatic digests. The advantages of combining multiple partial RNase digests to enhance mRNA sequence coverage was demonstrated in the analysis of combined partial RNase T1 digests (by altering the digest conditions) and combining partial RNase T1 and U2 digests.

Furthermore, the developed software provides additional detail by translating the information where multiple overlapping oligoribonucleotide fragments are identified to increase the confidence of the sequence mapping. The presence of multiple overlapping fragments may also provide insight into potential secondary/tertiary structure of mRNA, as well as RNase access or behaviour.

Finally, the software enables powerful visualization tools to generate spiral plots for enhanced characterisation of the mRNA sequence maps in a single output from combined multiple samples and multiple combined partial RNase digests.

## Author contributions

EW: Conceptualization, methodology, validation, investigation, formal analysis, data curation, visualization, writing – original draft preparation, review and editing. RC: Conceptualization, methodology, validation, visualization, software, writing – review and editing. GO: Conceptualization, methodology, investigation. CE: Conceptualization, methodology. KI: Visualization, software, writing – review and editing. KC: Conceptualization, review and editing. JC: Supervision, project administration, funding acquisition. ZK: Supervision, project administration, funding acquisition, writing – review and editing. PM: Supervision, project administration, funding acquisition, writing – review and editing. MD: Conceptualization, supervision, project administration, funding acquisition, writing – original draft preparation, review and editing. All authors contributed to the article and approved the submitted version.

## Data availability

Data supporting this article have been included as part of the ESI.† All other data is available on request. Software developed and utilised in this study is freely available online at https://wiz-app.up.railway.app/seqM.

## Conflicts of interest

Ken Cook is an employee of ThermoFisher and may hold shares and/or stock options in the company.

## Supplementary Material

AN-150-D5AN00033E-s001

AN-150-D5AN00033E-s002
