# Supplementary material for: Mass spectrometry-based mRNA sequence mapping via complementary RNase digests and bespoke visualisation tools
Source: Analyst. 2025 Jan 30;150(5):1012–21. doi: 10.1039/d5an00033e (PMC11809621; doi:10.1039/d5an00033e)
Supplement: AN-150-D5AN00033E-s001 [file AN-150-D5AN00033E-s001.pdf]

Supplementary Information (SI) for Analyst.
This journal is © The Royal Society of Chemistry 2025

Supplementary material

Mass spectrometry-based mRNA sequence mapping via complementary RNase digests and bespoke visualisation tools

Emma N. Welbourne[1], Royce J. Copley[1], Gareth R. Owen[1], Caroline A. Evans[1], Kesler Isoko[1,4], Ken Cook[2], Joan Cordiner[1], Zoltan Kis[1,3], Peyman Z. Moghadam[4], and Mark J. Dickman[1]*

[1] *School of Chemical, Materials and Biological Engineering, University of Sheffield, Sheffield, UK*

[2] *ThermoFisher Scientific, Hemel Hempstead, UK*

[3] *Department of Chemical Engineering, Imperial College London, London, UK*

[4] *Department of Chemical Engineering, University College London, London, UK*

*corresponding author email m.dickman@sheffield.ac.uk

Supplementary

Supplementary Table ST1: Reaction details (quantity of CSP mRNA, quantity of immobilised RNase T1, ratio of mRNA to RNase T1 and reaction time) for partial RNase T1 digests of CSP mRNA. The digest denoted 'middle' is that used in combination with the RNase U2 digest of CSP.

Digest	mRNA / µg	RNase / µL	mRNA:T1 / µg/µl	Time / min
Under	20	1.25	16	10
Over	20	2.5	8	60
Middle	80	2.5	32	30

Supplementary Table ST2: LC-MS/MS method details for the different types of mRNA digests.

RNase	Column length / mm	LC gradient / %B	Gradient time / min	MS1 scan range / m/z	Collision energies / %
T1 under	250	5-25	40	400-3000	15, 18, 21
T1 over	250	5-25	40	400-3000	15, 18, 21
T1 middle	250	3-21	60	450-2500	17, 20, 23
U2	100	0-21	60	400-3000	17, 20, 23
MazF	100	5-10-40	30	420-2500	17, 20, 23

Supplementary

A

B

Supplementary Figure SF1: Capillary electrophoretograms of (A) eGFP and (B) CSP mRNA. These confirm the expected sizes of the corresponding mRNAs–eGFP (~930 nt) and CSP (~4300 nt)–and the integrity of the mRNA synthesised.

Supplementary

A

500 1000 1500 2000 2500 3000 3500 4000

42%

79%

83%

B

Supplementary Figure SF2: LC-MS/MS analysis of combined RNase T1 partial digests of CSP mRNA with N1-methylpseudouridine incorporated. (A) Linear mRNA sequence map generated for the combined RNase T1 digests of CSP mRNA (2.5 µl immobilised T1, 10 or 40 minute incubations). The blue map contains fragments from the 10 minute digest sample and the yellow map contains fragments from the 40 minute digest sample. The percentage coverage of the mapped fragments is indicated for the individual digests and their combination. (B) Spiral mRNA sequence maps generated from the combined mRNA digests. Fragments from 10 minute digest are mapped in blue, 40 minute digest fragments are mapped in yellow and overlapping fragments are mapped in red.

Supplementary

A

B

Supplementary Figure SF3: LC-MS/MS analysis of RNase digests of mRNA. UV traces generated from partial (A) RNase T1 and (B) RNase U2 digests of CSP.
