# Supplementary material for: Mass spectrometry-based mRNA sequence mapping via complementary RNase digests and bespoke visualisation tools
Source: Analyst. 2025 Jan 30;150(5):1012–21. doi: 10.1039/d5an00033e (PMC11809621; doi:10.1039/d5an00033e)
Supplement: AN-150-D5AN00033E-s002 [file AN-150-D5AN00033E-s002.pdf]

Supplementary Information (SI) for Analyst.
This journal is © The Royal Society of Chemistry 2025

| Positions | Oligo Sequence | 3' Termini | Seq Length | Δ ppm | Conf. Score | Best ASR | RT | M/Z | Charge St. | Mono Mass Exp. | Avg Mass Exp. | Mono Mass Theo. | MS Area |
|---|---|---|---|---|---|---|---|---|---|---|---|---|---|
| 3878-3882 | Ar-pCr-pUr-pCr-pGr | Cyclic phosphorylation | 5 | 1.49 | 98.8 | 1.7 | 3.8 | 794.098 | -2 | 1590.2102 | 1590.82 | 1590.2078 | 572486.12 |
| 3878-3882 | Ar-pCr-pUr-pCr-pGr | Cyclic phosphorylation | 5 | 1.72 | 98.8 | 1 | 3.8 | 529.063 | -3 | 1590.2106 | 1590.76 | 1590.2078 | 280112.25 |
| 2808-2813 | Ar-pAr-pCr-pCr-pAr-pGr | Cyclic phosphorylation | 6 | 1.33 | 90.1 | 1 | 4.09 | 646.423 | -3 | 1942.2902 | 1943.22 | 1942.2876 | 1257378.62 |
| 1801-1808 | Ar-pAr-pAr-pUr-pCr-pCr-pUr-pGr | Cyclic phosphorylation | 8 | 1.13 | 95.6 | 1 | 4.24 | 850.44 | -3 | 2554.3411 | 2555.37 | 2554.3382 | 609617.75 |
| 1801-1808 | Ar-pAr-pAr-pUr-pCr-pCr-pUr-pGr | Cyclic phosphorylation | 8 | 1.32 | 95.6 | 1 | 4.25 | 637.578 | -4 | 2554.3416 | 2555.36 | 2554.3382 | 1268959.12 |
| 3516-3524 | Ur-pAr-pCr-pUr-pUr-pUr-pAr-pAr-pGr | Cyclic phosphorylation | 9 | 0.91 | 100 | 1 | 4.3 | 714.581 | -4 | 2861.3501 | 2862.48 | 2861.3475 | 1241382.5 |
| 3516-3524 | Ur-pAr-pCr-pUr-pUr-pUr-pAr-pAr-pGr | Cyclic phosphorylation | 9 | 1 | 100 | 1 | 4.3 | 953.11 | -3 | 2861.3503 | 2862.49 | 2861.3475 | 618573.44 |
| 3516-3524 | Ur-pAr-pCr-pUr-pUr-pUr-pAr-pAr-pGr | Cyclic phosphorylation | 9 | 1.59 | 100 | 1 | 4.33 | 571.464 | -5 | 2861.3521 | 2862.49 | 2861.3475 | 743171.88 |
| 2085-2092 | Ar-pCr-pAr-pAr-pAr-pCr-pAr-pGr | Cyclic phosphorylation | 8 | 1.4 | 99.3 | 1 | 4.5 | 649.342 | -4 | 2600.3962 | 2601.45 | 2600.3926 | 335677.94 |
| 3678-3686 | Ur-pAr-pCr-pAr-pUr-pCr-pAr-pAr-pGr | Cyclic phosphorylation | 9 | 0.99 | 99.9 | 1 | 4.61 | 960.458 | -3 | 2883.3936 | 2884.51 | 2883.3907 | 517254.88 |
| 3678-3686 | Ur-pAr-pCr-pAr-pUr-pCr-pAr-pAr-pGr | Cyclic phosphorylation | 9 | 1.16 | 99.9 | 1 | 4.61 | 720.092 | -4 | 2883.394 | 2884.49 | 2883.3907 | 941242.38 |
| 3678-3686 | Ur-pAr-pCr-pAr-pUr-pCr-pAr-pAr-pGr | Cyclic phosphorylation | 9 | 1.5 | 99.9 | 1 | 4.61 | 575.872 | -5 | 2883.395 | 2884.54 | 2883.3907 | 661852.19 |
| 4097-4105 | Cr-pAr-pAr-pUr-pAr-pAr-pAr-pCr-pGr | Cyclic phosphorylation | 9 | 0.69 | 99.9 | 1 | 4.69 | 725.848 | -4 | 2906.4199 | 2907.59 | 2906.4179 | 716399.56 |
| 4097-4105 | Cr-pAr-pAr-pUr-pAr-pAr-pAr-pCr-pGr | Cyclic phosphorylation | 9 | 1.36 | 99.9 | 1 | 4.69 | 580.478 | -5 | 2906.4219 | 2907.54 | 2906.4179 | 437515.12 |
| 1024-1032 | Ar-pAr-pUr-pCr-pCr-pAr-pUr-pCr-pGr | Cyclic phosphorylation | 9 | 0.49 | 98 | 1.5 | 4.71 | 952.454 | -3 | 2859.3809 | 2860.52 | 2859.3795 | 684205.25 |
| 1024-1032 | Ar-pAr-pUr-pCr-pCr-pAr-pUr-pCr-pGr | Cyclic phosphorylation | 9 | 0.57 | 98 | 1 | 4.71 | 714.089 | -4 | 2859.3811 | 2860.48 | 2859.3795 | 1233666.62 |
| 1024-1032 | Ar-pAr-pUr-pCr-pCr-pAr-pUr-pCr-pGr | Cyclic phosphorylation | 9 | 1.09 | 98 | 1 | 4.71 | 571.07 | -5 | 2859.3826 | 2860.5 | 2859.3795 | 1092189.5 |
| 3566-3573 | Ar-pAr-pUr-pCr-pAr-pAr-pUr-pGr | Cyclic phosphorylation | 8 | 0.65 | 96.3 | 1 | 4.74 | 643.831 | -4 | 2578.3511 | 2579.39 | 2578.3494 | 1079225.88 |
| 872-881 | Ar-pAr-pCr-pCr-pUr-pUr-pCr-pCr-pUr-pGr | Cyclic phosphorylation | 10 | 0.79 | 100 | 1 | 4.75 | 627.472 | -5 | 3141.396 | 3142.75 | 3141.3935 | 2464622.75 |
| 872-881 | Ar-pAr-pCr-pCr-pUr-pUr-pCr-pCr-pUr-pGr | Cyclic phosphorylation | 10 | 0.63 | 100 | 1 | 4.76 | 1046.459 | -3 | 3141.3955 | 3142.78 | 3141.3935 | 646863.12 |
| 872-881 | Ar-pAr-pCr-pCr-pUr-pUr-pCr-pCr-pUr-pGr | Cyclic phosphorylation | 10 | 0.71 | 100 | 1.1 | 4.76 | 784.592 | -4 | 3141.3958 | 3142.76 | 3141.3935 | 1365970 |
| 2235-2246 | Cr-pCr-pUr-pGr-pUr-pGr-pUr-pCr-pCr-pAr-pUr-pGr | Cyclic phosphorylation | 12 | 0.71 | 97.5 | 1.3 | 4.8 | 760.886 | -5 | 3808.4639 | 3810.05 | 3808.4612 | 780154.44 |
| 2235-2246 | Cr-pCr-pUr-pGr-pUr-pGr-pUr-pCr-pCr-pAr-pUr-pGr | Cyclic phosphorylation | 12 | 1.09 | 97.5 | 1 | 4.81 | 634.071 | -6 | 3808.4653 | 3810.2 | 3808.4612 | 1194545.5 |
| 1643-1653 | Cr-pAr-pCr-pCr-pAr-pAr-pUr-pCr-pUr-pCr-pGr | Cyclic phosphorylation | 11 | 1.44 | 100 | 1.2 | 4.92 | 866.61 | -4 | 3469.467 | 3470.92 | 3469.462 | 1166950.75 |
| 1643-1653 | Cr-pAr-pCr-pCr-pAr-pAr-pUr-pCr-pUr-pCr-pGr | Cyclic phosphorylation | 11 | 1.23 | 100 | 1.1 | 4.92 | 693.086 | -5 | 3469.4663 | 3470.93 | 3469.462 | 2070198.12 |
| 1643-1653 | Cr-pAr-pCr-pCr-pAr-pAr-pUr-pCr-pUr-pCr-pGr | Cyclic phosphorylation | 11 | 1.37 | 100 | 1.1 | 4.92 | 577.404 | -6 | 3469.4668 | 3470.94 | 3469.462 | 824472.94 |
| 4110-4119 | Ur-pUr-pUr-pAr-pAr-pCr-pUr-pAr-pAr-pGr | Cyclic phosphorylation | 10 | 1.04 | 99.5 | 1 | 5.12 | 637.274 | -5 | 3190.4033 | 3191.61 | 3190.4 | 618886.25 |
| 3585-3593 | Ar-pAr-pCr-pAr-pUr-pCr-pCr-pAr-pGr | Cyclic phosphorylation | 9 | 0.95 | 99.9 | 1 | 5.17 | 575.675 | -5 | 2882.4094 | 2883.74 | 2882.4067 | 2458787.75 |
| 1226-1236 | Cr-pUr-pUr-pCr-pAr-pCr-pAr-pAr-pAr-pCr-pGr | Cyclic phosphorylation | 11 | 1.01 | 96 | 1 | 5.19 | 697.888 | -5 | 3493.4768 | 3494.96 | 3493.4733 | 700813.44 |
| 3585-3593 | Ar-pAr-pCr-pAr-pUr-pCr-pCr-pAr-pGr | Cyclic phosphorylation | 9 | 0.53 | 99.9 | 1.1 | 5.19 | 960.129 | -3 | 2882.4082 | 2883.57 | 2882.4067 | 1195631.62 |
| 3585-3593 | Ar-pAr-pCr-pAr-pUr-pCr-pCr-pAr-pGr | Cyclic phosphorylation | 9 | 0.28 | 99.9 | 1 | 5.19 | 719.845 | -4 | 2882.4075 | 2883.56 | 2882.4067 | 2785917 |
| 3846-3857 | Cr-pUr-pGr-pAr-pAr-pGr-pGr-pGr-pCr-pGr-pUr-pGr | Cyclic phosphorylation | 12 | 0.59 | 100 | 1.3 | 5.21 | 789.298 | -5 | 3950.5251 | 3952.11 | 3950.5228 | 694397.06 |
| 3846-3857 | Cr-pUr-pGr-pAr-pAr-pGr-pGr-pGr-pCr-pGr-pUr-pGr | Cyclic phosphorylation | 12 | 1.14 | 100 | 1 | 5.21 | 657.581 | -6 | 3950.5273 | 3952.12 | 3950.5228 | 1357080.12 |
| 3375-3384 | Ar-pAr-pCr-pUr-pUr-pCr-pUr-pAr-pCr-pGr | Cyclic phosphorylation | 10 | 0.78 | 100 | 1 | 5.28 | 632.275 | -5 | 3165.4072 | 3166.78 | 3165.4048 | 1826580.25 |
| 3375-3384 | Ar-pAr-pCr-pUr-pUr-pCr-pUr-pAr-pCr-pGr | Cyclic phosphorylation | 10 | 0.93 | 100 | 1 | 5.29 | 1054.463 | -3 | 3165.4077 | 3166.58 | 3165.4048 | 549620.88 |
| 3375-3384 | Ar-pAr-pCr-pUr-pUr-pCr-pUr-pAr-pCr-pGr | Cyclic phosphorylation | 10 | 0.78 | 100 | 1 | 5.29 | 790.595 | -4 | 3165.4072 | 3166.63 | 3165.4048 | 1329666.12 |
| 2520-2529 | Ur-pUr-pCr-pAr-pAr-pCr-pAr-pAr-pAr-pGr | Cyclic phosphorylation | 10 | 1.26 | 92 | 1.1 | 5.33 | 641.682 | -5 | 3212.4473 | 3213.69 | 3212.4432 | 1038097.81 |
| 2520-2529 | Ur-pUr-pCr-pAr-pAr-pCr-pAr-pAr-pAr-pGr | Cyclic phosphorylation | 10 | 0.81 | 92 | 1.2 | 5.33 | 802.355 | -4 | 3212.4458 | 3213.75 | 3212.4432 | 398941.25 |
| 3786-3796 | Ar-pAr-pGr-pGr-pGr-pCr-pUr-pGr-pUr-pUr-pGr | Cyclic phosphorylation | 11 | 1.17 | 100 | 1 | 5.38 | 720.486 | -5 | 3606.4636 | 3607.99 | 3606.4594 | 705979.31 |
| 3585-3593 | Ar-pAr-pCr-pAr-pUr-pCr-pCr-pAr-pGr | Phosphorylation | 9 | 1.01 | 95.6 | 1 | 5.4 | 724.348 | -4 | 2900.4202 | 2901.56 | 2900.4172 | 742660.69 |
| 3786-3796 | Ar-pAr-pGr-pGr-pGr-pCr-pUr-pGr-pUr-pUr-pGr | Cyclic phosphorylation | 11 | 1.58 | 100 | 1 | 5.4 | 600.237 | -6 | 3606.4651 | 3607.96 | 3606.4594 | 1049777.12 |
| 3864-3874 | Cr-pAr-pCr-pUr-pAr-pCr-pAr-pCr-pAr-pUr-pGr | Cyclic phosphorylation | 11 | 0.73 | 100 | 1.2 | 5.48 | 872.611 | -4 | 3493.4758 | 3495.08 | 3493.4733 | 2374774.25 |
| 3864-3874 | Cr-pAr-pCr-pUr-pAr-pCr-pAr-pCr-pAr-pUr-pGr | Cyclic phosphorylation | 11 | 0.8 | 100 | 1.1 | 5.48 | 1163.818 | -3 | 3493.4761 | 3495.04 | 3493.4733 | 1242704.75 |
| 3864-3874 | Cr-pAr-pCr-pUr-pAr-pCr-pAr-pCr-pAr-pUr-pGr | Cyclic phosphorylation | 11 | 0.18 | 100 | 1 | 5.48 | 697.887 | -5 | 3493.4739 | 3495.07 | 3493.4733 | 4792377.5 |
| 2520-2529 | Ur-pUr-pCr-pAr-pAr-pCr-pAr-pAr-pAr-pGr | Phosphorylation | 10 | 3.05 | 94.7 | 1 | 5.5 | 645.285 | -5 | 3230.4636 | 3231.87 | 3230.4538 | 239609.39 |
| 3286-3298 | Cr-pUr-pCr-pCr-pAr-pGr-pCr-pCr-pAr-pUr-pCr-pUr-pGr | Cyclic phosphorylation | 13 | 1.17 | 100 | 1.9 | 5.5 | 818.499 | -5 | 4096.5283 | 4098.2 | 4096.5235 | 609068.88 |
| 3286-3298 | Cr-pUr-pCr-pCr-pAr-pGr-pCr-pCr-pAr-pUr-pCr-pUr-pGr | Cyclic phosphorylation | 13 | 1.29 | 100 | 1 | 5.5 | 681.914 | -6 | 4096.5288 | 4098.2 | 4096.5235 | 1111699.75 |
| 3864-3874 | Cr-pAr-pCr-pUr-pAr-pCr-pAr-pCr-pAr-pUr-pGr | Cyclic phosphorylation | 11 | 1.64 | 100 | 1 | 5.5 | 581.405 | -6 | 3493.479 | 3495.19 | 3493.4733 | 1689644.5 |
| 2496-2508 | Cr-pGr-pGr-pAr-pGr-pCr-pUr-pUr-pCr-pAr-pUr-pCr-pGr | Cyclic phosphorylation | 13 | 3.35 | 98.8 | 1.6 | 5.64 | 595.785 | -7 | 4176.5498 | 4178.16 | 4176.5358 | 54694 |
| 2496-2508 | Cr-pGr-pGr-pAr-pGr-pCr-pUr-pUr-pCr-pAr-pUr-pCr-pGr | Cyclic phosphorylation | 13 | 2.18 | 98.8 | 1.2 | 5.64 | 695.251 | -6 | 4176.5449 | 4178.33 | 4176.5358 | 243338.81 |
| 1582-1594 | Ur-pGr-pGr-pUr-pGr-pGr-pUr-pGr-pCr-pUr-pGr-pAr-pGr | Cyclic phosphorylation | 13 | 1.44 | 92.1 | 1.2 | 5.67 | 711.416 | -6 | 4273.5332 | 4275.19 | 4273.5271 | 88566.27 |
| 3616-3626 | Ar-pGr-pGr-pUr-pGr-pGr-pCr-pCr-pAr-pAr-pGr | Cyclic phosphorylation | 11 | 1.91 | 97.9 | 1.1 | 5.71 | 724.895 | -5 | 3628.5095 | 3630 | 3628.5026 | 342108.19 |
| 3616-3626 | Ar-pGr-pGr-pUr-pGr-pGr-pCr-pCr-pAr-pAr-pGr | Cyclic phosphorylation | 11 | 2.78 | 97.9 | 1.8 | 5.71 | 603.912 | -6 | 3628.5127 | 3630.01 | 3628.5026 | 272383.78 |
| 2041-2052 | Ar-pCr-pAr-pUr-pCr-pCr-pCr-pCr-pAr-pUr-pCr-pGr | Cyclic phosphorylation | 12 | 1.84 | 98.4 | 1.1 | 5.73 | 754.095 | -5 | 3774.5103 | 3776.05 | 3774.5033 | 486455.12 |
| 2041-2052 | Ar-pCr-pAr-pUr-pCr-pCr-pCr-pCr-pAr-pUr-pCr-pGr | Cyclic phosphorylation | 12 | 2.29 | 98.4 | 1.2 | 5.73 | 628.245 | -6 | 3774.512 | 3775.99 | 3774.5033 | 548519.12 |
| 1941-1951 | Ar-pCr-pAr-pCr-pCr-pUr-pAr-pCr-pAr-pUr-pGr | Cyclic phosphorylation | 11 | 1.99 | 99.8 | 1 | 6.09 | 581.406 | -6 | 3493.4802 | 3494.93 | 3493.4733 | 708455 |
| 1941-1951 | Ar-pCr-pAr-pCr-pCr-pUr-pAr-pCr-pAr-pUr-pGr | Cyclic phosphorylation | 11 | 1.29 | 99.8 | 1 | 6.11 | 697.889 | -5 | 3493.4778 | 3494.95 | 3493.4733 | 820458.56 |
| 1941-1951 | Ar-pCr-pAr-pCr-pCr-pUr-pAr-pCr-pAr-pUr-pGr | Cyclic phosphorylation | 11 | 1.5 | 99.8 | 1 | 6.11 | 872.613 | -4 | 3493.4785 | 3495.04 | 3493.4733 | 323555.09 |
| 928-941 | Cr-pUr-pCr-pUr-pGr-pGr-pAr-pUr-pCr-pCr-pUr-pCr-pUr-pGr | Cyclic phosphorylation | 14 | 2.11 | 99.7 | 1.3 | 6.13 | 630.641 | -7 | 4419.5371 | 4421.45 | 4419.5278 | 289150.44 |
| 928-941 | Cr-pUr-pCr-pUr-pGr-pGr-pAr-pUr-pCr-pCr-pUr-pCr-pUr-pGr | Cyclic phosphorylation | 14 | 2.33 | 99.7 | 1.1 | 6.13 | 735.749 | -6 | 4419.5381 | 4421.33 | 4419.5278 | 363499.31 |
| 3885-3897 | Cr-pUr-pGr-pGr-pUr-pAr-pCr-pUr-pGr-pCr-pAr-pUr-pGr | Cyclic phosphorylation | 13 | 2.61 | 99.9 | 1.2 | 6.22 | 695.415 | -6 | 4177.5308 | 4179.15 | 4177.5198 | 746347.81 |
| 3885-3897 | Cr-pUr-pGr-pGr-pUr-pAr-pCr-pUr-pGr-pCr-pAr-pUr-pGr | Cyclic phosphorylation | 13 | 2.5 | 99.9 | 1.6 | 6.22 | 595.926 | -7 | 4177.5303 | 4179.21 | 4177.5198 | 458593.38 |
| 3441-3452 | Ar-pUr-pCr-pGr-pGr-pCr-pAr-pUr-pUr-pGr-pUr-pGr | Cyclic phosphorylation | 12 | 1.5 | 97.4 | 1 | 6.43 | 644.574 | -6 | 3872.4844 | 3874.1 | 3872.4786 | 514899.16 |
| 1991-2003 | Ar-pGr-pCr-pCr-pGr-pGr-pCr-pUr-pGr-pUr-pCr-pUr-pGr | Cyclic phosphorylation | 13 | 1.05 | 99.9 | 1 | 6.46 | 697.916 | -6 | 4192.5352 | 4194.27 | 4192.5307 | 617460.31 |
| 567-581 | Ur-pCr-pCr-pCr-pAr-pGr-pCr-pCr-pUr-pUr-pUr-pCr-pCr-pUr-pG | Cyclic phosphorylation | 15 | 1.58 | 100 | 1.7 | 6.48 | 584.815 | -8 | 4684.5703 | 4686.57 | 4684.5629 | 562722.81 |
| 567-581 | Ur-pCr-pCr-pCr-pAr-pGr-pCr-pCr-pUr-pUr-pUr-pCr-pCr-pUr-pG | Cyclic phosphorylation | 15 | 0.96 | 100 | 1 | 6.49 | 668.36 | -7 | 4684.5674 | 4686.54 | 4684.5629 | 1659992.12 |
| 567-581 | Ur-pCr-pCr-pCr-pAr-pGr-pCr-pCr-pUr-pUr-pUr-pCr-pCr-pUr-pG | Cyclic phosphorylation | 15 | 0.75 | 100 | 1.2 | 6.5 | 779.921 | -6 | 4684.5664 | 4686.56 | 4684.5629 | 755647.56 |
| 4013-4024 | Ar-pCr-pAr-pCr-pCr-pUr-pCr-pCr-pCr-pAr-pAr-pGr | Cyclic phosphorylation | 12 | 0.96 | 100 | 1.2 | 6.53 | 1265.171 | -3 | 3797.5342 | 3799.21 | 3797.5305 | 444471.66 |
| 4013-4024 | Ar-pCr-pAr-pCr-pCr-pUr-pCr-pCr-pCr-pAr-pAr-pGr | Cyclic phosphorylation | 12 | 0.96 | 100 | 1 | 6.53 | 758.7 | -5 | 3797.5342 | 3799.2 | 3797.5305 | 1339680.62 |
| 4013-4024 | Ar-pCr-pAr-pCr-pCr-pUr-pCr-pCr-pCr-pAr-pAr-pGr | Cyclic phosphorylation | 12 | 0.77 | 100 | 2 | 6.53 | 948.627 | -4 | 3797.5334 | 3799.21 | 3797.5305 | 551860.06 |
| 4013-4024 | Ar-pCr-pAr-pCr-pCr-pUr-pCr-pCr-pCr-pAr-pAr-pGr | Cyclic phosphorylation | 12 | 1.47 | 100 | 1.1 | 6.56 | 632.082 | -6 | 3797.5361 | 3799.18 | 3797.5305 | 1784512.88 |
| 3038-3048 | Ar-pCr-pUr-pGr-pAr-pUr-pCr-pAr-pCr-pAr-pGr | Cyclic phosphorylation | 11 | 1.68 | 99.8 | 1 | 6.7 | 588.074 | -6 | 3533.4854 | 3535 | 3533.4794 | 555247.69 |
| 3038-3048 | Ar-pCr-pUr-pGr-pAr-pUr-pCr-pAr-pCr-pAr-pGr | Cyclic phosphorylation | 11 | 1.2 | 99.8 | 1 | 6.7 | 705.89 | -5 | 3533.4836 | 3534.95 | 3533.4794 | 562975.75 |
| 495-503 | Ar-pAr-pCr-pAr-pAr-pCr-pAr-pAr-pGr | Cyclic phosphorylation | 9 | 0.81 | 99.9 | 1 | 6.71 | 731.605 | -4 | 2929.4475 | 2930.59 | 2929.4451 | 1514710.88 |
| 495-503 | Ar-pAr-pCr-pAr-pAr-pCr-pAr-pAr-pGr | Cyclic phosphorylation | 9 | 0.81 | 99.9 | 1 | 6.71 | 975.81 | -3 | 2929.4475 | 2930.59 | 2929.4451 | 562222.12 |
| 495-503 | Ar-pAr-pCr-pAr-pAr-pCr-pAr-pAr-pGr | Cyclic phosphorylation | 9 | 0.73 | 99.9 | 1.3 | 6.72 | 1464.217 | -2 | 2929.4473 | 2930.63 | 2929.4451 | 317046.84 |
| 495-503 | Ar-pAr-pCr-pAr-pAr-pCr-pAr-pAr-pGr | Cyclic phosphorylation | 9 | 1.23 | 99.9 | 1 | 6.72 | 585.083 | -5 | 2929.4487 | 2930.6 | 2929.4451 | 1297501 |
| 1624-1637 | Ur-pGr-pUr-pGr-pCr-pGr-pGr-pCr-pCr-pCr-pUr-pAr-pAr-pGr | Cyclic phosphorylation | 14 | 1.89 | 99.8 | 1.2 | 6.84 | 645.221 | -7 | 4521.5918 | 4523.54 | 4521.5833 | 601583.5 |
| 1624-1637 | Ur-pGr-pUr-pGr-pCr-pGr-pGr-pCr-pCr-pCr-pUr-pAr-pAr-pGr | Cyclic phosphorylation | 14 | 1.45 | 99.8 | 1 | 6.84 | 752.758 | -6 | 4521.5898 | 4523.53 | 4521.5833 | 746921.62 |
| 1643-1655 | Cr-pAr-pCr-pCr-pAr-pAr-pUr-pCr-pUr-pCr-pGr-pUr-pGr | Cyclic phosphorylation | 13 | 1.87 | 99.8 | 1.2 | 6.91 | 587.785 | -7 | 4120.5425 | 4122.31 | 4120.5348 | 279642.66 |
| 1643-1655 | Cr-pAr-pCr-pCr-pAr-pAr-pUr-pCr-pUr-pCr-pGr-pUr-pGr | Cyclic phosphorylation | 13 | 1.63 | 99.8 | 1 | 6.91 | 685.916 | -6 | 4120.5415 | 4122.2 | 4120.5348 | 838103.75 |
| 1643-1655 | Cr-pAr-pCr-pCr-pAr-pAr-pUr-pCr-pUr-pCr-pGr-pUr-pGr | Cyclic phosphorylation | 13 | 1.63 | 99.8 | 1.4 | 6.91 | 823.301 | -5 | 4120.5415 | 4122.2 | 4120.5348 | 411426.62 |
| 1810-1822 | Ar-pCr-pAr-pUr-pCr-pAr-pCr-pCr-pCr-pCr-pUr-pUr-pGr | Cyclic phosphorylation | 13 | 0.88 | 100 | 1.2 | 6.91 | 1359.505 | -3 | 4080.5322 | 4082.22 | 4080.5286 | 343261.16 |
| 1810-1822 | Ar-pCr-pAr-pUr-pCr-pAr-pCr-pCr-pCr-pCr-pUr-pUr-pGr | Cyclic phosphorylation | 13 | 1.84 | 100 | 1.2 | 6.91 | 582.07 | -7 | 4080.5361 | 4082.15 | 4080.5286 | 1022031.44 |
| 1810-1822 | Ar-pCr-pAr-pUr-pCr-pAr-pCr-pCr-pCr-pCr-pUr-pUr-pGr | Cyclic phosphorylation | 13 | 1.12 | 100 | 1.2 | 6.91 | 679.248 | -6 | 4080.5332 | 4082.18 | 4080.5286 | 1751064.62 |
| 1810-1822 | Ar-pCr-pAr-pUr-pCr-pAr-pCr-pCr-pCr-pCr-pUr-pUr-pGr | Cyclic phosphorylation | 13 | 1.24 | 100 | 1.4 | 6.91 | 1019.377 | -4 | 4080.5337 | 4082.23 | 4080.5286 | 281871.78 |
| 1810-1822 | Ar-pCr-pAr-pUr-pCr-pAr-pCr-pCr-pCr-pCr-pUr-pUr-pGr | Cyclic phosphorylation | 13 | 1.54 | 100 | 1.4 | 6.91 | 815.3 | -5 | 4080.5349 | 4082.23 | 4080.5286 | 788247.56 |
| 3453-3462 | Ar-pAr-pCr-pAr-pAr-pUr-pAr-pCr-pCr-pGr | Cyclic phosphorylation | 10 | 1.45 | 100 | 1.1 | 7.25 | 534.404 | -6 | 3211.4639 | 3212.85 | 3211.4592 | 410137.06 |
| 3453-3462 | Ar-pAr-pCr-pAr-pAr-pUr-pAr-pCr-pCr-pGr | Cyclic phosphorylation | 10 | 0.31 | 100 | 1 | 7.25 | 641.485 | -5 | 3211.4602 | 3212.71 | 3211.4592 | 2325984.75 |
| 3453-3462 | Ar-pAr-pCr-pAr-pAr-pUr-pAr-pCr-pCr-pGr | Cyclic phosphorylation | 10 | 0.85 | 100 | 1 | 7.25 | 802.109 | -4 | 3211.4619 | 3212.83 | 3211.4592 | 1180395.75 |
| 3453-3462 | Ar-pAr-pCr-pAr-pAr-pUr-pAr-pCr-pCr-pGr | Cyclic phosphorylation | 10 | 1.07 | 100 | 1.1 | 7.25 | 1069.815 | -3 | 3211.4626 | 3212.83 | 3211.4592 | 582909.25 |
| 3453-3462 | Ar-pAr-pCr-pAr-pAr-pUr-pAr-pCr-pCr-pGr | Cyclic phosphorylation | 10 | 1.45 | 100 | 1.2 | 7.26 | 1605.226 | -2 | 3211.4639 | 3212.86 | 3211.4592 | 246425.22 |
| 2100-2110 | Ar-pGr-pAr-pGr-pCr-pCr-pAr-pGr-pAr-pAr-pGr | Cyclic phosphorylation | 11 | 2.75 | 90.8 | 1.1 | 7.73 | 726.302 | -5 | 3635.5449 | 3637.05 | 3635.5349 | 203474.75 |
| 2060-2071 | Ar-pAr-pUr-pCr-pUr-pGr-pCr-pGr-pCr-pCr-pAr-pGr | Cyclic phosphorylation | 12 | 1.78 | 100 | 1.1 | 8.03 | 641.58 | -6 | 3854.5225 | 3856.13 | 3854.5156 | 1278704.12 |
| 2060-2071 | Ar-pAr-pUr-pCr-pUr-pGr-pCr-pGr-pCr-pCr-pAr-pGr | Cyclic phosphorylation | 12 | 1.78 | 100 | 1.1 | 8.03 | 1284.168 | -3 | 3854.5225 | 3856.11 | 3854.5156 | 257314.44 |
| 2060-2071 | Ar-pAr-pUr-pCr-pUr-pGr-pCr-pGr-pCr-pCr-pAr-pGr | Cyclic phosphorylation | 12 | 1.59 | 100 | 1.2 | 8.05 | 770.098 | -5 | 3854.5217 | 3856.12 | 3854.5156 | 678014.5 |
| 2060-2071 | Ar-pAr-pUr-pCr-pUr-pGr-pCr-pGr-pCr-pCr-pAr-pGr | Cyclic phosphorylation | 12 | 1.71 | 100 | 1.7 | 8.05 | 962.874 | -4 | 3854.5222 | 3856.14 | 3854.5156 | 236921.67 |
| 2247-2257 | Ar-pCr-pCr-pAr-pAr-pGr-pAr-pCr-pCr-pAr-pGr | Cyclic phosphorylation | 11 | 2.02 | 99.6 | 1.2 | 8.05 | 710.299 | -5 | 3555.5298 | 3557.03 | 3555.5226 | 414837.91 |
| 2247-2257 | Ar-pCr-pCr-pAr-pAr-pGr-pAr-pCr-pCr-pAr-pGr | Cyclic phosphorylation | 11 | 1.6 | 99.6 | 1.2 | 8.06 | 888.126 | -4 | 3555.5283 | 3556.98 | 3555.5226 | 140838.97 |
| 2247-2257 | Ar-pCr-pCr-pAr-pAr-pGr-pAr-pCr-pCr-pAr-pGr | Cyclic phosphorylation | 11 | 2.43 | 99.6 | 1.2 | 8.07 | 591.748 | -6 | 3555.5312 | 3556.99 | 3555.5226 | 428225.34 |
| 3002-3015 | Ar-pCr-pUr-pGr-pGr-pAr-pCr-pCr-pCr-pUr-pCr-pCr-pUr-pGr | Cyclic phosphorylation | 14 | 1.83 | 99.9 | 1.6 | 8.76 | 633.647 | -7 | 4441.5791 | 4443.54 | 4441.571 | 594003.25 |
| 3002-3015 | Ar-pCr-pUr-pGr-pGr-pAr-pCr-pCr-pCr-pUr-pCr-pCr-pUr-pGr | Cyclic phosphorylation | 14 | 2.05 | 99.9 | 1.3 | 8.77 | 739.423 | -6 | 4441.5801 | 4443.54 | 4441.571 | 483072.12 |
| 3969-3984 | Cr-pUr-pCr-pCr-pCr-pAr-pCr-pCr-pUr-pCr-pCr-pAr-pCr-pCr-pUr- | Cyclic phosphorylation | 16 | 1.45 | 100 | 1.6 | 8.99 | 827.935 | -6 | 4971.6484 | 4973.79 | 4971.6412 | 892926.88 |
| 3969-3984 | Cr-pUr-pCr-pCr-pCr-pAr-pCr-pCr-pUr-pCr-pCr-pAr-pCr-pCr-pUr- | Cyclic phosphorylation | 16 | 1.35 | 100 | 1.1 | 8.99 | 709.514 | -7 | 4971.6479 | 4973.74 | 4971.6412 | 1959769.88 |
| 3249-3261 | Ar-pCr-pAr-pUr-pAr-pUr-pGr-pUr-pGr-pCr-pCr-pCr-pGr | Cyclic phosphorylation | 13 | -1.85 | 99.4 | 1.3 | 9 | 831.503 | -5 | 4160.5332 | 4162.51 | 4160.5409 | 270933.03 |
| 3249-3261 | Ar-pCr-pAr-pUr-pAr-pUr-pGr-pUr-pGr-pCr-pCr-pCr-pGr | Cyclic phosphorylation | 13 | 1.43 | 99.4 | 1 | 9.01 | 692.584 | -6 | 4160.5469 | 4162.22 | 4160.5409 | 745710.56 |
| 3249-3261 | Ar-pCr-pAr-pUr-pAr-pUr-pGr-pUr-pGr-pCr-pCr-pCr-pGr | Cyclic phosphorylation | 13 | 1.55 | 99.4 | 1.6 | 9.01 | 593.5 | -7 | 4160.5474 | 4162.22 | 4160.5409 | 489274.88 |
| 3969-3984 | Cr-pUr-pCr-pCr-pCr-pAr-pCr-pCr-pUr-pCr-pCr-pAr-pCr-pCr-pUr- | Cyclic phosphorylation | 16 | 1.74 | 100 | 1.1 | 9.01 | 620.699 | -8 | 4971.6499 | 4973.75 | 4971.6412 | 1952414.88 |
| 3-12 | Ar-pAr-pUr-pAr-pAr-pAr-pCr-pUr-pAr-pGr | Cyclic phosphorylation | 10 | 0.42 | 100 | 1 | 9.17 | 808.357 | -4 | 3236.4558 | 3237.71 | 3236.4544 | 2663992.25 |
| 3-12 | Ar-pAr-pUr-pAr-pAr-pAr-pCr-pUr-pAr-pGr | Cyclic phosphorylation | 10 | 0.57 | 100 | 1.1 | 9.17 | 1617.723 | -2 | 3236.4563 | 3237.86 | 3236.4544 | 802346.88 |
| 3-12 | Ar-pAr-pUr-pAr-pAr-pAr-pCr-pUr-pAr-pGr | Cyclic phosphorylation | 10 | 0.95 | 100 | 1 | 9.17 | 1078.146 | -3 | 3236.4575 | 3237.86 | 3236.4544 | 1363734.5 |
| 3675-3686 | Cr-pAr-pGr-pUr-pAr-pCr-pAr-pUr-pCr-pAr-pAr-pGr | Cyclic phosphorylation | 12 | 0.83 | 99.5 | 1 | 9.17 | 771.7 | -5 | 3862.5352 | 3864.12 | 3862.5319 | 496071.81 |
| 3675-3686 | Cr-pAr-pGr-pUr-pAr-pCr-pAr-pUr-pCr-pAr-pAr-pGr | Cyclic phosphorylation | 12 | 1.4 | 99.5 | 1 | 9.17 | 642.916 | -6 | 3862.5374 | 3864.15 | 3862.5319 | 944264.56 |
| 3-12 | Ar-pAr-pUr-pAr-pAr-pAr-pCr-pUr-pAr-pGr | Cyclic phosphorylation | 10 | -0.48 | 100 | 1 | 9.19 | 646.484 | -5 | 3236.4529 | 3237.84 | 3236.4544 | 7413729 |
| 3-12 | Ar-pAr-pUr-pAr-pAr-pAr-pCr-pUr-pAr-pGr | Cyclic phosphorylation | 10 | 0.5 | 100 | 1 | 9.19 | 538.569 | -6 | 3236.4561 | 3237.67 | 3236.4544 | 1444306.88 |
| 963-975 | Ar-pAr-pGr-pUr-pCr-pCr-pUr-pUr-pCr-pAr-pCr-pCr-pGr | Cyclic phosphorylation | 13 | 1.28 | 98.3 | 1 | 9.25 | 685.916 | -6 | 4120.54 | 4121.93 | 4120.5348 | 273002.09 |

| | | | | | | | | | | | | | |
|---|---|---|---|---|---|---|---|---|---|---|---|---|---|
| 3898-3910 | Cr-pAr-pCr-pGr-pCr-pAr-pAr-pUr-pGr-pCr-pUr-pAr-pGr | Cyclic phosphorylation | 13 | 1.34 | 100 | 1.2 | 9.27 | 835.908 | -5 | 4183.5737 | 4185.21 | 4183.5681 | 464773.12 |
| 3898-3910 | Cr-pAr-pCr-pGr-pCr-pAr-pAr-pUr-pGr-pCr-pUr-pAr-pGr | Cyclic phosphorylation | 13 | 1.34 | 100 | 1 | 9.27 | 696.422 | -6 | 4183.5737 | 4185.3 | 4183.5681 | 1041489.88 |
| 3490-3501 | Ar-pCr-pAr-pGr-pCr-pUr-pUr-pCr-pAr-pAr-pAr-pGr | Cyclic phosphorylation | 12 | 0.96 | 100 | 1.1 | 9.48 | 1286.839 | -3 | 3862.5356 | 3864.12 | 3862.5319 | 306139.25 |
| 3490-3501 | Ar-pCr-pAr-pGr-pCr-pUr-pUr-pCr-pAr-pAr-pAr-pGr | Cyclic phosphorylation | 12 | 0.9 | 100 | 1.2 | 9.5 | 771.7 | -5 | 3862.5354 | 3864.17 | 3862.5319 | 797249.69 |
| 3490-3501 | Ar-pCr-pAr-pGr-pCr-pUr-pUr-pCr-pAr-pAr-pAr-pGr | Cyclic phosphorylation | 12 | 1.09 | 100 | 1 | 9.5 | 642.915 | -6 | 3862.5361 | 3864.13 | 3862.5319 | 1704255.5 |
| 4085-4096 | Ar-pUr-pUr-pAr-pAr-pCr-pCr-pUr-pUr-pUr-pAr-pGr | Cyclic phosphorylation | 12 | 1.14 | 100 | 1 | 9.52 | 949.611 | -4 | 3801.4709 | 3803 | 3801.4666 | 365095.91 |
| 4085-4096 | Ar-pUr-pUr-pAr-pAr-pCr-pCr-pUr-pUr-pUr-pAr-pGr | Cyclic phosphorylation | 12 | 1.08 | 100 | 1.1 | 9.53 | 759.487 | -5 | 3801.4707 | 3803.06 | 3801.4666 | 1306292.5 |
| 4085-4096 | Ar-pUr-pUr-pAr-pAr-pCr-pCr-pUr-pUr-pUr-pAr-pGr | Cyclic phosphorylation | 12 | 0.82 | 100 | 1 | 9.53 | 632.738 | -6 | 3801.4697 | 3803.05 | 3801.4666 | 2706220.5 |
| 4085-4096 | Ar-pUr-pUr-pAr-pAr-pCr-pCr-pUr-pUr-pUr-pAr-pGr | Cyclic phosphorylation | 12 | 0.95 | 100 | 1 | 9.53 | 1266.484 | -3 | 3801.4702 | 3803.05 | 3801.4666 | 489408.44 |
| 292-305 | Ar-pCr-pAr-pAr-pCr-pCr-pCr-pCr-pGr-pUr-pGr-pCr-pUr-pGr | Cyclic phosphorylation | 14 | 2.29 | 99.9 | 1.1 | 9.8 | 637.08 | -7 | 4464.6084 | 4466.72 | 4464.5982 | 619231.44 |
| 292-305 | Ar-pCr-pAr-pAr-pCr-pCr-pCr-pCr-pGr-pUr-pGr-pCr-pUr-pGr | Cyclic phosphorylation | 14 | 1.63 | 99.9 | 1 | 9.8 | 743.427 | -6 | 4464.6055 | 4466.78 | 4464.5982 | 591440.75 |
| 292-305 | Ar-pCr-pAr-pAr-pCr-pCr-pCr-pCr-pGr-pUr-pGr-pCr-pUr-pGr | Cyclic phosphorylation | 14 | 1.85 | 99.9 | 2 | 9.8 | 892.313 | -5 | 4464.6064 | 4466.91 | 4464.5982 | 208423.81 |
| 1728-1742 | Ur-pUr-pCr-pCr-pUr-pGr-pCr-pCr-pAr-pUr-pUr-pCr-pCr-pAr-pG | Cyclic phosphorylation | 15 | 1.26 | 100 | 1.9 | 9.81 | 1568.854 | -3 | 4708.5801 | 4710.55 | 4708.5741 | 170585.28 |
| 1728-1742 | Ur-pUr-pCr-pCr-pUr-pGr-pCr-pCr-pAr-pUr-pUr-pCr-pCr-pAr-pG | Cyclic phosphorylation | 15 | 1.99 | 100 | 1 | 9.83 | 783.923 | -6 | 4708.5835 | 4710.6 | 4708.5741 | 535256.56 |
| 1728-1742 | Ur-pUr-pCr-pCr-pUr-pGr-pCr-pCr-pAr-pUr-pUr-pCr-pCr-pAr-pG | Cyclic phosphorylation | 15 | 1.78 | 100 | 1 | 9.83 | 671.79 | -7 | 4708.5825 | 4710.59 | 4708.5741 | 1406684.12 |
| 1728-1742 | Ur-pUr-pCr-pCr-pUr-pGr-pCr-pCr-pAr-pUr-pUr-pCr-pCr-pAr-pG | Cyclic phosphorylation | 15 | 2.3 | 100 | 1.9 | 9.83 | 587.817 | -8 | 4708.585 | 4710.57 | 4708.5741 | 456776.44 |
| 3-12 | Ar-pAr-pUr-pAr-pAr-pAr-pCr-pUr-pAr-pGr | Phosphorylation | 10 | 1.3 | 100 | 1 | 9.92 | 1084.15 | -3 | 3254.4692 | 3255.89 | 3254.465 | 327466.5 |
| 3-12 | Ar-pAr-pUr-pAr-pAr-pAr-pCr-pUr-pAr-pGr | Phosphorylation | 10 | 0.78 | 100 | 1 | 9.92 | 812.86 | -4 | 3254.4675 | 3255.84 | 3254.465 | 1099209.62 |
| 3-12 | Ar-pAr-pUr-pAr-pAr-pAr-pCr-pUr-pAr-pGr | Phosphorylation | 10 | 1.45 | 100 | 1 | 9.92 | 1626.728 | -2 | 3254.4697 | 3255.87 | 3254.465 | 248955.67 |
| 3-12 | Ar-pAr-pUr-pAr-pAr-pAr-pCr-pUr-pAr-pGr | Phosphorylation | 10 | 1.45 | 100 | 1 | 9.92 | 650.087 | -5 | 3254.4697 | 3255.87 | 3254.465 | 739553.69 |
| 3393-3405 | Ar-pUr-pCr-pAr-pUr-pCr-pAr-pCr-pCr-pAr-pCr-pCr-pGr | Cyclic phosphorylation | 13 | 1.5 | 100 | 1.1 | 10.13 | 585.359 | -7 | 4103.562 | 4105.23 | 4103.5558 | 1358194.25 |
| 3393-3405 | Ar-pUr-pCr-pAr-pUr-pCr-pAr-pCr-pCr-pAr-pCr-pCr-pGr | Cyclic phosphorylation | 13 | 0.91 | 100 | 1.1 | 10.13 | 1367.18 | -3 | 4103.5596 | 4105.23 | 4103.5558 | 795660.12 |
| 3393-3405 | Ar-pUr-pCr-pAr-pUr-pCr-pAr-pCr-pCr-pAr-pCr-pCr-pGr | Cyclic phosphorylation | 13 | 0.43 | 100 | 1.1 | 10.13 | 683.086 | -6 | 4103.5576 | 4105.25 | 4103.5558 | 3939381.75 |
| 3393-3405 | Ar-pUr-pCr-pAr-pUr-pCr-pAr-pCr-pCr-pAr-pCr-pCr-pGr | Cyclic phosphorylation | 13 | 0.91 | 100 | 1.2 | 10.13 | 1025.133 | -4 | 4103.5596 | 4105.23 | 4103.5558 | 657837.5 |
| 3393-3405 | Ar-pUr-pCr-pAr-pUr-pCr-pAr-pCr-pCr-pAr-pCr-pCr-pGr | Cyclic phosphorylation | 13 | 0.67 | 100 | 1.3 | 10.13 | 819.904 | -5 | 4103.5586 | 4105.27 | 4103.5558 | 2037273 |
| 4085-4096 | Ar-pUr-pUr-pAr-pAr-pCr-pCr-pUr-pUr-pUr-pAr-pGr | Phosphorylation | 12 | 1.12 | 100 | 1.1 | 10.14 | 763.089 | -5 | 3819.4814 | 3821.07 | 3819.4772 | 602808.94 |
| 3525-3535 | Ar-pAr-pCr-pCr-pAr-pCr-pAr-pCr-pAr-pAr-pGr | Cyclic phosphorylation | 11 | 0.86 | 100 | 1 | 10.25 | 884.126 | -4 | 3539.5308 | 3541.03 | 3539.5277 | 1552657.62 |
| 3525-3535 | Ar-pAr-pCr-pCr-pAr-pCr-pAr-pCr-pAr-pAr-pGr | Cyclic phosphorylation | 11 | 0.8 | 100 | 1 | 10.26 | 589.081 | -6 | 3539.5305 | 3541.05 | 3539.5277 | 2481891.25 |
| 3525-3535 | Ar-pAr-pCr-pCr-pAr-pCr-pAr-pCr-pAr-pAr-pGr | Cyclic phosphorylation | 11 | 1.49 | 100 | 1.6 | 10.27 | 1769.26 | -2 | 3539.533 | 3541.02 | 3539.5277 | 454330.22 |
| 3525-3535 | Ar-pAr-pCr-pCr-pAr-pCr-pAr-pCr-pAr-pAr-pGr | Cyclic phosphorylation | 11 | 0.8 | 100 | 1.1 | 10.27 | 1179.17 | -3 | 3539.5305 | 3541.04 | 3539.5277 | 1054828 |
| 3525-3535 | Ar-pAr-pCr-pCr-pAr-pCr-pAr-pCr-pAr-pAr-pGr | Cyclic phosphorylation | 11 | 0.04 | 100 | 1 | 10.27 | 707.098 | -5 | 3539.5278 | 3541.02 | 3539.5277 | 3955949.5 |
| 867-881 | Cr-pCr-pUr-pAr-pGr-pAr-pAr-pCr-pCr-pUr-pUr-pCr-pCr-pUr-pGr | Cyclic phosphorylation | 15 | 1.28 | 98.3 | 1.5 | 10.35 | 787.761 | -6 | 4731.6074 | 4733.5 | 4731.6014 | 130441.56 |
| 867-881 | Cr-pCr-pUr-pAr-pGr-pAr-pAr-pCr-pCr-pUr-pUr-pCr-pCr-pUr-pGr | Cyclic phosphorylation | 15 | 2 | 98.3 | 1.4 | 10.37 | 675.223 | -7 | 4731.6108 | 4733.58 | 4731.6014 | 324584.91 |
| 660-671 | Ar-pUr-pCr-pUr-pAr-pCr-pAr-pGr-pCr-pAr-pAr-pGr | Cyclic phosphorylation | 12 | 2.23 | 99.9 | 1.1 | 10.55 | 771.701 | -5 | 3862.5405 | 3864.21 | 3862.5319 | 212184.62 |
| 660-671 | Ar-pUr-pCr-pUr-pAr-pCr-pAr-pGr-pCr-pAr-pAr-pGr | Cyclic phosphorylation | 12 | 2.73 | 99.9 | 1.1 | 10.55 | 642.917 | -6 | 3862.5425 | 3864.12 | 3862.5319 | 433198.09 |
| 633-645 | Ur-pUr-pUr-pAr-pAr-pGr-pAr-pAr-pCr-pAr-pUr-pCr-pGr | Cyclic phosphorylation | 13 | 1.35 | 98.3 | 1.2 | 10.63 | 694.085 | -6 | 4169.5469 | 4171.41 | 4169.5413 | 364473.03 |
| 3273-3285 | Ar-pAr-pUr-pUr-pUr-pCr-pAr-pCr-pCr-pAr-pCr-pCr-pGr | Cyclic phosphorylation | 13 | 1 | 100 | 1 | 10.67 | 683.25 | -6 | 4104.5439 | 4106.21 | 4104.5399 | 3138650.5 |
| 3273-3285 | Ar-pAr-pUr-pUr-pUr-pCr-pAr-pCr-pCr-pAr-pCr-pCr-pGr | Cyclic phosphorylation | 13 | 1.47 | 100 | 1.2 | 10.67 | 1367.508 | -3 | 4104.5459 | 4106.2 | 4104.5399 | 677548.88 |
| 3273-3285 | Ar-pAr-pUr-pUr-pUr-pCr-pAr-pCr-pCr-pAr-pCr-pCr-pGr | Cyclic phosphorylation | 13 | 1.47 | 100 | 1.2 | 10.67 | 1025.379 | -4 | 4104.5459 | 4106.23 | 4104.5399 | 562818.12 |
| 3273-3285 | Ar-pAr-pUr-pUr-pUr-pCr-pAr-pCr-pCr-pAr-pCr-pCr-pGr | Cyclic phosphorylation | 13 | 0.88 | 100 | 1.4 | 10.67 | 820.102 | -5 | 4104.5435 | 4106.21 | 4104.5399 | 1735863.38 |
| 3273-3285 | Ar-pAr-pUr-pUr-pUr-pCr-pAr-pCr-pCr-pAr-pCr-pCr-pGr | Cyclic phosphorylation | 13 | 2.19 | 100 | 1.2 | 10.67 | 585.5 | -7 | 4104.5488 | 4106.23 | 4104.5399 | 1028778.19 |
| 1656-1666 | Ar-pAr-pGr-pAr-pAr-pCr-pAr-pAr-pAr-pUr-pGr | Cyclic phosphorylation | 11 | 1.82 | 99.8 | 1.1 | 10.72 | 599.916 | -6 | 3604.5356 | 3606.05 | 3604.5291 | 322012.03 |
| 1656-1666 | Ar-pAr-pGr-pAr-pAr-pCr-pAr-pAr-pAr-pUr-pGr | Cyclic phosphorylation | 11 | 1.34 | 99.8 | 1 | 10.72 | 720.1 | -5 | 3604.5339 | 3606.01 | 3604.5291 | 265881.5 |
| 2239-2252 | Ur-pGr-pUr-pCr-pCr-pAr-pUr-pGr-pAr-pCr-pCr-pAr-pAr-pGr | Cyclic phosphorylation | 14 | 1.92 | 90.8 | 1.2 | 10.77 | 640.508 | -7 | 4489.6021 | 4491.35 | 4489.5934 | 415158.78 |
| 2239-2252 | Ur-pGr-pUr-pCr-pCr-pAr-pUr-pGr-pAr-pCr-pCr-pAr-pAr-pGr | Cyclic phosphorylation | 14 | 1.59 | 90.8 | 1 | 10.79 | 747.427 | -6 | 4489.6006 | 4491.51 | 4489.5934 | 614080.75 |
| 1346-1361 | Cr-pUr-pGr-pUr-pGr-pUr-pGr-pAr-pUr-pUr-pGr-pCr-pCr-pUr-pG | Cyclic phosphorylation | 16 | 1.99 | 98.9 | 1.1 | 10.81 | 642.945 | -8 | 5150.623 | 5151.28 | 5150.6128 | 198820.05 |
| 2808-2819 | Ar-pAr-pCr-pCr-pAr-pGr-pAr-pAr-pGr-pCr-pUr-pGr | Cyclic phosphorylation | 12 | 3.48 | 99.9 | 1 | 10.84 | 779.506 | -5 | 3901.5676 | 3903.26 | 3901.5541 | 451873.03 |
| 2808-2819 | Ar-pAr-pCr-pCr-pAr-pGr-pAr-pAr-pGr-pCr-pUr-pGr | Cyclic phosphorylation | 12 | 2.47 | 99.9 | 1 | 10.84 | 649.42 | -6 | 3901.5637 | 3903.21 | 3901.5541 | 880895.19 |
| 3761-3775 | Cr-pAr-pUr-pGr-pAr-pCr-pAr-pGr-pCr-pUr-pGr-pCr-pUr-pG | Cyclic phosphorylation | 15 | 2.28 | 99.4 | 1.1 | 10.84 | 801.1 | -6 | 4810.6406 | 4812.82 | 4810.6296 | 363673.28 |
| 3761-3775 | Cr-pAr-pUr-pGr-pAr-pCr-pAr-pGr-pCr-pUr-pGr-pCr-pUr-pG | Cyclic phosphorylation | 15 | 2.59 | 99.4 | 1 | 10.88 | 686.513 | -7 | 4810.6421 | 4812.79 | 4810.6296 | 614576.75 |
| 3201-3215 | Ar-pUr-pGr-pAr-pGr-pCr-pUr-pUr-pCr-pCr-pCr-pUr-pCr-pAr-pG | Cyclic phosphorylation | 15 | 2.85 | 99.1 | 1.2 | 10.97 | 794.596 | -6 | 4771.6211 | 4773.73 | 4771.6075 | 276328.91 |
| 3201-3215 | Ar-pUr-pGr-pAr-pGr-pCr-pUr-pUr-pCr-pCr-pCr-pUr-pCr-pAr-pG | Cyclic phosphorylation | 15 | 2.64 | 99.1 | 1 | 10.97 | 680.796 | -7 | 4771.6201 | 4773.62 | 4771.6075 | 699044.19 |
| 2253-2266 | Ar-pCr-pAr-pGr-pCr-pGr-pUr-pGr-pGr-pAr-pCr-pUr-pGr | Cyclic phosphorylation | 14 | 1.8 | 98.7 | 1.1 | 10.98 | 648.367 | -7 | 4544.6187 | 4546.57 | 4544.6105 | 415464.88 |
| 2253-2266 | Ar-pCr-pAr-pGr-pCr-pGr-pUr-pGr-pGr-pAr-pCr-pUr-pGr | Cyclic phosphorylation | 14 | 2.33 | 98.7 | 1.1 | 10.98 | 756.597 | -6 | 4544.6211 | 4546.61 | 4544.6105 | 307472.12 |
| 2247-2259 | Ar-pCr-pCr-pAr-pAr-pGr-pAr-pCr-pCr-pAr-pGr-pCr-pGr | Cyclic phosphorylation | 13 | 2.78 | 99.9 | 1.3 | 11.12 | 700.096 | -6 | 4205.623 | 4207.34 | 4205.6113 | 390209.59 |
| 2247-2259 | Ar-pCr-pCr-pAr-pAr-pGr-pAr-pCr-pCr-pAr-pGr-pCr-pGr | Cyclic phosphorylation | 13 | 1.86 | 99.9 | 1 | 11.12 | 840.317 | -5 | 4205.6191 | 4207.5 | 4205.6113 | 220092.03 |
| 2085-2098 | Ar-pCr-pAr-pAr-pAr-pCr-pAr-pGr-pCr-pCr-pCr-pUr-pCr-pGr | Cyclic phosphorylation | 14 | 2.48 | 100 | 1.1 | 11.26 | 638.085 | -7 | 4471.6416 | 4473.47 | 4471.6305 | 494772.56 |
| 2085-2098 | Ar-pCr-pAr-pAr-pAr-pCr-pAr-pGr-pCr-pCr-pCr-pUr-pCr-pGr | Cyclic phosphorylation | 14 | 2.7 | 100 | 1.1 | 11.26 | 744.433 | -6 | 4471.6426 | 4473.59 | 4471.6305 | 528789.56 |
| 2085-2098 | Ar-pCr-pAr-pAr-pAr-pCr-pAr-pGr-pCr-pCr-pCr-pUr-pCr-pGr | Cyclic phosphorylation | 14 | 1.94 | 100 | 1.4 | 11.26 | 1489.874 | -3 | 4471.6392 | 4473.56 | 4471.6305 | 175364.14 |
| 2085-2098 | Ar-pCr-pAr-pAr-pAr-pCr-pAr-pGr-pCr-pCr-pCr-pUr-pCr-pGr | Cyclic phosphorylation | 14 | 2.81 | 100 | 1.6 | 11.26 | 893.722 | -5 | 4471.6431 | 4473.58 | 4471.6305 | 218308.92 |
| 3907-3924 | Cr-pUr-pAr-pGr-pCr-pUr-pGr-pCr-pCr-pCr-pCr-pUr-pUr-pCr | Cyclic phosphorylation | 18 | 2.24 | 99.2 | 1.2 | 11.26 | 704.206 | -8 | 5639.7056 | 5642.02 | 5639.6929 | 280633.34 |
| 3907-3924 | Cr-pUr-pAr-pGr-pCr-pUr-pGr-pCr-pCr-pCr-pCr-pUr-pUr-pCr | Cyclic phosphorylation | 18 | 2.24 | 99.2 | 2 | 11.26 | 804.951 | -7 | 5639.7056 | 5641.99 | 5639.6929 | 182654.38 |
| 646-659 | Ar-pCr-pGr-pGr-pCr-pUr-pAr-pUr-pCr-pUr-pCr-pAr-pAr-pGr | Cyclic phosphorylation | 14 | 2.35 | 99.7 | 1 | 11.39 | 640.508 | -7 | 4489.604 | 4491.53 | 4489.5934 | 538564.25 |
| 646-659 | Ar-pCr-pGr-pGr-pCr-pUr-pAr-pUr-pCr-pUr-pCr-pAr-pAr-pGr | Cyclic phosphorylation | 14 | 1.92 | 99.7 | 1 | 11.4 | 747.427 | -6 | 4489.6021 | 4491.58 | 4489.5934 | 415228.84 |
| 1786-1799 | Ar-pUr-pCr-pCr-pCr-pCr-pAr-pGr-pAr-pCr-pAr-pCr-pUr-pGr | Cyclic phosphorylation | 14 | 2.03 | 100 | 1.1 | 11.44 | 740.762 | -6 | 4448.6123 | 4450.47 | 4448.6033 | 598575.5 |
| 1786-1799 | Ar-pUr-pCr-pCr-pCr-pCr-pAr-pGr-pAr-pCr-pAr-pCr-pUr-pGr | Cyclic phosphorylation | 14 | 2.47 | 100 | 1.2 | 11.44 | 634.795 | -7 | 4448.6143 | 4450.6 | 4448.6033 | 672073.06 |
| 2392-2406 | Ar-pGr-pGr-pUr-pGr-pUr-pUr-pCr-pGr-pCr-pCr-pCr-pAr-pAr-pG | Cyclic phosphorylation | 15 | 2.51 | 99.8 | 1.4 | 11.47 | 807.769 | -6 | 4850.6479 | 4852.73 | 4850.6358 | 293265.31 |
| 2392-2406 | Ar-pGr-pGr-pUr-pGr-pUr-pUr-pCr-pGr-pCr-pCr-pCr-pAr-pAr-pG | Cyclic phosphorylation | 15 | 2.81 | 99.8 | 1.2 | 11.47 | 692.229 | -7 | 4850.6494 | 4852.82 | 4850.6358 | 432664.22 |
| 2578-2593 | Gr-pCr-pGr-pCr-pAr-pUr-pUr-pGr-pCr-pCr-pGr-pCr-pCr-pAr | Cyclic phosphorylation | 16 | 2.24 | 98.1 | 1.3 | 11.49 | 735.665 | -7 | 5154.7046 | 5156.92 | 5154.6931 | 271440.31 |
| 3864-3877 | Cr-pAr-pCr-pUr-pAr-pCr-pAr-pCr-pAr-pUr-pGr-pAr-pUr-pGr | Cyclic phosphorylation | 14 | 2.1 | 100 | 1.3 | 11.56 | 893.915 | -5 | 4473.6079 | 4475.55 | 4473.5985 | 252319.2 |
| 1281-1295 | Ar-pUr-pUr-pGr-pCr-pCr-pCr-pUr-pGr-pGr-pAr-pCr-pAr-pG | Cyclic phosphorylation | 15 | 2.69 | 99.6 | 1.1 | 11.58 | 800.934 | -6 | 4810.6426 | 4812.81 | 4810.6296 | 385592.78 |
| 1281-1295 | Ar-pUr-pUr-pGr-pCr-pCr-pCr-pUr-pGr-pGr-pAr-pCr-pAr-pG | Cyclic phosphorylation | 15 | 2.28 | 99.6 | 1 | 11.58 | 686.37 | -7 | 4810.6406 | 4812.66 | 4810.6296 | 648831.5 |
| 3864-3877 | Cr-pAr-pCr-pUr-pAr-pCr-pAr-pCr-pAr-pUr-pGr-pAr-pUr-pGr | Cyclic phosphorylation | 14 | 2.32 | 100 | 1.1 | 11.58 | 744.761 | -6 | 4473.6089 | 4475.54 | 4473.5985 | 562998.12 |
| 3864-3877 | Cr-pAr-pCr-pUr-pAr-pCr-pAr-pCr-pAr-pUr-pGr-pAr-pUr-pGr | Cyclic phosphorylation | 14 | 2.97 | 100 | 1.6 | 11.58 | 638.223 | -7 | 4473.6118 | 4475.51 | 4473.5985 | 179580.66 |
| 1017-1032 | Cr-pCr-pCr-pAr-pCr-pCr-pGr-pAr-pAr-pUr-pCr-pCr-pAr-pUr-pCr | Cyclic phosphorylation | 16 | 1.97 | 96.1 | 1.3 | 11.89 | 721.95 | -7 | 5058.6958 | 5060.81 | 5058.6858 | 228648.83 |
| 3639-3653 | Ar-pGr-pCr-pCr-pUr-pGr-pAr-pUr-pCr-pGr-pAr-pCr-pCr-pUr-pG | Cyclic phosphorylation | 15 | 2.49 | 100 | 1.4 | 12.02 | 686.514 | -7 | 4810.6416 | 4812.72 | 4810.6296 | 316100.22 |
| 3639-3653 | Ar-pGr-pCr-pCr-pUr-pGr-pAr-pUr-pCr-pGr-pAr-pCr-pCr-pUr-pG | Cyclic phosphorylation | 15 | 2.49 | 100 | 1.2 | 12.02 | 800.933 | -6 | 4810.6416 | 4812.67 | 4810.6296 | 303801.31 |
| 3563-3577 | Cr-pGr-pGr-pAr-pAr-pUr-pCr-pAr-pAr-pUr-pGr-pCr-pAr-pG | Cyclic phosphorylation | 15 | 3.8 | 100 | 1.1 | 12.21 | 808.774 | -6 | 4857.6865 | 4859.62 | 4857.6681 | 866418.19 |
| 3563-3577 | Cr-pGr-pGr-pAr-pAr-pUr-pCr-pAr-pAr-pUr-pGr-pCr-pAr-pG | Cyclic phosphorylation | 15 | 2.59 | 100 | 1.2 | 12.23 | 693.09 | -7 | 4857.6807 | 4859.66 | 4857.6681 | 919907.38 |
| 3985-4002 | Cr-pCr-pCr-pCr-pAr-pCr-pUr-pCr-pAr-pCr-pCr-pAr-pCr-pCr-pUr- | Cyclic phosphorylation | 18 | 1.01 | 100 | 1.8 | 12.26 | 933.617 | -6 | 5605.7407 | 5608.17 | 5605.7351 | 1671653.38 |
| 3985-4002 | Cr-pCr-pCr-pCr-pAr-pCr-pUr-pCr-pAr-pCr-pCr-pAr-pCr-pCr-pUr- | Cyclic phosphorylation | 18 | 0.92 | 100 | 1.3 | 12.26 | 1120.542 | -5 | 5605.7402 | 5608.22 | 5605.7351 | 575869.31 |
| 3985-4002 | Cr-pCr-pCr-pCr-pAr-pCr-pUr-pCr-pAr-pCr-pCr-pAr-pCr-pCr-pUr- | Cyclic phosphorylation | 18 | 1.19 | 100 | 1.1 | 12.26 | 800.098 | -7 | 5605.7417 | 5608.2 | 5605.7351 | 4131458 |
| 3985-4002 | Cr-pCr-pCr-pCr-pAr-pCr-pUr-pCr-pAr-pCr-pCr-pAr-pCr-pCr-pUr- | Cyclic phosphorylation | 18 | 0.75 | 100 | 1.2 | 12.26 | 1400.929 | -4 | 5605.7393 | 5608.23 | 5605.7351 | 582035.88 |
| 3985-4002 | Cr-pCr-pCr-pCr-pAr-pCr-pUr-pCr-pAr-pCr-pCr-pAr-pCr-pCr-pUr- | Cyclic phosphorylation | 18 | 1.19 | 100 | 1 | 12.26 | 699.96 | -8 | 5605.7417 | 5608.05 | 5605.7351 | 5853854 |
| 3985-4002 | Cr-pCr-pCr-pCr-pAr-pCr-pUr-pCr-pAr-pCr-pCr-pAr-pCr-pCr-pUr- | Cyclic phosphorylation | 18 | 1.45 | 100 | 1.3 | 12.26 | 622.076 | -9 | 5605.7432 | 5608.07 | 5605.7351 | 1064776.38 |
| 2916-2930 | Ar-pUr-pGr-pCr-pCr-pCr-pAr-pGr-pGr-pCr-pAr-pCr-pUr-pG | Cyclic phosphorylation | 15 | 1.39 | 100 | 1.1 | 12.38 | 689.802 | -7 | 4833.6636 | 4835.85 | 4833.6569 | 1277698.75 |
| 2916-2930 | Ar-pUr-pGr-pCr-pCr-pCr-pAr-pGr-pGr-pCr-pAr-pCr-pUr-pG | Cyclic phosphorylation | 15 | 1.79 | 100 | 1.4 | 12.38 | 804.938 | -6 | 4833.6655 | 4835.77 | 4833.6569 | 655151.38 |
| 3883-3897 | Ar-pGr-pCr-pUr-pGr-pGr-pUr-pAr-pCr-pUr-pGr-pCr-pAr-pUr-pG | Cyclic phosphorylation | 15 | 1.47 | 99.7 | 1.4 | 12.38 | 692.369 | -7 | 4851.627 | 4853.86 | 4851.6198 | 695635 |
| 3883-3897 | Ar-pGr-pCr-pUr-pGr-pGr-pUr-pAr-pCr-pUr-pGr-pCr-pAr-pUr-pG | Cyclic phosphorylation | 15 | 0.57 | 99.7 | 1 | 12.4 | 807.931 | -6 | 4851.6226 | 4853.77 | 4851.6198 | 808985.12 |
| 567-584 | Ur-pCr-pCr-pAr-pGr-pCr-pCr-pUr-pUr-pCr-pUr-pGr-pCr-pUr-pG | Cyclic phosphorylation | 18 | 1.35 | 100 | 1.1 | 12.4 | 628.626 | -9 | 5664.6958 | 5667.17 | 5664.6882 | 1119125.62 |
| 567-584 | Ur-pCr-pCr-pAr-pGr-pCr-pCr-pUr-pUr-pCr-pUr-pGr-pCr-pUr-pG | Cyclic phosphorylation | 18 | 0.92 | 100 | 1 | 12.4 | 707.33 | -8 | 5664.6934 | 5667.17 | 5664.6882 | 2037599.75 |
| 986-1000 | Cr-pAr-pUr-pCr-pUr-pAr-pCr-pCr-pAr-pGr-pAr-pCr-pCr-pAr-pG | Cyclic phosphorylation | 15 | 1.53 | 100 | 1.2 | 12.43 | 681.659 | -7 | 4777.6631 | 4779.72 | 4777.6558 | 674534.19 |
| 986-1000 | Cr-pAr-pUr-pCr-pUr-pAr-pCr-pCr-pAr-pGr-pAr-pCr-pCr-pAr-pG | Cyclic phosphorylation | 15 | 1.32 | 100 | 1.2 | 12.43 | 795.604 | -6 | 4777.6621 | 4779.75 | 4777.6558 | 649891 |
| 2085-2099 | Ar-pCr-pAr-pAr-pAr-pCr-pAr-pGr-pCr-pCr-pCr-pUr-pCr-pGr-pG | Cyclic phosphorylation | 15 | 2.29 | 91.8 | 1.2 | 12.49 | 687.377 | -7 | 4816.689 | 4818.84 | 4816.6779 | 709052.31 |
| 3898-3913 | Cr-pAr-pCr-pGr-pCr-pAr-pAr-pUr-pGr-pCr-pUr-pAr-pGr-pCr-pU | Cyclic phosphorylation | 16 | 1.8 | 96.2 | 1 | 12.54 | 733.521 | -7 | 5139.6914 | 5141.85 | 5139.6822 | 780933 |
| 3898-3913 | Cr-pAr-pCr-pGr-pCr-pAr-pAr-pUr-pGr-pCr-pUr-pAr-pGr-pCr-pU | Cyclic phosphorylation | 16 | 1.42 | 96.2 | 1.5 | 12.54 | 855.775 | -6 | 5139.6895 | 5141.86 | 5139.6822 | 375220.97 |
| 1786-1800 | Ar-pUr-pCr-pCr-pCr-pCr-pAr-pGr-pAr-pCr-pAr-pCr-pUr-pGr-pG | Cyclic phosphorylation | 15 | 1.67 | 99.8 | 1.1 | 12.57 | 684.088 | -7 | 4793.6587 | 4795.74 | 4793.6507 | 403710.88 |
| 2022-2034 | Ar-pAr-pCr-pAr-pAr-pUr-pAr-pGr-pCr-pUr-pAr-pCr-pGr | Cyclic phosphorylation | 13 | 1.52 | 100 | 1.1 | 12.57 | 1396.523 | -3 | 4191.5908 | 4193.33 | 4191.5845 | 282504.66 |
| 2022-2034 | Ar-pAr-pCr-pAr-pAr-pUr-pAr-pGr-pCr-pUr-pAr-pCr-pGr | Cyclic phosphorylation | 13 | 1.64 | 100 | 1 | 12.57 | 697.758 | -6 | 4191.5913 | 4193.29 | 4191.5845 | 1183135 |
| 2022-2034 | Ar-pAr-pCr-pAr-pAr-pUr-pAr-pGr-pCr-pUr-pAr-pCr-pGr | Cyclic phosphorylation | 13 | 1.17 | 100 | 1.1 | 12.57 | 837.511 | -5 | 4191.5894 | 4193.31 | 4191.5845 | 685701.56 |
| 3742-3757 | Ur-pCr-pAr-pCr-pAr-pAr-pUr-pCr-pAr-pUr-pGr-pCr-pUr-pGr-pU | Cyclic phosphorylation | 16 | 2.01 | 100 | 1.6 | 12.73 | 849.435 | -6 | 5101.6543 | 5103.75 | 5101.644 | 222086.7 |
| 3742-3757 | Ur-pCr-pAr-pCr-pAr-pAr-pUr-pCr-pAr-pUr-pGr-pCr-pUr-pGr-pU | Cyclic phosphorylation | 16 | 2.01 | 100 | 1 | 12.75 | 728.087 | -7 | 5101.6543 | 5103.75 | 5101.644 | 480407.56 |
| 2628-2645 | Ar-pCr-pAr-pGr-pUr-pGr-pCr-pUr-pGr-pCr-pCr-pUr-pCr-pGr-pU | Cyclic phosphorylation | 18 | 2.34 | 95.1 | 1.3 | 12.78 | 712.209 | -8 | 5703.7236 | 5706.26 | 5703.7103 | 284510.59 |
| 3985-4002 | Cr-pCr-pCr-pCr-pAr-pCr-pUr-pCr-pAr-pCr-pCr-pAr-pCr-pCr-pUr- | Phosphorylation | 18 | 2.43 | 100 | 1.6 | 12.8 | 936.62 | -6 | 5623.7593 | 5626.01 | 5623.7456 | 262162.69 |
| 3985-4002 | Cr-pCr-pCr-pCr-pAr-pCr-pUr-pCr-pAr-pCr-pCr-pAr-pCr-pCr-pUr- | Phosphorylation | 18 | 1.47 | 100 | 1 | 12.8 | 802.53 | -7 | 5623.7539 | 5626.24 | 5623.7456 | 307554 |
| 3985-4002 | Cr-pCr-pCr-pCr-pAr-pCr-pUr-pCr-pAr-pCr-pCr-pAr-pCr-pCr-pUr- | Phosphorylation | 18 | 2 | 100 | 1.3 | 12.82 | 702.088 | -8 | 5623.7568 | 5625.96 | 5623.7456 | 136761.98 |
| 3578-3593 | Cr-pGr-pUr-pCr-pGr-pUr-pGr-pAr-pAr-pCr-pAr-pUr-pCr-pCr-pA | Cyclic phosphorylation | 16 | 2.46 | 100 | 1 | 12.87 | 733.521 | -7 | 5139.6948 | 5141.83 | 5139.6822 | 1009579.94 |
| 3578-3593 | Cr-pGr-pUr-pCr-pGr-pUr-pGr-pAr-pAr-pCr-pAr-pUr-pCr-pCr-pA | Cyclic phosphorylation | 16 | 2.65 | 100 | 1.1 | 12.87 | 855.943 | -6 | 5139.6958 | 5141.83 | 5139.6822 | 566959.44 |
| 3286-3303 | Cr-pUr-pCr-pAr-pGr-pCr-pAr-pUr-pCr-pUr-pGr-pCr-pGr-pCr | Cyclic phosphorylation | 18 | 2.27 | 99.6 | 2 | 12.92 | 630.967 | -9 | 5685.7603 | 5688.38 | 5685.7473 | 213849.7 |
| 3286-3303 | Cr-pUr-pCr-pAr-pGr-pCr-pAr-pUr-pCr-pUr-pGr-pCr-pGr-pCr | Cyclic phosphorylation | 18 | 2.36 | 99.6 | 1 | 12.96 | 710.088 | -8 | 5685.7607 | 5688.54 | 5685.7473 | 694716.56 |
| 3286-3303 | Cr-pUr-pCr-pAr-pGr-pCr-pAr-pUr-pCr-pUr-pGr-pCr-pGr-pCr | Cyclic phosphorylation | 18 | 1.67 | 99.6 | 1.4 | 12.96 | 811.53 | -7 | 5685.7568 | 5688.49 | 5685.7473 | 432071.47 |
| 3486-3501 | Cr-pUr-pGr-pAr-pCr-pAr-pGr-pCr-pUr-pUr-pCr-pAr-pAr-pA | Cyclic phosphorylation | 16 | 1.41 | 100 | 1 | 13.06 | 736.951 | -7 | 5163.7007 | 5165.91 | 5163.6934 | 1152903 |
| 3486-3501 | Cr-pUr-pGr-pAr-pCr-pAr-pGr-pCr-pUr-pUr-pCr-pAr-pAr-pA | Cyclic phosphorylation | 16 | 0.94 | 100 | 1.1 | 13.06 | 859.943 | -6 | 5163.6982 | 5165.9 | 5163.6934 | 491388.81 |
| 4013-4028 | Ar-pCr-pAr-pCr-pCr-pUr-pCr-pCr-pAr-pAr-pGr-pCr-pAr-pCr- | Cyclic phosphorylation | 16 | 0.83 | 100 | 1.1 | 13.1 | 846.28 | -6 | 5081.7173 | 5083.85 | 5081.7131 | 740022.38 |
| 4013-4028 | Ar-pCr-pAr-pCr-pCr-pUr-pCr-pCr-pAr-pAr-pGr-pCr-pAr-pCr- | Cyclic phosphorylation | 16 | 1.7 | 100 | 1 | 13.1 | 725.239 | -7 | 5081.7217 | 5083.86 | 5081.7131 | 1022262.81 |
| 4013-4028 | Ar-pCr-pAr-pCr-pCr-pUr-pCr-pCr-pAr-pAr-pGr-pCr-pAr-pCr- | Cyclic phosphorylation | 16 | 1.6 | 100 | 2 | 13.1 | 634.458 | -8 | 5081.7212 | 5083.87 | 5081.7131 | 437919.22 |
| 3511-3524 | Ar-pCr-pAr-pAr-pGr-pUr-pAr-pCr-pUr-pUr-pUr-pAr-pAr-pGr | Cyclic phosphorylation | 14 | 0.65 | 100 | 1 | 13.21 | 1499.193 | -3 | 4498.5967 | 4500.55 | 4498.5938 | 573690.25 |

| | | | | | | | | | | | | |
|---|---|---|---|---|---|---|---|---|---|---|---|---|
| 3511-3524 | Ar-pCr-pAr-pAr-pGr-pUr-pAr-pCr-pUr-pUr-pUr-pAr-pAr-pGr | Cyclic phosphorylation | 14 | 1.52 | 100 | 1.1 | 13.21 | 641.793 | -7 | 4498.6006 | 4500.57 | 4498.5938 | 1961985.88 |
| 3511-3524 | Ar-pCr-pAr-pAr-pGr-pUr-pAr-pCr-pUr-pUr-pUr-pAr-pAr-pGr | Cyclic phosphorylation | 14 | 0.86 | 100 | 1 | 13.21 | 748.926 | -6 | 4498.5977 | 4500.55 | 4498.5938 | 2215776 |
| 567-585 | Ur-pCr-pCr-pCr-pAr-pGr-pCr-pCr-pUr-pUr-pUr-pCr-pCr-pUr-pG | Cyclic phosphorylation | 19 | 2.15 | 99.9 | 1.1 | 13.21 | 666.965 | -9 | 6009.7485 | 6012.05 | 6009.7356 | 910440.62 |
| 3511-3524 | Ar-pCr-pAr-pAr-pGr-pUr-pAr-pCr-pUr-pUr-pUr-pAr-pAr-pGr | Cyclic phosphorylation | 14 | 1.08 | 100 | 1 | 13.22 | 898.913 | -5 | 4498.5986 | 4500.57 | 4498.5938 | 791676.88 |
| 567-585 | Ur-pCr-pCr-pCr-pAr-pGr-pCr-pCr-pUr-pUr-pUr-pCr-pCr-pUr-pG | Cyclic phosphorylation | 19 | 1.42 | 99.9 | 1.1 | 13.23 | 750.461 | -8 | 6009.7441 | 6012.32 | 6009.7356 | 1260887.12 |
| 567-585 | Ur-pCr-pCr-pCr-pAr-pGr-pCr-pCr-pUr-pUr-pUr-pCr-pCr-pUr-pG | Cyclic phosphorylation | 19 | 2.48 | 99.9 | 1.1 | 13.24 | 857.814 | -7 | 6009.7505 | 6012.2 | 6009.7356 | 720460.81 |
| 3878-3893 | Ar-pCr-pUr-pCr-pGr-pAr-pGr-pCr-pUr-pGr-pGr-pUr-pAr-pCr-pU | Cyclic phosphorylation | 16 | 2.85 | 100 | 1 | 13.35 | 858.772 | -6 | 5156.6758 | 5158.67 | 5156.6611 | 411739.47 |
| 1643-1658 | Cr-pAr-pCr-pCr-pAr-pAr-pUr-pCr-pUr-pCr-pGr-pUr-pGr-pAr-pA | Cyclic phosphorylation | 16 | 1.38 | 99.1 | 1 | 13.36 | 731.236 | -7 | 5123.6943 | 5125.82 | 5123.6872 | 854225.56 |
| 1643-1658 | Cr-pAr-pCr-pCr-pAr-pAr-pUr-pCr-pUr-pCr-pGr-pUr-pGr-pAr-pA | Cyclic phosphorylation | 16 | 0.91 | 99.1 | 1.1 | 13.36 | 853.276 | -6 | 5123.6919 | 5125.83 | 5123.6872 | 613140.69 |
| 3878-3893 | Ar-pCr-pUr-pCr-pGr-pAr-pGr-pCr-pUr-pGr-pGr-pUr-pAr-pCr-pU | Cyclic phosphorylation | 16 | 2 | 100 | 1 | 13.36 | 735.946 | -7 | 5156.6714 | 5158.87 | 5156.6611 | 905987.06 |
| 3738-3752 | Ar-pUr-pGr-pGr-pUr-pCr-pAr-pCr-pAr-pAr-pUr-pCr-pAr-pUr-pG | Cyclic phosphorylation | 15 | 2.01 | 100 | 1 | 13.38 | 687.799 | -7 | 4819.6396 | 4821.7 | 4819.63 | 1734666.75 |
| 3738-3752 | Ar-pUr-pGr-pGr-pUr-pCr-pAr-pCr-pAr-pAr-pUr-pCr-pAr-pUr-pG | Cyclic phosphorylation | 15 | 2.01 | 100 | 1 | 13.4 | 802.6 | -6 | 4819.6396 | 4821.7 | 4819.63 | 1015137.75 |
| 3849-3863 | Ar-pAr-pGr-pGr-pGr-pCr-pGr-pUr-pGr-pAr-pAr-pAr-pCr-pUr-pG | Cyclic phosphorylation | 15 | 1.74 | 100 | 1 | 13.55 | 704.663 | -7 | 4937.689 | 4939.76 | 4937.6804 | 1226278.5 |
| 3849-3863 | Ar-pAr-pGr-pGr-pGr-pCr-pGr-pUr-pGr-pAr-pAr-pAr-pCr-pUr-pG | Cyclic phosphorylation | 15 | 1.84 | 100 | 1 | 13.55 | 822.108 | -6 | 4937.6895 | 4939.75 | 4937.6804 | 421491.38 |
| 3849-3863 | Ar-pAr-pGr-pGr-pGr-pCr-pGr-pUr-pGr-pAr-pAr-pAr-pCr-pUr-pG | Cyclic phosphorylation | 15 | 1.34 | 100 | 1.5 | 13.56 | 616.329 | -8 | 4937.687 | 4939.78 | 4937.6804 | 355466.31 |
| 3758-3775 | Ur-pUr-pGr-pCr-pAr-pUr-pGr-pAr-pCr-pCr-pAr-pGr-pCr-pUr-pG | Cyclic phosphorylation | 18 | 1.33 | 99.9 | 1.1 | 13.59 | 823.385 | -7 | 5767.7354 | 5770.08 | 5767.7277 | 373509.44 |
| 3758-3775 | Ur-pUr-pGr-pCr-pAr-pUr-pGr-pAr-pCr-pCr-pAr-pGr-pCr-pUr-pG | Cyclic phosphorylation | 18 | 2.18 | 99.9 | 1.1 | 13.61 | 720.211 | -8 | 5767.7402 | 5770.12 | 5767.7277 | 617132.75 |
| 2004-2019 | Ar-pUr-pCr-pGr-pGr-pAr-pGr-pCr-pCr-pGr-pAr-pGr-pCr-pAr-pC | Cyclic phosphorylation | 16 | 3.68 | 98 | 1.1 | 13.62 | 744.67 | -7 | 5217.7456 | 5220.03 | 5217.7264 | 525778.5 |
| 1312-1325 | Ar-pCr-pUr-pAr-pCr-pAr-pAr-pCr-pUr-pAr-pCr-pAr-pAr-pGr | Cyclic phosphorylation | 14 | 1.75 | 99.1 | 1.2 | 13.69 | 746.1 | -6 | 4480.6387 | 4482.61 | 4480.6308 | 442087.03 |
| 2041-2058 | Ar-pCr-pAr-pUr-pCr-pCr-pCr-pCr-pAr-pUr-pCr-pGr-pGr-pCr-pGr | Cyclic phosphorylation | 18 | 3.65 | 99.7 | 1.1 | 13.71 | 817.245 | -7 | 5725.7744 | 5728.31 | 5725.7535 | 323575 |
| 2041-2058 | Ar-pCr-pAr-pUr-pCr-pCr-pCr-pCr-pAr-pUr-pCr-pGr-pGr-pCr-pGr | Cyclic phosphorylation | 18 | 1.86 | 99.7 | 1.1 | 13.73 | 715.089 | -8 | 5725.7642 | 5728.28 | 5725.7535 | 590351.19 |
| 864-881 | Cr-pAr-pGr-pCr-pCr-pUr-pAr-pGr-pAr-pAr-pCr-pCr-pUr-pUr-pC | Cyclic phosphorylation | 18 | 2.32 | 100 | 1.3 | 13.76 | 633.744 | -9 | 5710.7559 | 5713.05 | 5710.7426 | 1077068.5 |
| 864-881 | Cr-pAr-pGr-pCr-pCr-pUr-pAr-pGr-pAr-pAr-pCr-pCr-pUr-pUr-pC | Cyclic phosphorylation | 18 | 2.07 | 100 | 1.1 | 13.76 | 713.087 | -8 | 5710.7544 | 5713.24 | 5710.7426 | 1771112.25 |
| 864-881 | Cr-pAr-pGr-pCr-pCr-pUr-pAr-pGr-pAr-pAr-pCr-pCr-pUr-pUr-pC | Cyclic phosphorylation | 18 | 1.98 | 100 | 1.1 | 13.76 | 815.1 | -7 | 5710.7539 | 5713.25 | 5710.7426 | 976046.06 |
| 2229-2246 | Ar-pUr-pCr-pCr-pUr-pGr-pCr-pCr-pUr-pGr-pUr-pGr-pUr-pCr-pC | Cyclic phosphorylation | 18 | 1.8 | 100 | 1.1 | 13.87 | 633.071 | -9 | 5704.7046 | 5707.02 | 5704.6943 | 1770631.12 |
| 2229-2246 | Ar-pUr-pCr-pCr-pUr-pGr-pCr-pCr-pUr-pGr-pUr-pGr-pUr-pCr-pC | Cyclic phosphorylation | 18 | 1.46 | 100 | 1 | 13.88 | 712.331 | -8 | 5704.7026 | 5707.05 | 5704.6943 | 2460808.5 |
| 2229-2246 | Ar-pUr-pCr-pCr-pUr-pGr-pCr-pCr-pUr-pGr-pUr-pGr-pUr-pCr-pC | Cyclic phosphorylation | 18 | 1.2 | 100 | 1 | 13.88 | 814.236 | -7 | 5704.7012 | 5706.84 | 5704.6943 | 1077099 |
| 541-558 | Cr-pCr-pAr-pAr-pCr-pAr-pAr-pCr-pUr-pGr-pCr-pAr-pCr-pCr-pUr | Cyclic phosphorylation | 18 | 2.92 | 97.5 | 1.1 | 13.92 | 631.857 | -9 | 5693.7803 | 5696.22 | 5693.7637 | 197892.72 |
| 541-558 | Cr-pCr-pAr-pAr-pCr-pAr-pAr-pCr-pUr-pGr-pCr-pAr-pCr-pCr-pUr | Cyclic phosphorylation | 18 | 1.63 | 97.5 | 1.1 | 13.92 | 710.964 | -8 | 5693.7729 | 5696.38 | 5693.7637 | 796366.25 |
| 541-558 | Cr-pCr-pAr-pAr-pCr-pAr-pAr-pCr-pUr-pGr-pCr-pAr-pCr-pCr-pUr | Cyclic phosphorylation | 18 | 1.63 | 97.5 | 1.1 | 13.94 | 812.532 | -7 | 5693.7729 | 5696.2 | 5693.7637 | 803710.38 |
| 3547-3562 | Ar-pCr-pCr-pUr-pGr-pGr-pGr-pCr-pGr-pAr-pUr-pAr-pUr-pCr-pA | Cyclic phosphorylation | 16 | 1.16 | 100 | 1 | 14.01 | 739.235 | -7 | 5179.6943 | 5181.83 | 5179.6883 | 1656561.75 |
| 3547-3562 | Ar-pCr-pCr-pUr-pGr-pGr-pGr-pCr-pGr-pAr-pUr-pAr-pUr-pCr-pA | Cyclic phosphorylation | 16 | -2.23 | 100 | 1.1 | 14.01 | 862.608 | -6 | 5179.6768 | 5181.78 | 5179.6883 | 830463.56 |
| 3547-3562 | Ar-pCr-pCr-pUr-pGr-pGr-pGr-pCr-pGr-pAr-pUr-pAr-pUr-pCr-pA | Cyclic phosphorylation | 16 | 1.64 | 100 | 1.2 | 14.01 | 646.705 | -8 | 5179.6968 | 5181.95 | 5179.6883 | 820280.75 |
| 897-912 | Ar-pAr-pCr-pGr-pGr-pCr-pAr-pCr-pCr-pAr-pUr-pCr-pAr-pCr-pCr | Cyclic phosphorylation | 16 | 1.72 | 100 | 1.5 | 14.02 | 639.458 | -8 | 5121.728 | 5124.32 | 5121.7192 | 640817.94 |
| 897-912 | Ar-pAr-pCr-pGr-pGr-pCr-pAr-pCr-pCr-pAr-pUr-pCr-pAr-pCr-pCr | Cyclic phosphorylation | 16 | 1.44 | 100 | 1 | 14.04 | 730.953 | -7 | 5121.7266 | 5124.15 | 5121.7192 | 2093656.75 |
| 897-912 | Ar-pAr-pCr-pGr-pGr-pCr-pAr-pCr-pCr-pAr-pUr-pCr-pAr-pCr-pCr | Cyclic phosphorylation | 16 | 0.96 | 100 | 1.2 | 14.06 | 852.947 | -6 | 5121.7241 | 5124.22 | 5121.7192 | 1119789.38 |
| 897-912 | Ar-pAr-pCr-pGr-pGr-pCr-pAr-pCr-pCr-pAr-pUr-pCr-pAr-pCr-pCr | Cyclic phosphorylation | 16 | 0.48 | 100 | 1.6 | 14.06 | 1023.738 | -5 | 5121.7217 | 5124.04 | 5121.7192 | 424293.66 |
| 1226-1242 | Cr-pUr-pUr-pCr-pAr-pCr-pAr-pAr-pAr-pCr-pGr-pUr-pGr-pUr-pA | Cyclic phosphorylation | 17 | 0.87 | 94.1 | 1 | 14.13 | 774.953 | -7 | 5429.7173 | 5432.01 | 5429.7125 | 440562.66 |
| 2739-2756 | Ar-pUr-pCr-pCr-pCr-pUr-pUr-pUr-pGr-pCr-pUr-pAr-pUr-pG | Cyclic phosphorylation | 18 | 1.43 | 99.4 | 1 | 14.14 | 710.332 | -8 | 5688.7075 | 5690.87 | 5688.6994 | 416954.28 |
| 1600-1617 | Ar-pAr-pCr-pUr-pGr-pCr-pUr-pGr-pCr-pAr-pUr-pGr-pCr-pCr-pC | Cyclic phosphorylation | 18 | 1.58 | 100 | 1.1 | 14.18 | 817.385 | -7 | 5726.7466 | 5729.37 | 5726.7375 | 1001791.88 |
| 1600-1617 | Ar-pAr-pCr-pUr-pGr-pCr-pUr-pGr-pCr-pAr-pUr-pGr-pCr-pCr-pC | Cyclic phosphorylation | 18 | 1.58 | 100 | 1 | 14.18 | 715.087 | -8 | 5726.7466 | 5729.29 | 5726.7375 | 997428.62 |
| 4037-4053 | Cr-pAr-pGr-pCr-pUr-pCr-pAr-pAr-pAr-pAr-pCr-pGr-pCr-pUr-pU | Cyclic phosphorylation | 17 | 1.07 | 100 | 1 | 14.22 | 680.836 | -8 | 5452.7456 | 5455.16 | 5452.7398 | 2731725.5 |
| 4037-4053 | Cr-pAr-pGr-pCr-pUr-pCr-pAr-pAr-pAr-pAr-pCr-pGr-pCr-pUr-pU | Cyclic phosphorylation | 17 | 0.98 | 100 | 1 | 14.22 | 778.242 | -7 | 5452.7451 | 5455.03 | 5452.7398 | 3765355.75 |
| 3736-3752 | Ur-pGr-pAr-pUr-pGr-pGr-pUr-pCr-pAr-pCr-pAr-pAr-pUr-pCr-pA | Cyclic phosphorylation | 17 | 1.68 | 98.1 | 1 | 14.23 | 780.809 | -7 | 5470.7119 | 5473.56 | 5470.7027 | 910409.12 |
| 4037-4053 | Cr-pAr-pGr-pCr-pUr-pCr-pAr-pAr-pAr-pAr-pCr-pGr-pCr-pUr-pU | Cyclic phosphorylation | 17 | 1.16 | 100 | 1.2 | 14.23 | 908.117 | -6 | 5452.7461 | 5455.17 | 5452.7398 | 1676684.75 |
| 4037-4053 | Cr-pAr-pGr-pCr-pUr-pCr-pAr-pAr-pAr-pAr-pCr-pGr-pCr-pUr-pU | Cyclic phosphorylation | 17 | 0.71 | 100 | 1.7 | 14.23 | 1089.942 | -5 | 5452.7437 | 5454.99 | 5452.7398 | 613579.75 |
| 2235-2252 | Cr-pCr-pUr-pGr-pUr-pGr-pUr-pCr-pCr-pAr-pUr-pGr-pAr-pCr-pC | Cyclic phosphorylation | 18 | 1.75 | 100 | 1.1 | 14.25 | 820.816 | -7 | 5750.7588 | 5753.22 | 5750.7487 | 1412520 |
| 2235-2252 | Cr-pCr-pUr-pGr-pUr-pGr-pUr-pCr-pCr-pAr-pUr-pGr-pAr-pCr-pC | Cyclic phosphorylation | 18 | 2.17 | 100 | 1 | 14.28 | 718.088 | -8 | 5750.7612 | 5753.39 | 5750.7487 | 2173548 |
| 2235-2252 | Cr-pCr-pUr-pGr-pUr-pGr-pUr-pCr-pCr-pAr-pUr-pGr-pAr-pCr-pC | Cyclic phosphorylation | 18 | 2.51 | 100 | 1.3 | 14.3 | 638.189 | -9 | 5750.7632 | 5753.27 | 5750.7487 | 755646.31 |
| 3546-3562 | Gr-pAr-pCr-pCr-pUr-pGr-pGr-pGr-pCr-pGr-pAr-pUr-pAr-pUr-pC | Cyclic phosphorylation | 17 | 1.87 | 99 | 1.1 | 14.35 | 689.836 | -8 | 5524.7461 | 5526.92 | 5524.7357 | 300232.75 |
| 3546-3562 | Gr-pAr-pCr-pCr-pUr-pGr-pGr-pGr-pCr-pGr-pAr-pUr-pAr-pUr-pC | Cyclic phosphorylation | 17 | 1.7 | 99 | 1 | 14.35 | 788.528 | -7 | 5524.7451 | 5527.25 | 5524.7357 | 852855.25 |
| 3525-3540 | Ar-pAr-pCr-pCr-pAr-pCr-pAr-pCr-pAr-pAr-pGr-pCr-pCr-pCr-pCr- | Cyclic phosphorylation | 16 | 1.9 | 100 | 1 | 14.39 | 637.337 | -8 | 5104.75 | 5106.95 | 5104.7403 | 1355330.5 |
| 3525-3540 | Ar-pAr-pCr-pCr-pAr-pCr-pAr-pCr-pAr-pAr-pGr-pCr-pCr-pCr-pCr- | Cyclic phosphorylation | 16 | 1.14 | 100 | 1.3 | 14.39 | 1701.244 | -3 | 5104.7461 | 5106.93 | 5104.7403 | 585328.88 |
| 3525-3540 | Ar-pAr-pCr-pCr-pAr-pCr-pAr-pCr-pAr-pAr-pGr-pCr-pCr-pCr-pCr- | Cyclic phosphorylation | 16 | 1.14 | 100 | 1 | 14.39 | 728.528 | -7 | 5104.7461 | 5106.92 | 5104.7403 | 3152277.25 |
| 3525-3540 | Ar-pAr-pCr-pCr-pAr-pCr-pAr-pCr-pAr-pAr-pGr-pCr-pCr-pCr-pCr- | Cyclic phosphorylation | 16 | 0.95 | 100 | 1.2 | 14.39 | 850.117 | -6 | 5104.7451 | 5106.94 | 5104.7403 | 1744735.62 |
| 3525-3540 | Ar-pAr-pCr-pCr-pAr-pCr-pAr-pCr-pAr-pAr-pGr-pCr-pCr-pCr-pCr- | Cyclic phosphorylation | 16 | 0.95 | 100 | 1.2 | 14.39 | 1020.343 | -5 | 5104.7451 | 5106.96 | 5104.7403 | 630438.94 |
| 2041-2059 | Ar-pCr-pAr-pUr-pCr-pCr-pCr-pCr-pAr-pUr-pCr-pGr-pGr-pCr-pGr | Cyclic phosphorylation | 19 | 1.42 | 100 | 1.1 | 14.46 | 673.75 | -9 | 6070.8096 | 6073.43 | 6070.8009 | 964548.75 |
| 2041-2059 | Ar-pCr-pAr-pUr-pCr-pCr-pCr-pCr-pAr-pUr-pCr-pGr-pGr-pCr-pGr | Cyclic phosphorylation | 19 | 1.26 | 100 | 1 | 14.49 | 758.094 | -8 | 6070.8086 | 6073.38 | 6070.8009 | 1579095.5 |
| 2041-2059 | Ar-pCr-pAr-pUr-pCr-pCr-pCr-pCr-pAr-pUr-pCr-pGr-pGr-pCr-pGr | Cyclic phosphorylation | 19 | 1.42 | 100 | 1.2 | 14.49 | 866.537 | -7 | 6070.8096 | 6073.49 | 6070.8009 | 745943.12 |
| 1362-1376 | Ar-pAr-pCr-pAr-pGr-pCr-pAr-pAr-pCr-pAr-pAr-pCr-pCr-pUr-pGr | Cyclic phosphorylation | 15 | 1.84 | 100 | 1.4 | 14.53 | 964.334 | -5 | 4824.7031 | 4826.75 | 4824.6942 | 304492 |
| 1362-1376 | Ar-pAr-pCr-pAr-pGr-pCr-pAr-pAr-pCr-pAr-pAr-pCr-pCr-pUr-pGr | Cyclic phosphorylation | 15 | 1.33 | 100 | 1.2 | 14.53 | 803.444 | -6 | 4824.7007 | 4826.81 | 4824.6942 | 756725.62 |
| 1362-1376 | Ar-pAr-pCr-pAr-pGr-pCr-pAr-pAr-pCr-pAr-pAr-pCr-pCr-pUr-pGr | Cyclic phosphorylation | 15 | 1.64 | 100 | 1.2 | 14.53 | 688.522 | -7 | 4824.7021 | 4826.76 | 4824.6942 | 1055568.88 |
| 1671-1686 | Ar-pAr-pCr-pUr-pUr-pCr-pAr-pAr-pCr-pUr-pUr-pCr-pAr-pAr-pC | Cyclic phosphorylation | 16 | 1.01 | 100 | 1.1 | 14.65 | 844.106 | -6 | 5068.6753 | 5070.84 | 5068.6702 | 1478023.12 |
| 1671-1686 | Ar-pAr-pCr-pUr-pUr-pCr-pAr-pAr-pCr-pUr-pUr-pCr-pAr-pAr-pC | Cyclic phosphorylation | 16 | 1.29 | 100 | 1.1 | 14.66 | 723.232 | -7 | 5068.6768 | 5070.77 | 5068.6702 | 2078289.75 |
| 1671-1686 | Ar-pAr-pCr-pUr-pUr-pCr-pAr-pAr-pCr-pUr-pUr-pCr-pAr-pAr-pC | Cyclic phosphorylation | 16 | 2.74 | 100 | 1.1 | 14.66 | 632.703 | -8 | 5068.6841 | 5070.97 | 5068.6702 | 307161.44 |
| 1743-1761 | Cr-pAr-pGr-pUr-pUr-pUr-pGr-pGr-pCr-pCr-pGr-pGr-pGr-pAr-pU | Cyclic phosphorylation | 19 | 1.11 | 100 | 1.6 | 14.66 | 1024.958 | -6 | 6152.7881 | 6155.25 | 6152.7813 | 171914.91 |
| 1743-1761 | Cr-pAr-pGr-pUr-pUr-pUr-pGr-pGr-pCr-pCr-pGr-pGr-pGr-pAr-pU | Cyclic phosphorylation | 19 | 0.79 | 100 | 1 | 14.66 | 768.342 | -8 | 6152.7861 | 6155.44 | 6152.7813 | 1370020 |
| 1725-1742 | Ar-pAr-pGr-pUr-pUr-pCr-pCr-pUr-pGr-pCr-pCr-pAr-pUr-pUr-pC | Cyclic phosphorylation | 18 | 1.7 | 100 | 1.1 | 14.7 | 633.853 | -9 | 5711.7363 | 5714.23 | 5711.7266 | 1360264 |
| 1725-1742 | Ar-pAr-pGr-pUr-pUr-pCr-pCr-pUr-pGr-pCr-pCr-pAr-pUr-pUr-pC | Cyclic phosphorylation | 18 | 1.53 | 100 | 1 | 14.7 | 713.21 | -8 | 5711.7354 | 5714.24 | 5711.7266 | 4511168 |
| 1725-1742 | Ar-pAr-pGr-pUr-pUr-pCr-pCr-pUr-pGr-pCr-pCr-pAr-pUr-pUr-pC | Cyclic phosphorylation | 18 | 1.19 | 100 | 1.1 | 14.7 | 815.241 | -7 | 5711.7334 | 5714.23 | 5711.7266 | 1963398.25 |
| 1725-1742 | Ar-pAr-pGr-pUr-pUr-pCr-pCr-pUr-pGr-pCr-pCr-pAr-pUr-pUr-pC | Cyclic phosphorylation | 18 | -0.18 | 100 | 1.1 | 14.7 | 951.282 | -6 | 5711.7256 | 5714.36 | 5711.7266 | 591106.38 |
| 1669-1686 | Ur-pGr-pAr-pAr-pCr-pUr-pUr-pCr-pAr-pAr-pCr-pUr-pUr-pCr-pA | Cyclic phosphorylation | 18 | 3.97 | 99.9 | 1.6 | 14.77 | 634.855 | -9 | 5719.7656 | 5721.83 | 5719.7429 | 67926.59 |
| 1669-1686 | Ur-pGr-pAr-pAr-pCr-pUr-pUr-pCr-pAr-pAr-pCr-pUr-pUr-pCr-pA | Cyclic phosphorylation | 18 | 0.98 | 99.9 | 1.2 | 14.77 | 714.213 | -8 | 5719.7485 | 5721.99 | 5719.7429 | 455770.97 |
| 3387-3405 | Cr-pCr-pCr-pCr-pAr-pGr-pAr-pUr-pCr-pAr-pUr-pCr-pAr-pCr-pCr | Cyclic phosphorylation | 19 | 1.28 | 100 | 1.1 | 14.77 | 748.972 | -8 | 5997.8286 | 6000.5 | 5997.8209 | 929761.69 |
| 1669-1686 | Ur-pGr-pAr-pAr-pCr-pUr-pUr-pCr-pAr-pAr-pCr-pUr-pUr-pCr-pA | Cyclic phosphorylation | 18 | 1.75 | 99.9 | 1.1 | 14.79 | 816.387 | -7 | 5719.7529 | 5722.28 | 5719.7429 | 653055.38 |
| 1669-1686 | Ur-pGr-pAr-pAr-pCr-pUr-pUr-pCr-pAr-pAr-pCr-pUr-pUr-pCr-pA | Cyclic phosphorylation | 18 | 1.41 | 99.9 | 1.2 | 14.79 | 952.619 | -6 | 5719.751 | 5722.34 | 5719.7429 | 265981.72 |
| 3387-3405 | Cr-pCr-pCr-pCr-pAr-pGr-pAr-pUr-pCr-pAr-pUr-pCr-pAr-pCr-pCr | Cyclic phosphorylation | 19 | 1.44 | 100 | 1.1 | 14.79 | 856.112 | -7 | 5997.8296 | 6000.45 | 5997.8209 | 740021 |
| 1638-1653 | Ar-pAr-pAr-pAr-pGr-pCr-pAr-pCr-pCr-pAr-pAr-pUr-pCr-pUr-pCr | Cyclic phosphorylation | 16 | 2.41 | 100 | 1.1 | 14.91 | 640.459 | -8 | 5130.7319 | 5132.88 | 5130.7195 | 834732.69 |
| 1638-1653 | Ar-pAr-pAr-pAr-pGr-pCr-pAr-pCr-pCr-pAr-pAr-pUr-pCr-pUr-pCr | Cyclic phosphorylation | 16 | 1.84 | 100 | 1 | 14.91 | 732.24 | -7 | 5130.729 | 5132.89 | 5130.7195 | 1974660.25 |
| 1638-1653 | Ar-pAr-pAr-pAr-pGr-pCr-pAr-pCr-pCr-pAr-pAr-pUr-pCr-pUr-pCr | Cyclic phosphorylation | 16 | 1.37 | 100 | 1.2 | 14.91 | 854.448 | -6 | 5130.7266 | 5132.9 | 5130.7195 | 1186841 |
| 1638-1653 | Ar-pAr-pAr-pAr-pGr-pCr-pAr-pCr-pCr-pAr-pAr-pUr-pCr-pUr-pCr | Cyclic phosphorylation | 16 | 2.03 | 100 | 1.3 | 14.91 | 1025.539 | -5 | 5130.73 | 5132.91 | 5130.7195 | 355401 |
| 1638-1653 | Ar-pAr-pAr-pAr-pGr-pCr-pAr-pCr-pCr-pAr-pAr-pUr-pCr-pUr-pCr | Cyclic phosphorylation | 16 | 2.03 | 100 | 1.5 | 14.91 | 1709.904 | -3 | 5130.73 | 5132.92 | 5130.7195 | 373178.84 |
| 1618-1637 | Cr-pCr-pAr-pCr-pAr-pGr-pUr-pGr-pUr-pGr-pCr-pGr-pGr-pCr-pC | Cyclic phosphorylation | 20 | 3.15 | 100 | 1.1 | 15.05 | 919.261 | -7 | 6439.8799 | 6442.66 | 6439.8596 | 578246.75 |
| 1618-1637 | Cr-pCr-pAr-pCr-pAr-pGr-pUr-pGr-pUr-pGr-pCr-pGr-pGr-pCr-pC | Cyclic phosphorylation | 20 | 3.07 | 100 | 1.1 | 15.05 | 804.228 | -8 | 6439.8794 | 6442.6 | 6439.8596 | 1196105.12 |
| 1618-1637 | Cr-pCr-pAr-pCr-pAr-pGr-pUr-pGr-pUr-pGr-pCr-pGr-pGr-pCr-pC | Cyclic phosphorylation | 20 | 3 | 100 | 1 | 15.05 | 714.757 | -9 | 6439.8789 | 6442.6 | 6439.8596 | 619269.69 |
| 2004-2021 | Ar-pUr-pCr-pGr-pGr-pAr-pGr-pCr-pCr-pGr-pAr-pGr-pCr-pAr-pC | Cyclic phosphorylation | 18 | 2.52 | 93.5 | 2 | 15.1 | 651.306 | -9 | 5868.814 | 5871.03 | 5868.7992 | 245004.56 |
| 2004-2021 | Ar-pUr-pCr-pGr-pGr-pAr-pGr-pCr-pCr-pGr-pAr-pGr-pCr-pAr-pC | Cyclic phosphorylation | 18 | 2.86 | 93.5 | 1 | 15.1 | 732.845 | -8 | 5868.8159 | 5871.3 | 5868.7992 | 516026.31 |
| 2004-2021 | Ar-pUr-pCr-pGr-pGr-pAr-pGr-pCr-pCr-pGr-pAr-pGr-pCr-pAr-pC | Cyclic phosphorylation | 18 | 2.77 | 93.5 | 1.4 | 15.11 | 837.681 | -7 | 5868.8154 | 5871.16 | 5868.7992 | 269551.53 |
| 287-305 | Ar-pUr-pUr-pCr-pGr-pAr-pCr-pAr-pAr-pCr-pCr-pCr-pCr-pGr-pU | Cyclic phosphorylation | 19 | 2.9 | 100 | 1.6 | 15.15 | 672.194 | -9 | 6055.8076 | 6058.82 | 6055.79 | 354459.44 |
| 287-305 | Ar-pUr-pUr-pCr-pGr-pAr-pCr-pAr-pAr-pCr-pCr-pCr-pCr-pGr-pU | Cyclic phosphorylation | 19 | 2.9 | 100 | 1.1 | 15.17 | 756.344 | -8 | 6055.8076 | 6058.73 | 6055.79 | 503819.47 |
| 3829-3848 | Ar-pCr-pGr-pAr-pUr-pUr-pCr-pUr-pGr-pAr-pGr-pCr-pCr-pCr-pG | Cyclic phosphorylation | 20 | 2.66 | 100 | 1.1 | 15.17 | 801.472 | -8 | 6417.8335 | 6420.65 | 6417.8164 | 536021.12 |
| 3829-3848 | Ar-pCr-pGr-pAr-pUr-pUr-pCr-pUr-pGr-pAr-pGr-pCr-pCr-pCr-pG | Cyclic phosphorylation | 20 | 3.43 | 100 | 1 | 15.19 | 712.419 | -9 | 6417.8384 | 6420.66 | 6417.8164 | 467227.69 |
| 3829-3848 | Ar-pCr-pGr-pAr-pUr-pUr-pCr-pUr-pGr-pAr-pGr-pCr-pCr-pCr-pG | Cyclic phosphorylation | 20 | 3.81 | 100 | 1.7 | 15.19 | 641.177 | -10 | 6417.8408 | 6420.6 | 6417.8164 | 88555.79 |
| 650-667 | Cr-pUr-pAr-pCr-pUr-pUr-pCr-pAr-pAr-pGr-pAr-pUr-pCr-pUr-pA | Cyclic phosphorylation | 18 | 1.66 | 100 | 1.1 | 15.29 | 714.212 | -8 | 5719.7524 | 5722.3 | 5719.7429 | 728540.19 |
| 650-667 | Cr-pUr-pAr-pCr-pUr-pUr-pCr-pAr-pAr-pGr-pAr-pUr-pCr-pUr-pA | Cyclic phosphorylation | 18 | 2.43 | 100 | 1.1 | 15.29 | 816.387 | -7 | 5719.7568 | 5722.26 | 5719.7429 | 433303.12 |
| 3375-3392 | Ar-pAr-pCr-pUr-pUr-pCr-pUr-pAr-pCr-pGr-pAr-pGr-pCr-pCr-pC | Cyclic phosphorylation | 18 | 1.82 | 99.9 | 1.1 | 15.32 | 818.39 | -7 | 5733.7803 | 5736.36 | 5733.7698 | 461408.81 |
| 3375-3392 | Ar-pAr-pCr-pUr-pUr-pCr-pUr-pAr-pCr-pGr-pAr-pGr-pCr-pCr-pC | Cyclic phosphorylation | 18 | 1.4 | 99.9 | 1.1 | 15.33 | 715.966 | -8 | 5733.7778 | 5736.31 | 5733.7698 | 519057.47 |
| 3985-4006 | Cr-pCr-pCr-pCr-pAr-pCr-pUr-pCr-pAr-pCr-pCr-pAr-pCr-pCr-pUr- | Cyclic phosphorylation | 22 | 2.66 | 99.9 | 1.4 | 15.36 | 688.385 | -10 | 6890.9199 | 6893.84 | 6890.9016 | 647320.44 |
| 3985-4006 | Cr-pCr-pCr-pCr-pAr-pCr-pUr-pCr-pAr-pCr-pCr-pAr-pCr-pCr-pUr- | Cyclic phosphorylation | 22 | 0.82 | 99.9 | 1 | 15.36 | 764.984 | -9 | 6890.9072 | 6894.08 | 6890.9016 | 749242.88 |
| 3738-3755 | Ar-pUr-pGr-pGr-pUr-pCr-pAr-pCr-pAr-pAr-pUr-pCr-pAr-pUr-pG | Cyclic phosphorylation | 18 | 1.8 | 100 | 1.2 | 15.39 | 640.966 | -9 | 5775.7544 | 5778.11 | 5775.744 | 572497 |
| 3738-3755 | Ar-pUr-pGr-pGr-pUr-pCr-pAr-pCr-pAr-pAr-pUr-pCr-pAr-pUr-pG | Cyclic phosphorylation | 18 | 2.14 | 100 | 1.1 | 15.41 | 721.212 | -8 | 5775.7563 | 5778.31 | 5775.744 | 1137778.62 |
| 3738-3755 | Ar-pUr-pGr-pGr-pUr-pCr-pAr-pCr-pAr-pAr-pUr-pCr-pAr-pUr-pG | Cyclic phosphorylation | 18 | 1.63 | 100 | 1.1 | 15.41 | 824.387 | -7 | 5775.7534 | 5778.14 | 5775.744 | 518192.91 |
| 1952-1972 | Gr-pCr-pGr-pGr-pGr-pUr-pGr-pUr-pAr-pCr-pUr-pCr-pCr-pAr-pC | Cyclic phosphorylation | 21 | 4.29 | 99.6 | 1 | 15.53 | 849.483 | -8 | 6800.9312 | 6804.09 | 6800.9019 | 479513.59 |
| 1952-1972 | Gr-pCr-pGr-pGr-pGr-pUr-pGr-pUr-pAr-pCr-pUr-pCr-pCr-pAr-pC | Cyclic phosphorylation | 21 | 2.64 | 99.6 | 1.2 | 15.53 | 754.873 | -9 | 6800.9199 | 6804.29 | 6800.9019 | 416233.03 |
| 3273-3291 | Ar-pAr-pUr-pUr-pUr-pCr-pAr-pCr-pCr-pAr-pCr-pCr-pGr-pCr-pU | Cyclic phosphorylation | 19 | 2.46 | 100 | 1.1 | 15.6 | 856.393 | -7 | 5999.8037 | 6002.45 | 5999.789 | 387646.97 |
| 3273-3291 | Ar-pAr-pUr-pUr-pUr-pCr-pAr-pCr-pCr-pAr-pCr-pCr-pGr-pCr-pU | Cyclic phosphorylation | 19 | 2.62 | 100 | 1 | 15.6 | 749.219 | -8 | 5999.8047 | 6002.35 | 5999.789 | 743330.25 |
| 1756-1773 | Ar-pUr-pAr-pUr-pCr-pGr-pCr-pCr-pGr-pAr-pUr-pAr-pCr-pCr-pA | Cyclic phosphorylation | 18 | 2.32 | 97.8 | 1.2 | 15.71 | 719.093 | -8 | 5757.7944 | 5760.58 | 5757.781 | 254813.59 |
| 954-975 | Ur-pGr-pCr-pAr-pCr-pCr-pCr-pUr-pGr-pAr-pAr-pGr-pUr-pCr-pC | Cyclic phosphorylation | 22 | 1.69 | 100 | 1 | 15.76 | 870.732 | -8 | 6971.9097 | 6974.98 | 6971.8979 | 1231568.88 |
| 954-975 | Ur-pGr-pCr-pAr-pCr-pCr-pCr-pUr-pGr-pAr-pAr-pGr-pUr-pCr-pC | Cyclic phosphorylation | 22 | 1.76 | 100 | 1 | 15.76 | 773.983 | -9 | 6971.9102 | 6975.02 | 6971.8979 | 909246.56 |
| 3484-3501 | Ar-pGr-pCr-pUr-pGr-pGr-pAr-pCr-pAr-pGr-pCr-pUr-pUr-pCr-pA | Cyclic phosphorylation | 18 | 1.11 | 98.3 | 1 | 15.81 | 833.108 | -7 | 5837.7998 | 5840.37 | 5837.7933 | 652769.44 |
| 3858-3874 | Ar-pAr-pAr-pCr-pUr-pGr-pCr-pAr-pCr-pUr-pAr-pCr-pAr-pCr-pAr | Cyclic phosphorylation | 17 | 1.76 | 100 | 1.1 | 15.81 | 905.452 | -6 | 5436.7544 | 5439 | 5436.7448 | 714363.62 |
| 3858-3874 | Ar-pAr-pAr-pCr-pUr-pGr-pCr-pAr-pCr-pUr-pAr-pCr-pAr-pCr-pAr | Cyclic phosphorylation | 17 | 1.49 | 100 | 1.1 | 15.83 | 678.837 | -8 | 5436.7529 | 5439 | 5436.7448 | 2133564.75 |
| 3858-3874 | Ar-pAr-pAr-pCr-pUr-pGr-pCr-pAr-pCr-pUr-pAr-pCr-pAr-pCr-pAr | Cyclic phosphorylation | 17 | 0.86 | 100 | 1 | 15.83 | 775.958 | -7 | 5436.7495 | 5439.04 | 5436.7448 | 1816894.75 |
| 2060-2078 | Ar-pAr-pUr-pCr-pUr-pGr-pCr-pGr-pCr-pCr-pAr-pGr-pCr-pUr-pA | Cyclic phosphorylation | 19 | 1.31 | 100 | 1.3 | 15.86 | 867.683 | -7 | 6078.8252 | 6081.44 | 6078.8172 | 915482.44 |
| 2060-2078 | Ar-pAr-pUr-pCr-pUr-pGr-pCr-pGr-pCr-pCr-pAr-pGr-pCr-pUr-pA | Cyclic phosphorylation | 19 | 1.31 | 100 | 1 | 15.86 | 759.096 | -8 | 6078.8252 | 6081.46 | 6078.8172 | 1975583.25 |
| 2060-2078 | Ar-pAr-pUr-pCr-pUr-pGr-pCr-pGr-pCr-pCr-pAr-pGr-pCr-pUr-pA | Cyclic phosphorylation | 19 | 2.11 | 100 | 1.5 | 15.86 | 674.641 | -9 | 6078.8301 | 6081.44 | 6078.8172 | 822321.62 |
| 3594-3611 | Ar-pAr-pAr-pGr-pAr-pGr-pAr-pUr-pCr-pGr-pAr-pCr-pCr-pGr-pG | Cyclic phosphorylation | 18 | 2.07 | 99.8 | 1.3 | 15.97 | 733.972 | -8 | 5876.8276 | 5879.22 | 5876.8155 | 701467.5 |

| | | | | | | | | | | | | | |
|---|---|---|---|---|---|---|---|---|---|---|---|---|---|
| 2170-2190 | Cr-pCr-pUr-pAr-pCr-pUr-pCr-pCr-pAr-pAr-pCr-pAr-pAr-pCr-pUr | Cyclic phosphorylation | 21 | 0.7 | 100 | 1.2 | 16.04 | 823.602 | -8 | 6594.8652 | 6597.84 | 6594.8606 | 1094918.12 |
| 2170-2190 | Cr-pCr-pUr-pAr-pCr-pUr-pCr-pCr-pAr-pAr-pCr-pAr-pAr-pCr-pUr | Cyclic phosphorylation | 21 | 1.81 | 100 | 1.1 | 16.04 | 732.09 | -9 | 6594.8726 | 6597.82 | 6594.8606 | 766653.69 |
| 1638-1655 | Ar-pAr-pAr-pGr-pCr-pAr-pCr-pCr-pAr-pAr-pUr-pCr-pUr-pCr | Cyclic phosphorylation | 18 | 1.98 | 99.1 | 1.1 | 16.18 | 721.969 | -8 | 5781.8037 | 5784.35 | 5781.7923 | 613534.44 |
| 1638-1655 | Ar-pAr-pAr-pGr-pCr-pAr-pCr-pCr-pAr-pAr-pUr-pCr-pUr-pCr | Cyclic phosphorylation | 18 | 1.81 | 99.1 | 1.2 | 16.19 | 825.251 | -7 | 5781.8027 | 5784.38 | 5781.7923 | 384964.34 |
| 897-915 | Ar-pCr-pGr-pGr-pCr-pAr-pCr-pAr-pUr-pCr-pAr-pCr-pCr | Cyclic phosphorylation | 19 | 0.62 | 100 | 1 | 16.21 | 870.832 | -7 | 6100.8643 | 6103.55 | 6100.8605 | 722754.31 |
| 897-915 | Ar-pCr-pGr-pGr-pCr-pAr-pCr-pAr-pUr-pCr-pAr-pCr-pCr | Cyclic phosphorylation | 19 | 1.34 | 100 | 1 | 16.23 | 761.852 | -8 | 6100.8687 | 6103.54 | 6100.8605 | 1185102.12 |
| 3878-3897 | Ar-pCr-pUr-pCr-pGr-pAr-pGr-pCr-pUr-pGr-pGr-pUr-pAr-pCr-pU | Cyclic phosphorylation | 20 | 1.59 | 100 | 1 | 16.3 | 714.975 | -9 | 6441.8379 | 6444.62 | 6441.8276 | 4767283 |
| 3878-3897 | Ar-pCr-pUr-pCr-pGr-pAr-pGr-pCr-pUr-pGr-pGr-pUr-pAr-pCr-pU | Cyclic phosphorylation | 20 | 2.35 | 100 | 1.2 | 16.3 | 643.377 | -10 | 6441.8428 | 6444.61 | 6441.8276 | 788638.06 |
| 3878-3897 | Ar-pCr-pUr-pCr-pGr-pAr-pGr-pCr-pUr-pGr-pGr-pUr-pAr-pCr-pU | Cyclic phosphorylation | 20 | 1.82 | 100 | 1 | 16.3 | 804.472 | -8 | 6441.8394 | 6444.59 | 6441.8276 | 4568223.5 |
| 3878-3897 | Ar-pCr-pUr-pCr-pGr-pAr-pGr-pCr-pUr-pGr-pGr-pUr-pAr-pCr-pU | Cyclic phosphorylation | 20 | 1.97 | 100 | 1.1 | 16.3 | 919.541 | -7 | 6441.8403 | 6444.62 | 6441.8276 | 1781603.88 |
| 4059-4079 | Cr-pCr-pAr-pCr-pAr-pCr-pCr-pCr-pCr-pAr-pCr-pGr-pGr-pGr- | Cyclic phosphorylation | 21 | 1.88 | 100 | 1 | 16.35 | 747.435 | -9 | 6733.9829 | 6736.86 | 6733.9702 | 1328527.88 |
| 4059-4079 | Cr-pCr-pAr-pCr-pAr-pCr-pCr-pCr-pCr-pAr-pCr-pGr-pGr-pGr- | Cyclic phosphorylation | 21 | 0.29 | 100 | 1.1 | 16.36 | 961.276 | -7 | 6733.9722 | 6736.97 | 6733.9702 | 665511.44 |
| 4059-4079 | Cr-pCr-pAr-pCr-pAr-pCr-pCr-pCr-pCr-pAr-pCr-pGr-pGr-pGr- | Cyclic phosphorylation | 21 | 0.87 | 100 | 1 | 16.36 | 841.116 | -8 | 6733.9761 | 6737 | 6733.9702 | 1285297.88 |
| 1038-1058 | Ur-pUr-pCr-pCr-pCr-pAr-pAr-pUr-pAr-pUr-pCr-pAr-pCr-pCr | Cyclic phosphorylation | 21 | 1.05 | 100 | 1 | 16.43 | 823.725 | -8 | 6595.8516 | 6598.65 | 6595.8447 | 1472991.5 |
| 1038-1058 | Ur-pUr-pCr-pCr-pCr-pAr-pAr-pUr-pAr-pUr-pCr-pAr-pCr-pCr | Cyclic phosphorylation | 21 | 1.64 | 100 | 1 | 16.43 | 732.088 | -9 | 6595.8555 | 6598.68 | 6595.8447 | 3435815.75 |
| 1038-1058 | Ur-pUr-pCr-pCr-pCr-pAr-pAr-pUr-pAr-pUr-pCr-pAr-pCr-pCr | Cyclic phosphorylation | 21 | 1.34 | 100 | 1.2 | 16.43 | 658.779 | -10 | 6595.8535 | 6598.7 | 6595.8447 | 1366444.88 |
| 240-261 | Ar-pCr-pUr-pGr-pGr-pUr-pCr-pCr-pAr-pCr-pGr-pCr-pCr-pCr | Cyclic phosphorylation | 22 | 1.64 | 99.8 | 1 | 16.5 | 773.762 | -9 | 6970.9253 | 6973.84 | 6970.9139 | 1108115.38 |
| 561-584 | Ur-pAr-pCr-pGr-pUr-pGr-pUr-pCr-pCr-pAr-pGr-pCr-pCr-pUr | Cyclic phosphorylation | 24 | 1.26 | 100 | 1 | 16.54 | 759.387 | -10 | 7600.937 | 7604.15 | 7600.9274 | 703817.5 |
| 561-584 | Ur-pAr-pCr-pGr-pUr-pGr-pUr-pCr-pCr-pAr-pGr-pCr-pCr-pUr | Cyclic phosphorylation | 24 | 0.68 | 100 | 1.2 | 16.55 | 843.987 | -9 | 7600.9326 | 7604.49 | 7600.9274 | 351538.84 |
| 1038-1058 | Ur-pUr-pCr-pCr-pCr-pAr-pAr-pUr-pAr-pUr-pCr-pAr-pCr-pCr | Phosphorylation | 21 | 1.51 | 99.4 | 1.1 | 16.68 | 826.102 | -8 | 6613.8652 | 6616.8 | 6613.8552 | 266256.03 |
| 3304-3325 | Ar-pCr-pGr-pGr-pCr-pAr-pAr-pAr-pGr-pCr-pCr-pAr-pCr-pUr | Cyclic phosphorylation | 22 | 1.62 | 100 | 1 | 16.85 | 701.188 | -10 | 7018.9478 | 7021.93 | 7018.9364 | 1597691.12 |
| 3304-3325 | Ar-pCr-pGr-pGr-pCr-pAr-pAr-pAr-pGr-pCr-pCr-pAr-pCr-pUr | Cyclic phosphorylation | 22 | 0.93 | 100 | 1.1 | 16.85 | 876.737 | -8 | 7018.9429 | 7021.85 | 7018.9364 | 1103092.5 |
| 3304-3325 | Ar-pCr-pGr-pGr-pCr-pAr-pAr-pAr-pGr-pCr-pCr-pAr-pCr-pUr | Cyclic phosphorylation | 22 | 1.42 | 100 | 1 | 16.85 | 779.098 | -9 | 7018.9463 | 7021.94 | 7018.9364 | 1848215.88 |
| 3736-3757 | Ur-pGr-pAr-pUr-pGr-pGr-pUr-pCr-pAr-pCr-pAr-pAr-pUr-pCr-pA | Cyclic phosphorylation | 22 | 1.2 | 100 | 1.1 | 17.01 | 785.76 | -9 | 7077.8979 | 7080.96 | 7077.8895 | 1382359 |
| 3736-3757 | Ur-pGr-pAr-pUr-pGr-pGr-pUr-pCr-pAr-pCr-pAr-pAr-pUr-pCr-pA | Cyclic phosphorylation | 22 | 0.3 | 100 | 1 | 17.01 | 884.106 | -8 | 7077.8916 | 7080.81 | 7077.8895 | 1069182.88 |
| 4037-4058 | Cr-pAr-pGr-pCr-pUr-pCr-pAr-pAr-pAr-pCr-pGr-pCr-pUr-pUr | Cyclic phosphorylation | 22 | 2.1 | 99.9 | 1.3 | 17.16 | 879.739 | -8 | 7042.9624 | 7046.03 | 7042.9476 | 343994.81 |
| 4037-4058 | Cr-pAr-pGr-pCr-pUr-pCr-pAr-pAr-pAr-pCr-pGr-pCr-pUr-pUr | Cyclic phosphorylation | 22 | 2.17 | 99.9 | 1.2 | 17.16 | 781.878 | -9 | 7042.9629 | 7046.13 | 7042.9476 | 591462.81 |
| 4037-4058 | Cr-pAr-pGr-pCr-pUr-pCr-pAr-pAr-pAr-pCr-pGr-pCr-pUr-pUr | Cyclic phosphorylation | 22 | 2.03 | 99.9 | 1.4 | 17.16 | 703.49 | -10 | 7042.9619 | 7046.01 | 7042.9476 | 359223.44 |
| 495-512 | Ar-pAr-pCr-pAr-pCr-pAr-pAr-pGr-pAr-pGr-pCr-pUr-pGr-pGr | Cyclic phosphorylation | 18 | -0.62 | 99.8 | 1 | 17.28 | 839.971 | -7 | 5884.8281 | 5887.45 | 5884.8318 | 581155.44 |
| 495-512 | Ar-pAr-pCr-pAr-pCr-pAr-pAr-pGr-pAr-pGr-pCr-pUr-pGr-pGr | Cyclic phosphorylation | 18 | 1.62 | 99.8 | 1.1 | 17.28 | 734.849 | -8 | 5884.8413 | 5887.41 | 5884.8318 | 459538.34 |
| 2167-2190 | Ur-pGr-pGr-pCr-pCr-pUr-pAr-pCr-pUr-pCr-pCr-pAr-pAr-pCr-pAr | Cyclic phosphorylation | 24 | 1.18 | 99.6 | 1 | 17.3 | 842.659 | -9 | 7590.9897 | 7594.3 | 7590.9808 | 505178.03 |
| 2167-2190 | Ur-pGr-pGr-pCr-pCr-pUr-pAr-pCr-pUr-pCr-pCr-pAr-pAr-pCr-pAr | Cyclic phosphorylation | 24 | 4.97 | 99.6 | 1 | 17.3 | 758.493 | -10 | 7591.0186 | 7594.14 | 7590.9808 | 450756.03 |
| 1671-1691 | Ar-pAr-pCr-pUr-pUr-pCr-pAr-pAr-pCr-pUr-pUr-pCr-pAr-pAr-pCr | Cyclic phosphorylation | 21 | 2.06 | 100 | 1 | 17.35 | 740.869 | -9 | 6674.8867 | 6677.87 | 6674.8729 | 1376582.38 |
| 1671-1691 | Ar-pAr-pCr-pUr-pUr-pCr-pAr-pAr-pCr-pUr-pUr-pCr-pAr-pAr-pCr | Cyclic phosphorylation | 21 | 1.63 | 100 | 1.3 | 17.35 | 952.977 | -7 | 6674.8838 | 6677.7 | 6674.8729 | 388380.5 |
| 1671-1691 | Ar-pAr-pCr-pUr-pUr-pCr-pAr-pAr-pCr-pUr-pUr-pCr-pAr-pAr-pCr | Cyclic phosphorylation | 21 | 1.48 | 100 | 1 | 17.35 | 833.604 | -8 | 6674.8828 | 6677.7 | 6674.8729 | 1094710.12 |
| 3525-3545 | Ar-pAr-pCr-pCr-pAr-pCr-pAr-pCr-pAr-pAr-pGr-pCr-pCr-pCr- | Cyclic phosphorylation | 21 | 0.34 | 99.2 | 1.1 | 17.49 | 841.239 | -8 | 6734.9565 | 6738.1 | 6734.9543 | 507332.06 |
| 3525-3545 | Ar-pAr-pCr-pCr-pAr-pCr-pAr-pCr-pAr-pAr-pGr-pCr-pCr-pCr- | Cyclic phosphorylation | 21 | 1.79 | 99.2 | 1 | 17.49 | 747.546 | -9 | 6734.9663 | 6737.83 | 6734.9543 | 606180.5 |
| 1035-1058 | Cr-pGr-pGr-pUr-pUr-pCr-pCr-pCr-pAr-pAr-pUr-pAr-pUr-pCr | Cyclic phosphorylation | 24 | 0.66 | 99.3 | 1.3 | 17.51 | 948.117 | -8 | 7590.9858 | 7594.34 | 7590.9808 | 847558.88 |
| 1035-1058 | Cr-pGr-pGr-pUr-pUr-pCr-pCr-pCr-pAr-pAr-pUr-pAr-pUr-pCr | Cyclic phosphorylation | 24 | 1.31 | 99.3 | 1.1 | 17.53 | 842.77 | -9 | 7590.9907 | 7594.38 | 7590.9808 | 1080533 |
| 1035-1058 | Cr-pGr-pGr-pUr-pUr-pCr-pCr-pCr-pAr-pAr-pUr-pAr-pUr-pCr | Cyclic phosphorylation | 24 | 1.76 | 99.3 | 1 | 17.53 | 758.393 | -10 | 7590.9941 | 7594.38 | 7590.9808 | 871705.12 |
| 1533-1557 | Ur-pCr-pCr-pUr-pAr-pCr-pGr-pGr-pCr-pUr-pUr-pUr-pCr-pAr-pG | Cyclic phosphorylation | 25 | 6.53 | 93.1 | 1 | 17.68 | 792.798 | -10 | 7936.0801 | 7939.45 | 7936.0282 | 767977.44 |
| 1533-1557 | Ur-pCr-pCr-pUr-pAr-pCr-pGr-pGr-pCr-pUr-pUr-pUr-pCr-pAr-pG | Cyclic phosphorylation | 25 | 4.93 | 93.1 | 1 | 17.68 | 881.109 | -9 | 7936.0674 | 7939.51 | 7936.0282 | 691678.25 |
| 1533-1557 | Ur-pCr-pCr-pUr-pAr-pCr-pGr-pGr-pCr-pUr-pUr-pUr-pCr-pAr-pG | Cyclic phosphorylation | 25 | 3.27 | 93.1 | 1.2 | 17.68 | 720.725 | -11 | 7936.0542 | 7939.62 | 7936.0282 | 730281.94 |
| 3823-3845 | Ar-pCr-pGr-pAr-pGr-pGr-pAr-pCr-pGr-pAr-pUr-pUr-pCr-pUr-pG | Cyclic phosphorylation | 23 | 1.21 | 99.9 | 1 | 17.77 | 931.743 | -8 | 7460 | 7463.54 | 7459.991 | 442797.53 |
| 3823-3845 | Ar-pCr-pGr-pAr-pGr-pGr-pAr-pCr-pGr-pAr-pUr-pUr-pCr-pUr-pG | Cyclic phosphorylation | 23 | 2.12 | 99.9 | 1 | 17.77 | 745.394 | -10 | 7460.0068 | 7463.31 | 7459.991 | 874922.62 |
| 3823-3845 | Ar-pCr-pGr-pAr-pGr-pGr-pAr-pCr-pGr-pAr-pUr-pUr-pCr-pUr-pG | Cyclic phosphorylation | 23 | 2.12 | 99.9 | 1 | 17.77 | 828.216 | -9 | 7460.0068 | 7463.47 | 7459.991 | 886202.19 |
| 2191-2215 | Cr-pUr-pAr-pUr-pCr-pCr-pCr-pAr-pCr-pCr-pAr-pAr-pCr-pUr | Cyclic phosphorylation | 25 | 0.82 | 100 | 1.1 | 17.82 | 783.198 | -10 | 7839.0435 | 7842.26 | 7839.037 | 1077846.38 |
| 2191-2215 | Cr-pUr-pAr-pUr-pCr-pCr-pCr-pAr-pCr-pCr-pAr-pAr-pCr-pUr | Cyclic phosphorylation | 25 | 2.38 | 100 | 1.1 | 17.82 | 870.332 | -9 | 7839.0557 | 7842.41 | 7839.037 | 1006324.5 |
| 2191-2215 | Cr-pUr-pAr-pUr-pCr-pCr-pCr-pAr-pCr-pCr-pAr-pAr-pCr-pUr | Cyclic phosphorylation | 25 | 1.94 | 100 | 1.2 | 17.84 | 711.816 | -11 | 7839.0522 | 7842.61 | 7839.037 | 1038859.25 |
| 3797-3822 | Ur-pAr-pGr-pCr-pUr-pGr-pUr-pGr-pGr-pCr-pAr-pGr-pCr-pUr-pG | Cyclic phosphorylation | 26 | 2.06 | 100 | 1 | 17.98 | 932.001 | -9 | 8394.0791 | 8397.94 | 8394.0618 | 964856.12 |
| 3797-3822 | Ur-pAr-pGr-pCr-pUr-pGr-pUr-pGr-pGr-pCr-pAr-pGr-pCr-pUr-pG | Cyclic phosphorylation | 26 | 2.06 | 100 | 1 | 17.98 | 838.701 | -10 | 8394.0791 | 8397.8 | 8394.0618 | 1853917.62 |
| 3797-3822 | Ur-pAr-pGr-pCr-pUr-pGr-pUr-pGr-pGr-pCr-pAr-pGr-pCr-pUr-pG | Cyclic phosphorylation | 26 | 0.78 | 100 | 1 | 17.98 | 762.364 | -11 | 8394.0684 | 8397.54 | 8394.0618 | 1050540.5 |
| 13-38 | Ur-pAr-pUr-pCr-pUr-pCr-pUr-pUr-pGr-pGr-pUr-pCr-pCr-pC | Cyclic phosphorylation | 26 | 1.79 | 99.9 | 1 | 18.03 | 821.097 | -10 | 8219.041 | 8222.71 | 8219.0263 | 813996.75 |
| 639-659 | Ar-pAr-pCr-pAr-pUr-pCr-pGr-pAr-pCr-pGr-pGr-pCr-pUr-pAr-pCr | Cyclic phosphorylation | 21 | 1.73 | 99.1 | 1 | 18.03 | 841.608 | -8 | 6737.918 | 6740.82 | 6737.9063 | 1018604 |
| 639-659 | Ar-pAr-pCr-pAr-pUr-pCr-pGr-pAr-pCr-pGr-pGr-pCr-pUr-pAr-pCr | Cyclic phosphorylation | 21 | 1.15 | 99.1 | 1.2 | 18.03 | 747.984 | -9 | 6737.9141 | 6740.96 | 6737.9063 | 721602.31 |
| 639-659 | Ar-pAr-pCr-pAr-pUr-pCr-pGr-pAr-pCr-pGr-pGr-pCr-pUr-pAr-pCr | Cyclic phosphorylation | 21 | 2.17 | 99.1 | 1.8 | 18.03 | 672.985 | -10 | 6737.9209 | 6741.03 | 6737.9063 | 163850.77 |
| 13-38 | Ur-pAr-pUr-pCr-pUr-pCr-pUr-pUr-pGr-pGr-pUr-pCr-pCr-pC | Cyclic phosphorylation | 26 | 2.74 | 99.9 | 1 | 18.06 | 746.451 | -11 | 8219.0488 | 8222.52 | 8219.0263 | 624599.19 |
| 2229-2252 | Ar-pUr-pCr-pCr-pUr-pGr-pCr-pCr-pUr-pGr-pUr-pGr-pUr-pCr-pC | Cyclic phosphorylation | 24 | 1.73 | 100 | 1.7 | 18.11 | 694.448 | -11 | 7646.9951 | 7650.27 | 7646.9819 | 539311.75 |
| 1305-1325 | Ar-pUr-pCr-pGr-pCr-pCr-pGr-pAr-pCr-pUr-pAr-pCr-pAr-pAr-pCr | Cyclic phosphorylation | 21 | 2.38 | 90.7 | 1.2 | 18.13 | 745.987 | -9 | 6720.9434 | 6724.18 | 6720.9274 | 519317.78 |
| 1305-1325 | Ar-pUr-pCr-pGr-pCr-pCr-pGr-pAr-pCr-pUr-pAr-pCr-pAr-pAr-pCr | Cyclic phosphorylation | 21 | 0.49 | 90.7 | 1.2 | 18.13 | 839.361 | -8 | 6720.9307 | 6723.68 | 6720.9274 | 261506.77 |
| 2229-2252 | Ar-pUr-pCr-pCr-pUr-pGr-pCr-pCr-pUr-pGr-pUr-pGr-pUr-pCr-pC | Cyclic phosphorylation | 24 | 2.24 | 100 | 1 | 18.13 | 763.993 | -10 | 7646.999 | 7650.45 | 7646.9819 | 1042330.25 |
| 2229-2252 | Ar-pUr-pCr-pCr-pUr-pGr-pCr-pCr-pUr-pGr-pUr-pGr-pUr-pCr-pC | Cyclic phosphorylation | 24 | 1.35 | 100 | 1 | 18.13 | 848.993 | -9 | 7646.9922 | 7650.37 | 7646.9819 | 686650.38 |
| 1952-1977 | Gr-pCr-pGr-pGr-pGr-pUr-pGr-pUr-pAr-pCr-pUr-pCr-pCr-pAr-pC | Cyclic phosphorylation | 26 | 0.24 | 98.9 | 1 | 18.3 | 934.342 | -9 | 8415.123 | 8418.87 | 8415.121 | 733652 |
| 1952-1977 | Gr-pCr-pGr-pGr-pGr-pUr-pGr-pUr-pAr-pCr-pUr-pCr-pCr-pAr-pC | Cyclic phosphorylation | 26 | 0.94 | 98.9 | 1.1 | 18.3 | 840.907 | -10 | 8415.1289 | 8418.9 | 8415.121 | 1038595.44 |
| 1033-1058 | Ur-pGr-pCr-pGr-pGr-pUr-pUr-pCr-pCr-pCr-pAr-pAr-pUr-pA | Cyclic phosphorylation | 26 | 2.39 | 100 | 1.4 | 18.39 | 748.636 | -11 | 8242.0732 | 8245.76 | 8242.0535 | 635142.25 |
| 1033-1058 | Ur-pGr-pCr-pGr-pGr-pUr-pUr-pCr-pCr-pCr-pAr-pAr-pUr-pA | Cyclic phosphorylation | 26 | 1.32 | 100 | 1 | 18.39 | 823.5 | -10 | 8242.0645 | 8245.9 | 8242.0535 | 1621503.5 |
| 1033-1058 | Ur-pGr-pCr-pGr-pGr-pUr-pUr-pCr-pCr-pCr-pAr-pAr-pUr-pA | Cyclic phosphorylation | 26 | 0.73 | 100 | 1.2 | 18.39 | 915.223 | -9 | 8242.0596 | 8245.87 | 8242.0535 | 1653792.12 |
| 1033-1058 | Ur-pGr-pCr-pGr-pGr-pUr-pUr-pCr-pCr-pCr-pAr-pAr-pUr-pA | Cyclic phosphorylation | 26 | 1.56 | 100 | 1.4 | 18.39 | 1029.626 | -8 | 8242.0664 | 8245.72 | 8242.0535 | 746475.06 |
| 3758-3785 | Ur-pUr-pGr-pCr-pAr-pUr-pGr-pAr-pCr-pCr-pAr-pGr-pCr-pUr-pG | Cyclic phosphorylation | 28 | 6.08 | 100 | 1 | 18.48 | 746.338 | -12 | 8965.1768 | 8969.08 | 8965.1223 | 2881220.5 |
| 3758-3785 | Ur-pUr-pGr-pCr-pAr-pUr-pGr-pAr-pCr-pCr-pAr-pGr-pCr-pUr-pG | Cyclic phosphorylation | 28 | 2.27 | 100 | 1 | 18.48 | 814.278 | -11 | 8965.1426 | 8969.01 | 8965.1223 | 2195684 |
| 3758-3785 | Ur-pUr-pGr-pCr-pAr-pUr-pGr-pAr-pCr-pCr-pAr-pGr-pCr-pUr-pG | Cyclic phosphorylation | 28 | 1.61 | 100 | 1 | 18.48 | 895.907 | -10 | 8965.1367 | 8968.97 | 8965.1223 | 1289182.75 |
| 4013-4036 | Ar-pCr-pAr-pCr-pCr-pUr-pCr-pCr-pCr-pAr-pAr-pGr-pCr-pAr-pCr- | Cyclic phosphorylation | 24 | 1.38 | 98.3 | 1.7 | 18.51 | 958.753 | -8 | 7675.084 | 7678.68 | 7675.0734 | 642632.38 |
| 4013-4036 | Ar-pCr-pAr-pCr-pCr-pUr-pCr-pCr-pCr-pAr-pAr-pGr-pCr-pAr-pCr- | Cyclic phosphorylation | 24 | 2.72 | 98.3 | 1 | 18.51 | 697.002 | -11 | 7675.0942 | 7678.61 | 7675.0734 | 547090.62 |
| 4013-4036 | Ar-pCr-pAr-pCr-pCr-pUr-pCr-pCr-pCr-pAr-pAr-pGr-pCr-pAr-pCr- | Cyclic phosphorylation | 24 | -0.02 | 98.3 | 1.1 | 18.51 | 852.114 | -9 | 7675.0732 | 7678.37 | 7675.0734 | 1141758.12 |
| 4013-4036 | Ar-pCr-pAr-pCr-pCr-pUr-pCr-pCr-pCr-pAr-pAr-pGr-pCr-pAr-pCr- | Cyclic phosphorylation | 24 | 1.13 | 98.3 | 1 | 18.51 | 766.802 | -10 | 7675.082 | 7678.4 | 7675.0734 | 1229799.12 |
| 3273-3298 | Ar-pAr-pUr-pUr-pUr-pCr-pAr-pCr-pAr-pCr-pCr-pGr-pCr-pUr | Cyclic phosphorylation | 26 | 1.44 | 100 | 1 | 18.63 | 910.557 | -9 | 8201.0752 | 8204.76 | 8201.0634 | 1640713 |
| 3273-3298 | Ar-pAr-pUr-pUr-pUr-pCr-pAr-pCr-pAr-pCr-pCr-pGr-pCr-pUr | Cyclic phosphorylation | 26 | 1.44 | 100 | 1.3 | 18.63 | 1024.503 | -8 | 8201.0752 | 8204.75 | 8201.0634 | 501780.31 |
| 3273-3298 | Ar-pAr-pUr-pUr-pUr-pCr-pAr-pCr-pAr-pCr-pCr-pGr-pCr-pUr | Cyclic phosphorylation | 26 | -0.11 | 100 | 1 | 18.63 | 819.401 | -10 | 8201.0625 | 8204.87 | 8201.0634 | 3168917.75 |
| 3273-3298 | Ar-pAr-pUr-pUr-pUr-pCr-pAr-pCr-pAr-pCr-pCr-pGr-pCr-pUr | Cyclic phosphorylation | 26 | -1.77 | 100 | 1 | 18.65 | 744.818 | -11 | 8201.0488 | 8204.64 | 8201.0634 | 3175425.5 |
| 3273-3298 | Ar-pAr-pUr-pUr-pUr-pCr-pAr-pCr-pAr-pCr-pCr-pGr-pCr-pUr | Cyclic phosphorylation | 26 | 1.44 | 100 | 1.2 | 18.65 | 682.666 | -12 | 8201.0752 | 8204.6 | 8201.0634 | 775222.38 |
| 1638-1658 | Ar-pAr-pAr-pGr-pCr-pAr-pCr-pCr-pAr-pAr-pUr-pCr-pUr-pCr | Cyclic phosphorylation | 21 | 0.01 | 96.4 | 1 | 18.68 | 847.489 | -8 | 6784.9448 | 6787.57 | 6784.9448 | 499814.84 |
| 1638-1658 | Ar-pAr-pAr-pGr-pCr-pAr-pCr-pCr-pAr-pAr-pUr-pCr-pUr-pCr | Cyclic phosphorylation | 21 | 0.8 | 96.4 | 1 | 18.68 | 753.211 | -9 | 6784.9502 | 6788.1 | 6784.9448 | 708268.5 |
| 3594-3615 | Ar-pAr-pAr-pGr-pAr-pGr-pAr-pUr-pCr-pGr-pAr-pCr-pCr-pGr-pG | Cyclic phosphorylation | 22 | 3.88 | 98.4 | 1.2 | 18.72 | 1025.998 | -7 | 7185.0371 | 7188.15 | 7185.0092 | 179792.19 |
| 3594-3615 | Ar-pAr-pAr-pGr-pAr-pGr-pAr-pUr-pCr-pGr-pAr-pCr-pCr-pGr-pG | Cyclic phosphorylation | 22 | 2.72 | 98.4 | 1 | 18.73 | 897.496 | -8 | 7185.0288 | 7187.81 | 7185.0092 | 607372.19 |
| 3594-3615 | Ar-pAr-pAr-pGr-pAr-pGr-pAr-pUr-pCr-pGr-pAr-pCr-pCr-pGr-pG | Cyclic phosphorylation | 22 | 1.3 | 98.4 | 1 | 18.73 | 797.551 | -9 | 7185.0186 | 7188.18 | 7185.0092 | 913972.94 |
| 3594-3615 | Ar-pAr-pAr-pGr-pAr-pGr-pAr-pUr-pCr-pGr-pAr-pCr-pCr-pGr-pG | Cyclic phosphorylation | 22 | 0.82 | 98.4 | 1.2 | 18.74 | 717.695 | -10 | 7185.0151 | 7188.07 | 7185.0092 | 432724.78 |
| 3299-3325 | Cr-pCr-pAr-pCr-pGr-pAr-pCr-pGr-pGr-pCr-pAr-pAr-pAr-pGr-pCr | Cyclic phosphorylation | 27 | 1.81 | 99.9 | 1.2 | 18.77 | 716.758 | -12 | 8608.1758 | 8611.83 | 8608.1602 | 659062.06 |
| 3299-3325 | Cr-pCr-pAr-pCr-pGr-pAr-pCr-pGr-pGr-pCr-pAr-pAr-pAr-pGr-pCr | Cyclic phosphorylation | 27 | 0.68 | 99.9 | 1 | 18.79 | 781.827 | -11 | 8608.166 | 8611.88 | 8608.1602 | 1248763.12 |
| 3299-3325 | Cr-pCr-pAr-pCr-pGr-pAr-pCr-pGr-pGr-pCr-pAr-pAr-pAr-pGr-pCr | Cyclic phosphorylation | 27 | -0.46 | 99.9 | 1.1 | 18.79 | 860.211 | -10 | 8608.1562 | 8612.15 | 8608.1602 | 1259439.75 |
| 4085-4105 | Ar-pUr-pUr-pAr-pAr-pCr-pCr-pUr-pUr-pUr-pAr-pGr-pCr-pAr-pA | Cyclic phosphorylation | 21 | 0.91 | 100 | 1 | 18.86 | 837.855 | -8 | 6707.8906 | 6710.79 | 6707.8845 | 1320549.62 |
| 4085-4105 | Ar-pUr-pUr-pAr-pAr-pCr-pCr-pUr-pUr-pUr-pAr-pGr-pCr-pAr-pA | Cyclic phosphorylation | 21 | 1.64 | 100 | 1.2 | 18.86 | 744.537 | -9 | 6707.8955 | 6710.86 | 6707.8845 | 991770.44 |
| 3112-3140 | Cr-pCr-pUr-pCr-pUr-pGr-pCr-pCr-pAr-pAr-pUr-pCr-pUr-pGr-pG | Cyclic phosphorylation | 29 | 0.42 | 98.5 | 1.4 | 18.89 | 922.817 | -10 | 9235.2256 | 9239.37 | 9235.2217 | 588947.25 |
| 3112-3140 | Cr-pCr-pUr-pCr-pUr-pGr-pCr-pCr-pAr-pAr-pUr-pCr-pUr-pGr-pG | Cyclic phosphorylation | 29 | 1.27 | 98.5 | 1.1 | 18.89 | 838.924 | -11 | 9235.2334 | 9239.61 | 9235.2217 | 989636 |
| 3112-3140 | Cr-pCr-pUr-pCr-pUr-pGr-pCr-pCr-pAr-pAr-pUr-pCr-pUr-pGr-pG | Cyclic phosphorylation | 29 | 2.43 | 98.5 | 1 | 18.89 | 768.846 | -12 | 9235.2441 | 9239.29 | 9235.2217 | 972853.31 |
| 3794-3822 | Ur-pUr-pGr-pUr-pAr-pGr-pCr-pUr-pGr-pUr-pGr-pGr-pCr-pAr-pG | Cyclic phosphorylation | 29 | 2.33 | 98.5 | 1 | 19.05 | 934.611 | -10 | 9351.1816 | 9354.92 | 9351.1599 | 357105.78 |
| 3794-3822 | Ur-pUr-pGr-pUr-pAr-pGr-pCr-pUr-pGr-pUr-pGr-pGr-pCr-pAr-pG | Cyclic phosphorylation | 29 | 2.96 | 98.5 | 1 | 19.05 | 849.373 | -11 | 9351.1875 | 9355.33 | 9351.1599 | 612464.5 |
| 3794-3822 | Ur-pUr-pGr-pUr-pAr-pGr-pCr-pUr-pGr-pUr-pGr-pGr-pCr-pAr-pG | Cyclic phosphorylation | 29 | 2.02 | 98.5 | 1.1 | 19.06 | 778.592 | -12 | 9351.1787 | 9355.64 | 9351.1599 | 489893.12 |
| 13-40 | Ur-pAr-pUr-pCr-pUr-pCr-pUr-pUr-pGr-pGr-pUr-pCr-pCr-pC | Cyclic phosphorylation | 28 | 1.61 | 100 | 1 | 19.19 | 888.607 | -10 | 8893.1406 | 8896.93 | 8893.1263 | 1342985.38 |
| 13-40 | Ur-pAr-pUr-pCr-pUr-pCr-pUr-pUr-pGr-pGr-pUr-pCr-pCr-pC | Cyclic phosphorylation | 28 | 7.76 | 100 | 1.1 | 19.19 | 987.453 | -9 | 8893.1953 | 8896.94 | 8893.1263 | 597925 |
| 13-40 | Ur-pAr-pUr-pCr-pUr-pCr-pUr-pUr-pGr-pGr-pUr-pCr-pCr-pC | Cyclic phosphorylation | 28 | 2.71 | 100 | 1.1 | 19.21 | 740.339 | -12 | 8893.1504 | 8897.28 | 8893.1263 | 932113 |
| 13-40 | Ur-pAr-pUr-pCr-pUr-pCr-pUr-pUr-pGr-pGr-pUr-pCr-pCr-pC | Cyclic phosphorylation | 28 | 2.71 | 100 | 1 | 19.21 | 807.733 | -11 | 8893.1504 | 8897.22 | 8893.1263 | 1674377.5 |
| 832-863 | Cr-pUr-pGr-pGr-pUr-pGr-pCr-pCr-pGr-pCr-pCr-pGr-pCr-pUr-pU | Cyclic phosphorylation | 32 | 0.95 | 100 | 1 | 19.4 | 787.169 | -13 | 10242.2822 | 10246.91 | 10242.2725 | 1307316.5 |
| 832-863 | Cr-pUr-pGr-pGr-pUr-pGr-pCr-pCr-pGr-pCr-pCr-pGr-pCr-pUr-pU | Cyclic phosphorylation | 32 | -3.72 | 100 | 1 | 19.4 | 852.934 | -12 | 10242.2344 | 10246.84 | 10242.2725 | 1122340.88 |
| 832-863 | Cr-pUr-pGr-pGr-pUr-pGr-pCr-pCr-pGr-pCr-pCr-pGr-pCr-pUr-pU | Cyclic phosphorylation | 32 | 0.76 | 100 | 1.1 | 19.4 | 730.799 | -14 | 10242.2803 | 10246.78 | 10242.2725 | 510289.69 |
| 1219-1245 | Ar-pCr-pCr-pUr-pGr-pUr-pGr-pCr-pUr-pCr-pCr-pAr-pAr-pAr | Cyclic phosphorylation | 27 | -0.46 | 90.9 | 1 | 19.45 | 862.007 | -10 | 8626.1191 | 8630.18 | 8626.1231 | 599268 |
| 1219-1245 | Ar-pCr-pCr-pUr-pGr-pUr-pGr-pCr-pUr-pCr-pCr-pAr-pAr-pAr | Cyclic phosphorylation | 27 | -0.57 | 90.9 | 1.1 | 19.47 | 783.369 | -11 | 8626.1182 | 8629.9 | 8626.1231 | 409931.59 |
| 3516-3540 | Ur-pAr-pCr-pCr-pUr-pUr-pAr-pAr-pGr-pAr-pAr-pCr-pCr-pAr-pCr | Cyclic phosphorylation | 25 | 1.85 | 100 | 1 | 19.52 | 884.56 | -9 | 7966.1025 | 7969.52 | 7966.0878 | 638153.75 |
| 3516-3540 | Ur-pAr-pCr-pCr-pUr-pUr-pAr-pAr-pGr-pAr-pAr-pCr-pCr-pAr-pCr | Cyclic phosphorylation | 25 | 2.1 | 100 | 1 | 19.52 | 795.904 | -10 | 7966.1045 | 7969.58 | 7966.0878 | 690988.38 |
| 1986-2013 | Ar-pCr-pCr-pAr-pGr-pAr-pGr-pCr-pCr-pGr-pGr-pCr-pUr-pGr-pU | Cyclic phosphorylation | 28 | 0.35 | 90.8 | 1.2 | 19.56 | 754.679 | -12 | 9065.2129 | 9068.82 | 9065.2097 | 480614.47 |
| 1986-2013 | Ar-pCr-pCr-pAr-pGr-pAr-pGr-pCr-pCr-pGr-pGr-pCr-pUr-pGr-pU | Cyclic phosphorylation | 28 | 2.18 | 90.8 | 1 | 19.57 | 823.469 | -11 | 9065.2295 | 9069.36 | 9065.2097 | 654287.06 |
| 172-197 | Ar-pCr-pAr-pGr-pGr-pUr-pGr-pUr-pUr-pCr-pAr-pGr-pAr-pU | Cyclic phosphorylation | 26 | 1.7 | 99.1 | 1.1 | 19.59 | 763.003 | -11 | 8401.1084 | 8404.58 | 8401.0941 | 695705.88 |
| 1746-1773 | Ur-pUr-pUr-pGr-pCr-pCr-pCr-pGr-pGr-pAr-pUr-pAr-pUr-pC | Cyclic phosphorylation | 28 | 0.12 | 90.5 | 1 | 19.68 | 818.465 | -11 | 9011.1777 | 9015.45 | 9011.1767 | 343784.56 |
| 3765-3793 | Ar-pCr-pAr-pGr-pCr-pUr-pGr-pCr-pUr-pGr-pUr-pAr-pGr-pC | Cyclic phosphorylation | 29 | 0.59 | 99.2 | 1.1 | 19.74 | 851.287 | -11 | 9372.2246 | 9376.66 | 9372.219 | 316517.88 |
| 3765-3793 | Ar-pCr-pAr-pGr-pCr-pUr-pGr-pCr-pUr-pGr-pUr-pAr-pGr-pC | Cyclic phosphorylation | 29 | 1.74 | 99.2 | 1.2 | 19.74 | 780.43 | -12 | 9372.2354 | 9376.3 | 9372.219 | 316004.06 |
| 2060-2084 | Ar-pAr-pUr-pCr-pUr-pGr-pCr-pGr-pCr-pAr-pGr-pCr-pUr-pAr | Cyclic phosphorylation | 25 | 1.54 | 100 | 1 | 19.8 | 890.451 | -9 | 8021.1172 | 8024.65 | 8021.1048 | 620131.69 |
| 2060-2084 | Ar-pAr-pUr-pCr-pUr-pGr-pCr-pGr-pCr-pAr-pGr-pCr-pUr-pAr | Cyclic phosphorylation | 25 | 3.61 | 100 | 1.3 | 19.8 | 728.458 | -11 | 8021.1338 | 8024.6 | 8021.1048 | 351442.03 |
| 2060-2084 | Ar-pAr-pUr-pCr-pUr-pGr-pCr-pGr-pCr-pAr-pGr-pCr-pUr-pAr | Cyclic phosphorylation | 25 | 1.54 | 100 | 1 | 19.8 | 801.405 | -10 | 8021.1172 | 8024.55 | 8021.1048 | 1159330.88 |

| | | | | | | | | | | | | | |
|---|---|---|---|---|---|---|---|---|---|---|---|---|---|
| 948-975 | Ar-pCr-pAr-pAr-pAr-pGr-pUr-pGr-pCr-pAr-pCr-pCr-pCr-pUr-pGr | Cyclic phosphorylation | 28 | 1.05 | 100 | 1 | 20 | 744.178 | -12 | 8938.2061 | 8942.19 | 8938.1967 | 1242790.25 |
| 948-975 | Ar-pCr-pAr-pAr-pAr-pGr-pUr-pGr-pCr-pAr-pCr-pCr-pCr-pUr-pGr | Cyclic phosphorylation | 28 | 2.25 | 100 | 1 | 20 | 811.922 | -11 | 8938.2168 | 8942.18 | 8938.1967 | 3416594.75 |
| 948-975 | Ar-pCr-pAr-pAr-pAr-pGr-pUr-pGr-pCr-pAr-pCr-pCr-pCr-pUr-pGr | Cyclic phosphorylation | 28 | 1.7 | 100 | 1.2 | 20 | 992.461 | -9 | 8938.2119 | 8942.3 | 8938.1967 | 1038595.31 |
| 948-975 | Ar-pCr-pAr-pAr-pAr-pGr-pUr-pGr-pCr-pAr-pCr-pCr-pCr-pUr-pGr | Cyclic phosphorylation | 28 | 1.59 | 100 | 1.1 | 20 | 893.215 | -10 | 8938.2109 | 8942.35 | 8938.1967 | 2343247.25 |
| 4054-4082 | Cr-pCr-pUr-pAr-pGr-pCr-pCr-pAr-pCr-pAr-pCr-pCr-pCr-pCr-pCr- | Cyclic phosphorylation | 29 | 1.58 | 99.8 | 1 | 20.18 | 774.605 | -12 | 9303.334 | 9307.64 | 9303.3193 | 1177717.5 |
| 4054-4082 | Cr-pCr-pUr-pAr-pGr-pCr-pCr-pAr-pCr-pAr-pCr-pCr-pCr-pCr-pCr- | Cyclic phosphorylation | 29 | -1.68 | 99.8 | 1 | 20.18 | 929.727 | -10 | 9303.3037 | 9307.55 | 9303.3193 | 968231.56 |
| 4054-4082 | Cr-pCr-pUr-pAr-pGr-pCr-pCr-pAr-pCr-pAr-pCr-pCr-pCr-pCr-pCr- | Cyclic phosphorylation | 29 | 1.16 | 99.8 | 1 | 20.18 | 845.115 | -11 | 9303.3301 | 9307.44 | 9303.3193 | 1238005 |
| 4054-4082 | Cr-pCr-pUr-pAr-pGr-pCr-pCr-pAr-pCr-pAr-pCr-pCr-pCr-pCr-pCr- | Cyclic phosphorylation | 29 | 2 | 99.8 | 1.4 | 20.18 | 1032.92 | -9 | 9303.3379 | 9307.31 | 9303.3193 | 466514.81 |
| 2085-2110 | Ar-pCr-pAr-pAr-pAr-pCr-pAr-pGr-pCr-pCr-pCr-pUr-pCr-pGr-pGr | Cyclic phosphorylation | 26 | 0.24 | 99.3 | 1 | 20.47 | 844.516 | -10 | 8452.2148 | 8456.68 | 8452.2128 | 1057073.5 |
| 2085-2110 | Ar-pCr-pAr-pAr-pAr-pCr-pAr-pGr-pCr-pCr-pCr-pUr-pCr-pGr-pGr | Cyclic phosphorylation | 26 | -2.88 | 99.3 | 1.5 | 20.47 | 767.65 | -11 | 8452.1885 | 8455.88 | 8452.2128 | 609018 |
| 4007-4036 | Ur-pUr-pCr-pCr-pAr-pGr-pAr-pCr-pAr-pCr-pCr-pUr-pCr-pCr-pCr | Cyclic phosphorylation | 30 | -2.13 | 92.6 | 1.2 | 20.52 | 735.557 | -13 | 9571.2861 | 9575.37 | 9571.3065 | 1197080.62 |
| 3110-3140 | Ar-pGr-pCr-pCr-pUr-pCr-pUr-pGr-pCr-pCr-pAr-pAr-pUr-pCr-pUr | Cyclic phosphorylation | 31 | 1.05 | 100 | 1 | 20.56 | 825.105 | -12 | 9909.332 | 9913.98 | 9909.3216 | 846673.81 |
| 3110-3140 | Ar-pGr-pCr-pCr-pUr-pCr-pUr-pGr-pCr-pCr-pAr-pAr-pUr-pCr-pUr | Cyclic phosphorylation | 31 | 1.25 | 100 | 1 | 20.57 | 900.297 | -11 | 9909.334 | 9914.02 | 9909.3216 | 950525.44 |
| 3145-3171 | Ar-pGr-pUr-pGr-pUr-pGr-pUr-pGr-pCr-pUr-pGr-pGr-pGr-pCr-p( | Cyclic phosphorylation | 27 | 1.31 | 100 | 1 | 20.66 | 888.216 | -10 | 8888.2148 | 8892.32 | 8888.2032 | 1094432.88 |
| 3145-3171 | Ar-pGr-pUr-pGr-pUr-pGr-pUr-pGr-pCr-pUr-pGr-pGr-pGr-pCr-p( | Cyclic phosphorylation | 27 | 7.46 | 100 | 1.2 | 20.66 | 807.286 | -11 | 8888.2695 | 8891.93 | 8888.2032 | 569573.38 |
| 3145-3171 | Ar-pGr-pUr-pGr-pUr-pGr-pUr-pGr-pCr-pUr-pGr-pGr-pGr-pCr-p( | Cyclic phosphorylation | 27 | 1.86 | 100 | 1 | 20.66 | 986.906 | -9 | 8888.2197 | 8892.11 | 8888.2032 | 627611.06 |
| 3849-3874 | Ar-pAr-pGr-pGr-pGr-pCr-pGr-pUr-pGr-pAr-pAr-pAr-pCr-pUr-pG | Cyclic phosphorylation | 26 | -0.97 | 100 | 1 | 20.88 | 936.123 | -9 | 8431.1455 | 8435.1 | 8431.1536 | 497984.34 |
| 3849-3874 | Ar-pAr-pGr-pGr-pGr-pCr-pGr-pUr-pGr-pAr-pAr-pAr-pCr-pUr-pG | Cyclic phosphorylation | 26 | 1.12 | 100 | 1 | 20.89 | 842.41 | -10 | 8431.1631 | 8434.65 | 8431.1536 | 1135533.12 |
| 3849-3874 | Ar-pAr-pGr-pGr-pGr-pCr-pGr-pUr-pGr-pAr-pAr-pAr-pCr-pUr-pG | Cyclic phosphorylation | 26 | 1.7 | 100 | 1.1 | 20.9 | 765.736 | -11 | 8431.168 | 8435.1 | 8431.1536 | 887861.56 |
| 3849-3874 | Ar-pAr-pGr-pGr-pGr-pCr-pGr-pUr-pGr-pAr-pAr-pAr-pCr-pUr-pG | Cyclic phosphorylation | 26 | 3.09 | 100 | 1.4 | 20.92 | 701.841 | -12 | 8431.1797 | 8434.92 | 8431.1536 | 771868.19 |
| 4054-4084 | Cr-pCr-pUr-pAr-pGr-pCr-pCr-pAr-pCr-pAr-pCr-pCr-pCr-pCr-pCr- | Cyclic phosphorylation | 31 | -3.97 | 100 | 1.2 | 20.99 | 765.026 | -13 | 9954.3525 | 9958.84 | 9954.392 | 670949.75 |
| 4054-4084 | Cr-pCr-pUr-pAr-pGr-pCr-pCr-pAr-pCr-pAr-pCr-pCr-pCr-pCr-pCr- | Cyclic phosphorylation | 31 | 1.23 | 100 | 1 | 21.01 | 828.861 | -12 | 9954.4043 | 9958.87 | 9954.392 | 1313129 |
| 4054-4084 | Cr-pCr-pUr-pAr-pGr-pCr-pCr-pAr-pCr-pAr-pCr-pCr-pCr-pCr-pCr- | Cyclic phosphorylation | 31 | 1.62 | 100 | 1 | 21.01 | 904.304 | -11 | 9954.4082 | 9958.97 | 9954.392 | 1011630.81 |
| 4097-4119 | Cr-pAr-pAr-pUr-pAr-pAr-pAr-pCr-pGr-pAr-pAr-pAr-pGr-pUr-pU | Cyclic phosphorylation | 23 | 2.31 | 98.5 | 1 | 21.02 | 824.776 | -9 | 7429.04 | 7432.3 | 7429.0229 | 1513554.25 |
| 4097-4119 | Cr-pAr-pAr-pUr-pAr-pAr-pAr-pCr-pGr-pAr-pAr-pAr-pGr-pUr-pU | Cyclic phosphorylation | 23 | 2.7 | 98.5 | 1 | 21.04 | 927.998 | -8 | 7429.043 | 7432.4 | 7429.0229 | 1082707.5 |
| 4097-4119 | Cr-pAr-pAr-pUr-pAr-pAr-pAr-pCr-pGr-pAr-pAr-pAr-pGr-pUr-pU | Cyclic phosphorylation | 23 | 3.29 | 98.5 | 1.2 | 21.04 | 742.097 | -10 | 7429.0474 | 7432.08 | 7429.0229 | 619275.88 |
| 3268-3298 | Ar-pGr-pAr-pAr-pGr-pAr-pAr-pUr-pUr-pUr-pCr-pAr-pCr-pCr-pA | Cyclic phosphorylation | 31 | -3.2 | 99.7 | 1.1 | 21.32 | 822.605 | -12 | 9878.2842 | 9882.58 | 9878.3158 | 570859.81 |
| 3268-3298 | Ar-pGr-pAr-pAr-pGr-pAr-pAr-pUr-pUr-pUr-pCr-pAr-pCr-pCr-pA | Cyclic phosphorylation | 31 | 3.12 | 99.7 | 1 | 21.32 | 897.388 | -11 | 9878.3467 | 9882.6 | 9878.3158 | 710970.38 |
| 3141-3171 | Ur-pCr-pUr-pGr-pAr-pGr-pUr-pGr-pUr-pGr-pUr-pGr-pCr-pUr-p( | Cyclic phosphorylation | 31 | 0.89 | 100 | 1 | 21.47 | 845.19 | -12 | 10150.3516 | 10154.81 | 10150.3425 | 3037383.5 |
| 3141-3171 | Ur-pCr-pUr-pGr-pAr-pGr-pUr-pGr-pUr-pGr-pUr-pGr-pCr-pUr-p( | Cyclic phosphorylation | 31 | 1.18 | 100 | 1 | 21.49 | 922.208 | -11 | 10150.3545 | 10154.98 | 10150.3425 | 2211866.25 |
| 3453-3483 | Ar-pAr-pCr-pAr-pAr-pUr-pAr-pCr-pCr-pGr-pUr-pGr-pUr-pAr-pCr | Cyclic phosphorylation | 31 | 3.7 | 99.8 | 1 | 21.8 | 760.251 | -13 | 9893.3633 | 9897.78 | 9893.3267 | 1464897.25 |
| 3453-3483 | Ar-pAr-pCr-pAr-pAr-pUr-pAr-pCr-pCr-pGr-pUr-pGr-pUr-pAr-pCr | Cyclic phosphorylation | 31 | 1.43 | 99.8 | 1 | 21.82 | 823.772 | -12 | 9893.3408 | 9897.64 | 9893.3267 | 4831192.5 |
| 3453-3483 | Ar-pAr-pCr-pAr-pAr-pUr-pAr-pCr-pCr-pGr-pUr-pGr-pUr-pAr-pCr | Cyclic phosphorylation | 31 | 1.82 | 99.8 | 1.1 | 21.83 | 1098.587 | -9 | 9893.3447 | 9897.53 | 9893.3267 | 904745.06 |
| 3453-3483 | Ar-pAr-pCr-pAr-pAr-pUr-pAr-pCr-pCr-pGr-pUr-pGr-pUr-pAr-pCr | Cyclic phosphorylation | 31 | 1.13 | 99.8 | 1 | 21.83 | 898.752 | -11 | 9893.3379 | 9897.93 | 9893.3267 | 4476176.5 |
| 3453-3483 | Ar-pAr-pCr-pAr-pAr-pUr-pAr-pCr-pCr-pGr-pUr-pGr-pUr-pAr-pCr | Cyclic phosphorylation | 31 | 1.52 | 99.8 | 1 | 21.83 | 988.828 | -10 | 9893.3418 | 9897.92 | 9893.3267 | 2471035.25 |
| 3453-3483 | Ar-pAr-pCr-pAr-pAr-pUr-pAr-pCr-pCr-pGr-pUr-pGr-pUr-pAr-pCr | Cyclic phosphorylation | 31 | 2.91 | 98.7 | 1 | 21.92 | 898.843 | -11 | 9893.3555 | 9897.56 | 9893.3267 | 527814.19 |
| 3758-3793 | Ur-pUr-pGr-pCr-pAr-pUr-pGr-pAr-pCr-pCr-pAr-pGr-pCr-pUr-pG | Cyclic phosphorylation | 36 | 2 | 96.9 | 1 | 21.95 | 828.958 | -14 | 11614.5068 | 11619.81 | 11614.4836 | 2205377.75 |
| 3758-3793 | Ur-pUr-pGr-pCr-pAr-pUr-pGr-pAr-pCr-pCr-pAr-pGr-pCr-pUr-pG | Cyclic phosphorylation | 36 | 3.76 | 96.9 | 1 | 21.95 | 773.56 | -15 | 11614.5273 | 11619.7 | 11614.4836 | 2410512 |
| 2160-2190 | Ar-pAr-pCr-pAr-pGr-pCr-pGr-pUr-pGr-pGr-pCr-pCr-pUr-pAr-pCr | Cyclic phosphorylation | 31 | 2.14 | 100 | 1.3 | 22.01 | 759.25 | -13 | 9878.3369 | 9883.27 | 9878.3158 | 650686.44 |
| 2160-2190 | Ar-pAr-pCr-pAr-pGr-pCr-pGr-pUr-pGr-pGr-pCr-pCr-pUr-pAr-pCr | Cyclic phosphorylation | 31 | -1.82 | 100 | 1 | 22.02 | 822.521 | -12 | 9878.2979 | 9882.85 | 9878.3158 | 3208816.5 |
| 2160-2190 | Ar-pAr-pCr-pAr-pGr-pCr-pGr-pUr-pGr-pGr-pCr-pCr-pUr-pAr-pCr | Cyclic phosphorylation | 31 | 2.53 | 100 | 1 | 22.02 | 897.388 | -11 | 9878.3408 | 9883.09 | 9878.3158 | 2914412.5 |
| 2160-2190 | Ar-pAr-pCr-pAr-pGr-pCr-pGr-pUr-pGr-pGr-pCr-pCr-pUr-pAr-pCr | Cyclic phosphorylation | 31 | 1.05 | 100 | 1.1 | 22.04 | 987.227 | -10 | 9878.3262 | 9883.09 | 9878.3158 | 1454433.62 |
| 2160-2190 | Ar-pAr-pCr-pAr-pGr-pCr-pGr-pUr-pGr-pGr-pCr-pCr-pUr-pAr-pCr | Cyclic phosphorylation | 31 | 0.26 | 100 | 1.3 | 22.04 | 1097.143 | -9 | 9878.3184 | 9882.77 | 9878.3158 | 489169.03 |
| 1667-1701 | Cr-pGr-pUr-pGr-pAr-pAr-pCr-pUr-pUr-pCr-pAr-pAr-pCr-pUr-pU | Cyclic phosphorylation | 35 | 4.55 | 94.2 | 1.2 | 22.07 | 798.887 | -14 | 11194.5391 | 11199.59 | 11194.4882 | 548155.94 |
| 1667-1701 | Cr-pGr-pUr-pGr-pAr-pAr-pCr-pUr-pUr-pCr-pAr-pAr-pCr-pUr-pU | Cyclic phosphorylation | 35 | 3.5 | 94.2 | 1 | 22.09 | 932.452 | -12 | 11194.5273 | 11198.57 | 11194.4882 | 386862.44 |
| 3141-3173 | Ur-pCr-pUr-pGr-pAr-pGr-pUr-pGr-pUr-pGr-pUr-pGr-pCr-pUr-p( | Cyclic phosphorylation | 33 | 3.05 | 99.9 | 1.1 | 22.25 | 770.882 | -14 | 10801.4482 | 10806.2 | 10801.4153 | 1206591.25 |
| 3141-3173 | Ur-pCr-pUr-pGr-pAr-pGr-pUr-pGr-pUr-pGr-pUr-pGr-pCr-pUr-p( | Cyclic phosphorylation | 33 | 1.06 | 99.9 | 1 | 22.27 | 830.258 | -13 | 10801.4268 | 10806.15 | 10801.4153 | 2002029.25 |
| 3141-3173 | Ur-pCr-pUr-pGr-pAr-pGr-pUr-pGr-pUr-pGr-pUr-pGr-pCr-pUr-p( | Cyclic phosphorylation | 33 | 4.23 | 99.9 | 1 | 22.27 | 899.53 | -12 | 10801.4609 | 10806.12 | 10801.4153 | 1629548.12 |
| 3141-3173 | Ur-pCr-pUr-pGr-pAr-pGr-pUr-pGr-pUr-pGr-pUr-pGr-pCr-pUr-p( | Cyclic phosphorylation | 33 | 1.24 | 99.9 | 1 | 22.28 | 981.306 | -11 | 10801.4287 | 10806.07 | 10801.4153 | 963149.88 |
| 414-441 | Ar-pAr-pCr-pAr-pAr-pCr-pGr-pCr-pCr-pAr-pCr-pCr-pAr-pAr-pCr- | Cyclic phosphorylation | 28 | -0.51 | 100 | 1 | 22.42 | 1000.247 | -9 | 9008.2578 | 9012.3 | 9008.2624 | 882802.69 |
| 414-441 | Ar-pAr-pCr-pAr-pAr-pCr-pGr-pCr-pCr-pAr-pCr-pCr-pAr-pAr-pCr- | Cyclic phosphorylation | 28 | 3.18 | 100 | 1 | 22.42 | 900.121 | -10 | 9008.291 | 9012.34 | 9008.2624 | 1557430.88 |
| 414-441 | Ar-pAr-pCr-pAr-pAr-pCr-pGr-pCr-pCr-pAr-pCr-pCr-pAr-pAr-pCr- | Cyclic phosphorylation | 28 | -0.72 | 100 | 1 | 22.42 | 818.292 | -11 | 9008.2559 | 9012.37 | 9008.2624 | 1310231.62 |
| 882-912 | Cr-pUr-pGr-pAr-pAr-pGr-pUr-pAr-pCr-pAr-pAr-pCr-pGr-pAr-pG | Cyclic phosphorylation | 31 | 1.36 | 99.2 | 1.1 | 22.89 | 908.759 | -11 | 10003.4121 | 10007.7 | 10003.3985 | 870716.19 |
| 882-912 | Cr-pUr-pGr-pAr-pAr-pGr-pUr-pAr-pCr-pAr-pAr-pCr-pGr-pAr-pG | Cyclic phosphorylation | 31 | -1.77 | 99.2 | 1.1 | 22.89 | 768.872 | -13 | 10003.3809 | 10007.85 | 10003.3985 | 357618 |
| 882-912 | Cr-pUr-pGr-pAr-pAr-pGr-pUr-pAr-pCr-pAr-pAr-pCr-pGr-pAr-pG | Cyclic phosphorylation | 31 | -2.16 | 99.2 | 1 | 22.89 | 833.029 | -12 | 10003.377 | 10008.02 | 10003.3985 | 1117226.88 |
| 3511-3540 | Ar-pCr-pAr-pAr-pGr-pUr-pAr-pCr-pUr-pUr-pUr-pAr-pAr-pGr-pA | Cyclic phosphorylation | 30 | -9.77 | 94.5 | 1 | 23.42 | 799.523 | -12 | 9603.2402 | 9607.42 | 9603.334 | 551349.06 |
| 3511-3540 | Ar-pCr-pAr-pAr-pGr-pUr-pAr-pCr-pUr-pUr-pUr-pAr-pAr-pGr-pA | Cyclic phosphorylation | 30 | 3.15 | 94.5 | 1 | 23.44 | 872.39 | -11 | 9603.3643 | 9607.54 | 9603.334 | 767286.88 |
| 1559-1599 | Cr-pGr-pUr-pGr-pGr-pGr-pCr-pUr-pAr-pUr-pCr-pAr-pGr-pCr-pC | Cyclic phosphorylation | 41 | 7.21 | 100 | 1 | 23.63 | 883.375 | -15 | 13260.7881 | 13266.28 | 13260.6925 | 1068277.25 |
| 1559-1599 | Cr-pGr-pUr-pGr-pGr-pGr-pCr-pUr-pAr-pUr-pCr-pAr-pGr-pCr-pC | Cyclic phosphorylation | 41 | 2.86 | 100 | 1 | 23.63 | 946.545 | -14 | 13260.7305 | 13266.58 | 13260.6925 | 777318.56 |

| Positions | Oligo Sequence | 3' Termini | Seq Length | Δ ppm | Conf. Score | Best ASR | RT | M/Z | Charge St. | Mono Mass Exp. | Avg Mass Exp. | Mono Mass Theo. | MS Area |
|---|---|---|---|---|---|---|---|---|---|---|---|---|---|
| 3878-3882 | Ar-pCr-pUr-pCr-pGr | Cyclic phosphorylation | 5 | 0.26 | 98.3 | 1.7 | 3.82 | 794.097 | -2 | 1590.2083 | 1590.78 | 1590.2078 | 1404503.38 |
| 2808-2813 | Ar-pAr-pCr-pUr-pCr-pAr-pGr | Phosphorylation | 6 | 1.17 | 97.2 | 1 | 4 | 652.426 | -3 | 1960.3004 | 1961.17 | 1960.2981 | 1281875.88 |
| 3375-3384 | Ar-pAr-pCr-pUr-pUr-pCr-pUr-pAr-pCr-pGr | Cyclic phosphorylation | 10 | 1.4 | 92.3 | 1 | 4.17 | 790.596 | -4 | 3165.4092 | 3166.73 | 3165.4048 | 2375707.5 |
| 3585-3593 | Ar-pAr-pAr-pAr-pUr-pCr-pCr-pAr-pGr | Phosphorylation | 9 | 1.18 | 97 | 1 | 4.24 | 724.348 | -4 | 2900.4207 | 2901.63 | 2900.4172 | 2098805 |
| 3864-3874 | Cr-pAr-pCr-pUr-pAr-pCr-pAr-pCr-pAr-pUr-pGr | Phosphorylation | 11 | 0.92 | 100 | 1 | 4.48 | 877.113 | -4 | 3511.4871 | 3513.21 | 3511.4838 | 1597736.5 |
| 3864-3874 | Cr-pAr-pCr-pUr-pAr-pCr-pAr-pCr-pAr-pUr-pGr | Phosphorylation | 11 | 1.13 | 100 | 1 | 4.5 | 701.689 | -5 | 3511.4878 | 3513.57 | 3511.4838 | 1043124.06 |
| 1941-1951 | Ar-pCr-pAr-pCr-pCr-pUr-pAr-pCr-pAr-pUr-pGr | Cyclic phosphorylation | 11 | 1.5 | 100 | 1 | 4.71 | 581.406 | -6 | 3493.4785 | 3495.02 | 3493.4733 | 1833832 |
| 1941-1951 | Ar-pCr-pAr-pCr-pCr-pUr-pAr-pCr-pAr-pUr-pGr | Cyclic phosphorylation | 11 | 0.8 | 100 | 1 | 4.71 | 1163.819 | -3 | 3493.4761 | 3494.95 | 3493.4733 | 1249237.38 |
| 1941-1951 | Ar-pCr-pAr-pCr-pCr-pUr-pAr-pCr-pAr-pUr-pGr | Cyclic phosphorylation | 11 | 0.45 | 100 | 1 | 4.72 | 872.612 | -4 | 3493.4749 | 3494.97 | 3493.4733 | 1918356.62 |
| 1941-1951 | Ar-pCr-pAr-pCr-pCr-pUr-pAr-pCr-pAr-pUr-pGr | Cyclic phosphorylation | 11 | 0.66 | 100 | 1 | 4.74 | 697.888 | -5 | 3493.4756 | 3494.91 | 3493.4733 | 3368986.75 |
| 2450-2461 | Cr-pUr-pUr-pCr-pAr-pAr-pUr-pUr-pUr-pCr-pAr-pGr | Phosphorylation | 12 | 0.62 | 100 | 1.2 | 4.75 | 758.287 | -5 | 3795.4683 | 3796.97 | 3795.4659 | 1393378.75 |
| 2382-2391 | Ar-pAr-pCr-pAr-pCr-pCr-pCr-pAr-pAr-pGr | Cyclic phosphorylation | 10 | 0.73 | 100 | 1 | 5.07 | 801.863 | -4 | 3210.4775 | 3211.86 | 3210.4752 | 1030230.62 |
| 2382-2391 | Ar-pAr-pCr-pAr-pCr-pCr-pCr-pAr-pAr-pGr | Cyclic phosphorylation | 10 | 0.96 | 100 | 1 | 5.1 | 641.289 | -5 | 3210.4783 | 3211.71 | 3210.4752 | 1500120.38 |
| 3406-3417 | Ar-pCr-pAr-pAr-pCr-pAr-pCr-pCr-pUr-pUr-pCr-pGr | Cyclic phosphorylation | 12 | 1.83 | 100 | 1 | 5.24 | 632.246 | -6 | 3798.5215 | 3799.99 | 3798.5146 | 7389065 |
| 3406-3417 | Ar-pCr-pAr-pAr-pCr-pAr-pCr-pCr-pUr-pUr-pCr-pGr | Cyclic phosphorylation | 12 | 1.7 | 100 | 1.2 | 5.24 | 948.873 | -4 | 3798.521 | 3799.97 | 3798.5146 | 3035178.5 |
| 3406-3417 | Ar-pCr-pAr-pAr-pCr-pAr-pCr-pCr-pUr-pUr-pCr-pGr | Cyclic phosphorylation | 12 | 1.44 | 100 | 1.2 | 5.26 | 758.896 | -5 | 3798.52 | 3799.99 | 3798.5146 | 6944982 |
| 495-503 | Ar-pAr-pCr-pAr-pAr-pCr-pAr-pAr-pGr | Cyclic phosphorylation | 9 | 0.56 | 99.1 | 1 | 5.34 | 975.809 | -3 | 2929.4468 | 2930.6 | 2929.4451 | 1364777.75 |
| 495-503 | Ar-pAr-pCr-pAr-pAr-pCr-pAr-pAr-pGr | Cyclic phosphorylation | 9 | 0.31 | 99.1 | 1 | 5.34 | 731.605 | -4 | 2929.446 | 2930.59 | 2929.4451 | 3310373.5 |
| 495-503 | Ar-pAr-pCr-pAr-pAr-pCr-pAr-pAr-pGr | Cyclic phosphorylation | 9 | 0.98 | 99.1 | 1 | 5.36 | 585.083 | -5 | 2929.448 | 2930.59 | 2929.4451 | 1961912 |
| 1810-1822 | Ar-pCr-pAr-pUr-pCr-pAr-pCr-pCr-pCr-pCr-pUr-pUr-pGr | Cyclic phosphorylation | 13 | 1 | 100 | 1.1 | 5.46 | 582.069 | -7 | 4080.5327 | 4082.19 | 4080.5286 | 2744711.25 |
| 1810-1822 | Ar-pCr-pAr-pUr-pCr-pAr-pCr-pCr-pCr-pCr-pUr-pUr-pGr | Cyclic phosphorylation | 13 | 0.05 | 100 | 1.1 | 5.46 | 679.247 | -6 | 4080.5288 | 4082.21 | 4080.5286 | 7842275 |
| 1810-1822 | Ar-pCr-pAr-pUr-pCr-pAr-pCr-pCr-pCr-pCr-pUr-pUr-pGr | Cyclic phosphorylation | 13 | 0.7 | 100 | 1 | 5.46 | 1019.376 | -4 | 4080.5315 | 4082.2 | 4080.5286 | 1539908.88 |
| 1810-1822 | Ar-pCr-pAr-pUr-pCr-pAr-pCr-pCr-pCr-pCr-pUr-pUr-pGr | Cyclic phosphorylation | 13 | 0.52 | 100 | 1.2 | 5.46 | 1359.504 | -3 | 4080.5308 | 4082.18 | 4080.5286 | 1419623 |
| 1810-1822 | Ar-pCr-pAr-pUr-pCr-pAr-pCr-pCr-pCr-pCr-pUr-pUr-pGr | Cyclic phosphorylation | 13 | 0.17 | 100 | 1.2 | 5.46 | 815.299 | -5 | 4080.5293 | 4082.18 | 4080.5286 | 3868782.25 |
| 1955-1968 | Gr-pGr-pUr-pGr-pUr-pAr-pCr-pUr-pCr-pCr-pAr-pCr-pCr-pGr | Cyclic phosphorylation | 14 | 1.1 | 100 | 1 | 5.52 | 746.09 | -6 | 4481.582 | 4483.54 | 4481.5771 | 2590676.25 |
| 1955-1968 | Gr-pGr-pUr-pGr-pUr-pAr-pCr-pUr-pCr-pCr-pAr-pCr-pCr-pGr | Cyclic phosphorylation | 14 | 0.99 | 100 | 1.6 | 5.53 | 895.71 | -5 | 4481.5815 | 4483.54 | 4481.5771 | 1300751 |
| 1856-1867 | Cr-pAr-pCr-pCr-pAr-pAr-pCr-pAr-pCr-pCr-pAr-pGr | Cyclic phosphorylation | 12 | 0.86 | 100 | 1 | 5.58 | 635.92 | -6 | 3820.561 | 3822.07 | 3820.5578 | 1463645.62 |
| 4013-4024 | Ar-pCr-pAr-pCr-pCr-pUr-pCr-pCr-pCr-pAr-pAr-pGr | Phosphorylation | 12 | 0.36 | 100 | 1 | 5.58 | 762.301 | -5 | 3815.5425 | 3816.61 | 3815.5411 | 3705103.75 |
| 1856-1867 | Cr-pAr-pCr-pCr-pAr-pAr-pCr-pAr-pCr-pCr-pAr-pGr | Cyclic phosphorylation | 12 | 0.48 | 100 | 1.1 | 5.58 | 954.383 | -4 | 3820.5596 | 3822.05 | 3820.5578 | 1166246.38 |
| 1856-1867 | Cr-pAr-pCr-pCr-pAr-pAr-pCr-pAr-pCr-pCr-pAr-pGr | Cyclic phosphorylation | 12 | 0.09 | 100 | 1 | 5.6 | 763.305 | -5 | 3820.5581 | 3822.07 | 3820.5578 | 2761299.75 |
| 4013-4024 | Ar-pCr-pAr-pCr-pCr-pUr-pCr-pCr-pCr-pAr-pAr-pGr | Phosphorylation | 12 | 0.23 | 100 | 1 | 5.62 | 953.129 | -4 | 3815.542 | 3816.92 | 3815.5411 | 2236445.75 |
| 3453-3462 | Ar-pAr-pCr-pAr-pAr-pUr-pAr-pCr-pCr-pGr | Cyclic phosphorylation | 10 | -0.07 | 100 | 1.1 | 5.67 | 802.108 | -4 | 3211.459 | 3212.83 | 3211.4592 | 5858675.5 |
| 495-503 | Ar-pAr-pCr-pAr-pAr-pCr-pAr-pAr-pGr | Phosphorylation | 9 | 0.29 | 99.9 | 1 | 5.68 | 981.812 | -3 | 2947.4565 | 2948.64 | 2947.4557 | 1494073 |
| 495-503 | Ar-pAr-pCr-pAr-pAr-pCr-pAr-pAr-pGr | Phosphorylation | 9 | 0.29 | 99.9 | 1 | 5.69 | 736.107 | -4 | 2947.4565 | 2948.62 | 2947.4557 | 3923323.5 |
| 3453-3462 | Ar-pAr-pCr-pAr-pAr-pUr-pAr-pCr-pCr-pGr | Cyclic phosphorylation | 10 | 0.54 | 100 | 1 | 5.69 | 1069.814 | -3 | 3211.4609 | 3212.84 | 3211.4592 | 2777685 |
| 3453-3462 | Ar-pAr-pCr-pAr-pAr-pUr-pAr-pCr-pCr-pGr | Cyclic phosphorylation | 10 | -0.22 | 100 | 1 | 5.72 | 641.485 | -5 | 3211.4585 | 3212.69 | 3211.4592 | 9116100 |
| 3453-3462 | Ar-pAr-pCr-pAr-pAr-pUr-pAr-pCr-pCr-pGr | Cyclic phosphorylation | 10 | 0.77 | 100 | 1.2 | 5.72 | 1605.225 | -2 | 3211.4617 | 3212.84 | 3211.4592 | 714297.56 |
| 495-503 | Ar-pAr-pCr-pAr-pAr-pCr-pAr-pAr-pGr | Phosphorylation | 9 | 1.12 | 99.9 | 1 | 5.74 | 588.485 | -5 | 2947.459 | 2948.83 | 2947.4557 | 1016315.31 |
| 3453-3462 | Ar-pAr-pCr-pAr-pAr-pUr-pAr-pCr-pCr-pGr | Cyclic phosphorylation | 10 | 0.62 | 100 | 1.1 | 5.74 | 534.403 | -6 | 3211.4612 | 3212.82 | 3211.4592 | 1012310.12 |
| 3406-3417 | Ar-pCr-pAr-pAr-pCr-pAr-pCr-pCr-pUr-pUr-pCr-pGr | Phosphorylation | 12 | 0.9 | 100 | 1 | 6.05 | 762.499 | -5 | 3816.5286 | 3818.27 | 3816.5251 | 1756506.38 |
| 3406-3417 | Ar-pCr-pAr-pAr-pCr-pAr-pCr-pCr-pUr-pUr-pCr-pGr | Phosphorylation | 12 | 1.54 | 100 | 1 | 6.08 | 635.248 | -6 | 3816.531 | 3818.24 | 3816.5251 | 966356.69 |
| 2705-2718 | Cr-pGr-pGr-pCr-pUr-pGr-pGr-pAr-pCr-pAr-pUr-pUr-pUr-pGr | Cyclic phosphorylation | 14 | 1.32 | 100 | 1.1 | 6.1 | 753.089 | -6 | 4522.5732 | 4524.5 | 4522.5673 | 1219134.88 |
| 3216-3231 | Ur-pCr-pUr-pGr-pCr-pCr-pCr-pCr-pUr-pCr-pAr-pCr-pGr-pGr-pCr | Cyclic phosphorylation | 16 | 0.96 | 100 | 1.2 | 6.25 | 843.936 | -6 | 5067.6533 | 5069.76 | 5067.6485 | 1640404.25 |
| 3216-3231 | Ur-pCr-pUr-pGr-pCr-pCr-pCr-pCr-pUr-pCr-pAr-pCr-pGr-pGr-pCr | Cyclic phosphorylation | 16 | 1.35 | 100 | 1 | 6.26 | 723.229 | -7 | 5067.6553 | 5069.81 | 5067.6485 | 2971755.75 |
| 3216-3231 | Ur-pCr-pUr-pGr-pCr-pCr-pCr-pCr-pUr-pCr-pAr-pCr-pGr-pGr-pCr | Cyclic phosphorylation | 16 | 1.06 | 100 | 1 | 6.26 | 632.7 | -8 | 5067.6538 | 5069.81 | 5067.6485 | 1585948.25 |
| 3969-3984 | Cr-pUr-pCr-pCr-pCr-pAr-pCr-pCr-pUr-pCr-pCr-pAr-pCr-pCr-pUr- | Cyclic phosphorylation | 16 | 1.05 | 100 | 1.1 | 6.36 | 620.574 | -8 | 4971.6465 | 4973.73 | 4971.6412 | 4520100.5 |
| 3969-3984 | Cr-pUr-pCr-pCr-pCr-pAr-pCr-pCr-pUr-pCr-pCr-pAr-pCr-pCr-pUr- | Cyclic phosphorylation | 16 | 0.56 | 100 | 1 | 6.39 | 709.514 | -7 | 4971.644 | 4973.77 | 4971.6412 | 7024183 |
| 3969-3984 | Cr-pUr-pCr-pCr-pCr-pAr-pCr-pCr-pUr-pCr-pCr-pAr-pCr-pCr-pUr- | Cyclic phosphorylation | 16 | 0.37 | 100 | 1.1 | 6.39 | 827.934 | -6 | 4971.6431 | 4973.78 | 4971.6412 | 4275658 |
| 133-145 | Cr-pCr-pUr-pAr-pCr-pAr-pCr-pCr-pAr-pAr-pCr-pAr-pGr | Cyclic phosphorylation | 13 | 0.7 | 100 | 1.1 | 6.63 | 1030.89 | -4 | 4126.5859 | 4128.3 | 4126.5831 | 1068430.88 |
| 133-145 | Cr-pCr-pUr-pAr-pCr-pAr-pCr-pCr-pAr-pAr-pCr-pAr-pGr | Cyclic phosphorylation | 13 | 0.7 | 100 | 1.2 | 6.63 | 824.51 | -5 | 4126.5859 | 4128.41 | 4126.5831 | 3302789.25 |
| 133-145 | Cr-pCr-pUr-pAr-pCr-pAr-pCr-pCr-pAr-pAr-pCr-pAr-pGr | Cyclic phosphorylation | 13 | 1.29 | 100 | 1.1 | 6.65 | 588.792 | -7 | 4126.5884 | 4128.35 | 4126.5831 | 1594213.75 |
| 133-145 | Cr-pCr-pUr-pAr-pCr-pAr-pCr-pCr-pAr-pAr-pCr-pAr-pGr | Cyclic phosphorylation | 13 | 0.23 | 100 | 1 | 6.65 | 686.923 | -6 | 4126.584 | 4128.3 | 4126.5831 | 5588630 |
| 1377-1389 | Gr-pAr-pCr-pUr-pCr-pCr-pAr-pAr-pAr-pGr-pUr-pCr-pGr | Cyclic phosphorylation | 13 | 1.57 | 99.1 | 1 | 6.72 | 696.422 | -6 | 4183.5747 | 4185.42 | 4183.5681 | 1289137.75 |
| 1918-1932 | Ur-pGr-pGr-pCr-pCr-pAr-pUr-pUr-pCr-pAr-pCr-pGr-pCr-pCr-pG | Cyclic phosphorylation | 15 | 0.97 | 100 | 1.2 | 6.74 | 956.518 | -5 | 4786.623 | 4788.71 | 4786.6184 | 841499.88 |
| 1377-1389 | Gr-pAr-pCr-pUr-pCr-pCr-pAr-pAr-pAr-pGr-pUr-pCr-pGr | Cyclic phosphorylation | 13 | 1.57 | 99.1 | 1.2 | 6.75 | 596.79 | -7 | 4183.5747 | 4185.2 | 4183.5681 | 156643.52 |
| 1918-1932 | Ur-pGr-pGr-pCr-pCr-pAr-pUr-pUr-pCr-pAr-pCr-pGr-pCr-pCr-pG | Cyclic phosphorylation | 15 | 0.77 | 100 | 1.5 | 6.75 | 597.571 | -8 | 4786.6221 | 4788.79 | 4786.6184 | 735399.25 |
| 1918-1932 | Ur-pGr-pGr-pCr-pCr-pAr-pUr-pUr-pCr-pAr-pCr-pGr-pCr-pCr-pG | Cyclic phosphorylation | 15 | 1.07 | 100 | 1.1 | 6.75 | 797.097 | -6 | 4786.6235 | 4788.67 | 4786.6184 | 2506075 |
| 1918-1932 | Ur-pGr-pGr-pCr-pCr-pAr-pUr-pUr-pCr-pAr-pCr-pGr-pCr-pCr-pG | Cyclic phosphorylation | 15 | 1.17 | 100 | 1 | 6.76 | 682.939 | -7 | 4786.624 | 4788.67 | 4786.6184 | 4350336.5 |
| 3147-3161 | Ur-pGr-pUr-pGr-pUr-pGr-pCr-pUr-pGr-pGr-pGr-pCr-pCr-pAr-pG | Cyclic phosphorylation | 15 | 1.35 | 100 | 1.1 | 6.8 | 813.263 | -6 | 4883.6162 | 4885.7 | 4883.6096 | 1042962.44 |
| 3147-3161 | Ur-pGr-pUr-pGr-pUr-pGr-pCr-pUr-pGr-pGr-pGr-pCr-pCr-pAr-pG | Cyclic phosphorylation | 15 | 1.95 | 100 | 1.2 | 6.81 | 696.796 | -7 | 4883.6191 | 4885.67 | 4883.6096 | 1406312.62 |
| 4085-4096 | Ar-pUr-pUr-pAr-pAr-pCr-pCr-pUr-pUr-pUr-pAr-pGr | Cyclic phosphorylation | 12 | 1.02 | 100 | 1 | 7.08 | 542.203 | -7 | 3801.4705 | 3802.97 | 3801.4666 | 1281763.25 |
| 4085-4096 | Ar-pUr-pUr-pAr-pAr-pCr-pCr-pUr-pUr-pUr-pAr-pGr | Cyclic phosphorylation | 12 | 0.69 | 100 | 1 | 7.12 | 632.738 | -6 | 3801.4692 | 3803.06 | 3801.4666 | 5502384.5 |
| 4085-4096 | Ar-pUr-pUr-pAr-pAr-pCr-pCr-pUr-pUr-pUr-pAr-pGr | Cyclic phosphorylation | 12 | 0.82 | 100 | 1 | 7.12 | 1266.483 | -3 | 3801.4697 | 3803.06 | 3801.4666 | 963880.19 |
| 4085-4096 | Ar-pUr-pUr-pAr-pAr-pCr-pCr-pUr-pUr-pUr-pAr-pGr | Cyclic phosphorylation | 12 | 0.76 | 100 | 1.1 | 7.13 | 759.487 | -5 | 3801.4695 | 3803.06 | 3801.4666 | 2659271.5 |
| 4085-4096 | Ar-pUr-pUr-pAr-pAr-pCr-pCr-pUr-pUr-pUr-pAr-pGr | Cyclic phosphorylation | 12 | 0.63 | 100 | 1 | 7.13 | 949.611 | -4 | 3801.469 | 3802.99 | 3801.4666 | 857381.75 |
| 3-12 | Ar-pAr-pUr-pAr-pAr-pAr-pCr-pUr-pAr-pGr | Cyclic phosphorylation | 10 | 0.95 | 100 | 1 | 7.27 | 808.358 | -4 | 3236.4575 | 3237.83 | 3236.4544 | 1967007.75 |
| 3-12 | Ar-pAr-pUr-pAr-pAr-pAr-pCr-pUr-pAr-pGr | Cyclic phosphorylation | 10 | 0.35 | 100 | 1 | 7.27 | 646.484 | -5 | 3236.4556 | 3237.71 | 3236.4544 | 4943302 |
| 3-12 | Ar-pAr-pUr-pAr-pAr-pAr-pCr-pUr-pAr-pGr | Cyclic phosphorylation | 10 | 1.1 | 100 | 1 | 7.27 | 1078.146 | -3 | 3236.458 | 3237.85 | 3236.4544 | 998479 |
| 3969-3984 | Cr-pUr-pCr-pCr-pCr-pAr-pCr-pCr-pUr-pCr-pCr-pAr-pCr-pCr-pUr- | Phosphorylation | 16 | 1.09 | 100 | 1.2 | 7.29 | 997.325 | -5 | 4989.6572 | 4991.8 | 4989.6518 | 1521396.5 |
| 3969-3984 | Cr-pUr-pCr-pCr-pCr-pAr-pCr-pCr-pUr-pCr-pCr-pAr-pCr-pCr-pUr- | Phosphorylation | 16 | 1.28 | 100 | 1.1 | 7.31 | 830.936 | -6 | 4989.6582 | 4991.75 | 4989.6518 | 3973131.25 |
| 3969-3984 | Cr-pUr-pCr-pCr-pCr-pAr-pCr-pCr-pUr-pCr-pCr-pAr-pCr-pCr-pUr- | Phosphorylation | 16 | 1.48 | 100 | 1 | 7.33 | 711.944 | -7 | 4989.6592 | 4991.75 | 4989.6518 | 2573133.5 |
| 133-145 | Cr-pCr-pUr-pAr-pCr-pAr-pCr-pCr-pAr-pAr-pCr-pAr-pGr | Phosphorylation | 13 | 1.56 | 100 | 1 | 7.41 | 1035.393 | -4 | 4144.6001 | 4146.28 | 4144.5936 | 1543608.88 |
| 133-145 | Cr-pCr-pUr-pAr-pCr-pAr-pCr-pCr-pAr-pAr-pCr-pAr-pGr | Phosphorylation | 13 | 1.33 | 100 | 1 | 7.41 | 1380.86 | -3 | 4144.5991 | 4146.28 | 4144.5936 | 1085617.12 |
| 133-145 | Cr-pCr-pUr-pAr-pCr-pAr-pCr-pCr-pAr-pAr-pCr-pAr-pGr | Phosphorylation | 13 | 1.21 | 100 | 1.2 | 7.41 | 828.112 | -5 | 4144.5986 | 4146.29 | 4144.5936 | 5031560.5 |
| 133-145 | Cr-pCr-pUr-pAr-pCr-pAr-pCr-pCr-pAr-pAr-pCr-pAr-pGr | Phosphorylation | 13 | 1.45 | 100 | 1 | 7.43 | 689.926 | -6 | 4144.5996 | 4146.27 | 4144.5936 | 3312873.75 |
| 2693-2704 | Cr-pAr-pCr-pAr-pAr-pUr-pCr-pAr-pCr-pAr-pAr-pGr | Cyclic phosphorylation | 12 | 1.39 | 100 | 1.1 | 7.55 | 1281.18 | -3 | 3845.5583 | 3847.22 | 3845.553 | 844905.25 |
| 2693-2704 | Cr-pAr-pCr-pAr-pAr-pUr-pCr-pAr-pCr-pAr-pAr-pGr | Cyclic phosphorylation | 12 | 1.14 | 100 | 1 | 7.55 | 960.633 | -4 | 3845.5574 | 3847.15 | 3845.553 | 1150790.38 |
| 2693-2704 | Cr-pAr-pCr-pAr-pAr-pUr-pCr-pAr-pCr-pAr-pAr-pGr | Cyclic phosphorylation | 12 | 1.01 | 100 | 1 | 7.57 | 768.304 | -5 | 3845.5569 | 3847.14 | 3845.553 | 2946306.75 |
| 2693-2704 | Cr-pAr-pCr-pAr-pAr-pUr-pCr-pAr-pCr-pAr-pAr-pGr | Cyclic phosphorylation | 12 | 1.45 | 100 | 1 | 7.6 | 640.086 | -6 | 3845.5586 | 3847.15 | 3845.553 | 2883764.75 |
| 4085-4096 | Ar-pUr-pUr-pAr-pAr-pCr-pCr-pUr-pUr-pUr-pAr-pGr | Phosphorylation | 12 | -0.16 | 100 | 1 | 7.84 | 763.088 | -5 | 3819.4766 | 3821.05 | 3819.4772 | 11539448 |
| 4085-4096 | Ar-pUr-pUr-pAr-pAr-pCr-pCr-pUr-pUr-pUr-pAr-pGr | Phosphorylation | 12 | 0.55 | 100 | 1 | 7.85 | 954.113 | -4 | 3819.4792 | 3821.04 | 3819.4772 | 2796034.5 |
| 4085-4096 | Ar-pUr-pUr-pAr-pAr-pCr-pCr-pUr-pUr-pUr-pAr-pGr | Phosphorylation | 12 | 1.38 | 100 | 1 | 7.88 | 635.74 | -6 | 3819.4824 | 3821.04 | 3819.4772 | 2922244.5 |
| 4085-4096 | Ar-pUr-pUr-pAr-pAr-pCr-pCr-pUr-pUr-pUr-pAr-pGr | Phosphorylation | 12 | 0.87 | 100 | 1 | 7.89 | 1272.487 | -3 | 3819.4805 | 3821.09 | 3819.4772 | 1825394.62 |
| 3393-3405 | Ar-pUr-pCr-pAr-pUr-pCr-pAr-pCr-pAr-pCr-pCr-pCr-pGr | Cyclic phosphorylation | 13 | 0.43 | 100 | 1.4 | 7.95 | 819.904 | -5 | 4103.5576 | 4105.23 | 4103.5558 | 8244225.5 |
| 3393-3405 | Ar-pUr-pCr-pAr-pUr-pCr-pAr-pCr-pAr-pCr-pCr-pCr-pGr | Cyclic phosphorylation | 13 | 0.79 | 100 | 1.2 | 7.96 | 1025.384 | -4 | 4103.5591 | 4105.28 | 4103.5558 | 2982010.75 |
| 3393-3405 | Ar-pUr-pCr-pAr-pUr-pCr-pAr-pCr-pAr-pCr-pCr-pCr-pGr | Cyclic phosphorylation | 13 | -0.04 | 100 | 1 | 7.96 | 683.085 | -6 | 4103.5557 | 4105.24 | 4103.5558 | 18055262 |
| 3393-3405 | Ar-pUr-pCr-pAr-pUr-pCr-pAr-pCr-pAr-pCr-pCr-pCr-pGr | Cyclic phosphorylation | 13 | 0.55 | 100 | 1.1 | 8.01 | 1367.18 | -3 | 4103.5581 | 4105.25 | 4103.5558 | 3243015.25 |
| 3393-3405 | Ar-pUr-pCr-pAr-pUr-pCr-pAr-pCr-pAr-pCr-pCr-pCr-pGr | Cyclic phosphorylation | 13 | 1.15 | 100 | 1.1 | 8.03 | 585.359 | -7 | 4103.5605 | 4105.21 | 4103.5558 | 5586521 |
| 3-12 | Ar-pAr-pUr-pAr-pAr-pAr-pCr-pUr-pAr-pGr | Phosphorylation | 10 | 0.18 | 100 | 1 | 8.14 | 812.859 | -4 | 3254.4656 | 3255.87 | 3254.465 | 7238114 |
| 3-12 | Ar-pAr-pUr-pAr-pAr-pAr-pCr-pUr-pAr-pGr | Phosphorylation | 10 | 0.7 | 100 | 1 | 8.14 | 1084.149 | -3 | 3254.4673 | 3255.86 | 3254.465 | 2149447.25 |
| 3-12 | Ar-pAr-pUr-pAr-pAr-pAr-pCr-pUr-pAr-pGr | Phosphorylation | 10 | 0.7 | 100 | 1 | 8.14 | 1626.728 | -2 | 3254.4673 | 3255.89 | 3254.465 | 1223159.88 |
| 3-12 | Ar-pAr-pUr-pAr-pAr-pAr-pCr-pUr-pAr-pGr | Phosphorylation | 10 | 0.18 | 100 | 1 | 8.2 | 650.086 | -5 | 3254.4656 | 3255.85 | 3254.465 | 6622660 |
| 2693-2704 | Cr-pAr-pCr-pAr-pAr-pUr-pCr-pAr-pCr-pAr-pAr-pGr | Phosphorylation | 12 | 1.43 | 100 | 1 | 8.67 | 965.135 | -4 | 3863.5691 | 3865.18 | 3863.5636 | 836734.81 |
| 2693-2704 | Cr-pAr-pCr-pAr-pAr-pUr-pCr-pAr-pCr-pAr-pAr-pGr | Phosphorylation | 12 | 1.24 | 100 | 1.1 | 8.68 | 771.907 | -5 | 3863.5684 | 3865.17 | 3863.5636 | 1818264.88 |
| 2693-2704 | Cr-pAr-pCr-pAr-pAr-pUr-pCr-pAr-pCr-pAr-pAr-pGr | Phosphorylation | 12 | 1.49 | 100 | 1 | 8.68 | 1287.183 | -3 | 3863.5693 | 3865.2 | 3863.5636 | 482429.09 |
| 2693-2704 | Cr-pAr-pCr-pAr-pAr-pUr-pCr-pAr-pCr-pAr-pAr-pGr | Phosphorylation | 12 | 2.38 | 100 | 1 | 8.71 | 643.088 | -6 | 3863.5728 | 3865.18 | 3863.5636 | 670614.06 |
| 3525-3535 | Ar-pAr-pCr-pCr-pAr-pCr-pAr-pCr-pAr-pAr-pGr | Cyclic phosphorylation | 11 | 0.38 | 100 | 1 | 9.21 | 589.081 | -6 | 3539.5291 | 3541.02 | 3539.5277 | 4456325.5 |
| 3525-3535 | Ar-pAr-pCr-pCr-pAr-pCr-pAr-pCr-pAr-pAr-pGr | Cyclic phosphorylation | 11 | 1.49 | 100 | 1.6 | 9.22 | 1769.26 | -2 | 3539.533 | 3541.04 | 3539.5277 | 580986 |
| 3525-3535 | Ar-pAr-pCr-pCr-pAr-pCr-pAr-pCr-pAr-pAr-pGr | Cyclic phosphorylation | 11 | 0.8 | 100 | 1 | 9.22 | 1179.17 | -3 | 3539.5305 | 3541.03 | 3539.5277 | 1010568.44 |
| 3525-3535 | Ar-pAr-pCr-pCr-pAr-pCr-pAr-pCr-pAr-pAr-pGr | Cyclic phosphorylation | 11 | -0.45 | 100 | 1 | 9.22 | 707.098 | -5 | 3539.5261 | 3541.03 | 3539.5277 | 7107260 |
| 3525-3535 | Ar-pAr-pCr-pCr-pAr-pCr-pAr-pCr-pAr-pAr-pGr | Cyclic phosphorylation | 11 | 0.59 | 100 | 1 | 9.22 | 884.125 | -4 | 3539.5298 | 3541.04 | 3539.5277 | 2799810 |
| 2548-2561 | Gr-pCr-pUr-pUr-pCr-pAr-pUr-pCr-pAr-pAr-pGr-pCr-pAr-pGr | Cyclic phosphorylation | 14 | 1.7 | 100 | 1.6 | 9.47 | 897.113 | -5 | 4489.6011 | 4491.54 | 4489.5934 | 446422.19 |
| 2548-2561 | Gr-pCr-pUr-pUr-pCr-pAr-pUr-pCr-pAr-pAr-pGr-pCr-pAr-pGr | Cyclic phosphorylation | 14 | 2.03 | 100 | 1 | 9.49 | 640.651 | -7 | 4489.6025 | 4491.52 | 4489.5934 | 916798.81 |
| 2548-2561 | Gr-pCr-pUr-pUr-pCr-pAr-pUr-pCr-pAr-pAr-pGr-pCr-pAr-pGr | Cyclic phosphorylation | 14 | 1.81 | 100 | 1 | 9.49 | 747.426 | -6 | 4489.6016 | 4491.53 | 4489.5934 | 1707415.88 |
| 2548-2561 | Gr-pCr-pUr-pUr-pCr-pAr-pUr-pCr-pAr-pAr-pGr-pCr-pAr-pGr | Cyclic phosphorylation | 14 | 2.03 | 100 | 1.1 | 9.49 | 1496.195 | -3 | 4489.6025 | 4491.54 | 4489.5934 | 264207.5 |
| 3273-3285 | Ar-pAr-pUr-pUr-pUr-pCr-pAr-pCr-pCr-pAr-pCr-pCr-pGr | Cyclic phosphorylation | 13 | 0.05 | 100 | 1.4 | 9.73 | 820.101 | -5 | 4104.54 | 4106.21 | 4104.5399 | 10425040 |
| 3273-3285 | Ar-pAr-pUr-pUr-pUr-pCr-pAr-pCr-pCr-pAr-pCr-pCr-pGr | Cyclic phosphorylation | 13 | 0.52 | 100 | 1.2 | 9.73 | 1025.379 | -4 | 4104.542 | 4106.23 | 4104.5399 | 3416065.25 |
| 3273-3285 | Ar-pAr-pUr-pUr-pUr-pCr-pAr-pCr-pCr-pAr-pCr-pCr-pGr | Cyclic phosphorylation | 13 | 0.52 | 100 | 1.2 | 9.75 | 1367.507 | -3 | 4104.542 | 4106.2 | 4104.5399 | 4283012 |
| 3273-3285 | Ar-pAr-pUr-pUr-pUr-pCr-pAr-pCr-pCr-pAr-pCr-pCr-pGr | Cyclic phosphorylation | 13 | -0.55 | 100 | 1 | 9.75 | 683.249 | -6 | 4104.5376 | 4106.22 | 4104.5399 | 22297932 |
| 3273-3285 | Ar-pAr-pUr-pUr-pUr-pCr-pAr-pCr-pCr-pAr-pCr-pCr-pGr | Cyclic phosphorylation | 13 | 1 | 100 | 1 | 9.75 | 585.499 | -7 | 4104.5439 | 4106.23 | 4104.5399 | 7080019.5 |
| 3525-3535 | Ar-pAr-pCr-pCr-pAr-pCr-pAr-pCr-pAr-pAr-pGr | Phosphorylation | 11 | 0.09 | 98.1 | 1 | 10.01 | 888.628 | -4 | 3557.5386 | 3559.03 | 3557.5383 | 4134342.75 |
| 3525-3535 | Ar-pAr-pCr-pCr-pAr-pCr-pAr-pCr-pAr-pAr-pGr | Phosphorylation | 11 | 1.05 | 98.1 | 1 | 10.01 | 1185.174 | -3 | 3557.542 | 3559.04 | 3557.5383 | 1469482.25 |
| 3525-3535 | Ar-pAr-pCr-pCr-pAr-pCr-pAr-pCr-pAr-pAr-pGr | Phosphorylation | 11 | 0.36 | 98.1 | 1.1 | 10.02 | 710.701 | -5 | 3557.5396 | 3559.06 | 3557.5383 | 4155868.75 |
| 3525-3535 | Ar-pAr-pCr-pCr-pAr-pCr-pAr-pCr-pAr-pAr-pGr | Phosphorylation | 11 | 1.32 | 98.1 | 1 | 10.03 | 1778.265 | -2 | 3557.543 | 3559.03 | 3557.5383 | 560652.69 |
| 4150-4165 | Ur-pGr-pCr-pCr-pAr-pGr-pCr-pCr-pAr-pCr-pAr-pCr-pCr-pCr-pUr | Cyclic phosphorylation | 16 | 2.1 | 96.6 | 1.5 | 10.18 | 845.109 | -6 | 5074.6914 | 5076.85 | 5074.6808 | 483107.22 |
| 4150-4165 | Ur-pGr-pCr-pCr-pAr-pGr-pCr-pCr-pAr-pCr-pAr-pCr-pCr-pCr-pUr | Cyclic phosphorylation | 16 | 2.58 | 96.6 | 1.5 | 10.2 | 724.235 | -7 | 5074.6938 | 5076.89 | 5074.6808 | 565588.19 |

| | | | | | | | | | | | | | |
|---|---|---|---|---|---|---|---|---|---|---|---|---|---|
| 345-358 | Ur-pCr-pCr-pAr-pAr-pCr-pAr-pUr-pCr-pAr-pUr-pCr-pAr-pGr | Cyclic phosphorylation | 14 | 1.41 | 100 | 1 | 10.55 | 632.507 | -7 | 4433.5986 | 4435.4 | 4433.5924 | 3225919.75 |
| 345-358 | Ur-pCr-pCr-pAr-pAr-pCr-pAr-pUr-pCr-pAr-pUr-pCr-pAr-pGr | Cyclic phosphorylation | 14 | 1.19 | 100 | 1.1 | 10.55 | 1477.193 | -3 | 4433.5977 | 4435.5 | 4433.5924 | 865803.31 |
| 345-358 | Ur-pCr-pCr-pAr-pAr-pCr-pAr-pUr-pCr-pAr-pUr-pCr-pAr-pGr | Cyclic phosphorylation | 14 | 1.63 | 100 | 1.1 | 10.55 | 1107.643 | -4 | 4433.5996 | 4435.54 | 4433.5924 | 580035.31 |
| 345-358 | Ur-pCr-pCr-pAr-pAr-pCr-pAr-pUr-pCr-pAr-pUr-pCr-pAr-pGr | Cyclic phosphorylation | 14 | 1.08 | 100 | 1 | 10.57 | 738.259 | -6 | 4433.5972 | 4435.53 | 4433.5924 | 3391850 |
| 246-261 | Ur-pUr-pCr-pCr-pAr-pCr-pGr-pCr-pCr-pAr-pUr-pCr-pCr-pAr-pCr | Cyclic phosphorylation | 16 | 1.56 | 100 | 1.1 | 10.66 | 838.604 | -6 | 5035.6665 | 5037.81 | 5035.6586 | 642032.5 |
| 246-261 | Ur-pUr-pCr-pCr-pAr-pCr-pGr-pCr-pCr-pAr-pUr-pCr-pCr-pAr-pCr | Cyclic phosphorylation | 16 | 1.66 | 100 | 1.1 | 10.66 | 718.517 | -7 | 5035.667 | 5037.76 | 5035.6586 | 1755810.38 |
| 246-261 | Ur-pUr-pCr-pCr-pAr-pCr-pGr-pCr-pCr-pAr-pUr-pCr-pCr-pAr-pCr | Cyclic phosphorylation | 16 | 2.24 | 100 | 1.2 | 10.67 | 628.702 | -8 | 5035.6699 | 5037.78 | 5035.6586 | 1346613.38 |
| 1493-1506 | Ur-pAr-pAr-pCr-pGr-pGr-pCr-pGr-pUr-pGr-pGr-pAr-pAr-pGr | Cyclic phosphorylation | 14 | 2.13 | 99.4 | 1.1 | 11.09 | 657.656 | -7 | 4608.6377 | 4610.61 | 4608.6279 | 971701.19 |
| 1493-1506 | Ur-pAr-pAr-pCr-pGr-pGr-pCr-pGr-pUr-pGr-pGr-pAr-pAr-pGr | Cyclic phosphorylation | 14 | 2.03 | 99.4 | 1.2 | 11.09 | 767.266 | -6 | 4608.6372 | 4610.5 | 4608.6279 | 867318.88 |
| 345-358 | Ur-pCr-pCr-pAr-pAr-pCr-pAr-pUr-pCr-pAr-pUr-pCr-pAr-pGr | Phosphorylation | 14 | 2.11 | 100 | 1 | 11.16 | 635.224 | -7 | 4451.6123 | 4453.46 | 4451.6029 | 261538.8 |
| 345-358 | Ur-pCr-pCr-pAr-pAr-pCr-pAr-pUr-pCr-pAr-pUr-pCr-pAr-pGr | Phosphorylation | 14 | 1.12 | 100 | 1 | 11.18 | 741.094 | -6 | 4451.6079 | 4453.53 | 4451.6029 | 3953173.5 |
| 3216-3233 | Ur-pCr-pUr-pGr-pCr-pCr-pCr-pCr-pUr-pCr-pAr-pCr-pGr-pGr-pCr | Cyclic phosphorylation | 18 | 2.13 | 100 | 1 | 11.18 | 634.63 | -9 | 5718.7334 | 5721.31 | 5718.7212 | 1569693.5 |
| 345-358 | Ur-pCr-pCr-pAr-pAr-pCr-pAr-pUr-pCr-pAr-pUr-pCr-pAr-pGr | Phosphorylation | 14 | 1.23 | 100 | 1.4 | 11.18 | 889.715 | -5 | 4451.6084 | 4453.56 | 4451.6029 | 2988482.25 |
| 3216-3233 | Ur-pCr-pUr-pGr-pCr-pCr-pCr-pCr-pUr-pCr-pAr-pCr-pGr-pGr-pCr | Cyclic phosphorylation | 18 | 1.71 | 100 | 1.1 | 11.19 | 714.084 | -8 | 5718.731 | 5721.23 | 5718.7212 | 2867131.25 |
| 3216-3233 | Ur-pCr-pUr-pGr-pCr-pCr-pCr-pCr-pUr-pCr-pAr-pCr-pGr-pGr-pCr | Cyclic phosphorylation | 18 | 1.79 | 100 | 1 | 11.19 | 816.241 | -7 | 5718.7314 | 5721.22 | 5718.7212 | 2317012.75 |
| 316-330 | Ar-pCr-pGr-pGr-pGr-pGr-pUr-pGr-pUr-pAr-pCr-pUr-pUr-pUr-pC | Cyclic phosphorylation | 15 | 1.28 | 100 | 1 | 11.38 | 694.794 | -7 | 4868.605 | 4870.66 | 4868.5987 | 2839929.5 |
| 316-330 | Ar-pCr-pGr-pGr-pGr-pGr-pUr-pGr-pUr-pAr-pCr-pUr-pUr-pUr-pC | Cyclic phosphorylation | 15 | 1.58 | 100 | 1 | 11.4 | 607.819 | -8 | 4868.6064 | 4870.62 | 4868.5987 | 1231684.12 |
| 2424-2438 | Ar-pCr-pCr-pCr-pCr-pUr-pCr-pCr-pUr-pAr-pUr-pCr-pAr-pAr-pGr | Cyclic phosphorylation | 15 | 1.69 | 100 | 1 | 11.49 | 588.572 | -8 | 4714.6304 | 4716.66 | 4714.6224 | 1595355.5 |
| 2424-2438 | Ar-pCr-pCr-pCr-pCr-pUr-pCr-pCr-pUr-pAr-pUr-pCr-pAr-pAr-pGr | Cyclic phosphorylation | 15 | 0.96 | 100 | 1.2 | 11.5 | 942.319 | -5 | 4714.627 | 4716.67 | 4714.6224 | 1481639.5 |
| 2424-2438 | Ar-pCr-pCr-pCr-pCr-pUr-pCr-pCr-pUr-pAr-pUr-pCr-pAr-pAr-pGr | Cyclic phosphorylation | 15 | 1.17 | 100 | 1 | 11.5 | 785.098 | -6 | 4714.6279 | 4716.62 | 4714.6224 | 5004285.5 |
| 2424-2438 | Ar-pCr-pCr-pCr-pCr-pUr-pCr-pCr-pUr-pAr-pUr-pCr-pAr-pAr-pGr | Cyclic phosphorylation | 15 | 1.27 | 100 | 1 | 11.5 | 672.797 | -7 | 4714.6284 | 4716.67 | 4714.6224 | 6915135 |
| 2424-2438 | Ar-pCr-pCr-pCr-pCr-pUr-pCr-pCr-pUr-pAr-pUr-pCr-pAr-pAr-pGr | Cyclic phosphorylation | 15 | 1.17 | 100 | 1.4 | 11.5 | 1570.869 | -3 | 4714.6279 | 4716.65 | 4714.6224 | 1324698.62 |
| 2424-2438 | Ar-pCr-pCr-pCr-pCr-pUr-pCr-pCr-pUr-pAr-pUr-pCr-pAr-pAr-pGr | Cyclic phosphorylation | 15 | 1.17 | 100 | 1 | 11.5 | 1177.9 | -4 | 4714.6279 | 4716.61 | 4714.6224 | 902049.38 |
| 749-763 | Cr-pAr-pUr-pCr-pAr-pAr-pCr-pAr-pUr-pCr-pAr-pCr-pCr-pCr-pGr | Cyclic phosphorylation | 15 | 2.32 | 100 | 1 | 11.57 | 676.086 | -7 | 4737.6606 | 4739.9 | 4737.6496 | 1523630.88 |
| 749-763 | Cr-pAr-pUr-pCr-pAr-pAr-pCr-pAr-pUr-pCr-pAr-pCr-pCr-pCr-pGr | Cyclic phosphorylation | 15 | 1.7 | 100 | 1.2 | 11.59 | 788.935 | -6 | 4737.6577 | 4739.89 | 4737.6496 | 1160199.75 |
| 3985-4002 | Cr-pCr-pCr-pCr-pAr-pCr-pUr-pCr-pAr-pCr-pCr-pAr-pCr-pCr-pUr- | Cyclic phosphorylation | 18 | 1.1 | 100 | 1.1 | 11.74 | 1400.929 | -4 | 5605.7412 | 5608.06 | 5605.7351 | 1210638.12 |
| 3985-4002 | Cr-pCr-pCr-pCr-pAr-pCr-pUr-pCr-pAr-pCr-pCr-pAr-pCr-pCr-pUr- | Cyclic phosphorylation | 18 | 0.49 | 100 | 1.4 | 11.76 | 1120.542 | -5 | 5605.7378 | 5608.06 | 5605.7351 | 1123714 |
| 3985-4002 | Cr-pCr-pCr-pCr-pAr-pCr-pUr-pCr-pAr-pCr-pCr-pAr-pCr-pCr-pUr- | Cyclic phosphorylation | 18 | 1.53 | 100 | 2 | 11.76 | 933.617 | -6 | 5605.7437 | 5608.24 | 5605.7351 | 3906732.5 |
| 3985-4002 | Cr-pCr-pCr-pCr-pAr-pCr-pUr-pCr-pAr-pCr-pCr-pAr-pCr-pCr-pUr- | Cyclic phosphorylation | 18 | 1.19 | 100 | 1.1 | 11.76 | 800.098 | -7 | 5605.7417 | 5608.22 | 5605.7351 | 8832890 |
| 3985-4002 | Cr-pCr-pCr-pCr-pAr-pCr-pUr-pCr-pAr-pCr-pCr-pAr-pCr-pCr-pUr- | Cyclic phosphorylation | 18 | 1.45 | 100 | 1 | 11.76 | 699.96 | -8 | 5605.7432 | 5608.06 | 5605.7351 | 9980508 |
| 3985-4002 | Cr-pCr-pCr-pCr-pAr-pCr-pUr-pCr-pAr-pCr-pCr-pAr-pCr-pCr-pUr- | Cyclic phosphorylation | 18 | 1.97 | 100 | 1.2 | 11.76 | 621.965 | -9 | 5605.7461 | 5608.05 | 5605.7351 | 1568741.5 |
| 3112-3129 | Cr-pCr-pUr-pCr-pUr-pGr-pCr-pCr-pAr-pAr-pUr-pCr-pUr-pGr-pG | Cyclic phosphorylation | 18 | 1.33 | 100 | 1 | 11.9 | 712.085 | -8 | 5702.7339 | 5705.22 | 5702.7263 | 3944107 |
| 3112-3129 | Cr-pCr-pUr-pCr-pUr-pGr-pCr-pCr-pAr-pAr-pUr-pCr-pUr-pGr-pG | Cyclic phosphorylation | 18 | 1.59 | 100 | 1.4 | 11.9 | 949.782 | -6 | 5702.7354 | 5705.06 | 5702.7263 | 1980220 |
| 3112-3129 | Cr-pCr-pUr-pCr-pUr-pGr-pCr-pCr-pAr-pAr-pUr-pCr-pUr-pGr-pG | Cyclic phosphorylation | 18 | 1.16 | 100 | 1.1 | 11.9 | 813.955 | -7 | 5702.7329 | 5705.23 | 5702.7263 | 4893469.5 |
| 2424-2438 | Ar-pCr-pCr-pCr-pCr-pUr-pCr-pCr-pUr-pAr-pUr-pCr-pAr-pAr-pGr | Phosphorylation | 15 | 0.89 | 100 | 1.2 | 11.93 | 945.921 | -5 | 4732.6372 | 4734.66 | 4732.633 | 2620366.75 |
| 2424-2438 | Ar-pCr-pCr-pCr-pCr-pUr-pCr-pCr-pUr-pAr-pUr-pCr-pAr-pAr-pGr | Phosphorylation | 15 | 0.79 | 100 | 1 | 11.93 | 1576.873 | -3 | 4732.6367 | 4734.74 | 4732.633 | 835466.94 |
| 2424-2438 | Ar-pCr-pCr-pCr-pCr-pUr-pCr-pCr-pUr-pAr-pUr-pCr-pAr-pAr-pGr | Phosphorylation | 15 | 1.41 | 100 | 1.1 | 11.94 | 787.932 | -6 | 4732.6396 | 4734.69 | 4732.633 | 3924376.5 |
| 2424-2438 | Ar-pCr-pCr-pCr-pCr-pUr-pCr-pCr-pUr-pAr-pUr-pCr-pAr-pAr-pGr | Phosphorylation | 15 | 1.3 | 100 | 1 | 11.95 | 675.227 | -7 | 4732.6392 | 4734.69 | 4732.633 | 926282.56 |
| 2916-2930 | Ar-pAr-pUr-pGr-pCr-pCr-pCr-pAr-pGr-pGr-pCr-pAr-pCr-pUr-pG | Cyclic phosphorylation | 15 | 1.9 | 100 | 1.2 | 12 | 965.926 | -5 | 4833.666 | 4835.76 | 4833.6569 | 525764.75 |
| 2916-2930 | Ar-pAr-pUr-pGr-pCr-pCr-pCr-pAr-pGr-pGr-pCr-pAr-pCr-pUr-pG | Cyclic phosphorylation | 15 | 1.69 | 100 | 1.1 | 12.02 | 689.802 | -7 | 4833.665 | 4835.84 | 4833.6569 | 3280062.25 |
| 2916-2930 | Ar-pAr-pUr-pGr-pCr-pCr-pCr-pAr-pGr-pGr-pCr-pAr-pCr-pUr-pG | Cyclic phosphorylation | 15 | 1.69 | 100 | 1.2 | 12.04 | 804.937 | -6 | 4833.665 | 4835.85 | 4833.6569 | 1593659.75 |
| 3138-3152 | Ar-pUr-pGr-pUr-pCr-pUr-pGr-pAr-pGr-pUr-pGr-pUr-pGr-pUr-pC | Phosphorylation | 15 | 5.49 | 95.1 | 1.1 | 12.05 | 813.596 | -6 | 4887.6201 | 4890.93 | 4887.5933 | 529160.31 |
| 3138-3152 | Ar-pUr-pGr-pUr-pCr-pUr-pGr-pAr-pGr-pUr-pGr-pUr-pGr-pUr-pC | Phosphorylation | 15 | 5.29 | 95.1 | 1.4 | 12.05 | 697.224 | -7 | 4887.6191 | 4891.13 | 4887.5933 | 466975.25 |
| 3985-4002 | Cr-pCr-pCr-pCr-pAr-pCr-pUr-pCr-pAr-pCr-pCr-pAr-pCr-pCr-pUr- | Phosphorylation | 18 | 0.95 | 100 | 1.1 | 12.26 | 1124.144 | -5 | 5623.751 | 5626.21 | 5623.7456 | 1178141.62 |
| 3985-4002 | Cr-pCr-pCr-pCr-pAr-pCr-pUr-pCr-pAr-pCr-pCr-pAr-pCr-pCr-pUr- | Phosphorylation | 18 | 1.3 | 100 | 1 | 12.26 | 802.671 | -7 | 5623.7529 | 5626.06 | 5623.7456 | 5556492 |
| 3985-4002 | Cr-pCr-pCr-pCr-pAr-pCr-pUr-pCr-pAr-pCr-pCr-pAr-pCr-pCr-pUr- | Phosphorylation | 18 | 1.21 | 100 | 1.4 | 12.26 | 936.618 | -6 | 5623.7524 | 5626.17 | 5623.7456 | 4745685 |
| 3985-4002 | Cr-pCr-pCr-pCr-pAr-pCr-pUr-pCr-pAr-pCr-pCr-pAr-pCr-pCr-pUr- | Phosphorylation | 18 | 1.56 | 100 | 1 | 12.26 | 702.212 | -8 | 5623.7544 | 5626.22 | 5623.7456 | 2094471.75 |
| 3985-4002 | Cr-pCr-pCr-pCr-pAr-pCr-pUr-pCr-pAr-pCr-pCr-pAr-pCr-pCr-pUr- | Phosphorylation | 18 | 0.69 | 100 | 1 | 12.26 | 1405.432 | -4 | 5623.7495 | 5626.03 | 5623.7456 | 724093.19 |
| 3985-4002 | Cr-pCr-pCr-pCr-pAr-pCr-pUr-pCr-pAr-pCr-pCr-pAr-pCr-pCr-pUr- | Phosphorylation | 18 | 1.21 | 100 | 1.6 | 12.26 | 1874.247 | -3 | 5623.7524 | 5626.23 | 5623.7456 | 698417.44 |
| 2628-2645 | Ar-pCr-pAr-pGr-pUr-pGr-pCr-pUr-pGr-pCr-pCr-pUr-pCr-pCr-pU | Cyclic phosphorylation | 18 | 1.48 | 99.9 | 1.2 | 12.35 | 712.208 | -8 | 5703.7188 | 5706.24 | 5703.7103 | 621615.25 |
| 4152-4168 | Cr-pCr-pAr-pGr-pCr-pCr-pAr-pCr-pAr-pCr-pCr-pCr-pUr-pGr-pGr- | Cyclic phosphorylation | 17 | 1.97 | 100 | 1.2 | 12.37 | 776.817 | -7 | 5442.7661 | 5445.01 | 5442.7554 | 775185.81 |
| 4152-4168 | Cr-pCr-pAr-pGr-pCr-pCr-pAr-pCr-pAr-pCr-pCr-pCr-pUr-pGr-pGr- | Cyclic phosphorylation | 17 | 1.7 | 100 | 1.3 | 12.38 | 679.589 | -8 | 5442.7646 | 5445.03 | 5442.7554 | 657469.38 |
| 78-95 | Cr-pCr-pUr-pCr-pUr-pGr-pGr-pUr-pGr-pUr-pCr-pCr-pAr-pGr-pC | Cyclic phosphorylation | 18 | 2.3 | 100 | 1 | 12.59 | 717.212 | -8 | 5742.7456 | 5745.31 | 5742.7324 | 691913.19 |
| 1084-1101 | Cr-pCr-pAr-pCr-pCr-pAr-pGr-pAr-pUr-pUr-pCr-pGr-pCr-pCr-pUr | Cyclic phosphorylation | 18 | 2.42 | 100 | 1.1 | 12.59 | 710.087 | -8 | 5686.7451 | 5689.3 | 5686.7314 | 630273 |
| 78-95 | Cr-pCr-pUr-pCr-pUr-pGr-pGr-pUr-pGr-pUr-pCr-pCr-pAr-pGr-pC | Cyclic phosphorylation | 18 | 3.23 | 100 | 1.2 | 12.59 | 819.815 | -7 | 5742.751 | 5745.33 | 5742.7324 | 620667.62 |
| 3797-3814 | Ur-pAr-pGr-pCr-pUr-pGr-pUr-pGr-pGr-pCr-pAr-pGr-pCr-pUr-pG | Cyclic phosphorylation | 18 | 2.9 | 100 | 1.1 | 12.75 | 727.211 | -8 | 5823.7456 | 5826.28 | 5823.7287 | 912210.62 |
| 3797-3814 | Ur-pAr-pGr-pCr-pUr-pGr-pUr-pGr-pGr-pCr-pAr-pGr-pCr-pUr-pG | Cyclic phosphorylation | 18 | 2.14 | 100 | 2 | 12.75 | 646.298 | -9 | 5823.7412 | 5826.45 | 5823.7287 | 545066.25 |
| 1728-1745 | Ur-pUr-pCr-pCr-pUr-pGr-pCr-pCr-pAr-pUr-pUr-pCr-pCr-pAr-pG | Cyclic phosphorylation | 18 | 1.62 | 100 | 1 | 12.94 | 811.811 | -7 | 5687.7246 | 5689.87 | 5687.7154 | 882041.56 |
| 1728-1745 | Ur-pUr-pCr-pCr-pUr-pGr-pCr-pCr-pAr-pUr-pUr-pCr-pCr-pAr-pG | Cyclic phosphorylation | 18 | 2.74 | 100 | 1 | 12.94 | 710.209 | -8 | 5687.731 | 5689.9 | 5687.7154 | 1621716.62 |
| 1914-1932 | Cr-pCr-pCr-pGr-pUr-pGr-pGr-pCr-pCr-pAr-pUr-pUr-pCr-pAr-pCr | Cyclic phosphorylation | 19 | 1.67 | 100 | 1.3 | 13.18 | 863.108 | -7 | 6046.7998 | 6049.42 | 6046.7897 | 475517.06 |
| 1561-1579 | Ur-pGr-pGr-pGr-pCr-pUr-pAr-pUr-pCr-pAr-pGr-pCr-pCr-pCr-pU | Cyclic phosphorylation | 19 | 0.59 | 98.6 | 1 | 13.18 | 761.218 | -8 | 6095.7998 | 6098.49 | 6095.7962 | 1091894.5 |
| 1914-1932 | Cr-pCr-pCr-pGr-pUr-pGr-pGr-pCr-pCr-pAr-pUr-pUr-pCr-pAr-pCr | Cyclic phosphorylation | 19 | 2.64 | 100 | 1.1 | 13.2 | 755.219 | -8 | 6046.8057 | 6049.5 | 6046.7897 | 561898.69 |
| 1312-1325 | Ar-pCr-pUr-pAr-pCr-pAr-pAr-pCr-pUr-pAr-pCr-pAr-pAr-pGr | Cyclic phosphorylation | 14 | 0.88 | 100 | 1.1 | 13.35 | 895.32 | -5 | 4480.6348 | 4482.59 | 4480.6308 | 4027205.75 |
| 1312-1325 | Ar-pCr-pUr-pAr-pCr-pAr-pAr-pCr-pUr-pAr-pCr-pAr-pAr-pGr | Cyclic phosphorylation | 14 | 0.44 | 100 | 1.2 | 13.35 | 745.931 | -6 | 4480.6328 | 4482.59 | 4480.6308 | 12047220 |
| 1312-1325 | Ar-pCr-pUr-pAr-pCr-pAr-pAr-pCr-pUr-pAr-pCr-pAr-pAr-pGr | Cyclic phosphorylation | 14 | 0.66 | 100 | 1.2 | 13.35 | 1493.205 | -3 | 4480.6338 | 4482.6 | 4480.6308 | 2480365.25 |
| 1312-1325 | Ar-pCr-pUr-pAr-pCr-pAr-pAr-pCr-pUr-pAr-pCr-pAr-pAr-pGr | Cyclic phosphorylation | 14 | 0.77 | 100 | 1 | 13.35 | 1119.652 | -4 | 4480.6343 | 4482.58 | 4480.6308 | 1756479.38 |
| 1312-1325 | Ar-pCr-pUr-pAr-pCr-pAr-pAr-pCr-pUr-pAr-pCr-pAr-pAr-pGr | Cyclic phosphorylation | 14 | 1.21 | 100 | 1 | 13.35 | 639.226 | -7 | 4480.6362 | 4482.59 | 4480.6308 | 6977771 |
| 63-83 | Ur-pUr-pCr-pCr-pUr-pGr-pGr-pUr-pGr-pCr-pUr-pGr-pCr-pUr-pG | Cyclic phosphorylation | 21 | 2.11 | 99.9 | 1 | 13.44 | 738.525 | -9 | 6653.79 | 6656.71 | 6653.776 | 964061.44 |
| 63-83 | Ur-pUr-pCr-pCr-pUr-pGr-pGr-pUr-pGr-pCr-pUr-pGr-pCr-pUr-pG | Cyclic phosphorylation | 21 | -0.54 | 99.9 | 1 | 13.44 | 664.572 | -10 | 6653.7725 | 6656.63 | 6653.776 | 700205.75 |
| 63-83 | Ur-pUr-pCr-pCr-pUr-pGr-pGr-pUr-pGr-pCr-pUr-pGr-pCr-pUr-pG | Cyclic phosphorylation | 21 | 0.64 | 99.9 | 1.3 | 13.45 | 831.217 | -8 | 6653.7803 | 6656.75 | 6653.776 | 497542.06 |
| 198-215 | Cr-pAr-pCr-pUr-pCr-pUr-pAr-pCr-pCr-pCr-pAr-pGr-pGr-pAr-pCr | Cyclic phosphorylation | 18 | 1.83 | 99.9 | 1.1 | 13.58 | 814.96 | -7 | 5709.769 | 5712.37 | 5709.7586 | 943764.5 |
| 198-215 | Cr-pAr-pCr-pUr-pCr-pUr-pAr-pCr-pCr-pCr-pAr-pGr-pGr-pAr-pCr | Cyclic phosphorylation | 18 | 3.71 | 99.9 | 1.5 | 13.59 | 712.965 | -8 | 5709.7798 | 5712.06 | 5709.7586 | 542731.06 |
| 1394-1409 | Cr-pAr-pAr-pCr-pUr-pAr-pCr-pAr-pAr-pUr-pUr-pAr-pCr-pCr-pU | Cyclic phosphorylation | 16 | 1.49 | 100 | 1 | 13.66 | 632.827 | -8 | 5068.6777 | 5070.79 | 5068.6702 | 4913661 |
| 1394-1409 | Cr-pAr-pAr-pCr-pUr-pAr-pCr-pAr-pAr-pUr-pUr-pAr-pCr-pCr-pU | Cyclic phosphorylation | 16 | 0.52 | 100 | 1 | 13.66 | 723.375 | -7 | 5068.6729 | 5070.8 | 5068.6702 | 13237237 |
| 1394-1409 | Cr-pAr-pAr-pCr-pUr-pAr-pCr-pAr-pAr-pUr-pUr-pAr-pCr-pCr-pU | Cyclic phosphorylation | 16 | 1.2 | 100 | 1.6 | 13.66 | 1689.22 | -3 | 5068.6763 | 5070.88 | 5068.6702 | 1576923 |
| 1394-1409 | Cr-pAr-pAr-pCr-pUr-pAr-pCr-pAr-pAr-pUr-pUr-pAr-pCr-pCr-pU | Cyclic phosphorylation | 16 | 0.72 | 100 | 1.3 | 13.67 | 844.106 | -6 | 5068.6738 | 5070.84 | 5068.6702 | 5094996 |
| 1312-1325 | Ar-pCr-pUr-pAr-pCr-pAr-pAr-pCr-pUr-pAr-pCr-pAr-pAr-pGr | Phosphorylation | 14 | 0.48 | 100 | 1 | 13.7 | 748.933 | -6 | 4498.6436 | 4500.61 | 4498.6414 | 6040832.5 |
| 1312-1325 | Ar-pCr-pUr-pAr-pCr-pAr-pAr-pCr-pUr-pAr-pCr-pAr-pAr-pGr | Phosphorylation | 14 | 0.7 | 100 | 1.1 | 13.7 | 898.922 | -5 | 4498.6445 | 4500.62 | 4498.6414 | 6166476 |
| 1312-1325 | Ar-pCr-pUr-pAr-pCr-pAr-pAr-pCr-pUr-pAr-pCr-pAr-pAr-pGr | Phosphorylation | 14 | 0.7 | 100 | 1 | 13.71 | 1498.875 | -3 | 4498.6445 | 4500.58 | 4498.6414 | 1720915.12 |
| 897-912 | Ar-pAr-pCr-pGr-pGr-pCr-pAr-pCr-pCr-pAr-pUr-pCr-pAr-pCr-pCr- | Cyclic phosphorylation | 16 | 1.05 | 100 | 1.1 | 13.71 | 852.947 | -6 | 5121.7246 | 5123.86 | 5121.7192 | 2962535.5 |
| 1312-1325 | Ar-pCr-pUr-pAr-pCr-pAr-pAr-pCr-pUr-pAr-pCr-pAr-pAr-pGr | Phosphorylation | 14 | 0.92 | 100 | 1 | 13.71 | 1123.905 | -4 | 4498.6455 | 4500.59 | 4498.6414 | 1774907.62 |
| 897-912 | Ar-pAr-pCr-pGr-pGr-pCr-pAr-pCr-pCr-pAr-pUr-pCr-pAr-pCr-pCr- | Cyclic phosphorylation | 16 | 1.24 | 100 | 1 | 13.73 | 730.81 | -7 | 5121.7256 | 5123.89 | 5121.7192 | 5186876.5 |
| 4037-4053 | Cr-pAr-pGr-pCr-pUr-pCr-pAr-pAr-pAr-pAr-pCr-pGr-pCr-pUr-pU | Cyclic phosphorylation | 17 | 1.25 | 98.2 | 1.1 | 13.9 | 778.243 | -7 | 5452.7466 | 5454.97 | 5452.7398 | 1716812.88 |
| 4037-4053 | Cr-pAr-pGr-pCr-pUr-pCr-pAr-pAr-pAr-pAr-pCr-pGr-pCr-pUr-pU | Cyclic phosphorylation | 17 | 1.52 | 98.2 | 1.4 | 13.91 | 680.837 | -8 | 5452.748 | 5455.13 | 5452.7398 | 1322264 |
| 3406-3423 | Ar-pCr-pAr-pAr-pCr-pAr-pCr-pCr-pUr-pUr-pCr-pGr-pUr-pGr-pU | Cyclic phosphorylation | 18 | 2.04 | 100 | 1 | 14.06 | 633.853 | -9 | 5711.7383 | 5714.07 | 5711.7266 | 1206061.75 |
| 3406-3423 | Ar-pCr-pAr-pAr-pCr-pAr-pCr-pCr-pUr-pUr-pCr-pGr-pUr-pGr-pU | Cyclic phosphorylation | 18 | 1.44 | 100 | 1 | 14.06 | 713.21 | -8 | 5711.7349 | 5714.19 | 5711.7266 | 2718775.25 |
| 3406-3423 | Ar-pCr-pAr-pAr-pCr-pAr-pCr-pCr-pUr-pUr-pCr-pGr-pUr-pGr-pU | Cyclic phosphorylation | 18 | 0.85 | 100 | 1.1 | 14.07 | 815.241 | -7 | 5711.7314 | 5714.16 | 5711.7266 | 2181241 |
| 672-690 | Cr-pAr-pCr-pAr-pCr-pCr-pCr-pCr-pUr-pAr-pUr-pCr-pAr-pAr-pCr- | Cyclic phosphorylation | 19 | 1.72 | 100 | 1.3 | 14.19 | 661.305 | -9 | 5958.8091 | 5961.16 | 5958.7988 | 2069260.88 |
| 672-690 | Cr-pAr-pCr-pAr-pCr-pCr-pCr-pCr-pUr-pAr-pUr-pCr-pAr-pAr-pCr- | Cyclic phosphorylation | 19 | 1.23 | 100 | 1.4 | 14.19 | 850.536 | -7 | 5958.8062 | 5961.38 | 5958.7988 | 14865773 |
| 672-690 | Cr-pAr-pCr-pAr-pCr-pCr-pCr-pCr-pUr-pAr-pUr-pCr-pAr-pAr-pCr- | Cyclic phosphorylation | 19 | 1.4 | 100 | 1 | 14.19 | 744.093 | -8 | 5958.8071 | 5961.37 | 5958.7988 | 11321724 |
| 672-690 | Cr-pAr-pCr-pAr-pCr-pCr-pCr-pCr-pUr-pAr-pUr-pCr-pAr-pAr-pCr- | Cyclic phosphorylation | 19 | 0.91 | 100 | 1.7 | 14.19 | 992.461 | -6 | 5958.8042 | 5961.38 | 5958.7988 | 6210900 |
| 672-690 | Cr-pAr-pCr-pAr-pCr-pCr-pCr-pCr-pUr-pAr-pUr-pCr-pAr-pAr-pCr- | Cyclic phosphorylation | 19 | 1.15 | 100 | 1.3 | 14.19 | 1191.155 | -5 | 5958.8057 | 5961.16 | 5958.7988 | 1756393.88 |
| 672-690 | Cr-pAr-pCr-pAr-pCr-pCr-pCr-pCr-pUr-pAr-pUr-pCr-pAr-pAr-pCr- | Cyclic phosphorylation | 19 | 0.66 | 100 | 1 | 14.2 | 1489.195 | -4 | 5958.8027 | 5961.33 | 5958.7988 | 1705303 |
| 2447-2465 | Cr-pGr-pGr-pCr-pUr-pUr-pCr-pAr-pAr-pUr-pUr-pUr-pCr-pAr-pG | Cyclic phosphorylation | 19 | 1.19 | 100 | 1.1 | 14.21 | 864.533 | -7 | 6056.7812 | 6059.37 | 6056.774 | 6013711.5 |
| 2447-2465 | Cr-pGr-pGr-pCr-pUr-pUr-pCr-pAr-pAr-pUr-pUr-pUr-pCr-pAr-pG | Cyclic phosphorylation | 19 | 1.11 | 100 | 1 | 14.23 | 756.34 | -8 | 6056.7808 | 6059.43 | 6056.774 | 9158691 |
| 2447-2465 | Cr-pGr-pGr-pCr-pUr-pUr-pCr-pAr-pAr-pUr-pUr-pUr-pCr-pAr-pG | Cyclic phosphorylation | 19 | 1.59 | 100 | 1.2 | 14.23 | 672.303 | -9 | 6056.7837 | 6059.18 | 6056.774 | 1501042.12 |
| 2447-2465 | Cr-pGr-pGr-pCr-pUr-pUr-pCr-pAr-pAr-pUr-pUr-pUr-pCr-pAr-pG | Cyclic phosphorylation | 19 | 0.54 | 100 | 1.6 | 14.23 | 1008.79 | -6 | 6056.7773 | 6059.47 | 6056.774 | 1756738.12 |
| 1671-1686 | Ar-pAr-pCr-pUr-pUr-pCr-pAr-pAr-pCr-pUr-pUr-pCr-pAr-pAr-pCr | Cyclic phosphorylation | 16 | 1.49 | 100 | 1 | 14.33 | 632.828 | -8 | 5068.6777 | 5070.77 | 5068.6702 | 4970555 |
| 1671-1686 | Ar-pAr-pCr-pUr-pUr-pCr-pAr-pAr-pCr-pUr-pUr-pCr-pAr-pAr-pCr | Cyclic phosphorylation | 16 | 0.43 | 100 | 1.1 | 14.35 | 723.374 | -7 | 5068.6724 | 5070.85 | 5068.6702 | 34362480 |
| 1671-1686 | Ar-pAr-pCr-pUr-pUr-pCr-pAr-pAr-pCr-pUr-pUr-pCr-pAr-pAr-pCr | Cyclic phosphorylation | 16 | 0.52 | 100 | 1.1 | 14.35 | 1266.663 | -4 | 5068.6729 | 5070.79 | 5068.6702 | 3655560.5 |
| 1671-1686 | Ar-pAr-pCr-pUr-pUr-pCr-pAr-pAr-pCr-pUr-pUr-pCr-pAr-pAr-pCr | Cyclic phosphorylation | 16 | 0.52 | 100 | 1.6 | 14.35 | 1013.128 | -5 | 5068.6729 | 5070.81 | 5068.6702 | 5883654 |
| 1671-1686 | Ar-pAr-pCr-pUr-pUr-pCr-pAr-pAr-pCr-pUr-pUr-pCr-pAr-pAr-pCr | Cyclic phosphorylation | 16 | 0.24 | 100 | 1.5 | 14.35 | 844.104 | -6 | 5068.6714 | 5070.83 | 5068.6702 | 25035126 |
| 1671-1686 | Ar-pAr-pCr-pUr-pUr-pCr-pAr-pAr-pCr-pUr-pUr-pCr-pAr-pAr-pCr | Cyclic phosphorylation | 16 | 1.2 | 100 | 1.5 | 14.36 | 1688.885 | -3 | 5068.6763 | 5070.78 | 5068.6702 | 4119192.25 |
| 2447-2465 | Cr-pGr-pGr-pCr-pUr-pUr-pCr-pAr-pAr-pUr-pUr-pUr-pCr-pAr-pG | Phosphorylation | 19 | 1.62 | 99.9 | 1.4 | 14.5 | 1011.793 | -6 | 6074.7944 | 6077.54 | 6074.7846 | 366457.78 |
| 2447-2465 | Cr-pGr-pGr-pCr-pUr-pUr-pCr-pAr-pAr-pUr-pUr-pUr-pCr-pAr-pG | Phosphorylation | 19 | 1.06 | 99.9 | 1.1 | 14.52 | 867.107 | -7 | 6074.791 | 6077.59 | 6074.7846 | 674807.25 |
| 672-690 | Cr-pAr-pCr-pAr-pCr-pCr-pCr-pCr-pUr-pAr-pUr-pCr-pAr-pAr-pCr- | Phosphorylation | 19 | 1.67 | 100 | 1.1 | 14.59 | 1194.758 | -5 | 5976.8193 | 5979.45 | 5976.8094 | 334340.38 |
| 672-690 | Cr-pAr-pCr-pAr-pCr-pCr-pCr-pCr-pUr-pAr-pUr-pCr-pAr-pAr-pCr- | Phosphorylation | 19 | 2.81 | 100 | 1.6 | 14.61 | 995.464 | -6 | 5976.8262 | 5979.41 | 5976.8094 | 1308887.25 |
| 672-690 | Cr-pAr-pCr-pAr-pCr-pCr-pCr-pCr-pUr-pAr-pUr-pCr-pAr-pAr-pCr- | Phosphorylation | 19 | 2.16 | 100 | 1 | 14.62 | 853.11 | -7 | 5976.8223 | 5979.4 | 5976.8094 | 2240846.75 |
| 672-690 | Cr-pAr-pCr-pAr-pCr-pCr-pCr-pCr-pUr-pAr-pUr-pCr-pAr-pAr-pCr- | Phosphorylation | 19 | 2.4 | 100 | 1 | 14.62 | 746.346 | -8 | 5976.8237 | 5979.49 | 5976.8094 | 1229418.12 |
| 1671-1686 | Ar-pAr-pCr-pUr-pUr-pCr-pAr-pAr-pCr-pUr-pUr-pCr-pAr-pAr-pCr | Cyclic phosphorylation | 16 | 2.55 | 92.6 | 1.5 | 14.7 | 723.377 | -7 | 5068.6831 | 5070.58 | 5068.6702 | 39527.59 |
| 1671-1686 | Ar-pAr-pCr-pUr-pUr-pCr-pAr-pAr-pCr-pUr-pUr-pCr-pAr-pAr-pCr | Phosphorylation | 16 | 2.48 | 100 | 1 | 14.73 | 725.949 | -7 | 5086.6934 | 5088.86 | 5086.6808 | 1423335.62 |
| 1671-1686 | Ar-pAr-pCr-pUr-pUr-pCr-pAr-pAr-pCr-pUr-pUr-pCr-pAr-pAr-pCr | Phosphorylation | 16 | 2.09 | 100 | 1.2 | 14.76 | 847.108 | -6 | 5086.6914 | 5088.86 | 5086.6808 | 1937223.38 |
| 1671-1686 | Ar-pAr-pCr-pUr-pUr-pCr-pAr-pAr-pCr-pUr-pUr-pCr-pAr-pAr-pCr | Phosphorylation | 16 | 2.09 | 100 | 1.5 | 14.76 | 1016.732 | -5 | 5086.6914 | 5088.87 | 5086.6808 | 685057.19 |
| 1918-1937 | Ur-pGr-pGr-pCr-pCr-pAr-pUr-pUr-pCr-pAr-pCr-pGr-pCr-pCr-pG | Cyclic phosphorylation | 20 | 2.66 | 98.7 | 1.1 | 14.82 | 799.35 | -8 | 6400.8545 | 6403.71 | 6400.8375 | 607856.12 |
| 1918-1937 | Ur-pGr-pGr-pCr-pCr-pAr-pUr-pUr-pCr-pAr-pCr-pGr-pCr-pCr-pG | Cyclic phosphorylation | 20 | 2.66 | 98.7 | 1.1 | 14.83 | 710.422 | -9 | 6400.8545 | 6403.72 | 6400.8375 | 584745.19 |
| 1918-1937 | Ur-pGr-pGr-pCr-pCr-pAr-pUr-pUr-pCr-pAr-pCr-pGr-pCr-pCr-pG | Cyclic phosphorylation | 20 | 2.2 | 98.7 | 2 | 14.83 | 913.687 | -7 | 6400.8516 | 6403.74 | 6400.8375 | 435004.69 |
| 697-717 | Ar-pUr-pCr-pUr-pGr-pCr-pCr-pUr-pCr-pAr-pGr-pGr-pGr-pCr-pU | Cyclic phosphorylation | 21 | 3.78 | 99.9 | 1 | 14.97 | 739.307 | -9 | 6660.8335 | 6663.89 | 6660.8083 | 1299371.5 |

| | | | | | | | | | | | | |
|---|---|---|---|---|---|---|---|---|---|---|---|---|
| 1255-1272 | Ur-pGr-pAr-pUr-pCr-pCr-pGr-pGr-pGr-pGr-pAr-pGr-pAr-pUr-pG | Cyclic phosphorylation | 18 | 2.34 | 100 | 1 | 14.97 | 740.97 | -8 | 5933.8145 | 5936.47 | 5933.8006 | 588607 |
| 697-717 | Ar-pUr-pCr-pUr-pGr-pCr-pCr-pUr-pCr-pAr-pGr-pGr-pGr-pCr-pU | Cyclic phosphorylation | 21 | 2.39 | 99.9 | 1 | 14.97 | 831.846 | -8 | 6660.8242 | 6663.8 | 6660.8083 | 596112.38 |
| 1255-1272 | Ur-pGr-pAr-pUr-pCr-pCr-pGr-pGr-pGr-pGr-pAr-pGr-pAr-pUr-pG | Cyclic phosphorylation | 18 | 1.68 | 100 | 1 | 14.99 | 846.967 | -7 | 5933.8105 | 5936.14 | 5933.8006 | 848190 |
| 3950-3968 | Ar-pCr-pCr-pUr-pCr-pGr-pGr-pGr-pUr-pCr-pCr-pCr-pAr-pGr-pG | Cyclic phosphorylation | 19 | 1.9 | 100 | 1.1 | 15.04 | 763.219 | -8 | 6111.8027 | 6114.38 | 6111.7911 | 1866831.62 |
| 3950-3968 | Ar-pCr-pCr-pUr-pCr-pGr-pGr-pGr-pUr-pCr-pCr-pCr-pAr-pGr-pG | Cyclic phosphorylation | 19 | 2.38 | 100 | 1.1 | 15.06 | 678.305 | -9 | 6111.8057 | 6114.5 | 6111.7911 | 1307964 |
| 3950-3968 | Ar-pCr-pCr-pUr-pCr-pGr-pGr-pGr-pUr-pCr-pCr-pCr-pAr-pGr-pG | Cyclic phosphorylation | 19 | 2.14 | 100 | 1.1 | 15.06 | 872.537 | -7 | 6111.8042 | 6114.47 | 6111.7911 | 883362.94 |
| 63-86 | Ur-pUr-pCr-pCr-pUr-pGr-pGr-pUr-pGr-pCr-pUr-pGr-pCr-pUr-pG | Cyclic phosphorylation | 24 | 2.97 | 98.7 | 1.2 | 15.21 | 764.285 | -10 | 7649.9189 | 7653.36 | 7649.8962 | 866756.31 |
| 63-86 | Ur-pUr-pCr-pCr-pUr-pGr-pGr-pUr-pGr-pCr-pUr-pGr-pCr-pUr-pG | Cyclic phosphorylation | 24 | 3.68 | 98.7 | 1.1 | 15.21 | 849.206 | -9 | 7649.9243 | 7653.43 | 7649.8962 | 433552.62 |
| 1952-1972 | Gr-pCr-pGr-pGr-pGr-pUr-pGr-pUr-pAr-pCr-pUr-pCr-pCr-pAr-pC | Cyclic phosphorylation | 21 | 3.5 | 100 | 1.2 | 15.25 | 970.982 | -7 | 6800.9258 | 6803.72 | 6800.9019 | 929804.06 |
| 1952-1972 | Gr-pCr-pGr-pGr-pGr-pUr-pGr-pUr-pAr-pCr-pUr-pCr-pCr-pAr-pC | Cyclic phosphorylation | 21 | 4.8 | 100 | 1 | 15.25 | 849.357 | -8 | 6800.9346 | 6803.97 | 6800.9019 | 2193556.5 |
| 1952-1972 | Gr-pCr-pGr-pGr-pGr-pUr-pGr-pUr-pAr-pCr-pUr-pCr-pCr-pAr-pC | Cyclic phosphorylation | 21 | 2.5 | 100 | 1 | 15.25 | 754.873 | -9 | 6800.9189 | 6803.73 | 6800.9019 | 2576827.75 |
| 1952-1972 | Gr-pCr-pGr-pGr-pGr-pUr-pGr-pUr-pAr-pCr-pUr-pCr-pCr-pAr-pC | Cyclic phosphorylation | 21 | 2.36 | 100 | 1 | 15.26 | 679.285 | -10 | 6800.918 | 6803.81 | 6800.9019 | 618069.19 |
| 3273-3291 | Ar-pAr-pUr-pUr-pUr-pCr-pAr-pCr-pCr-pAr-pCr-pCr-pGr-pCr-pU | Cyclic phosphorylation | 19 | 1.89 | 100 | 1.3 | 15.3 | 665.749 | -9 | 5999.8003 | 6002.39 | 5999.789 | 670847.19 |
| 3273-3291 | Ar-pAr-pUr-pUr-pUr-pCr-pAr-pCr-pCr-pAr-pCr-pCr-pGr-pCr-pU | Cyclic phosphorylation | 19 | 2.54 | 100 | 1 | 15.31 | 749.219 | -8 | 5999.8042 | 6002.38 | 5999.789 | 1528428.12 |
| 3273-3291 | Ar-pAr-pUr-pUr-pUr-pCr-pAr-pCr-pCr-pAr-pCr-pCr-pGr-pCr-pU | Cyclic phosphorylation | 19 | 3.03 | 100 | 1.2 | 15.32 | 856.394 | -7 | 5999.8071 | 6002.59 | 5999.789 | 759706.06 |
| 954-975 | Ur-pGr-pCr-pAr-pCr-pCr-pCr-pUr-pGr-pAr-pAr-pGr-pUr-pCr-pC | Cyclic phosphorylation | 22 | 1.76 | 99.9 | 1.3 | 15.45 | 995.265 | -7 | 6971.9102 | 6974.79 | 6971.8979 | 1319268.12 |
| 954-975 | Ur-pGr-pCr-pAr-pCr-pCr-pCr-pUr-pGr-pAr-pAr-pGr-pUr-pCr-pC | Cyclic phosphorylation | 22 | 2.39 | 99.9 | 1 | 15.46 | 773.984 | -9 | 6971.9146 | 6974.78 | 6971.8979 | 2304024 |
| 954-975 | Ur-pGr-pCr-pAr-pCr-pCr-pCr-pUr-pGr-pAr-pAr-pGr-pUr-pCr-pC | Cyclic phosphorylation | 22 | 1.76 | 99.9 | 1.1 | 15.46 | 870.731 | -8 | 6971.9102 | 6974.75 | 6971.8979 | 2881782.5 |
| 2661-2679 | Ar-pUr-pCr-pGr-pCr-pCr-pCr-pAr-pGr-pUr-pAr-pCr-pAr-pCr-pAr | Cyclic phosphorylation | 19 | 2.39 | 100 | 1.1 | 15.52 | 670.305 | -9 | 6039.8096 | 6042.31 | 6039.7951 | 1675422.12 |
| 2661-2679 | Ar-pUr-pCr-pGr-pCr-pCr-pCr-pAr-pGr-pUr-pAr-pCr-pAr-pCr-pAr | Cyclic phosphorylation | 19 | 0.05 | 100 | 1 | 15.52 | 754.219 | -8 | 6039.7954 | 6042.51 | 6039.7951 | 2198937.5 |
| 2661-2679 | Ar-pUr-pCr-pGr-pCr-pCr-pCr-pAr-pGr-pUr-pAr-pCr-pAr-pCr-pAr | Cyclic phosphorylation | 19 | 1.67 | 100 | 1.1 | 15.52 | 862.251 | -7 | 6039.8052 | 6042.28 | 6039.7951 | 908011.69 |
| 2170-2190 | Cr-pCr-pUr-pAr-pCr-pUr-pCr-pCr-pAr-pAr-pCr-pAr-pAr-pCr-pUr | Cyclic phosphorylation | 21 | 0.99 | 100 | 1.4 | 15.73 | 941.403 | -7 | 6594.8672 | 6597.66 | 6594.8606 | 6292366.5 |
| 2170-2190 | Cr-pCr-pUr-pAr-pCr-pUr-pCr-pCr-pAr-pAr-pCr-pAr-pAr-pCr-pUr | Cyclic phosphorylation | 21 | -0.64 | 100 | 1.8 | 15.73 | 1098.472 | -6 | 6594.8564 | 6597.97 | 6594.8606 | 1694880.62 |
| 2170-2190 | Cr-pCr-pUr-pAr-pCr-pUr-pCr-pCr-pAr-pAr-pCr-pAr-pAr-pCr-pUr | Cyclic phosphorylation | 21 | 1.14 | 100 | 1.2 | 15.73 | 823.726 | -8 | 6594.8682 | 6597.69 | 6594.8606 | 17372808 |
| 2170-2190 | Cr-pCr-pUr-pAr-pCr-pUr-pCr-pCr-pAr-pAr-pCr-pAr-pAr-pCr-pUr | Cyclic phosphorylation | 21 | 1.81 | 100 | 1 | 15.75 | 732.09 | -9 | 6594.8726 | 6597.7 | 6594.8606 | 12029280 |
| 2170-2190 | Cr-pCr-pUr-pAr-pCr-pUr-pCr-pCr-pAr-pAr-pCr-pAr-pAr-pCr-pUr | Cyclic phosphorylation | 21 | 2.33 | 100 | 1 | 15.75 | 658.78 | -10 | 6594.876 | 6597.59 | 6594.8606 | 5386947 |
| 2170-2190 | Cr-pCr-pUr-pAr-pCr-pUr-pCr-pCr-pAr-pAr-pCr-pAr-pAr-pCr-pUr | Cyclic phosphorylation | 21 | 1.44 | 100 | 1.3 | 15.75 | 1648.211 | -4 | 6594.8701 | 6597.61 | 6594.8606 | 1690153.25 |
| 2274-2295 | Ur-pAr-pCr-pAr-pUr-pCr-pUr-pGr-pCr-pGr-pGr-pCr-pGr-pAr-pU | Cyclic phosphorylation | 22 | 2.47 | 100 | 1 | 15.92 | 700.385 | -10 | 7011.9214 | 7015.22 | 7011.9041 | 3588971.75 |
| 2274-2295 | Ur-pAr-pCr-pAr-pUr-pCr-pUr-pGr-pCr-pGr-pGr-pCr-pGr-pAr-pU | Cyclic phosphorylation | 22 | 0.87 | 100 | 1 | 15.97 | 1001.124 | -7 | 7011.9102 | 7015.02 | 7011.9041 | 3626491.75 |
| 2274-2295 | Ur-pAr-pCr-pAr-pUr-pCr-pUr-pGr-pCr-pGr-pGr-pCr-pGr-pAr-pU | Cyclic phosphorylation | 22 | 0.87 | 100 | 1.3 | 15.98 | 875.857 | -8 | 7011.9102 | 7014.85 | 7011.9041 | 10904252 |
| 2274-2295 | Ur-pAr-pCr-pAr-pUr-pCr-pUr-pGr-pCr-pGr-pGr-pCr-pGr-pAr-pU | Cyclic phosphorylation | 22 | 1.36 | 100 | 1 | 15.99 | 778.428 | -9 | 7011.9136 | 7015.02 | 7011.9041 | 13697743 |
| 2170-2190 | Cr-pCr-pUr-pAr-pCr-pUr-pCr-pCr-pAr-pAr-pCr-pAr-pAr-pCr-pUr | Phosphorylation | 21 | 0.65 | 100 | 1 | 16 | 825.853 | -8 | 6612.8755 | 6615.84 | 6612.8712 | 3321992 |
| 2170-2190 | Cr-pCr-pUr-pAr-pCr-pUr-pCr-pCr-pAr-pAr-pCr-pAr-pAr-pCr-pUr | Phosphorylation | 21 | 1.09 | 100 | 1.2 | 16.02 | 943.976 | -7 | 6612.8784 | 6615.78 | 6612.8712 | 1796749 |
| 4059-4079 | Cr-pCr-pAr-pCr-pAr-pCr-pCr-pCr-pCr-pCr-pAr-pCr-pGr-pGr-pGr- | Cyclic phosphorylation | 21 | 0.79 | 100 | 1 | 16.09 | 840.991 | -8 | 6733.9756 | 6736.89 | 6733.9702 | 2025184.88 |
| 4059-4079 | Cr-pCr-pAr-pCr-pAr-pCr-pCr-pCr-pCr-pCr-pAr-pCr-pGr-pGr-pGr- | Cyclic phosphorylation | 21 | 0.65 | 100 | 1 | 16.09 | 747.435 | -9 | 6733.9746 | 6736.9 | 6733.9702 | 1648669.62 |
| 1038-1058 | Ur-pUr-pCr-pCr-pCr-pCr-pAr-pAr-pUr-pAr-pUr-pCr-pAr-pCr-pCr | Cyclic phosphorylation | 21 | 0.6 | 100 | 1.4 | 16.14 | 941.687 | -7 | 6595.8486 | 6598.73 | 6595.8447 | 3095120.25 |
| 1038-1058 | Ur-pUr-pCr-pCr-pCr-pCr-pAr-pAr-pUr-pAr-pUr-pCr-pAr-pCr-pCr | Cyclic phosphorylation | 21 | 1.57 | 100 | 1.2 | 16.14 | 823.724 | -8 | 6595.855 | 6598.69 | 6595.8447 | 9790873 |
| 1038-1058 | Ur-pUr-pCr-pCr-pCr-pCr-pAr-pAr-pUr-pAr-pUr-pCr-pAr-pCr-pCr | Cyclic phosphorylation | 21 | 1.71 | 100 | 1 | 16.14 | 732.199 | -9 | 6595.856 | 6598.68 | 6595.8447 | 19880472 |
| 1038-1058 | Ur-pUr-pCr-pCr-pCr-pCr-pAr-pAr-pUr-pAr-pUr-pCr-pAr-pCr-pCr | Cyclic phosphorylation | 21 | 0.9 | 100 | 1.6 | 16.14 | 1098.635 | -6 | 6595.8506 | 6598.94 | 6595.8447 | 1143480.25 |
| 1038-1058 | Ur-pUr-pCr-pCr-pCr-pCr-pAr-pAr-pUr-pAr-pUr-pCr-pAr-pCr-pCr | Cyclic phosphorylation | 21 | 2.08 | 100 | 1 | 16.16 | 658.779 | -10 | 6595.8584 | 6598.71 | 6595.8447 | 7046065.5 |
| 1038-1058 | Ur-pUr-pCr-pCr-pCr-pCr-pAr-pAr-pUr-pAr-pUr-pCr-pAr-pCr-pCr | Cyclic phosphorylation | 21 | 0.6 | 100 | 1.3 | 16.16 | 1648.457 | -4 | 6595.8486 | 6598.72 | 6595.8447 | 1316506.25 |
| 240-261 | Ar-pCr-pCr-pUr-pGr-pGr-pUr-pUr-pCr-pCr-pAr-pCr-pGr-pCr-pCr | Cyclic phosphorylation | 22 | 0.73 | 100 | 1.1 | 16.24 | 870.734 | -8 | 6970.9189 | 6973.86 | 6970.9139 | 751063.31 |
| 240-261 | Ar-pCr-pCr-pUr-pGr-pGr-pUr-pUr-pCr-pCr-pAr-pCr-pGr-pCr-pCr | Cyclic phosphorylation | 22 | 2.34 | 100 | 1 | 16.24 | 773.985 | -9 | 6970.9302 | 6974.08 | 6970.9139 | 1337597.12 |
| 2274-2295 | Ur-pAr-pCr-pAr-pUr-pCr-pUr-pGr-pCr-pGr-pGr-pCr-pGr-pAr-pU | Phosphorylation | 22 | 0.96 | 95.1 | 1 | 16.26 | 877.984 | -8 | 7029.9214 | 7033.14 | 7029.9146 | 792030.44 |
| 240-261 | Ar-pCr-pCr-pUr-pGr-pGr-pUr-pUr-pCr-pCr-pAr-pCr-pGr-pCr-pCr | Cyclic phosphorylation | 22 | 2.62 | 100 | 2 | 16.26 | 696.386 | -10 | 6970.9321 | 6973.8 | 6970.9139 | 599522.12 |
| 4059-4079 | Cr-pCr-pAr-pCr-pAr-pCr-pCr-pCr-pCr-pCr-pAr-pCr-pGr-pGr-pGr- | Phosphorylation | 21 | 0.96 | 100 | 1.2 | 16.31 | 963.849 | -7 | 6751.9873 | 6754.89 | 6751.9808 | 2107145.25 |
| 4059-4079 | Cr-pCr-pAr-pCr-pAr-pCr-pCr-pCr-pCr-pCr-pAr-pCr-pGr-pGr-pGr- | Phosphorylation | 21 | 1.18 | 100 | 1 | 16.33 | 843.242 | -8 | 6751.9888 | 6754.75 | 6751.9808 | 1994179.88 |
| 1038-1058 | Ur-pUr-pCr-pCr-pCr-pCr-pAr-pAr-pUr-pAr-pUr-pCr-pAr-pCr-pCr | Phosphorylation | 21 | 1.96 | 100 | 1 | 16.4 | 825.976 | -8 | 6613.8682 | 6616.65 | 6613.8552 | 7425752 |
| 1038-1058 | Ur-pUr-pCr-pCr-pCr-pCr-pAr-pAr-pUr-pAr-pUr-pCr-pAr-pCr-pCr | Phosphorylation | 21 | 1.37 | 100 | 1 | 16.4 | 734.201 | -9 | 6613.8643 | 6616.69 | 6613.8552 | 3815709.25 |
| 1038-1058 | Ur-pUr-pCr-pCr-pCr-pCr-pAr-pAr-pUr-pAr-pUr-pCr-pAr-pCr-pCr | Phosphorylation | 21 | 1.96 | 100 | 1.2 | 16.4 | 1101.805 | -6 | 6613.8682 | 6616.77 | 6613.8552 | 1220126.38 |
| 1038-1058 | Ur-pUr-pCr-pCr-pCr-pCr-pAr-pAr-pUr-pAr-pUr-pCr-pAr-pCr-pCr | Phosphorylation | 21 | 1.22 | 100 | 1.3 | 16.4 | 944.116 | -7 | 6613.8633 | 6616.68 | 6613.8552 | 4496687.5 |
| 1172-1194 | Cr-pUr-pUr-pCr-pAr-pGr-pCr-pAr-pCr-pCr-pUr-pUr-pCr-pAr-pA | Cyclic phosphorylation | 23 | 1.49 | 100 | 1.3 | 16.53 | 1042.413 | -7 | 7301.9453 | 7305.19 | 7301.9344 | 2134240.5 |
| 1172-1194 | Cr-pUr-pUr-pCr-pAr-pGr-pCr-pAr-pCr-pCr-pUr-pUr-pCr-pAr-pA | Cyclic phosphorylation | 23 | 1.42 | 100 | 1 | 16.53 | 810.653 | -9 | 7301.9448 | 7305.02 | 7301.9344 | 17483730 |
| 1172-1194 | Cr-pUr-pUr-pCr-pAr-pGr-pCr-pAr-pCr-pCr-pUr-pUr-pCr-pAr-pA | Cyclic phosphorylation | 23 | 1.89 | 100 | 1 | 16.53 | 729.487 | -10 | 7301.9482 | 7305 | 7301.9344 | 10105635 |
| 1172-1194 | Cr-pUr-pUr-pCr-pAr-pGr-pCr-pAr-pCr-pCr-pUr-pUr-pCr-pAr-pA | Cyclic phosphorylation | 23 | 2.09 | 100 | 1 | 16.53 | 663.17 | -11 | 7301.9497 | 7305.08 | 7301.9344 | 3591386 |
| 1172-1194 | Cr-pUr-pUr-pCr-pAr-pGr-pCr-pAr-pCr-pCr-pUr-pUr-pCr-pAr-pA | Cyclic phosphorylation | 23 | 1.02 | 100 | 1.3 | 16.53 | 912.111 | -8 | 7301.9419 | 7305.18 | 7301.9344 | 7675611.5 |
| 3304-3325 | Ar-pCr-pGr-pGr-pCr-pAr-pAr-pAr-pGr-pCr-pCr-pCr-pAr-pCr-pUr | Cyclic phosphorylation | 22 | 2.25 | 100 | 1.1 | 16.58 | 701.088 | -10 | 7018.9521 | 7021.94 | 7018.9364 | 2682707.5 |
| 3304-3325 | Ar-pCr-pGr-pGr-pCr-pAr-pAr-pAr-pGr-pCr-pCr-pCr-pAr-pCr-pUr | Cyclic phosphorylation | 22 | 3.71 | 100 | 1 | 16.58 | 779.21 | -9 | 7018.9624 | 7022.2 | 7018.9364 | 2816769.75 |
| 198-221 | Cr-pAr-pCr-pUr-pCr-pUr-pAr-pCr-pCr-pCr-pAr-pGr-pGr-pAr-pCr | Cyclic phosphorylation | 24 | 0.82 | 98.9 | 1 | 16.59 | 841.768 | -9 | 7582.9707 | 7586.45 | 7582.9645 | 2230560.5 |
| 2424-2445 | Ar-pCr-pCr-pCr-pCr-pUr-pCr-pCr-pUr-pAr-pUr-pCr-pAr-pAr-pGr | Cyclic phosphorylation | 22 | -0.02 | 100 | 1.3 | 16.6 | 992.981 | -7 | 6955.9028 | 6958.95 | 6955.903 | 1605085.62 |
| 2424-2445 | Ar-pCr-pCr-pCr-pCr-pUr-pCr-pCr-pUr-pAr-pUr-pCr-pAr-pAr-pGr | Cyclic phosphorylation | 22 | 1.1 | 100 | 1.2 | 16.62 | 868.858 | -8 | 6955.9106 | 6958.88 | 6955.903 | 4931871.5 |
| 2424-2445 | Ar-pCr-pCr-pCr-pCr-pUr-pCr-pCr-pUr-pAr-pUr-pCr-pAr-pAr-pGr | Cyclic phosphorylation | 22 | 1.66 | 100 | 1 | 16.62 | 772.206 | -9 | 6955.9146 | 6959.01 | 6955.903 | 8575012 |
| 2424-2445 | Ar-pCr-pCr-pCr-pCr-pUr-pCr-pCr-pUr-pAr-pUr-pCr-pAr-pAr-pGr | Cyclic phosphorylation | 22 | 1.8 | 100 | 1 | 16.62 | 694.784 | -10 | 6955.9155 | 6959.01 | 6955.903 | 4109515.5 |
| 421-441 | Cr-pCr-pAr-pCr-pCr-pAr-pAr-pCr-pGr-pUr-pGr-pGr-pUr-pCr-pAr | Cyclic phosphorylation | 21 | 2.11 | 99.2 | 1.2 | 16.83 | 841.486 | -8 | 6736.9365 | 6740 | 6736.9223 | 798121.81 |
| 421-441 | Cr-pCr-pAr-pCr-pCr-pAr-pAr-pCr-pGr-pUr-pGr-pGr-pUr-pCr-pAr | Cyclic phosphorylation | 21 | 2.55 | 99.2 | 1.2 | 16.83 | 747.764 | -9 | 6736.9395 | 6739.94 | 6736.9223 | 655135.69 |
| 421-441 | Cr-pCr-pAr-pCr-pCr-pAr-pAr-pCr-pGr-pUr-pGr-pGr-pUr-pCr-pAr | Phosphorylation | 21 | 2.57 | 100 | 1.2 | 16.96 | 843.736 | -8 | 6754.9502 | 6757.98 | 6754.9328 | 475423.81 |
| 1394-1414 | Cr-pAr-pAr-pCr-pUr-pAr-pCr-pAr-pAr-pUr-pUr-pAr-pCr-pCr-pU | Cyclic phosphorylation | 21 | 2.33 | 100 | 1.2 | 17.03 | 739.092 | -9 | 6658.8936 | 6661.76 | 6658.878 | 775160.31 |
| 1394-1414 | Cr-pAr-pAr-pCr-pUr-pAr-pCr-pAr-pAr-pUr-pUr-pAr-pCr-pCr-pU | Cyclic phosphorylation | 21 | 1.67 | 100 | 1.1 | 17.03 | 831.605 | -8 | 6658.8892 | 6661.97 | 6658.878 | 673599.31 |
| 2355-2375 | Ar-pCr-pAr-pGr-pGr-pGr-pAr-pUr-pCr-pGr-pCr-pCr-pGr-pUr-pG | Cyclic phosphorylation | 21 | 1.57 | 100 | 1 | 17.12 | 981.131 | -7 | 6871.9624 | 6875.02 | 6871.9516 | 1796739.12 |
| 2355-2375 | Ar-pCr-pAr-pGr-pGr-pGr-pAr-pUr-pCr-pGr-pCr-pCr-pGr-pUr-pG | Cyclic phosphorylation | 21 | 1.78 | 100 | 1 | 17.12 | 762.766 | -9 | 6871.9639 | 6874.87 | 6871.9516 | 11041843 |
| 2355-2375 | Ar-pCr-pAr-pGr-pGr-pGr-pAr-pUr-pCr-pGr-pCr-pCr-pGr-pUr-pG | Cyclic phosphorylation | 21 | 1.5 | 100 | 1 | 17.12 | 858.363 | -8 | 6871.9619 | 6874.84 | 6871.9516 | 5813041.5 |
| 2355-2375 | Ar-pCr-pAr-pGr-pGr-pGr-pAr-pUr-pCr-pGr-pCr-pCr-pGr-pUr-pG | Cyclic phosphorylation | 21 | 1.07 | 100 | 1 | 17.12 | 686.49 | -10 | 6871.959 | 6874.89 | 6871.9516 | 2438278.5 |
| 3112-3137 | Cr-pCr-pUr-pCr-pUr-pGr-pCr-pCr-pAr-pAr-pUr-pCr-pUr-pGr-pG | Cyclic phosphorylation | 26 | -0.09 | 98.9 | 1.3 | 17.19 | 687.169 | -12 | 8255.0957 | 8259.06 | 8255.0964 | 390123.25 |
| 3112-3137 | Cr-pCr-pUr-pCr-pUr-pGr-pCr-pCr-pAr-pAr-pUr-pCr-pUr-pGr-pG | Cyclic phosphorylation | 26 | 2.04 | 98.9 | 1 | 17.24 | 824.804 | -10 | 8255.1133 | 8258.98 | 8255.0964 | 1738121.88 |
| 2355-2375 | Ar-pCr-pAr-pGr-pGr-pGr-pAr-pUr-pCr-pGr-pCr-pCr-pGr-pUr-pG | Phosphorylation | 21 | 1.31 | 100 | 1 | 17.26 | 764.88 | -9 | 6889.9712 | 6892.97 | 6889.9622 | 1470264.38 |
| 2355-2375 | Ar-pCr-pAr-pGr-pGr-pGr-pAr-pUr-pCr-pGr-pCr-pCr-pGr-pUr-pG | Phosphorylation | 21 | 1.94 | 100 | 1 | 17.27 | 860.615 | -8 | 6889.9756 | 6893.08 | 6889.9622 | 2028396 |
| 1533-1557 | Ur-pCr-pCr-pUr-pAr-pCr-pGr-pGr-pCr-pUr-pUr-pUr-pCr-pAr-pG | Cyclic phosphorylation | 25 | 7.64 | 99.9 | 1.3 | 17.41 | 991.374 | -8 | 7936.0889 | 7939.37 | 7936.0282 | 2382184.75 |
| 1533-1557 | Ur-pCr-pCr-pUr-pAr-pCr-pGr-pGr-pCr-pUr-pUr-pUr-pCr-pAr-pG | Cyclic phosphorylation | 25 | 7.88 | 99.9 | 1 | 17.41 | 792.797 | -10 | 7936.0908 | 7939.42 | 7936.0282 | 6852628 |
| 1533-1557 | Ur-pCr-pCr-pUr-pAr-pCr-pGr-pGr-pCr-pUr-pUr-pUr-pCr-pAr-pG | Cyclic phosphorylation | 25 | 7.95 | 99.9 | 1 | 17.41 | 881.109 | -9 | 7936.0913 | 7939.36 | 7936.0282 | 6151695.5 |
| 1533-1557 | Ur-pCr-pCr-pUr-pAr-pCr-pGr-pGr-pCr-pUr-pUr-pUr-pCr-pAr-pG | Cyclic phosphorylation | 25 | 5.73 | 99.9 | 1 | 17.41 | 720.724 | -11 | 7936.0737 | 7939.39 | 7936.0282 | 5389683.5 |
| 1533-1557 | Ur-pCr-pCr-pUr-pAr-pCr-pGr-pGr-pCr-pUr-pUr-pUr-pCr-pAr-pG | Cyclic phosphorylation | 25 | 7.7 | 99.9 | 1.3 | 17.43 | 1133.143 | -7 | 7936.0894 | 7939.38 | 7936.0282 | 943872.5 |
| 2191-2215 | Cr-pUr-pAr-pUr-pCr-pCr-pCr-pCr-pAr-pCr-pCr-pAr-pAr-pCr-pUr- | Cyclic phosphorylation | 25 | 1.88 | 100 | 1 | 17.57 | 783.198 | -10 | 7839.0518 | 7842.48 | 7839.037 | 5700650.5 |
| 2191-2215 | Cr-pUr-pAr-pUr-pCr-pCr-pCr-pCr-pAr-pCr-pCr-pAr-pAr-pCr-pUr- | Cyclic phosphorylation | 25 | 1.88 | 100 | 1 | 17.57 | 711.906 | -11 | 7839.0518 | 7842.49 | 7839.037 | 5372669.5 |
| 2191-2215 | Cr-pUr-pAr-pUr-pCr-pCr-pCr-pCr-pAr-pCr-pCr-pAr-pAr-pCr-pUr- | Cyclic phosphorylation | 25 | 1.38 | 100 | 1 | 17.57 | 870.332 | -9 | 7839.0479 | 7842.45 | 7839.037 | 4871974 |
| 1845-1867 | Ar-pUr-pCr-pAr-pCr-pCr-pCr-pCr-pUr-pGr-pGr-pCr-pAr-pCr-pCr | Cyclic phosphorylation | 23 | 1.46 | 99.6 | 1 | 17.57 | 729.896 | -10 | 7306.0254 | 7309.24 | 7306.0147 | 2191786.5 |
| 1845-1867 | Ar-pUr-pCr-pAr-pCr-pCr-pCr-pCr-pUr-pGr-pGr-pCr-pAr-pCr-pCr | Cyclic phosphorylation | 23 | 2.2 | 99.6 | 1 | 17.58 | 810.996 | -9 | 7306.0308 | 7309.22 | 7306.0147 | 3172809.75 |
| 2191-2215 | Cr-pUr-pAr-pUr-pCr-pCr-pCr-pCr-pAr-pCr-pCr-pAr-pAr-pCr-pUr- | Cyclic phosphorylation | 25 | 1.82 | 100 | 1 | 17.58 | 979.249 | -8 | 7839.0513 | 7842.34 | 7839.037 | 1845415.25 |
| 1533-1557 | Ur-pCr-pCr-pUr-pAr-pCr-pGr-pGr-pCr-pUr-pUr-pUr-pCr-pAr-pG | Phosphorylation | 25 | 1.44 | 100 | 1 | 17.6 | 883.109 | -9 | 7954.0503 | 7957.56 | 7954.0388 | 3080203.25 |
| 1533-1557 | Ur-pCr-pCr-pUr-pAr-pCr-pGr-pGr-pCr-pUr-pUr-pUr-pCr-pAr-pG | Phosphorylation | 25 | 3.53 | 100 | 1 | 17.6 | 794.698 | -10 | 7954.0669 | 7957.69 | 7954.0388 | 1508402.12 |
| 1533-1557 | Ur-pCr-pCr-pUr-pAr-pCr-pGr-pGr-pCr-pUr-pUr-pUr-pCr-pAr-pG | Phosphorylation | 25 | 0.89 | 100 | 1.1 | 17.6 | 993.499 | -8 | 7954.0459 | 7957.65 | 7954.0388 | 2190458.75 |
| 2191-2215 | Cr-pUr-pAr-pUr-pCr-pCr-pCr-pCr-pAr-pCr-pCr-pAr-pAr-pCr-pUr- | Phosphorylation | 25 | 0.97 | 100 | 1.4 | 17.7 | 981.5 | -8 | 7857.0552 | 7860.52 | 7857.0476 | 7222769 |
| 2191-2215 | Cr-pUr-pAr-pUr-pCr-pCr-pCr-pCr-pAr-pCr-pCr-pAr-pAr-pCr-pUr- | Phosphorylation | 25 | 1.71 | 100 | 1 | 17.7 | 872.333 | -9 | 7857.061 | 7860.44 | 7857.0476 | 11214685 |
| 2191-2215 | Cr-pUr-pAr-pUr-pCr-pCr-pCr-pCr-pAr-pCr-pCr-pAr-pAr-pCr-pUr- | Phosphorylation | 25 | 1.34 | 100 | 1.2 | 17.72 | 1121.716 | -7 | 7857.0581 | 7860.48 | 7857.0476 | 2208331 |
| 2191-2215 | Cr-pUr-pAr-pUr-pCr-pCr-pCr-pCr-pAr-pCr-pCr-pAr-pAr-pCr-pUr- | Phosphorylation | 25 | 1.09 | 100 | 1 | 17.72 | 784.999 | -10 | 7857.0562 | 7860.4 | 7857.0476 | 4625923 |
| 13-38 | Ur-pAr-pUr-pUr-pCr-pUr-pUr-pCr-pUr-pGr-pGr-pUr-pCr-pCr-pC | Cyclic phosphorylation | 26 | 1.79 | 100 | 1.1 | 17.82 | 746.452 | -11 | 8219.041 | 8222.64 | 8219.0263 | 1822862 |
| 13-38 | Ur-pAr-pUr-pUr-pCr-pUr-pUr-pCr-pUr-pGr-pGr-pUr-pCr-pCr-pC | Cyclic phosphorylation | 26 | 2.26 | 100 | 1 | 17.82 | 912.554 | -9 | 8219.0449 | 8222.92 | 8219.0263 | 799460.19 |
| 13-38 | Ur-pAr-pUr-pUr-pCr-pUr-pUr-pCr-pUr-pGr-pGr-pUr-pCr-pCr-pC | Cyclic phosphorylation | 26 | 2.5 | 100 | 1 | 17.84 | 821.198 | -10 | 8219.0469 | 8222.71 | 8219.0263 | 1754617.88 |
| 1453-1475 | Ar-pCr-pAr-pUr-pCr-pUr-pCr-pCr-pAr-pCr-pCr-pGr-pAr-pGr-pAr | Cyclic phosphorylation | 23 | 2.55 | 100 | 1.4 | 18.03 | 1043.563 | -7 | 7308.9854 | 7312.14 | 7308.9667 | 445083 |
| 1453-1475 | Ar-pCr-pAr-pUr-pCr-pUr-pCr-pCr-pAr-pCr-pCr-pGr-pAr-pGr-pAr | Cyclic phosphorylation | 23 | 2.55 | 100 | 1.2 | 18.03 | 912.991 | -8 | 7308.9854 | 7312.2 | 7308.9667 | 1454391.38 |
| 1453-1475 | Ar-pCr-pAr-pUr-pCr-pUr-pCr-pCr-pAr-pCr-pCr-pGr-pAr-pGr-pAr | Cyclic phosphorylation | 23 | 2.41 | 100 | 1 | 18.05 | 811.435 | -9 | 7308.9844 | 7312.28 | 7308.9667 | 3103778.25 |
| 1453-1475 | Ar-pCr-pAr-pUr-pCr-pUr-pCr-pCr-pAr-pCr-pCr-pGr-pAr-pGr-pAr | Cyclic phosphorylation | 23 | 2.48 | 100 | 1 | 18.05 | 730.191 | -10 | 7308.9849 | 7312.14 | 7308.9667 | 2591542.5 |
| 1453-1475 | Ar-pCr-pAr-pUr-pCr-pUr-pCr-pCr-pAr-pCr-pCr-pGr-pAr-pGr-pAr | Cyclic phosphorylation | 23 | 2.41 | 100 | 1.5 | 18.05 | 663.719 | -11 | 7308.9844 | 7312.12 | 7308.9667 | 571785.31 |
| 1453-1475 | Ar-pCr-pAr-pUr-pCr-pUr-pCr-pCr-pAr-pCr-pCr-pGr-pAr-pGr-pAr | Phosphorylation | 23 | 2.7 | 100 | 1.4 | 18.15 | 1046.135 | -7 | 7326.9971 | 7330 | 7326.9773 | 1417826.38 |
| 1453-1475 | Ar-pCr-pAr-pUr-pCr-pUr-pCr-pCr-pAr-pCr-pCr-pGr-pAr-pGr-pAr | Phosphorylation | 23 | 1.5 | 100 | 1 | 18.17 | 915.242 | -8 | 7326.9883 | 7330.19 | 7326.9773 | 3249870 |
| 1453-1475 | Ar-pCr-pAr-pUr-pCr-pUr-pCr-pCr-pAr-pCr-pCr-pGr-pAr-pGr-pAr | Phosphorylation | 23 | 2.43 | 100 | 1 | 18.17 | 813.437 | -9 | 7326.9951 | 7330.48 | 7326.9773 | 2254136 |
| 897-920 | Ar-pAr-pCr-pGr-pGr-pCr-pAr-pCr-pCr-pAr-pUr-pCr-pAr-pCr-pCr- | Cyclic phosphorylation | 24 | 2.76 | 100 | 1 | 18.2 | 770.001 | -10 | 7707.0845 | 7710.35 | 7707.0632 | 1483752.12 |
| 897-920 | Ar-pAr-pCr-pGr-pGr-pCr-pAr-pCr-pCr-pAr-pUr-pCr-pAr-pCr-pCr- | Cyclic phosphorylation | 24 | 1.87 | 100 | 1.2 | 18.2 | 855.558 | -9 | 7707.0776 | 7710.45 | 7707.0632 | 1028933.25 |
| 897-920 | Ar-pAr-pCr-pGr-pGr-pCr-pAr-pCr-pCr-pAr-pUr-pCr-pAr-pCr-pCr- | Cyclic phosphorylation | 24 | 1.43 | 100 | 1.1 | 18.2 | 962.753 | -8 | 7707.0742 | 7710.58 | 7707.0632 | 537165.38 |
| 2440-2465 | Ar-pCr-pUr-pUr-pCr-pGr-pGr-pCr-pGr-pGr-pCr-pUr-pUr-pCr-pA | Cyclic phosphorylation | 26 | 6.6 | 99.9 | 1.1 | 18.66 | 829.201 | -10 | 8298.1094 | 8301.85 | 8298.0546 | 839613 |
| 2440-2465 | Ar-pCr-pUr-pUr-pCr-pGr-pGr-pCr-pGr-pGr-pCr-pUr-pUr-pCr-pA | Cyclic phosphorylation | 26 | 1.89 | 99.9 | 1.2 | 18.67 | 753.727 | -11 | 8298.0703 | 8301.78 | 8298.0546 | 815812.62 |
| 1172-1199 | Cr-pUr-pUr-pCr-pAr-pGr-pCr-pAr-pCr-pCr-pUr-pUr-pCr-pAr-pA | Cyclic phosphorylation | 28 | 0.35 | 99.8 | 1 | 18.67 | 812.735 | -11 | 8948.1465 | 8951.97 | 8948.1433 | 848800 |
| 1172-1199 | Cr-pUr-pUr-pCr-pAr-pGr-pCr-pAr-pCr-pCr-pUr-pUr-pCr-pAr-pA | Cyclic phosphorylation | 28 | 2.97 | 99.8 | 1.9 | 18.67 | 745.091 | -12 | 8948.1699 | 8952.15 | 8948.1433 | 480939.88 |
| 1172-1199 | Cr-pUr-pUr-pCr-pAr-pGr-pCr-pAr-pCr-pCr-pUr-pUr-pCr-pAr-pA | Cyclic phosphorylation | 28 | 1.12 | 99.8 | 1.3 | 18.67 | 894.108 | -10 | 8948.1533 | 8952.06 | 8948.1433 | 484826.31 |
| 2440-2465 | Ar-pCr-pUr-pUr-pCr-pGr-pGr-pCr-pGr-pGr-pCr-pUr-pUr-pCr-pA | Cyclic phosphorylation | 26 | -2.11 | 99.9 | 1.1 | 18.67 | 921.334 | -9 | 8298.0371 | 8301.48 | 8298.0546 | 425164.94 |
| 1410-1436 | Ur-pAr-pCr-pCr-pGr-pGr-pCr-pUr-pGr-pUr-pUr-pCr-pCr-pGr-pG | Cyclic phosphorylation | 27 | 1.86 | 100 | 1.1 | 18.81 | 785.096 | -11 | 8643.1182 | 8646.94 | 8643.102 | 632121.12 |
| 1410-1436 | Ur-pAr-pCr-pCr-pGr-pGr-pCr-pUr-pGr-pUr-pUr-pCr-pCr-pGr-pG | Cyclic phosphorylation | 27 | 2.99 | 100 | 1 | 18.81 | 863.605 | -10 | 8643.1279 | 8646.99 | 8643.102 | 545368.12 |
| 3141-3167 | Ur-pCr-pUr-pGr-pAr-pGr-pUr-pGr-pUr-pGr-pUr-pGr-pCr-pUr-p | Cyclic phosphorylation | 27 | 5.76 | 98.1 | 1.1 | 18.88 | 879.609 | -10 | 8802.1934 | 8805.95 | 8802.1426 | 696152.5 |

| | | | | | | | | | | | | | |
|---|---|---|---|---|---|---|---|---|---|---|---|---|---|
| 3141-3167 | Ur-pCr-pUr-pGr-pAr-pGr-pUr-pGr-pUr-pGr-pUr-pGr-pCr-pUr-p | Cyclic phosphorylation | 27 | 1.66 | 98.1 | 1 | 18.9 | 799.554 | -11 | 8802.1572 | 8806.02 | 8802.1426 | 820702.44 |
| 13-40 | Ur-pAr-pUr-pUr-pCr-pUr-pUr-pCr-pUr-pGr-pGr-pUr-pCr-pCr-p | Cyclic phosphorylation | 28 | 2.05 | 100 | 1 | 19 | 987.565 | -9 | 8893.1445 | 8897.21 | 8893.1263 | 2555462.5 |
| 13-40 | Ur-pAr-pUr-pUr-pCr-pUr-pUr-pCr-pUr-pGr-pGr-pUr-pCr-pCr-p | Cyclic phosphorylation | 28 | 1.94 | 100 | 1 | 19 | 740.338 | -12 | 8893.1436 | 8897.25 | 8893.1263 | 5661560.5 |
| 13-40 | Ur-pAr-pUr-pUr-pCr-pUr-pUr-pCr-pUr-pGr-pGr-pUr-pCr-pCr-p | Cyclic phosphorylation | 28 | 1.17 | 100 | 1 | 19 | 807.823 | -11 | 8893.1367 | 8897.15 | 8893.1263 | 9909419 |
| 13-40 | Ur-pAr-pUr-pUr-pCr-pUr-pUr-pCr-pUr-pGr-pGr-pUr-pCr-pCr-p | Cyclic phosphorylation | 28 | 0.84 | 100 | 1 | 19 | 888.607 | -10 | 8893.1338 | 8897.13 | 8893.1263 | 7046942 |
| 2191-2219 | Cr-pUr-pAr-pUr-pCr-pCr-pCr-pCr-pAr-pCr-pCr-pAr-pAr-pCr-pUr- | Cyclic phosphorylation | 29 | 1.15 | 93.2 | 1 | 19.07 | 830.195 | -11 | 9140.209 | 9144.41 | 9140.1985 | 413368.94 |
| 2191-2219 | Cr-pUr-pAr-pUr-pCr-pCr-pCr-pCr-pAr-pCr-pCr-pAr-pAr-pCr-pUr- | Cyclic phosphorylation | 29 | 4.14 | 93.2 | 1.2 | 19.07 | 760.928 | -12 | 9140.2363 | 9144.15 | 9140.1985 | 529511.69 |
| 13-40 | Ur-pAr-pUr-pUr-pCr-pUr-pUr-pCr-pUr-pGr-pGr-pUr-pCr-pCr-p | Cyclic phosphorylation | 28 | 1.72 | 100 | 1 | 19.1 | 807.733 | -11 | 8893.1416 | 8897.17 | 8893.1263 | 3621418.75 |
| 3914-3949 | Cr-pCr-pCr-pCr-pUr-pUr-pUr-pCr-pCr-pCr-pGr-pUr-pCr-pCr-pUr | Cyclic phosphorylation | 36 | 1.8 | 100 | 1.1 | 19.12 | 753.823 | -15 | 11317.4443 | 11322.08 | 11317.4239 | 215727.48 |
| 3914-3949 | Cr-pCr-pCr-pCr-pUr-pUr-pUr-pCr-pCr-pCr-pGr-pUr-pCr-pCr-pUr | Cyclic phosphorylation | 36 | 2.06 | 100 | 1 | 19.14 | 870.028 | -13 | 11317.4473 | 11322.68 | 11317.4239 | 1420999.75 |
| 3914-3949 | Cr-pCr-pCr-pCr-pUr-pUr-pUr-pCr-pCr-pCr-pGr-pUr-pCr-pCr-pUr | Cyclic phosphorylation | 36 | -0.27 | 100 | 1.1 | 19.14 | 942.531 | -12 | 11317.4209 | 11322.65 | 11317.4239 | 750740.56 |
| 2492-2519 | Cr-pAr-pAr-pGr-pCr-pGr-pGr-pAr-pGr-pCr-pUr-pUr-pCr-pAr-pU | Cyclic phosphorylation | 28 | 2.42 | 100 | 1 | 19.31 | 904.415 | -10 | 9050.2207 | 9054.31 | 9050.1988 | 1107070.25 |
| 2492-2519 | Cr-pAr-pAr-pGr-pCr-pGr-pGr-pAr-pGr-pCr-pUr-pUr-pCr-pAr-pU | Cyclic phosphorylation | 28 | 2.74 | 100 | 1 | 19.33 | 822.104 | -11 | 9050.2236 | 9054.26 | 9050.1988 | 1661720.75 |
| 2492-2519 | Cr-pAr-pAr-pGr-pCr-pGr-pGr-pAr-pGr-pCr-pUr-pUr-pCr-pAr-pU | Cyclic phosphorylation | 28 | 1.55 | 100 | 1.3 | 19.33 | 1005.017 | -9 | 9050.2129 | 9054.11 | 9050.1988 | 474859.81 |
| 2492-2519 | Cr-pAr-pAr-pGr-pCr-pGr-pGr-pAr-pGr-pCr-pUr-pUr-pCr-pAr-pU | Cyclic phosphorylation | 28 | 1.55 | 100 | 1.1 | 19.36 | 753.428 | -12 | 9050.2129 | 9054.28 | 9050.1988 | 1104604 |
| 2377-2400 | Ar-pCr-pAr-pAr-pGr-pAr-pAr-pCr-pAr-pCr-pCr-pCr-pAr-pAr-pGr | Cyclic phosphorylation | 24 | 1.15 | 93.8 | 1.3 | 19.59 | 777.303 | -10 | 7780.0898 | 7783.58 | 7780.0809 | 701396.75 |
| 2377-2400 | Ar-pCr-pAr-pAr-pGr-pAr-pAr-pCr-pAr-pCr-pCr-pCr-pAr-pAr-pGr | Cyclic phosphorylation | 24 | 1.9 | 93.8 | 1 | 19.6 | 863.67 | -9 | 7780.0957 | 7783.74 | 7780.0809 | 705698.38 |
| 385-411 | Ar-pCr-pAr-pGr-pCr-pAr-pAr-pGr-pAr-pCr-pCr-pCr-pAr-pGr-pAr | Cyclic phosphorylation | 27 | 0.73 | 99.6 | 1 | 19.78 | 791.286 | -11 | 8711.2061 | 8715.3 | 8711.1997 | 1319121 |
| 385-411 | Ar-pCr-pAr-pGr-pCr-pAr-pAr-pGr-pAr-pCr-pCr-pCr-pAr-pGr-pAr | Cyclic phosphorylation | 27 | 3.42 | 99.6 | 1 | 19.79 | 870.415 | -10 | 8711.2295 | 8714.95 | 8711.1997 | 1008309.81 |
| 736-763 | Ar-pUr-pCr-pUr-pGr-pCr-pCr-pCr-pAr-pUr-pCr-pGr-pGr-pCr-pA | Cyclic phosphorylation | 28 | 7.3 | 99.9 | 1.2 | 19.81 | 738.844 | -12 | 8874.2441 | 8878.74 | 8874.1793 | 1692133 |
| 736-763 | Ar-pUr-pCr-pUr-pGr-pCr-pCr-pCr-pAr-pUr-pCr-pGr-pGr-pCr-pA | Cyclic phosphorylation | 28 | 2.68 | 99.9 | 1.1 | 19.83 | 886.712 | -10 | 8874.2031 | 8878.42 | 8874.1793 | 2136640.5 |
| 345-372 | Ur-pCr-pCr-pAr-pAr-pCr-pAr-pUr-pCr-pAr-pUr-pCr-pAr-pGr-pAr | Cyclic phosphorylation | 28 | 1.15 | 100 | 1 | 19.83 | 894.91 | -10 | 8956.1699 | 8960.42 | 8956.1597 | 2403076.75 |
| 345-372 | Ur-pCr-pCr-pAr-pAr-pCr-pAr-pUr-pCr-pAr-pUr-pCr-pAr-pGr-pAr | Cyclic phosphorylation | 28 | -0.6 | 100 | 1 | 19.85 | 813.555 | -11 | 8956.1543 | 8960.27 | 8956.1597 | 3046913.25 |
| 345-372 | Ur-pCr-pCr-pAr-pAr-pCr-pAr-pUr-pCr-pAr-pUr-pCr-pAr-pGr-pAr | Cyclic phosphorylation | 28 | 2.56 | 100 | 1.2 | 19.85 | 745.675 | -12 | 8956.1826 | 8959.9 | 8956.1597 | 1985371.12 |
| 1561-1592 | Ur-pGr-pGr-pGr-pCr-pUr-pAr-pUr-pCr-pAr-pGr-pCr-pCr-pCr-pU | Cyclic phosphorylation | 32 | 5.37 | 99.8 | 1 | 20.05 | 863.438 | -12 | 10369.3789 | 10374.19 | 10369.3232 | 1277617 |
| 13-42 | Ur-pAr-pUr-pUr-pCr-pUr-pUr-pCr-pUr-pGr-pGr-pUr-pCr-pCr-p | Phosphorylation | 30 | -3.82 | 100 | 1 | 20.15 | 870.562 | -11 | 9585.2002 | 9587.83 | 9585.2368 | 391919.34 |
| 596-622 | Cr-pAr-pAr-pGr-pCr-pAr-pGr-pGr-pGr-pCr-pAr-pAr-pCr-pUr-pU | Cyclic phosphorylation | 27 | 1.46 | 100 | 1 | 20.29 | 793.378 | -11 | 8735.2236 | 8738.23 | 8735.2109 | 2642116 |
| 596-622 | Cr-pAr-pAr-pGr-pCr-pAr-pGr-pGr-pGr-pCr-pAr-pAr-pCr-pUr-pU | Cyclic phosphorylation | 27 | 0.56 | 100 | 1 | 20.31 | 872.916 | -10 | 8735.2158 | 8739.16 | 8735.2109 | 3626794 |
| 3104-3133 | Cr-pUr-pAr-pCr-pAr-pCr-pCr-pAr-pAr-pCr-pCr-pUr-pGr-pGr-pUr | Cyclic phosphorylation | 30 | -1.84 | 99 | 1 | 20.31 | 742.399 | -13 | 9660.2637 | 9664.46 | 9660.2814 | 3195276.25 |
| 596-622 | Cr-pAr-pAr-pGr-pCr-pAr-pGr-pGr-pGr-pCr-pAr-pAr-pCr-pUr-pU | Cyclic phosphorylation | 27 | 0.79 | 100 | 1.2 | 20.33 | 970.018 | -9 | 8735.2178 | 8738.42 | 8735.2109 | 1868535.25 |
| 3104-3133 | Cr-pUr-pAr-pCr-pAr-pCr-pCr-pAr-pAr-pCr-pCr-pUr-pGr-pGr-pUr | Cyclic phosphorylation | 30 | -1.43 | 99 | 1 | 20.33 | 804.266 | -12 | 9660.2676 | 9664.62 | 9660.2814 | 6523568 |
| 3104-3133 | Cr-pUr-pAr-pCr-pAr-pCr-pCr-pAr-pAr-pCr-pCr-pUr-pGr-pGr-pUr | Cyclic phosphorylation | 30 | -0.72 | 99 | 1.1 | 20.34 | 965.42 | -10 | 9660.2744 | 9664.67 | 9660.2814 | 2777803.25 |
| 3104-3133 | Cr-pUr-pAr-pCr-pAr-pCr-pCr-pAr-pAr-pCr-pCr-pUr-pGr-pGr-pUr | Cyclic phosphorylation | 30 | -0.72 | 99 | 1 | 20.34 | 877.564 | -11 | 9660.2744 | 9664.6 | 9660.2814 | 5210300 |
| 4120-4149 | Cr-pUr-pAr-pUr-pAr-pCr-pUr-pAr-pAr-pCr-pCr-pCr-pCr-pAr-pGr | Phosphorylation | 30 | 4.47 | 100 | 1.8 | 20.71 | 736.705 | -13 | 9586.2637 | 9589.74 | 9586.2208 | 253964.5 |
| 4120-4149 | Cr-pUr-pAr-pUr-pAr-pCr-pUr-pAr-pAr-pCr-pCr-pCr-pCr-pAr-pGr | Phosphorylation | 30 | 0.19 | 100 | 1 | 20.71 | 958.017 | -10 | 9586.2227 | 9590.5 | 9586.2208 | 2201642.75 |
| 4120-4149 | Cr-pUr-pAr-pUr-pAr-pCr-pUr-pAr-pAr-pCr-pCr-pCr-pCr-pAr-pGr | Phosphorylation | 30 | 4.17 | 100 | 1 | 20.71 | 870.924 | -11 | 9586.2607 | 9590.53 | 9586.2208 | 3184819 |
| 4120-4149 | Cr-pUr-pAr-pUr-pAr-pCr-pUr-pAr-pAr-pCr-pCr-pCr-pCr-pAr-pGr | Phosphorylation | 30 | 1.41 | 100 | 1 | 20.72 | 798.096 | -12 | 9586.2344 | 9590.39 | 9586.2208 | 1800110.62 |
| 596-624 | Cr-pAr-pAr-pGr-pCr-pAr-pGr-pGr-pGr-pCr-pAr-pAr-pCr-pUr-pU | Cyclic phosphorylation | 29 | 1.16 | 100 | 1 | 21.01 | 781.353 | -12 | 9385.3105 | 9389.55 | 9385.2996 | 2052977.75 |
| 461-494 | Cr-pAr-pAr-pCr-pGr-pAr-pCr-pCr-pCr-pCr-pUr-pUr-pCr-pCr-pUr | Phosphorylation | 34 | 3.8 | 100 | 1 | 21.01 | 832.106 | -13 | 10827.4863 | 10832.11 | 10827.4451 | 2610372 |
| 596-624 | Cr-pAr-pAr-pGr-pCr-pAr-pGr-pGr-pGr-pCr-pAr-pAr-pCr-pUr-pU | Cyclic phosphorylation | 29 | 1.89 | 100 | 1 | 21.03 | 852.567 | -11 | 9385.3174 | 9389.53 | 9385.2996 | 4012750.25 |
| 596-624 | Cr-pAr-pAr-pGr-pCr-pAr-pGr-pGr-pGr-pCr-pAr-pAr-pCr-pUr-pU | Cyclic phosphorylation | 29 | 1.99 | 100 | 1 | 21.03 | 937.926 | -10 | 9385.3184 | 9389.18 | 9385.2996 | 2244931 |
| 461-494 | Cr-pAr-pAr-pCr-pGr-pAr-pCr-pCr-pCr-pCr-pUr-pUr-pCr-pCr-pUr | Phosphorylation | 34 | 3.71 | 100 | 1 | 21.1 | 901.615 | -12 | 10827.4854 | 10832.35 | 10827.4451 | 8762376 |
| 461-494 | Cr-pAr-pAr-pCr-pGr-pAr-pCr-pCr-pCr-pCr-pUr-pUr-pCr-pCr-pUr | Phosphorylation | 34 | 3.53 | 100 | 1 | 21.12 | 983.763 | -11 | 10827.4834 | 10832.53 | 10827.4451 | 7439386.5 |
| 461-494 | Cr-pAr-pAr-pCr-pGr-pAr-pCr-pCr-pCr-pCr-pUr-pUr-pCr-pCr-pUr | Phosphorylation | 34 | 1.91 | 100 | 1.1 | 21.12 | 1082.24 | -10 | 10827.4658 | 10832.47 | 10827.4451 | 3522336.5 |
| 461-494 | Cr-pAr-pAr-pCr-pGr-pAr-pCr-pCr-pCr-pCr-pUr-pUr-pCr-pCr-pUr | Phosphorylation | 34 | 2.09 | 100 | 1 | 21.12 | 832.26 | -13 | 10827.4678 | 10832.7 | 10827.4451 | 4271258 |
| 116-145 | Ar-pAr-pCr-pAr-pCr-pAr-pGr-pCr-pUr-pGr-pCr-pCr-pUr-pCr-pCr | Cyclic phosphorylation | 30 | 1.9 | 100 | 1 | 21.27 | 954.928 | -10 | 9554.3457 | 9558.75 | 9554.3276 | 916417.12 |
| 116-145 | Ar-pAr-pCr-pAr-pCr-pAr-pGr-pCr-pUr-pGr-pCr-pCr-pUr-pCr-pCr | Cyclic phosphorylation | 30 | 1.9 | 100 | 1 | 21.29 | 867.934 | -11 | 9554.3457 | 9558.65 | 9554.3276 | 1162951.75 |
| 116-145 | Ar-pAr-pCr-pAr-pCr-pAr-pGr-pCr-pUr-pGr-pCr-pCr-pUr-pCr-pCr | Phosphorylation | 30 | 2.73 | 100 | 1 | 21.32 | 956.629 | -10 | 9572.3643 | 9576.68 | 9572.3381 | 931191.5 |
| 116-145 | Ar-pAr-pCr-pAr-pCr-pAr-pGr-pCr-pUr-pGr-pCr-pCr-pUr-pCr-pCr | Phosphorylation | 30 | 2.73 | 100 | 1.2 | 21.32 | 1063.034 | -9 | 9572.3643 | 9576.68 | 9572.3381 | 723462.69 |
| 116-145 | Ar-pAr-pCr-pAr-pCr-pAr-pGr-pCr-pUr-pGr-pCr-pCr-pUr-pCr-pCr | Phosphorylation | 30 | 5.18 | 100 | 1.2 | 21.32 | 869.571 | -11 | 9572.3877 | 9576.98 | 9572.3381 | 572122.81 |
| 1845-1874 | Ar-pUr-pCr-pAr-pCr-pCr-pCr-pCr-pUr-pGr-pGr-pCr-pAr-pCr-pCr | Cyclic phosphorylation | 30 | 3.33 | 99.7 | 1.2 | 21.51 | 795.439 | -12 | 9554.3594 | 9558.79 | 9554.3276 | 1053591 |
| 1845-1874 | Ar-pUr-pCr-pAr-pCr-pCr-pCr-pCr-pUr-pGr-pGr-pCr-pAr-pCr-pCr | Cyclic phosphorylation | 30 | -0.55 | 99.7 | 1.1 | 21.51 | 868.027 | -11 | 9554.3223 | 9558.67 | 9554.3276 | 298595.91 |
| 1453-1483 | Ar-pCr-pAr-pUr-pCr-pUr-pCr-pCr-pAr-pCr-pCr-pGr-pAr-pGr-pAr | Cyclic phosphorylation | 31 | 0.31 | 94.2 | 1.2 | 21.6 | 827.022 | -12 | 9933.3359 | 9937.89 | 9933.3329 | 845800.5 |
| 1453-1483 | Ar-pCr-pAr-pUr-pCr-pUr-pCr-pCr-pAr-pCr-pCr-pGr-pAr-pGr-pAr | Cyclic phosphorylation | 31 | 1.59 | 94.2 | 1.1 | 21.6 | 763.328 | -13 | 9933.3486 | 9937.8 | 9933.3329 | 772106.88 |
| 1453-1483 | Ar-pCr-pAr-pUr-pCr-pUr-pCr-pCr-pAr-pCr-pCr-pGr-pAr-pGr-pAr | Cyclic phosphorylation | 31 | 2.67 | 94.2 | 1.1 | 21.62 | 902.389 | -11 | 9933.3594 | 9937.64 | 9933.3329 | 714554.5 |
| 414-441 | Ar-pAr-pCr-pAr-pAr-pCr-pGr-pCr-pCr-pAr-pCr-pCr-pAr-pAr-pCr- | Phosphorylation | 28 | -0.81 | 100 | 1.2 | 22.2 | 1127.78 | -8 | 9026.2656 | 9030.06 | 9026.2729 | 2223844.25 |
| 414-441 | Ar-pAr-pCr-pAr-pAr-pCr-pGr-pCr-pCr-pAr-pCr-pCr-pAr-pAr-pCr- | Phosphorylation | 28 | 1.79 | 100 | 1.1 | 22.2 | 1002.247 | -9 | 9026.2891 | 9030.22 | 9026.2729 | 4620815.5 |
| 414-441 | Ar-pAr-pCr-pAr-pAr-pCr-pGr-pCr-pCr-pAr-pCr-pCr-pAr-pAr-pCr- | Phosphorylation | 28 | 0.71 | 100 | 1 | 22.21 | 902.122 | -10 | 9026.2793 | 9030.51 | 9026.2729 | 3947247 |
| 414-441 | Ar-pAr-pCr-pAr-pAr-pCr-pGr-pCr-pCr-pAr-pCr-pCr-pAr-pAr-pCr- | Cyclic phosphorylation | 28 | 0.58 | 100 | 1 | 22.24 | 900.121 | -10 | 9008.2676 | 9012.34 | 9008.2624 | 6990708 |
| 414-441 | Ar-pAr-pCr-pAr-pAr-pCr-pGr-pCr-pCr-pAr-pCr-pCr-pAr-pAr-pCr- | Cyclic phosphorylation | 28 | 0.14 | 100 | 1 | 22.24 | 1000.358 | -9 | 9008.2637 | 9012.33 | 9008.2624 | 3953914.5 |
| 414-441 | Ar-pAr-pCr-pAr-pAr-pCr-pGr-pCr-pCr-pAr-pCr-pCr-pAr-pAr-pCr- | Cyclic phosphorylation | 28 | 2.42 | 100 | 1 | 22.25 | 818.2 | -11 | 9008.2842 | 9012.21 | 9008.2624 | 5885061.5 |
| 414-441 | Ar-pAr-pCr-pAr-pAr-pCr-pGr-pCr-pCr-pAr-pCr-pCr-pAr-pAr-pCr- | Cyclic phosphorylation | 28 | 1.77 | 100 | 1.6 | 22.25 | 1125.402 | -8 | 9008.2783 | 9012.16 | 9008.2624 | 1239636.75 |
| 2377-2406 | Ar-pCr-pAr-pAr-pGr-pAr-pAr-pCr-pAr-pCr-pCr-pCr-pAr-pAr-pGr | Cyclic phosphorylation | 30 | 3.34 | 100 | 1.2 | 22.51 | 745.253 | -13 | 9698.3896 | 9702.42 | 9698.3572 | 1594685.75 |
| 2377-2406 | Ar-pCr-pAr-pAr-pGr-pAr-pAr-pCr-pAr-pCr-pCr-pCr-pAr-pAr-pGr | Cyclic phosphorylation | 30 | 3.04 | 100 | 1 | 22.51 | 807.525 | -12 | 9698.3867 | 9702.62 | 9698.3572 | 2642235 |
| 2377-2406 | Ar-pCr-pAr-pAr-pGr-pAr-pAr-pCr-pAr-pCr-pCr-pCr-pAr-pAr-pGr | Cyclic phosphorylation | 30 | -1.09 | 100 | 1 | 22.53 | 881.119 | -11 | 9698.3467 | 9702.95 | 9698.3572 | 1828285.75 |
| 2377-2406 | Ar-pCr-pAr-pAr-pGr-pAr-pAr-pCr-pAr-pCr-pCr-pCr-pAr-pAr-pGr | Cyclic phosphorylation | 30 | 0.02 | 100 | 1.2 | 22.53 | 969.332 | -10 | 9698.3574 | 9702.89 | 9698.3572 | 1051852 |
| 2343-2375 | Ar-pAr-pUr-pAr-pGr-pAr-pGr-pCr-pCr-pCr-pUr-pGr-pAr-pCr-pA | Cyclic phosphorylation | 33 | 4.64 | 98.5 | 1 | 23.88 | 826.34 | -13 | 10750.5283 | 10755.36 | 10750.4785 | 2288560 |
| 2343-2375 | Ar-pAr-pUr-pAr-pGr-pAr-pGr-pCr-pCr-pCr-pUr-pGr-pAr-pCr-pA | Cyclic phosphorylation | 33 | 4.45 | 98.5 | 1.1 | 23.88 | 767.244 | -14 | 10750.5264 | 10755.17 | 10750.4785 | 1616083.75 |
| 2343-2375 | Ar-pAr-pUr-pAr-pGr-pAr-pGr-pCr-pCr-pCr-pUr-pGr-pAr-pCr-pA | Cyclic phosphorylation | 33 | 1.82 | 98.5 | 1 | 23.9 | 895.202 | -12 | 10750.498 | 10755.46 | 10750.4785 | 1929161.62 |
| 4173-4204 | Cr-pAr-pAr-pAr-pAr-pAr-pAr-pAr-pAr-pAr-pAr-pAr-pAr-pAr-pAr | Phosphorylation | 32 | 2.59 | 100 | 1 | 33.49 | 1170.515 | -9 | 10539.7021 | 10544.53 | 10539.6749 | 1284222 |
| 4173-4204 | Cr-pAr-pAr-pAr-pAr-pAr-pAr-pAr-pAr-pAr-pAr-pAr-pAr-pAr-pAr | Phosphorylation | 32 | 0.73 | 100 | 1 | 33.49 | 1053.363 | -10 | 10539.6826 | 10544.38 | 10539.6749 | 2757186.25 |
| 4173-4204 | Cr-pAr-pAr-pAr-pAr-pAr-pAr-pAr-pAr-pAr-pAr-pAr-pAr-pAr-pAr | Cyclic phosphorylation | 32 | 4.99 | 100 | 1.9 | 33.59 | 808.739 | -13 | 10521.7168 | 10526.43 | 10521.6643 | 824361.56 |
| 4173-4204 | Cr-pAr-pAr-pAr-pAr-pAr-pAr-pAr-pAr-pAr-pAr-pAr-pAr-pAr-pAr | Cyclic phosphorylation | 32 | 8.61 | 100 | 1 | 33.59 | 876.218 | -12 | 10521.7549 | 10526.38 | 10521.6643 | 2671393.25 |
| 4173-4204 | Cr-pAr-pAr-pAr-pAr-pAr-pAr-pAr-pAr-pAr-pAr-pAr-pAr-pAr-pAr | Cyclic phosphorylation | 32 | -1.32 | 100 | 1 | 33.61 | 1051.562 | -10 | 10521.6504 | 10526.44 | 10521.6643 | 1508548.88 |

| Positions | Oligo Sequence | 3' Termini | Seq Length | Δ ppm | Conf. Score | Best ASR | RT | M/Z | Charge St. | Mono Mass Exp. | Avg Mass Exp. | Mono Mass Theo. | MS Area |
|---|---|---|---|---|---|---|---|---|---|---|---|---|---|
| 3175-3181 | Ar-pCr-pUr-pUr-pUr-pUr-pGr | Cyclic phosphorylation | 7 | -0.78 | 98.3 | 1 | 4.28 | 550.054 | -4 | 2203.2407 | 2204.21 | 2203.2425 | 1089182.75 |
| 3175-3181 | Ar-pCr-pUr-pUr-pUr-pUr-pGr | Cyclic phosphorylation | 7 | -1.01 | 98.3 | 1 | 4.28 | 733.741 | -3 | 2203.2402 | 2204.17 | 2203.2425 | 909654.62 |
| 2808-2813 | Ar-pAr-pCr-pCr-pAr-pGr | Cyclic phosphorylation | 6 | -0.17 | 94.7 | 1 | 4.38 | 646.422 | -3 | 1942.2872 | 1943.17 | 1942.2876 | 3256721.25 |
| 639-645 | Ar-pAr-pCr-pAr-pUr-pCr-pGr | Cyclic phosphorylation | 7 | -0.82 | 100 | 1 | 4.64 | 561.07 | -4 | 2248.311 | 2249.05 | 2248.3129 | 3712597.5 |
| 639-645 | Ar-pAr-pCr-pAr-pUr-pCr-pGr | Cyclic phosphorylation | 7 | -1.03 | 100 | 1 | 4.64 | 748.43 | -3 | 2248.3105 | 2249.22 | 2248.3129 | 3258482.5 |
| 639-645 | Ar-pAr-pCr-pAr-pUr-pCr-pGr | Cyclic phosphorylation | 7 | 0.05 | 100 | 1 | 4.64 | 1123.149 | -2 | 2248.313 | 2249.26 | 2248.3129 | 4144379 |
| 3007-3015 | Ar-pCr-pCr-pCr-pUr-pCr-pCr-pUr-pGr | Cyclic phosphorylation | 9 | 0.07 | 99.8 | 1 | 4.81 | 701.832 | -4 | 2811.3572 | 2812.44 | 2811.357 | 1397027.5 |
| 3007-3015 | Ar-pCr-pCr-pCr-pUr-pCr-pCr-pUr-pGr | Cyclic phosphorylation | 9 | 0.41 | 99.8 | 1 | 4.81 | 936.446 | -3 | 2811.3582 | 2812.47 | 2811.357 | 723837.75 |
| 3007-3015 | Ar-pCr-pCr-pCr-pUr-pCr-pCr-pUr-pGr | Cyclic phosphorylation | 9 | 0.33 | 99.8 | 1 | 4.82 | 1405.173 | -2 | 2811.3579 | 2812.45 | 2811.357 | 744693.62 |
| 3007-3015 | Ar-pCr-pCr-pCr-pUr-pCr-pCr-pUr-pGr | Cyclic phosphorylation | 9 | 0.24 | 99.8 | 1 | 4.86 | 561.465 | -5 | 2811.3577 | 2812.47 | 2811.357 | 1127758.62 |
| 116-122 | Ar-pAr-pCr-pAr-pCr-pAr-pGr | Cyclic phosphorylation | 7 | 0.54 | 96.6 | 1 | 5.78 | 756.107 | -3 | 2271.3413 | 2272.27 | 2271.3401 | 543787.5 |
| 116-122 | Ar-pAr-pCr-pAr-pCr-pAr-pGr | Cyclic phosphorylation | 7 | 0.64 | 96.6 | 1 | 5.82 | 1134.663 | -2 | 2271.3416 | 2272.28 | 2271.3401 | 864430.19 |
| 3914-3924 | Cr-pCr-pCr-pCr-pUr-pUr-pUr-pCr-pCr-pCr-pGr | Cyclic phosphorylation | 11 | 1.58 | 100 | 1 | 6.16 | 1698.703 | -2 | 3398.4177 | 3399.85 | 3398.4123 | 228747.67 |
| 3914-3924 | Cr-pCr-pCr-pCr-pUr-pUr-pUr-pCr-pCr-pCr-pGr | Cyclic phosphorylation | 11 | 1.01 | 100 | 1 | 6.17 | 678.876 | -5 | 3398.4158 | 3399.8 | 3398.4123 | 566360.12 |
| 3914-3924 | Cr-pCr-pCr-pCr-pUr-pUr-pUr-pCr-pCr-pCr-pGr | Cyclic phosphorylation | 11 | 1.51 | 100 | 1 | 6.17 | 1132.129 | -3 | 3398.4175 | 3399.67 | 3398.4123 | 258103.41 |
| 3914-3924 | Cr-pCr-pCr-pCr-pUr-pUr-pUr-pCr-pCr-pCr-pGr | Cyclic phosphorylation | 11 | 1.65 | 100 | 1 | 6.17 | 565.562 | -6 | 3398.418 | 3399.91 | 3398.4123 | 363120.38 |
| 3914-3924 | Cr-pCr-pCr-pCr-pUr-pUr-pUr-pCr-pCr-pCr-pGr | Cyclic phosphorylation | 11 | 1.37 | 100 | 1 | 6.19 | 848.848 | -4 | 3398.417 | 3399.81 | 3398.4123 | 311910.91 |
| 660-667 | Ar-pUr-pCr-pUr-pAr-pCr-pAr-pGr | Cyclic phosphorylation | 8 | -0.88 | 95.1 | 1 | 6.51 | 509.86 | -5 | 2554.3359 | 2555.32 | 2554.3382 | 4066365.75 |
| 660-667 | Ar-pUr-pCr-pUr-pAr-pCr-pAr-pGr | Cyclic phosphorylation | 8 | 0.08 | 95.1 | 1 | 6.51 | 850.439 | -3 | 2554.3384 | 2555.37 | 2554.3382 | 2379712.75 |
| 660-667 | Ar-pUr-pCr-pUr-pAr-pCr-pAr-pGr | Cyclic phosphorylation | 8 | -0.97 | 95.1 | 1 | 6.51 | 637.577 | -4 | 2554.3357 | 2555.37 | 2554.3382 | 6030426 |
| 660-667 | Ar-pUr-pCr-pUr-pAr-pCr-pAr-pGr | Cyclic phosphorylation | 8 | -0.78 | 95.1 | 1 | 6.51 | 1276.161 | -2 | 2554.3362 | 2555.37 | 2554.3382 | 6116844.5 |
| 3898-3906 | Cr-pAr-pCr-pGr-pCr-pAr-pAr-pUr-pGr | Cyclic phosphorylation | 9 | 0.76 | 99.9 | 1 | 6.62 | 578.874 | -5 | 2898.4038 | 2899.51 | 2898.4016 | 902947.62 |
| 3898-3906 | Cr-pAr-pCr-pGr-pCr-pAr-pAr-pUr-pGr | Cyclic phosphorylation | 9 | 0.76 | 99.9 | 1 | 6.63 | 1448.696 | -2 | 2898.4038 | 2899.55 | 2898.4016 | 814683.69 |
| 1336-1344 | Ar-pCr-pUr-pUr-pCr-pAr-pCr-pCr-pGr | Cyclic phosphorylation | 9 | -0.28 | 99.9 | 1 | 6.67 | 566.267 | -5 | 2835.3674 | 2836.48 | 2835.3682 | 4575911 |
| 1336-1344 | Ar-pCr-pUr-pUr-pCr-pAr-pCr-pCr-pGr | Cyclic phosphorylation | 9 | 0.24 | 99.9 | 1 | 6.68 | 1417.178 | -2 | 2835.3689 | 2836.48 | 2835.3682 | 3776220.75 |
| 1336-1344 | Ar-pCr-pUr-pUr-pCr-pAr-pCr-pCr-pGr | Cyclic phosphorylation | 9 | -0.37 | 99.9 | 1 | 6.68 | 708.085 | -4 | 2835.3672 | 2836.49 | 2835.3682 | 4258754.5 |
| 1336-1344 | Ar-pCr-pUr-pUr-pCr-pAr-pCr-pCr-pGr | Cyclic phosphorylation | 9 | 0.49 | 99.9 | 1 | 6.69 | 944.45 | -3 | 2835.3696 | 2836.5 | 2835.3682 | 1867529 |
| 306-315 | Cr-pCr-pCr-pUr-pUr-pCr-pAr-pAr-pCr-pGr | Cyclic phosphorylation | 10 | 0.52 | 100 | 1 | 6.75 | 627.275 | -5 | 3140.4111 | 3141.62 | 3140.4095 | 1603350 |
| 306-315 | Cr-pCr-pCr-pUr-pUr-pCr-pAr-pAr-pCr-pGr | Cyclic phosphorylation | 10 | 0.36 | 100 | 1 | 6.75 | 784.346 | -4 | 3140.4106 | 3141.63 | 3140.4095 | 1116985.88 |
| 13-22 | Ur-pAr-pUr-pUr-pCr-pUr-pUr-pCr-pUr-pGr | Cyclic phosphorylation | 10 | 0.12 | 100 | 1 | 6.87 | 623.26 | -5 | 3120.3347 | 3121.68 | 3120.3343 | 1964578.25 |
| 13-22 | Ur-pAr-pUr-pUr-pCr-pUr-pUr-pCr-pUr-pGr | Cyclic phosphorylation | 10 | 1.06 | 100 | 1 | 6.87 | 1039.439 | -3 | 3120.3376 | 3121.72 | 3120.3343 | 513843.53 |
| 13-22 | Ur-pAr-pUr-pUr-pCr-pUr-pUr-pCr-pUr-pGr | Cyclic phosphorylation | 10 | 0.9 | 100 | 1 | 6.87 | 779.328 | -4 | 3120.3372 | 3121.58 | 3120.3343 | 713358.75 |
| 13-22 | Ur-pAr-pUr-pUr-pCr-pUr-pUr-pCr-pUr-pGr | Cyclic phosphorylation | 10 | 0.67 | 100 | 1 | 6.87 | 1559.662 | -2 | 3120.3364 | 3121.53 | 3120.3343 | 764206.12 |
| 13-22 | Ur-pAr-pUr-pUr-pCr-pUr-pUr-pCr-pUr-pGr | Cyclic phosphorylation | 10 | 0.82 | 100 | 1 | 6.88 | 519.216 | -6 | 3120.3369 | 3121.53 | 3120.3343 | 768162.31 |
| 2300-2309 | Cr-pUr-pCr-pCr-pAr-pAr-pCr-pCr-pUr-pGr | Cyclic phosphorylation | 10 | 1.14 | 94 | 1 | 7.01 | 784.347 | -4 | 3140.4131 | 3141.62 | 3140.4095 | 81411.35 |
| 292-300 | Ar-pCr-pAr-pAr-pCr-pCr-pCr-pCr-pGr | Cyclic phosphorylation | 9 | 0.92 | 100 | 1 | 7.16 | 951.798 | -3 | 2857.4141 | 2858.54 | 2857.4114 | 1428444.5 |
| 292-300 | Ar-pCr-pAr-pAr-pCr-pCr-pCr-pCr-pGr | Cyclic phosphorylation | 9 | -0.19 | 100 | 1 | 7.16 | 570.675 | -5 | 2857.4109 | 2858.52 | 2857.4114 | 3326617.5 |
| 292-300 | Ar-pCr-pAr-pAr-pCr-pCr-pCr-pCr-pGr | Cyclic phosphorylation | 9 | 0.32 | 100 | 1 | 7.17 | 1428.2 | -2 | 2857.4124 | 2858.54 | 2857.4114 | 2777387.25 |
| 292-300 | Ar-pCr-pAr-pAr-pCr-pCr-pCr-pCr-pGr | Cyclic phosphorylation | 9 | -0.36 | 100 | 1 | 7.18 | 713.596 | -4 | 2857.4104 | 2858.56 | 2857.4114 | 3431611 |
| 3258-3267 | Cr-pCr-pCr-pGr-pCr-pUr-pCr-pAr-pAr-pGr | Cyclic phosphorylation | 10 | 0.92 | 99.1 | 1 | 7.3 | 635.08 | -5 | 3179.4346 | 3180.66 | 3179.4316 | 1113490 |
| 3258-3267 | Cr-pCr-pCr-pGr-pCr-pUr-pCr-pAr-pAr-pGr | Cyclic phosphorylation | 10 | 1.23 | 99.1 | 1 | 7.3 | 794.102 | -4 | 3179.4355 | 3180.82 | 3179.4316 | 512408.31 |
| 4040-4048 | Cr-pUr-pCr-pAr-pAr-pAr-pAr-pCr-pGr | Cyclic phosphorylation | 9 | 0.36 | 100 | 1 | 7.3 | 719.845 | -4 | 2882.4077 | 2883.54 | 2882.4067 | 1367298.25 |
| 4040-4048 | Cr-pUr-pCr-pAr-pAr-pAr-pAr-pCr-pGr | Cyclic phosphorylation | 9 | 0.19 | 100 | 1 | 7.3 | 575.675 | -5 | 2882.4072 | 2883.54 | 2882.4067 | 1946394.5 |
| 4040-4048 | Cr-pUr-pCr-pAr-pAr-pAr-pAr-pCr-pGr | Cyclic phosphorylation | 9 | 0.87 | 100 | 1 | 7.3 | 960.13 | -3 | 2882.4092 | 2883.56 | 2882.4067 | 674790.88 |
| 4040-4048 | Cr-pUr-pCr-pAr-pAr-pAr-pAr-pCr-pGr | Cyclic phosphorylation | 9 | 0.36 | 100 | 1 | 7.3 | 1440.698 | -2 | 2882.4077 | 2883.53 | 2882.4067 | 1607474 |
| 3191-3200 | Cr-pUr-pAr-pCr-pCr-pAr-pCr-pCr-pUr-pGr | Cyclic phosphorylation | 10 | 0.75 | 100 | 1 | 7.39 | 522.562 | -6 | 3140.4119 | 3141.73 | 3140.4095 | 1071581.75 |
| 3191-3200 | Cr-pUr-pAr-pCr-pCr-pAr-pCr-pCr-pUr-pGr | Cyclic phosphorylation | 10 | 0.13 | 100 | 1 | 7.39 | 627.275 | -5 | 3140.4099 | 3141.6 | 3140.4095 | 2666541.5 |
| 3191-3200 | Cr-pUr-pAr-pCr-pCr-pAr-pCr-pCr-pUr-pGr | Cyclic phosphorylation | 10 | 1.22 | 100 | 1 | 7.39 | 784.346 | -4 | 3140.4133 | 3141.73 | 3140.4095 | 937404.88 |
| 3191-3200 | Cr-pUr-pAr-pCr-pCr-pAr-pCr-pCr-pUr-pGr | Cyclic phosphorylation | 10 | 1.22 | 100 | 1 | 7.39 | 1046.131 | -3 | 3140.4133 | 3141.77 | 3140.4095 | 767708.75 |
| 1281-1290 | Ar-pUr-pUr-pGr-pCr-pCr-pCr-pCr-pUr-pGr | Cyclic phosphorylation | 10 | 1.23 | 92.8 | 1 | 7.56 | 630.672 | -5 | 3157.3923 | 3158.75 | 3157.3884 | 666442.88 |
| 3516-3524 | Ur-pAr-pCr-pUr-pUr-pUr-pAr-pAr-pGr | Cyclic phosphorylation | 9 | 0.4 | 100 | 1 | 7.68 | 953.11 | -3 | 2861.3486 | 2862.47 | 2861.3475 | 2294820.75 |
| 3516-3524 | Ur-pAr-pCr-pUr-pUr-pUr-pAr-pAr-pGr | Cyclic phosphorylation | 9 | -0.8 | 100 | 1 | 7.68 | 714.58 | -4 | 2861.3452 | 2862.47 | 2861.3475 | 5433733 |
| 3516-3524 | Ur-pAr-pCr-pUr-pUr-pUr-pAr-pAr-pGr | Cyclic phosphorylation | 9 | -0.2 | 100 | 1 | 7.68 | 1430.167 | -2 | 2861.3469 | 2862.47 | 2861.3475 | 4767246 |
| 3516-3524 | Ur-pAr-pCr-pUr-pUr-pUr-pAr-pAr-pGr | Cyclic phosphorylation | 9 | -0.62 | 100 | 1 | 7.72 | 571.462 | -5 | 2861.3457 | 2862.48 | 2861.3475 | 5750960.5 |
| 162-171 | Ur-pAr-pCr-pUr-pAr-pCr-pCr-pCr-pCr-pGr | Cyclic phosphorylation | 10 | 0.83 | 100 | 1 | 7.94 | 522.562 | -6 | 3140.4121 | 3141.75 | 3140.4095 | 621860.94 |
| 162-171 | Ur-pAr-pCr-pUr-pAr-pCr-pCr-pCr-pCr-pGr | Cyclic phosphorylation | 10 | 0.13 | 100 | 1 | 7.95 | 784.346 | -4 | 3140.4099 | 3141.61 | 3140.4095 | 2607249.5 |
| 162-171 | Ur-pAr-pCr-pUr-pAr-pCr-pCr-pCr-pCr-pGr | Cyclic phosphorylation | 10 | 0.13 | 100 | 1 | 7.96 | 627.275 | -5 | 3140.4099 | 3141.62 | 3140.4095 | 3201848.5 |
| 162-171 | Ur-pAr-pCr-pUr-pAr-pCr-pCr-pCr-pCr-pGr | Cyclic phosphorylation | 10 | 1.22 | 100 | 1 | 7.98 | 1046.131 | -3 | 3140.4133 | 3141.61 | 3140.4095 | 1387597.88 |
| 2739-2748 | Ar-pUr-pCr-pCr-pCr-pCr-pUr-pUr-pUr-pGr | Cyclic phosphorylation | 10 | 1.22 | 100 | 1 | 8.11 | 518.888 | -6 | 3118.3701 | 3119.54 | 3118.3663 | 1117030.88 |
| 2739-2748 | Ar-pUr-pCr-pCr-pCr-pCr-pUr-pUr-pUr-pGr | Cyclic phosphorylation | 10 | 0.83 | 100 | 1 | 8.12 | 1558.678 | -2 | 3118.3689 | 3119.7 | 3118.3663 | 1437702.62 |
| 2739-2748 | Ar-pUr-pCr-pCr-pCr-pCr-pUr-pUr-pUr-pGr | Cyclic phosphorylation | 10 | 1.06 | 100 | 1 | 8.12 | 1038.784 | -3 | 3118.3696 | 3119.7 | 3118.3663 | 892751 |
| 2739-2748 | Ar-pUr-pCr-pCr-pCr-pCr-pUr-pUr-pUr-pGr | Cyclic phosphorylation | 10 | 0.2 | 100 | 1 | 8.12 | 622.867 | -5 | 3118.3669 | 3119.55 | 3118.3663 | 3174841.25 |
| 2085-2092 | Ar-pCr-pAr-pAr-pAr-pCr-pAr-pGr | Cyclic phosphorylation | 8 | 0.93 | 100 | 1 | 8.12 | 519.072 | -5 | 2600.395 | 2601.42 | 2600.3926 | 1006905 |
| 2085-2092 | Ar-pCr-pAr-pAr-pAr-pCr-pAr-pGr | Cyclic phosphorylation | 8 | 0.08 | 100 | 1 | 8.12 | 1299.189 | -2 | 2600.3928 | 2601.46 | 2600.3926 | 2892061.75 |
| 2085-2092 | Ar-pCr-pAr-pAr-pAr-pCr-pAr-pGr | Cyclic phosphorylation | 8 | -0.11 | 100 | 1 | 8.12 | 649.341 | -4 | 2600.3923 | 2601.45 | 2600.3926 | 2640998 |
| 2739-2748 | Ar-pUr-pCr-pCr-pCr-pCr-pUr-pUr-pUr-pGr | Cyclic phosphorylation | 10 | 0.83 | 100 | 1 | 8.13 | 778.836 | -4 | 3118.3689 | 3119.7 | 3118.3663 | 955530.62 |
| 2085-2092 | Ar-pCr-pAr-pAr-pAr-pCr-pAr-pGr | Cyclic phosphorylation | 8 | 0.74 | 100 | 1 | 8.13 | 866.125 | -3 | 2600.3945 | 2601.46 | 2600.3926 | 1097164.12 |
| 433-441 | Ur-pCr-pAr-pUr-pCr-pAr-pAr-pAr-pGr | Cyclic phosphorylation | 9 | 1.25 | 99.6 | 1 | 8.31 | 960.458 | -3 | 2883.3943 | 2884.55 | 2883.3907 | 439197.94 |
| 433-441 | Ur-pCr-pAr-pUr-pCr-pAr-pAr-pAr-pGr | Cyclic phosphorylation | 9 | 0.82 | 99.6 | 1 | 8.31 | 575.671 | -5 | 2883.3931 | 2884.52 | 2883.3907 | 949336.81 |
| 433-441 | Ur-pCr-pAr-pUr-pCr-pAr-pAr-pAr-pGr | Cyclic phosphorylation | 9 | 0.82 | 99.6 | 1 | 8.32 | 1441.19 | -2 | 2883.3931 | 2884.54 | 2883.3907 | 1092660.75 |
| 660-667 | Ar-pUr-pCr-pUr-pAr-pCr-pAr-pGr | Phosphorylation | 8 | 1.19 | 92 | 1 | 8.33 | 1285.169 | -2 | 2572.3518 | 2573.39 | 2572.3487 | 430601.62 |
| 2728-2738 | Cr-pCr-pGr-pCr-pUr-pCr-pUr-pGr-pCr-pAr-pGr | Cyclic phosphorylation | 11 | 0.85 | 94.1 | 1 | 8.57 | 699.484 | -5 | 3501.4548 | 3502.99 | 3501.4519 | 477112.44 |
| 3566-3573 | Ar-pAr-pUr-pCr-pAr-pAr-pUr-pGr | Cyclic phosphorylation | 8 | -0.11 | 100 | 1 | 8.66 | 514.663 | -5 | 2578.3491 | 2579.42 | 2578.3494 | 2176874.75 |
| 3566-3573 | Ar-pAr-pUr-pCr-pAr-pAr-pUr-pGr | Cyclic phosphorylation | 8 | 0.08 | 100 | 1 | 8.67 | 858.443 | -3 | 2578.3496 | 2579.4 | 2578.3494 | 2093369.75 |
| 3566-3573 | Ar-pAr-pUr-pCr-pAr-pAr-pUr-pGr | Cyclic phosphorylation | 8 | -0.39 | 100 | 1 | 8.68 | 1288.167 | -2 | 2578.3484 | 2579.39 | 2578.3494 | 6270136 |
| 3566-3573 | Ar-pAr-pUr-pCr-pAr-pAr-pUr-pGr | Cyclic phosphorylation | 8 | -0.96 | 100 | 1 | 8.69 | 643.83 | -4 | 2578.3469 | 2579.4 | 2578.3494 | 5736198.5 |
| 3069-3077 | Ar-pCr-pAr-pUr-pAr-pCr-pGr-pUr-pGr | Cyclic phosphorylation | 9 | 0.21 | 93.9 | 1 | 8.73 | 579.07 | -5 | 2899.3862 | 2900.66 | 2899.3856 | 671309.06 |
| 222-232 | Cr-pCr-pUr-pUr-pUr-pCr-pUr-pUr-pCr-pAr-pGr | Cyclic phosphorylation | 11 | 1.07 | 100 | 1 | 8.9 | 855.343 | -4 | 3424.3953 | 3425.63 | 3424.3916 | 356300.41 |
| 222-232 | Cr-pCr-pUr-pUr-pUr-pCr-pUr-pUr-pCr-pAr-pGr | Cyclic phosphorylation | 11 | 1.14 | 100 | 1 | 8.9 | 684.072 | -5 | 3424.3955 | 3425.83 | 3424.3916 | 899709.38 |
| 222-232 | Cr-pCr-pUr-pUr-pUr-pCr-pUr-pUr-pCr-pAr-pGr | Cyclic phosphorylation | 11 | 1.71 | 100 | 1 | 8.9 | 1140.792 | -3 | 3424.3975 | 3425.85 | 3424.3916 | 434758.19 |
| 222-232 | Cr-pCr-pUr-pUr-pUr-pCr-pUr-pUr-pCr-pAr-pGr | Cyclic phosphorylation | 11 | 1.28 | 100 | 1.1 | 8.9 | 569.892 | -6 | 3424.396 | 3425.84 | 3424.3916 | 674097.75 |
| 222-232 | Cr-pCr-pUr-pUr-pUr-pCr-pUr-pUr-pCr-pAr-pGr | Cyclic phosphorylation | 11 | 1.49 | 100 | 1 | 8.9 | 1711.692 | -2 | 3424.3967 | 3425.85 | 3424.3916 | 378619.84 |
| 853-863 | Ur-pGr-pGr-pGr-pCr-pUr-pAr-pCr-pCr-pUr-pGr | Cyclic phosphorylation | 11 | 1.48 | 97.6 | 1 | 9.16 | 707.683 | -5 | 3542.4473 | 3543.98 | 3542.442 | 565065.75 |
| 853-863 | Ur-pGr-pGr-pGr-pCr-pUr-pAr-pCr-pCr-pUr-pGr | Cyclic phosphorylation | 11 | 1.48 | 97.6 | 1.1 | 9.18 | 589.568 | -6 | 3542.4473 | 3543.92 | 3542.442 | 472302.41 |
| 2931-2939 | Ar-pAr-pCr-pAr-pCr-pCr-pCr-pUr-pGr | Cyclic phosphorylation | 9 | 1.22 | 100 | 1 | 9.18 | 952.127 | -3 | 2858.3989 | 2859.5 | 2858.3954 | 508238.31 |
| 2931-2939 | Ar-pAr-pCr-pAr-pCr-pCr-pCr-pUr-pGr | Cyclic phosphorylation | 9 | 0.53 | 100 | 1 | 9.18 | 713.843 | -4 | 2858.397 | 2859.5 | 2858.3954 | 1208880.75 |
| 2931-2939 | Ar-pAr-pCr-pAr-pCr-pCr-pCr-pUr-pGr | Cyclic phosphorylation | 9 | 0.79 | 100 | 1 | 9.18 | 1428.693 | -2 | 2858.3977 | 2859.52 | 2858.3954 | 1150132.12 |
| 2931-2939 | Ar-pAr-pCr-pAr-pCr-pCr-pCr-pUr-pGr | Cyclic phosphorylation | 9 | 0.96 | 100 | 1 | 9.18 | 570.873 | -5 | 2858.3982 | 2859.54 | 2858.3954 | 981119.75 |
| 3678-3686 | Ur-pAr-pCr-pAr-pUr-pCr-pAr-pAr-pGr | Cyclic phosphorylation | 9 | -0.19 | 100 | 1 | 9.42 | 575.871 | -5 | 2883.3901 | 2884.52 | 2883.3907 | 3336706.75 |
| 3678-3686 | Ur-pAr-pCr-pAr-pUr-pCr-pAr-pAr-pGr | Cyclic phosphorylation | 9 | -0.62 | 100 | 1 | 9.42 | 720.09 | -4 | 2883.3889 | 2884.52 | 2883.3907 | 4120304.5 |
| 3678-3686 | Ur-pAr-pCr-pAr-pUr-pCr-pAr-pAr-pGr | Cyclic phosphorylation | 9 | 0.32 | 100 | 1 | 9.42 | 960.457 | -3 | 2883.3916 | 2884.54 | 2883.3907 | 1704281.88 |
| 3678-3686 | Ur-pAr-pCr-pAr-pUr-pCr-pAr-pAr-pGr | Cyclic phosphorylation | 9 | -0.36 | 100 | 1 | 9.42 | 1441.189 | -2 | 2883.3896 | 2884.52 | 2883.3907 | 3264790.5 |
| 1845-1854 | Ar-pUr-pCr-pAr-pCr-pCr-pCr-pCr-pUr-pGr | Cyclic phosphorylation | 10 | 0.9 | 100 | 1 | 9.55 | 784.347 | -4 | 3140.4124 | 3141.79 | 3140.4095 | 759969.25 |
| 1845-1854 | Ar-pUr-pCr-pAr-pCr-pCr-pCr-pCr-pUr-pGr | Cyclic phosphorylation | 10 | 0.83 | 100 | 1 | 9.55 | 627.275 | -5 | 3140.4121 | 3141.63 | 3140.4095 | 2036733.75 |
| 1659-1666 | Ar-pAr-pCr-pAr-pAr-pAr-pUr-pGr | Cyclic phosphorylation | 8 | 0.31 | 98.6 | 1 | 9.62 | 866.453 | -3 | 2601.3774 | 2602.41 | 2601.3766 | 3920833 |
| 1659-1666 | Ar-pAr-pCr-pAr-pAr-pAr-pUr-pGr | Cyclic phosphorylation | 8 | -0.34 | 98.6 | 1 | 9.64 | 1300.181 | -2 | 2601.3757 | 2602.43 | 2601.3766 | 9495831 |
| 1659-1666 | Ar-pAr-pCr-pAr-pAr-pAr-pUr-pGr | Cyclic phosphorylation | 8 | -0.81 | 98.6 | 1 | 9.64 | 649.336 | -4 | 2601.3745 | 2602.43 | 2601.3766 | 9436208 |
| 1659-1666 | Ar-pAr-pCr-pAr-pAr-pAr-pUr-pGr | Cyclic phosphorylation | 8 | 0.31 | 98.6 | 1 | 9.67 | 519.268 | -5 | 2601.3774 | 2602.43 | 2601.3766 | 2938335.5 |
| 4097-4105 | Cr-pAr-pAr-pUr-pAr-pAr-pAr-pCr-pGr | Cyclic phosphorylation | 9 | 0.19 | 100 | 1 | 9.7 | 1452.703 | -2 | 2906.4185 | 2907.6 | 2906.4179 | 3763286.75 |
| 4097-4105 | Cr-pAr-pAr-pUr-pAr-pAr-pAr-pCr-pGr | Cyclic phosphorylation | 9 | 0.19 | 100 | 1 | 9.7 | 580.477 | -5 | 2906.4185 | 2907.55 | 2906.4179 | 3581454 |
| 4097-4105 | Cr-pAr-pAr-pUr-pAr-pAr-pAr-pCr-pGr | Cyclic phosphorylation | 9 | 0.19 | 100 | 1 | 9.72 | 725.848 | -4 | 2906.4185 | 2907.58 | 2906.4179 | 3703423.75 |
| 1024-1032 | Ar-pAr-pUr-pCr-pCr-pAr-pUr-pCr-pGr | Cyclic phosphorylation | 9 | 0.06 | 100 | 1 | 9.76 | 571.069 | -5 | 2859.3796 | 2860.51 | 2859.3795 | 4712538 |
| 1024-1032 | Ar-pAr-pUr-pCr-pCr-pAr-pUr-pCr-pGr | Cyclic phosphorylation | 9 | 0.49 | 100 | 1 | 9.77 | 1429.184 | -2 | 2859.3809 | 2860.52 | 2859.3795 | 3475402 |
| 1024-1032 | Ar-pAr-pUr-pCr-pCr-pAr-pUr-pCr-pGr | Cyclic phosphorylation | 9 | 0.23 | 100 | 1 | 9.77 | 714.088 | -4 | 2859.3801 | 2860.49 | 2859.3795 | 3189111.75 |
| 1437-1446 | Ar-pAr-pGr-pCr-pCr-pCr-pUr-pUr-pCr-pGr | Cyclic phosphorylation | 10 | 0.73 | 95.6 | 1 | 9.82 | 635.277 | -5 | 3180.418 | 3181.85 | 3180.4157 | 1227176.62 |
| 650-659 | Cr-pUr-pAr-pCr-pUr-pUr-pCr-pAr-pAr-pGr | Cyclic phosphorylation | 10 | 0.47 | 100 | 1 | 9.86 | 526.727 | -6 | 3165.4062 | 3166.64 | 3165.4048 | 4072629.5 |
| 650-659 | Cr-pUr-pAr-pCr-pUr-pUr-pCr-pAr-pAr-pGr | Cyclic phosphorylation | 10 | -0.15 | 100 | 1 | 9.86 | 632.274 | -5 | 3165.4043 | 3166.59 | 3165.4048 | 14821535 |
| 650-659 | Cr-pUr-pAr-pCr-pUr-pUr-pCr-pAr-pAr-pGr | Cyclic phosphorylation | 10 | 1.01 | 100 | 1 | 9.87 | 1054.463 | -3 | 3165.408 | 3166.73 | 3165.4048 | 5018607.5 |
| 650-659 | Cr-pUr-pAr-pCr-pUr-pUr-pCr-pAr-pAr-pGr | Cyclic phosphorylation | 10 | 0.93 | 100 | 1 | 9.87 | 1582.197 | -2 | 3165.4077 | 3166.77 | 3165.4048 | 8569266 |
| 650-659 | Cr-pUr-pAr-pCr-pUr-pUr-pCr-pAr-pAr-pGr | Cyclic phosphorylation | 10 | 0.93 | 100 | 1 | 9.87 | 790.595 | -4 | 3165.4077 | 3166.74 | 3165.4048 | 7539954 |
| 2022-2029 | Ar-pAr-pCr-pAr-pAr-pUr-pAr-pGr | Cyclic phosphorylation | 8 | -0.62 | 100 | 1 | 9.98 | 649.587 | -4 | 2601.375 | 2602.43 | 2601.3766 | 5571497.5 |
| 2022-2029 | Ar-pAr-pCr-pAr-pAr-pUr-pAr-pGr | Cyclic phosphorylation | 8 | 0.31 | 100 | 1 | 9.98 | 866.453 | -3 | 2601.3774 | 2602.45 | 2601.3766 | 2446428 |
| 2022-2029 | Ar-pAr-pCr-pAr-pAr-pUr-pAr-pGr | Cyclic phosphorylation | 8 | -0.16 | 100 | 1 | 9.98 | 1300.182 | -2 | 2601.3762 | 2602.44 | 2601.3766 | 6144255.5 |
| 2022-2029 | Ar-pAr-pCr-pAr-pAr-pUr-pAr-pGr | Cyclic phosphorylation | 8 | 0.88 | 100 | 1 | 10.01 | 519.469 | -5 | 2601.3789 | 2602.41 | 2601.3766 | 1758636 |
| 1378-1386 | Ar-pCr-pUr-pCr-pCr-pAr-pAr-pAr-pGr | Cyclic phosphorylation | 9 | 0.95 | 99.8 | 1 | 10.08 | 1440.698 | -2 | 2882.4094 | 2883.54 | 2882.4067 | 878888.25 |
| 541-550 | Cr-pCr-pAr-pAr-pCr-pAr-pAr-pCr-pUr-pGr | Cyclic phosphorylation | 10 | 1.01 | 100 | 1 | 10.22 | 796.106 | -4 | 3187.4512 | 3188.83 | 3187.448 | 1755245.5 |

| | | | | | | | | | | | | | |
|---|---|---|---|---|---|---|---|---|---|---|---|---|---|
| 541-550 | Cr-pCr-pAr-pAr-pCr-pAr-pAr-pCr-pUr-pGr | Cyclic phosphorylation | 10 | 1.39 | 100 | 1 | 10.22 | 1061.811 | -3 | 3187.4524 | 3188.8 | 3187.448 | 1215765.88 |
| 541-550 | Cr-pCr-pAr-pAr-pCr-pAr-pAr-pCr-pUr-pGr | Cyclic phosphorylation | 10 | 0.32 | 100 | 1 | 10.22 | 636.683 | -5 | 3187.449 | 3188.69 | 3187.448 | 3753890.25 |
| 541-550 | Cr-pCr-pAr-pAr-pCr-pAr-pAr-pCr-pUr-pGr | Cyclic phosphorylation | 10 | 1.24 | 100 | 1 | 10.23 | 1593.219 | -2 | 3187.4519 | 3188.83 | 3187.448 | 2425529.5 |
| 1296-1304 | Ar-pCr-pAr-pGr-pGr-pCr-pAr-pAr-pGr | Cyclic phosphorylation | 9 | 1.19 | 99.6 | 1 | 10.29 | 739.603 | -4 | 2961.4385 | 2962.6 | 2961.435 | 721574.31 |
| 1296-1304 | Ar-pCr-pAr-pGr-pGr-pCr-pAr-pAr-pGr | Cyclic phosphorylation | 9 | 0.94 | 99.6 | 1 | 10.3 | 591.481 | -5 | 2961.4377 | 2962.61 | 2961.435 | 1085671.25 |
| 1296-1304 | Ar-pCr-pAr-pGr-pGr-pCr-pAr-pAr-pGr | Cyclic phosphorylation | 9 | 0.86 | 99.6 | 1 | 10.3 | 1480.213 | -2 | 2961.4375 | 2962.75 | 2961.435 | 1114362.62 |
| 1765-1773 | Ar-pUr-pAr-pCr-pCr-pAr-pCr-pAr-pGr | Cyclic phosphorylation | 9 | -0.57 | 100 | 1 | 10.48 | 575.674 | -5 | 2882.405 | 2883.55 | 2882.4067 | 3533545 |
| 1765-1773 | Ar-pUr-pAr-pCr-pCr-pAr-pCr-pAr-pGr | Cyclic phosphorylation | 9 | -0.4 | 100 | 1 | 10.49 | 1440.696 | -2 | 2882.4055 | 2883.54 | 2882.4067 | 3257036.25 |
| 1765-1773 | Ar-pUr-pAr-pCr-pCr-pAr-pCr-pAr-pGr | Cyclic phosphorylation | 9 | -0.23 | 100 | 1 | 10.49 | 719.845 | -4 | 2882.406 | 2883.55 | 2882.4067 | 2338276.5 |
| 1765-1773 | Ar-pUr-pAr-pCr-pCr-pAr-pCr-pAr-pGr | Cyclic phosphorylation | 9 | 0.61 | 100 | 1 | 10.5 | 960.13 | -3 | 2882.4084 | 2883.55 | 2882.4067 | 1148137.12 |
| 3566-3573 | Ar-pAr-pUr-pCr-pAr-pAr-pUr-pGr | Phosphorylation | 8 | 0.15 | 100 | 1 | 10.62 | 648.083 | -4 | 2596.3604 | 2597.32 | 2596.36 | 262574.19 |
| 3566-3573 | Ar-pAr-pUr-pCr-pAr-pAr-pUr-pGr | Phosphorylation | 8 | 1.09 | 100 | 1 | 10.62 | 1297.174 | -2 | 2596.3628 | 2597.4 | 2596.36 | 282612.03 |
| 1367-1376 | Cr-pAr-pAr-pCr-pAr-pAr-pCr-pCr-pUr-pGr | Cyclic phosphorylation | 10 | 0.39 | 100 | 1 | 10.7 | 796.105 | -4 | 3187.4492 | 3188.83 | 3187.448 | 1075200.75 |
| 3441-3450 | Ar-pUr-pCr-pGr-pGr-pCr-pAr-pUr-pUr-pGr | Cyclic phosphorylation | 10 | 0.74 | 92.4 | 1 | 10.7 | 643.475 | -5 | 3221.4082 | 3222.8 | 3221.4058 | 558156.31 |
| 1367-1376 | Cr-pAr-pAr-pCr-pAr-pAr-pCr-pCr-pUr-pGr | Cyclic phosphorylation | 10 | 0.78 | 100 | 1 | 10.72 | 1593.219 | -2 | 3187.4504 | 3188.83 | 3187.448 | 1522672.75 |
| 1367-1376 | Cr-pAr-pAr-pCr-pAr-pAr-pCr-pCr-pUr-pGr | Cyclic phosphorylation | 10 | -0.37 | 100 | 1 | 10.72 | 636.682 | -5 | 3187.4468 | 3188.8 | 3187.448 | 2262533.5 |
| 872-881 | Ar-pAr-pCr-pCr-pUr-pUr-pCr-pCr-pUr-pGr | Cyclic phosphorylation | 10 | 0.16 | 100 | 1 | 10.76 | 522.725 | -6 | 3141.394 | 3142.6 | 3141.3935 | 2530387.25 |
| 872-881 | Ar-pAr-pCr-pCr-pUr-pUr-pCr-pCr-pUr-pGr | Cyclic phosphorylation | 10 | 0.16 | 100 | 1 | 10.76 | 1570.191 | -2 | 3141.394 | 3142.76 | 3141.3935 | 4499834.5 |
| 872-881 | Ar-pAr-pCr-pCr-pUr-pUr-pCr-pCr-pUr-pGr | Cyclic phosphorylation | 10 | -0.77 | 100 | 1 | 10.77 | 627.471 | -5 | 3141.3911 | 3142.6 | 3141.3935 | 7699246 |
| 872-881 | Ar-pAr-pCr-pCr-pUr-pUr-pCr-pCr-pUr-pGr | Cyclic phosphorylation | 10 | -0.07 | 100 | 1 | 10.77 | 784.591 | -4 | 3141.3933 | 3142.6 | 3141.3935 | 3773603.25 |
| 872-881 | Ar-pAr-pCr-pCr-pUr-pUr-pCr-pCr-pUr-pGr | Cyclic phosphorylation | 10 | 0.47 | 100 | 1 | 10.77 | 1046.458 | -3 | 3141.395 | 3142.61 | 3141.3935 | 2682116.25 |
| 2415-2423 | Ar-pUr-pCr-pUr-pAr-pCr-pAr-pAr-pGr | Cyclic phosphorylation | 9 | -0.02 | 100 | 1 | 10.84 | 1441.189 | -2 | 2883.3906 | 2884.54 | 2883.3907 | 3903885.75 |
| 2415-2423 | Ar-pUr-pCr-pUr-pAr-pCr-pAr-pAr-pGr | Cyclic phosphorylation | 9 | -0.28 | 100 | 1 | 10.86 | 720.091 | -4 | 2883.3899 | 2884.5 | 2883.3907 | 2977704.5 |
| 2415-2423 | Ar-pUr-pCr-pUr-pAr-pCr-pAr-pAr-pGr | Cyclic phosphorylation | 9 | -0.36 | 100 | 1 | 10.86 | 575.871 | -5 | 2883.3896 | 2884.53 | 2883.3907 | 4452089 |
| 2415-2423 | Ar-pUr-pCr-pUr-pAr-pCr-pAr-pAr-pGr | Cyclic phosphorylation | 9 | 0.82 | 100 | 1 | 10.86 | 960.458 | -3 | 2883.3931 | 2884.53 | 2883.3907 | 1530012.75 |
| 1703-1713 | Cr-pGr-pUr-pGr-pCr-pUr-pGr-pAr-pCr-pAr-pGr | Cyclic phosphorylation | 11 | 1.3 | 100 | 1 | 11.06 | 890.612 | -4 | 3565.4739 | 3566.98 | 3565.4692 | 534269.19 |
| 1703-1713 | Cr-pGr-pUr-pGr-pCr-pUr-pGr-pAr-pCr-pAr-pGr | Cyclic phosphorylation | 11 | 1.64 | 100 | 1 | 11.06 | 1187.818 | -3 | 3565.4751 | 3566.93 | 3565.4692 | 676585.06 |
| 3585-3593 | Ar-pAr-pCr-pAr-pUr-pCr-pCr-pAr-pGr | Cyclic phosphorylation | 9 | 0.36 | 100 | 1 | 11.06 | 960.129 | -3 | 2882.4077 | 2883.56 | 2882.4067 | 2844555.5 |
| 3585-3593 | Ar-pAr-pCr-pAr-pUr-pCr-pCr-pAr-pGr | Cyclic phosphorylation | 9 | -0.4 | 100 | 1 | 11.06 | 1440.696 | -2 | 2882.4055 | 2883.54 | 2882.4067 | 6214304.5 |
| 1703-1713 | Cr-pGr-pUr-pGr-pCr-pUr-pGr-pAr-pCr-pAr-pGr | Cyclic phosphorylation | 11 | 1.51 | 100 | 1 | 11.07 | 712.288 | -5 | 3565.4746 | 3566.93 | 3565.4692 | 1157024.12 |
| 3585-3593 | Ar-pAr-pCr-pAr-pUr-pCr-pCr-pAr-pGr | Cyclic phosphorylation | 9 | -0.91 | 100 | 1 | 11.08 | 719.844 | -4 | 2882.4041 | 2883.55 | 2882.4067 | 7119147 |
| 1703-1713 | Cr-pGr-pUr-pGr-pCr-pUr-pGr-pAr-pCr-pAr-pGr | Cyclic phosphorylation | 11 | 2.05 | 100 | 1 | 11.08 | 593.406 | -6 | 3565.4766 | 3566.94 | 3565.4692 | 637563.19 |
| 3585-3593 | Ar-pAr-pCr-pAr-pUr-pCr-pCr-pAr-pGr | Cyclic phosphorylation | 9 | -0.15 | 100 | 1 | 11.08 | 575.674 | -5 | 2882.4062 | 2883.53 | 2882.4067 | 3815404.25 |
| 605-614 | Cr-pAr-pAr-pCr-pUr-pUr-pCr-pAr-pAr-pGr | Cyclic phosphorylation | 10 | 1.27 | 100 | 1 | 11.17 | 1062.139 | -3 | 3188.436 | 3189.81 | 3188.432 | 760921.44 |
| 605-614 | Cr-pAr-pAr-pCr-pUr-pUr-pCr-pAr-pAr-pGr | Cyclic phosphorylation | 10 | 0.97 | 100 | 1 | 11.17 | 530.566 | -6 | 3188.4351 | 3189.8 | 3188.432 | 549506.69 |
| 605-614 | Cr-pAr-pAr-pCr-pUr-pUr-pCr-pAr-pAr-pGr | Cyclic phosphorylation | 10 | 0.81 | 100 | 1 | 11.17 | 796.352 | -4 | 3188.4346 | 3189.82 | 3188.432 | 1281131 |
| 605-614 | Cr-pAr-pAr-pCr-pUr-pUr-pCr-pAr-pAr-pGr | Cyclic phosphorylation | 10 | 0.28 | 100 | 1 | 11.17 | 636.88 | -5 | 3188.4329 | 3189.65 | 3188.432 | 2170971.5 |
| 605-614 | Cr-pAr-pAr-pCr-pUr-pUr-pCr-pAr-pAr-pGr | Cyclic phosphorylation | 10 | 0.89 | 100 | 1 | 11.17 | 1593.711 | -2 | 3188.4348 | 3189.79 | 3188.432 | 1583589.38 |
| 1659-1666 | Ar-pAr-pCr-pAr-pAr-pAr-pUr-pGr | Phosphorylation | 8 | 1.22 | 99.3 | 1 | 11.36 | 872.457 | -3 | 2619.3904 | 2620.47 | 2619.3872 | 245167.19 |
| 1659-1666 | Ar-pAr-pCr-pAr-pAr-pAr-pUr-pGr | Phosphorylation | 8 | 1.41 | 99.3 | 1 | 11.36 | 1309.189 | -2 | 2619.3909 | 2620.46 | 2619.3872 | 475266.56 |
| 1659-1666 | Ar-pAr-pCr-pAr-pAr-pAr-pUr-pGr | Phosphorylation | 8 | 1.97 | 99.3 | 1 | 11.36 | 653.841 | -4 | 2619.3923 | 2620.35 | 2619.3872 | 345489 |
| 4110-4119 | Ur-pUr-pUr-pAr-pAr-pCr-pUr-pAr-pAr-pGr | Cyclic phosphorylation | 10 | 0.96 | 100 | 1 | 11.52 | 796.844 | -4 | 3190.4031 | 3191.76 | 3190.4 | 1159322.62 |
| 3838-3848 | Ar-pGr-pCr-pCr-pCr-pGr-pUr-pGr-pCr-pUr-pGr | Cyclic phosphorylation | 11 | 1.17 | 95.7 | 1 | 11.52 | 707.486 | -5 | 3541.4622 | 3542.84 | 3541.458 | 544831 |
| 4110-4119 | Ur-pUr-pUr-pAr-pAr-pCr-pUr-pAr-pAr-pGr | Cyclic phosphorylation | 10 | 1.11 | 100 | 1 | 11.52 | 1594.696 | -2 | 3190.4036 | 3191.78 | 3190.4 | 1543223.25 |
| 4110-4119 | Ur-pUr-pUr-pAr-pAr-pCr-pUr-pAr-pAr-pGr | Cyclic phosphorylation | 10 | 1.73 | 100 | 1 | 11.52 | 1062.795 | -3 | 3190.4055 | 3191.74 | 3190.4 | 791547.31 |
| 4110-4119 | Ur-pUr-pUr-pAr-pAr-pCr-pUr-pAr-pAr-pGr | Cyclic phosphorylation | 10 | 0.43 | 100 | 1 | 11.52 | 637.273 | -5 | 3190.4014 | 3191.74 | 3190.4 | 3035860.75 |
| 4110-4119 | Ur-pUr-pUr-pAr-pAr-pCr-pUr-pAr-pAr-pGr | Cyclic phosphorylation | 10 | 1.5 | 100 | 1 | 11.52 | 530.894 | -6 | 3190.4048 | 3191.73 | 3190.4 | 630873.12 |
| 650-659 | Cr-pUr-pAr-pCr-pUr-pUr-pCr-pAr-pAr-pGr | Phosphorylation | 10 | 1.91 | 99.5 | 1 | 11.53 | 795.098 | -4 | 3183.4214 | 3184.78 | 3183.4153 | 513898.69 |
| 650-659 | Cr-pUr-pAr-pCr-pUr-pUr-pCr-pAr-pAr-pGr | Phosphorylation | 10 | 2.06 | 99.5 | 1 | 11.53 | 1591.204 | -2 | 3183.4219 | 3184.75 | 3183.4153 | 490011.44 |
| 650-659 | Cr-pUr-pAr-pCr-pUr-pUr-pCr-pAr-pAr-pGr | Phosphorylation | 10 | 1.68 | 99.5 | 1.7 | 11.57 | 635.877 | -5 | 3183.4207 | 3184.94 | 3183.4153 | 470905.09 |
| 2022-2029 | Ar-pAr-pCr-pAr-pAr-pUr-pAr-pGr | Phosphorylation | 8 | 1.5 | 91.5 | 1 | 11.57 | 872.457 | -3 | 2619.3911 | 2620.45 | 2619.3872 | 388025.66 |
| 2022-2029 | Ar-pAr-pCr-pAr-pAr-pUr-pAr-pGr | Phosphorylation | 8 | 1.41 | 91.5 | 1 | 11.57 | 1309.189 | -2 | 2619.3909 | 2620.45 | 2619.3872 | 738382.44 |
| 2022-2029 | Ar-pAr-pCr-pAr-pAr-pUr-pAr-pGr | Phosphorylation | 8 | 1.13 | 91.5 | 1 | 11.57 | 654.091 | -4 | 2619.3901 | 2620.45 | 2619.3872 | 571135.25 |
| 1823-1834 | Cr-pAr-pGr-pCr-pUr-pUr-pCr-pGr-pGr-pCr-pGr-pGr | Cyclic phosphorylation | 12 | 0.86 | 100 | 1 | 11.59 | 646.911 | -6 | 3886.5088 | 3888.11 | 3886.5054 | 1121329.62 |
| 1823-1834 | Cr-pAr-pGr-pCr-pUr-pUr-pCr-pGr-pGr-pCr-pGr-pGr | Cyclic phosphorylation | 12 | 1.43 | 100 | 1 | 11.59 | 1294.83 | -3 | 3886.511 | 3888.18 | 3886.5054 | 670269.44 |
| 1823-1834 | Cr-pAr-pGr-pCr-pUr-pUr-pCr-pGr-pGr-pCr-pGr-pGr | Cyclic phosphorylation | 12 | 1.49 | 100 | 1.1 | 11.6 | 776.495 | -5 | 3886.5112 | 3888.12 | 3886.5054 | 572950.06 |
| 986-995 | Cr-pAr-pUr-pCr-pUr-pAr-pCr-pCr-pAr-pGr | Phosphorylation | 10 | 1.26 | 96.1 | 1 | 11.75 | 794.852 | -4 | 3182.4353 | 3183.86 | 3182.4313 | 622711.38 |
| 986-995 | Cr-pAr-pUr-pCr-pUr-pAr-pCr-pCr-pAr-pGr | Phosphorylation | 10 | 1.03 | 96.1 | 1 | 11.76 | 1590.711 | -2 | 3182.4346 | 3183.85 | 3182.4313 | 447069.78 |
| 1643-1653 | Cr-pAr-pCr-pCr-pAr-pAr-pUr-pCr-pUr-pCr-pGr | Cyclic phosphorylation | 11 | 2.43 | 100 | 1 | 11.83 | 1734.228 | -2 | 3469.4705 | 3470.87 | 3469.462 | 3976271 |
| 1643-1653 | Cr-pAr-pCr-pCr-pAr-pAr-pUr-pCr-pUr-pCr-pGr | Cyclic phosphorylation | 11 | 2.08 | 100 | 1 | 11.83 | 1155.816 | -3 | 3469.4692 | 3470.86 | 3469.462 | 4220048 |
| 1643-1653 | Cr-pAr-pCr-pCr-pAr-pAr-pUr-pCr-pUr-pCr-pGr | Cyclic phosphorylation | 11 | 0.67 | 100 | 1 | 11.83 | 577.404 | -6 | 3469.4644 | 3470.86 | 3469.462 | 5619784 |
| 1643-1653 | Cr-pAr-pCr-pCr-pAr-pAr-pUr-pCr-pUr-pCr-pGr | Cyclic phosphorylation | 11 | -0.03 | 100 | 1 | 11.83 | 693.085 | -5 | 3469.4619 | 3470.83 | 3469.462 | 8346800 |
| 1643-1653 | Cr-pAr-pCr-pCr-pAr-pAr-pUr-pCr-pUr-pCr-pGr | Cyclic phosphorylation | 11 | 1.73 | 100 | 1 | 11.84 | 866.61 | -4 | 3469.468 | 3470.86 | 3469.462 | 4262254 |
| 3375-3384 | Ar-pAr-pCr-pUr-pUr-pCr-pUr-pAr-pCr-pGr | Cyclic phosphorylation | 10 | 0.78 | 100 | 1 | 11.9 | 1054.463 | -3 | 3165.4072 | 3166.77 | 3165.4048 | 2743796.5 |
| 3375-3384 | Ar-pAr-pCr-pUr-pUr-pCr-pUr-pAr-pCr-pGr | Cyclic phosphorylation | 10 | 0.16 | 100 | 1 | 11.91 | 790.594 | -4 | 3165.4053 | 3166.63 | 3165.4048 | 5102487 |
| 3375-3384 | Ar-pAr-pCr-pUr-pUr-pCr-pUr-pAr-pCr-pGr | Cyclic phosphorylation | 10 | -0.3 | 100 | 1 | 11.91 | 632.274 | -5 | 3165.4038 | 3166.78 | 3165.4048 | 7673983.5 |
| 3375-3384 | Ar-pAr-pCr-pUr-pUr-pCr-pUr-pAr-pCr-pGr | Cyclic phosphorylation | 10 | 0.62 | 100 | 1 | 11.91 | 1582.197 | -2 | 3165.4067 | 3166.61 | 3165.4048 | 4596242.5 |
| 3375-3384 | Ar-pAr-pCr-pUr-pUr-pCr-pUr-pAr-pCr-pGr | Cyclic phosphorylation | 10 | 0.93 | 100 | 1 | 11.91 | 526.728 | -6 | 3165.4077 | 3166.8 | 3165.4048 | 806810.81 |
| 1765-1773 | Ar-pUr-pAr-pCr-pCr-pAr-pCr-pAr-pGr | Phosphorylation | 9 | 0.84 | 99.3 | 1 | 11.93 | 724.348 | -4 | 2900.4197 | 2901.53 | 2900.4172 | 434541.25 |
| 1765-1773 | Ar-pUr-pAr-pCr-pCr-pAr-pCr-pAr-pGr | Phosphorylation | 9 | 1.09 | 99.3 | 1 | 11.95 | 1449.703 | -2 | 2900.4204 | 2901.56 | 2900.4172 | 417559.22 |
| 2520-2529 | Ur-pUr-pCr-pAr-pAr-pCr-pAr-pAr-pAr-pGr | Cyclic phosphorylation | 10 | 0.05 | 100 | 1 | 12.05 | 641.682 | -5 | 3212.4434 | 3213.81 | 3212.4432 | 4446799 |
| 3765-3775 | Ar-pCr-pAr-pGr-pCr-pUr-pGr-pCr-pUr-pGr | Cyclic phosphorylation | 11 | 1.33 | 100 | 1 | 12.05 | 586.738 | -6 | 3525.4678 | 3526.97 | 3525.4631 | 1158061.62 |
| 3765-3775 | Ar-pCr-pAr-pGr-pCr-pUr-pGr-pCr-pUr-pGr | Cyclic phosphorylation | 11 | 1.26 | 100 | 1 | 12.05 | 704.286 | -5 | 3525.4675 | 3526.91 | 3525.4631 | 1154994.25 |
| 3765-3775 | Ar-pCr-pAr-pGr-pCr-pUr-pGr-pCr-pUr-pGr | Cyclic phosphorylation | 11 | 1.47 | 100 | 1 | 12.05 | 1174.483 | -3 | 3525.4683 | 3526.94 | 3525.4631 | 727896.62 |
| 2520-2529 | Ur-pUr-pCr-pAr-pAr-pCr-pAr-pAr-pAr-pGr | Cyclic phosphorylation | 10 | 0.96 | 100 | 1 | 12.07 | 1605.717 | -2 | 3212.4463 | 3213.84 | 3212.4432 | 2566183.25 |
| 2520-2529 | Ur-pUr-pCr-pAr-pAr-pCr-pAr-pAr-pAr-pGr | Cyclic phosphorylation | 10 | 1.19 | 100 | 1 | 12.07 | 1070.143 | -3 | 3212.447 | 3213.87 | 3212.4432 | 1295537.38 |
| 2520-2529 | Ur-pUr-pCr-pAr-pAr-pCr-pAr-pAr-pAr-pGr | Cyclic phosphorylation | 10 | 0.58 | 100 | 1 | 12.07 | 802.354 | -4 | 3212.4451 | 3213.83 | 3212.4432 | 1933042.62 |
| 3292-3303 | Cr-pCr-pAr-pUr-pCr-pUr-pGr-pCr-pCr-pAr-pCr-pGr | Cyclic phosphorylation | 12 | 2.66 | 99.9 | 1 | 12.23 | 1262.829 | -3 | 3790.5083 | 3792.09 | 3790.4982 | 1446357.88 |
| 3292-3303 | Cr-pCr-pAr-pUr-pCr-pUr-pGr-pCr-pCr-pAr-pCr-pGr | Cyclic phosphorylation | 12 | 2.85 | 99.9 | 1 | 12.26 | 757.294 | -5 | 3790.509 | 3792.11 | 3790.4982 | 1443890.62 |
| 3292-3303 | Cr-pCr-pAr-pUr-pCr-pUr-pGr-pCr-pCr-pAr-pCr-pGr | Cyclic phosphorylation | 12 | 2.53 | 99.9 | 1 | 12.28 | 631.077 | -6 | 3790.5078 | 3792.1 | 3790.4982 | 1847712.88 |
| 3585-3593 | Ar-pAr-pCr-pAr-pUr-pCr-pCr-pAr-pGr | Phosphorylation | 9 | 1.09 | 99.9 | 1 | 12.4 | 724.348 | -4 | 2900.4204 | 2901.55 | 2900.4172 | 997206.25 |
| 3585-3593 | Ar-pAr-pCr-pAr-pUr-pCr-pCr-pAr-pGr | Phosphorylation | 9 | 1.01 | 99.9 | 1 | 12.41 | 1449.704 | -2 | 2900.4202 | 2901.59 | 2900.4172 | 1241573 |
| 1226-1236 | Cr-pUr-pUr-pCr-pAr-pCr-pAr-pAr-pAr-pCr-pGr | Cyclic phosphorylation | 11 | 0.94 | 100 | 1 | 12.43 | 872.612 | -4 | 3493.4766 | 3494.99 | 3493.4733 | 1912328.38 |
| 1226-1236 | Cr-pUr-pUr-pCr-pAr-pCr-pAr-pAr-pAr-pCr-pGr | Cyclic phosphorylation | 11 | -0.03 | 100 | 1 | 12.45 | 697.888 | -5 | 3493.4731 | 3494.94 | 3493.4733 | 4487198 |
| 1226-1236 | Cr-pUr-pUr-pCr-pAr-pCr-pAr-pAr-pAr-pCr-pGr | Cyclic phosphorylation | 11 | 0.94 | 100 | 1 | 12.45 | 1163.819 | -3 | 3493.4766 | 3494.93 | 3493.4733 | 2040840 |
| 1226-1236 | Cr-pUr-pUr-pCr-pAr-pCr-pAr-pAr-pAr-pCr-pGr | Cyclic phosphorylation | 11 | 0.24 | 100 | 1 | 12.47 | 581.405 | -6 | 3493.4741 | 3494.94 | 3493.4733 | 2786693.75 |
| 1226-1236 | Cr-pUr-pUr-pCr-pAr-pCr-pAr-pAr-pAr-pCr-pGr | Cyclic phosphorylation | 11 | 0.94 | 100 | 1 | 12.47 | 1746.232 | -2 | 3493.4766 | 3494.95 | 3493.4733 | 2136072.5 |
| 1692-1702 | Ar-pCr-pCr-pGr-pGr-pCr-pAr-pCr-pCr-pGr-pGr | Cyclic phosphorylation | 11 | 0.55 | 100 | 1 | 12.47 | 711.894 | -5 | 3563.5032 | 3565.04 | 3563.5012 | 986254.88 |
| 1692-1702 | Ar-pCr-pCr-pGr-pGr-pCr-pAr-pCr-pCr-pGr-pGr | Cyclic phosphorylation | 11 | 1.03 | 100 | 1 | 12.47 | 593.077 | -6 | 3563.5049 | 3564.99 | 3563.5012 | 852099 |
| 1692-1702 | Ar-pCr-pCr-pGr-pGr-pCr-pAr-pCr-pCr-pGr-pGr | Cyclic phosphorylation | 11 | 0.76 | 100 | 1 | 12.47 | 1187.162 | -3 | 3563.5039 | 3565.04 | 3563.5012 | 753803.31 |
| 49-60 | Cr-pCr-pAr-pCr-pCr-pAr-pUr-pGr-pUr-pUr-pCr-pGr | Cyclic phosphorylation | 12 | 0.95 | 91.1 | 1 | 12.47 | 631.074 | -6 | 3791.4858 | 3793.12 | 3791.4822 | 687429.12 |
| 49-60 | Cr-pCr-pAr-pCr-pCr-pAr-pUr-pGr-pUr-pUr-pCr-pGr | Cyclic phosphorylation | 12 | 1.46 | 91.1 | 1 | 12.5 | 757.691 | -5 | 3791.4878 | 3793.35 | 3791.4822 | 2007365.88 |
| 3786-3796 | Ar-pAr-pGr-pGr-pCr-pUr-pGr-pUr-pUr-pGr | Cyclic phosphorylation | 11 | 1.64 | 100 | 1 | 12.59 | 1201.482 | -3 | 3606.4653 | 3608.01 | 3606.4594 | 569144.94 |
| 3786-3796 | Ar-pAr-pGr-pGr-pCr-pUr-pGr-pUr-pUr-pGr | Cyclic phosphorylation | 11 | 1.44 | 100 | 1 | 12.59 | 720.486 | -5 | 3606.4646 | 3607.94 | 3606.4594 | 667923.5 |
| 3786-3796 | Ar-pAr-pGr-pGr-pCr-pUr-pGr-pUr-pUr-pGr | Cyclic phosphorylation | 11 | 1.51 | 100 | 1 | 12.59 | 600.237 | -6 | 3606.4648 | 3607.94 | 3606.4594 | 1240762.25 |
| 3189-3200 | Gr-pGr-pCr-pUr-pAr-pCr-pAr-pCr-pCr-pCr-pUr-pGr | Cyclic phosphorylation | 12 | 2.3 | 97.9 | 1 | 12.66 | 1276.164 | -3 | 3830.5132 | 3832.21 | 3830.5044 | 265086.44 |
| 3622-3632 | Cr-pCr-pAr-pGr-pAr-pAr-pUr-pCr-pUr-pGr | Cyclic phosphorylation | 11 | 2.44 | 98.5 | 1.1 | 12.71 | 588.074 | -6 | 3533.488 | 3535.05 | 3533.4794 | 334088 |
| 3622-3632 | Cr-pCr-pAr-pGr-pAr-pAr-pUr-pCr-pUr-pGr | Cyclic phosphorylation | 11 | 2.1 | 98.5 | 1 | 12.75 | 705.89 | -5 | 3533.4868 | 3535.11 | 3533.4794 | 471865.22 |
| 495-503 | Ar-pAr-pCr-pAr-pAr-pCr-pAr-pAr-pGr | Cyclic phosphorylation | 9 | 0.65 | 99.9 | 1 | 12.82 | 585.083 | -5 | 2929.447 | 2930.63 | 2929.4451 | 2793015.5 |
| 3864-3874 | Cr-pAr-pCr-pUr-pAr-pAr-pCr-pAr-pUr-pGr | Cyclic phosphorylation | 11 | 1.08 | 100 | 1 | 12.83 | 581.404 | -6 | 3493.4771 | 3495.3 | 3493.4733 | 4879208.5 |
| 495-503 | Ar-pAr-pCr-pAr-pAr-pCr-pAr-pAr-pGr | Cyclic phosphorylation | 9 | 0.15 | 99.9 | 1 | 12.83 | 731.605 | -4 | 2929.4456 | 2930.61 | 2929.4451 | 4449021 |
| 495-503 | Ar-pAr-pCr-pAr-pAr-pCr-pAr-pAr-pGr | Cyclic phosphorylation | 9 | 0.4 | 99.9 | 1 | 12.85 | 1464.217 | -2 | 2929.4463 | 2930.58 | 2929.4451 | 4031693.75 |
| 3864-3874 | Cr-pAr-pCr-pUr-pAr-pAr-pCr-pAr-pUr-pGr | Cyclic phosphorylation | 11 | -0.31 | 100 | 1 | 12.85 | 697.886 | -5 | 3493.4722 | 3495.15 | 3493.4733 | 12890837 |
| 3864-3874 | Cr-pAr-pCr-pUr-pAr-pAr-pCr-pAr-pUr-pGr | Cyclic phosphorylation | 11 | 0.45 | 100 | 1 | 12.85 | 872.61 | -4 | 3493.4749 | 3495.14 | 3493.4733 | 6178547.5 |
| 3864-3874 | Cr-pAr-pCr-pUr-pAr-pAr-pCr-pAr-pUr-pGr | Cyclic phosphorylation | 11 | 0.73 | 100 | 1 | 12.85 | 1163.817 | -3 | 3493.4758 | 3495.19 | 3493.4733 | 5687010.5 |
| 495-503 | Ar-pAr-pCr-pAr-pAr-pCr-pAr-pAr-pGr | Cyclic phosphorylation | 9 | 0.9 | 99.9 | 1 | 12.85 | 975.809 | -3 | 2929.4478 | 2930.59 | 2929.4451 | 1728807.12 |
| 3864-3874 | Cr-pAr-pCr-pUr-pAr-pAr-pCr-pAr-pUr-pGr | Cyclic phosphorylation | 11 | 1.15 | 100 | 1 | 12.86 | 1746.23 | -2 | 3493.4773 | 3495.21 | 3493.4733 | 5245899 |
| 3846-3857 | Cr-pUr-pGr-pAr-pAr-pGr-pGr-pGr-pCr-pGr-pUr-pGr | Cyclic phosphorylation | 12 | 2.26 | 99.5 | 1.2 | 12.92 | 657.581 | -6 | 3950.5317 | 3952.14 | 3950.5228 | 683813.69 |
| 3846-3857 | Cr-pUr-pGr-pAr-pAr-pGr-pGr-pGr-pCr-pGr-pUr-pGr | Cyclic phosphorylation | 12 | 3 | 99.5 | 1 | 12.92 | 789.3 | -5 | 3950.5347 | 3952.09 | 3950.5228 | 544719.88 |
| 1200-1211 | Ur-pCr-pCr-pCr-pUr-pAr-pCr-pCr-pAr-pAr-pGr | Cyclic phosphorylation | 12 | 1.58 | 100 | 1 | 13.07 | 1886.748 | -2 | 3774.5093 | 3776.1 | 3774.5033 | 959319.62 |
| 1200-1211 | Ur-pCr-pCr-pCr-pUr-pAr-pCr-pCr-pAr-pAr-pGr | Cyclic phosphorylation | 12 | 0.61 | 100 | 1 | 13.07 | 754.094 | -5 | 3774.5056 | 3776.06 | 3774.5033 | 2657359.25 |
| 1200-1211 | Ur-pCr-pCr-pCr-pUr-pAr-pCr-pCr-pAr-pAr-pGr | Cyclic phosphorylation | 12 | 1.32 | 100 | 1 | 13.07 | 942.87 | -4 | 3774.5083 | 3776.09 | 3774.5033 | 1201546.88 |
| 1200-1211 | Ur-pCr-pCr-pCr-pUr-pAr-pCr-pCr-pAr-pAr-pGr | Cyclic phosphorylation | 12 | 0.54 | 100 | 1 | 13.07 | 628.244 | -6 | 3774.5054 | 3776.07 | 3774.5033 | 2854077.75 |
| 1200-1211 | Ur-pCr-pCr-pCr-pUr-pAr-pCr-pCr-pAr-pAr-pGr | Cyclic phosphorylation | 12 | 1.06 | 100 | 1 | 13.07 | 1257.496 | -3 | 3774.5073 | 3776.08 | 3774.5033 | 2095932.25 |
| 1643-1653 | Cr-pAr-pCr-pCr-pAr-pAr-pUr-pCr-pUr-pCr-pGr | Phosphorylation | 11 | 1.49 | 99.3 | 1 | 13.07 | 696.689 | -5 | 3487.4778 | 3488.79 | 3487.4726 | 178369.36 |
| 2632-2645 | Ur-pGr-pCr-pUr-pGr-pCr-pCr-pUr-pCr-pCr-pUr-pCr-pUr-pGr | Cyclic phosphorylation | 14 | 0.9 | 95 | 1 | 13.09 | 731.747 | -6 | 4395.5205 | 4397.34 | 4395.5165 | 164042.25 |
| 2632-2645 | Ur-pGr-pCr-pUr-pGr-pCr-pCr-pUr-pCr-pCr-pUr-pCr-pUr-pGr | Cyclic phosphorylation | 14 | 0.57 | 95 | 1.1 | 13.09 | 1464.5 | -3 | 4395.519 | 4397.44 | 4395.5165 | 138180.84 |

| | | | | | | | | | | | | | |
|---|---|---|---|---|---|---|---|---|---|---|---|---|---|
| 2520-2529 | Ur-pUr-pCr-pAr-pAr-pCr-pAr-pAr-pAr-pGr | Phosphorylation | 10 | 1.76 | 100 | 1 | 13.18 | 806.858 | -4 | 3230.4595 | 3231.68 | 3230.4538 | 334259.91 |
| 2520-2529 | Ur-pUr-pCr-pAr-pAr-pCr-pAr-pAr-pAr-pGr | Phosphorylation | 10 | 1.46 | 100 | 1 | 13.18 | 1614.723 | -2 | 3230.4585 | 3231.87 | 3230.4538 | 355282.06 |
| 1084-1095 | Cr-pCr-pAr-pCr-pCr-pAr-pGr-pAr-pUr-pUr-pCr-pGr | Cyclic phosphorylation | 12 | 2.51 | 98.2 | 1 | 13.25 | 634.913 | -6 | 3814.519 | 3816.08 | 3814.5095 | 444002.72 |
| 1084-1095 | Cr-pCr-pAr-pCr-pCr-pAr-pGr-pAr-pUr-pUr-pCr-pGr | Cyclic phosphorylation | 12 | 2.83 | 98.2 | 1 | 13.26 | 762.097 | -5 | 3814.5203 | 3816.02 | 3814.5095 | 438358.44 |
| 303-315 | Cr-pUr-pGr-pCr-pCr-pCr-pUr-pUr-pCr-pAr-pAr-pCr-pGr | Cyclic phosphorylation | 13 | 1.64 | 100 | 1 | 13.32 | 1364.837 | -3 | 4096.5303 | 4098.23 | 4096.5235 | 843752.31 |
| 303-315 | Cr-pUr-pGr-pCr-pCr-pCr-pUr-pUr-pCr-pAr-pAr-pCr-pGr | Cyclic phosphorylation | 13 | 1.53 | 100 | 1 | 13.32 | 681.914 | -6 | 4096.5298 | 4098.25 | 4096.5235 | 571551.12 |
| 2382-2391 | Ar-pAr-pCr-pAr-pCr-pCr-pCr-pAr-pAr-pGr | Cyclic phosphorylation | 10 | 1.04 | 100 | 1 | 13.32 | 1604.733 | -2 | 3210.4785 | 3211.85 | 3210.4752 | 785889.06 |
| 2382-2391 | Ar-pAr-pCr-pAr-pCr-pCr-pCr-pAr-pAr-pGr | Cyclic phosphorylation | 10 | 1.8 | 100 | 1 | 13.32 | 1069.487 | -3 | 3210.481 | 3211.88 | 3210.4752 | 305120 |
| 2382-2391 | Ar-pAr-pCr-pAr-pCr-pCr-pCr-pAr-pAr-pGr | Cyclic phosphorylation | 10 | 0.58 | 100 | 1 | 13.32 | 641.289 | -5 | 3210.4771 | 3211.71 | 3210.4752 | 1048486.62 |
| 2382-2391 | Ar-pAr-pCr-pAr-pCr-pCr-pCr-pAr-pAr-pGr | Cyclic phosphorylation | 10 | 1.11 | 100 | 1 | 13.32 | 801.863 | -4 | 3210.4788 | 3211.88 | 3210.4752 | 660049.5 |
| 4059-4071 | Cr-pCr-pAr-pCr-pAr-pCr-pCr-pCr-pCr-pCr-pAr-pCr-pGr | Cyclic phosphorylation | 13 | 1.46 | 100 | 1.3 | 13.45 | 581.648 | -7 | 4077.5825 | 4079.25 | 4077.5766 | 627847.75 |
| 4059-4071 | Cr-pCr-pAr-pCr-pAr-pCr-pCr-pCr-pCr-pCr-pAr-pCr-pGr | Cyclic phosphorylation | 13 | 1.1 | 100 | 1 | 13.46 | 1358.52 | -3 | 4077.5811 | 4079.21 | 4077.5766 | 1546965.62 |
| 4059-4071 | Cr-pCr-pAr-pCr-pAr-pCr-pCr-pCr-pCr-pCr-pAr-pCr-pGr | Cyclic phosphorylation | 13 | 0.74 | 100 | 1 | 13.46 | 678.756 | -6 | 4077.5796 | 4079.25 | 4077.5766 | 1937063.75 |
| 4059-4071 | Cr-pCr-pAr-pCr-pAr-pCr-pCr-pCr-pCr-pCr-pAr-pCr-pGr | Cyclic phosphorylation | 13 | 1.4 | 100 | 1 | 13.46 | 1018.639 | -4 | 4077.5823 | 4079.25 | 4077.5766 | 590852.94 |
| 4059-4071 | Cr-pCr-pAr-pCr-pAr-pCr-pCr-pCr-pCr-pCr-pAr-pCr-pGr | Cyclic phosphorylation | 13 | 1.04 | 100 | 1 | 13.47 | 814.709 | -5 | 4077.5808 | 4079.28 | 4077.5766 | 1364093.12 |
| 303-315 | Cr-pUr-pGr-pCr-pCr-pCr-pUr-pUr-pCr-pAr-pAr-pCr-pGr | Cyclic phosphorylation | 13 | 1.17 | 99.9 | 1 | 13.53 | 682.08 | -6 | 4096.5283 | 4098.77 | 4096.5235 | 1083678.12 |
| 1941-1951 | Ar-pCr-pAr-pCr-pCr-pUr-pAr-pCr-pAr-pUr-pGr | Cyclic phosphorylation | 11 | 0.73 | 100 | 1 | 13.59 | 872.612 | -4 | 3493.4758 | 3494.91 | 3493.4733 | 1879347 |
| 1941-1951 | Ar-pCr-pAr-pCr-pCr-pUr-pAr-pCr-pAr-pUr-pGr | Cyclic phosphorylation | 11 | 0.52 | 100 | 1 | 13.59 | 697.888 | -5 | 3493.4751 | 3494.93 | 3493.4733 | 4170996 |
| 1941-1951 | Ar-pCr-pAr-pCr-pCr-pUr-pAr-pCr-pAr-pUr-pGr | Cyclic phosphorylation | 11 | 0.73 | 100 | 1 | 13.59 | 1163.819 | -3 | 3493.4758 | 3494.99 | 3493.4733 | 2324717 |
| 1941-1951 | Ar-pCr-pAr-pCr-pCr-pUr-pAr-pCr-pAr-pUr-pGr | Cyclic phosphorylation | 11 | 1.15 | 100 | 1 | 13.59 | 1746.233 | -2 | 3493.4773 | 3494.94 | 3493.4733 | 2192160.75 |
| 3453-3462 | Ar-pAr-pCr-pAr-pAr-pUr-pAr-pCr-pCr-pGr | Cyclic phosphorylation | 10 | 0.85 | 100 | 1 | 13.61 | 1069.814 | -3 | 3211.4619 | 3212.84 | 3211.4592 | 2371335.25 |
| 3453-3462 | Ar-pAr-pCr-pAr-pAr-pUr-pAr-pCr-pCr-pGr | Cyclic phosphorylation | 10 | 0.09 | 100 | 1 | 13.61 | 641.485 | -5 | 3211.4595 | 3212.71 | 3211.4592 | 5700426.5 |
| 1941-1951 | Ar-pCr-pAr-pCr-pCr-pUr-pAr-pCr-pAr-pUr-pGr | Cyclic phosphorylation | 11 | 0.31 | 100 | 1 | 13.61 | 581.406 | -6 | 3493.4744 | 3494.94 | 3493.4733 | 4264086 |
| 3453-3462 | Ar-pAr-pCr-pAr-pAr-pUr-pAr-pCr-pCr-pGr | Cyclic phosphorylation | 10 | 0.31 | 100 | 1 | 13.61 | 802.108 | -4 | 3211.4602 | 3212.84 | 3211.4592 | 4236235.5 |
| 3453-3462 | Ar-pAr-pCr-pAr-pAr-pUr-pAr-pCr-pCr-pGr | Cyclic phosphorylation | 10 | 0.77 | 100 | 1 | 13.63 | 1605.224 | -2 | 3211.4617 | 3212.87 | 3211.4592 | 3771665.25 |
| 2041-2052 | Ar-pCr-pAr-pUr-pCr-pCr-pCr-pCr-pAr-pUr-pCr-pGr | Cyclic phosphorylation | 12 | 0.41 | 99.9 | 1 | 13.64 | 628.244 | -6 | 3774.5049 | 3775.85 | 3774.5033 | 5746526.5 |
| 2041-2052 | Ar-pCr-pAr-pUr-pCr-pCr-pCr-pCr-pAr-pUr-pCr-pGr | Cyclic phosphorylation | 12 | 0.74 | 99.9 | 1 | 13.64 | 1257.495 | -3 | 3774.5061 | 3776.08 | 3774.5033 | 3774156.25 |
| 2041-2052 | Ar-pCr-pAr-pUr-pCr-pCr-pCr-pCr-pAr-pUr-pCr-pGr | Cyclic phosphorylation | 12 | 0.54 | 99.9 | 1 | 13.64 | 754.094 | -5 | 3774.5054 | 3776 | 3774.5033 | 4687001 |
| 2041-2052 | Ar-pCr-pAr-pUr-pCr-pCr-pCr-pCr-pAr-pUr-pCr-pGr | Cyclic phosphorylation | 12 | 0.67 | 99.9 | 1 | 13.65 | 943.12 | -4 | 3774.5059 | 3776.07 | 3774.5033 | 1735729 |
| 2041-2052 | Ar-pCr-pAr-pUr-pCr-pCr-pCr-pCr-pAr-pUr-pCr-pGr | Cyclic phosphorylation | 12 | 1.19 | 99.9 | 1 | 13.66 | 1886.748 | -2 | 3774.5078 | 3776.32 | 3774.5033 | 1547731.12 |
| 3286-3298 | Cr-pUr-pCr-pCr-pAr-pGr-pCr-pCr-pAr-pUr-pCr-pUr-pGr | Cyclic phosphorylation | 13 | 1.53 | 100 | 1 | 13.78 | 584.354 | -7 | 4096.5298 | 4098.19 | 4096.5235 | 1278812.25 |
| 3286-3298 | Cr-pUr-pCr-pCr-pAr-pGr-pCr-pCr-pAr-pUr-pCr-pUr-pGr | Cyclic phosphorylation | 13 | 1.17 | 100 | 1 | 13.8 | 1364.836 | -3 | 4096.5283 | 4098.2 | 4096.5235 | 1291107.12 |
| 3286-3298 | Cr-pUr-pCr-pCr-pAr-pGr-pCr-pCr-pAr-pUr-pCr-pUr-pGr | Cyclic phosphorylation | 13 | 0.93 | 100 | 1 | 13.8 | 681.914 | -6 | 4096.5273 | 4098.21 | 4096.5235 | 1657847.38 |
| 3286-3298 | Cr-pUr-pCr-pCr-pAr-pGr-pCr-pCr-pAr-pUr-pCr-pUr-pGr | Cyclic phosphorylation | 13 | 1.41 | 100 | 1 | 13.8 | 818.499 | -5 | 4096.5293 | 4098.24 | 4096.5235 | 627599.94 |
| 2840-2852 | Cr-pGr-pCr-pCr-pAr-pUr-pCr-pGr-pGr-pCr-pAr-pAr-pGr | Cyclic phosphorylation | 13 | 1.3 | 100 | 1 | 13.94 | 838.91 | -5 | 4198.5845 | 4200.33 | 4198.579 | 1417164.12 |
| 2840-2852 | Cr-pGr-pCr-pCr-pAr-pUr-pCr-pGr-pGr-pCr-pAr-pAr-pGr | Cyclic phosphorylation | 13 | 1.3 | 100 | 1 | 13.94 | 1398.855 | -3 | 4198.5845 | 4200.47 | 4198.579 | 1478312.62 |
| 2840-2852 | Cr-pGr-pCr-pCr-pAr-pUr-pCr-pGr-pGr-pCr-pAr-pAr-pGr | Cyclic phosphorylation | 13 | 1.18 | 100 | 1 | 13.96 | 698.923 | -6 | 4198.584 | 4200.29 | 4198.579 | 1654331.75 |
| 3038-3048 | Ar-pCr-pUr-pGr-pAr-pUr-pCr-pAr-pCr-pAr-pGr | Cyclic phosphorylation | 11 | 1.54 | 100 | 1 | 14.03 | 588.073 | -6 | 3533.4849 | 3535.13 | 3533.4794 | 607196.81 |
| 3038-3048 | Ar-pCr-pUr-pGr-pAr-pUr-pCr-pAr-pCr-pAr-pGr | Cyclic phosphorylation | 11 | 1.96 | 100 | 1 | 14.03 | 705.89 | -5 | 3533.4863 | 3535.11 | 3533.4794 | 667914.88 |
| 3038-3048 | Ar-pCr-pUr-pGr-pAr-pUr-pCr-pAr-pCr-pAr-pGr | Cyclic phosphorylation | 11 | 1.2 | 100 | 1 | 14.04 | 1177.154 | -3 | 3533.4836 | 3535.08 | 3533.4794 | 374152.81 |
| 3038-3048 | Ar-pCr-pUr-pGr-pAr-pUr-pCr-pAr-pCr-pAr-pGr | Cyclic phosphorylation | 11 | 1.2 | 100 | 1 | 14.04 | 1766.235 | -2 | 3533.4836 | 3535.09 | 3533.4794 | 370181.69 |
| 3864-3874 | Cr-pAr-pCr-pUr-pAr-pCr-pAr-pCr-pAr-pUr-pGr | Phosphorylation | 11 | 1.55 | 100 | 1 | 14.04 | 877.115 | -4 | 3511.4893 | 3513.17 | 3511.4838 | 541403.75 |
| 3864-3874 | Cr-pAr-pCr-pUr-pAr-pCr-pAr-pCr-pAr-pUr-pGr | Phosphorylation | 11 | 1.34 | 100 | 1 | 14.04 | 1755.237 | -2 | 3511.4885 | 3513.19 | 3511.4838 | 367614.94 |
| 3441-3452 | Ar-pUr-pCr-pGr-pGr-pCr-pAr-pUr-pUr-pGr-pUr-pGr | Cyclic phosphorylation | 12 | 1.88 | 100 | 1 | 14.22 | 644.574 | -6 | 3872.4858 | 3874.05 | 3872.4786 | 654554.69 |
| 3441-3452 | Ar-pUr-pCr-pGr-pGr-pCr-pAr-pUr-pUr-pGr-pUr-pGr | Cyclic phosphorylation | 12 | 1.38 | 100 | 1 | 14.22 | 1290.155 | -3 | 3872.4839 | 3873.88 | 3872.4786 | 360325.47 |
| 3038-3048 | Ar-pCr-pUr-pGr-pAr-pUr-pCr-pAr-pCr-pAr-pGr | Cyclic phosphorylation | 11 | 1.54 | 98.7 | 1 | 14.24 | 705.89 | -5 | 3533.4849 | 3534.97 | 3533.4794 | 303883.41 |
| 3042-3052 | Ar-pUr-pCr-pAr-pCr-pAr-pGr-pGr-pCr-pAr-pGr | Cyclic phosphorylation | 11 | 1.48 | 100 | 1 | 14.25 | 713.694 | -5 | 3572.5068 | 3574.04 | 3572.5015 | 1017673.62 |
| 3042-3052 | Ar-pUr-pCr-pAr-pCr-pAr-pGr-pGr-pCr-pAr-pGr | Cyclic phosphorylation | 11 | 1.62 | 100 | 1 | 14.25 | 594.578 | -6 | 3572.5073 | 3574 | 3572.5015 | 487839.5 |
| 3042-3052 | Ar-pUr-pCr-pAr-pCr-pAr-pGr-pGr-pCr-pAr-pGr | Cyclic phosphorylation | 11 | 1.75 | 100 | 1 | 14.25 | 1190.163 | -3 | 3572.5078 | 3574 | 3572.5015 | 319346.81 |
| 3038-3048 | Ar-pCr-pUr-pGr-pAr-pUr-pCr-pAr-pCr-pAr-pGr | Cyclic phosphorylation | 11 | 1.82 | 98.7 | 1 | 14.25 | 1766.236 | -2 | 3533.4858 | 3535.02 | 3533.4794 | 177148.3 |
| 2634-2648 | Cr-pUr-pGr-pCr-pCr-pUr-pCr-pCr-pUr-pCr-pUr-pGr-pCr-pUr-pG | Cyclic phosphorylation | 15 | 1.52 | 99.8 | 1 | 14.27 | 782.587 | -6 | 4700.5649 | 4702.5 | 4700.5578 | 216574.53 |
| 3078-3089 | Ar-pCr-pCr-pCr-pAr-pGr-pCr-pAr-pGr-pCr-pUr-pGr | Cyclic phosphorylation | 12 | 1.68 | 100 | 1 | 14.29 | 1283.84 | -3 | 3853.5381 | 3855.14 | 3853.5316 | 804010.75 |
| 3078-3089 | Ar-pCr-pCr-pCr-pAr-pGr-pCr-pAr-pGr-pCr-pUr-pGr | Cyclic phosphorylation | 12 | 1.11 | 100 | 1 | 14.29 | 641.416 | -6 | 3853.5359 | 3855.17 | 3853.5316 | 1213937.25 |
| 1001-1013 | Cr-pAr-pAr-pCr-pUr-pUr-pCr-pCr-pGr-pGr-pGr-pUr-pGr | Cyclic phosphorylation | 13 | 0.42 | 99.9 | 1 | 14.32 | 695.25 | -6 | 4176.5376 | 4178.46 | 4176.5358 | 1308318.25 |
| 1001-1013 | Cr-pAr-pAr-pCr-pUr-pUr-pCr-pCr-pGr-pGr-pGr-pUr-pGr | Cyclic phosphorylation | 13 | 2.06 | 99.9 | 1 | 14.34 | 834.702 | -5 | 4176.5444 | 4178.59 | 4176.5358 | 632647.5 |
| 4013-4024 | Ar-pCr-pAr-pCr-pCr-pUr-pCr-pCr-pCr-pAr-pAr-pGr | Cyclic phosphorylation | 12 | 1.8 | 100 | 1 | 14.34 | 1898.26 | -2 | 3797.5364 | 3799.52 | 3797.5305 | 1792128.38 |
| 1001-1013 | Cr-pAr-pAr-pCr-pUr-pUr-pCr-pCr-pGr-pGr-pGr-pUr-pGr | Cyclic phosphorylation | 13 | 1.48 | 99.9 | 1 | 14.34 | 1391.506 | -3 | 4176.542 | 4178.49 | 4176.5358 | 989425.38 |
| 1001-1013 | Cr-pAr-pAr-pCr-pUr-pUr-pCr-pCr-pGr-pGr-pGr-pUr-pGr | Cyclic phosphorylation | 13 | 0.66 | 99.9 | 1.1 | 14.34 | 595.784 | -7 | 4176.5386 | 4178.51 | 4176.5358 | 669393.56 |
| 4013-4024 | Ar-pCr-pAr-pCr-pCr-pUr-pCr-pCr-pCr-pAr-pAr-pGr | Cyclic phosphorylation | 12 | 1.22 | 100 | 1 | 14.35 | 1265.169 | -3 | 3797.5352 | 3799.37 | 3797.5305 | 3818827 |
| 4013-4024 | Ar-pCr-pAr-pCr-pCr-pUr-pCr-pCr-pCr-pAr-pAr-pGr | Cyclic phosphorylation | 12 | 0.96 | 100 | 1 | 14.35 | 632.08 | -6 | 3797.5342 | 3799.51 | 3797.5305 | 6464786 |
| 4013-4024 | Ar-pCr-pAr-pCr-pCr-pUr-pCr-pCr-pCr-pAr-pAr-pGr | Cyclic phosphorylation | 12 | 0.96 | 100 | 1 | 14.35 | 758.698 | -5 | 3797.5342 | 3799.5 | 3797.5305 | 4855531.5 |
| 4013-4024 | Ar-pCr-pAr-pCr-pCr-pUr-pCr-pCr-pCr-pAr-pAr-pGr | Cyclic phosphorylation | 12 | 1.54 | 100 | 1 | 14.36 | 948.626 | -4 | 3797.5364 | 3799.48 | 3797.5305 | 2194237.5 |
| 3-12 | Ar-pAr-pUr-pAr-pAr-pAr-pCr-pUr-pAr-pGr | Cyclic phosphorylation | 10 | -0.56 | 100 | 1 | 14.49 | 646.483 | -5 | 3236.4526 | 3237.83 | 3236.4544 | 10479132 |
| 3-12 | Ar-pAr-pUr-pAr-pAr-pAr-pCr-pUr-pAr-pGr | Cyclic phosphorylation | 10 | 0.87 | 100 | 1 | 14.49 | 1078.146 | -3 | 3236.4573 | 3237.86 | 3236.4544 | 3081932.5 |
| 1991-2003 | Ar-pGr-pCr-pCr-pGr-pGr-pCr-pUr-pGr-pUr-pCr-pUr-pGr | Cyclic phosphorylation | 13 | 1.05 | 100 | 1 | 14.49 | 1396.838 | -3 | 4192.5352 | 4194.29 | 4192.5307 | 898813.06 |
| 1991-2003 | Ar-pGr-pCr-pCr-pGr-pGr-pCr-pUr-pGr-pUr-pCr-pUr-pGr | Cyclic phosphorylation | 13 | 1.17 | 100 | 1 | 14.49 | 837.7 | -5 | 4192.5356 | 4194.24 | 4192.5307 | 891980.62 |
| 1991-2003 | Ar-pGr-pCr-pCr-pGr-pGr-pCr-pUr-pGr-pUr-pCr-pUr-pGr | Cyclic phosphorylation | 13 | 0.82 | 100 | 1 | 14.49 | 697.916 | -6 | 4192.5342 | 4194.34 | 4192.5307 | 1073382.5 |
| 3-12 | Ar-pAr-pUr-pAr-pAr-pAr-pCr-pUr-pAr-pGr | Cyclic phosphorylation | 10 | 0.42 | 100 | 1 | 14.49 | 1617.722 | -2 | 3236.4558 | 3237.86 | 3236.4544 | 6528939.5 |
| 3-12 | Ar-pAr-pUr-pAr-pAr-pAr-pCr-pUr-pAr-pGr | Cyclic phosphorylation | 10 | 0.65 | 100 | 1 | 14.49 | 538.569 | -6 | 3236.4565 | 3237.83 | 3236.4544 | 1444558.12 |
| 3-12 | Ar-pAr-pUr-pAr-pAr-pAr-pCr-pUr-pAr-pGr | Cyclic phosphorylation | 10 | 0.42 | 100 | 1 | 14.49 | 808.357 | -4 | 3236.4558 | 3237.86 | 3236.4544 | 4541060 |
| 1856-1867 | Cr-pAr-pCr-pCr-pAr-pAr-pCr-pAr-pCr-pCr-pAr-pGr | Cyclic phosphorylation | 12 | 1.37 | 100 | 1 | 14.6 | 1272.848 | -3 | 3820.563 | 3822.17 | 3820.5578 | 848558.94 |
| 1856-1867 | Cr-pAr-pCr-pCr-pAr-pAr-pCr-pAr-pCr-pCr-pAr-pGr | Cyclic phosphorylation | 12 | 1.5 | 100 | 1 | 14.61 | 954.384 | -4 | 3820.5635 | 3822.14 | 3820.5578 | 578400.38 |
| 1856-1867 | Cr-pAr-pCr-pCr-pAr-pAr-pCr-pAr-pCr-pCr-pAr-pGr | Cyclic phosphorylation | 12 | 1.24 | 100 | 1 | 14.61 | 763.305 | -5 | 3820.5625 | 3822.15 | 3820.5578 | 1354485.25 |
| 1856-1867 | Cr-pAr-pCr-pCr-pAr-pAr-pCr-pAr-pCr-pCr-pAr-pGr | Cyclic phosphorylation | 12 | 1.37 | 100 | 1 | 14.61 | 635.92 | -6 | 3820.563 | 3822.16 | 3820.5578 | 985251.38 |
| 3806-3818 | Cr-pAr-pGr-pCr-pUr-pGr-pCr-pUr-pGr-pCr-pAr-pAr-pGr | Cyclic phosphorylation | 13 | 1.5 | 97 | 1 | 14.63 | 839.107 | -5 | 4199.5693 | 4201.29 | 4199.563 | 496838.06 |
| 1810-1822 | Ar-pCr-pAr-pUr-pCr-pAr-pCr-pCr-pCr-pCr-pUr-pUr-pGr | Cyclic phosphorylation | 13 | 0.17 | 100 | 1 | 14.79 | 679.248 | -6 | 4080.5293 | 4082.16 | 4080.5286 | 6919271 |
| 1810-1822 | Ar-pCr-pAr-pUr-pCr-pAr-pCr-pCr-pCr-pCr-pUr-pUr-pGr | Cyclic phosphorylation | 13 | 1 | 100 | 1 | 14.79 | 1359.504 | -3 | 4080.5327 | 4082.2 | 4080.5286 | 4731708 |
| 1810-1822 | Ar-pCr-pAr-pUr-pCr-pAr-pCr-pCr-pCr-pCr-pUr-pUr-pGr | Cyclic phosphorylation | 13 | 1.12 | 100 | 1 | 14.79 | 815.299 | -5 | 4080.5332 | 4082.17 | 4080.5286 | 3200567.75 |
| 1810-1822 | Ar-pCr-pAr-pUr-pCr-pAr-pCr-pCr-pCr-pCr-pUr-pUr-pGr | Cyclic phosphorylation | 13 | 1 | 100 | 1 | 14.79 | 582.069 | -7 | 4080.5327 | 4082.22 | 4080.5286 | 3231536.5 |
| 1810-1822 | Ar-pCr-pAr-pUr-pCr-pAr-pCr-pCr-pCr-pCr-pUr-pUr-pGr | Cyclic phosphorylation | 13 | 1.6 | 100 | 1 | 14.8 | 1019.628 | -4 | 4080.5352 | 4082.25 | 4080.5286 | 1467982.75 |
| 1823-1836 | Cr-pAr-pGr-pCr-pUr-pUr-pCr-pGr-pGr-pCr-pGr-pGr-pAr-pGr | Cyclic phosphorylation | 14 | 1.62 | 100 | 1 | 14.89 | 759.262 | -6 | 4560.6128 | 4562.35 | 4560.6054 | 981680.31 |
| 1823-1836 | Cr-pAr-pGr-pCr-pUr-pUr-pCr-pGr-pGr-pCr-pGr-pGr-pAr-pGr | Cyclic phosphorylation | 14 | 1.62 | 100 | 1 | 14.89 | 650.652 | -7 | 4560.6128 | 4562.58 | 4560.6054 | 855440.75 |
| 1823-1836 | Cr-pAr-pGr-pCr-pUr-pUr-pCr-pGr-pGr-pCr-pGr-pGr-pAr-pGr | Cyclic phosphorylation | 14 | 1.19 | 100 | 1 | 14.89 | 1519.865 | -3 | 4560.6108 | 4562.63 | 4560.6054 | 741861.25 |
| 1703-1715 | Cr-pGr-pUr-pGr-pCr-pUr-pGr-pAr-pCr-pAr-pGr-pAr-pGr | Cyclic phosphorylation | 13 | 1.3 | 100 | 1 | 14.94 | 1412.518 | -3 | 4239.5747 | 4241.29 | 4239.5692 | 662016.31 |
| 1703-1715 | Cr-pGr-pUr-pGr-pCr-pUr-pGr-pAr-pCr-pAr-pGr-pAr-pGr | Cyclic phosphorylation | 13 | 1.07 | 100 | 1 | 14.96 | 705.756 | -6 | 4239.5737 | 4241.21 | 4239.5692 | 829993.19 |
| 1624-1637 | Ur-pGr-pUr-pGr-pCr-pGr-pGr-pCr-pCr-pCr-pUr-pAr-pAr-pGr | Cyclic phosphorylation | 14 | 1.35 | 100 | 1 | 15.05 | 752.925 | -6 | 4521.5894 | 4523.53 | 4521.5833 | 972879.88 |
| 567-581 | Ur-pCr-pCr-pCr-pAr-pGr-pCr-pCr-pUr-pUr-pUr-pCr-pCr-pUr-pGr | Cyclic phosphorylation | 15 | 1.58 | 100 | 1 | 15.05 | 584.815 | -8 | 4684.5703 | 4686.55 | 4684.5629 | 1256342.88 |
| 567-581 | Ur-pCr-pCr-pCr-pAr-pGr-pCr-pCr-pUr-pUr-pUr-pCr-pCr-pUr-pGr | Cyclic phosphorylation | 15 | 1.06 | 100 | 1 | 15.06 | 668.503 | -7 | 4684.5679 | 4686.6 | 4684.5629 | 3232884 |
| 567-581 | Ur-pCr-pCr-pCr-pAr-pGr-pCr-pCr-pUr-pUr-pUr-pCr-pCr-pUr-pGr | Cyclic phosphorylation | 15 | 1.27 | 100 | 1 | 15.06 | 1561.183 | -3 | 4684.5688 | 4686.58 | 4684.5629 | 2245691.75 |
| 567-581 | Ur-pCr-pCr-pCr-pAr-pGr-pCr-pCr-pUr-pUr-pUr-pCr-pCr-pUr-pGr | Cyclic phosphorylation | 15 | 1.69 | 100 | 1 | 15.06 | 1170.636 | -4 | 4684.5708 | 4686.52 | 4684.5629 | 552302.62 |
| 567-581 | Ur-pCr-pCr-pCr-pAr-pGr-pCr-pCr-pUr-pUr-pUr-pCr-pCr-pUr-pGr | Cyclic phosphorylation | 15 | 1.37 | 100 | 1 | 15.08 | 780.088 | -6 | 4684.5693 | 4686.58 | 4684.5629 | 1716924.88 |
| 567-581 | Ur-pCr-pCr-pCr-pAr-pGr-pCr-pCr-pUr-pUr-pUr-pCr-pCr-pUr-pGr | Cyclic phosphorylation | 15 | 1.69 | 100 | 1.1 | 15.08 | 936.107 | -5 | 4684.5708 | 4686.57 | 4684.5629 | 676322.5 |
| 3675-3686 | Cr-pAr-pGr-pUr-pAr-pCr-pAr-pUr-pCr-pAr-pAr-pGr | Cyclic phosphorylation | 12 | 1.85 | 99.7 | 1 | 15.19 | 1286.84 | -3 | 3862.5391 | 3864.1 | 3862.5319 | 242383.34 |
| 3675-3686 | Cr-pAr-pGr-pUr-pAr-pCr-pAr-pUr-pCr-pAr-pAr-pGr | Cyclic phosphorylation | 12 | 0.9 | 99.7 | 1 | 15.2 | 771.901 | -5 | 3862.5354 | 3864.19 | 3862.5319 | 250012.14 |
| 3675-3686 | Cr-pAr-pGr-pUr-pAr-pCr-pAr-pUr-pCr-pAr-pAr-pGr | Cyclic phosphorylation | 12 | 1.78 | 99.7 | 1 | 15.2 | 642.916 | -6 | 3862.5388 | 3864.12 | 3862.5319 | 333147.41 |
| 3350-3363 | Cr-pAr-pCr-pCr-pCr-pAr-pUr-pUr-pGr-pGr-pUr-pUr-pCr-pGr | Cyclic phosphorylation | 14 | 3.01 | 100 | 1.2 | 15.25 | 633.788 | -7 | 4442.5684 | 4444.51 | 4442.555 | 424240.41 |
| 3350-3363 | Cr-pAr-pCr-pCr-pCr-pAr-pUr-pUr-pGr-pGr-pUr-pUr-pCr-pGr | Cyclic phosphorylation | 14 | 1.25 | 100 | 1 | 15.25 | 739.754 | -6 | 4442.5605 | 4444.53 | 4442.555 | 667917 |
| 3350-3363 | Cr-pAr-pCr-pCr-pCr-pAr-pUr-pUr-pGr-pGr-pUr-pUr-pCr-pGr | Cyclic phosphorylation | 14 | 1.03 | 100 | 1 | 15.25 | 1480.18 | -3 | 4442.5596 | 4444.51 | 4442.555 | 588492.75 |
| 4085-4096 | Ar-pUr-pUr-pAr-pAr-pCr-pCr-pUr-pUr-pUr-pAr-pGr | Cyclic phosphorylation | 12 | 1.21 | 100 | 1 | 15.32 | 1900.23 | -2 | 3801.4712 | 3803.04 | 3801.4666 | 1770286.5 |
| 4085-4096 | Ar-pUr-pUr-pAr-pAr-pCr-pCr-pUr-pUr-pUr-pAr-pGr | Cyclic phosphorylation | 12 | 0.76 | 100 | 1 | 15.32 | 1266.483 | -3 | 3801.4695 | 3803.06 | 3801.4666 | 4503565.5 |
| 4085-4096 | Ar-pUr-pUr-pAr-pAr-pCr-pCr-pUr-pUr-pUr-pAr-pGr | Cyclic phosphorylation | 12 | 0.18 | 100 | 1 | 15.32 | 759.486 | -5 | 3801.4673 | 3803 | 3801.4666 | 4936272 |
| 4085-4096 | Ar-pUr-pUr-pAr-pAr-pCr-pCr-pUr-pUr-pUr-pAr-pGr | Cyclic phosphorylation | 12 | -0.01 | 100 | 1 | 15.32 | 632.737 | -6 | 3801.4666 | 3803.03 | 3801.4666 | 8751588 |
| 4085-4096 | Ar-pUr-pUr-pAr-pAr-pCr-pCr-pUr-pUr-pUr-pAr-pGr | Cyclic phosphorylation | 12 | 0.82 | 100 | 1 | 15.32 | 949.611 | -4 | 3801.4697 | 3803.08 | 3801.4666 | 1769663.25 |
| 4085-4096 | Ar-pUr-pUr-pAr-pAr-pCr-pCr-pUr-pUr-pUr-pAr-pGr | Cyclic phosphorylation | 12 | 0.89 | 100 | 1 | 15.32 | 542.203 | -7 | 3801.47 | 3803.01 | 3801.4666 | 1482550.25 |
| 1059-1073 | Ur-pGr-pCr-pCr-pCr-pCr-pUr-pUr-pCr-pGr-pGr-pCr-pGr-pAr-pG | Cyclic phosphorylation | 15 | 0.91 | 100 | 1 | 15.39 | 959.716 | -5 | 4802.6177 | 4804.65 | 4802.6133 | 842026.19 |
| 1059-1073 | Ur-pGr-pCr-pCr-pCr-pCr-pUr-pUr-pCr-pGr-pGr-pCr-pGr-pAr-pG | Cyclic phosphorylation | 15 | 1.21 | 100 | 1 | 15.41 | 685.224 | -7 | 4802.6191 | 4804.49 | 4802.6133 | 1837364.62 |
| 1059-1073 | Ur-pGr-pCr-pCr-pCr-pCr-pUr-pUr-pCr-pGr-pGr-pCr-pGr-pAr-pG | Cyclic phosphorylation | 15 | 0.91 | 100 | 1 | 15.41 | 799.763 | -6 | 4802.6177 | 4804.66 | 4802.6133 | 1771154.62 |
| 3490-3501 | Ar-pCr-pAr-pGr-pCr-pUr-pUr-pCr-pAr-pAr-pAr-pGr | Cyclic phosphorylation | 12 | 0.58 | 99.7 | 1 | 15.41 | 642.916 | -6 | 3862.5342 | 3864.18 | 3862.5319 | 1654083.62 |
| 1059-1073 | Ur-pGr-pCr-pCr-pCr-pCr-pUr-pUr-pCr-pGr-pGr-pCr-pGr-pAr-pG | Cyclic phosphorylation | 15 | 0.81 | 100 | 1 | 15.41 | 1600.533 | -3 | 4802.6172 | 4804.71 | 4802.6133 | 1457394.25 |
| 3490-3501 | Ar-pCr-pAr-pGr-pCr-pUr-pUr-pCr-pAr-pAr-pAr-pGr | Cyclic phosphorylation | 12 | 0.52 | 99.7 | 1 | 15.41 | 771.7 | -5 | 3862.5339 | 3864.06 | 3862.5319 | 889877.25 |
| 3490-3501 | Ar-pCr-pAr-pGr-pCr-pUr-pUr-pCr-pAr-pAr-pAr-pGr | Cyclic phosphorylation | 12 | 1.09 | 99.7 | 1 | 15.41 | 1286.839 | -3 | 3862.5361 | 3864.17 | 3862.5319 | 934923.31 |
| 963-975 | Ar-pAr-pGr-pUr-pCr-pCr-pUr-pUr-pCr-pAr-pCr-pCr-pGr | Cyclic phosphorylation | 13 | 1.99 | 94.4 | 1 | 15.41 | 685.917 | -6 | 4120.543 | 4122.15 | 4120.5348 | 371357.06 |
| 3-12 | Ar-pAr-pUr-pAr-pAr-pAr-pCr-pUr-pAr-pGr | Phosphorylation | 10 | 0.48 | 100 | 1 | 15.48 | 812.86 | -4 | 3254.4666 | 3255.86 | 3254.465 | 1735929.5 |
| 3-12 | Ar-pAr-pUr-pAr-pAr-pAr-pCr-pUr-pAr-pGr | Phosphorylation | 10 | 1 | 100 | 1 | 15.48 | 1626.728 | -2 | 3254.4683 | 3255.87 | 3254.465 | 1537278 |
| 3-12 | Ar-pAr-pUr-pAr-pAr-pAr-pCr-pUr-pAr-pGr | Phosphorylation | 10 | 1.15 | 100 | 1 | 15.48 | 650.087 | -5 | 3254.4688 | 3255.77 | 3254.465 | 975006.69 |
| 3898-3910 | Cr-pAr-pCr-pGr-pCr-pAr-pAr-pUr-pGr-pCr-pUr-pAr-pGr | Cyclic phosphorylation | 13 | 0.99 | 93.1 | 1 | 15.48 | 696.422 | -6 | 4183.5723 | 4185.32 | 4183.5681 | 1216166.5 |
| 133-145 | Cr-pCr-pUr-pAr-pCr-pAr-pCr-pCr-pAr-pAr-pCr-pAr-pGr | Cyclic phosphorylation | 13 | 1.05 | 100 | 1 | 15.54 | 824.51 | -5 | 4126.5874 | 4128.26 | 4126.5831 | 866538.88 |

| | | | | | | | | | | | | |
|---|---|---|---|---|---|---|---|---|---|---|---|---|
| 133-145 | Cr-pCr-pUr-pAr-pCr-pAr-pCr-pCr-pAr-pAr-pCr-pAr-pGr | Cyclic phosphorylation | 13 | 0.82 | 100 | 1 | 15.55 | 1374.856 | -3 | 4126.5864 | 4128.26 | 4126.5831 | 1044761.19 |
| 133-145 | Cr-pCr-pUr-pAr-pCr-pAr-pCr-pCr-pAr-pAr-pCr-pAr-pGr | Cyclic phosphorylation | 13 | 1.53 | 100 | 1 | 15.55 | 686.924 | -6 | 4126.5894 | 4128.2 | 4126.5831 | 1444808.25 |
| 133-145 | Cr-pCr-pUr-pAr-pCr-pAr-pCr-pCr-pAr-pAr-pCr-pAr-pGr | Cyclic phosphorylation | 13 | 1.41 | 100 | 1.1 | 15.58 | 588.649 | -7 | 4126.5889 | 4128.71 | 4126.5831 | 494411.84 |
| 3525-3535 | Ar-pAr-pCr-pCr-pAr-pCr-pAr-pCr-pAr-pAr-pGr | Cyclic phosphorylation | 11 | 0.45 | 100 | 1 | 15.62 | 589.081 | -6 | 3539.5293 | 3541.02 | 3539.5277 | 5117848.5 |
| 3525-3535 | Ar-pAr-pCr-pCr-pAr-pCr-pAr-pCr-pAr-pAr-pGr | Cyclic phosphorylation | 11 | 1.35 | 100 | 1 | 15.62 | 1769.26 | -2 | 3539.5325 | 3541.03 | 3539.5277 | 3886048 |
| 3525-3535 | Ar-pAr-pCr-pCr-pAr-pCr-pAr-pCr-pAr-pAr-pGr | Cyclic phosphorylation | 11 | -0.31 | 100 | 1 | 15.62 | 707.098 | -5 | 3539.5266 | 3541.01 | 3539.5277 | 7854143 |
| 3525-3535 | Ar-pAr-pCr-pCr-pAr-pCr-pAr-pCr-pAr-pAr-pGr | Cyclic phosphorylation | 11 | 1 | 100 | 1 | 15.62 | 1179.17 | -3 | 3539.5312 | 3541.03 | 3539.5277 | 3493582 |
| 1810-1822 | Ar-pCr-pAr-pUr-pCr-pAr-pCr-pCr-pCr-pCr-pUr-pUr-pGr | Phosphorylation | 13 | 0.69 | 95.8 | 1 | 15.63 | 818.902 | -5 | 4098.542 | 4100.14 | 4098.5392 | 426656.94 |
| 3525-3535 | Ar-pAr-pCr-pCr-pAr-pCr-pAr-pCr-pAr-pAr-pGr | Cyclic phosphorylation | 11 | 0.52 | 100 | 1 | 15.63 | 884.126 | -4 | 3539.5295 | 3541.02 | 3539.5277 | 3229685 |
| 2545-2558 | Cr-pCr-pGr-pGr-pCr-pUr-pUr-pCr-pAr-pUr-pCr-pAr-pAr-pGr | Cyclic phosphorylation | 14 | 1.71 | 96.9 | 1 | 15.77 | 743.425 | -6 | 4465.5898 | 4467.49 | 4465.5822 | 314022.19 |
| 2545-2558 | Cr-pCr-pGr-pGr-pCr-pUr-pUr-pCr-pAr-pUr-pCr-pAr-pAr-pGr | Cyclic phosphorylation | 14 | 1.71 | 96.9 | 1.2 | 15.77 | 637.077 | -7 | 4465.5898 | 4467.48 | 4465.5822 | 314885.34 |
| 2483-2495 | Cr-pAr-pAr-pGr-pCr-pCr-pCr-pAr-pGr-pCr-pAr-pAr-pGr | Cyclic phosphorylation | 13 | 1.97 | 100 | 1 | 15.8 | 840.517 | -5 | 4205.6196 | 4207.54 | 4205.6113 | 368985.97 |
| 2483-2495 | Cr-pAr-pAr-pGr-pCr-pCr-pCr-pAr-pGr-pCr-pAr-pAr-pGr | Cyclic phosphorylation | 13 | 1.51 | 100 | 1 | 15.8 | 1401.199 | -3 | 4205.6177 | 4207.33 | 4205.6113 | 583004.62 |
| 2483-2495 | Cr-pAr-pAr-pGr-pCr-pCr-pCr-pAr-pGr-pCr-pAr-pAr-pGr | Cyclic phosphorylation | 13 | 1.62 | 100 | 1 | 15.8 | 700.096 | -6 | 4205.6182 | 4207.34 | 4205.6113 | 723633.06 |
| 3375-3386 | Ar-pAr-pCr-pUr-pUr-pCr-pUr-pAr-pCr-pGr-pAr-pGr | Cyclic phosphorylation | 12 | 1.7 | 99.9 | 1 | 15.82 | 639.078 | -6 | 3839.5112 | 3841.11 | 3839.5047 | 367251.5 |
| 3375-3386 | Ar-pAr-pCr-pUr-pUr-pCr-pUr-pAr-pCr-pGr-pAr-pGr | Cyclic phosphorylation | 12 | 1.57 | 99.9 | 1 | 15.82 | 767.095 | -5 | 3839.5107 | 3841.08 | 3839.5047 | 535062.06 |
| 3393-3405 | Ar-pUr-pCr-pAr-pUr-pCr-pAr-pCr-pCr-pAr-pCr-pCr-pGr | Cyclic phosphorylation | 13 | 1.39 | 100 | 1 | 16 | 1025.134 | -4 | 4103.5615 | 4105.21 | 4103.5558 | 1982295.25 |
| 3393-3405 | Ar-pUr-pCr-pAr-pUr-pCr-pAr-pCr-pCr-pAr-pCr-pCr-pGr | Cyclic phosphorylation | 13 | 0.91 | 100 | 1 | 16 | 1367.179 | -3 | 4103.5596 | 4105.23 | 4103.5558 | 6058994.5 |
| 3393-3405 | Ar-pUr-pCr-pAr-pUr-pCr-pAr-pCr-pCr-pAr-pCr-pCr-pGr | Cyclic phosphorylation | 13 | 0.55 | 100 | 1 | 16 | 819.905 | -5 | 4103.5581 | 4105.24 | 4103.5558 | 4740181 |
| 3393-3405 | Ar-pUr-pCr-pAr-pUr-pCr-pAr-pCr-pCr-pAr-pCr-pCr-pGr | Cyclic phosphorylation | 13 | 0.43 | 100 | 1 | 16 | 683.085 | -6 | 4103.5576 | 4105.23 | 4103.5558 | 9237335 |
| 3393-3405 | Ar-pUr-pCr-pAr-pUr-pCr-pAr-pCr-pCr-pAr-pCr-pCr-pGr | Cyclic phosphorylation | 13 | 1.86 | 100 | 1 | 16.01 | 2051.275 | -2 | 4103.5635 | 4105.28 | 4103.5558 | 1200967 |
| 3393-3405 | Ar-pUr-pCr-pAr-pUr-pCr-pAr-pCr-pCr-pAr-pCr-pCr-pGr | Cyclic phosphorylation | 13 | 1.15 | 100 | 1 | 16.01 | 585.359 | -7 | 4103.5605 | 4105.26 | 4103.5558 | 3606679.25 |
| 3969-3984 | Cr-pUr-pCr-pCr-pCr-pAr-pCr-pCr-pUr-pCr-pCr-pAr-pCr-pCr-pUr- | Cyclic phosphorylation | 16 | 1.35 | 100 | 1 | 16.1 | 620.699 | -8 | 4971.6479 | 4973.73 | 4971.6412 | 4967197.5 |
| 3969-3984 | Cr-pUr-pCr-pCr-pCr-pAr-pCr-pCr-pUr-pCr-pCr-pAr-pCr-pCr-pUr- | Cyclic phosphorylation | 16 | 1.84 | 100 | 1 | 16.11 | 993.523 | -5 | 4971.6504 | 4973.74 | 4971.6412 | 993328.06 |
| 3969-3984 | Cr-pUr-pCr-pCr-pCr-pAr-pCr-pCr-pUr-pCr-pCr-pAr-pCr-pCr-pUr- | Cyclic phosphorylation | 16 | 1.64 | 100 | 1 | 16.11 | 1656.877 | -3 | 4971.6494 | 4973.76 | 4971.6412 | 3784279 |
| 3969-3984 | Cr-pUr-pCr-pCr-pCr-pAr-pCr-pCr-pUr-pCr-pCr-pAr-pCr-pCr-pUr- | Cyclic phosphorylation | 16 | 0.96 | 100 | 1 | 16.11 | 709.514 | -7 | 4971.646 | 4973.74 | 4971.6412 | 4673351 |
| 3969-3984 | Cr-pUr-pCr-pCr-pCr-pAr-pCr-pCr-pUr-pCr-pCr-pAr-pCr-pCr-pUr- | Cyclic phosphorylation | 16 | 1.35 | 100 | 1 | 16.12 | 827.768 | -6 | 4971.6479 | 4973.78 | 4971.6412 | 2115141.5 |
| 1728-1742 | Ur-pUr-pCr-pCr-pUr-pGr-pCr-pCr-pAr-pUr-pUr-pCr-pCr-pAr-pG | Cyclic phosphorylation | 15 | 1.37 | 100 | 1 | 16.15 | 1568.853 | -3 | 4708.5806 | 4710.59 | 4708.5741 | 1664574.25 |
| 1728-1742 | Ur-pUr-pCr-pCr-pUr-pGr-pCr-pCr-pAr-pUr-pUr-pCr-pCr-pAr-pG | Cyclic phosphorylation | 15 | 1.26 | 100 | 1 | 16.15 | 783.923 | -6 | 4708.5801 | 4710.53 | 4708.5741 | 1168026 |
| 1728-1742 | Ur-pUr-pCr-pCr-pUr-pGr-pCr-pCr-pAr-pUr-pUr-pCr-pCr-pAr-pG | Cyclic phosphorylation | 15 | 1.57 | 100 | 1 | 16.17 | 671.933 | -7 | 4708.5815 | 4710.62 | 4708.5741 | 2443262.75 |
| 4085-4096 | Ar-pUr-pUr-pAr-pAr-pCr-pCr-pUr-pUr-pUr-pAr-pGr | Phosphorylation | 12 | 1.12 | 100 | 1 | 16.19 | 1272.488 | -3 | 3819.4814 | 3821.03 | 3819.4772 | 851240.19 |
| 4085-4096 | Ar-pUr-pUr-pAr-pAr-pCr-pCr-pUr-pUr-pUr-pAr-pGr | Phosphorylation | 12 | 1.51 | 100 | 1 | 16.19 | 635.74 | -6 | 3819.4829 | 3821.04 | 3819.4772 | 298721.09 |
| 4085-4096 | Ar-pUr-pUr-pAr-pAr-pCr-pCr-pUr-pUr-pUr-pAr-pGr | Phosphorylation | 12 | 0.99 | 100 | 1 | 16.2 | 763.089 | -5 | 3819.481 | 3821.1 | 3819.4772 | 1857413.62 |
| 1158-1171 | Ur-pAr-pCr-pAr-pAr-pCr-pUr-pCr-pCr-pGr-pCr-pCr-pAr-pGr | Cyclic phosphorylation | 14 | 2.14 | 97.1 | 1 | 16.25 | 889.115 | -5 | 4448.6128 | 4450.62 | 4448.6033 | 806014.5 |
| 1158-1171 | Ur-pAr-pCr-pAr-pAr-pCr-pUr-pCr-pCr-pGr-pCr-pCr-pAr-pGr | Cyclic phosphorylation | 14 | 2.03 | 97.1 | 1 | 16.25 | 634.794 | -7 | 4448.6123 | 4450.73 | 4448.6033 | 1325920.62 |
| 1158-1171 | Ur-pAr-pCr-pAr-pAr-pCr-pUr-pCr-pCr-pGr-pCr-pCr-pAr-pGr | Cyclic phosphorylation | 14 | 1.92 | 97.1 | 1 | 16.27 | 1482.529 | -3 | 4448.6118 | 4450.74 | 4448.6033 | 1545555.25 |
| 1158-1171 | Ur-pAr-pCr-pAr-pAr-pCr-pUr-pCr-pCr-pGr-pCr-pCr-pAr-pGr | Cyclic phosphorylation | 14 | 1.81 | 97.1 | 1 | 16.27 | 740.595 | -6 | 4448.6113 | 4450.69 | 4448.6033 | 1615793.25 |
| 1200-1214 | Ur-pCr-pCr-pCr-pCr-pUr-pAr-pCr-pCr-pAr-pAr-pGr-pCr-pUr-pGr | Cyclic phosphorylation | 15 | 1.41 | 99.9 | 1 | 16.38 | 675.082 | -7 | 4730.624 | 4732.68 | 4730.6173 | 806859.12 |
| 3453-3464 | Ar-pAr-pCr-pAr-pAr-pUr-pAr-pCr-pCr-pGr-pUr-pGr | Cyclic phosphorylation | 12 | 1.09 | 99.7 | 1 | 16.41 | 642.916 | -6 | 3862.5361 | 3864.11 | 3862.5319 | 2481622.25 |
| 3453-3464 | Ar-pAr-pCr-pAr-pAr-pUr-pAr-pCr-pCr-pGr-pUr-pGr | Cyclic phosphorylation | 12 | 1.21 | 99.7 | 1 | 16.41 | 1286.839 | -3 | 3862.5366 | 3864.18 | 3862.5319 | 1484727.5 |
| 3453-3464 | Ar-pAr-pCr-pAr-pAr-pUr-pAr-pCr-pCr-pGr-pUr-pGr | Cyclic phosphorylation | 12 | 1.09 | 99.7 | 1 | 16.41 | 771.701 | -5 | 3862.5361 | 3864.19 | 3862.5319 | 1348599.38 |
| 3273-3285 | Ar-pAr-pUr-pUr-pUr-pCr-pAr-pCr-pCr-pAr-pCr-pCr-pGr | Cyclic phosphorylation | 13 | 1.83 | 100 | 1 | 16.43 | 2051.767 | -2 | 4104.5474 | 4106.24 | 4104.5399 | 1207298.12 |
| 3273-3285 | Ar-pAr-pUr-pUr-pUr-pCr-pAr-pCr-pCr-pAr-pCr-pCr-pGr | Cyclic phosphorylation | 13 | 0.76 | 100 | 1 | 16.43 | 1367.507 | -3 | 4104.543 | 4106.2 | 4104.5399 | 6016561 |
| 3273-3285 | Ar-pAr-pUr-pUr-pUr-pCr-pAr-pCr-pCr-pAr-pCr-pCr-pGr | Cyclic phosphorylation | 13 | 0.05 | 100 | 1 | 16.43 | 683.249 | -6 | 4104.54 | 4106.22 | 4104.5399 | 9368131 |
| 3273-3285 | Ar-pAr-pUr-pUr-pUr-pCr-pAr-pCr-pCr-pAr-pCr-pCr-pGr | Cyclic phosphorylation | 13 | 0.64 | 100 | 1 | 16.43 | 820.101 | -5 | 4104.5425 | 4106.21 | 4104.5399 | 5138323.5 |
| 3273-3285 | Ar-pAr-pUr-pUr-pUr-pCr-pAr-pCr-pCr-pAr-pCr-pCr-pGr | Cyclic phosphorylation | 13 | 1.23 | 100 | 1 | 16.43 | 585.499 | -7 | 4104.5449 | 4106.21 | 4104.5399 | 3184488.5 |
| 3273-3285 | Ar-pAr-pUr-pUr-pUr-pCr-pAr-pCr-pCr-pAr-pCr-pCr-pGr | Cyclic phosphorylation | 13 | 1 | 100 | 1 | 16.43 | 1025.379 | -4 | 4104.5439 | 4106.23 | 4104.5399 | 2014330.12 |
| 2808-2819 | Ar-pAr-pCr-pCr-pAr-pGr-pAr-pAr-pGr-pCr-pUr-pGr | Cyclic phosphorylation | 12 | 1.47 | 99.9 | 1 | 16.43 | 779.705 | -5 | 3901.5598 | 3903.4 | 3901.5541 | 411642 |
| 2808-2819 | Ar-pAr-pCr-pCr-pAr-pGr-pAr-pAr-pGr-pCr-pUr-pGr | Cyclic phosphorylation | 12 | 1.41 | 99.9 | 1 | 16.44 | 649.42 | -6 | 3901.5596 | 3903.15 | 3901.5541 | 782202.62 |
| 3469-3483 | Ar-pCr-pCr-pCr-pUr-pCr-pUr-pGr-pCr-pAr-pGr-pCr-pCr-pCr-pGr | Cyclic phosphorylation | 15 | 1.79 | 93.5 | 1 | 16.51 | 677.084 | -7 | 4745.6367 | 4747.66 | 4745.6282 | 751382.75 |
| 1275-1290 | Cr-pGr-pGr-pCr-pAr-pGr-pAr-pUr-pUr-pGr-pCr-pCr-pCr-pCr-pU | Cyclic phosphorylation | 16 | 1.37 | 100 | 1.1 | 16.56 | 854.605 | -6 | 5131.6729 | 5133.9 | 5131.6658 | 540819.75 |
| 1275-1290 | Cr-pGr-pGr-pCr-pAr-pGr-pAr-pUr-pUr-pGr-pCr-pCr-pCr-pCr-pU | Cyclic phosphorylation | 16 | 0.89 | 100 | 1 | 16.56 | 732.375 | -7 | 5131.6704 | 5133.9 | 5131.6658 | 744900.69 |
| 3742-3755 | Ur-pCr-pAr-pCr-pAr-pAr-pUr-pCr-pAr-pUr-pGr-pCr-pUr-pGr | Cyclic phosphorylation | 14 | 2.19 | 99.6 | 1 | 16.65 | 740.923 | -6 | 4450.5811 | 4452.48 | 4450.5713 | 283682.34 |
| 3742-3755 | Ur-pCr-pAr-pCr-pAr-pAr-pUr-pCr-pAr-pUr-pGr-pCr-pUr-pGr | Cyclic phosphorylation | 14 | 1.97 | 99.6 | 1 | 16.65 | 1482.853 | -3 | 4450.5801 | 4452.47 | 4450.5713 | 253064.09 |
| 3742-3755 | Ur-pCr-pAr-pCr-pAr-pAr-pUr-pCr-pAr-pUr-pGr-pCr-pUr-pGr | Cyclic phosphorylation | 14 | 2.3 | 99.6 | 1.1 | 16.67 | 635.076 | -7 | 4450.5815 | 4452.44 | 4450.5713 | 206560.2 |
| 3619-3632 | Ur-pGr-pGr-pCr-pCr-pAr-pAr-pGr-pAr-pAr-pUr-pCr-pUr-pGr | Cyclic phosphorylation | 14 | 1.51 | 99.9 | 1 | 16.71 | 754.094 | -6 | 4529.6064 | 4531.33 | 4529.5996 | 456082.53 |
| 3619-3632 | Ur-pGr-pGr-pCr-pCr-pAr-pAr-pGr-pAr-pAr-pUr-pCr-pUr-pGr | Cyclic phosphorylation | 14 | 2.81 | 99.9 | 1.1 | 16.72 | 1509.195 | -3 | 4529.6123 | 4531.53 | 4529.5996 | 439172.03 |
| 172-184 | Ar-pCr-pAr-pAr-pGr-pGr-pUr-pGr-pUr-pUr-pCr-pAr-pGr | Cyclic phosphorylation | 13 | 0.76 | 100 | 1 | 16.81 | 1407.849 | -3 | 4224.5615 | 4226.44 | 4224.5583 | 313601.59 |
| 172-184 | Ar-pCr-pAr-pAr-pGr-pGr-pUr-pGr-pUr-pUr-pCr-pAr-pGr | Cyclic phosphorylation | 13 | 1.34 | 100 | 1 | 16.81 | 703.254 | -6 | 4224.564 | 4226.27 | 4224.5583 | 529763.62 |
| 172-184 | Ar-pCr-pAr-pAr-pGr-pGr-pUr-pGr-pUr-pUr-pCr-pAr-pGr | Cyclic phosphorylation | 13 | 1.34 | 100 | 1 | 16.83 | 844.106 | -5 | 4224.564 | 4226.16 | 4224.5583 | 147636.59 |
| 2239-2252 | Ur-pGr-pUr-pCr-pCr-pAr-pUr-pGr-pAr-pCr-pCr-pAr-pAr-pGr | Cyclic phosphorylation | 14 | 1.16 | 100 | 1 | 16.86 | 747.426 | -6 | 4489.5986 | 4491.54 | 4489.5934 | 1417136.25 |
| 2239-2252 | Ur-pGr-pUr-pCr-pCr-pAr-pUr-pGr-pAr-pCr-pCr-pAr-pAr-pGr | Cyclic phosphorylation | 14 | 1.27 | 100 | 1.1 | 16.86 | 640.65 | -7 | 4489.5991 | 4491.57 | 4489.5934 | 1084209.5 |
| 2239-2252 | Ur-pGr-pUr-pCr-pCr-pAr-pUr-pGr-pAr-pCr-pCr-pAr-pAr-pGr | Cyclic phosphorylation | 14 | 1.38 | 100 | 1 | 16.86 | 897.113 | -5 | 4489.5996 | 4491.54 | 4489.5934 | 570616.44 |
| 2239-2252 | Ur-pGr-pUr-pCr-pCr-pAr-pUr-pGr-pAr-pCr-pCr-pAr-pAr-pGr | Cyclic phosphorylation | 14 | 1.05 | 100 | 1 | 16.86 | 1495.859 | -3 | 4489.5981 | 4491.52 | 4489.5934 | 1268104.38 |
| 2239-2252 | Ur-pGr-pUr-pCr-pCr-pAr-pUr-pGr-pAr-pCr-pCr-pAr-pAr-pGr | Cyclic phosphorylation | 14 | 1.48 | 100 | 1 | 16.86 | 1121.894 | -4 | 4489.6001 | 4491.52 | 4489.5934 | 308697.72 |
| 1986-1999 | Ar-pCr-pCr-pAr-pGr-pAr-pGr-pCr-pCr-pGr-pGr-pCr-pUr-pGr | Cyclic phosphorylation | 14 | 1.61 | 100 | 1 | 16.93 | 1514.204 | -3 | 4543.6338 | 4545.74 | 4543.6265 | 488372.91 |
| 1986-1999 | Ar-pCr-pCr-pAr-pGr-pAr-pGr-pCr-pCr-pGr-pGr-pCr-pUr-pGr | Cyclic phosphorylation | 14 | 1.18 | 100 | 1.2 | 16.93 | 907.919 | -5 | 4543.6318 | 4545.59 | 4543.6265 | 200353.78 |
| 1986-1999 | Ar-pCr-pCr-pAr-pGr-pAr-pGr-pCr-pCr-pGr-pGr-pCr-pUr-pGr | Cyclic phosphorylation | 14 | 1.29 | 100 | 1.1 | 16.93 | 648.37 | -7 | 4543.6323 | 4545.63 | 4543.6265 | 491984.38 |
| 1986-1999 | Ar-pCr-pCr-pAr-pGr-pAr-pGr-pCr-pCr-pGr-pGr-pCr-pUr-pGr | Cyclic phosphorylation | 14 | 1.83 | 100 | 1 | 16.93 | 756.431 | -6 | 4543.6348 | 4545.72 | 4543.6265 | 471483 |
| 3969-3984 | Cr-pUr-pCr-pCr-pCr-pAr-pCr-pCr-pUr-pCr-pCr-pAr-pCr-pCr-pUr- | Phosphorylation | 16 | 1.67 | 100 | 1.1 | 16.98 | 830.937 | -6 | 4989.6602 | 4991.83 | 4989.6518 | 265956.94 |
| 3406-3419 | Ar-pCr-pAr-pAr-pCr-pAr-pCr-pCr-pUr-pUr-pCr-pGr-pUr-pGr | Cyclic phosphorylation | 14 | 2.66 | 100 | 1 | 17.04 | 740.76 | -6 | 4449.5991 | 4451.24 | 4449.5873 | 507710.16 |
| 2247-2259 | Ar-pCr-pCr-pAr-pAr-pGr-pAr-pCr-pCr-pAr-pGr-pCr-pGr | Cyclic phosphorylation | 13 | 1.62 | 97 | 1.1 | 17.04 | 1401.2 | -3 | 4205.6182 | 4207.38 | 4205.6113 | 228632.08 |
| 2247-2259 | Ar-pCr-pCr-pAr-pAr-pGr-pAr-pCr-pCr-pAr-pGr-pCr-pGr | Cyclic phosphorylation | 13 | 1.39 | 97 | 1 | 17.07 | 700.096 | -6 | 4205.6172 | 4207.32 | 4205.6113 | 274134.16 |
| 3761-3775 | Cr-pAr-pUr-pGr-pAr-pCr-pCr-pAr-pGr-pCr-pUr-pGr-pCr-pUr-pG | Cyclic phosphorylation | 15 | 1.88 | 100 | 1 | 17.14 | 800.933 | -6 | 4810.6387 | 4812.7 | 4810.6296 | 1134861.12 |
| 3761-3775 | Cr-pAr-pUr-pGr-pAr-pCr-pCr-pAr-pGr-pCr-pUr-pGr-pCr-pUr-pG | Cyclic phosphorylation | 15 | 1.88 | 100 | 1.1 | 17.14 | 961.521 | -5 | 4810.6387 | 4812.71 | 4810.6296 | 451699.97 |
| 3761-3775 | Cr-pAr-pUr-pGr-pAr-pCr-pCr-pAr-pGr-pCr-pUr-pGr-pCr-pUr-pG | Cyclic phosphorylation | 15 | 1.68 | 100 | 1 | 17.14 | 1603.207 | -3 | 4810.6377 | 4812.71 | 4810.6296 | 1233968 |
| 3761-3775 | Cr-pAr-pUr-pGr-pAr-pCr-pCr-pAr-pGr-pCr-pUr-pGr-pCr-pUr-pG | Cyclic phosphorylation | 15 | 1.88 | 100 | 1 | 17.16 | 686.513 | -7 | 4810.6387 | 4812.72 | 4810.6296 | 1715710.88 |
| 2253-2266 | Ar-pCr-pCr-pAr-pGr-pCr-pGr-pUr-pGr-pGr-pAr-pCr-pUr-pGr | Cyclic phosphorylation | 14 | 1.37 | 100 | 1 | 17.25 | 648.367 | -7 | 4544.6167 | 4546.62 | 4544.6105 | 772524.62 |
| 2253-2266 | Ar-pCr-pCr-pAr-pGr-pCr-pGr-pUr-pGr-pGr-pAr-pCr-pUr-pGr | Cyclic phosphorylation | 14 | 1.15 | 100 | 1 | 17.25 | 1514.199 | -3 | 4544.6157 | 4546.61 | 4544.6105 | 667720.25 |
| 2253-2266 | Ar-pCr-pCr-pAr-pGr-pCr-pGr-pUr-pGr-pGr-pAr-pCr-pUr-pGr | Cyclic phosphorylation | 14 | 1.37 | 100 | 1 | 17.25 | 756.763 | -6 | 4544.6167 | 4546.45 | 4544.6105 | 658999.56 |
| 2085-2098 | Ar-pCr-pAr-pAr-pAr-pCr-pAr-pGr-pCr-pCr-pCr-pUr-pCr-pGr | Cyclic phosphorylation | 14 | 0.85 | 100 | 1 | 17.3 | 893.52 | -5 | 4471.6343 | 4473.58 | 4471.6305 | 682603.62 |
| 2085-2098 | Ar-pCr-pAr-pAr-pAr-pCr-pAr-pGr-pCr-pCr-pCr-pUr-pCr-pGr | Cyclic phosphorylation | 14 | 1.17 | 100 | 1 | 17.3 | 744.432 | -6 | 4471.6357 | 4473.39 | 4471.6305 | 1571071.62 |
| 2085-2098 | Ar-pCr-pAr-pAr-pAr-pCr-pAr-pGr-pCr-pCr-pCr-pUr-pCr-pGr | Cyclic phosphorylation | 14 | 1.07 | 100 | 1 | 17.3 | 638.084 | -7 | 4471.6353 | 4473.61 | 4471.6305 | 1459547.25 |
| 2085-2098 | Ar-pCr-pAr-pAr-pAr-pCr-pAr-pGr-pCr-pCr-pCr-pUr-pCr-pGr | Cyclic phosphorylation | 14 | 0.85 | 100 | 1 | 17.3 | 1489.872 | -3 | 4471.6343 | 4473.6 | 4471.6305 | 1590991.88 |
| 3201-3215 | Ar-pUr-pGr-pAr-pGr-pCr-pUr-pUr-pCr-pCr-pCr-pUr-pCr-pAr-pG | Cyclic phosphorylation | 15 | 1.72 | 100 | 1 | 17.35 | 680.795 | -7 | 4771.6157 | 4773.64 | 4771.6075 | 1585136.62 |
| 3201-3215 | Ar-pUr-pGr-pAr-pGr-pCr-pUr-pUr-pCr-pCr-pCr-pUr-pCr-pAr-pG | Cyclic phosphorylation | 15 | 1.31 | 100 | 1 | 17.35 | 1589.865 | -3 | 4771.6138 | 4773.67 | 4771.6075 | 1170733 |
| 3201-3215 | Ar-pUr-pGr-pAr-pGr-pCr-pUr-pUr-pCr-pCr-pCr-pUr-pCr-pAr-pG | Cyclic phosphorylation | 15 | 1.42 | 100 | 1 | 17.35 | 794.596 | -6 | 4771.6143 | 4773.6 | 4771.6075 | 800176.06 |
| 345-358 | Ur-pCr-pCr-pAr-pAr-pCr-pAr-pUr-pCr-pAr-pUr-pCr-pAr-pGr | Cyclic phosphorylation | 14 | 1.3 | 100 | 1 | 17.4 | 1477.193 | -3 | 4433.5981 | 4435.56 | 4433.5924 | 853002.19 |
| 345-358 | Ur-pCr-pCr-pAr-pAr-pCr-pAr-pUr-pCr-pAr-pUr-pCr-pAr-pGr | Cyclic phosphorylation | 14 | 1.52 | 100 | 1 | 17.4 | 632.65 | -7 | 4433.5991 | 4435.35 | 4433.5924 | 774496.19 |
| 1953-1968 | Cr-pGr-pGr-pGr-pUr-pGr-pUr-pAr-pCr-pUr-pCr-pCr-pAr-pCr-pC | Cyclic phosphorylation | 16 | 1.84 | 100 | 1 | 17.4 | 854.439 | -6 | 5131.6753 | 5133.77 | 5131.6658 | 559616.19 |
| 345-358 | Ur-pCr-pCr-pAr-pAr-pCr-pAr-pUr-pCr-pAr-pUr-pCr-pAr-pGr | Cyclic phosphorylation | 14 | 1.41 | 100 | 1 | 17.42 | 738.093 | -6 | 4433.5986 | 4435.54 | 4433.5924 | 942591.06 |
| 1953-1968 | Cr-pGr-pGr-pGr-pUr-pGr-pUr-pAr-pCr-pUr-pCr-pCr-pAr-pCr-pC | Cyclic phosphorylation | 16 | 1.75 | 100 | 1 | 17.42 | 732.376 | -7 | 5131.6748 | 5133.83 | 5131.6658 | 617286.56 |
| 1608-1623 | Cr-pAr-pUr-pGr-pCr-pCr-pCr-pCr-pUr-pGr-pCr-pCr-pAr-pCr-pAr | Cyclic phosphorylation | 16 | 1.91 | 92.3 | 1 | 17.45 | 724.235 | -7 | 5074.6904 | 5076.83 | 5074.6808 | 776881.44 |
| 1868-1881 | Cr-pAr-pAr-pUr-pCr-pAr-pGr-pGr-pUr-pGr-pGr-pCr-pAr-pGr | Cyclic phosphorylation | 14 | 1.57 | 99.9 | 1 | 17.45 | 760.598 | -6 | 4568.6289 | 4570.42 | 4568.6217 | 605769.81 |
| 1868-1881 | Cr-pAr-pAr-pUr-pCr-pAr-pGr-pGr-pUr-pGr-pGr-pCr-pAr-pGr | Cyclic phosphorylation | 14 | 1.79 | 99.9 | 1 | 17.45 | 651.941 | -7 | 4568.6299 | 4570.67 | 4568.6217 | 451343.44 |
| 1608-1623 | Cr-pAr-pUr-pGr-pCr-pCr-pCr-pCr-pUr-pGr-pCr-pCr-pAr-pCr-pAr | Cyclic phosphorylation | 16 | 2 | 92.3 | 1.8 | 17.46 | 1691.224 | -3 | 5074.6909 | 5076.89 | 5074.6808 | 617040.62 |
| 1608-1623 | Cr-pAr-pUr-pGr-pCr-pCr-pCr-pCr-pUr-pGr-pCr-pCr-pAr-pCr-pAr | Cyclic phosphorylation | 16 | 2.1 | 92.3 | 1 | 17.47 | 845.108 | -6 | 5074.6914 | 5076.9 | 5074.6808 | 636829.25 |
| 1608-1623 | Cr-pAr-pUr-pGr-pCr-pCr-pCr-pCr-pUr-pGr-pCr-pCr-pAr-pCr-pAr | Cyclic phosphorylation | 16 | 2.77 | 92.3 | 1.2 | 17.47 | 633.58 | -8 | 5074.6948 | 5076.75 | 5074.6808 | 263678.94 |
| 43-56 | Ar-pAr-pCr-pCr-pCr-pGr-pCr-pCr-pAr-pCr-pCr-pAr-pUr-pGr | Cyclic phosphorylation | 14 | 2.06 | 100 | 1 | 17.67 | 1111.399 | -4 | 4447.6284 | 4449.75 | 4447.6193 | 314660.22 |
| 43-56 | Ar-pAr-pCr-pCr-pCr-pGr-pCr-pCr-pAr-pCr-pCr-pAr-pUr-pGr | Cyclic phosphorylation | 14 | 1.4 | 100 | 1 | 17.67 | 1482.199 | -3 | 4447.6255 | 4449.9 | 4447.6193 | 1213301.62 |
| 43-56 | Ar-pAr-pCr-pCr-pCr-pGr-pCr-pCr-pAr-pCr-pCr-pAr-pUr-pGr | Cyclic phosphorylation | 14 | 1.73 | 100 | 1 | 17.68 | 740.596 | -6 | 4447.627 | 4449.91 | 4447.6193 | 1237717.75 |
| 43-56 | Ar-pAr-pCr-pCr-pCr-pGr-pCr-pCr-pAr-pCr-pCr-pAr-pUr-pGr | Cyclic phosphorylation | 14 | 1.84 | 100 | 1 | 17.7 | 888.918 | -5 | 4447.6274 | 4449.83 | 4447.6193 | 502603.72 |
| 43-56 | Ar-pAr-pCr-pCr-pCr-pGr-pCr-pCr-pAr-pCr-pCr-pAr-pUr-pGr | Cyclic phosphorylation | 14 | 1.73 | 100 | 1 | 17.7 | 634.652 | -7 | 4447.627 | 4449.89 | 4447.6193 | 1334762.5 |
| 216-232 | Ur-pUr-pCr-pCr-pUr-pGr-pCr-pCr-pUr-pUr-pUr-pCr-pUr-pUr-pC | Cyclic phosphorylation | 17 | 1.78 | 100 | 1 | 17.7 | 661.444 | -8 | 5297.6069 | 5299.88 | 5297.5975 | 425139.06 |
| 646-659 | Ar-pCr-pGr-pGr-pCr-pUr-pAr-pCr-pUr-pUr-pCr-pAr-pAr-pGr | Cyclic phosphorylation | 14 | 1.92 | 99 | 1.3 | 17.7 | 640.651 | -7 | 4489.6021 | 4491.58 | 4489.5934 | 389793.62 |
| 1823-1838 | Cr-pAr-pGr-pCr-pUr-pUr-pCr-pGr-pGr-pCr-pGr-pGr-pAr-pGr-pU | Cyclic phosphorylation | 16 | 1.89 | 99.9 | 1.2 | 17.7 | 650.704 | -8 | 5211.688 | 5213.84 | 5211.6781 | 354775.84 |
| 646-659 | Ar-pCr-pGr-pGr-pCr-pUr-pAr-pCr-pUr-pUr-pCr-pAr-pAr-pGr | Cyclic phosphorylation | 14 | 2.46 | 99 | 1 | 17.72 | 747.593 | -6 | 4489.6045 | 4491.58 | 4489.5934 | 304912.78 |
| 646-659 | Ar-pCr-pGr-pGr-pCr-pUr-pAr-pCr-pUr-pUr-pCr-pAr-pAr-pGr | Cyclic phosphorylation | 14 | 1.59 | 99 | 1 | 17.73 | 1496.194 | -3 | 4489.6006 | 4491.6 | 4489.5934 | 301494.59 |
| 1823-1838 | Cr-pAr-pGr-pCr-pUr-pUr-pCr-pGr-pGr-pCr-pGr-pGr-pAr-pGr-pU | Cyclic phosphorylation | 16 | 1.89 | 99.9 | 1 | 17.73 | 743.806 | -7 | 5211.688 | 5213.93 | 5211.6781 | 536520.38 |
| 1449-1464 | Cr-pGr-pGr-pGr-pAr-pCr-pAr-pUr-pCr-pUr-pCr-pCr-pAr-pCr-pCr | Cyclic phosphorylation | 16 | 1.55 | 100 | 1 | 17.8 | 729.95 | -7 | 5114.6948 | 5116.89 | 5114.6869 | 495823.19 |
| 1449-1464 | Cr-pGr-pGr-pGr-pAr-pCr-pAr-pUr-pCr-pUr-pCr-pCr-pAr-pCr-pCr | Cyclic phosphorylation | 16 | 1.64 | 100 | 1 | 17.82 | 851.776 | -6 | 5114.6953 | 5116.86 | 5114.6869 | 461176.22 |
| 1449-1464 | Cr-pGr-pGr-pGr-pAr-pCr-pAr-pUr-pCr-pUr-pCr-pCr-pAr-pCr-pCr | Cyclic phosphorylation | 16 | 1.45 | 100 | 1.1 | 17.82 | 1704.559 | -3 | 5114.6943 | 5116.85 | 5114.6869 | 435802.38 |
| 246-261 | Ur-pUr-pCr-pCr-pAr-pCr-pGr-pCr-pCr-pAr-pUr-pCr-pCr-pAr-pCr | Cyclic phosphorylation | 16 | 2.34 | 100 | 1.8 | 17.85 | 628.577 | -8 | 5035.6704 | 5037.89 | 5035.6586 | 517898.47 |
| 246-261 | Ur-pUr-pCr-pCr-pAr-pCr-pGr-pCr-pCr-pAr-pUr-pCr-pCr-pAr-pCr | Cyclic phosphorylation | 16 | 1.86 | 100 | 1 | 17.86 | 838.604 | -6 | 5035.668 | 5038.24 | 5035.6586 | 432414.97 |
| 246-261 | Ur-pUr-pCr-pCr-pAr-pCr-pGr-pCr-pCr-pAr-pUr-pCr-pCr-pAr-pCr | Cyclic phosphorylation | 16 | 1.95 | 100 | 1 | 17.86 | 718.659 | -7 | 5035.6685 | 5037.98 | 5035.6586 | 878464.88 |
| 246-261 | Ur-pUr-pCr-pCr-pAr-pCr-pGr-pCr-pCr-pAr-pUr-pCr-pCr-pAr-pCr | Cyclic phosphorylation | 16 | 1.86 | 100 | 1.1 | 17.87 | 1678.215 | -3 | 5035.668 | 5038.14 | 5035.6586 | 621346.19 |
| 3864-3877 | Cr-pAr-pCr-pUr-pAr-pCr-pAr-pCr-pAr-pUr-pGr-pAr-pUr-pGr | Cyclic phosphorylation | 14 | 1.55 | 100 | 1.1 | 17.91 | 894.115 | -5 | 4473.6055 | 4475.37 | 4473.5985 | 267324.59 |

| | | | | | | | | | | | |
|---|---|---|---|---|---|---|---|---|---|---|---|
| 3864-3877 | Cr-pAr-pCr-pUr-pAr-pCr-pAr-pCr-pAr-pUr-pGr-pAr-pUr-pGr | Cyclic phosphorylation | 14 | 1.55 | 100 | 1 | 17.92 | 744.928 | -6 | 4473.6055 | 4475.59 | 4473.5985 | 497948.34 |
| 3864-3877 | Cr-pAr-pCr-pUr-pAr-pCr-pAr-pCr-pAr-pUr-pGr-pAr-pUr-pGr | Cyclic phosphorylation | 14 | 1.33 | 100 | 1 | 17.92 | 1490.529 | -3 | 4473.6045 | 4475.53 | 4473.5985 | 380763.06 |
| 2392-2406 | Ar-pGr-pGr-pUr-pGr-pUr-pUr-pCr-pGr-pCr-pCr-pCr-pAr-pAr-pG | Cyclic phosphorylation | 15 | 2.21 | 100 | 1 | 18.1 | 1616.543 | -3 | 4850.6465 | 4852.72 | 4850.6358 | 886401.75 |
| 2392-2406 | Ar-pGr-pGr-pUr-pGr-pUr-pUr-pCr-pGr-pCr-pCr-pCr-pAr-pAr-pG | Cyclic phosphorylation | 15 | 2.41 | 100 | 1 | 18.1 | 692.085 | -7 | 4850.6475 | 4852.66 | 4850.6358 | 1070546.5 |
| 2392-2406 | Ar-pGr-pGr-pUr-pGr-pUr-pUr-pCr-pGr-pCr-pCr-pCr-pAr-pAr-pG | Cyclic phosphorylation | 15 | 2 | 100 | 1 | 18.1 | 807.601 | -6 | 4850.6455 | 4852.68 | 4850.6358 | 790533.06 |
| 2392-2406 | Ar-pGr-pGr-pUr-pGr-pUr-pUr-pCr-pGr-pCr-pCr-pCr-pAr-pAr-pG | Cyclic phosphorylation | 15 | 2.81 | 100 | 1 | 18.12 | 969.323 | -5 | 4850.6494 | 4852.67 | 4850.6358 | 312817.28 |
| 1281-1295 | Ar-pUr-pUr-pGr-pCr-pCr-pCr-pCr-pUr-pGr-pGr-pAr-pCr-pAr-pG | Cyclic phosphorylation | 15 | 2.08 | 100 | 1 | 18.17 | 1602.873 | -3 | 4810.6396 | 4813.06 | 4810.6296 | 442079.53 |
| 1281-1295 | Ar-pUr-pUr-pGr-pCr-pCr-pCr-pCr-pUr-pGr-pGr-pAr-pCr-pAr-pG | Cyclic phosphorylation | 15 | 1.98 | 100 | 1 | 18.17 | 801.1 | -6 | 4810.6392 | 4812.72 | 4810.6296 | 378044.5 |
| 1281-1295 | Ar-pUr-pUr-pGr-pCr-pCr-pCr-pCr-pUr-pGr-pGr-pAr-pCr-pAr-pG | Cyclic phosphorylation | 15 | 2.18 | 100 | 1 | 18.19 | 686.37 | -7 | 4810.6401 | 4812.65 | 4810.6296 | 726865.12 |
| 2578-2593 | Gr-pCr-pGr-pAr-pCr-pAr-pUr-pUr-pGr-pCr-pCr-pGr-pCr-pCr-pAr | Cyclic phosphorylation | 16 | 1.95 | 100 | 1 | 18.29 | 735.665 | -7 | 5154.7031 | 5156.9 | 5154.6931 | 1004617.06 |
| 2578-2593 | Gr-pCr-pGr-pAr-pCr-pAr-pUr-pUr-pGr-pCr-pCr-pGr-pCr-pCr-pAr | Cyclic phosphorylation | 16 | 2.14 | 100 | 1.1 | 18.31 | 1030.334 | -5 | 5154.7041 | 5157 | 5154.6931 | 279523.53 |
| 2578-2593 | Gr-pCr-pGr-pAr-pCr-pAr-pUr-pUr-pGr-pCr-pCr-pGr-pCr-pCr-pAr | Cyclic phosphorylation | 16 | 2.33 | 100 | 1.1 | 18.31 | 1717.896 | -3 | 5154.7051 | 5156.89 | 5154.6931 | 599718.06 |
| 2578-2593 | Gr-pCr-pGr-pAr-pCr-pAr-pUr-pUr-pGr-pCr-pCr-pGr-pCr-pCr-pAr | Cyclic phosphorylation | 16 | 1.95 | 100 | 1 | 18.31 | 858.444 | -6 | 5154.7031 | 5156.84 | 5154.6931 | 610086.62 |
| 2578-2593 | Gr-pCr-pGr-pAr-pCr-pAr-pUr-pUr-pGr-pCr-pCr-pGr-pCr-pCr-pAr | Cyclic phosphorylation | 16 | 2.14 | 100 | 1.2 | 18.32 | 643.581 | -8 | 5154.7041 | 5157.05 | 5154.6931 | 566130.62 |
| 1292-1304 | Ar-pCr-pAr-pGr-pAr-pCr-pAr-pGr-pGr-pCr-pAr-pAr-pGr | Cyclic phosphorylation | 13 | 1.99 | 100 | 1 | 18.4 | 710.766 | -6 | 4269.6372 | 4271.52 | 4269.6287 | 412979.38 |
| 1292-1304 | Ar-pCr-pAr-pGr-pAr-pCr-pAr-pGr-pGr-pCr-pAr-pAr-pGr | Cyclic phosphorylation | 13 | 1.53 | 100 | 1 | 18.4 | 1422.539 | -3 | 4269.6353 | 4271.53 | 4269.6287 | 384570.5 |
| 2424-2438 | Ar-pCr-pCr-pCr-pCr-pUr-pCr-pCr-pUr-pAr-pUr-pCr-pAr-pAr-pGr | Cyclic phosphorylation | 15 | 1.27 | 100 | 1 | 18.68 | 672.654 | -7 | 4714.6284 | 4716.63 | 4714.6224 | 1917789.5 |
| 2424-2438 | Ar-pCr-pCr-pCr-pCr-pUr-pCr-pCr-pUr-pAr-pUr-pCr-pAr-pAr-pGr | Cyclic phosphorylation | 15 | 2 | 100 | 1.2 | 18.68 | 588.572 | -8 | 4714.6318 | 4716.67 | 4714.6224 | 423561.28 |
| 2424-2438 | Ar-pCr-pCr-pCr-pCr-pUr-pCr-pCr-pUr-pAr-pUr-pCr-pAr-pAr-pGr | Cyclic phosphorylation | 15 | 1.48 | 100 | 1 | 18.68 | 942.319 | -5 | 4714.6294 | 4716.66 | 4714.6224 | 533665.12 |
| 2424-2438 | Ar-pCr-pCr-pCr-pCr-pUr-pCr-pCr-pUr-pAr-pUr-pCr-pAr-pAr-pGr | Cyclic phosphorylation | 15 | 1.27 | 100 | 1 | 18.69 | 1570.869 | -3 | 4714.6284 | 4716.66 | 4714.6224 | 1637722.38 |
| 2424-2438 | Ar-pCr-pCr-pCr-pCr-pUr-pCr-pCr-pUr-pAr-pUr-pCr-pAr-pAr-pGr | Cyclic phosphorylation | 15 | 1.27 | 100 | 1 | 18.7 | 784.931 | -6 | 4714.6284 | 4716.62 | 4714.6224 | 1315994.88 |
| 2424-2438 | Ar-pCr-pCr-pCr-pCr-pUr-pCr-pCr-pUr-pAr-pUr-pCr-pAr-pAr-pGr | Cyclic phosphorylation | 15 | 1.37 | 100 | 1 | 18.7 | 1177.901 | -4 | 4714.6289 | 4716.63 | 4714.6224 | 379087.84 |
| 1017-1032 | Cr-pCr-pCr-pAr-pCr-pCr-pGr-pAr-pAr-pUr-pCr-pCr-pAr-pUr-pCr- | Cyclic phosphorylation | 16 | 1.39 | 100 | 1 | 18.78 | 842.442 | -6 | 5058.6929 | 5060.82 | 5058.6858 | 371559.44 |
| 1017-1032 | Cr-pCr-pCr-pAr-pCr-pCr-pGr-pAr-pAr-pUr-pCr-pCr-pAr-pUr-pCr- | Cyclic phosphorylation | 16 | 1.1 | 100 | 1 | 18.79 | 722.092 | -7 | 5058.6914 | 5060.94 | 5058.6858 | 613326.12 |
| 1017-1032 | Cr-pCr-pCr-pAr-pCr-pCr-pGr-pAr-pAr-pUr-pCr-pCr-pAr-pUr-pCr- | Cyclic phosphorylation | 16 | 1 | 100 | 1.1 | 18.8 | 1685.891 | -3 | 5058.6909 | 5060.85 | 5058.6858 | 457924.03 |
| 316-330 | Ar-pCr-pGr-pGr-pGr-pGr-pUr-pGr-pUr-pAr-pCr-pUr-pUr-pUr-pC | Cyclic phosphorylation | 15 | 0.98 | 100 | 1 | 18.85 | 810.761 | -6 | 4868.6035 | 4870.55 | 4868.5987 | 584526 |
| 316-330 | Ar-pCr-pGr-pGr-pGr-pGr-pUr-pGr-pUr-pAr-pCr-pUr-pUr-pUr-pC | Cyclic phosphorylation | 15 | 0.88 | 100 | 1 | 18.85 | 1622.528 | -3 | 4868.603 | 4871.09 | 4868.5987 | 693589.12 |
| 316-330 | Ar-pCr-pGr-pGr-pGr-pGr-pUr-pGr-pUr-pAr-pCr-pUr-pUr-pUr-pC | Cyclic phosphorylation | 15 | 0.88 | 100 | 1 | 18.85 | 694.794 | -7 | 4868.603 | 4870.7 | 4868.5987 | 1391369.5 |
| 3618-3632 | Gr-pUr-pGr-pGr-pCr-pCr-pAr-pAr-pGr-pAr-pAr-pUr-pCr-pUr-pG | Cyclic phosphorylation | 15 | 1.69 | 99.6 | 1.1 | 18.92 | 695.659 | -7 | 4874.6553 | 4876.67 | 4874.647 | 352583 |
| 3618-3632 | Gr-pUr-pGr-pGr-pCr-pCr-pAr-pAr-pGr-pAr-pAr-pUr-pCr-pUr-pG | Cyclic phosphorylation | 15 | 2.29 | 99.6 | 1.2 | 18.94 | 811.603 | -6 | 4874.6582 | 4876.55 | 4874.647 | 334960.84 |
| 916-932 | Cr-pCr-pGr-pUr-pGr-pGr-pAr-pUr-pUr-pGr-pUr-pGr-pCr-pUr-pC | Cyclic phosphorylation | 17 | 1.65 | 99.4 | 1 | 19.05 | 778.659 | -7 | 5455.6631 | 5458.06 | 5455.6541 | 561190.44 |
| 3216-3233 | Ur-pCr-pUr-pGr-pCr-pCr-pCr-pCr-pUr-pCr-pAr-pCr-pGr-pGr-pCr | Cyclic phosphorylation | 18 | 1.2 | 100 | 1 | 19.11 | 816.24 | -7 | 5718.728 | 5721.18 | 5718.7212 | 2036373.75 |
| 3216-3233 | Ur-pCr-pUr-pGr-pCr-pCr-pCr-pCr-pUr-pCr-pAr-pCr-pGr-pGr-pCr | Cyclic phosphorylation | 18 | 1.88 | 100 | 1 | 19.11 | 952.448 | -6 | 5718.7319 | 5721.23 | 5718.7212 | 997643.75 |
| 3216-3233 | Ur-pCr-pUr-pGr-pCr-pCr-pCr-pCr-pUr-pCr-pAr-pCr-pGr-pGr-pCr | Cyclic phosphorylation | 18 | 1.37 | 100 | 1 | 19.11 | 714.084 | -8 | 5718.729 | 5721.05 | 5718.7212 | 2453554.25 |
| 3216-3233 | Ur-pCr-pUr-pGr-pCr-pCr-pCr-pCr-pUr-pCr-pAr-pCr-pGr-pGr-pCr | Cyclic phosphorylation | 18 | 1.2 | 100 | 1.1 | 19.12 | 634.63 | -9 | 5718.728 | 5721.28 | 5718.7212 | 1153875.25 |
| 3216-3233 | Ur-pCr-pUr-pGr-pCr-pCr-pCr-pCr-pUr-pCr-pAr-pCr-pGr-pGr-pCr | Cyclic phosphorylation | 18 | 1.96 | 100 | 1.2 | 19.13 | 1905.905 | -3 | 5718.7324 | 5721.25 | 5718.7212 | 1084381 |
| 2022-2034 | Ar-pAr-pCr-pAr-pAr-pUr-pAr-pGr-pCr-pUr-pAr-pCr-pGr | Cyclic phosphorylation | 13 | 1.29 | 100 | 1 | 19.13 | 1396.524 | -3 | 4191.5898 | 4193.3 | 4191.5845 | 1050961.12 |
| 2022-2034 | Ar-pAr-pCr-pAr-pAr-pUr-pAr-pGr-pCr-pUr-pAr-pCr-pGr | Cyclic phosphorylation | 13 | 1.75 | 100 | 1 | 19.13 | 697.758 | -6 | 4191.5918 | 4193.32 | 4191.5845 | 1324836.5 |
| 2022-2034 | Ar-pAr-pCr-pAr-pAr-pUr-pAr-pGr-pCr-pUr-pAr-pCr-pGr | Cyclic phosphorylation | 13 | 1.4 | 100 | 1 | 19.13 | 837.511 | -5 | 4191.5903 | 4193.29 | 4191.5845 | 943027.94 |
| 4025-4039 | Cr-pAr-pCr-pGr-pCr-pAr-pGr-pCr-pAr-pAr-pUr-pGr-pCr-pAr-pGr | Cyclic phosphorylation | 15 | 1.81 | 100 | 1 | 19.2 | 808.942 | -6 | 4856.6929 | 4859.29 | 4856.6841 | 1098077.12 |
| 4025-4039 | Cr-pAr-pCr-pGr-pCr-pAr-pGr-pCr-pAr-pAr-pUr-pGr-pCr-pAr-pGr | Cyclic phosphorylation | 15 | 1.51 | 100 | 1 | 19.2 | 1618.556 | -3 | 4856.6914 | 4859.03 | 4856.6841 | 816971.5 |
| 4025-4039 | Cr-pAr-pCr-pGr-pCr-pAr-pGr-pCr-pAr-pAr-pUr-pGr-pCr-pAr-pGr | Cyclic phosphorylation | 15 | 1.71 | 100 | 1.1 | 19.22 | 693.091 | -7 | 4856.6924 | 4859.01 | 4856.6841 | 532081.69 |
| 2577-2593 | Gr-pGr-pCr-pGr-pAr-pCr-pAr-pUr-pUr-pGr-pCr-pCr-pGr-pCr-pCr | Cyclic phosphorylation | 17 | 1.46 | 100 | 1 | 19.22 | 784.958 | -7 | 5499.7485 | 5502.15 | 5499.7405 | 497066 |
| 2916-2930 | Ar-pAr-pUr-pGr-pCr-pCr-pCr-pAr-pGr-pGr-pCr-pAr-pCr-pUr-pGr | Cyclic phosphorylation | 15 | 1.69 | 100 | 1 | 19.43 | 965.926 | -5 | 4833.665 | 4835.82 | 4833.6569 | 625651.5 |
| 2916-2930 | Ar-pAr-pUr-pGr-pCr-pCr-pCr-pAr-pGr-pGr-pCr-pAr-pCr-pUr-pGr | Cyclic phosphorylation | 15 | 1.49 | 100 | 1 | 19.44 | 689.802 | -7 | 4833.6641 | 4835.81 | 4833.6569 | 2510034.5 |
| 2916-2930 | Ar-pAr-pUr-pGr-pCr-pCr-pCr-pAr-pGr-pGr-pCr-pAr-pCr-pUr-pGr | Cyclic phosphorylation | 15 | 1.79 | 100 | 1 | 19.44 | 1207.909 | -4 | 4833.6655 | 4835.81 | 4833.6569 | 444612.97 |
| 2916-2930 | Ar-pAr-pUr-pGr-pCr-pCr-pCr-pAr-pGr-pGr-pCr-pAr-pCr-pUr-pGr | Cyclic phosphorylation | 15 | 1.29 | 100 | 1 | 19.46 | 1610.881 | -3 | 4833.6631 | 4835.82 | 4833.6569 | 1632667.62 |
| 2916-2930 | Ar-pAr-pUr-pGr-pCr-pCr-pCr-pAr-pGr-pGr-pCr-pAr-pCr-pUr-pGr | Cyclic phosphorylation | 15 | 1.39 | 100 | 1 | 19.46 | 804.937 | -6 | 4833.6636 | 4835.85 | 4833.6569 | 1552332.12 |
| 1953-1969 | Cr-pGr-pGr-pGr-pUr-pGr-pUr-pAr-pCr-pUr-pCr-pCr-pAr-pCr-pCr | Cyclic phosphorylation | 17 | 1.36 | 100 | 1.1 | 19.53 | 911.947 | -6 | 5476.7207 | 5479.12 | 5476.7133 | 580152.12 |
| 1953-1969 | Cr-pGr-pGr-pGr-pUr-pGr-pUr-pAr-pCr-pUr-pCr-pCr-pAr-pCr-pCr | Cyclic phosphorylation | 17 | 1.98 | 100 | 1 | 19.55 | 781.668 | -7 | 5476.7241 | 5478.96 | 5476.7133 | 918245.12 |
| 986-1000 | Cr-pAr-pUr-pCr-pUr-pAr-pCr-pCr-pAr-pGr-pAr-pCr-pCr-pAr-pGr | Cyclic phosphorylation | 15 | 1.63 | 100 | 1 | 19.55 | 795.437 | -6 | 4777.6636 | 4779.71 | 4777.6558 | 962092.19 |
| 986-1000 | Cr-pAr-pUr-pCr-pUr-pAr-pCr-pCr-pAr-pGr-pAr-pCr-pCr-pAr-pGr | Cyclic phosphorylation | 15 | 1.32 | 100 | 1 | 19.55 | 1591.881 | -3 | 4777.6621 | 4779.71 | 4777.6558 | 909025.19 |
| 986-1000 | Cr-pAr-pUr-pCr-pUr-pAr-pCr-pCr-pAr-pGr-pAr-pCr-pCr-pAr-pGr | Cyclic phosphorylation | 15 | 2.14 | 100 | 1 | 19.58 | 681.659 | -7 | 4777.666 | 4779.64 | 4777.6558 | 934920.94 |
| 3985-4002 | Cr-pCr-pCr-pCr-pAr-pCr-pUr-pCr-pAr-pCr-pCr-pAr-pCr-pCr-pUr- | Cyclic phosphorylation | 18 | 2.06 | 100 | 1.1 | 19.72 | 622.076 | -9 | 5605.7466 | 5608.23 | 5605.7351 | 1544843.88 |
| 948-962 | Ar-pCr-pAr-pAr-pAr-pGr-pUr-pGr-pCr-pAr-pCr-pCr-pCr-pUr-pGr | Cyclic phosphorylation | 15 | 1.05 | 100 | 1 | 19.72 | 1605.549 | -3 | 4817.667 | 4819.87 | 4817.6619 | 741290.56 |
| 948-962 | Ar-pCr-pAr-pAr-pAr-pGr-pUr-pGr-pCr-pAr-pCr-pCr-pCr-pUr-pGr | Cyclic phosphorylation | 15 | 2.87 | 100 | 1.2 | 19.72 | 687.375 | -7 | 4817.6758 | 4819.62 | 4817.6619 | 1331672.5 |
| 3985-4002 | Cr-pCr-pCr-pCr-pAr-pCr-pUr-pCr-pAr-pCr-pCr-pAr-pCr-pCr-pUr- | Cyclic phosphorylation | 18 | 1.62 | 100 | 1.1 | 19.74 | 1868.242 | -3 | 5605.7441 | 5608.22 | 5605.7351 | 4982385 |
| 3985-4002 | Cr-pCr-pCr-pCr-pAr-pCr-pUr-pCr-pAr-pCr-pCr-pAr-pCr-pCr-pUr- | Cyclic phosphorylation | 18 | 1.19 | 100 | 1 | 19.74 | 699.96 | -8 | 5605.7417 | 5608.05 | 5605.7351 | 9126507 |
| 3985-4002 | Cr-pCr-pCr-pCr-pAr-pCr-pUr-pCr-pAr-pCr-pCr-pAr-pCr-pCr-pUr- | Cyclic phosphorylation | 18 | 1.62 | 100 | 1 | 19.74 | 1120.542 | -5 | 5605.7441 | 5608.06 | 5605.7351 | 2001262 |
| 3985-4002 | Cr-pCr-pCr-pCr-pAr-pCr-pUr-pCr-pAr-pCr-pCr-pAr-pCr-pCr-pUr- | Cyclic phosphorylation | 18 | 1.27 | 100 | 1 | 19.76 | 800.099 | -7 | 5605.7422 | 5608.2 | 5605.7351 | 8353898 |
| 3985-4002 | Cr-pCr-pCr-pCr-pAr-pCr-pUr-pCr-pAr-pCr-pCr-pAr-pCr-pCr-pUr- | Cyclic phosphorylation | 18 | 1.45 | 100 | 1 | 19.76 | 933.617 | -6 | 5605.7432 | 5608.2 | 5605.7351 | 4318653 |
| 3985-4002 | Cr-pCr-pCr-pCr-pAr-pCr-pUr-pCr-pAr-pCr-pCr-pAr-pCr-pCr-pUr- | Cyclic phosphorylation | 18 | 1.1 | 100 | 1 | 19.77 | 1400.929 | -4 | 5605.7412 | 5608.1 | 5605.7351 | 1886007.12 |
| 1786-1800 | Ar-pUr-pCr-pCr-pCr-pCr-pAr-pGr-pAr-pCr-pAr-pCr-pUr-pGr-pGr | Cyclic phosphorylation | 15 | 1.77 | 99.7 | 1 | 19.84 | 684.088 | -7 | 4793.6592 | 4795.72 | 4793.6507 | 592211.31 |
| 1786-1800 | Ar-pUr-pCr-pCr-pCr-pCr-pAr-pGr-pAr-pCr-pAr-pCr-pUr-pGr-pGr | Cyclic phosphorylation | 15 | 1.77 | 99.7 | 1.1 | 19.86 | 1597.546 | -3 | 4793.6592 | 4795.7 | 4793.6507 | 443554.81 |
| 23-38 | Gr-pUr-pCr-pCr-pCr-pCr-pAr-pCr-pAr-pGr-pAr-pCr-pUr-pCr-pAr | Cyclic phosphorylation | 16 | 1.7 | 98.7 | 1 | 19.91 | 727.665 | -7 | 5098.7007 | 5100.92 | 5098.692 | 498182.78 |
| 23-38 | Gr-pUr-pCr-pCr-pCr-pCr-pAr-pCr-pAr-pGr-pAr-pCr-pUr-pCr-pAr | Cyclic phosphorylation | 16 | 1.7 | 98.7 | 1.6 | 19.93 | 1699.228 | -3 | 5098.7007 | 5100.9 | 5098.692 | 322177.25 |
| 23-38 | Gr-pUr-pCr-pCr-pCr-pCr-pAr-pCr-pAr-pGr-pAr-pCr-pUr-pCr-pAr | Cyclic phosphorylation | 16 | 1.61 | 98.7 | 1 | 19.95 | 849.11 | -6 | 5098.7002 | 5100.9 | 5098.692 | 298603.22 |
| 1329-1345 | Cr-pCr-pCr-pGr-pAr-pCr-pGr-pAr-pCr-pUr-pUr-pCr-pAr-pCr-pCr | Cyclic phosphorylation | 17 | 2.13 | 99.8 | 1 | 20.18 | 773.527 | -7 | 5419.7397 | 5422.13 | 5419.7282 | 407352.81 |
| 3112-3129 | Cr-pCr-pUr-pCr-pUr-pGr-pCr-pCr-pAr-pAr-pUr-pCr-pUr-pGr-pGr | Cyclic phosphorylation | 18 | 1.93 | 99.5 | 1 | 20.19 | 813.956 | -7 | 5702.7373 | 5705.25 | 5702.7263 | 720727.62 |
| 3112-3129 | Cr-pCr-pUr-pCr-pUr-pGr-pCr-pCr-pAr-pAr-pUr-pCr-pUr-pGr-pGr | Cyclic phosphorylation | 18 | 1.85 | 99.5 | 1 | 20.21 | 712.085 | -8 | 5702.7368 | 5705.07 | 5702.7263 | 688200.5 |
| 567-584 | Ur-pCr-pCr-pCr-pAr-pGr-pCr-pCr-pUr-pUr-pUr-pCr-pCr-pUr-pGr | Cyclic phosphorylation | 18 | 1.78 | 100 | 1.1 | 20.24 | 808.522 | -7 | 5664.6982 | 5667.04 | 5664.6882 | 1406784.12 |
| 567-584 | Ur-pCr-pCr-pCr-pAr-pGr-pCr-pCr-pUr-pUr-pUr-pCr-pCr-pUr-pGr | Cyclic phosphorylation | 18 | 2.04 | 100 | 1.6 | 20.26 | 628.627 | -9 | 5664.6997 | 5667.07 | 5664.6882 | 826109.31 |
| 567-584 | Ur-pCr-pCr-pCr-pAr-pGr-pCr-pCr-pUr-pUr-pUr-pCr-pCr-pUr-pGr | Cyclic phosphorylation | 18 | 2.12 | 100 | 1 | 20.26 | 707.33 | -8 | 5664.7002 | 5667.18 | 5664.6882 | 1510976.62 |
| 567-584 | Ur-pCr-pCr-pCr-pAr-pGr-pCr-pCr-pUr-pUr-pUr-pCr-pCr-pUr-pGr | Cyclic phosphorylation | 18 | 1.43 | 100 | 1.8 | 20.26 | 1415.418 | -4 | 5664.6963 | 5667.19 | 5664.6882 | 350318.72 |
| 567-584 | Ur-pCr-pCr-pCr-pAr-pGr-pCr-pCr-pUr-pUr-pUr-pCr-pCr-pUr-pGr | Cyclic phosphorylation | 18 | 2.3 | 100 | 1.1 | 20.26 | 1887.894 | -3 | 5664.7012 | 5667.21 | 5664.6882 | 825218.19 |
| 567-584 | Ur-pCr-pCr-pCr-pAr-pGr-pCr-pCr-pUr-pUr-pUr-pCr-pCr-pUr-pGr | Cyclic phosphorylation | 18 | 2.47 | 100 | 1.8 | 20.28 | 1132.334 | -5 | 5664.7021 | 5667.17 | 5664.6882 | 324927.5 |
| 567-584 | Ur-pCr-pCr-pCr-pAr-pGr-pCr-pCr-pUr-pUr-pUr-pCr-pCr-pUr-pGr | Cyclic phosphorylation | 18 | 2.04 | 100 | 1.1 | 20.28 | 943.443 | -6 | 5664.6997 | 5667.2 | 5664.6882 | 522772.88 |
| 245-261 | Gr-pUr-pUr-pCr-pCr-pAr-pCr-pGr-pCr-pCr-pAr-pUr-pCr-pCr-pAr | Cyclic phosphorylation | 17 | 2.09 | 99 | 1.2 | 20.31 | 767.81 | -7 | 5380.7173 | 5382.91 | 5380.7061 | 231401.59 |
| 3742-3757 | Ur-pCr-pAr-pCr-pAr-pAr-pUr-pCr-pAr-pUr-pGr-pCr-pUr-pGr-pU | Cyclic phosphorylation | 16 | 1.91 | 100 | 1 | 20.51 | 849.435 | -6 | 5101.6538 | 5103.73 | 5101.644 | 343831.91 |
| 1273-1290 | Ur-pGr-pCr-pGr-pGr-pCr-pAr-pGr-pAr-pUr-pUr-pGr-pCr-pCr-pC | Cyclic phosphorylation | 18 | 1.72 | 99.9 | 1 | 20.52 | 825.386 | -7 | 5782.7485 | 5785.25 | 5782.7386 | 382039.88 |
| 3742-3757 | Ur-pCr-pAr-pCr-pAr-pAr-pUr-pCr-pAr-pUr-pGr-pCr-pUr-pGr-pU | Cyclic phosphorylation | 16 | 2.49 | 100 | 1.1 | 20.52 | 1700.212 | -3 | 5101.6567 | 5103.84 | 5101.644 | 384153.66 |
| 3742-3757 | Ur-pCr-pAr-pCr-pAr-pAr-pUr-pCr-pAr-pUr-pGr-pCr-pUr-pGr-pU | Cyclic phosphorylation | 16 | 1.82 | 100 | 1 | 20.53 | 728.087 | -7 | 5101.6533 | 5103.88 | 5101.644 | 607634.25 |
| 2424-2439 | Ar-pCr-pCr-pCr-pCr-pUr-pCr-pCr-pUr-pAr-pUr-pCr-pAr-pAr-pGr | Cyclic phosphorylation | 16 | 1.85 | 99.9 | 1.1 | 20.77 | 722.09 | -7 | 5059.6792 | 5061.75 | 5059.6699 | 356007.56 |
| 1582-1599 | Ur-pGr-pGr-pUr-pGr-pGr-pUr-pGr-pCr-pUr-pGr-pAr-pGr-pCr-pU | Cyclic phosphorylation | 18 | 1.56 | 99.9 | 1 | 20.79 | 729.333 | -8 | 5840.7168 | 5843.38 | 5840.7077 | 532441.44 |
| 1582-1599 | Ur-pGr-pGr-pUr-pGr-pGr-pUr-pGr-pCr-pUr-pGr-pAr-pGr-pCr-pU | Cyclic phosphorylation | 18 | 1.4 | 99.9 | 1 | 20.79 | 833.667 | -7 | 5840.7158 | 5843.2 | 5840.7077 | 486112.25 |
| 3502-3515 | Ar-pGr-pGr-pAr-pAr-pCr-pUr-pGr-pGr-pAr-pCr-pAr-pAr-pGr | Cyclic phosphorylation | 14 | 1.37 | 96.1 | 1 | 20.79 | 768.438 | -6 | 4615.6665 | 4617.63 | 4615.6602 | 344289.06 |
| 3511-3524 | Ar-pCr-pAr-pAr-pGr-pUr-pAr-pCr-pUr-pUr-pUr-pAr-pAr-pGr | Cyclic phosphorylation | 14 | 1.73 | 100 | 1 | 20.91 | 898.913 | -5 | 4498.6016 | 4500.44 | 4498.5938 | 467280.66 |
| 3511-3524 | Ar-pCr-pAr-pAr-pGr-pUr-pAr-pCr-pUr-pUr-pUr-pAr-pAr-pGr | Cyclic phosphorylation | 14 | 1.19 | 100 | 1 | 20.91 | 749.093 | -6 | 4498.5991 | 4500.56 | 4498.5938 | 1122566.62 |
| 3511-3524 | Ar-pCr-pAr-pAr-pGr-pUr-pAr-pCr-pUr-pUr-pUr-pAr-pAr-pGr | Cyclic phosphorylation | 14 | 1.73 | 100 | 1.1 | 20.93 | 641.793 | -7 | 4498.6016 | 4500.56 | 4498.5938 | 1045473.19 |
| 3511-3524 | Ar-pCr-pAr-pAr-pGr-pUr-pAr-pCr-pUr-pUr-pUr-pAr-pAr-pGr | Cyclic phosphorylation | 14 | 1.52 | 100 | 1 | 20.93 | 1498.86 | -3 | 4498.6006 | 4500.56 | 4498.5938 | 1087104 |
| 4013-4028 | Ar-pCr-pAr-pCr-pCr-pUr-pCr-pCr-pCr-pAr-pAr-pGr-pCr-pAr-pCr- | Cyclic phosphorylation | 16 | 1.6 | 100 | 1 | 21 | 725.239 | -7 | 5081.7212 | 5083.9 | 5081.7131 | 1160043.5 |
| 4013-4028 | Ar-pCr-pAr-pCr-pCr-pUr-pCr-pCr-pCr-pAr-pAr-pGr-pCr-pAr-pCr- | Cyclic phosphorylation | 16 | 1.5 | 100 | 1 | 21.01 | 846.28 | -6 | 5081.7207 | 5083.91 | 5081.7131 | 791525.81 |
| 4013-4028 | Ar-pCr-pAr-pCr-pCr-pUr-pCr-pCr-pCr-pAr-pAr-pGr-pCr-pAr-pCr- | Cyclic phosphorylation | 16 | 1.6 | 100 | 1.1 | 21.03 | 1693.568 | -3 | 5081.7212 | 5083.91 | 5081.7131 | 976000.88 |
| 2628-2645 | Ar-pCr-pAr-pGr-pUr-pGr-pCr-pUr-pGr-pCr-pCr-pUr-pCr-pCr-pUr | Cyclic phosphorylation | 18 | 1.83 | 99.5 | 1 | 21.07 | 712.208 | -8 | 5703.7207 | 5706.33 | 5703.7103 | 793559.69 |
| 3985-4002 | Cr-pCr-pCr-pCr-pAr-pCr-pUr-pCr-pAr-pCr-pCr-pAr-pCr-pCr-pUr- | Phosphorylation | 18 | 2.08 | 100 | 1 | 21.14 | 802.673 | -7 | 5623.7573 | 5626.26 | 5623.7456 | 539567.5 |
| 3985-4002 | Cr-pCr-pCr-pCr-pAr-pCr-pUr-pCr-pAr-pCr-pCr-pAr-pCr-pCr-pUr- | Phosphorylation | 18 | 1.82 | 100 | 1 | 21.17 | 936.619 | -6 | 5623.7559 | 5626.13 | 5623.7456 | 510097.22 |
| 1794-1808 | Ar-pCr-pAr-pCr-pUr-pGr-pGr-pAr-pAr-pAr-pUr-pCr-pCr-pUr-pG | Cyclic phosphorylation | 15 | 2.95 | 98.4 | 1 | 21.28 | 802.269 | -6 | 4818.6602 | 4820.43 | 4818.646 | 581147.88 |
| 3486-3501 | Cr-pUr-pGr-pGr-pAr-pCr-pAr-pGr-pCr-pUr-pUr-pCr-pAr-pAr-pA | Cyclic phosphorylation | 16 | 2.36 | 100 | 1.6 | 21.29 | 644.581 | -8 | 5163.7056 | 5165.87 | 5163.6934 | 495233.19 |
| 3486-3501 | Cr-pUr-pGr-pGr-pAr-pCr-pAr-pGr-pCr-pUr-pUr-pCr-pAr-pAr-pA | Cyclic phosphorylation | 16 | 1.51 | 100 | 1 | 21.33 | 736.951 | -7 | 5163.7012 | 5165.88 | 5163.6934 | 1312472.38 |
| 3486-3501 | Cr-pUr-pGr-pGr-pAr-pCr-pAr-pGr-pCr-pUr-pUr-pCr-pAr-pAr-pA | Cyclic phosphorylation | 16 | 1.79 | 100 | 1 | 21.33 | 1720.895 | -3 | 5163.7026 | 5165.86 | 5163.6934 | 787232.25 |
| 3486-3501 | Cr-pUr-pGr-pGr-pAr-pCr-pAr-pGr-pCr-pUr-pUr-pCr-pAr-pAr-pA | Cyclic phosphorylation | 16 | 1.7 | 100 | 1 | 21.33 | 859.944 | -6 | 5163.7021 | 5165.85 | 5163.6934 | 735055.56 |
| 3216-3234 | Ur-pCr-pUr-pGr-pCr-pCr-pCr-pCr-pUr-pCr-pAr-pCr-pGr-pGr-pCr | Cyclic phosphorylation | 19 | 1.12 | 100 | 1 | 21.33 | 757.215 | -8 | 6063.7754 | 6066.39 | 6063.7686 | 525810.38 |
| 3286-3303 | Cr-pUr-pCr-pCr-pAr-pGr-pCr-pCr-pAr-pUr-pCr-pUr-pGr-pCr-pCr | Cyclic phosphorylation | 18 | 1.93 | 99.5 | 1 | 21.33 | 811.529 | -7 | 5685.7583 | 5688.51 | 5685.7473 | 1139262.38 |
| 3286-3303 | Cr-pUr-pCr-pCr-pAr-pGr-pCr-pCr-pAr-pUr-pCr-pUr-pGr-pCr-pCr | Cyclic phosphorylation | 18 | 2.18 | 99.5 | 1 | 21.34 | 710.088 | -8 | 5685.7598 | 5688.43 | 5685.7473 | 1673931.88 |
| 3286-3303 | Cr-pUr-pCr-pCr-pAr-pGr-pCr-pCr-pAr-pUr-pCr-pUr-pGr-pCr-pCr | Cyclic phosphorylation | 18 | 1.84 | 99.5 | 1.1 | 21.35 | 1894.913 | -3 | 5685.7578 | 5688.35 | 5685.7473 | 753951.44 |
| 3469-3485 | Ar-pCr-pCr-pCr-pUr-pCr-pUr-pGr-pCr-pAr-pGr-pCr-pCr-pCr-pGr | Cyclic phosphorylation | 17 | 2.04 | 99.4 | 1.9 | 21.45 | 902.617 | -6 | 5419.7393 | 5422.19 | 5419.7282 | 272050.44 |
| 3469-3485 | Ar-pCr-pCr-pCr-pUr-pCr-pUr-pGr-pCr-pAr-pGr-pCr-pCr-pCr-pGr | Cyclic phosphorylation | 17 | 1.68 | 99.4 | 1.9 | 21.45 | 1806.574 | -3 | 5419.7373 | 5422.24 | 5419.7282 | 355922.31 |
| 3469-3485 | Ar-pCr-pCr-pCr-pUr-pCr-pUr-pGr-pCr-pAr-pGr-pCr-pCr-pCr-pGr | Cyclic phosphorylation | 17 | 1.59 | 99.4 | 1.2 | 21.47 | 676.71 | -8 | 5419.7368 | 5422.11 | 5419.7282 | 530034.12 |
| 3469-3485 | Ar-pCr-pCr-pCr-pUr-pCr-pUr-pGr-pCr-pAr-pGr-pCr-pCr-pCr-pGr | Cyclic phosphorylation | 17 | 1.68 | 99.4 | 1 | 21.47 | 773.527 | -7 | 5419.7373 | 5422.16 | 5419.7282 | 558905.81 |
| 3832-3848 | Ar-pUr-pUr-pCr-pUr-pGr-pAr-pGr-pCr-pCr-pCr-pGr-pUr-pGr-pC | Cyclic phosphorylation | 17 | 1.55 | 97.5 | 1 | 21.56 | 776.234 | -7 | 5438.6836 | 5441.34 | 5438.6752 | 487298.5 |
| 1868-1883 | Cr-pAr-pAr-pUr-pCr-pAr-pGr-pGr-pUr-pGr-pGr-pCr-pAr-pGr-pU | Cyclic phosphorylation | 16 | 2.32 | 91.7 | 1.1 | 21.56 | 744.951 | -7 | 5219.7065 | 5221.81 | 5219.6945 | 346028.81 |
| 78-95 | Cr-pCr-pUr-pCr-pUr-pGr-pGr-pUr-pGr-pUr-pCr-pCr-pAr-pGr-pC | Cyclic phosphorylation | 18 | 2.12 | 100 | 1.1 | 21.57 | 819.814 | -7 | 5742.7446 | 5745.4 | 5742.7324 | 477336.41 |
| 78-95 | Cr-pCr-pUr-pCr-pUr-pGr-pGr-pUr-pGr-pUr-pCr-pCr-pAr-pGr-pC | Cyclic phosphorylation | 18 | 1.87 | 100 | 1.1 | 21.59 | 717.086 | -8 | 5742.7432 | 5745.21 | 5742.7324 | 451596.62 |
| 3738-3752 | Ar-pUr-pGr-pGr-pUr-pCr-pAr-pCr-pAr-pAr-pUr-pCr-pAr-pUr-pG | Cyclic phosphorylation | 15 | 1.5 | 100 | 1 | 21.7 | 1606.207 | -3 | 4819.6372 | 4821.71 | 4819.63 | 974902.19 |
| 3738-3752 | Ar-pUr-pGr-pGr-pUr-pCr-pAr-pCr-pAr-pAr-pUr-pCr-pAr-pUr-pG | Cyclic phosphorylation | 15 | 1.6 | 100 | 1 | 21.7 | 687.655 | -7 | 4819.6377 | 4821.68 | 4819.63 | 1209235.12 |
| 3738-3752 | Ar-pUr-pGr-pGr-pUr-pCr-pAr-pCr-pAr-pAr-pUr-pCr-pAr-pUr-pG | Cyclic phosphorylation | 15 | 2.01 | 100 | 1 | 21.7 | 802.433 | -6 | 4819.6396 | 4821.79 | 4819.63 | 663263.38 |
| 2545-2561 | Cr-pCr-pGr-pGr-pCr-pUr-pUr-pCr-pAr-pUr-pCr-pAr-pAr-pGr-pCr | Cyclic phosphorylation | 17 | 1.47 | 97.6 | 1 | 21.71 | 679.835 | -8 | 5444.7314 | 5447 | 5444.7234 | 1160806.25 |

| | | | | | | | | | | | | | |
|---|---|---|---|---|---|---|---|---|---|---|---|---|---|
| 2545-2561 | Cr-pCr-pGr-pGr-pCr-pUr-pUr-pCr-pAr-pUr-pCr-pAr-pAr-pGr-pC | Cyclic phosphorylation | 17 | 1.38 | 97.6 | 1 | 21.71 | 776.954 | -7 | 5444.731 | 5446.86 | 5444.7234 | 1175999.62 |
| 1312-1325 | Ar-pCr-pUr-pAr-pCr-pAr-pAr-pCr-pUr-pAr-pCr-pAr-pAr-pGr | Cyclic phosphorylation | 14 | 0.88 | 100 | 1 | 21.77 | 745.932 | -6 | 4480.6348 | 4482.58 | 4480.6308 | 3360979.5 |
| 1312-1325 | Ar-pCr-pUr-pAr-pCr-pAr-pAr-pCr-pUr-pAr-pCr-pAr-pAr-pGr | Cyclic phosphorylation | 14 | 1.53 | 100 | 1.1 | 21.77 | 639.227 | -7 | 4480.6377 | 4482.48 | 4480.6308 | 1967632.88 |
| 1312-1325 | Ar-pCr-pUr-pAr-pCr-pAr-pAr-pCr-pUr-pAr-pCr-pAr-pAr-pGr | Cyclic phosphorylation | 14 | 0.99 | 100 | 1 | 21.77 | 1493.206 | -3 | 4480.6353 | 4482.61 | 4480.6308 | 2684392.25 |
| 1312-1325 | Ar-pCr-pUr-pAr-pCr-pAr-pAr-pCr-pUr-pAr-pCr-pAr-pAr-pGr | Cyclic phosphorylation | 14 | 1.32 | 100 | 1 | 21.78 | 1119.653 | -4 | 4480.6367 | 4482.61 | 4480.6308 | 694800.31 |
| 1312-1325 | Ar-pCr-pUr-pAr-pCr-pAr-pAr-pCr-pUr-pAr-pCr-pAr-pAr-pGr | Cyclic phosphorylation | 14 | 1.53 | 100 | 1 | 21.8 | 895.321 | -5 | 4480.6377 | 4482.59 | 4480.6308 | 1386335.12 |
| 3042-3056 | Ar-pUr-pCr-pAr-pCr-pAr-pGr-pGr-pCr-pAr-pGr-pAr-pCr-pUr-pG | Cyclic phosphorylation | 15 | 1.18 | 100 | 1 | 21.83 | 970.929 | -5 | 4857.6738 | 4859.78 | 4857.6681 | 494940.12 |
| 3042-3056 | Ar-pUr-pCr-pAr-pCr-pAr-pGr-pGr-pCr-pAr-pGr-pAr-pCr-pUr-pG | Cyclic phosphorylation | 15 | 1.38 | 100 | 1 | 21.85 | 808.939 | -6 | 4857.6748 | 4859.81 | 4857.6681 | 1277981 |
| 3878-3893 | Ar-pCr-pUr-pCr-pGr-pAr-pGr-pCr-pUr-pGr-pGr-pUr-pAr-pCr-pU | Cyclic phosphorylation | 16 | 2.19 | 99.3 | 1 | 21.85 | 735.803 | -7 | 5156.6724 | 5158.82 | 5156.6611 | 661274.56 |
| 3878-3893 | Ar-pCr-pUr-pCr-pGr-pAr-pGr-pCr-pUr-pGr-pGr-pUr-pAr-pCr-pU | Cyclic phosphorylation | 16 | 2.28 | 99.3 | 1.1 | 21.87 | 858.771 | -6 | 5156.6729 | 5158.8 | 5156.6611 | 408405 |
| 3042-3056 | Ar-pUr-pCr-pAr-pCr-pAr-pGr-pGr-pCr-pAr-pGr-pAr-pCr-pUr-pG | Cyclic phosphorylation | 15 | 0.98 | 100 | 1 | 21.87 | 1618.886 | -3 | 4857.6729 | 4859.75 | 4857.6681 | 1550524.62 |
| 3042-3056 | Ar-pUr-pCr-pAr-pCr-pAr-pGr-pGr-pCr-pAr-pGr-pAr-pCr-pUr-pG | Cyclic phosphorylation | 15 | 1.18 | 100 | 1 | 21.87 | 693.232 | -7 | 4857.6738 | 4859.73 | 4857.6681 | 2315430 |
| 3849-3863 | Ar-pAr-pGr-pGr-pGr-pCr-pGr-pUr-pGr-pAr-pAr-pAr-pCr-pUr-pG | Cyclic phosphorylation | 15 | 1.54 | 100 | 1.1 | 22.01 | 1645.557 | -3 | 4937.688 | 4939.83 | 4937.6804 | 407404.62 |
| 3849-3863 | Ar-pAr-pGr-pGr-pGr-pCr-pGr-pUr-pGr-pAr-pAr-pAr-pCr-pUr-pG | Cyclic phosphorylation | 15 | 1.74 | 100 | 1 | 22.02 | 704.663 | -7 | 4937.689 | 4939.83 | 4937.6804 | 792050.12 |
| 3600-3615 | Ar-pUr-pCr-pGr-pAr-pCr-pCr-pGr-pGr-pCr-pUr-pGr-pAr-pAr-pC | Cyclic phosphorylation | 16 | 1.85 | 97.8 | 1.1 | 22.04 | 739.095 | -7 | 5178.7139 | 5180.93 | 5178.7043 | 424688.47 |
| 1728-1745 | Ur-pUr-pCr-pCr-pUr-pGr-pCr-pCr-pAr-pUr-pUr-pCr-pCr-pAr-pG | Cyclic phosphorylation | 18 | 2.48 | 100 | 1.2 | 22.22 | 947.281 | -6 | 5687.7295 | 5690.16 | 5687.7154 | 473083.69 |
| 1728-1745 | Ur-pUr-pCr-pCr-pUr-pGr-pCr-pCr-pAr-pUr-pUr-pCr-pCr-pAr-pG | Cyclic phosphorylation | 18 | 2.74 | 100 | 1 | 22.22 | 710.209 | -8 | 5687.731 | 5690.04 | 5687.7154 | 1779988.12 |
| 1728-1745 | Ur-pUr-pCr-pCr-pUr-pGr-pCr-pCr-pAr-pUr-pUr-pCr-pCr-pAr-pG | Cyclic phosphorylation | 18 | 2.91 | 100 | 1 | 22.22 | 811.668 | -7 | 5687.7319 | 5690.19 | 5687.7154 | 1113994.88 |
| 1728-1745 | Ur-pUr-pCr-pCr-pUr-pGr-pCr-pCr-pAr-pUr-pUr-pCr-pCr-pAr-pG | Cyclic phosphorylation | 18 | 2.91 | 100 | 1.1 | 22.22 | 1895.571 | -3 | 5687.7319 | 5690.01 | 5687.7154 | 855437.94 |
| 2824-2839 | Cr-pCr-pAr-pAr-pCr-pCr-pAr-pGr-pUr-pUr-pCr-pAr-pAr-pCr-pAr | Cyclic phosphorylation | 16 | 1.28 | 99.7 | 1.1 | 22.25 | 728.81 | -7 | 5106.7148 | 5109.07 | 5106.7083 | 355835.81 |
| 1728-1745 | Ur-pUr-pCr-pCr-pUr-pGr-pCr-pCr-pAr-pUr-pUr-pCr-pCr-pAr-pG | Cyclic phosphorylation | 18 | 2.4 | 100 | 1.8 | 22.25 | 631.185 | -9 | 5687.729 | 5690.23 | 5687.7154 | 416707.25 |
| 567-585 | Ur-pCr-pCr-pCr-pAr-pGr-pCr-pCr-pUr-pUr-pUr-pCr-pCr-pUr-pG | Cyclic phosphorylation | 19 | 2.07 | 99.5 | 1.2 | 22.29 | 857.957 | -7 | 6009.748 | 6012.22 | 6009.7356 | 422031.91 |
| 567-585 | Ur-pCr-pCr-pCr-pAr-pGr-pCr-pCr-pUr-pUr-pUr-pCr-pCr-pUr-pG | Cyclic phosphorylation | 19 | 2.56 | 99.5 | 1.1 | 22.3 | 750.462 | -8 | 6009.751 | 6012.3 | 6009.7356 | 658466.06 |
| 155-171 | Ar-pGr-pGr-pCr-pGr-pUr-pGr-pUr-pAr-pCr-pUr-pAr-pCr-pCr-pC | Cyclic phosphorylation | 17 | 1.41 | 93.2 | 1 | 22.32 | 779.525 | -7 | 5460.7261 | 5463.47 | 5460.7184 | 592326.81 |
| 269-284 | Cr-pAr-pCr-pCr-pAr-pAr-pUr-pGr-pGr-pCr-pAr-pCr-pCr-pAr-pAr | Cyclic phosphorylation | 16 | 2.66 | 100 | 1 | 22.39 | 856.951 | -6 | 5145.7441 | 5148.01 | 5145.7304 | 479058.06 |
| 269-284 | Cr-pAr-pCr-pCr-pAr-pAr-pUr-pGr-pGr-pCr-pAr-pCr-pCr-pAr-pAr | Cyclic phosphorylation | 16 | 2.28 | 100 | 1.1 | 22.39 | 1714.908 | -3 | 5145.7422 | 5147.93 | 5145.7304 | 631108.62 |
| 269-284 | Cr-pAr-pCr-pCr-pAr-pAr-pUr-pGr-pGr-pCr-pAr-pCr-pCr-pAr-pAr | Cyclic phosphorylation | 16 | 2.38 | 100 | 1 | 22.41 | 734.242 | -7 | 5145.7427 | 5147.9 | 5145.7304 | 878721 |
| 2004-2019 | Ar-pUr-pCr-pGr-pGr-pAr-pGr-pCr-pCr-pGr-pAr-pGr-pCr-pAr-pC | Cyclic phosphorylation | 16 | 1.99 | 100 | 1 | 22.43 | 744.67 | -7 | 5217.7368 | 5220.18 | 5217.7264 | 816922.06 |
| 2004-2019 | Ar-pUr-pCr-pGr-pGr-pAr-pGr-pCr-pCr-pGr-pAr-pGr-pCr-pAr-pC | Cyclic phosphorylation | 16 | 1.34 | 100 | 1 | 22.43 | 868.949 | -6 | 5217.7334 | 5220.04 | 5217.7264 | 383930.81 |
| 2004-2019 | Ar-pUr-pCr-pGr-pGr-pAr-pGr-pCr-pCr-pGr-pAr-pGr-pCr-pAr-pC | Cyclic phosphorylation | 16 | 2.46 | 100 | 1.1 | 22.43 | 1738.906 | -3 | 5217.7393 | 5220.28 | 5217.7264 | 474183.84 |
| 522-538 | Ur-pUr-pCr-pCr-pGr-pGr-pGr-pUr-pGr-pUr-pAr-pCr-pAr-pGr-pC | Cyclic phosphorylation | 17 | 2.21 | 97.9 | 1.5 | 22.73 | 686.958 | -8 | 5501.7207 | 5504.13 | 5501.7085 | 450836.88 |
| 522-538 | Ur-pUr-pCr-pCr-pGr-pGr-pGr-pUr-pGr-pUr-pAr-pCr-pAr-pGr-pC | Cyclic phosphorylation | 17 | 2.3 | 97.9 | 1 | 22.73 | 785.239 | -7 | 5501.7212 | 5504.22 | 5501.7085 | 370512.28 |
| 2079-2092 | Ar-pCr-pAr-pCr-pAr-pGr-pAr-pCr-pAr-pAr-pAr-pCr-pAr-pGr | Cyclic phosphorylation | 14 | 2.04 | 100 | 1 | 22.78 | 756.275 | -6 | 4542.6895 | 4544.61 | 4542.6802 | 270234.78 |
| 2079-2092 | Ar-pCr-pAr-pCr-pAr-pGr-pAr-pCr-pAr-pAr-pAr-pCr-pAr-pGr | Cyclic phosphorylation | 14 | 1.72 | 100 | 1 | 22.78 | 1513.556 | -3 | 4542.688 | 4544.66 | 4542.6802 | 261888.48 |
| 3758-3775 | Ur-pUr-pGr-pCr-pAr-pUr-pGr-pAr-pCr-pCr-pAr-pGr-pCr-pUr-pG | Cyclic phosphorylation | 18 | 2.26 | 100 | 1 | 22.81 | 823.242 | -7 | 5767.7407 | 5770.23 | 5767.7277 | 415245.19 |
| 3758-3775 | Ur-pUr-pGr-pCr-pAr-pUr-pGr-pAr-pCr-pCr-pAr-pGr-pCr-pUr-pG | Cyclic phosphorylation | 18 | 2.01 | 100 | 1 | 22.85 | 720.211 | -8 | 5767.7393 | 5770.23 | 5767.7277 | 829784.56 |
| 1914-1932 | Cr-pCr-pCr-pGr-pUr-pGr-pGr-pCr-pCr-pAr-pUr-pUr-pCr-pAr-pC | Cyclic phosphorylation | 19 | 2.4 | 100 | 1 | 22.92 | 755.093 | -8 | 6046.8042 | 6049.36 | 6046.7897 | 941628.94 |
| 1914-1932 | Cr-pCr-pCr-pGr-pUr-pGr-pGr-pCr-pCr-pAr-pUr-pUr-pCr-pAr-pC | Cyclic phosphorylation | 19 | 1.75 | 100 | 1 | 22.92 | 863.251 | -7 | 6046.8003 | 6049.46 | 6046.7897 | 766385 |
| 1914-1932 | Cr-pCr-pCr-pGr-pUr-pGr-pGr-pCr-pCr-pAr-pUr-pUr-pCr-pAr-pC | Cyclic phosphorylation | 19 | 2.16 | 100 | 1.3 | 22.92 | 671.194 | -9 | 6046.8027 | 6049.52 | 6046.7897 | 429636.16 |
| 2041-2058 | Ar-pCr-pAr-pUr-pCr-pCr-pCr-pCr-pAr-pUr-pCr-pGr-pGr-pCr-pGr | Cyclic phosphorylation | 18 | 2.2 | 100 | 1.6 | 22.98 | 1908.249 | -3 | 5725.7661 | 5728.15 | 5725.7535 | 623957 |
| 2041-2058 | Ar-pCr-pAr-pUr-pCr-pCr-pCr-pCr-pAr-pUr-pCr-pGr-pGr-pCr-pGr | Cyclic phosphorylation | 18 | 0.92 | 100 | 1 | 22.99 | 714.963 | -8 | 5725.7588 | 5728.39 | 5725.7535 | 1612709.25 |
| 2041-2058 | Ar-pCr-pAr-pUr-pCr-pCr-pCr-pCr-pAr-pUr-pCr-pGr-pGr-pCr-pGr | Cyclic phosphorylation | 18 | 1.18 | 100 | 1 | 22.99 | 817.245 | -7 | 5725.7603 | 5728.05 | 5725.7535 | 1016573.81 |
| 1394-1409 | Cr-pAr-pAr-pCr-pUr-pAr-pCr-pAr-pAr-pUr-pUr-pAr-pCr-pCr-pU | Cyclic phosphorylation | 16 | 1.68 | 100 | 1 | 23.04 | 844.106 | -6 | 5068.6787 | 5070.84 | 5068.6702 | 1155370.62 |
| 864-881 | Cr-pAr-pGr-pCr-pCr-pUr-pAr-pGr-pAr-pAr-pCr-pCr-pUr-pUr-pC | Cyclic phosphorylation | 18 | 1.55 | 98 | 1.5 | 23.06 | 633.744 | -9 | 5710.7515 | 5713.13 | 5710.7426 | 669277.69 |
| 864-881 | Cr-pAr-pGr-pCr-pCr-pUr-pAr-pGr-pAr-pAr-pCr-pCr-pUr-pUr-pC | Cyclic phosphorylation | 18 | 2.07 | 98 | 1 | 23.06 | 713.213 | -8 | 5710.7544 | 5713.36 | 5710.7426 | 1566415.25 |
| 1394-1409 | Cr-pAr-pAr-pCr-pUr-pAr-pCr-pAr-pAr-pUr-pUr-pAr-pCr-pCr-pU | Cyclic phosphorylation | 16 | 1.97 | 100 | 1.2 | 23.06 | 632.828 | -8 | 5068.6802 | 5070.88 | 5068.6702 | 1005645.38 |
| 1394-1409 | Cr-pAr-pAr-pCr-pUr-pAr-pCr-pAr-pAr-pUr-pUr-pAr-pCr-pCr-pU | Cyclic phosphorylation | 16 | 1.49 | 100 | 1 | 23.06 | 723.376 | -7 | 5068.6777 | 5070.79 | 5068.6702 | 2570200.5 |
| 1394-1409 | Cr-pAr-pAr-pCr-pUr-pAr-pCr-pAr-pAr-pUr-pUr-pAr-pCr-pCr-pU | Cyclic phosphorylation | 16 | 1.87 | 100 | 1 | 23.07 | 1689.221 | -3 | 5068.6797 | 5070.83 | 5068.6702 | 1675518.88 |
| 897-912 | Ar-pAr-pCr-pGr-pGr-pCr-pAr-pCr-pCr-pAr-pUr-pCr-pAr-pCr-pCr | Cyclic phosphorylation | 16 | 1.44 | 100 | 1 | 23.1 | 730.953 | -7 | 5121.7266 | 5124 | 5121.7192 | 4073472.75 |
| 897-912 | Ar-pAr-pCr-pGr-pGr-pCr-pAr-pCr-pCr-pAr-pUr-pCr-pAr-pCr-pCr | Cyclic phosphorylation | 16 | 1.72 | 100 | 1 | 23.11 | 1023.739 | -5 | 5121.728 | 5123.99 | 5121.7192 | 1099482.25 |
| 897-912 | Ar-pAr-pCr-pGr-pGr-pCr-pAr-pCr-pCr-pAr-pUr-pCr-pAr-pCr-pCr | Cyclic phosphorylation | 16 | 1.63 | 100 | 1 | 23.11 | 852.947 | -6 | 5121.7275 | 5124 | 5121.7192 | 2390384.5 |
| 897-912 | Ar-pAr-pCr-pGr-pGr-pCr-pAr-pCr-pCr-pAr-pUr-pCr-pAr-pCr-pCr | Cyclic phosphorylation | 16 | 1.44 | 100 | 1 | 23.11 | 1706.902 | -3 | 5121.7266 | 5124.04 | 5121.7192 | 2784261.5 |
| 897-912 | Ar-pAr-pCr-pGr-pGr-pCr-pAr-pCr-pCr-pAr-pUr-pCr-pAr-pCr-pCr | Cyclic phosphorylation | 16 | 1.91 | 100 | 1.2 | 23.11 | 639.459 | -8 | 5121.729 | 5124.1 | 5121.7192 | 1212703.25 |
| 306-323 | Cr-pCr-pCr-pUr-pUr-pCr-pAr-pAr-pCr-pGr-pAr-pCr-pGr-pGr-pG | Cyclic phosphorylation | 18 | 1.99 | 100 | 1 | 23.23 | 828.676 | -7 | 5805.7773 | 5808.12 | 5805.7658 | 945046.75 |
| 3547-3562 | Ar-pCr-pCr-pUr-pGr-pGr-pGr-pCr-pGr-pAr-pUr-pAr-pUr-pCr-pA | Cyclic phosphorylation | 16 | 1.92 | 100 | 1.1 | 23.28 | 646.706 | -8 | 5179.6982 | 5181.88 | 5179.6883 | 518228.69 |
| 3547-3562 | Ar-pCr-pCr-pUr-pGr-pGr-pGr-pCr-pGr-pAr-pUr-pAr-pUr-pCr-pA | Cyclic phosphorylation | 16 | 1.64 | 100 | 1 | 23.29 | 1035.333 | -5 | 5179.6968 | 5181.9 | 5179.6883 | 651764.06 |
| 3547-3562 | Ar-pCr-pCr-pUr-pGr-pGr-pGr-pCr-pGr-pAr-pUr-pAr-pUr-pCr-pA | Cyclic phosphorylation | 16 | 1.64 | 100 | 1 | 23.3 | 1294.418 | -4 | 5179.6968 | 5181.88 | 5179.6883 | 603788.25 |
| 3547-3562 | Ar-pCr-pCr-pUr-pGr-pGr-pGr-pCr-pGr-pAr-pUr-pAr-pUr-pCr-pA | Cyclic phosphorylation | 16 | 1.92 | 100 | 1 | 23.32 | 862.609 | -6 | 5179.6982 | 5181.91 | 5179.6883 | 1644564 |
| 3547-3562 | Ar-pCr-pCr-pUr-pGr-pGr-pGr-pCr-pGr-pAr-pUr-pAr-pUr-pCr-pA | Cyclic phosphorylation | 16 | 2.01 | 100 | 1 | 23.32 | 1726.226 | -3 | 5179.6987 | 5181.91 | 5179.6883 | 1759312 |
| 3547-3562 | Ar-pCr-pCr-pUr-pGr-pGr-pGr-pCr-pGr-pAr-pUr-pAr-pUr-pCr-pA | Cyclic phosphorylation | 16 | 1.64 | 100 | 1 | 23.32 | 739.235 | -7 | 5179.6968 | 5181.85 | 5179.6883 | 3096387 |
| 3547-3562 | Ar-pCr-pCr-pUr-pGr-pGr-pGr-pCr-pGr-pAr-pUr-pAr-pUr-pCr-pA | Cyclic phosphorylation | 16 | 1.92 | 91.6 | 1.2 | 23.35 | 646.706 | -8 | 5179.6982 | 5181.88 | 5179.6883 | 644396.62 |
| 541-558 | Cr-pCr-pAr-pAr-pCr-pAr-pAr-pCr-pUr-pGr-pCr-pAr-pCr-pCr-pUr | Cyclic phosphorylation | 18 | 1.72 | 100 | 1 | 23.37 | 812.675 | -7 | 5693.7734 | 5696.12 | 5693.7637 | 1437963.62 |
| 541-558 | Cr-pCr-pAr-pAr-pCr-pAr-pAr-pCr-pUr-pGr-pCr-pAr-pCr-pCr-pUr | Cyclic phosphorylation | 18 | 1.63 | 100 | 1 | 23.39 | 710.965 | -8 | 5693.7729 | 5696.31 | 5693.7637 | 1358567.62 |
| 541-558 | Cr-pCr-pAr-pAr-pCr-pAr-pAr-pCr-pUr-pGr-pCr-pAr-pCr-pCr-pUr | Cyclic phosphorylation | 18 | 1.8 | 100 | 1.4 | 23.39 | 1897.586 | -3 | 5693.7739 | 5696.28 | 5693.7637 | 894983.5 |
| 541-558 | Cr-pCr-pAr-pAr-pCr-pAr-pAr-pCr-pUr-pGr-pCr-pAr-pCr-pCr-pUr | Cyclic phosphorylation | 18 | 1.37 | 100 | 1.4 | 23.39 | 631.857 | -9 | 5693.7715 | 5696.11 | 5693.7637 | 254391.81 |
| 2229-2246 | Ar-pUr-pCr-pCr-pUr-pGr-pCr-pCr-pUr-pGr-pUr-pGr-pUr-pCr-pC | Cyclic phosphorylation | 18 | 2.06 | 100 | 1.2 | 23.48 | 1901.229 | -3 | 5704.7061 | 5707.92 | 5704.6943 | 854502.44 |
| 2229-2246 | Ar-pUr-pCr-pCr-pUr-pGr-pCr-pCr-pUr-pGr-pUr-pGr-pUr-pCr-pC | Cyclic phosphorylation | 18 | 1.8 | 100 | 1 | 23.48 | 814.237 | -7 | 5704.7046 | 5708.1 | 5704.6943 | 1188693.88 |
| 2229-2246 | Ar-pUr-pCr-pCr-pUr-pGr-pCr-pCr-pUr-pGr-pUr-pGr-pUr-pCr-pC | Cyclic phosphorylation | 18 | 1.63 | 100 | 1 | 23.48 | 712.331 | -8 | 5704.7036 | 5707.22 | 5704.6943 | 2389145.5 |
| 2229-2246 | Ar-pUr-pCr-pCr-pUr-pGr-pCr-pCr-pUr-pGr-pUr-pGr-pUr-pCr-pC | Cyclic phosphorylation | 18 | 2.32 | 100 | 1.3 | 23.49 | 633.071 | -9 | 5704.7075 | 5707.21 | 5704.6943 | 1296890.5 |
| 2229-2246 | Ar-pUr-pCr-pCr-pUr-pGr-pCr-pCr-pUr-pGr-pUr-pGr-pUr-pCr-pC | Cyclic phosphorylation | 18 | 1.55 | 100 | 1.5 | 23.49 | 950.111 | -6 | 5704.7031 | 5706.72 | 5704.6943 | 466884.97 |
| 3308-3325 | Cr-pAr-pAr-pAr-pGr-pCr-pCr-pCr-pAr-pCr-pUr-pUr-pUr-pCr-pCr | Cyclic phosphorylation | 18 | 3.07 | 99.1 | 1.2 | 23.66 | 710.963 | -8 | 5694.7651 | 5697.03 | 5694.7477 | 446487.75 |
| 3308-3325 | Cr-pAr-pAr-pAr-pGr-pCr-pCr-pCr-pAr-pCr-pUr-pUr-pUr-pCr-pCr | Cyclic phosphorylation | 18 | 2.72 | 99.1 | 1 | 23.67 | 812.816 | -7 | 5694.7632 | 5697.08 | 5694.7477 | 409720.34 |
| 3525-3540 | Ar-pAr-pCr-pCr-pAr-pCr-pAr-pCr-pAr-pAr-pGr-pCr-pCr-pCr-pCr- | Cyclic phosphorylation | 16 | 1.9 | 100 | 1.1 | 23.78 | 637.212 | -8 | 5104.75 | 5106.92 | 5104.7403 | 1176782.62 |
| 3525-3540 | Ar-pAr-pCr-pCr-pAr-pCr-pAr-pCr-pAr-pAr-pGr-pCr-pCr-pCr-pCr- | Cyclic phosphorylation | 16 | 1.71 | 100 | 1 | 23.79 | 728.528 | -7 | 5104.749 | 5106.93 | 5104.7403 | 3537766.25 |
| 3525-3540 | Ar-pAr-pCr-pCr-pAr-pCr-pAr-pCr-pAr-pAr-pGr-pCr-pCr-pCr-pCr- | Cyclic phosphorylation | 16 | 2 | 100 | 1 | 23.79 | 1701.244 | -3 | 5104.7505 | 5106.94 | 5104.7403 | 2731673.25 |
| 1226-1242 | Cr-pUr-pUr-pCr-pAr-pCr-pAr-pAr-pAr-pCr-pGr-pUr-pGr-pUr-pA | Cyclic phosphorylation | 17 | 1.86 | 100 | 1.2 | 23.8 | 677.959 | -8 | 5429.7227 | 5431.96 | 5429.7125 | 836069.81 |
| 3525-3540 | Ar-pAr-pCr-pCr-pAr-pCr-pAr-pCr-pAr-pAr-pGr-pCr-pCr-pCr-pCr- | Cyclic phosphorylation | 16 | 1.52 | 100 | 1 | 23.81 | 850.118 | -6 | 5104.748 | 5106.92 | 5104.7403 | 2404186.5 |
| 3525-3540 | Ar-pAr-pCr-pCr-pAr-pCr-pAr-pCr-pAr-pAr-pGr-pCr-pCr-pCr-pCr- | Cyclic phosphorylation | 16 | 1.81 | 100 | 1 | 23.81 | 1275.681 | -4 | 5104.7495 | 5106.89 | 5104.7403 | 725522.5 |
| 3525-3540 | Ar-pAr-pCr-pCr-pAr-pCr-pAr-pCr-pAr-pAr-pGr-pCr-pCr-pCr-pCr- | Cyclic phosphorylation | 16 | 1.71 | 100 | 1 | 23.81 | 1020.344 | -5 | 5104.749 | 5106.95 | 5104.7403 | 1046047.56 |
| 1226-1242 | Cr-pUr-pUr-pCr-pAr-pCr-pAr-pAr-pAr-pCr-pGr-pUr-pGr-pUr-pA | Cyclic phosphorylation | 17 | 1.68 | 100 | 1.3 | 23.81 | 1809.569 | -3 | 5429.7217 | 5432.1 | 5429.7125 | 595836.25 |
| 1226-1242 | Cr-pUr-pUr-pCr-pAr-pCr-pAr-pAr-pAr-pCr-pGr-pUr-pGr-pUr-pA | Cyclic phosphorylation | 17 | 1.86 | 100 | 1 | 23.83 | 774.954 | -7 | 5429.7227 | 5432.01 | 5429.7125 | 1091708.38 |
| 1362-1376 | Ar-pAr-pCr-pAr-pGr-pCr-pAr-pAr-pCr-pAr-pAr-pCr-pCr-pUr-pGr | Cyclic phosphorylation | 15 | 1.74 | 100 | 1 | 23.88 | 803.277 | -6 | 4824.7026 | 4826.76 | 4824.6942 | 1115456 |
| 1362-1376 | Ar-pAr-pCr-pAr-pGr-pCr-pAr-pAr-pCr-pAr-pAr-pCr-pCr-pUr-pGr | Cyclic phosphorylation | 15 | 1.74 | 100 | 1 | 23.9 | 688.379 | -7 | 4824.7026 | 4826.83 | 4824.6942 | 1199114.25 |
| 1362-1376 | Ar-pAr-pCr-pAr-pGr-pCr-pAr-pAr-pCr-pAr-pAr-pCr-pCr-pUr-pGr | Cyclic phosphorylation | 15 | 1.64 | 100 | 1 | 23.9 | 1607.895 | -3 | 4824.7021 | 4826.77 | 4824.6942 | 1133544.38 |
| 1600-1617 | Ar-pAr-pCr-pUr-pGr-pCr-pUr-pGr-pCr-pAr-pUr-pGr-pCr-pCr-pC | Cyclic phosphorylation | 18 | 1.84 | 100 | 1 | 24 | 817.386 | -7 | 5726.748 | 5729.21 | 5726.7375 | 1436788.5 |
| 1600-1617 | Ar-pAr-pCr-pUr-pGr-pCr-pUr-pGr-pCr-pAr-pUr-pGr-pCr-pCr-pC | Cyclic phosphorylation | 18 | 1.93 | 100 | 1 | 24.02 | 715.087 | -8 | 5726.7485 | 5729.25 | 5726.7375 | 1408133.5 |
| 4037-4053 | Cr-pAr-pGr-pCr-pUr-pCr-pAr-pAr-pAr-pAr-pCr-pGr-pCr-pUr-pU | Cyclic phosphorylation | 17 | 2.41 | 100 | 1 | 24.06 | 1089.944 | -5 | 5452.7529 | 5455.06 | 5452.7398 | 734879.44 |
| 4037-4053 | Cr-pAr-pGr-pCr-pUr-pCr-pAr-pAr-pAr-pAr-pCr-pGr-pCr-pUr-pU | Cyclic phosphorylation | 17 | 2.24 | 100 | 1 | 24.07 | 1362.682 | -4 | 5452.752 | 5455.04 | 5452.7398 | 664812.5 |
| 4037-4053 | Cr-pAr-pGr-pCr-pUr-pCr-pAr-pAr-pAr-pAr-pCr-pGr-pCr-pUr-pU | Cyclic phosphorylation | 17 | 2.41 | 100 | 1 | 24.07 | 908.119 | -6 | 5452.7529 | 5455 | 5452.7398 | 1622614.75 |
| 4037-4053 | Cr-pAr-pGr-pCr-pUr-pCr-pAr-pAr-pAr-pAr-pCr-pGr-pCr-pUr-pU | Cyclic phosphorylation | 17 | 2.59 | 100 | 1 | 24.07 | 1817.245 | -3 | 5452.7539 | 5455.02 | 5452.7398 | 2152061.5 |
| 4037-4053 | Cr-pAr-pGr-pCr-pUr-pCr-pAr-pAr-pAr-pAr-pCr-pGr-pCr-pUr-pU | Cyclic phosphorylation | 17 | 2.24 | 100 | 1 | 24.07 | 778.243 | -7 | 5452.752 | 5455.18 | 5452.7398 | 3316809 |
| 4037-4053 | Cr-pAr-pGr-pCr-pUr-pCr-pAr-pAr-pAr-pAr-pCr-pGr-pCr-pUr-pU | Cyclic phosphorylation | 17 | 2.15 | 100 | 1 | 24.09 | 680.837 | -8 | 5452.7515 | 5455.05 | 5452.7398 | 2676067.5 |
| 3736-3752 | Ur-pGr-pAr-pUr-pGr-pGr-pUr-pCr-pAr-pCr-pAr-pAr-pUr-pCr-pA | Cyclic phosphorylation | 17 | 3.47 | 100 | 1.1 | 24.14 | 910.945 | -6 | 5470.7217 | 5473.29 | 5470.7027 | 536921.31 |
| 3736-3752 | Ur-pGr-pAr-pUr-pGr-pGr-pUr-pCr-pAr-pCr-pAr-pAr-pUr-pCr-pA | Cyclic phosphorylation | 17 | 2.58 | 100 | 1.9 | 24.16 | 683.082 | -8 | 5470.7168 | 5473.04 | 5470.7027 | 473715.97 |
| 3736-3752 | Ur-pGr-pAr-pUr-pGr-pGr-pUr-pCr-pAr-pCr-pAr-pAr-pUr-pCr-pA | Cyclic phosphorylation | 17 | 3.38 | 100 | 1.5 | 24.18 | 1823.233 | -3 | 5470.7212 | 5473.33 | 5470.7027 | 592868.81 |
| 3736-3752 | Ur-pGr-pAr-pUr-pGr-pGr-pUr-pCr-pAr-pCr-pAr-pAr-pUr-pCr-pA | Cyclic phosphorylation | 17 | 1.95 | 100 | 1 | 24.19 | 780.809 | -7 | 5470.7134 | 5473.11 | 5470.7027 | 1016441.69 |
| 268-284 | Gr-pCr-pAr-pCr-pCr-pAr-pAr-pUr-pGr-pGr-pCr-pAr-pCr-pCr-pAr | Cyclic phosphorylation | 17 | 1.95 | 97.1 | 1.2 | 24.33 | 783.678 | -7 | 5490.7886 | 5493 | 5490.7779 | 428952.81 |
| 2235-2252 | Cr-pCr-pUr-pGr-pUr-pGr-pUr-pCr-pCr-pAr-pUr-pGr-pAr-pCr-pC | Cyclic phosphorylation | 18 | 2.34 | 100 | 1.1 | 24.46 | 1916.581 | -3 | 5750.7622 | 5753.29 | 5750.7487 | 1449239.12 |
| 2235-2252 | Cr-pCr-pUr-pGr-pUr-pGr-pUr-pCr-pCr-pAr-pUr-pGr-pAr-pCr-pC | Cyclic phosphorylation | 18 | 2.43 | 100 | 1 | 24.46 | 718.088 | -8 | 5750.7627 | 5753.29 | 5750.7487 | 2523930.25 |
| 2235-2252 | Cr-pCr-pUr-pGr-pUr-pGr-pUr-pCr-pCr-pAr-pUr-pGr-pAr-pCr-pC | Cyclic phosphorylation | 18 | 2.34 | 100 | 1 | 24.47 | 820.816 | -7 | 5750.7622 | 5753.22 | 5750.7487 | 1929464.25 |
| 1986-2003 | Ar-pCr-pCr-pAr-pGr-pAr-pGr-pCr-pCr-pGr-pGr-pCr-pUr-pGr-pU | Cyclic phosphorylation | 18 | 2.83 | 100 | 1.3 | 24.47 | 1934.921 | -3 | 5805.7822 | 5808.21 | 5805.7658 | 755210.5 |
| 2235-2252 | Cr-pCr-pUr-pGr-pUr-pGr-pUr-pCr-pCr-pAr-pUr-pGr-pAr-pCr-pC | Cyclic phosphorylation | 18 | 2.43 | 100 | 1 | 24.47 | 957.787 | -6 | 5750.7627 | 5753.3 | 5750.7487 | 926367.12 |
| 2235-2252 | Cr-pCr-pUr-pGr-pUr-pGr-pUr-pCr-pCr-pAr-pUr-pGr-pAr-pCr-pC | Cyclic phosphorylation | 18 | 2.68 | 100 | 1.5 | 24.47 | 638.189 | -9 | 5750.7642 | 5753.16 | 5750.7487 | 1387313.12 |
| 1986-2003 | Ar-pCr-pCr-pAr-pGr-pAr-pGr-pCr-pCr-pGr-pGr-pCr-pUr-pGr-pU | Cyclic phosphorylation | 18 | 2.24 | 100 | 1.5 | 24.47 | 644.191 | -9 | 5805.7788 | 5808.26 | 5805.7658 | 691047.69 |
| 1986-2003 | Ar-pCr-pCr-pAr-pGr-pAr-pGr-pCr-pCr-pGr-pGr-pCr-pUr-pGr-pU | Cyclic phosphorylation | 18 | 2.58 | 100 | 1 | 24.49 | 724.965 | -8 | 5805.7808 | 5808.09 | 5805.7658 | 2042061.88 |
| 1986-2003 | Ar-pCr-pCr-pAr-pGr-pAr-pGr-pCr-pCr-pGr-pGr-pCr-pUr-pGr-pU | Cyclic phosphorylation | 18 | 2.33 | 100 | 1 | 24.51 | 828.676 | -7 | 5805.7793 | 5808.24 | 5805.7658 | 1174702 |
| 1671-1686 | Ar-pAr-pCr-pUr-pUr-pCr-pAr-pAr-pCr-pUr-pUr-pCr-pAr-pAr-pC | Cyclic phosphorylation | 16 | 1.49 | 100 | 1 | 24.7 | 1013.129 | -5 | 5068.6777 | 5070.83 | 5068.6702 | 2212074.75 |
| 3406-3423 | Ar-pCr-pAr-pAr-pCr-pAr-pCr-pCr-pUr-pUr-pCr-pGr-pUr-pGr-pU | Cyclic phosphorylation | 18 | 2.21 | 100 | 1 | 24.7 | 815.241 | -7 | 5711.7393 | 5714.11 | 5711.7266 | 1163803.5 |
| 1671-1686 | Ar-pAr-pCr-pUr-pUr-pCr-pAr-pAr-pCr-pUr-pUr-pCr-pAr-pAr-pC | Cyclic phosphorylation | 16 | 1.2 | 100 | 1 | 24.72 | 844.105 | -6 | 5068.6763 | 5070.84 | 5068.6702 | 6650448.5 |
| 1671-1686 | Ar-pAr-pCr-pUr-pUr-pCr-pAr-pAr-pCr-pUr-pUr-pCr-pAr-pAr-pC | Cyclic phosphorylation | 16 | 1.58 | 100 | 1 | 24.72 | 1266.413 | -4 | 5068.6782 | 5070.84 | 5068.6702 | 1451330 |
| 1671-1686 | Ar-pAr-pCr-pUr-pUr-pCr-pAr-pAr-pCr-pUr-pUr-pCr-pAr-pAr-pC | Cyclic phosphorylation | 16 | 1.68 | 100 | 1 | 24.72 | 1689.22 | -3 | 5068.6787 | 5070.81 | 5068.6702 | 5741614.5 |
| 3406-3423 | Ar-pCr-pAr-pAr-pCr-pAr-pCr-pCr-pUr-pUr-pCr-pGr-pUr-pGr-pU | Cyclic phosphorylation | 18 | 1.96 | 100 | 1.5 | 24.73 | 633.853 | -9 | 5711.7378 | 5714.06 | 5711.7266 | 616781.06 |
| 1671-1686 | Ar-pAr-pCr-pUr-pUr-pCr-pAr-pAr-pCr-pUr-pUr-pCr-pAr-pAr-pC | Cyclic phosphorylation | 16 | 1.2 | 100 | 1 | 24.73 | 723.375 | -7 | 5068.6763 | 5070.82 | 5068.6702 | 8188903.5 |
| 1671-1686 | Ar-pAr-pCr-pUr-pUr-pCr-pAr-pAr-pCr-pUr-pUr-pCr-pAr-pAr-pC | Cyclic phosphorylation | 16 | 1.97 | 100 | 1.6 | 24.73 | 632.828 | -8 | 5068.6802 | 5070.85 | 5068.6702 | 1104649.38 |
| 672-690 | Cr-pAr-pCr-pAr-pCr-pCr-pCr-pCr-pUr-pAr-pUr-pCr-pAr-pAr-pCr | Cyclic phosphorylation | 19 | 1.64 | 100 | 1.1 | 25.05 | 992.461 | -6 | 5958.8086 | 5961.37 | 5958.7988 | 945278.94 |
| 672-690 | Cr-pAr-pCr-pAr-pCr-pCr-pCr-pCr-pUr-pAr-pUr-pCr-pAr-pAr-pCr | Cyclic phosphorylation | 19 | 1.64 | 100 | 1 | 25.05 | 850.537 | -7 | 5958.8086 | 5961.4 | 5958.7988 | 2287429 |
| 672-690 | Cr-pAr-pCr-pAr-pCr-pCr-pCr-pCr-pUr-pAr-pUr-pCr-pAr-pAr-pCr | Cyclic phosphorylation | 19 | 1.4 | 100 | 1.1 | 25.05 | 1489.196 | -4 | 5958.8071 | 5961.36 | 5958.7988 | 523777.78 |

| | | | | | | | | | | | | | |
|---|---|---|---|---|---|---|---|---|---|---|---|---|---|
| 672-690 | Cr-pAr-pAr-pCr-pCr-pCr-pCr-pUr-pAr-pUr-pCr-pAr-pAr-pCr· | Cyclic phosphorylation | 19 | 1.97 | 100 | 1.2 | 25.05 | 1985.931 | -3 | 5958.8105 | 5961.41 | 5958.7988 | 1172120 |
| 672-690 | Cr-pAr-pAr-pCr-pCr-pCr-pCr-pUr-pAr-pUr-pCr-pAr-pAr-pCr· | Cyclic phosphorylation | 19 | 1.56 | 100 | 1 | 25.06 | 1191.155 | -5 | 5958.8081 | 5961.32 | 5958.7988 | 503209.94 |
| 672-690 | Cr-pAr-pAr-pCr-pCr-pCr-pCr-pUr-pAr-pUr-pCr-pAr-pAr-pCr· | Cyclic phosphorylation | 19 | 1.81 | 100 | 1 | 25.06 | 744.094 | -8 | 5958.8096 | 5961.4 | 5958.7988 | 2250277.5 |
| 672-690 | Cr-pAr-pAr-pCr-pCr-pCr-pCr-pUr-pAr-pUr-pCr-pAr-pAr-pCr· | Cyclic phosphorylation | 19 | -4.26 | 100 | 1.1 | 25.1 | 661.417 | -9 | 5958.7734 | 5961.34 | 5958.7988 | 530356.25 |
| 2728-2748 | Cr-pCr-pGr-pCr-pUr-pCr-pUr-pGr-pCr-pAr-pGr-pAr-pUr-pCr-pCr | Cyclic phosphorylation | 21 | 2.09 | 100 | 1 | 25.13 | 826.722 | -8 | 6619.832 | 6622.61 | 6619.8182 | 1314278 |
| 2728-2748 | Cr-pCr-pGr-pCr-pUr-pCr-pUr-pGr-pCr-pAr-pGr-pAr-pUr-pCr-pCr | Cyclic phosphorylation | 21 | 1.73 | 100 | 1 | 25.13 | 734.863 | -9 | 6619.8296 | 6622.69 | 6619.8182 | 1704538.12 |
| 2728-2748 | Cr-pCr-pGr-pCr-pUr-pCr-pUr-pGr-pCr-pAr-pGr-pAr-pUr-pCr-pCr | Cyclic phosphorylation | 21 | 0.99 | 100 | 1.1 | 25.17 | 661.277 | -10 | 6619.8247 | 6622.99 | 6619.8182 | 548525 |
| 2041-2059 | Ar-pCr-pAr-pUr-pCr-pCr-pCr-pCr-pAr-pUr-pCr-pGr-pGr-pCr-pGr | Cyclic phosphorylation | 19 | 1.99 | 100 | 1 | 25.18 | 673.75 | -9 | 6070.813 | 6073.45 | 6070.8009 | 1781348.38 |
| 2041-2059 | Ar-pCr-pAr-pUr-pCr-pCr-pCr-pCr-pAr-pUr-pCr-pGr-pGr-pCr-pGr | Cyclic phosphorylation | 19 | 1.66 | 100 | 1.5 | 25.2 | 2023.265 | -3 | 6070.811 | 6073.44 | 6070.8009 | 946351.25 |
| 2041-2059 | Ar-pCr-pAr-pUr-pCr-pCr-pCr-pCr-pAr-pUr-pCr-pGr-pGr-pCr-pGr | Cyclic phosphorylation | 19 | 1.5 | 100 | 1 | 25.2 | 758.094 | -8 | 6070.8101 | 6073.49 | 6070.8009 | 3378273.75 |
| 2041-2059 | Ar-pCr-pAr-pUr-pCr-pCr-pCr-pCr-pAr-pUr-pCr-pGr-pGr-pCr-pGr | Cyclic phosphorylation | 19 | 1.42 | 100 | 1 | 25.2 | 866.538 | -7 | 6070.8096 | 6073.46 | 6070.8009 | 1783014.62 |
| 1638-1653 | Ar-pAr-pAr-pAr-pGr-pCr-pAr-pCr-pCr-pAr-pAr-pUr-pCr-pUr-pCr | Cyclic phosphorylation | 16 | 2.03 | 100 | 1.1 | 25.31 | 640.584 | -8 | 5130.73 | 5132.86 | 5130.7195 | 1399390.25 |
| 1638-1653 | Ar-pAr-pAr-pAr-pGr-pCr-pAr-pCr-pCr-pAr-pAr-pUr-pCr-pUr-pCr | Cyclic phosphorylation | 16 | 1.56 | 100 | 1 | 25.31 | 732.24 | -7 | 5130.7275 | 5132.95 | 5130.7195 | 1928771.75 |
| 1638-1653 | Ar-pAr-pAr-pAr-pGr-pCr-pAr-pCr-pCr-pAr-pAr-pUr-pCr-pUr-pCr | Cyclic phosphorylation | 16 | 1.75 | 100 | 1 | 25.31 | 1709.904 | -3 | 5130.7285 | 5132.9 | 5130.7195 | 1985359.12 |
| 1638-1653 | Ar-pAr-pAr-pAr-pGr-pCr-pAr-pCr-pCr-pAr-pAr-pUr-pCr-pUr-pCr | Cyclic phosphorylation | 16 | 1.65 | 100 | 1 | 25.31 | 1025.54 | -5 | 5130.728 | 5132.84 | 5130.7195 | 596145.25 |
| 1638-1653 | Ar-pAr-pAr-pAr-pGr-pCr-pAr-pCr-pCr-pAr-pAr-pUr-pCr-pUr-pCr | Cyclic phosphorylation | 16 | 1.18 | 100 | 1 | 25.31 | 1282.176 | -4 | 5130.7256 | 5132.92 | 5130.7195 | 511698.53 |
| 1638-1653 | Ar-pAr-pAr-pAr-pGr-pCr-pAr-pCr-pCr-pAr-pAr-pUr-pCr-pUr-pCr | Cyclic phosphorylation | 16 | 1.18 | 100 | 1 | 25.31 | 854.281 | -6 | 5130.7256 | 5132.9 | 5130.7195 | 1480674.38 |
| 78-97 | Cr-pCr-pUr-pCr-pUr-pGr-pGr-pUr-pGr-pUr-pCr-pCr-pAr-pGr-pC | Cyclic phosphorylation | 20 | 1.38 | 100 | 1 | 25.46 | 798.47 | -8 | 6393.814 | 6396.58 | 6393.8052 | 1057456.5 |
| 78-97 | Cr-pCr-pUr-pCr-pUr-pGr-pGr-pUr-pGr-pUr-pCr-pCr-pAr-pGr-pC | Cyclic phosphorylation | 20 | 1.68 | 100 | 1.4 | 25.48 | 709.751 | -9 | 6393.8159 | 6396.71 | 6393.8052 | 734592.31 |
| 1725-1742 | Ar-pAr-pGr-pUr-pUr-pCr-pCr-pUr-pGr-pCr-pCr-pAr-pUr-pUr-pC | Cyclic phosphorylation | 18 | 1.53 | 100 | 1 | 25.5 | 713.21 | -8 | 5711.7354 | 5714.25 | 5711.7266 | 2770351.25 |
| 2447-2465 | Cr-pGr-pGr-pCr-pUr-pUr-pCr-pAr-pAr-pUr-pUr-pUr-pCr-pAr-pG | Cyclic phosphorylation | 19 | 1.43 | 100 | 1 | 25.5 | 756.341 | -8 | 6056.7827 | 6059.4 | 6056.774 | 1776720.88 |
| 1725-1742 | Ar-pAr-pGr-pUr-pUr-pCr-pCr-pUr-pGr-pCr-pCr-pAr-pUr-pUr-pC | Cyclic phosphorylation | 18 | 1.62 | 100 | 1 | 25.51 | 815.241 | -7 | 5711.7358 | 5714.23 | 5711.7266 | 1589018.75 |
| 2447-2465 | Cr-pGr-pGr-pCr-pUr-pUr-pCr-pAr-pAr-pUr-pUr-pUr-pCr-pAr-pG | Cyclic phosphorylation | 19 | 1.83 | 100 | 1 | 25.51 | 1008.958 | -6 | 6056.7852 | 6059.54 | 6056.774 | 669294 |
| 2447-2465 | Cr-pGr-pGr-pCr-pUr-pUr-pCr-pAr-pAr-pUr-pUr-pUr-pCr-pAr-pG | Cyclic phosphorylation | 19 | 1.59 | 100 | 1 | 25.51 | 864.534 | -7 | 6056.7837 | 6059.4 | 6056.774 | 1582550.12 |
| 1725-1742 | Ar-pAr-pGr-pUr-pUr-pCr-pCr-pUr-pGr-pCr-pCr-pAr-pUr-pUr-pC | Cyclic phosphorylation | 18 | 1.27 | 100 | 1.2 | 25.53 | 951.283 | -6 | 5711.7339 | 5714.14 | 5711.7266 | 621768.69 |
| 1725-1742 | Ar-pAr-pGr-pUr-pUr-pCr-pCr-pUr-pGr-pCr-pCr-pAr-pUr-pUr-pC | Cyclic phosphorylation | 18 | 1.79 | 100 | 1.3 | 25.53 | 1903.573 | -3 | 5711.7368 | 5714.28 | 5711.7266 | 960318.56 |
| 1725-1742 | Ar-pAr-pGr-pUr-pUr-pCr-pCr-pUr-pGr-pCr-pCr-pAr-pUr-pUr-pC | Cyclic phosphorylation | 18 | 1.96 | 100 | 1.2 | 25.55 | 633.964 | -9 | 5711.7378 | 5714.38 | 5711.7266 | 917721.5 |
| 1014-1032 | Cr-pAr-pGr-pCr-pCr-pCr-pAr-pCr-pCr-pGr-pAr-pAr-pUr-pCr-pCr | Cyclic phosphorylation | 19 | 0.9 | 100 | 1 | 25.6 | 753.973 | -8 | 6037.8325 | 6040.59 | 6037.8271 | 626561.75 |
| 2093-2110 | Cr-pCr-pCr-pUr-pCr-pGr-pGr-pAr-pGr-pAr-pGr-pCr-pCr-pAr-pGr | Cyclic phosphorylation | 18 | 1.68 | 100 | 1 | 25.62 | 835.398 | -7 | 5851.8301 | 5854.44 | 5851.8202 | 633918.44 |
| 2093-2110 | Cr-pCr-pCr-pUr-pCr-pGr-pGr-pAr-pGr-pAr-pGr-pCr-pCr-pAr-pGr | Cyclic phosphorylation | 18 | 1.52 | 100 | 1.1 | 25.62 | 730.722 | -8 | 5851.8291 | 5854.4 | 5851.8202 | 626415.25 |
| 1823-1842 | Cr-pAr-pGr-pCr-pUr-pUr-pCr-pGr-pGr-pCr-pGr-pGr-pAr-pGr-pU | Cyclic phosphorylation | 20 | 2.1 | 100 | 1.1 | 25.67 | 718.641 | -9 | 6473.8311 | 6476.64 | 6473.8175 | 653085.75 |
| 1823-1842 | Cr-pAr-pGr-pCr-pUr-pUr-pCr-pGr-pGr-pCr-pGr-pGr-pAr-pGr-pU | Cyclic phosphorylation | 20 | 2.1 | 100 | 1 | 25.72 | 808.597 | -8 | 6473.8311 | 6476.61 | 6473.8175 | 547456.12 |
| 1669-1686 | Ur-pGr-pAr-pAr-pCr-pUr-pUr-pCr-pAr-pAr-pCr-pUr-pUr-pCr-pA | Cyclic phosphorylation | 18 | 1.58 | 100 | 1.2 | 25.76 | 952.452 | -6 | 5719.752 | 5722.15 | 5719.7429 | 281391.28 |
| 1669-1686 | Ur-pGr-pAr-pAr-pCr-pUr-pUr-pCr-pAr-pAr-pCr-pUr-pUr-pCr-pA | Cyclic phosphorylation | 18 | 1.83 | 100 | 1 | 25.76 | 816.386 | -7 | 5719.7534 | 5722.21 | 5719.7429 | 594078.31 |
| 1669-1686 | Ur-pGr-pAr-pAr-pCr-pUr-pUr-pCr-pAr-pAr-pCr-pUr-pUr-pCr-pA | Cyclic phosphorylation | 18 | 1.58 | 100 | 1.2 | 25.77 | 714.213 | -8 | 5719.752 | 5721.97 | 5719.7429 | 391566.12 |
| 1743-1761 | Cr-pAr-pGr-pUr-pUr-pUr-pGr-pGr-pCr-pCr-pGr-pGr-pGr-pAr-pL | Cyclic phosphorylation | 19 | 2.46 | 100 | 1.1 | 25.83 | 682.859 | -9 | 6152.7964 | 6155.33 | 6152.7813 | 528590.94 |
| 1743-1761 | Cr-pAr-pGr-pUr-pUr-pUr-pGr-pGr-pCr-pCr-pGr-pGr-pGr-pAr-pL | Cyclic phosphorylation | 19 | 2.46 | 100 | 1.5 | 25.88 | 1024.792 | -6 | 6152.7964 | 6155.39 | 6152.7813 | 386210.44 |
| 3387-3405 | Cr-pCr-pCr-pCr-pAr-pGr-pAr-pUr-pCr-pAr-pUr-pCr-pAr-pCr-pCr· | Cyclic phosphorylation | 19 | 1.61 | 100 | 1 | 25.88 | 856.112 | -7 | 5997.8306 | 6000.45 | 5997.8209 | 1088580.88 |
| 1743-1761 | Cr-pAr-pGr-pUr-pUr-pUr-pGr-pGr-pCr-pCr-pGr-pGr-pGr-pAr-pL | Cyclic phosphorylation | 19 | 1.51 | 100 | 1 | 25.88 | 768.342 | -8 | 6152.7905 | 6155.44 | 6152.7813 | 1794536.12 |
| 1743-1761 | Cr-pAr-pGr-pUr-pUr-pUr-pGr-pGr-pCr-pCr-pGr-pGr-pGr-pAr-pL | Cyclic phosphorylation | 19 | 1.43 | 100 | 1 | 25.88 | 878.392 | -7 | 6152.79 | 6155.47 | 6152.7813 | 1037148 |
| 3387-3405 | Cr-pCr-pCr-pCr-pAr-pGr-pAr-pUr-pCr-pAr-pUr-pCr-pAr-pCr-pCr· | Cyclic phosphorylation | 19 | 1.44 | 100 | 1.6 | 25.88 | 1998.938 | -3 | 5997.8296 | 6000.48 | 5997.8209 | 626889.75 |
| 3387-3405 | Cr-pCr-pCr-pCr-pAr-pGr-pAr-pUr-pCr-pAr-pUr-pCr-pAr-pCr-pCr· | Cyclic phosphorylation | 19 | 1.36 | 100 | 1.2 | 25.9 | 998.965 | -6 | 5997.8291 | 6000.28 | 5997.8209 | 442724.91 |
| 3387-3405 | Cr-pCr-pCr-pCr-pAr-pGr-pAr-pUr-pCr-pAr-pUr-pCr-pAr-pCr-pCr· | Cyclic phosphorylation | 19 | 1.61 | 100 | 1 | 25.9 | 748.972 | -8 | 5997.8306 | 6000.43 | 5997.8209 | 1376844.12 |
| 1743-1761 | Cr-pAr-pGr-pUr-pUr-pUr-pGr-pGr-pCr-pCr-pGr-pGr-pGr-pAr-pL | Cyclic phosphorylation | 19 | 1.9 | 97.9 | 1.2 | 25.93 | 682.859 | -9 | 6152.793 | 6155.49 | 6152.7813 | 449171.44 |
| 996-1013 | Ar-pCr-pCr-pAr-pGr-pCr-pAr-pCr-pUr-pUr-pCr-pCr-pGr-pGr-pGr | Cyclic phosphorylation | 18 | 1.29 | 99.9 | 1.8 | 25.93 | 722.966 | -8 | 5789.7783 | 5792.24 | 5789.7709 | 491827.38 |
| 3846-3863 | Cr-pUr-pGr-pAr-pAr-pGr-pGr-pCr-pGr-pUr-pGr-pCr-pAr-pAr-pA | Cyclic phosphorylation | 18 | 2.08 | 100 | 1.2 | 25.98 | 735.969 | -8 | 5893.8066 | 5896.46 | 5893.7944 | 477683.47 |
| 3453-3468 | Ar-pAr-pCr-pAr-pAr-pUr-pAr-pCr-pCr-pGr-pUr-pGr-pUr-pAr-pCr | Cyclic phosphorylation | 16 | 2.04 | 99.9 | 1 | 26.11 | 734.666 | -7 | 5147.709 | 5149.99 | 5147.6985 | 301085.94 |
| 832-852 | Cr-pUr-pGr-pGr-pUr-pGr-pCr-pCr-pGr-pCr-pGr-pCr-pUr-pU | Cyclic phosphorylation | 21 | 1.98 | 99.6 | 1.2 | 26.16 | 956.4 | -7 | 6699.8435 | 6702.68 | 6699.8305 | 379460.91 |
| 832-852 | Cr-pUr-pGr-pGr-pUr-pGr-pCr-pCr-pGr-pCr-pGr-pCr-pUr-pU | Cyclic phosphorylation | 21 | 1.4 | 99.6 | 1 | 26.18 | 836.723 | -8 | 6699.8398 | 6702.72 | 6699.8305 | 783544.12 |
| 832-852 | Cr-pUr-pGr-pGr-pUr-pGr-pCr-pCr-pGr-pCr-pGr-pCr-pUr-pU | Cyclic phosphorylation | 21 | 1.76 | 99.6 | 1 | 26.18 | 743.754 | -9 | 6699.8423 | 6702.63 | 6699.8305 | 1012390.31 |
| 832-852 | Cr-pUr-pGr-pGr-pUr-pGr-pCr-pCr-pGr-pCr-pGr-pCr-pUr-pU | Cyclic phosphorylation | 21 | 2.86 | 99.6 | 1.5 | 26.21 | 669.278 | -10 | 6699.8496 | 6702.87 | 6699.8305 | 601375.62 |
| 522-540 | Ur-pUr-pCr-pGr-pGr-pGr-pUr-pGr-pUr-pAr-pCr-pAr-pGr-pC | Cyclic phosphorylation | 19 | 1.13 | 100 | 1 | 26.23 | 768.219 | -8 | 6151.8042 | 6154.6 | 6151.7972 | 579937.88 |
| 522-540 | Ur-pUr-pCr-pGr-pGr-pGr-pUr-pGr-pUr-pAr-pCr-pAr-pGr-pC | Cyclic phosphorylation | 19 | 1.85 | 100 | 1.1 | 26.25 | 878.109 | -7 | 6151.8086 | 6154.62 | 6151.7972 | 358075.19 |
| 561-581 | Ur-pAr-pCr-pGr-pUr-pGr-pUr-pCr-pCr-pAr-pGr-pCr-pCr-pUr | Cyclic phosphorylation | 21 | 1.93 | 98.2 | 1 | 26.35 | 826.845 | -8 | 6620.8149 | 6623.88 | 6620.8022 | 371260.09 |
| 561-581 | Ur-pAr-pCr-pGr-pUr-pGr-pUr-pCr-pCr-pAr-pGr-pCr-pCr-pUr | Cyclic phosphorylation | 21 | 2 | 98.2 | 1 | 26.35 | 734.862 | -9 | 6620.8154 | 6623.58 | 6620.8022 | 571281.25 |
| 1953-1972 | Cr-pGr-pGr-pUr-pGr-pUr-pAr-pCr-pUr-pCr-pCr-pAr-pCr-pCr | Cyclic phosphorylation | 20 | 2.04 | 98.4 | 1.7 | 26.37 | 921.546 | -7 | 6455.8677 | 6458.63 | 6455.8545 | 306949.91 |
| 1953-1972 | Cr-pGr-pGr-pUr-pGr-pUr-pAr-pCr-pUr-pCr-pCr-pAr-pCr-pCr | Cyclic phosphorylation | 20 | 1.81 | 98.4 | 1 | 26.37 | 806.352 | -8 | 6455.8662 | 6458.71 | 6455.8545 | 459047.56 |
| 1953-1972 | Cr-pGr-pGr-pUr-pGr-pUr-pAr-pCr-pUr-pCr-pCr-pAr-pCr-pCr | Cyclic phosphorylation | 20 | 1.89 | 98.4 | 1.5 | 26.37 | 716.644 | -9 | 6455.8667 | 6459.13 | 6455.8545 | 250042.03 |
| 2840-2858 | Cr-pGr-pCr-pCr-pAr-pUr-pCr-pGr-pGr-pCr-pAr-pAr-pGr-pAr-pUr | Cyclic phosphorylation | 19 | 1.59 | 100 | 1 | 26.42 | 763.975 | -8 | 6117.8491 | 6120.52 | 6117.8394 | 695487.81 |
| 2840-2858 | Cr-pGr-pCr-pCr-pAr-pUr-pCr-pGr-pGr-pCr-pAr-pAr-pGr-pAr-pUr | Cyclic phosphorylation | 19 | 1.99 | 100 | 1.1 | 26.44 | 873.258 | -7 | 6117.8516 | 6120.45 | 6117.8394 | 680574.19 |
| 1219-1236 | Ar-pCr-pCr-pUr-pGr-pUr-pGr-pCr-pUr-pUr-pCr-pAr-pCr-pAr-pAr | Cyclic phosphorylation | 18 | 2.48 | 100 | 1.1 | 26.44 | 818.531 | -7 | 5734.7681 | 5737.11 | 5734.7538 | 390286.06 |
| 1219-1236 | Ar-pCr-pCr-pUr-pGr-pUr-pGr-pCr-pUr-pUr-pCr-pAr-pCr-pAr-pAr | Cyclic phosphorylation | 18 | 1.89 | 100 | 1 | 26.46 | 716.089 | -8 | 5734.7646 | 5737.22 | 5734.7538 | 448398.44 |
| 956-975 | Cr-pAr-pCr-pCr-pCr-pUr-pGr-pAr-pAr-pGr-pUr-pCr-pCr-pUr-pUr | Cyclic phosphorylation | 20 | 2.4 | 100 | 1.2 | 26.48 | 701.531 | -9 | 6320.8403 | 6323.59 | 6320.8252 | 681911.06 |
| 956-975 | Cr-pAr-pCr-pCr-pCr-pUr-pGr-pAr-pAr-pGr-pUr-pCr-pCr-pUr-pUr | Cyclic phosphorylation | 20 | 2.09 | 100 | 1.1 | 26.49 | 902.256 | -7 | 6320.8384 | 6323.57 | 6320.8252 | 640883.19 |
| 956-975 | Cr-pAr-pCr-pCr-pCr-pUr-pGr-pAr-pAr-pGr-pUr-pCr-pCr-pUr-pUr | Cyclic phosphorylation | 20 | 1.94 | 100 | 1.1 | 26.51 | 789.473 | -8 | 6320.8374 | 6323.55 | 6320.8252 | 973176.38 |
| 3016-3033 | Ar-pGr-pGr-pCr-pCr-pCr-pGr-pAr-pGr-pGr-pUr-pGr-pCr-pAr-pGr-pA | Cyclic phosphorylation | 18 | 2.04 | 90.7 | 1 | 26.51 | 737.845 | -8 | 5908.8174 | 5911.36 | 5908.8053 | 282015.88 |
| 3016-3033 | Ar-pGr-pGr-pCr-pCr-pCr-pGr-pAr-pGr-pGr-pUr-pGr-pCr-pAr-pGr-pA | Cyclic phosphorylation | 18 | 2.04 | 90.7 | 1 | 26.53 | 843.539 | -7 | 5908.8174 | 5911.42 | 5908.8053 | 341311.59 |
| 2004-2021 | Ar-pUr-pCr-pGr-pGr-pAr-pGr-pCr-pCr-pGr-pAr-pGr-pCr-pAr-pCr | Cyclic phosphorylation | 18 | 1.94 | 100 | 2 | 26.7 | 651.417 | -9 | 5868.8105 | 5871.43 | 5868.7992 | 448978.09 |
| 2004-2021 | Ar-pUr-pCr-pGr-pGr-pAr-pGr-pCr-pCr-pGr-pAr-pGr-pCr-pAr-pCr | Cyclic phosphorylation | 18 | 1.86 | 100 | 1.1 | 26.74 | 732.844 | -8 | 5868.8101 | 5871.36 | 5868.7992 | 1007978.25 |
| 2004-2021 | Ar-pUr-pCr-pGr-pGr-pAr-pGr-pCr-pCr-pGr-pAr-pGr-pCr-pAr-pCr | Cyclic phosphorylation | 18 | 2.02 | 100 | 1 | 26.74 | 837.681 | -7 | 5868.811 | 5871.39 | 5868.7992 | 600434.5 |
| 2997-3015 | Ar-pGr-pCr-pAr-pGr-pAr-pCr-pUr-pGr-pAr-pCr-pCr-pCr-pUr | Cyclic phosphorylation | 19 | 2.54 | 98 | 1.2 | 26.75 | 761.096 | -8 | 6094.8276 | 6097.65 | 6094.8122 | 356444.59 |
| 2997-3015 | Ar-pGr-pCr-pAr-pGr-pAr-pCr-pUr-pGr-pAr-pCr-pCr-pCr-pUr | Cyclic phosphorylation | 19 | 2.86 | 98 | 1 | 26.75 | 869.967 | -7 | 6094.8296 | 6097.66 | 6094.8122 | 406826.44 |
| 3406-3424 | Ar-pCr-pAr-pAr-pCr-pAr-pCr-pCr-pUr-pUr-pCr-pGr-pUr-pGr-pUr | Cyclic phosphorylation | 19 | 4.25 | 97 | 1.1 | 26.86 | 864.535 | -7 | 6056.7998 | 6059.49 | 6056.774 | 426973.53 |
| 3406-3424 | Ar-pCr-pAr-pAr-pCr-pAr-pCr-pCr-pUr-pUr-pCr-pGr-pUr-pGr-pUr | Cyclic phosphorylation | 19 | 4.82 | 97 | 1.1 | 26.9 | 756.218 | -8 | 6056.8032 | 6059.11 | 6056.774 | 710526.56 |
| 3406-3424 | Ar-pCr-pAr-pAr-pCr-pAr-pCr-pCr-pUr-pUr-pCr-pGr-pUr-pGr-pUr | Cyclic phosphorylation | 19 | 3.61 | 97 | 1.5 | 26.91 | 672.192 | -9 | 6056.7959 | 6059.35 | 6056.774 | 497439.41 |
| 3185-3203 | Cr-pAr-pAr-pGr-pGr-pGr-pCr-pUr-pAr-pCr-pCr-pAr-pCr-pCr-pUr | Cyclic phosphorylation | 19 | 1.49 | 98.2 | 1 | 26.93 | 764.223 | -8 | 6118.8325 | 6121.47 | 6118.8234 | 550100.56 |
| 3185-3203 | Cr-pAr-pAr-pGr-pGr-pGr-pCr-pUr-pAr-pCr-pCr-pAr-pCr-pCr-pUr | Cyclic phosphorylation | 19 | 2.37 | 98.2 | 1 | 26.93 | 873.398 | -7 | 6118.8379 | 6121.46 | 6118.8234 | 425681.91 |
| 3547-3564 | Ar-pCr-pCr-pUr-pGr-pGr-pCr-pGr-pAr-pUr-pAr-pUr-pCr-pA | Cyclic phosphorylation | 18 | 1.31 | 99.9 | 1 | 26.96 | 728.092 | -8 | 5829.7847 | 5832.25 | 5829.777 | 530609.81 |
| 3547-3564 | Ar-pCr-pCr-pUr-pGr-pGr-pCr-pGr-pAr-pUr-pAr-pUr-pCr-pA | Cyclic phosphorylation | 18 | 0.98 | 99.9 | 1 | 27 | 832.106 | -7 | 5829.7827 | 5832.35 | 5829.777 | 517359.78 |
| 3118-3137 | Cr-pCr-pAr-pAr-pUr-pCr-pUr-pGr-pGr-pCr-pCr-pGr-pCr-pAr | Cyclic phosphorylation | 20 | -9.33 | 97.5 | 1.1 | 27.05 | 797.229 | -8 | 6382.8149 | 6385.64 | 6382.8745 | 496712.97 |
| 1618-1637 | Cr-pCr-pAr-pCr-pAr-pGr-pUr-pGr-pUr-pGr-pCr-pGr-pGr-pCr-pCr | Cyclic phosphorylation | 20 | 1.94 | 100 | 1 | 27.07 | 804.227 | -8 | 6439.8721 | 6442.6 | 6439.8596 | 1473798.5 |
| 1618-1637 | Cr-pCr-pAr-pCr-pAr-pGr-pUr-pGr-pUr-pGr-pCr-pGr-pGr-pCr-pCr | Cyclic phosphorylation | 20 | 1.71 | 100 | 1 | 27.07 | 919.261 | -7 | 6439.8706 | 6442.67 | 6439.8596 | 782091.69 |
| 3118-3137 | Cr-pCr-pAr-pAr-pUr-pCr-pUr-pGr-pGr-pCr-pCr-pGr-pCr-pAr | Cyclic phosphorylation | 20 | 2.68 | 97.5 | 1.8 | 27.07 | 708.537 | -9 | 6382.8916 | 6385.66 | 6382.8745 | 519408.81 |
| 1618-1637 | Cr-pCr-pAr-pCr-pAr-pGr-pUr-pGr-pUr-pGr-pCr-pGr-pGr-pCr-pCr | Cyclic phosphorylation | 20 | 1.63 | 100 | 1.1 | 27.07 | 714.868 | -9 | 6439.8701 | 6442.67 | 6439.8596 | 666353.75 |
| 3375-3392 | Ar-pAr-pCr-pUr-pAr-pCr-pUr-pAr-pCr-pGr-pAr-pGr-pAr-pUr-pCr | Cyclic phosphorylation | 18 | 1.65 | 100 | 1.1 | 27.12 | 715.965 | -8 | 5733.7793 | 5736.3 | 5733.7698 | 598807.06 |
| 650-667 | Cr-pUr-pAr-pCr-pUr-pUr-pCr-pAr-pAr-pGr-pAr-pUr-pCr-pUr-pA | Cyclic phosphorylation | 18 | 0.98 | 100 | 1 | 27.14 | 714.212 | -8 | 5719.7485 | 5722.1 | 5719.7429 | 1303657 |
| 1671-1687 | Ar-pAr-pCr-pUr-pUr-pCr-pAr-pAr-pCr-pUr-pUr-pCr-pAr-pGr-pCr | Cyclic phosphorylation | 17 | -0.15 | 100 | 1.1 | 27.14 | 772.668 | -7 | 5413.7168 | 5415.95 | 5413.7176 | 585983.88 |
| 650-667 | Cr-pUr-pAr-pCr-pUr-pUr-pCr-pAr-pAr-pGr-pAr-pUr-pCr-pUr-pA | Cyclic phosphorylation | 18 | 1.41 | 100 | 1.6 | 27.14 | 1906.245 | -3 | 5719.751 | 5722.26 | 5719.7429 | 555928.69 |
| 1918-1937 | Ur-pGr-pGr-pCr-pCr-pAr-pUr-pCr-pAr-pUr-pCr-pGr-pCr-pGr | Cyclic phosphorylation | 20 | 0.45 | 100 | 1.1 | 27.14 | 710.42 | -9 | 6400.8403 | 6403.45 | 6400.8375 | 924019.69 |
| 3375-3392 | Ar-pAr-pCr-pUr-pAr-pCr-pUr-pAr-pCr-pGr-pAr-pGr-pAr-pUr-pCr | Cyclic phosphorylation | 18 | 0.8 | 100 | 1 | 27.16 | 818.39 | -7 | 5733.7744 | 5736.37 | 5733.7698 | 722973.56 |
| 650-667 | Cr-pUr-pAr-pCr-pUr-pUr-pCr-pAr-pAr-pGr-pAr-pUr-pCr-pUr-pA | Cyclic phosphorylation | 18 | 1.32 | 100 | 1 | 27.17 | 816.386 | -7 | 5719.7505 | 5722.25 | 5719.7429 | 845519.62 |
| 1918-1937 | Ur-pGr-pGr-pCr-pCr-pAr-pUr-pCr-pAr-pUr-pCr-pGr-pCr-pGr | Cyclic phosphorylation | 20 | 0.68 | 100 | 1 | 27.17 | 799.349 | -8 | 6400.8418 | 6403.6 | 6400.8375 | 1143288.5 |
| 1918-1937 | Ur-pGr-pGr-pCr-pCr-pAr-pUr-pCr-pAr-pUr-pCr-pGr-pCr-pGr | Cyclic phosphorylation | 20 | 2.05 | 100 | 1.1 | 27.18 | 913.829 | -7 | 6400.8506 | 6403.59 | 6400.8375 | 684849 |
| 844-863 | Cr-pUr-pUr-pAr-pCr-pUr-pAr-pUr-pGr-pUr-pGr-pGr-pCr-pL | Cyclic phosphorylation | 20 | 2 | 99.8 | 1 | 27.19 | 708.081 | -9 | 6379.791 | 6382.46 | 6379.7783 | 733580.31 |
| 2757-2775 | Ar-pUr-pGr-pGr-pCr-pUr-pAr-pCr-pCr-pGr-pGr-pUr-pCr-pCr | Cyclic phosphorylation | 19 | 2.76 | 99.7 | 1.3 | 27.26 | 761.219 | -8 | 6095.813 | 6098.36 | 6095.7962 | 422742.78 |
| 3206-3228 | Cr-pUr-pUr-pCr-pCr-pCr-pUr-pAr-pGr-pUr-pCr-pUr-pGr-pCr | Cyclic phosphorylation | 23 | 1.96 | 100 | 1.2 | 27.37 | 798.206 | -9 | 7189.9087 | 7193.27 | 7189.8946 | 383908.31 |
| 913-932 | Ar-pCr-pGr-pCr-pGr-pUr-pGr-pGr-pAr-pUr-pUr-pGr-pUr-pG | Cyclic phosphorylation | 20 | 1.99 | 99.8 | 1.2 | 27.4 | 714.194 | -9 | 6434.8081 | 6437.88 | 6434.7953 | 327515.59 |
| 3738-3755 | Ar-pUr-pGr-pGr-pUr-pCr-pAr-pCr-pAr-pAr-pUr-pCr-pAr-pUr-pG | Cyclic phosphorylation | 18 | 1.63 | 100 | 1.8 | 27.47 | 641.078 | -9 | 5775.7534 | 5778.47 | 5775.744 | 383812.5 |
| 3738-3755 | Ar-pUr-pGr-pGr-pUr-pCr-pAr-pCr-pAr-pAr-pUr-pCr-pAr-pUr-pG | Cyclic phosphorylation | 18 | 2.56 | 100 | 1 | 27.49 | 824.245 | -7 | 5775.7588 | 5778.05 | 5775.744 | 380497.69 |
| 3738-3755 | Ar-pUr-pGr-pGr-pUr-pCr-pAr-pCr-pAr-pAr-pUr-pCr-pAr-pUr-pG | Cyclic phosphorylation | 18 | 2.31 | 100 | 1.1 | 27.51 | 721.212 | -8 | 5775.7573 | 5778.25 | 5775.744 | 711069.5 |
| 316-334 | Ar-pCr-pGr-pGr-pGr-pGr-pUr-pGr-pUr-pAr-pCr-pUr-pUr-pUr-pC | Cyclic phosphorylation | 19 | 1.43 | 97.2 | 1.1 | 27.53 | 768.342 | -8 | 6152.79 | 6155.44 | 6152.7813 | 349414.53 |
| 2227-2246 | Ar-pGr-pAr-pUr-pCr-pCr-pUr-pGr-pCr-pCr-pUr-pGr-pUr-pGr-pU | Cyclic phosphorylation | 20 | 1.33 | 100 | 1 | 27.56 | 796.719 | -8 | 6378.8027 | 6381.62 | 6378.7943 | 509282.31 |
| 2227-2246 | Ar-pGr-pAr-pUr-pCr-pCr-pUr-pGr-pCr-pCr-pUr-pGr-pUr-pGr-pU | Cyclic phosphorylation | 20 | 1.33 | 100 | 1.1 | 27.58 | 910.537 | -7 | 6378.8027 | 6381.6 | 6378.7943 | 372174 |
| 1312-1328 | Ar-pCr-pUr-pAr-pCr-pAr-pAr-pCr-pUr-pAr-pCr-pAr-pAr-pGr-pCr | Cyclic phosphorylation | 17 | 1.67 | 100 | 1.3 | 27.59 | 678.712 | -8 | 5436.7539 | 5439 | 5436.7448 | 447903.25 |
| 1312-1328 | Ar-pCr-pUr-pAr-pCr-pAr-pAr-pCr-pUr-pAr-pCr-pAr-pAr-pGr-pCr | Cyclic phosphorylation | 17 | 1.31 | 100 | 1 | 27.59 | 775.958 | -7 | 5436.752 | 5439.03 | 5436.7448 | 680831.5 |
| 3249-3267 | Ar-pCr-pAr-pUr-pAr-pUr-pGr-pUr-pGr-pCr-pCr-pGr-pCr-pU | Cyclic phosphorylation | 19 | 1.85 | 100 | 1 | 27.66 | 759.22 | -8 | 6079.8125 | 6082.47 | 6079.8013 | 1346937.88 |
| 3249-3267 | Ar-pCr-pAr-pUr-pAr-pUr-pGr-pUr-pGr-pCr-pCr-pGr-pCr-pU | Cyclic phosphorylation | 19 | 2.01 | 100 | 1.4 | 27.67 | 674.75 | -9 | 6079.8135 | 6082.43 | 6079.8013 | 925243.56 |
| 3249-3267 | Ar-pCr-pAr-pUr-pAr-pUr-pGr-pUr-pGr-pCr-pCr-pGr-pCr-pU | Cyclic phosphorylation | 19 | 1.85 | 100 | 1 | 27.68 | 867.824 | -7 | 6079.8125 | 6082.47 | 6079.8013 | 792387.44 |
| 3249-3267 | Ar-pCr-pAr-pUr-pAr-pUr-pGr-pUr-pGr-pCr-pCr-pGr-pCr-pU | Cyclic phosphorylation | 19 | 2.17 | 100 | 1.3 | 27.68 | 1012.629 | -6 | 6079.8145 | 6082.53 | 6079.8013 | 320691.53 |
| 3249-3267 | Ar-pCr-pAr-pUr-pAr-pUr-pGr-pUr-pGr-pCr-pCr-pGr-pCr-pU | Cyclic phosphorylation | 19 | 2.65 | 100 | 1.5 | 27.68 | 2026.266 | -3 | 6079.8174 | 6082.44 | 6079.8013 | 444137.69 |
| 2814-2831 | Ar-pAr-pGr-pCr-pUr-pGr-pAr-pUr-pCr-pGr-pCr-pCr-pAr-pAr-pCr | Cyclic phosphorylation | 18 | 2.11 | 99.9 | 1 | 27.91 | 827.396 | -7 | 5796.8154 | 5799.35 | 5796.8032 | 470250.84 |
| 2472-2491 | Cr-pGr-pCr-pGr-pAr-pUr-pCr-pUr-pAr-pGr-pCr-pAr-pAr-pGr-pCr | Cyclic phosphorylation | 20 | 2.14 | 93 | 2 | 27.93 | 797.104 | -8 | 6382.8882 | 6385.62 | 6382.8745 | 380503.5 |
| 2814-2831 | Ar-pAr-pGr-pCr-pUr-pGr-pAr-pUr-pCr-pGr-pCr-pCr-pAr-pAr-pCr | Cyclic phosphorylation | 18 | 1.86 | 99.9 | 1.6 | 27.93 | 1931.933 | -3 | 5796.814 | 5799.38 | 5796.8032 | 379541.06 |
| 2814-2831 | Ar-pAr-pGr-pCr-pUr-pGr-pAr-pUr-pCr-pGr-pCr-pCr-pAr-pAr-pCr | Cyclic phosphorylation | 18 | 2.45 | 99.9 | 1.3 | 27.95 | 723.845 | -8 | 5796.8174 | 5799.25 | 5796.8032 | 903094.81 |
| 2814-2831 | Ar-pAr-pGr-pCr-pUr-pGr-pAr-pUr-pCr-pGr-pCr-pCr-pAr-pAr-pCr | Cyclic phosphorylation | 18 | 1.19 | 99.9 | 1.2 | 27.96 | 643.306 | -9 | 5796.8101 | 5799.28 | 5796.8032 | 429804.91 |
| 1559-1579 | Cr-pGr-pUr-pGr-pGr-pGr-pCr-pUr-pAr-pUr-pCr-pAr-pGr-pCr-pC | Cyclic phosphorylation | 21 | 3.09 | 95.4 | 1.2 | 27.96 | 748.76 | -9 | 6745.9058 | 6748.8 | 6745.8849 | 1153034.88 |
| 3273-3291 | Ar-pAr-pUr-pUr-pCr-pAr-pCr-pCr-pAr-pCr-pCr-pGr-pCr-pUr | Cyclic phosphorylation | 19 | 1.32 | 100 | 1 | 28.05 | 749.344 | -8 | 5999.7969 | 6002.4 | 5999.789 | 505023.25 |

| | | | | | | | | | | | | | |
|---|---|---|---|---|---|---|---|---|---|---|---|---|---|
| 3858-3874 | Ar-pAr-pAr-pCr-pUr-pGr-pCr-pAr-pCr-pUr-pAr-pCr-pAr-pCr-pAr | Cyclic phosphorylation | 17 | 1.67 | 100 | 1 | 28.12 | 775.815 | -7 | 5436.7539 | 5439.13 | 5436.7448 | 1266209.38 |
| 3858-3874 | Ar-pAr-pAr-pCr-pUr-pGr-pCr-pAr-pCr-pUr-pAr-pCr-pAr-pCr-pAr | Cyclic phosphorylation | 17 | 2.47 | 100 | 1.1 | 28.12 | 905.453 | -6 | 5436.7583 | 5439.03 | 5436.7448 | 582997.44 |
| 3985-4006 | Cr-pCr-pCr-pCr-pAr-pCr-pUr-pCr-pAr-pCr-pCr-pAr-pCr-pCr-pUr- | Cyclic phosphorylation | 22 | 2.02 | 100 | 1.3 | 28.12 | 764.872 | -9 | 6890.9155 | 6893.92 | 6890.9016 | 710778.19 |
| 3273-3291 | Ar-pAr-pUr-pUr-pUr-pCr-pAr-pCr-pCr-pAr-pCr-pCr-pGr-pCr-pU | Cyclic phosphorylation | 19 | 1.48 | 100 | 1 | 28.12 | 856.393 | -7 | 5999.7979 | 6002.44 | 5999.789 | 1269256.5 |
| 3858-3874 | Ar-pAr-pAr-pCr-pUr-pGr-pCr-pAr-pCr-pUr-pAr-pCr-pAr-pCr-pAr | Cyclic phosphorylation | 17 | 2.12 | 100 | 1.1 | 28.12 | 1811.913 | -3 | 5436.7563 | 5439.04 | 5436.7448 | 957360.94 |
| 979-995 | Ar-pAr-pAr-pAr-pGr-pGr-pGr-pCr-pAr-pUr-pCr-pUr-pAr-pCr-pC | Cyclic phosphorylation | 17 | 0.94 | 94 | 1.4 | 28.12 | 688.716 | -8 | 5515.7783 | 5518.3 | 5515.7731 | 450172.09 |
| 3985-4006 | Cr-pCr-pCr-pCr-pAr-pCr-pUr-pCr-pAr-pCr-pCr-pAr-pCr-pCr-pUr- | Cyclic phosphorylation | 22 | 0.96 | 100 | 1.8 | 28.13 | 860.607 | -8 | 6890.9082 | 6893.95 | 6890.9016 | 510146.78 |
| 3273-3291 | Ar-pAr-pUr-pUr-pUr-pCr-pAr-pCr-pCr-pAr-pCr-pCr-pGr-pCr-pU | Cyclic phosphorylation | 19 | 0.5 | 100 | 1 | 28.13 | 749.218 | -8 | 5999.792 | 6002.41 | 5999.789 | 2296673.5 |
| 3858-3874 | Ar-pAr-pAr-pCr-pUr-pGr-pCr-pAr-pCr-pUr-pAr-pCr-pAr-pCr-pAr | Cyclic phosphorylation | 17 | 1.76 | 100 | 1.1 | 28.13 | 678.837 | -8 | 5436.7544 | 5439.04 | 5436.7448 | 1452352.12 |
| 3273-3291 | Ar-pAr-pUr-pUr-pUr-pCr-pAr-pCr-pCr-pAr-pCr-pCr-pGr-pCr-pU | Cyclic phosphorylation | 19 | 1.97 | 100 | 1.3 | 28.14 | 1999.595 | -3 | 5999.8008 | 6002.43 | 5999.789 | 796968.12 |
| 3273-3291 | Ar-pAr-pUr-pUr-pUr-pCr-pAr-pCr-pCr-pAr-pCr-pCr-pGr-pCr-pU | Cyclic phosphorylation | 19 | 1.97 | 100 | 1 | 28.14 | 665.86 | -9 | 5999.8008 | 6002.44 | 5999.789 | 936230.94 |
| 2037-2058 | Ur-pGr-pCr-pGr-pAr-pCr-pAr-pUr-pCr-pCr-pCr-pCr-pAr-pUr-pCr | Cyclic phosphorylation | 22 | 2.1 | 100 | 1 | 28.38 | 877.609 | -8 | 7026.9297 | 7029.92 | 7026.915 | 692404.75 |
| 2037-2058 | Ur-pGr-pCr-pGr-pAr-pCr-pAr-pUr-pCr-pCr-pCr-pCr-pAr-pUr-pCr | Cyclic phosphorylation | 22 | 1.82 | 100 | 1 | 28.38 | 779.985 | -9 | 7026.9277 | 7029.97 | 7026.915 | 509916.06 |
| 2037-2058 | Ur-pGr-pCr-pGr-pAr-pCr-pAr-pUr-pCr-pCr-pCr-pCr-pAr-pUr-pCr | Cyclic phosphorylation | 22 | 1.89 | 100 | 1.3 | 28.39 | 1003.269 | -7 | 7026.9282 | 7029.93 | 7026.915 | 395948.91 |
| 948-965 | Ar-pCr-pAr-pAr-pAr-pGr-pUr-pGr-pCr-pAr-pCr-pCr-pCr-pUr-pG | Cyclic phosphorylation | 18 | 2.19 | 96.9 | 1.6 | 28.4 | 726.846 | -8 | 5820.8271 | 5823.49 | 5820.8144 | 386814.56 |
| 2968-2988 | Cr-pCr-pAr-pUr-pCr-pAr-pGr-pCr-pUr-pCr-pUr-pGr-pUr-pGr-pC | Cyclic phosphorylation | 21 | 3.57 | 100 | 1 | 28.42 | 837.603 | -8 | 6706.8867 | 6709.61 | 6706.8628 | 880872.25 |
| 1112-1129 | Gr-pAr-pAr-pCr-pCr-pGr-pGr-pAr-pAr-pGr-pCr-pGr-pGr-pAr-pU | Cyclic phosphorylation | 18 | 3.18 | 98.8 | 1.2 | 28.43 | 844.544 | -7 | 5915.8564 | 5918.5 | 5915.8376 | 268419.12 |
| 2968-2988 | Cr-pCr-pAr-pUr-pCr-pAr-pGr-pCr-pUr-pCr-pUr-pGr-pUr-pGr-pC | Cyclic phosphorylation | 21 | 3.57 | 100 | 1.1 | 28.45 | 744.536 | -9 | 6706.8867 | 6709.57 | 6706.8628 | 986885.81 |
| 1113-1129 | Ar-pAr-pCr-pCr-pGr-pGr-pAr-pAr-pGr-pCr-pGr-pGr-pAr-pUr-pC | Cyclic phosphorylation | 17 | 2.08 | 100 | 1.1 | 28.5 | 795.107 | -7 | 5570.8018 | 5573.17 | 5570.7902 | 372797.84 |
| 3484-3501 | Ar-pGr-pCr-pUr-pGr-pGr-pAr-pCr-pAr-pGr-pCr-pUr-pUr-pCr-pA | Cyclic phosphorylation | 18 | 1.44 | 100 | 1 | 28.57 | 972.294 | -6 | 5837.8018 | 5840.6 | 5837.7933 | 298704.31 |
| 3484-3501 | Ar-pGr-pCr-pUr-pGr-pGr-pAr-pCr-pAr-pGr-pCr-pUr-pUr-pCr-pA | Cyclic phosphorylation | 18 | 2.11 | 100 | 1 | 28.59 | 833.251 | -7 | 5837.8057 | 5840.39 | 5837.7933 | 603073.81 |
| 1952-1972 | Gr-pCr-pGr-pGr-pGr-pUr-pGr-pUr-pAr-pCr-pUr-pCr-pCr-pAr-pC | Cyclic phosphorylation | 21 | 2.5 | 100 | 1 | 28.66 | 970.839 | -7 | 6800.9189 | 6803.83 | 6800.9019 | 932048.38 |
| 1952-1972 | Gr-pCr-pGr-pGr-pGr-pUr-pGr-pUr-pAr-pCr-pUr-pCr-pCr-pAr-pC | Cyclic phosphorylation | 21 | 1.64 | 100 | 1.3 | 28.67 | 1132.813 | -6 | 6800.9131 | 6804.06 | 6800.9019 | 471659.47 |
| 1952-1972 | Gr-pCr-pGr-pGr-pGr-pUr-pGr-pUr-pAr-pCr-pUr-pCr-pCr-pAr-pC | Cyclic phosphorylation | 21 | 2.07 | 100 | 1 | 28.67 | 849.482 | -8 | 6800.916 | 6803.98 | 6800.9019 | 1456192.75 |
| 1952-1972 | Gr-pCr-pGr-pGr-pGr-pUr-pGr-pUr-pAr-pCr-pUr-pCr-pCr-pAr-pC | Cyclic phosphorylation | 21 | 1.78 | 100 | 1 | 28.68 | 754.873 | -9 | 6800.9141 | 6804.02 | 6800.9019 | 1304482.5 |
| 1952-1972 | Gr-pCr-pGr-pGr-pGr-pUr-pGr-pUr-pAr-pCr-pUr-pCr-pCr-pAr-pC | Cyclic phosphorylation | 21 | 1.71 | 100 | 1.9 | 28.68 | 1700.222 | -4 | 6800.9136 | 6804.3 | 6800.9019 | 343618.69 |
| 3268-3285 | Ar-pGr-pAr-pAr-pGr-pAr-pAr-pUr-pUr-pUr-pCr-pAr-pCr-pCr-pA | Cyclic phosphorylation | 18 | 2.15 | 99.9 | 1 | 28.73 | 825.251 | -7 | 5781.8047 | 5784.03 | 5781.7923 | 419131.78 |
| 2661-2679 | Ar-pUr-pCr-pGr-pCr-pCr-pCr-pAr-pGr-pUr-pAr-pCr-pAr-pCr-pAr | Cyclic phosphorylation | 19 | 2.07 | 100 | 1 | 28.76 | 754.219 | -8 | 6039.8076 | 6042.47 | 6039.7951 | 783486.5 |
| 2661-2679 | Ar-pUr-pCr-pGr-pCr-pCr-pCr-pAr-pGr-pUr-pAr-pCr-pAr-pCr-pAr | Cyclic phosphorylation | 19 | 2.23 | 100 | 1.4 | 28.76 | 670.305 | -9 | 6039.8086 | 6042.39 | 6039.7951 | 570752.31 |
| 1226-1245 | Cr-pUr-pUr-pCr-pAr-pCr-pAr-pAr-pAr-pCr-pGr-pUr-pGr-pUr-pA | Cyclic phosphorylation | 20 | 1.64 | 99.9 | 1.4 | 28.87 | 708.644 | -9 | 6384.853 | 6387.59 | 6384.8426 | 512676.88 |
| 1226-1245 | Cr-pUr-pUr-pCr-pAr-pCr-pAr-pAr-pAr-pCr-pGr-pUr-pGr-pUr-pA | Cyclic phosphorylation | 20 | 2.41 | 99.9 | 1 | 28.88 | 797.35 | -8 | 6384.8579 | 6387.57 | 6384.8426 | 577641.69 |
| 2060-2078 | Ar-pAr-pUr-pCr-pUr-pGr-pCr-pGr-pCr-pCr-pAr-pGr-pCr-pUr-pA | Cyclic phosphorylation | 19 | 2.19 | 100 | 1 | 28.94 | 1012.465 | -6 | 6078.8306 | 6081.52 | 6078.8172 | 616151.69 |
| 2060-2078 | Ar-pAr-pUr-pCr-pUr-pGr-pCr-pGr-pCr-pCr-pAr-pGr-pCr-pUr-pA | Cyclic phosphorylation | 19 | 1.31 | 100 | 1 | 28.94 | 674.641 | -9 | 6078.8252 | 6081.47 | 6078.8172 | 1263297.5 |
| 2060-2078 | Ar-pAr-pUr-pCr-pUr-pGr-pCr-pGr-pCr-pCr-pAr-pGr-pCr-pUr-pA | Cyclic phosphorylation | 19 | 2.11 | 100 | 1.3 | 28.94 | 2025.938 | -3 | 6078.8301 | 6081.52 | 6078.8172 | 867989.44 |
| 2060-2078 | Ar-pAr-pUr-pCr-pUr-pGr-pCr-pGr-pCr-pCr-pAr-pGr-pCr-pUr-pA | Cyclic phosphorylation | 19 | 1.23 | 100 | 1.1 | 28.94 | 1519.201 | -4 | 6078.8247 | 6081.54 | 6078.8172 | 516205.66 |
| 2060-2078 | Ar-pAr-pUr-pCr-pUr-pGr-pCr-pGr-pCr-pCr-pAr-pGr-pCr-pUr-pA | Cyclic phosphorylation | 19 | 2.03 | 100 | 1 | 28.94 | 867.683 | -7 | 6078.8296 | 6081.5 | 6078.8172 | 1555155.88 |
| 2060-2078 | Ar-pAr-pUr-pCr-pUr-pGr-pCr-pGr-pCr-pCr-pAr-pGr-pCr-pUr-pA | Cyclic phosphorylation | 19 | 1.71 | 100 | 1 | 28.94 | 759.096 | -8 | 6078.8276 | 6081.47 | 6078.8172 | 2980378.75 |
| 246-267 | Ur-pUr-pCr-pCr-pAr-pCr-pGr-pCr-pCr-pAr-pUr-pCr-pCr-pAr-pCr | Cyclic phosphorylation | 22 | 1.83 | 98.8 | 1 | 28.95 | 771.204 | -9 | 6947.8994 | 6950.95 | 6947.8867 | 487184.56 |
| 78-99 | Cr-pCr-pUr-pCr-pUr-pGr-pGr-pUr-pGr-pUr-pCr-pCr-pAr-pGr-pC | Cyclic phosphorylation | 22 | 1.39 | 100 | 2 | 28.97 | 703.782 | -10 | 7044.8877 | 7048.03 | 7044.8779 | 403399.34 |
| 78-99 | Cr-pCr-pUr-pCr-pUr-pGr-pGr-pUr-pGr-pUr-pCr-pCr-pAr-pGr-pC | Cyclic phosphorylation | 22 | 0.7 | 100 | 1 | 28.99 | 879.98 | -8 | 7044.8828 | 7047.84 | 7044.8779 | 529195.75 |
| 78-99 | Cr-pCr-pUr-pCr-pUr-pGr-pGr-pUr-pGr-pUr-pCr-pCr-pAr-pGr-pC | Cyclic phosphorylation | 22 | 1.39 | 100 | 1 | 29.01 | 782.093 | -9 | 7044.8877 | 7047.99 | 7044.8779 | 695654.44 |
| 3372-3392 | Cr-pGr-pGr-pAr-pAr-pCr-pUr-pUr-pCr-pUr-pAr-pCr-pGr-pAr-pG | Cyclic phosphorylation | 21 | 2.94 | 100 | 1 | 29.18 | 960.554 | -7 | 6728.9258 | 6731.51 | 6728.906 | 492135.69 |
| 2761-2781 | Cr-pCr-pUr-pAr-pCr-pCr-pGr-pGr-pUr-pUr-pCr-pAr-pAr-pCr-pG | Cyclic phosphorylation | 21 | 2.64 | 100 | 1 | 29.32 | 744.314 | -9 | 6705.8965 | 6708.9 | 6705.8788 | 1016238.31 |
| 2761-2781 | Cr-pCr-pUr-pAr-pCr-pCr-pGr-pGr-pUr-pUr-pCr-pAr-pAr-pCr-pG | Cyclic phosphorylation | 21 | 1.55 | 100 | 1.2 | 29.34 | 957.407 | -7 | 6705.8892 | 6708.81 | 6705.8788 | 340435.44 |
| 2761-2781 | Cr-pCr-pUr-pAr-pCr-pCr-pGr-pGr-pUr-pUr-pCr-pAr-pAr-pCr-pG | Cyclic phosphorylation | 21 | 1.63 | 100 | 1 | 29.35 | 837.605 | -8 | 6705.8896 | 6708.72 | 6705.8788 | 935964.44 |
| 1638-1655 | Ar-pAr-pAr-pAr-pGr-pCr-pAr-pCr-pCr-pAr-pAr-pUr-pCr-pUr-pCr | Cyclic phosphorylation | 18 | 3.33 | 96.6 | 1.2 | 29.44 | 722.094 | -8 | 5781.8115 | 5784.35 | 5781.7923 | 376173.88 |
| 2496-2516 | Cr-pGr-pGr-pAr-pGr-pCr-pUr-pUr-pCr-pAr-pUr-pCr-pGr-pAr-pG | Cyclic phosphorylation | 21 | 2.02 | 99.9 | 1.1 | 29.44 | 847.481 | -8 | 6785.9048 | 6788.79 | 6785.891 | 476989.62 |
| 2496-2516 | Cr-pGr-pGr-pAr-pGr-pCr-pUr-pUr-pCr-pAr-pUr-pCr-pGr-pAr-pG | Cyclic phosphorylation | 21 | 1.81 | 99.9 | 1.3 | 29.46 | 753.205 | -9 | 6785.9033 | 6788.76 | 6785.891 | 448158.94 |
| 926-947 | Ur-pGr-pCr-pUr-pCr-pUr-pGr-pGr-pAr-pUr-pCr-pCr-pUr-pCr-pU | Cyclic phosphorylation | 22 | 2.91 | 100 | 1.6 | 29.66 | 1009.266 | -7 | 7068.9097 | 7072.03 | 7068.8891 | 246601.31 |
| 926-947 | Ur-pGr-pCr-pUr-pCr-pUr-pGr-pGr-pAr-pUr-pCr-pCr-pUr-pCr-pU | Cyclic phosphorylation | 22 | 2.01 | 100 | 1 | 29.67 | 882.982 | -8 | 7068.9033 | 7072.05 | 7068.8891 | 566144.56 |
| 926-947 | Ur-pGr-pCr-pUr-pCr-pUr-pGr-pGr-pAr-pUr-pCr-pCr-pUr-pCr-pU | Cyclic phosphorylation | 22 | 2.35 | 100 | 1 | 29.67 | 784.649 | -9 | 7068.9058 | 7072 | 7068.8891 | 406006.22 |
| 1933-1951 | Ar-pUr-pCr-pAr-pGr-pCr-pUr-pGr-pAr-pCr-pAr-pCr-pCr-pUr-pA | Cyclic phosphorylation | 19 | 1.66 | 99.6 | 1.7 | 29.79 | 757.221 | -8 | 6063.8164 | 6066.46 | 6063.8063 | 278041.31 |
| 897-915 | Ar-pAr-pCr-pGr-pGr-pCr-pAr-pCr-pCr-pAr-pUr-pCr-pAr-pCr-pCr- | Cyclic phosphorylation | 19 | 0.62 | 100 | 1 | 29.88 | 761.852 | -8 | 6100.8643 | 6103.49 | 6100.8605 | 417198.56 |
| 897-915 | Ar-pAr-pCr-pGr-pGr-pCr-pAr-pCr-pCr-pAr-pUr-pCr-pAr-pCr-pCr- | Cyclic phosphorylation | 19 | 0.7 | 100 | 1 | 29.9 | 870.832 | -7 | 6100.8647 | 6103.71 | 6100.8605 | 709203.5 |
| 596-614 | Cr-pAr-pAr-pGr-pCr-pAr-pGr-pGr-pGr-pCr-pAr-pAr-pCr-pUr-pU | Cyclic phosphorylation | 19 | 1.5 | 100 | 1.1 | 29.9 | 880.118 | -7 | 6165.8711 | 6168.41 | 6165.8618 | 594938.94 |
| 2170-2190 | Cr-pCr-pUr-pAr-pCr-pUr-pCr-pCr-pAr-pAr-pCr-pAr-pAr-pCr-pUr | Cyclic phosphorylation | 21 | 0.84 | 100 | 1 | 29.91 | 941.403 | -7 | 6594.8662 | 6597.72 | 6594.8606 | 1857401.88 |
| 2170-2190 | Cr-pCr-pUr-pAr-pCr-pUr-pCr-pCr-pAr-pAr-pCr-pAr-pAr-pCr-pUr | Cyclic phosphorylation | 21 | 1.44 | 100 | 1 | 29.93 | 823.601 | -8 | 6594.8701 | 6597.68 | 6594.8606 | 4126986.75 |
| 2170-2190 | Cr-pCr-pUr-pAr-pCr-pUr-pCr-pCr-pAr-pAr-pCr-pAr-pAr-pCr-pUr | Cyclic phosphorylation | 21 | 1.44 | 100 | 1.5 | 29.93 | 2197.951 | -3 | 6594.8701 | 6597.67 | 6594.8606 | 902995.94 |
| 596-614 | Cr-pAr-pAr-pGr-pCr-pAr-pGr-pGr-pGr-pCr-pAr-pAr-pCr-pUr-pU | Cyclic phosphorylation | 19 | 1.74 | 100 | 1.1 | 29.93 | 769.977 | -8 | 6165.8726 | 6168.44 | 6165.8618 | 918212.12 |
| 2170-2190 | Cr-pCr-pUr-pAr-pCr-pUr-pCr-pCr-pAr-pAr-pCr-pAr-pAr-pCr-pUr | Cyclic phosphorylation | 21 | 1.96 | 100 | 1 | 29.93 | 1648.212 | -4 | 6594.8735 | 6597.72 | 6594.8606 | 850475.88 |
| 2170-2190 | Cr-pCr-pUr-pAr-pCr-pUr-pCr-pCr-pAr-pAr-pCr-pAr-pAr-pCr-pUr | Cyclic phosphorylation | 21 | 2.03 | 100 | 1.9 | 29.93 | 1318.568 | -5 | 6594.874 | 6597.71 | 6594.8606 | 584174.06 |
| 2170-2190 | Cr-pCr-pUr-pAr-pCr-pUr-pCr-pCr-pAr-pAr-pCr-pAr-pAr-pCr-pUr | Cyclic phosphorylation | 21 | 1.44 | 100 | 1 | 29.93 | 1098.639 | -6 | 6594.8701 | 6597.68 | 6594.8606 | 723169.75 |
| 897-915 | Ar-pAr-pCr-pGr-pGr-pCr-pAr-pCr-pCr-pAr-pUr-pCr-pAr-pCr-pCr- | Cyclic phosphorylation | 19 | 0.14 | 100 | 1 | 29.97 | 761.852 | -8 | 6100.8613 | 6103.48 | 6100.8605 | 527807.06 |
| 2170-2190 | Cr-pCr-pUr-pAr-pCr-pUr-pCr-pCr-pAr-pAr-pCr-pAr-pAr-pCr-pUr | Cyclic phosphorylation | 21 | 1.44 | 100 | 1 | 29.97 | 731.978 | -9 | 6594.8701 | 6597.68 | 6594.8606 | 3260720.25 |
| 956-977 | Cr-pAr-pCr-pCr-pCr-pUr-pGr-pAr-pAr-pGr-pUr-pCr-pCr-pUr-pU | Cyclic phosphorylation | 22 | 1.69 | 100 | 1.4 | 30.12 | 773.872 | -9 | 6971.9097 | 6974.79 | 6971.8979 | 642816.12 |
| 956-977 | Cr-pAr-pCr-pCr-pCr-pUr-pGr-pAr-pAr-pGr-pUr-pCr-pCr-pUr-pU | Cyclic phosphorylation | 22 | 1.76 | 100 | 1 | 30.12 | 870.732 | -8 | 6971.9102 | 6975.08 | 6971.8979 | 470211.34 |
| 2037-2059 | Ur-pGr-pCr-pGr-pAr-pCr-pAr-pUr-pCr-pCr-pCr-pCr-pAr-pUr-pCr | Cyclic phosphorylation | 23 | 3.25 | 100 | 1.2 | 30.19 | 818.324 | -9 | 7371.9863 | 7375.18 | 7371.9624 | 297551.44 |
| 2037-2059 | Ur-pGr-pCr-pGr-pAr-pCr-pAr-pUr-pCr-pCr-pCr-pCr-pAr-pUr-pCr | Cyclic phosphorylation | 23 | 1.53 | 100 | 1.2 | 30.19 | 920.865 | -8 | 7371.9736 | 7374.9 | 7371.9624 | 319242.69 |
| 3525-3543 | Ar-pAr-pCr-pCr-pAr-pCr-pAr-pCr-pAr-pAr-pGr-pCr-pCr-pCr-pCr- | Cyclic phosphorylation | 19 | 1.74 | 100 | 1.1 | 30.41 | 868.55 | -7 | 6083.8921 | 6086.6 | 6083.8815 | 349947.25 |
| 3525-3543 | Ar-pAr-pCr-pCr-pAr-pCr-pAr-pCr-pAr-pAr-pGr-pCr-pCr-pCr-pCr- | Cyclic phosphorylation | 19 | 1.66 | 100 | 1.1 | 30.41 | 759.855 | -8 | 6083.8916 | 6086.47 | 6083.8815 | 413928.31 |
| 3878-3897 | Ar-pCr-pUr-pCr-pGr-pAr-pGr-pCr-pUr-pGr-pGr-pUr-pAr-pCr-pU | Cyclic phosphorylation | 20 | 1.14 | 100 | 1.3 | 30.44 | 714.975 | -9 | 6441.835 | 6444.54 | 6441.8276 | 1834545.88 |
| 3878-3897 | Ar-pCr-pUr-pCr-pGr-pAr-pGr-pCr-pUr-pGr-pGr-pUr-pAr-pCr-pU | Cyclic phosphorylation | 20 | 1.37 | 100 | 1 | 30.46 | 804.472 | -8 | 6441.8364 | 6444.56 | 6441.8276 | 1714947 |
| 3878-3897 | Ar-pCr-pUr-pCr-pGr-pAr-pGr-pCr-pUr-pGr-pGr-pUr-pAr-pCr-pU | Cyclic phosphorylation | 20 | 2.12 | 100 | 1 | 30.46 | 919.542 | -7 | 6441.8413 | 6444.69 | 6441.8276 | 813220.62 |
| 3878-3897 | Ar-pCr-pUr-pCr-pGr-pAr-pGr-pCr-pUr-pGr-pGr-pUr-pAr-pCr-pU | Cyclic phosphorylation | 20 | 2.73 | 100 | 1.5 | 30.47 | 2146.941 | -3 | 6441.8452 | 6444.82 | 6441.8276 | 392154.69 |
| 1743-1764 | Cr-pAr-pGr-pUr-pUr-pUr-pGr-pGr-pCr-pCr-pGr-pGr-pGr-pAr-pU | Cyclic phosphorylation | 22 | 3.21 | 100 | 1.7 | 30.47 | 710.086 | -10 | 7107.9341 | 7111.05 | 7107.9113 | 576615.88 |
| 3878-3897 | Ar-pCr-pUr-pCr-pGr-pAr-pGr-pCr-pUr-pGr-pGr-pUr-pAr-pCr-pU | Cyclic phosphorylation | 20 | 0.99 | 100 | 1.5 | 30.47 | 1609.953 | -4 | 6441.834 | 6444.58 | 6441.8276 | 380322.94 |
| 1743-1764 | Cr-pAr-pGr-pUr-pUr-pUr-pGr-pGr-pCr-pCr-pGr-pGr-pGr-pAr-pU | Cyclic phosphorylation | 22 | 1.56 | 100 | 1 | 30.53 | 788.985 | -9 | 7107.9224 | 7111.09 | 7107.9113 | 860571.94 |
| 1743-1764 | Cr-pAr-pGr-pUr-pUr-pUr-pGr-pGr-pCr-pCr-pGr-pGr-pGr-pAr-pU | Cyclic phosphorylation | 22 | 1.84 | 100 | 1 | 30.54 | 887.859 | -8 | 7107.9243 | 7110.97 | 7107.9113 | 592227 |
| 3038-3056 | Ar-pCr-pUr-pGr-pAr-pUr-pCr-pAr-pCr-pAr-pGr-pGr-pCr-pAr-pG | Cyclic phosphorylation | 19 | 1.01 | 100 | 1 | 30.65 | 876.971 | -7 | 6142.8408 | 6145.43 | 6142.8346 | 694654.94 |
| 2545-2565 | Cr-pCr-pGr-pGr-pCr-pUr-pUr-pCr-pAr-pUr-pCr-pAr-pAr-pGr-pC | Cyclic phosphorylation | 21 | 2.83 | 98.3 | 1 | 30.66 | 747.092 | -9 | 6730.8931 | 6733.69 | 6730.874 | 635212.44 |
| 3038-3056 | Ar-pCr-pUr-pGr-pAr-pUr-pCr-pAr-pCr-pAr-pGr-pGr-pCr-pAr-pG | Cyclic phosphorylation | 19 | 1.8 | 100 | 1.5 | 30.67 | 681.754 | -9 | 6142.8457 | 6145.5 | 6142.8346 | 743147.75 |
| 3038-3056 | Ar-pCr-pUr-pGr-pAr-pUr-pCr-pAr-pCr-pAr-pGr-pGr-pCr-pAr-pG | Cyclic phosphorylation | 19 | 1.33 | 100 | 1 | 30.67 | 767.099 | -8 | 6142.8428 | 6145.6 | 6142.8346 | 1204584.75 |
| 1270-1290 | Ar-pAr-pGr-pUr-pGr-pCr-pGr-pGr-pCr-pAr-pGr-pAr-pUr-pUr-pG | Cyclic phosphorylation | 21 | 1.02 | 99.8 | 1.2 | 30.74 | 753.205 | -9 | 6785.8979 | 6788.72 | 6785.891 | 466819.69 |
| 2274-2295 | Ur-pAr-pCr-pAr-pUr-pCr-pUr-pGr-pCr-pGr-pGr-pCr-pGr-pAr-pU | Cyclic phosphorylation | 22 | 1.98 | 100 | 1 | 30.75 | 778.317 | -9 | 7011.918 | 7015.01 | 7011.9041 | 933028.5 |
| 2274-2295 | Ur-pAr-pCr-pAr-pUr-pCr-pUr-pGr-pCr-pGr-pGr-pCr-pGr-pAr-pU | Cyclic phosphorylation | 22 | 2.47 | 100 | 1 | 30.77 | 875.858 | -8 | 7011.9214 | 7014.95 | 7011.9041 | 819560.06 |
| 4059-4079 | Cr-pCr-pAr-pCr-pAr-pCr-pCr-pCr-pCr-pCr-pAr-pCr-pGr-pGr-pGr- | Cyclic phosphorylation | 21 | 1.59 | 100 | 1 | 30.93 | 841.115 | -8 | 6733.981 | 6736.87 | 6733.9702 | 4186730.5 |
| 4059-4079 | Cr-pCr-pAr-pCr-pAr-pCr-pCr-pCr-pCr-pCr-pAr-pCr-pGr-pGr-pGr- | Cyclic phosphorylation | 21 | 1.59 | 100 | 1 | 30.94 | 961.276 | -7 | 6733.981 | 6736.81 | 6733.9702 | 2557500.5 |
| 4059-4079 | Cr-pCr-pAr-pCr-pAr-pCr-pCr-pCr-pCr-pCr-pAr-pCr-pGr-pGr-pGr- | Cyclic phosphorylation | 21 | 0.79 | 100 | 1 | 30.94 | 747.435 | -9 | 6733.9756 | 6737.01 | 6733.9702 | 4251537.5 |
| 4059-4079 | Cr-pCr-pAr-pCr-pAr-pCr-pCr-pCr-pCr-pCr-pAr-pCr-pGr-pGr-pGr- | Cyclic phosphorylation | 21 | 1.23 | 100 | 1 | 30.94 | 1121.824 | -6 | 6733.9785 | 6736.9 | 6733.9702 | 1273051.75 |
| 4059-4079 | Cr-pCr-pAr-pCr-pAr-pCr-pCr-pCr-pCr-pCr-pAr-pCr-pGr-pGr-pGr- | Cyclic phosphorylation | 21 | 1.66 | 100 | 1.3 | 30.94 | 1682.989 | -4 | 6733.9814 | 6737.09 | 6733.9702 | 1104687.88 |
| 1038-1058 | Ur-pUr-pCr-pCr-pCr-pCr-pAr-pAr-pUr-pAr-pUr-pCr-pAr-pCr-pCr | Cyclic phosphorylation | 21 | 1.34 | 100 | 1 | 30.96 | 1648.457 | -4 | 6595.8535 | 6598.69 | 6595.8447 | 1689323.12 |
| 1038-1058 | Ur-pUr-pCr-pCr-pCr-pCr-pAr-pAr-pUr-pAr-pUr-pCr-pAr-pCr-pCr | Cyclic phosphorylation | 21 | 1.27 | 100 | 1 | 30.96 | 1098.803 | -6 | 6595.853 | 6598.68 | 6595.8447 | 1182763.38 |
| 1038-1058 | Ur-pUr-pCr-pCr-pCr-pCr-pAr-pAr-pUr-pAr-pUr-pCr-pAr-pCr-pCr | Cyclic phosphorylation | 21 | 1.19 | 100 | 1 | 30.98 | 658.778 | -10 | 6595.8525 | 6598.69 | 6595.8447 | 4128604 |
| 1038-1058 | Ur-pUr-pCr-pCr-pCr-pCr-pAr-pAr-pUr-pAr-pUr-pCr-pAr-pCr-pCr | Cyclic phosphorylation | 21 | 1.34 | 100 | 1 | 30.98 | 823.724 | -8 | 6595.8535 | 6598.81 | 6595.8447 | 6019362 |
| 1038-1058 | Ur-pUr-pCr-pCr-pCr-pCr-pAr-pAr-pUr-pAr-pUr-pCr-pAr-pCr-pCr | Cyclic phosphorylation | 21 | 1.64 | 100 | 1.1 | 30.98 | 2198.278 | -3 | 6595.8555 | 6598.69 | 6595.8447 | 1608310.75 |
| 1038-1058 | Ur-pUr-pCr-pCr-pCr-pCr-pAr-pAr-pUr-pAr-pUr-pCr-pAr-pCr-pCr | Cyclic phosphorylation | 21 | 1.34 | 100 | 1 | 30.98 | 732.198 | -9 | 6595.8535 | 6598.66 | 6595.8447 | 10935998 |
| 1038-1058 | Ur-pUr-pCr-pCr-pCr-pCr-pAr-pAr-pUr-pAr-pUr-pCr-pAr-pCr-pCr | Cyclic phosphorylation | 21 | 1.27 | 100 | 1 | 30.98 | 941.687 | -7 | 6595.853 | 6598.71 | 6595.8447 | 2415432.25 |
| 264-284 | Ur-pCr-pCr-pGr-pGr-pCr-pAr-pCr-pCr-pAr-pAr-pUr-pGr-pGr-pC | Cyclic phosphorylation | 21 | 1 | 95 | 1.8 | 31.18 | 1687.729 | -4 | 6751.9399 | 6755.3 | 6751.9332 | 810285.94 |
| 1001-1023 | Cr-pAr-pAr-pCr-pUr-pUr-pCr-pCr-pGr-pGr-pGr-pUr-pGr-pCr-pA | Cyclic phosphorylation | 23 | 1.72 | 100 | 1 | 31.19 | 816.548 | -9 | 7354.9961 | 7358.53 | 7354.9835 | 1264962.25 |
| 264-284 | Ur-pCr-pCr-pGr-pGr-pCr-pAr-pCr-pCr-pAr-pAr-pUr-pGr-pGr-pC | Cyclic phosphorylation | 21 | 1.36 | 95 | 1 | 31.19 | 749.541 | -9 | 6751.9424 | 6755.31 | 6751.9332 | 3968182.5 |
| 1001-1023 | Cr-pAr-pAr-pCr-pUr-pUr-pCr-pCr-pGr-pGr-pGr-pUr-pGr-pCr-pA | Cyclic phosphorylation | 23 | 1.32 | 100 | 1 | 31.2 | 918.742 | -8 | 7354.9932 | 7358.31 | 7354.9835 | 886275.75 |
| 264-284 | Ur-pCr-pCr-pGr-pGr-pCr-pAr-pCr-pCr-pAr-pAr-pUr-pGr-pGr-pC | Cyclic phosphorylation | 21 | 1.22 | 95 | 1 | 31.2 | 963.841 | -7 | 6751.9414 | 6755.21 | 6751.9332 | 1340091.12 |
| 1001-1023 | Cr-pAr-pAr-pCr-pUr-pUr-pCr-pCr-pGr-pGr-pGr-pUr-pGr-pCr-pA | Cyclic phosphorylation | 23 | 2.32 | 100 | 1.4 | 31.22 | 734.693 | -10 | 7355.0005 | 7358.28 | 7354.9835 | 426344.56 |
| 841-863 | Cr-pCr-pGr-pCr-pUr-pUr-pAr-pCr-pUr-pAr-pUr-pGr-pUr-pGr-pG | Cyclic phosphorylation | 23 | 2.32 | 99.9 | 1.2 | 31.27 | 814.318 | -9 | 7334.9253 | 7338.3 | 7334.9083 | 593806.69 |
| 841-863 | Cr-pCr-pGr-pCr-pUr-pUr-pAr-pCr-pUr-pAr-pUr-pGr-pUr-pGr-pG | Cyclic phosphorylation | 23 | 0.79 | 99.9 | 1 | 31.29 | 732.785 | -10 | 7334.9141 | 7338.01 | 7334.9083 | 625788.94 |
| 2895-2915 | Cr-pUr-pGr-pCr-pAr-pGr-pGr-pAr-pCr-pGr-pUr-pGr-pGr-pUr-pC | Cyclic phosphorylation | 21 | 1.1 | 97.4 | 1.9 | 31.39 | 755.874 | -9 | 6808.9258 | 6811.83 | 6808.9183 | 320065.16 |
| 976-995 | Ur-pGr-pGr-pAr-pAr-pAr-pAr-pGr-pGr-pGr-pCr-pAr-pUr-pCr-pU | Cyclic phosphorylation | 20 | 0.56 | 99.1 | 1 | 31.5 | 813.231 | -8 | 6511.897 | 6514.6 | 6511.8933 | 630380.62 |
| 976-995 | Ur-pGr-pGr-pAr-pAr-pAr-pAr-pGr-pGr-pGr-pCr-pAr-pUr-pCr-pU | Cyclic phosphorylation | 20 | 1.01 | 99.1 | 1.5 | 31.52 | 929.55 | -7 | 6511.8999 | 6514.5 | 6511.8933 | 324184.88 |
| 240-261 | Ar-pCr-pCr-pUr-pGr-pGr-pUr-pUr-pCr-pCr-pAr-pCr-pGr-pCr-pC | Cyclic phosphorylation | 22 | 1.22 | 100 | 1.3 | 31.52 | 696.385 | -10 | 6970.9224 | 6974.4 | 6970.9139 | 830892.44 |
| 240-261 | Ar-pCr-pCr-pUr-pGr-pGr-pUr-pUr-pCr-pCr-pAr-pCr-pGr-pCr-pC | Cyclic phosphorylation | 22 | 0.94 | 100 | 1 | 31.52 | 773.761 | -9 | 6970.9204 | 6974 | 6970.9139 | 1897221.75 |
| 240-261 | Ar-pCr-pCr-pUr-pGr-pGr-pUr-pUr-pCr-pCr-pAr-pCr-pGr-pCr-pC | Cyclic phosphorylation | 22 | 0.59 | 100 | 2 | 31.52 | 1742.474 | -4 | 6970.918 | 6973.88 | 6970.9139 | 342930.72 |
| 240-261 | Ar-pCr-pCr-pUr-pGr-pGr-pUr-pUr-pCr-pCr-pAr-pCr-pGr-pCr-pC | Cyclic phosphorylation | 22 | 0.59 | 100 | 1.1 | 31.52 | 995.125 | -7 | 6970.918 | 6974.06 | 6970.9139 | 508813.22 |
| 240-261 | Ar-pCr-pCr-pUr-pGr-pGr-pUr-pUr-pCr-pCr-pAr-pCr-pGr-pCr-pC | Cyclic phosphorylation | 22 | 0.87 | 100 | 1 | 31.53 | 870.733 | -8 | 6970.9199 | 6974.11 | 6970.9139 | 1185039.88 |
| 559-581 | Ar-pGr-pUr-pAr-pCr-pGr-pUr-pGr-pUr-pCr-pCr-pCr-pAr-pGr-pC | Cyclic phosphorylation | 23 | 1.5 | 99.9 | 1 | 31.9 | 809.873 | -9 | 7294.9131 | 7298.29 | 7294.9021 | 262079.25 |
| 1224-1245 | Ur-pGr-pCr-pUr-pUr-pCr-pAr-pCr-pAr-pAr-pAr-pCr-pGr-pUr-pG | Cyclic phosphorylation | 22 | 2.05 | 98.9 | 1.1 | 31.97 | 878.734 | -8 | 7035.9297 | 7038.99 | 7035.9153 | 325508.25 |
| 4059-4079 | Cr-pCr-pAr-pCr-pAr-pCr-pCr-pCr-pCr-pCr-pAr-pCr-pGr-pGr-pGr- | Phosphorylation | 21 | 1.4 | 100 | 1 | 32.01 | 963.849 | -7 | 6751.9902 | 6754.84 | 6751.9808 | 591113.38 |
| 4059-4079 | Cr-pCr-pAr-pCr-pAr-pCr-pCr-pCr-pCr-pCr-pAr-pCr-pGr-pGr-pGr- | Phosphorylation | 21 | 1.83 | 100 | 1 | 32.02 | 843.368 | -8 | 6751.9932 | 6754.71 | 6751.9808 | 428936.66 |

| | | | | | | | | | | | | | |
|---|---|---|---|---|---|---|---|---|---|---|---|---|---|
| 1038-1058 | Ur-pUr-pCr-pCr-pCr-pCr-pAr-pAr-pUr-pAr-pUr-pCr-pAr-pCr-pCr | Phosphorylation | 21 | 2.11 | 100 | 1.2 | 32.11 | 944.261 | -7 | 6613.8691 | 6616.69 | 6613.8552 | 333335.16 |
| 1038-1058 | Ur-pUr-pCr-pCr-pCr-pCr-pAr-pAr-pUr-pAr-pUr-pCr-pAr-pCr-pCr | Phosphorylation | 21 | 1.88 | 100 | 1 | 32.11 | 826.102 | -8 | 6613.8677 | 6616.71 | 6613.8552 | 464362.91 |
| 1038-1058 | Ur-pUr-pCr-pCr-pCr-pCr-pAr-pAr-pUr-pAr-pUr-pCr-pAr-pCr-pCr | Phosphorylation | 21 | 2.55 | 100 | 1 | 32.13 | 734.201 | -9 | 6613.8721 | 6616.86 | 6613.8552 | 223829.77 |
| 495-512 | Ar-pAr-pCr-pAr-pAr-pCr-pAr-pAr-pGr-pAr-pGr-pCr-pUr-pGr-pGı | Cyclic phosphorylation | 18 | 1.87 | 100 | 1.1 | 32.14 | 734.849 | -8 | 5884.8428 | 5887.5 | 5884.8318 | 396023.12 |
| 495-512 | Ar-pAr-pCr-pAr-pAr-pCr-pAr-pAr-pGr-pAr-pGr-pCr-pUr-pGr-pGı | Cyclic phosphorylation | 18 | 1.37 | 100 | 1 | 32.15 | 839.827 | -7 | 5884.8398 | 5887.35 | 5884.8318 | 559538.12 |
| 123-145 | Cr-pUr-pGr-pCr-pCr-pUr-pCr-pCr-pAr-pGr-pCr-pCr-pUr-pAr-pCr | Cyclic phosphorylation | 23 | 2.26 | 99.9 | 1.2 | 32.34 | 808.549 | -9 | 7283.0039 | 7285.91 | 7282.9875 | 272401.47 |
| 3350-3371 | Cr-pAr-pCr-pCr-pCr-pAr-pUr-pUr-pGr-pGr-pUr-pUr-pCr-pGr-pU | Cyclic phosphorylation | 22 | 2.25 | 97.3 | 1.2 | 32.43 | 781.097 | -9 | 7035.9312 | 7038.98 | 7035.9153 | 415275.38 |
| 3350-3371 | Cr-pAr-pCr-pCr-pCr-pAr-pUr-pUr-pGr-pGr-pUr-pUr-pCr-pGr-pU | Cyclic phosphorylation | 22 | 2.25 | 97.3 | 1 | 32.44 | 878.859 | -8 | 7035.9312 | 7038.86 | 7035.9153 | 331481.75 |
| 2476-2495 | Ar-pUr-pCr-pCr-pUr-pAr-pGr-pCr-pAr-pAr-pGr-pCr-pCr-pCr-pAr | Cyclic phosphorylation | 20 | -0.53 | 93.4 | 1.2 | 32.6 | 713.76 | -9 | 6430.8936 | 6433.42 | 6430.897 | 819628.69 |
| 2424-2445 | Ar-pCr-pCr-pCr-pCr-pUr-pCr-pCr-pUr-pAr-pUr-pCr-pAr-pAr-pGr | Cyclic phosphorylation | 22 | 1.1 | 100 | 1 | 32.62 | 868.732 | -8 | 6955.9106 | 6958.81 | 6955.903 | 1473778.5 |
| 2476-2495 | Ar-pUr-pCr-pCr-pUr-pAr-pGr-pCr-pAr-pAr-pGr-pCr-pCr-pCr-pAr | Cyclic phosphorylation | 20 | 0.07 | 93.4 | 1 | 32.64 | 803.106 | -8 | 6430.8975 | 6433.58 | 6430.897 | 820570.56 |
| 3304-3325 | Ar-pCr-pGr-pGr-pCr-pAr-pAr-pAr-pGr-pCr-pCr-pCr-pAr-pCr-pUr | Cyclic phosphorylation | 22 | 1.14 | 100 | 1 | 32.64 | 1001.985 | -7 | 7018.9443 | 7022.12 | 7018.9364 | 1422307 |
| 2424-2445 | Ar-pCr-pCr-pCr-pCr-pUr-pCr-pCr-pUr-pAr-pUr-pCr-pAr-pAr-pGr | Cyclic phosphorylation | 22 | 0.82 | 100 | 1.4 | 32.64 | 694.885 | -10 | 6955.9087 | 6958.82 | 6955.903 | 1119638.12 |
| 2424-2445 | Ar-pCr-pCr-pCr-pCr-pUr-pCr-pCr-pUr-pAr-pUr-pCr-pAr-pAr-pGr | Cyclic phosphorylation | 22 | 1.03 | 100 | 1 | 32.65 | 772.206 | -9 | 6955.9102 | 6958.85 | 6955.903 | 2201555.25 |
| 3304-3325 | Ar-pCr-pGr-pGr-pCr-pAr-pAr-pAr-pGr-pCr-pCr-pCr-pAr-pCr-pUr | Cyclic phosphorylation | 22 | 2.67 | 100 | 1.1 | 32.65 | 1169.151 | -6 | 7018.9551 | 7022.13 | 7018.9364 | 813225.81 |
| 3304-3325 | Ar-pCr-pGr-pGr-pCr-pAr-pAr-pAr-pGr-pCr-pCr-pCr-pAr-pCr-pUr | Cyclic phosphorylation | 22 | 1.21 | 100 | 1 | 32.65 | 876.736 | -8 | 7018.9448 | 7022.05 | 7018.9364 | 3254417.5 |
| 3304-3325 | Ar-pCr-pGr-pGr-pCr-pAr-pAr-pAr-pGr-pCr-pCr-pCr-pAr-pCr-pUr | Cyclic phosphorylation | 22 | 1.55 | 100 | 1 | 32.66 | 701.188 | -10 | 7018.9473 | 7022.03 | 7018.9364 | 3701586.25 |
| 3304-3325 | Ar-pCr-pGr-pGr-pCr-pAr-pAr-pAr-pGr-pCr-pCr-pCr-pAr-pCr-pUr | Cyclic phosphorylation | 22 | 1.42 | 100 | 1 | 32.67 | 779.097 | -9 | 7018.9463 | 7022.06 | 7018.9364 | 4912391 |
| 3304-3325 | Ar-pCr-pGr-pGr-pCr-pAr-pAr-pAr-pGr-pCr-pCr-pCr-pAr-pCr-pUr | Cyclic phosphorylation | 22 | 1.28 | 100 | 1.2 | 32.67 | 1754.481 | -4 | 7018.9453 | 7022.05 | 7018.9364 | 1140242.38 |
| 1172-1194 | Cr-pUr-pUr-pCr-pAr-pGr-pCr-pAr-pCr-pCr-pUr-pUr-pCr-pAr-pAı | Cyclic phosphorylation | 23 | 2.22 | 99.9 | 1.1 | 32.74 | 729.588 | -10 | 7301.9507 | 7305.19 | 7301.9344 | 823407.19 |
| 1172-1194 | Cr-pUr-pUr-pCr-pAr-pGr-pCr-pAr-pCr-pCr-pUr-pUr-pCr-pAr-pAı | Cyclic phosphorylation | 23 | 1.36 | 99.9 | 1 | 32.74 | 810.765 | -9 | 7301.9443 | 7305.2 | 7301.9344 | 1417262.5 |
| 1172-1194 | Cr-pUr-pUr-pCr-pAr-pGr-pCr-pAr-pCr-pCr-pUr-pUr-pCr-pAr-pAı | Cyclic phosphorylation | 23 | 1.56 | 99.9 | 1 | 32.76 | 911.986 | -8 | 7301.9458 | 7305.19 | 7301.9344 | 760991.25 |
| 3736-3757 | Ur-pGr-pAr-pUr-pGr-pGr-pUr-pCr-pAr-pCr-pAr-pAr-pUr-pCr-pA | Cyclic phosphorylation | 22 | 0.51 | 90.3 | 1.6 | 32.83 | 883.98 | -8 | 7077.8931 | 7080.71 | 7077.8895 | 574417.5 |
| 3736-3757 | Ur-pGr-pAr-pUr-pGr-pGr-pUr-pCr-pAr-pCr-pAr-pAr-pUr-pCr-pA | Cyclic phosphorylation | 22 | 0.3 | 90.3 | 1 | 32.83 | 785.648 | -9 | 7077.8916 | 7080.88 | 7077.8895 | 622625.12 |
| 198-221 | Cr-pAr-pCr-pUr-pCr-pUr-pAr-pCr-pCr-pCr-pAr-pGr-pGr-pAr-pCr | Cyclic phosphorylation | 24 | 0.75 | 99.7 | 1.1 | 33.04 | 841.991 | -9 | 7582.9702 | 7586.36 | 7582.9645 | 342223.5 |
| 198-221 | Cr-pAr-pCr-pUr-pCr-pUr-pAr-pCr-pCr-pCr-pAr-pGr-pGr-pAr-pCr | Cyclic phosphorylation | 24 | 1.27 | 99.7 | 1.6 | 33.06 | 947.241 | -8 | 7582.9741 | 7586.29 | 7582.9645 | 253847.23 |
| 1394-1414 | Cr-pAr-pAr-pCr-pUr-pAr-pCr-pAr-pAr-pUr-pUr-pAr-pCr-pCr-pUı | Cyclic phosphorylation | 21 | 1.45 | 100 | 1 | 33.18 | 831.604 | -8 | 6658.8877 | 6661.77 | 6658.878 | 374036.66 |
| 1394-1414 | Cr-pAr-pAr-pCr-pUr-pAr-pCr-pAr-pAr-pUr-pUr-pAr-pCr-pCr-pUı | Cyclic phosphorylation | 21 | 3.73 | 100 | 1.2 | 33.18 | 739.204 | -9 | 6658.9028 | 6661.79 | 6658.878 | 432727.34 |
| 4037-4058 | Cr-pAr-pGr-pCr-pUr-pCr-pAr-pAr-pAr-pCr-pGr-pCr-pUr-pUı | Cyclic phosphorylation | 22 | 1.55 | 99.9 | 1.5 | 33.36 | 879.738 | -8 | 7042.9585 | 7046.01 | 7042.9476 | 375664.41 |
| 4037-4058 | Cr-pAr-pGr-pCr-pUr-pCr-pAr-pAr-pAr-pCr-pGr-pCr-pUr-pUı | Cyclic phosphorylation | 22 | 1.06 | 99.9 | 1 | 33.37 | 781.766 | -9 | 7042.9551 | 7045.77 | 7042.9476 | 446428.12 |
| 1914-1937 | Cr-pCr-pCr-pGr-pUr-pGr-pGr-pCr-pCr-pAr-pUr-pUr-pCr-pAr-pCı | Cyclic phosphorylation | 24 | 0.9 | 100 | 1 | 33.39 | 850.551 | -9 | 7661.0156 | 7664.48 | 7661.0088 | 990169.69 |
| 1914-1937 | Cr-pCr-pCr-pGr-pUr-pGr-pGr-pCr-pCr-pAr-pUr-pUr-pCr-pAr-pCı | Cyclic phosphorylation | 24 | 2.04 | 100 | 1.1 | 33.41 | 956.87 | -8 | 7661.0244 | 7664.35 | 7661.0088 | 554110.31 |
| 1914-1937 | Cr-pCr-pCr-pGr-pUr-pGr-pGr-pCr-pCr-pAr-pUr-pUr-pCr-pAr-pCı | Cyclic phosphorylation | 24 | 1.6 | 100 | 1.2 | 33.41 | 765.395 | -10 | 7661.021 | 7664.59 | 7661.0088 | 1037054.12 |
| 1671-1691 | Ar-pAr-pCr-pUr-pUr-pCr-pAr-pAr-pCr-pUr-pUr-pCr-pAr-pAr-pCı | Cyclic phosphorylation | 21 | 1.85 | 100 | 1 | 33.43 | 952.977 | -7 | 6674.8853 | 6677.7 | 6674.8729 | 842691.81 |
| 1671-1691 | Ar-pAr-pCr-pUr-pUr-pCr-pAr-pAr-pCr-pUr-pUr-pCr-pAr-pAr-pCı | Cyclic phosphorylation | 21 | 1.63 | 100 | 1 | 33.44 | 833.603 | -8 | 6674.8838 | 6677.71 | 6674.8729 | 2002817.5 |
| 1671-1691 | Ar-pAr-pCr-pUr-pUr-pCr-pAr-pAr-pCr-pUr-pUr-pCr-pAr-pAr-pCı | Cyclic phosphorylation | 21 | 1.7 | 100 | 1 | 33.44 | 740.98 | -9 | 6674.8843 | 6677.75 | 6674.8729 | 2114552 |
| 1671-1691 | Ar-pAr-pCr-pUr-pUr-pCr-pAr-pAr-pCr-pUr-pUr-pCr-pAr-pAr-pCı | Cyclic phosphorylation | 21 | 1.99 | 100 | 1.9 | 33.44 | 1668.214 | -4 | 6674.8862 | 6677.76 | 6674.8729 | 388086 |
| 668-690 | Cr-pAr-pAr-pGr-pCr-pAr-pCr-pAr-pCr-pCr-pCr-pCr-pUr-pAr-pUr | Cyclic phosphorylation | 23 | 1.56 | 100 | 1.1 | 33.44 | 907.744 | -8 | 7267.0039 | 7270.26 | 7266.9926 | 389796.09 |
| 668-690 | Cr-pAr-pAr-pGr-pCr-pAr-pCr-pAr-pCr-pCr-pCr-pCr-pUr-pAr-pUr | Cyclic phosphorylation | 23 | 1.29 | 100 | 1.1 | 33.44 | 806.66 | -9 | 7267.002 | 7270.18 | 7266.9926 | 551637.69 |
| 1312-1332 | Ar-pCr-pUr-pAr-pCr-pAr-pCr-pUr-pAr-pCr-pAr-pAr-pGr-pCr-pCr | Cyclic phosphorylation | 21 | 1.66 | 98.6 | 1.2 | 33.58 | 836.484 | -8 | 6696.9272 | 6699.85 | 6696.9161 | 325449.91 |
| 2496-2519 | Cr-pGr-pGr-pAr-pGr-pCr-pUr-pUr-pCr-pAr-pUr-pCr-pGr-pAr-pG | Cyclic phosphorylation | 24 | 2.06 | 98.3 | 1.1 | 33.67 | 859.551 | -9 | 7742.021 | 7745.37 | 7742.0051 | 478870.78 |
| 2496-2519 | Cr-pGr-pGr-pAr-pGr-pCr-pUr-pUr-pCr-pAr-pUr-pCr-pGr-pAr-pG | Cyclic phosphorylation | 24 | 3.63 | 98.3 | 1.3 | 33.69 | 773.495 | -10 | 7742.0332 | 7744.96 | 7742.0051 | 444041.91 |
| 2111-2133 | Cr-pGr-pUr-pGr-pGr-pCr-pCr-pAr-pGr-pCr-pCr-pAr-pGr-pAr-pGı | Cyclic phosphorylation | 23 | 1.84 | 100 | 1.1 | 33.7 | 823.77 | -9 | 7419.9985 | 7423.12 | 7419.9849 | 591142.25 |
| 2111-2133 | Cr-pGr-pUr-pGr-pGr-pCr-pCr-pAr-pGr-pCr-pCr-pAr-pGr-pAr-pGı | Cyclic phosphorylation | 23 | 3.29 | 100 | 1.1 | 33.71 | 926.742 | -8 | 7420.0093 | 7423.59 | 7419.9849 | 353205.09 |
| 3525-3545 | Ar-pAr-pCr-pCr-pAr-pCr-pAr-pCr-pAr-pGr-pCr-pCr-pCr-pCr- | Cyclic phosphorylation | 21 | 0.41 | 99.9 | 1.4 | 33.88 | 841.239 | -8 | 6734.957 | 6738.23 | 6734.9543 | 366848.56 |
| 495-513 | Ar-pAr-pCr-pAr-pAr-pCr-pAr-pAr-pGr-pAr-pGr-pCr-pUr-pGr-pGı | Cyclic phosphorylation | 19 | 1.44 | 100 | 1.4 | 33.88 | 777.979 | -8 | 6229.8882 | 6232.57 | 6229.8792 | 382722.91 |
| 3525-3545 | Ar-pAr-pCr-pCr-pAr-pCr-pAr-pCr-pAr-pGr-pCr-pCr-pCr-pCr- | Cyclic phosphorylation | 21 | 1.57 | 99.9 | 1 | 33.9 | 747.545 | -9 | 6734.9648 | 6737.84 | 6734.9543 | 402851.34 |
| 495-513 | Ar-pAr-pCr-pAr-pAr-pCr-pAr-pAr-pGr-pAr-pGr-pCr-pUr-pGr-pGı | Cyclic phosphorylation | 19 | 1.59 | 100 | 1.1 | 33.92 | 889.407 | -7 | 6229.8892 | 6232.7 | 6229.8792 | 387190.09 |
| 2167-2190 | Ur-pGr-pGr-pCr-pCr-pUr-pAr-pCr-pUr-pCr-pCr-pAr-pAr-pCr-pAı | Cyclic phosphorylation | 24 | 1.69 | 100 | 1.1 | 34.2 | 948.243 | -8 | 7590.9937 | 7594.04 | 7590.9808 | 439151.16 |
| 2167-2190 | Ur-pGr-pGr-pCr-pCr-pUr-pAr-pCr-pUr-pCr-pCr-pAr-pAr-pCr-pAı | Cyclic phosphorylation | 24 | 1.95 | 100 | 1.3 | 34.2 | 758.393 | -10 | 7590.9956 | 7594.25 | 7590.9808 | 587472 |
| 2167-2190 | Ur-pGr-pGr-pCr-pCr-pUr-pAr-pCr-pUr-pCr-pCr-pAr-pAr-pCr-pAı | Cyclic phosphorylation | 24 | 1.43 | 100 | 1 | 34.23 | 842.77 | -9 | 7590.9917 | 7594.41 | 7590.9808 | 738208.69 |
| 3516-3535 | Ur-pAr-pCr-pUr-pUr-pUr-pAr-pAr-pGr-pAr-pAr-pCr-pCr-pAr-pCı | Cyclic phosphorylation | 20 | 2.26 | 100 | 1.4 | 34.25 | 710.425 | -9 | 6400.8896 | 6403.66 | 6400.8752 | 571076.31 |
| 3516-3535 | Ur-pAr-pCr-pUr-pUr-pUr-pAr-pAr-pGr-pAr-pAr-pCr-pCr-pAr-pCı | Cyclic phosphorylation | 20 | 1.27 | 100 | 1 | 34.26 | 799.354 | -8 | 6400.8833 | 6403.66 | 6400.8752 | 1036544.19 |
| 3516-3535 | Ur-pAr-pCr-pUr-pUr-pUr-pAr-pAr-pGr-pAr-pAr-pCr-pCr-pAr-pCı | Cyclic phosphorylation | 20 | 1.42 | 100 | 1 | 34.27 | 913.691 | -7 | 6400.8843 | 6403.78 | 6400.8752 | 625579.75 |
| 1801-1822 | Ar-pAr-pAr-pUr-pCr-pCr-pUr-pGr-pGr-pAr-pCr-pAr-pUr-pCr-pAı | Cyclic phosphorylation | 22 | 1.8 | 91.7 | 1.2 | 34.27 | 871.859 | -8 | 6979.9268 | 6983.08 | 6979.9142 | 424793.19 |
| 1801-1822 | Ar-pAr-pAr-pUr-pCr-pCr-pUr-pGr-pGr-pAr-pCr-pAr-pUr-pCr-pAı | Cyclic phosphorylation | 22 | 1.94 | 91.7 | 1 | 34.27 | 774.874 | -9 | 6979.9277 | 6983.06 | 6979.9142 | 608638.44 |
| 1038-1060 | Ur-pUr-pCr-pCr-pCr-pCr-pAr-pAr-pUr-pAr-pUr-pCr-pAr-pCr-pCr | Cyclic phosphorylation | 23 | 3.31 | 98.8 | 1.8 | 34.34 | 723.887 | -10 | 7246.9414 | 7250.09 | 7246.9174 | 432259.75 |
| 1038-1060 | Ur-pUr-pCr-pCr-pCr-pCr-pAr-pAr-pUr-pAr-pUr-pCr-pAr-pCr-pCr | Cyclic phosphorylation | 23 | 0.28 | 98.8 | 1.4 | 34.35 | 804.541 | -9 | 7246.9194 | 7250.18 | 7246.9174 | 344686.81 |
| 240-263 | Ar-pCr-pCr-pUr-pGr-pGr-pUr-pUr-pCr-pCr-pAr-pCr-pCr-pCr-pCı | Cyclic phosphorylation | 24 | 1.69 | 100 | 1.2 | 34.67 | 761.393 | -10 | 7621.9995 | 7625.33 | 7621.9866 | 467015.91 |
| 3206-3233 | Cr-pUr-pUr-pCr-pCr-pCr-pUr-pCr-pAr-pGr-pUr-pCr-pUr-pGr-pCı | Cyclic phosphorylation | 28 | 0.89 | 100 | 1.3 | 34.69 | 883.005 | -10 | 8836.1113 | 8840.27 | 8836.1035 | 412828.25 |
| 240-263 | Ar-pCr-pCr-pUr-pGr-pGr-pUr-pUr-pCr-pCr-pAr-pCr-pCr-pCr-pCı | Cyclic phosphorylation | 24 | 2.01 | 100 | 1.5 | 34.69 | 846.215 | -9 | 7622.002 | 7625.56 | 7621.9866 | 346469.84 |
| 3206-3233 | Cr-pUr-pUr-pCr-pCr-pCr-pUr-pCr-pAr-pGr-pUr-pCr-pUr-pGr-pCı | Cyclic phosphorylation | 28 | 1.11 | 100 | 1.4 | 34.71 | 802.732 | -11 | 8836.1133 | 8840.31 | 8836.1035 | 534403.06 |
| 864-887 | Cr-pAr-pGr-pCr-pCr-pUr-pAr-pGr-pAr-pAr-pCr-pCr-pUr-pUr-pCı | Cyclic phosphorylation | 24 | 1.11 | 99.5 | 1.2 | 34.79 | 766.296 | -10 | 7670.0176 | 7673.35 | 7670.0091 | 442997.78 |
| 1035-1058 | Cr-pGr-pGr-pUr-pUr-pCr-pCr-pCr-pCr-pAr-pAr-pUr-pAr-pUr-pCı | Cyclic phosphorylation | 24 | 2.08 | 99.2 | 1.2 | 34.88 | 948.117 | -8 | 7590.9966 | 7594.19 | 7590.9808 | 418031.25 |
| 1035-1058 | Cr-pGr-pGr-pUr-pUr-pCr-pCr-pCr-pCr-pAr-pAr-pUr-pAr-pUr-pCı | Cyclic phosphorylation | 24 | 1.24 | 99.2 | 1.1 | 34.9 | 758.393 | -10 | 7590.9902 | 7594.36 | 7590.9808 | 462573.28 |
| 1035-1058 | Cr-pGr-pGr-pUr-pUr-pCr-pCr-pCr-pCr-pAr-pAr-pUr-pAr-pUr-pCı | Cyclic phosphorylation | 24 | 1.24 | 99.2 | 1.1 | 34.9 | 842.659 | -9 | 7590.9902 | 7594.33 | 7590.9808 | 747007.06 |
| 627-648 | Ur-pUr-pCr-pGr-pUr-pGr-pUr-pUr-pUr-pAr-pAr-pGr-pAr-pAr-p( | Cyclic phosphorylation | 22 | 4.35 | 95.2 | 1 | 34.9 | 881.984 | -8 | 7061.9253 | 7063.83 | 7061.8946 | 315874.62 |
| 2908-2930 | Ur-pCr-pAr-pAr-pCr-pCr-pAr-pGr-pAr-pAr-pUr-pGr-pCr-pCr-pCr | Cyclic phosphorylation | 23 | 1.68 | 99.8 | 1 | 34.93 | 737.996 | -10 | 7387.0234 | 7390.29 | 7387.011 | 615431.25 |
| 2908-2930 | Ur-pCr-pAr-pAr-pCr-pCr-pAr-pGr-pAr-pAr-pUr-pGr-pCr-pCr-pCr | Cyclic phosphorylation | 23 | 1.48 | 99.8 | 1 | 34.93 | 820.107 | -9 | 7387.022 | 7390.2 | 7387.011 | 589241.19 |
| 2908-2930 | Ur-pCr-pAr-pAr-pCr-pCr-pAr-pGr-pAr-pAr-pUr-pGr-pCr-pCr-pCr | Cyclic phosphorylation | 23 | 1.15 | 99.8 | 1.5 | 34.95 | 922.746 | -8 | 7387.0195 | 7390.07 | 7387.011 | 303660.09 |
| 828-852 | Ar-pCr-pAr-pGr-pCr-pUr-pGr-pGr-pUr-pGr-pCr-pCr-pGr-pCr-pCı | Cyclic phosphorylation | 25 | 1 | 98.8 | 1.1 | 35.18 | 800.097 | -10 | 8008.0322 | 8011.64 | 8008.0242 | 868440.44 |
| 828-852 | Ar-pCr-pAr-pGr-pCr-pUr-pGr-pGr-pUr-pGr-pCr-pCr-pGr-pCr-pCı | Cyclic phosphorylation | 25 | 0.82 | 98.8 | 1.2 | 35.19 | 889.22 | -9 | 8008.0308 | 8011.64 | 8008.0242 | 540503.62 |
| 3823-3845 | Ar-pCr-pGr-pAr-pGr-pGr-pAr-pCr-pGr-pAr-pUr-pUr-pCr-pUr-pG | Cyclic phosphorylation | 23 | -0.04 | 100 | 1 | 35.19 | 828.216 | -9 | 7459.9907 | 7463.28 | 7459.991 | 789798.88 |
| 3823-3845 | Ar-pCr-pGr-pAr-pGr-pGr-pAr-pCr-pGr-pAr-pUr-pUr-pCr-pUr-pG | Cyclic phosphorylation | 23 | 2.78 | 100 | 1.1 | 35.23 | 745.293 | -10 | 7460.0117 | 7463.1 | 7459.991 | 907487 |
| 639-659 | Ar-pAr-pCr-pAr-pUr-pCr-pGr-pAr-pCr-pGr-pGr-pCr-pUr-pAr-pCı | Cyclic phosphorylation | 21 | 1.44 | 99.4 | 1.4 | 35.23 | 747.983 | -9 | 6737.916 | 6740.89 | 6737.9063 | 479604.12 |
| 639-659 | Ar-pAr-pCr-pAr-pUr-pCr-pGr-pAr-pCr-pGr-pGr-pCr-pUr-pAr-pCı | Cyclic phosphorylation | 21 | 0.86 | 99.4 | 1 | 35.24 | 841.483 | -8 | 6737.9121 | 6740.78 | 6737.9063 | 665244.56 |
| 3112-3137 | Cr-pCr-pUr-pCr-pUr-pGr-pCr-pCr-pAr-pAr-pUr-pCr-pUr-pGr-pGı | Cyclic phosphorylation | 26 | 1.33 | 100 | 1.4 | 35.37 | 687.169 | -12 | 8255.1074 | 8259.03 | 8255.0964 | 456924.88 |
| 3112-3137 | Cr-pCr-pUr-pCr-pUr-pGr-pCr-pCr-pAr-pAr-pUr-pCr-pUr-pGr-pGı | Cyclic phosphorylation | 26 | 0.98 | 100 | 1.2 | 35.42 | 749.821 | -11 | 8255.1045 | 8258.96 | 8255.0964 | 1502780 |
| 3112-3137 | Cr-pCr-pUr-pCr-pUr-pGr-pCr-pCr-pAr-pAr-pUr-pCr-pUr-pGr-pGı | Cyclic phosphorylation | 26 | 2.28 | 100 | 1 | 35.44 | 916.672 | -9 | 8255.1152 | 8258.82 | 8255.0964 | 1631256.88 |
| 3112-3137 | Cr-pCr-pUr-pCr-pUr-pGr-pCr-pCr-pAr-pAr-pUr-pCr-pUr-pGr-pGı | Cyclic phosphorylation | 26 | 2.04 | 100 | 1 | 35.45 | 824.804 | -10 | 8255.1133 | 8258.62 | 8255.0964 | 2099670.75 |
| 3112-3137 | Cr-pCr-pUr-pCr-pUr-pGr-pCr-pCr-pAr-pAr-pUr-pCr-pUr-pGr-pGı | Cyclic phosphorylation | 26 | 3.23 | 100 | 1.2 | 35.45 | 1031.257 | -8 | 8255.123 | 8258.66 | 8255.0964 | 822603.69 |
| 1845-1867 | Ar-pUr-pCr-pCr-pAr-pCr-pCr-pCr-pUr-pGr-pGr-pCr-pAr-pCr-pCr | Cyclic phosphorylation | 23 | 1.8 | 100 | 1 | 35.51 | 912.622 | -8 | 7306.0278 | 7309.28 | 7306.0147 | 1045657.75 |
| 1845-1867 | Ar-pUr-pCr-pCr-pAr-pCr-pCr-pCr-pUr-pGr-pGr-pCr-pAr-pCr-pCr | Cyclic phosphorylation | 23 | 1.2 | 100 | 1 | 35.52 | 810.996 | -9 | 7306.0234 | 7309.23 | 7306.0147 | 1816351 |
| 1845-1867 | Ar-pUr-pCr-pCr-pAr-pCr-pCr-pCr-pUr-pGr-pGr-pCr-pAr-pCr-pCr | Cyclic phosphorylation | 23 | 1.53 | 100 | 1.1 | 35.54 | 729.896 | -10 | 7306.0259 | 7309.3 | 7306.0147 | 1370273.75 |
| 3016-3037 | Ar-pGr-pGr-pCr-pGr-pAr-pGr-pGr-pUr-pGr-pCr-pGr-pAr-pGr-pA | Cyclic phosphorylation | 22 | 1.21 | 99.9 | 1 | 35.56 | 801.106 | -9 | 7217.0078 | 7220.06 | 7216.9991 | 645693.44 |
| 1305-1325 | Ar-pUr-pGr-pCr-pCr-pGr-pAr-pCr-pUr-pAr-pCr-pAr-pCr-pUr-pAr | Cyclic phosphorylation | 21 | 1.51 | 100 | 1 | 35.63 | 745.986 | -9 | 6720.9375 | 6723.82 | 6720.9274 | 1966024.75 |
| 1305-1325 | Ar-pUr-pGr-pCr-pCr-pGr-pAr-pCr-pUr-pAr-pCr-pAr-pCr-pUr-pAr | Cyclic phosphorylation | 21 | 1.22 | 100 | 1 | 35.63 | 839.36 | -8 | 6720.9355 | 6723.76 | 6720.9274 | 1152797 |
| 1533-1557 | Ur-pCr-pCr-pUr-pAr-pCr-pGr-pGr-pCr-pUr-pUr-pUr-pCr-pAr-pG | Cyclic phosphorylation | 25 | 1.3 | 100 | 1 | 35.73 | 991.374 | -8 | 7936.0386 | 7939.54 | 7936.0282 | 1044364.5 |
| 1533-1557 | Ur-pCr-pCr-pUr-pAr-pCr-pGr-pGr-pCr-pUr-pUr-pUr-pCr-pAr-pG | Cyclic phosphorylation | 25 | 1.49 | 100 | 1 | 35.73 | 881.109 | -9 | 7936.04 | 7939.74 | 7936.0282 | 2272957 |
| 1533-1557 | Ur-pCr-pCr-pUr-pAr-pCr-pGr-pGr-pCr-pUr-pUr-pUr-pCr-pAr-pG | Cyclic phosphorylation | 25 | 1.55 | 100 | 1.8 | 35.73 | 1133.142 | -7 | 7936.0405 | 7939.76 | 7936.0282 | 599107 |
| 1533-1557 | Ur-pCr-pCr-pUr-pAr-pCr-pGr-pGr-pCr-pUr-pUr-pUr-pCr-pAr-pG | Cyclic phosphorylation | 25 | 1.55 | 100 | 1 | 35.76 | 792.897 | -10 | 7936.0405 | 7939.4 | 7936.0282 | 2779533 |
| 1533-1557 | Ur-pCr-pCr-pUr-pAr-pCr-pGr-pGr-pCr-pUr-pUr-pUr-pCr-pAr-pG | Cyclic phosphorylation | 25 | 1.67 | 100 | 1.2 | 35.77 | 720.724 | -11 | 7936.0415 | 7939.48 | 7936.0282 | 2124659 |
| 1953-1977 | Cr-pGr-pGr-pUr-pGr-pUr-pAr-pCr-pUr-pCr-pAr-pCr-pAr-pCr-pCı | Cyclic phosphorylation | 25 | 3.47 | 100 | 1.2 | 35.77 | 806.402 | -10 | 8070.1016 | 8073.99 | 8070.0736 | 730915.19 |
| 1953-1977 | Cr-pGr-pGr-pUr-pGr-pUr-pAr-pCr-pUr-pCr-pAr-pCr-pAr-pCr-pCı | Cyclic phosphorylation | 25 | 1.9 | 100 | 1 | 35.8 | 896.004 | -9 | 8070.0889 | 8073.42 | 8070.0736 | 643860.75 |
| 3237-3261 | Ur-pUr-pUr-pCr-pUr-pGr-pCr-pAr-pCr-pGr-pUr-pGr-pAr-pCr-pA | Cyclic phosphorylation | 25 | 1.44 | 100 | 1 | 35.87 | 884.993 | -9 | 7969.9976 | 7973.61 | 7969.9861 | 1173694 |
| 3237-3261 | Ur-pUr-pUr-pCr-pUr-pGr-pCr-pAr-pCr-pGr-pUr-pGr-pAr-pCr-pA | Cyclic phosphorylation | 25 | 1.62 | 100 | 1.7 | 35.87 | 723.811 | -11 | 7969.999 | 7973.63 | 7969.9861 | 1712317.75 |
| 3237-3261 | Ur-pUr-pUr-pCr-pUr-pGr-pCr-pAr-pCr-pGr-pUr-pGr-pAr-pCr-pA | Cyclic phosphorylation | 25 | 1.99 | 100 | 1 | 35.89 | 796.293 | -10 | 7970.002 | 7973.61 | 7969.9861 | 1961735.62 |
| 2447-2471 | Cr-pGr-pGr-pCr-pUr-pUr-pCr-pCr-pAr-pAr-pUr-pUr-pCr-pAr-pG | Cyclic phosphorylation | 25 | 2.52 | 95.6 | 1 | 35.96 | 794.694 | -10 | 7954.0112 | 7957.64 | 7953.9912 | 895044.25 |
| 2447-2471 | Cr-pGr-pGr-pCr-pUr-pUr-pCr-pCr-pAr-pAr-pUr-pUr-pCr-pAr-pG | Cyclic phosphorylation | 25 | 0.8 | 95.6 | 1 | 35.96 | 883.105 | -9 | 7953.9976 | 7957.57 | 7953.9912 | 881192.5 |
| 1703-1724 | Cr-pGr-pUr-pGr-pCr-pUr-pGr-pAr-pCr-pAr-pGr-pAr-pGr-pAr-pG | Cyclic phosphorylation | 22 | 1.57 | 96.6 | 1.3 | 35.98 | 897.371 | -8 | 7185.0205 | 7188.25 | 7185.0092 | 378880.66 |
| 1703-1724 | Cr-pGr-pUr-pGr-pCr-pUr-pGr-pAr-pCr-pAr-pGr-pAr-pGr-pAr-pG | Cyclic phosphorylation | 22 | 1.7 | 96.6 | 1.3 | 35.98 | 797.662 | -9 | 7185.0215 | 7188.22 | 7185.0092 | 454275.53 |
| 306-330 | Cr-pCr-pCr-pUr-pUr-pCr-pAr-pAr-pCr-pGr-pAr-pCr-pGr-pGr-pGı | Cyclic phosphorylation | 25 | 1.9 | 98.1 | 1 | 36.01 | 800.195 | -10 | 8009.0234 | 8012.39 | 8009.0082 | 1614694.38 |
| 306-330 | Cr-pCr-pCr-pUr-pUr-pCr-pAr-pAr-pCr-pGr-pAr-pCr-pGr-pGr-pGı | Cyclic phosphorylation | 25 | 1.53 | 98.1 | 1 | 36.02 | 889.218 | -9 | 8009.0205 | 8012.49 | 8009.0082 | 1228026.12 |
| 306-330 | Cr-pCr-pCr-pUr-pUr-pCr-pAr-pAr-pCr-pGr-pAr-pCr-pGr-pGr-pGı | Cyclic phosphorylation | 25 | 3.24 | 98.1 | 1.1 | 36.03 | 727.358 | -11 | 8009.0342 | 8012.46 | 8009.0082 | 1117471.38 |
| 306-330 | Cr-pCr-pCr-pUr-pUr-pCr-pAr-pAr-pCr-pGr-pAr-pCr-pGr-pGr-pGı | Cyclic phosphorylation | 25 | 2.08 | 98.1 | 1.2 | 36.03 | 1000.496 | -8 | 8009.0249 | 8012.54 | 8009.0082 | 545171.19 |
| 3206-3234 | Cr-pUr-pUr-pCr-pCr-pCr-pUr-pCr-pAr-pGr-pUr-pCr-pUr-pGr-pCı | Cyclic phosphorylation | 29 | -1.55 | 99.1 | 1.4 | 36.2 | 764.34 | -12 | 9181.1367 | 9185.33 | 9181.1509 | 422310.69 |
| 2191-2215 | Cr-pUr-pAr-pUr-pCr-pCr-pCr-pAr-pCr-pCr-pAr-pAr-pCr-pCr-pUı | Cyclic phosphorylation | 25 | 1.57 | 100 | 1 | 36.2 | 870.332 | -9 | 7839.0493 | 7842.42 | 7839.037 | 2929388 |
| 2191-2215 | Cr-pUr-pAr-pUr-pCr-pCr-pCr-pAr-pCr-pCr-pAr-pAr-pCr-pCr-pUı | Cyclic phosphorylation | 25 | 1.32 | 100 | 1 | 36.21 | 979.25 | -8 | 7839.0474 | 7842.39 | 7839.037 | 1266808.88 |
| 2191-2215 | Cr-pUr-pAr-pUr-pCr-pCr-pCr-pAr-pCr-pCr-pAr-pAr-pCr-pCr-pUı | Cyclic phosphorylation | 25 | 0.76 | 100 | 1.3 | 36.22 | 1119.287 | -7 | 7839.043 | 7842.49 | 7839.037 | 515332.44 |
| 2191-2215 | Cr-pUr-pAr-pUr-pCr-pCr-pCr-pAr-pCr-pCr-pAr-pAr-pCr-pCr-pUı | Cyclic phosphorylation | 25 | 3 | 100 | 1.8 | 36.22 | 1306.001 | -6 | 7839.0605 | 7842.48 | 7839.037 | 425343.12 |
| 2191-2215 | Cr-pUr-pAr-pUr-pCr-pCr-pCr-pAr-pCr-pCr-pAr-pAr-pCr-pCr-pUı | Cyclic phosphorylation | 25 | 1.69 | 100 | 1.1 | 36.22 | 711.997 | -11 | 7839.0503 | 7842.49 | 7839.037 | 3209219.25 |
| 2191-2215 | Cr-pUr-pAr-pUr-pCr-pCr-pCr-pAr-pCr-pCr-pAr-pAr-pCr-pCr-pUı | Cyclic phosphorylation | 25 | 1.51 | 100 | 1 | 36.23 | 783.197 | -10 | 7839.0488 | 7842.45 | 7839.037 | 3642920.5 |
| 2191-2215 | Cr-pUr-pAr-pUr-pCr-pCr-pCr-pAr-pCr-pCr-pAr-pAr-pCr-pCr-pUı | Cyclic phosphorylation | 25 | 2 | 100 | 1.8 | 36.23 | 1959.256 | -4 | 7839.0527 | 7842.44 | 7839.037 | 946234.56 |
| 3206-3234 | Cr-pUr-pUr-pCr-pCr-pCr-pUr-pCr-pAr-pGr-pUr-pCr-pUr-pGr-pCı | Cyclic phosphorylation | 29 | 1.43 | 99.1 | 1.9 | 36.23 | 834.009 | -11 | 9181.1641 | 9185.55 | 9181.1509 | 248120.06 |
| 2229-2252 | Ar-pUr-pCr-pCr-pUr-pGr-pCr-pCr-pUr-pGr-pUr-pGr-pUr-pCr-pC | Cyclic phosphorylation | 24 | 0.77 | 100 | 1.5 | 36.46 | 955.118 | -8 | 7646.9878 | 7650.36 | 7646.9819 | 297910.31 |
| 2229-2252 | Ar-pUr-pCr-pCr-pUr-pGr-pCr-pCr-pUr-pGr-pUr-pGr-pUr-pCr-pC | Cyclic phosphorylation | 24 | 2.37 | 100 | 1 | 36.46 | 763.993 | -10 | 7647 | 7650.38 | 7646.9819 | 988018.38 |
| 2229-2252 | Ar-pUr-pCr-pCr-pUr-pGr-pCr-pCr-pUr-pGr-pUr-pGr-pUr-pCr-pC | Cyclic phosphorylation | 24 | 1.8 | 100 | 1 | 36.46 | 848.881 | -9 | 7646.9956 | 7650.48 | 7646.9819 | 662917.75 |

| | | | | | | | | | | | | | |
|---|---|---|---|---|---|---|---|---|---|---|---|---|---|
| 3797-3822 | Ur-pAr-pGr-pCr-pUr-pGr-pUr-pGr-pGr-pCr-pAr-pGr-pCr-pUr-pC | Cyclic phosphorylation | 26 | 2.99 | 99.9 | 1.2 | 36.55 | 762.364 | -11 | 8394.0869 | 8397.8 | 8394.0618 | 775049.94 |
| 3797-3822 | Ur-pAr-pGr-pCr-pUr-pGr-pUr-pGr-pGr-pCr-pAr-pGr-pCr-pUr-pC | Cyclic phosphorylation | 26 | 1.13 | 99.9 | 1 | 36.57 | 838.801 | -10 | 8394.0713 | 8397.84 | 8394.0618 | 1250477.62 |
| 3797-3822 | Ur-pAr-pGr-pCr-pUr-pGr-pUr-pGr-pGr-pCr-pAr-pGr-pCr-pUr-pC | Cyclic phosphorylation | 26 | 2.06 | 99.9 | 1 | 36.57 | 931.891 | -9 | 8394.0791 | 8397.53 | 8394.0618 | 756635.25 |
| 4059-4082 | Cr-pCr-pAr-pCr-pAr-pCr-pCr-pCr-pCr-pAr-pCr-pGr-pGr-pGr- | Cyclic phosphorylation | 24 | 5.61 | 100 | 1.1 | 36.57 | 963.634 | -8 | 7713.1548 | 7716.77 | 7713.1115 | 448077.69 |
| 4059-4082 | Cr-pCr-pAr-pCr-pAr-pCr-pCr-pCr-pCr-pAr-pCr-pGr-pGr-pGr- | Cyclic phosphorylation | 24 | 1.5 | 100 | 1.4 | 36.58 | 770.605 | -10 | 7713.123 | 7716.51 | 7713.1115 | 626500.5 |
| 4059-4082 | Cr-pCr-pAr-pCr-pAr-pCr-pCr-pCr-pCr-pAr-pCr-pGr-pGr-pGr- | Cyclic phosphorylation | 24 | 1.44 | 100 | 1.1 | 36.58 | 856.452 | -9 | 7713.1226 | 7716.42 | 7713.1115 | 634805.06 |
| 1453-1475 | Ar-pCr-pAr-pUr-pCr-pUr-pCr-pCr-pAr-pCr-pCr-pGr-pAr-pGr-pAr | Cyclic phosphorylation | 23 | 1.34 | 99.9 | 1 | 36.6 | 811.435 | -9 | 7308.9766 | 7312.15 | 7308.9667 | 664771.81 |
| 1453-1475 | Ar-pCr-pAr-pUr-pCr-pUr-pCr-pCr-pAr-pCr-pCr-pGr-pAr-pGr-pAr | Cyclic phosphorylation | 23 | 2.61 | 99.9 | 1.1 | 36.62 | 912.991 | -8 | 7308.9858 | 7312.05 | 7308.9667 | 336415.38 |
| 1453-1475 | Ar-pCr-pAr-pUr-pCr-pUr-pCr-pCr-pAr-pCr-pCr-pGr-pAr-pGr-pAr | Cyclic phosphorylation | 23 | 1.74 | 99.9 | 1.8 | 36.62 | 730.191 | -10 | 7308.9795 | 7312.09 | 7308.9667 | 534336 |
| 13-38 | Ur-pAr-pUr-pUr-pCr-pUr-pUr-pCr-pUr-pGr-pGr-pUr-pCr-pCr-pC | Cyclic phosphorylation | 26 | 1.43 | 100 | 1 | 36.81 | 912.553 | -9 | 8219.0381 | 8222.64 | 8219.0263 | 564350.81 |
| 13-38 | Ur-pAr-pUr-pUr-pCr-pUr-pUr-pCr-pUr-pGr-pGr-pUr-pCr-pCr-pC | Cyclic phosphorylation | 26 | 2.5 | 100 | 1 | 36.81 | 821.197 | -10 | 8219.0469 | 8222.8 | 8219.0263 | 1013612.19 |
| 13-38 | Ur-pAr-pUr-pUr-pCr-pUr-pUr-pCr-pUr-pGr-pGr-pUr-pCr-pCr-pC | Cyclic phosphorylation | 26 | 0.36 | 100 | 1 | 36.85 | 746.451 | -11 | 8219.0293 | 8222.79 | 8219.0263 | 756447.88 |
| 2085-2107 | Ar-pCr-pAr-pAr-pAr-pCr-pAr-pGr-pCr-pCr-pCr-pUr-pCr-pGr-pGr | Cyclic phosphorylation | 23 | -5.48 | 96.5 | 1.1 | 36.86 | 827.002 | -9 | 7449.0195 | 7452.08 | 7449.0604 | 360035.53 |
| 2191-2215 | Cr-pUr-pAr-pUr-pCr-pCr-pCr-pCr-pAr-pCr-pCr-pAr-pAr-pCr-pUr | Phosphorylation | 25 | 1.59 | 100 | 1.1 | 36.95 | 981.501 | -8 | 7857.0601 | 7860.82 | 7857.0476 | 506315.06 |
| 2191-2215 | Cr-pUr-pAr-pUr-pCr-pCr-pCr-pCr-pAr-pCr-pCr-pAr-pAr-pCr-pUr | Phosphorylation | 25 | 2.15 | 100 | 1 | 36.98 | 872.333 | -9 | 7857.0645 | 7860.14 | 7857.0476 | 598492.25 |
| 4085-4105 | Ar-pUr-pUr-pAr-pAr-pCr-pCr-pUr-pUr-pUr-pAr-pGr-pCr-pAr-pA | Cyclic phosphorylation | 21 | 1.71 | 100 | 1.4 | 37 | 1676.717 | -4 | 6707.896 | 6710.87 | 6707.8845 | 397426.59 |
| 4085-4105 | Ar-pUr-pUr-pAr-pAr-pCr-pCr-pUr-pUr-pUr-pAr-pGr-pCr-pAr-pA | Cyclic phosphorylation | 21 | 2.59 | 100 | 1 | 37 | 744.537 | -9 | 6707.9019 | 6710.81 | 6707.8845 | 1820028.5 |
| 4085-4105 | Ar-pUr-pUr-pAr-pAr-pCr-pCr-pUr-pUr-pUr-pAr-pGr-pCr-pAr-pA | Cyclic phosphorylation | 21 | 1.06 | 100 | 1 | 37.02 | 837.73 | -8 | 6707.8916 | 6710.67 | 6707.8845 | 1805777.12 |
| 4085-4105 | Ar-pUr-pUr-pAr-pAr-pCr-pCr-pUr-pUr-pUr-pAr-pGr-pCr-pAr-pA | Cyclic phosphorylation | 21 | 0.62 | 100 | 1 | 37.03 | 957.549 | -7 | 6707.8887 | 6710.77 | 6707.8845 | 582758.38 |
| 1914-1940 | Cr-pCr-pCr-pGr-pUr-pGr-pCr-pCr-pAr-pUr-pUr-pCr-pAr-pCr | Cyclic phosphorylation | 27 | -0.54 | 98.4 | 1.2 | 37.07 | 956.675 | -9 | 8617.1182 | 8621.05 | 8617.1228 | 284699.75 |
| 1914-1940 | Cr-pCr-pCr-pGr-pUr-pGr-pCr-pCr-pAr-pUr-pUr-pCr-pAr-pCr | Cyclic phosphorylation | 27 | 1.73 | 98.4 | 1.4 | 37.12 | 782.733 | -11 | 8617.1377 | 8621.27 | 8617.1228 | 759388.56 |
| 3594-3615 | Ar-pAr-pAr-pGr-pAr-pGr-pAr-pUr-pCr-pGr-pAr-pCr-pCr-pGr-pG | Cyclic phosphorylation | 22 | 1.5 | 99.9 | 1 | 37.19 | 797.662 | -9 | 7185.02 | 7187.99 | 7185.0092 | 549397.44 |
| 3594-3615 | Ar-pAr-pAr-pGr-pAr-pGr-pAr-pUr-pCr-pGr-pAr-pCr-pCr-pGr-pG | Cyclic phosphorylation | 22 | 1.77 | 99.9 | 1 | 37.19 | 897.371 | -8 | 7185.022 | 7188.01 | 7185.0092 | 376524.88 |
| 102-122 | Ar-pAr-pCr-pCr-pUr-pGr-pAr-pCr-pCr-pAr-pCr-pCr-pAr-pGr-pAr | Cyclic phosphorylation | 21 | 1.68 | 97.9 | 1.3 | 37.28 | 748.548 | -9 | 6742.9819 | 6746.13 | 6742.9706 | 436381.97 |
| 897-920 | Ar-pAr-pCr-pGr-pGr-pCr-pAr-pCr-pCr-pAr-pUr-pCr-pAr-pCr-pCr | Cyclic phosphorylation | 24 | 2.13 | 100 | 1 | 37.3 | 855.668 | -9 | 7707.0796 | 7710.81 | 7707.0632 | 851305.5 |
| 102-122 | Ar-pAr-pCr-pCr-pUr-pGr-pAr-pCr-pCr-pAr-pCr-pCr-pAr-pGr-pAr | Cyclic phosphorylation | 21 | 0.45 | 97.9 | 1.8 | 37.3 | 842.116 | -8 | 6742.9736 | 6746.25 | 6742.9706 | 374000.56 |
| 897-920 | Ar-pAr-pCr-pGr-pGr-pCr-pAr-pCr-pCr-pAr-pUr-pCr-pAr-pCr-pCr | Cyclic phosphorylation | 24 | 2.19 | 100 | 1.1 | 37.31 | 770.001 | -10 | 7707.0801 | 7710.54 | 7707.0632 | 1219229.62 |
| 897-920 | Ar-pAr-pCr-pGr-pGr-pCr-pAr-pCr-pCr-pAr-pUr-pCr-pAr-pCr-pCr | Cyclic phosphorylation | 24 | 2.38 | 100 | 1.1 | 37.31 | 962.628 | -8 | 7707.0815 | 7710.19 | 7707.0632 | 496149.66 |
| 1786-1808 | Ar-pUr-pCr-pCr-pCr-pCr-pAr-pGr-pAr-pCr-pAr-pCr-pUr-pGr-pGr | Cyclic phosphorylation | 23 | 1.71 | 100 | 1.2 | 37.4 | 917.743 | -8 | 7348.0015 | 7351.06 | 7347.9889 | 611951.69 |
| 1786-1808 | Ar-pUr-pCr-pCr-pCr-pCr-pAr-pGr-pAr-pCr-pAr-pCr-pUr-pGr-pGr | Cyclic phosphorylation | 23 | 0.65 | 100 | 1 | 37.42 | 815.771 | -9 | 7347.9937 | 7351.26 | 7347.9889 | 1405105.25 |
| 1786-1808 | Ar-pUr-pCr-pCr-pCr-pCr-pAr-pGr-pAr-pCr-pAr-pCr-pUr-pGr-pGr | Cyclic phosphorylation | 23 | 1.12 | 100 | 1 | 37.42 | 733.993 | -10 | 7347.9971 | 7351.27 | 7347.9889 | 1410379 |
| 1952-1977 | Gr-pCr-pGr-pGr-pGr-pUr-pGr-pUr-pAr-pCr-pUr-pCr-pCr-pAr-pC | Cyclic phosphorylation | 26 | 1.05 | 100 | 1.1 | 37.44 | 764.46 | -11 | 8415.1299 | 8418.99 | 8415.121 | 1404126 |
| 1952-1977 | Gr-pCr-pGr-pGr-pGr-pUr-pGr-pUr-pAr-pCr-pUr-pCr-pCr-pAr-pC | Cyclic phosphorylation | 26 | 1.17 | 100 | 1 | 37.51 | 840.907 | -10 | 8415.1309 | 8418.83 | 8415.121 | 2448240 |
| 1952-1977 | Gr-pCr-pGr-pGr-pGr-pUr-pGr-pUr-pAr-pCr-pUr-pCr-pCr-pAr-pC | Cyclic phosphorylation | 26 | 0.94 | 100 | 1 | 37.51 | 934.342 | -9 | 8415.1289 | 8418.9 | 8415.121 | 1752562 |
| 1952-1977 | Gr-pCr-pGr-pGr-pGr-pUr-pGr-pUr-pAr-pCr-pUr-pCr-pCr-pAr-pC | Cyclic phosphorylation | 26 | 1.17 | 100 | 1 | 37.51 | 1051.26 | -8 | 8415.1309 | 8419.02 | 8415.121 | 992291.94 |
| 4013-4036 | Ar-pCr-pAr-pCr-pCr-pUr-pCr-pCr-pCr-pAr-pAr-pGr-pCr-pAr-pCr- | Cyclic phosphorylation | 24 | 0.24 | 100 | 1.1 | 37.66 | 766.802 | -10 | 7675.0752 | 7678.41 | 7675.0734 | 1049856.25 |
| 4013-4036 | Ar-pCr-pAr-pCr-pCr-pUr-pCr-pCr-pCr-pAr-pAr-pGr-pCr-pAr-pCr- | Cyclic phosphorylation | 24 | -1.42 | 100 | 1 | 37.66 | 852.114 | -9 | 7675.0625 | 7678.41 | 7675.0734 | 876838.44 |
| 4013-4036 | Ar-pCr-pAr-pCr-pCr-pUr-pCr-pCr-pCr-pAr-pAr-pGr-pCr-pAr-pCr- | Cyclic phosphorylation | 24 | -0.21 | 100 | 1.6 | 37.68 | 958.754 | -8 | 7675.0718 | 7678.44 | 7675.0734 | 495511.69 |
| 1033-1058 | Ur-pGr-pCr-pGr-pGr-pUr-pUr-pCr-pCr-pCr-pAr-pAr-pUr-pA | Cyclic phosphorylation | 26 | 1.32 | 100 | 1 | 37.7 | 823.5 | -10 | 8242.0645 | 8245.74 | 8242.0535 | 714558.5 |
| 1033-1058 | Ur-pGr-pCr-pGr-pGr-pUr-pUr-pCr-pCr-pCr-pAr-pAr-pUr-pA | Cyclic phosphorylation | 26 | 1.8 | 100 | 1 | 37.72 | 915.223 | -9 | 8242.0684 | 8245.9 | 8242.0535 | 907088.88 |
| 3078-3102 | Ar-pCr-pCr-pCr-pAr-pGr-pCr-pAr-pGr-pCr-pUr-pGr-pAr-pUr-pCr | Cyclic phosphorylation | 25 | 3.12 | 100 | 1 | 38.08 | 804.506 | -10 | 8052.1357 | 8055.71 | 8052.1106 | 598183.12 |
| 3078-3102 | Ar-pCr-pCr-pCr-pAr-pGr-pCr-pAr-pGr-pCr-pUr-pGr-pAr-pUr-pCr | Cyclic phosphorylation | 25 | 3 | 100 | 1.1 | 38.1 | 894.007 | -9 | 8052.1348 | 8056.16 | 8052.1106 | 448939.91 |
| 4025-4048 | Cr-pAr-pCr-pGr-pCr-pAr-pGr-pCr-pAr-pAr-pUr-pGr-pCr-pAr-pGr | Cyclic phosphorylation | 24 | 1.33 | 99.8 | 1.1 | 38.22 | 966.756 | -8 | 7739.1011 | 7742.64 | 7739.0907 | 393609.59 |
| 4025-4048 | Cr-pAr-pCr-pGr-pCr-pAr-pGr-pCr-pAr-pAr-pUr-pGr-pCr-pAr-pGr | Cyclic phosphorylation | 24 | 2.03 | 99.8 | 1.1 | 38.26 | 859.115 | -9 | 7739.1064 | 7742.59 | 7739.0907 | 611399.88 |
| 3758-3785 | Ur-pUr-pGr-pCr-pAr-pUr-pGr-pAr-pCr-pAr-pGr-pCr-pUr-pG | Cyclic phosphorylation | 28 | 0.52 | 97.4 | 1.2 | 38.31 | 746.338 | -12 | 8965.127 | 8968.9 | 8965.1223 | 1587357.12 |
| 3758-3785 | Ur-pUr-pGr-pCr-pAr-pUr-pGr-pAr-pCr-pAr-pGr-pCr-pUr-pG | Cyclic phosphorylation | 28 | 3.03 | 97.4 | 1 | 38.33 | 814.37 | -11 | 8965.1494 | 8969.13 | 8965.1223 | 1276009.62 |
| 3758-3785 | Ur-pUr-pGr-pCr-pAr-pUr-pGr-pAr-pCr-pAr-pGr-pCr-pUr-pG | Cyclic phosphorylation | 28 | 6.08 | 97.4 | 1 | 38.33 | 895.908 | -10 | 8965.1768 | 8969.13 | 8965.1223 | 987964.56 |
| 2415-2438 | Ar-pUr-pCr-pCr-pAr-pCr-pAr-pAr-pGr-pAr-pCr-pCr-pCr-pUr | Cyclic phosphorylation | 24 | 0.84 | 90.7 | 1 | 38.34 | 759.096 | -10 | 7598.0195 | 7601.57 | 7598.0131 | 387274.19 |
| 2415-2438 | Ar-pUr-pCr-pCr-pAr-pCr-pAr-pAr-pGr-pAr-pCr-pCr-pCr-pUr | Cyclic phosphorylation | 24 | 0.59 | 90.7 | 1.1 | 38.36 | 843.552 | -9 | 7598.0176 | 7601.26 | 7598.0131 | 474950.09 |
| 3273-3298 | Ar-pAr-pUr-pUr-pUr-pCr-pAr-pCr-pCr-pAr-pCr-pCr-pGr-pCr-pUr | Cyclic phosphorylation | 26 | 1.68 | 100 | 1 | 38.38 | 1024.628 | -8 | 8201.0771 | 8204.96 | 8201.0634 | 659564.94 |
| 3273-3298 | Ar-pAr-pUr-pUr-pUr-pCr-pAr-pCr-pCr-pAr-pCr-pCr-pGr-pCr-pUr | Cyclic phosphorylation | 26 | 0.96 | 100 | 1 | 38.38 | 819.4 | -10 | 8201.0713 | 8204.74 | 8201.0634 | 2798413 |
| 3273-3298 | Ar-pAr-pUr-pUr-pUr-pCr-pAr-pCr-pCr-pAr-pCr-pCr-pGr-pCr-pUr | Cyclic phosphorylation | 26 | 1.92 | 100 | 1 | 38.39 | 744.909 | -11 | 8201.0791 | 8204.76 | 8201.0634 | 3189267.75 |
| 3273-3298 | Ar-pAr-pUr-pUr-pUr-pCr-pAr-pCr-pCr-pAr-pCr-pCr-pGr-pCr-pUr | Cyclic phosphorylation | 26 | 1.68 | 100 | 1 | 38.4 | 910.669 | -9 | 8201.0771 | 8204.91 | 8201.0634 | 1606909.62 |
| 3273-3298 | Ar-pAr-pUr-pUr-pUr-pCr-pAr-pCr-pCr-pAr-pCr-pCr-pGr-pCr-pUr | Cyclic phosphorylation | 26 | 1.56 | 100 | 1.9 | 38.43 | 682.75 | -12 | 8201.0762 | 8204.55 | 8201.0634 | 959433.69 |
| 3175-3200 | Ar-pCr-pUr-pUr-pUr-pUr-pGr-pCr-pGr-pGr-pCr-pAr-pAr-pGr-pG | Cyclic phosphorylation | 26 | 1.81 | 100 | 1 | 38.66 | 833.002 | -10 | 8337.0918 | 8340.82 | 8337.0767 | 1108981.5 |
| 3175-3200 | Ar-pCr-pUr-pUr-pUr-pUr-pGr-pCr-pGr-pGr-pCr-pAr-pAr-pGr-pG | Cyclic phosphorylation | 26 | 0.52 | 100 | 1.5 | 38.67 | 757.183 | -11 | 8337.0811 | 8340.9 | 8337.0767 | 1111309.12 |
| 3175-3200 | Ar-pCr-pUr-pUr-pUr-pUr-pGr-pCr-pGr-pGr-pCr-pAr-pAr-pGr-pG | Cyclic phosphorylation | 26 | 1.57 | 100 | 1.1 | 38.69 | 925.67 | -9 | 8337.0898 | 8340.96 | 8337.0767 | 664547 |
| 897-921 | Ar-pAr-pCr-pGr-pGr-pCr-pAr-pCr-pCr-pAr-pUr-pCr-pAr-pCr-pCr | Cyclic phosphorylation | 25 | 1.78 | 100 | 1 | 38.73 | 894.007 | -9 | 8052.125 | 8055.9 | 8052.1106 | 503788.03 |
| 1718-1742 | Cr-pAr-pCr-pAr-pAr-pGr-pAr-pCr-pAr-pGr-pUr-pUr-pCr-pCr-pUr | Cyclic phosphorylation | 25 | 0.82 | 100 | 1.1 | 38.74 | 886.336 | -9 | 7983.0732 | 7986.55 | 7983.0667 | 464336.12 |
| 4054-4079 | Cr-pCr-pUr-pAr-pGr-pCr-pCr-pAr-pCr-pAr-pCr-pCr-pCr-pCr-pCr- | Cyclic phosphorylation | 26 | 0.9 | 100 | 1.4 | 38.74 | 831.813 | -10 | 8324.1855 | 8327.76 | 8324.1781 | 649689.81 |
| 4054-4079 | Cr-pCr-pUr-pAr-pGr-pCr-pCr-pAr-pCr-pAr-pCr-pCr-pCr-pCr-pCr- | Cyclic phosphorylation | 26 | -0.63 | 100 | 1.2 | 38.75 | 924.236 | -9 | 8324.1729 | 8327.9 | 8324.1781 | 492917.47 |
| 897-921 | Ar-pAr-pCr-pGr-pGr-pCr-pAr-pCr-pCr-pAr-pUr-pCr-pAr-pCr-pCr | Cyclic phosphorylation | 25 | 1.72 | 100 | 1 | 38.75 | 804.505 | -10 | 8052.1245 | 8055.71 | 8052.1106 | 791863.38 |
| 1718-1742 | Cr-pAr-pCr-pAr-pAr-pGr-pAr-pCr-pAr-pGr-pUr-pUr-pCr-pCr-pUr | Cyclic phosphorylation | 25 | 2.29 | 100 | 1 | 38.76 | 797.601 | -10 | 7983.085 | 7986.61 | 7983.0667 | 609111 |
| 4054-4079 | Cr-pCr-pUr-pAr-pGr-pCr-pCr-pAr-pCr-pAr-pCr-pCr-pCr-pCr-pCr- | Cyclic phosphorylation | 26 | 0.78 | 100 | 1.5 | 38.76 | 756.102 | -11 | 8324.1846 | 8327.77 | 8324.1781 | 611045.75 |
| 2440-2465 | Ar-pCr-pUr-pUr-pCr-pGr-pGr-pCr-pGr-pCr-pUr-pUr-pCr-pA | Cyclic phosphorylation | 26 | 2.01 | 97 | 1.5 | 38.86 | 829.1 | -10 | 8298.0713 | 8301.75 | 8298.0546 | 480591.84 |
| 3299-3325 | Cr-pCr-pAr-pCr-pGr-pAr-pCr-pGr-pGr-pCr-pAr-pAr-pAr-pGr-pCr | Cyclic phosphorylation | 27 | 2.95 | 100 | 1 | 39.22 | 860.111 | -10 | 8608.1855 | 8612.16 | 8608.1602 | 1131513 |
| 3299-3325 | Cr-pCr-pAr-pCr-pGr-pAr-pCr-pGr-pGr-pCr-pAr-pAr-pAr-pGr-pCr | Cyclic phosphorylation | 27 | 1.93 | 100 | 1.1 | 39.22 | 955.79 | -9 | 8608.1768 | 8611.91 | 8608.1602 | 692424.06 |
| 3299-3325 | Cr-pCr-pAr-pCr-pGr-pAr-pCr-pGr-pGr-pCr-pAr-pAr-pAr-pGr-pCr | Cyclic phosphorylation | 27 | 2.61 | 100 | 1.9 | 39.22 | 1075.391 | -8 | 8608.1826 | 8611.93 | 8608.1602 | 327200.34 |
| 3299-3325 | Cr-pCr-pAr-pCr-pGr-pAr-pCr-pGr-pGr-pCr-pAr-pAr-pAr-pGr-pCr | Cyclic phosphorylation | 27 | 2.27 | 100 | 1.2 | 39.22 | 781.918 | -11 | 8608.1797 | 8612.04 | 8608.1602 | 1315074.38 |
| 303-330 | Cr-pUr-pGr-pCr-pCr-pCr-pUr-pUr-pCr-pAr-pAr-pCr-pGr-pAr-pCr | Cyclic phosphorylation | 28 | 0.52 | 100 | 1.4 | 39.23 | 895.807 | -10 | 8965.127 | 8969.19 | 8965.1223 | 393004.28 |
| 303-330 | Cr-pUr-pGr-pCr-pCr-pCr-pUr-pUr-pCr-pAr-pAr-pCr-pGr-pAr-pCr | Cyclic phosphorylation | 28 | 0.74 | 100 | 1.1 | 39.24 | 814.279 | -11 | 8965.1289 | 8968.91 | 8965.1223 | 571167.94 |
| 3393-3417 | Ar-pUr-pCr-pAr-pUr-pCr-pAr-pCr-pCr-pAr-pCr-pGr-pAr-pCr-pCr | Cyclic phosphorylation | 25 | 2.15 | 100 | 1 | 39.6 | 789.602 | -10 | 7902.0874 | 7905.46 | 7902.0704 | 1200142.88 |
| 3393-3417 | Ar-pUr-pCr-pAr-pUr-pCr-pAr-pCr-pCr-pAr-pCr-pGr-pAr-pCr-pCr | Cyclic phosphorylation | 25 | 2.4 | 100 | 1 | 39.64 | 877.336 | -9 | 7902.0894 | 7905.59 | 7902.0704 | 679985 |
| 3141-3167 | Ur-pCr-pUr-pGr-pAr-pGr-pUr-pGr-pUr-pGr-pUr-pCr-pUr-p( | Cyclic phosphorylation | 27 | 0.33 | 100 | 1.2 | 39.64 | 799.553 | -11 | 8802.1455 | 8806.13 | 8802.1426 | 945440.06 |
| 3141-3167 | Ur-pCr-pUr-pGr-pAr-pGr-pUr-pGr-pUr-pGr-pUr-pCr-pUr-p( | Cyclic phosphorylation | 27 | 1.88 | 100 | 1.1 | 39.66 | 879.509 | -10 | 8802.1592 | 8806.07 | 8802.1426 | 702834.56 |
| 853-881 | Ur-pGr-pGr-pGr-pCr-pUr-pAr-pCr-pCr-pUr-pGr-pCr-pAr-pGr-pC | Cyclic phosphorylation | 29 | 2 | 99.5 | 1.5 | 39.8 | 770.343 | -12 | 9253.2031 | 9257.18 | 9253.1846 | 459090.03 |
| 853-881 | Ur-pGr-pGr-pGr-pCr-pUr-pAr-pCr-pCr-pUr-pGr-pCr-pAr-pGr-pC | Cyclic phosphorylation | 29 | 1.79 | 99.5 | 1.5 | 39.81 | 840.466 | -11 | 9253.2012 | 9257.08 | 9253.1846 | 485583.28 |
| 3794-3822 | Ur-pUr-pGr-pUr-pAr-pGr-pCr-pUr-pGr-pUr-pGr-pGr-pCr-pAr-p( | Cyclic phosphorylation | 29 | 1.08 | 98.5 | 1.2 | 40.2 | 849.464 | -11 | 9351.1699 | 9355.31 | 9351.1599 | 521214.03 |
| 3112-3140 | Cr-pCr-pUr-pCr-pUr-pGr-pCr-pCr-pAr-pAr-pUr-pCr-pUr-pGr-pG | Cyclic phosphorylation | 29 | 1.06 | 100 | 1.5 | 40.25 | 1025.576 | -9 | 9235.2314 | 9239.78 | 9235.2217 | 399083.97 |
| 3112-3140 | Cr-pCr-pUr-pCr-pUr-pGr-pCr-pCr-pAr-pAr-pUr-pCr-pUr-pGr-pG | Cyclic phosphorylation | 29 | 3.49 | 100 | 1.1 | 40.26 | 922.917 | -10 | 9235.2539 | 9239.73 | 9235.2217 | 646501.94 |
| 3112-3140 | Cr-pCr-pUr-pCr-pUr-pGr-pCr-pCr-pAr-pAr-pUr-pCr-pUr-pGr-pG | Cyclic phosphorylation | 29 | 3.91 | 100 | 1 | 40.29 | 838.833 | -11 | 9235.2578 | 9239.36 | 9235.2217 | 950221 |
| 3112-3140 | Cr-pCr-pUr-pCr-pUr-pGr-pCr-pCr-pAr-pAr-pUr-pCr-pUr-pGr-pG | Cyclic phosphorylation | 29 | 1.27 | 100 | 1.3 | 40.32 | 768.93 | -12 | 9235.2334 | 9239.63 | 9235.2217 | 891662.19 |
| 3516-3540 | Ur-pAr-pCr-pUr-pUr-pAr-pAr-pGr-pAr-pAr-pGr-pCr-pCr-pAr-pC | Cyclic phosphorylation | 25 | 1.92 | 100 | 1.1 | 40.32 | 795.903 | -10 | 7966.103 | 7969.57 | 7966.0878 | 609449.88 |
| 3516-3540 | Ur-pAr-pCr-pUr-pUr-pAr-pAr-pGr-pAr-pAr-pGr-pCr-pCr-pAr-pC | Cyclic phosphorylation | 25 | 0.75 | 100 | 1 | 40.34 | 884.449 | -9 | 7966.0938 | 7969.62 | 7966.0878 | 595020.75 |
| 13-40 | Ur-pAr-pUr-pUr-pCr-pUr-pUr-pCr-pUr-pGr-pGr-pUr-pCr-pCr-pC | Cyclic phosphorylation | 28 | 0.51 | 100 | 1 | 40.53 | 807.733 | -11 | 8893.1309 | 8897.09 | 8893.1263 | 3060569 |
| 13-40 | Ur-pAr-pUr-pUr-pCr-pUr-pUr-pCr-pUr-pGr-pGr-pUr-pCr-pCr-pC | Cyclic phosphorylation | 28 | 2.71 | 100 | 1 | 40.55 | 987.453 | -9 | 8893.1504 | 8897.19 | 8893.1263 | 1138527.12 |
| 13-40 | Ur-pAr-pUr-pUr-pCr-pUr-pUr-pCr-pUr-pGr-pGr-pUr-pCr-pCr-pC | Cyclic phosphorylation | 28 | 1.39 | 100 | 1 | 40.56 | 888.707 | -10 | 8893.1387 | 8897.23 | 8893.1263 | 2326414.5 |
| 13-40 | Ur-pAr-pUr-pUr-pCr-pUr-pUr-pCr-pUr-pGr-pGr-pUr-pCr-pCr-pC | Cyclic phosphorylation | 28 | 1.06 | 100 | 1.3 | 40.57 | 740.338 | -12 | 8893.1357 | 8896.95 | 8893.1263 | 1620202.25 |
| 3110-3137 | Ar-pGr-pCr-pCr-pUr-pCr-pUr-pGr-pCr-pCr-pAr-pAr-pUr-pCr-pUr | Cyclic phosphorylation | 28 | 1.3 | 100 | 1.1 | 40.57 | 991.572 | -9 | 8929.208 | 8933.18 | 8929.1964 | 662318.94 |
| 1784-1808 | Ar-pCr-pAr-pUr-pCr-pCr-pCr-pCr-pAr-pGr-pAr-pCr-pAr-pCr-pUr | Cyclic phosphorylation | 25 | 1.77 | 99.2 | 1 | 40.57 | 890.671 | -9 | 8022.103 | 8025.8 | 8022.0888 | 569610.5 |
| 3110-3137 | Ar-pGr-pCr-pCr-pUr-pCr-pUr-pGr-pCr-pCr-pAr-pAr-pUr-pCr-pUr | Cyclic phosphorylation | 28 | -0.56 | 100 | 1.6 | 40.6 | 811.103 | -11 | 8929.1914 | 8933.12 | 8929.1964 | 768291.69 |
| 3110-3137 | Ar-pGr-pCr-pCr-pUr-pCr-pUr-pGr-pCr-pCr-pAr-pAr-pUr-pCr-pUr | Cyclic phosphorylation | 28 | 3.16 | 100 | 1 | 40.6 | 892.314 | -10 | 8929.2246 | 8933.23 | 8929.1964 | 897052.94 |
| 996-1023 | Ar-pGr-pCr-pAr-pGr-pCr-pAr-pAr-pCr-pUr-pUr-pCr-pCr-pGr-pGr | Cyclic phosphorylation | 28 | 2.21 | 100 | 1 | 40.93 | 814.56 | -11 | 8968.2383 | 8972.21 | 8968.2185 | 731375.5 |
| 996-1023 | Ar-pGr-pCr-pAr-pGr-pCr-pAr-pAr-pCr-pUr-pUr-pCr-pCr-pGr-pGr | Cyclic phosphorylation | 28 | 1.88 | 100 | 1.3 | 40.95 | 995.909 | -9 | 8968.2354 | 8972.36 | 8968.2185 | 405837.31 |
| 996-1023 | Ar-pGr-pCr-pAr-pGr-pCr-pAr-pAr-pCr-pUr-pUr-pCr-pCr-pGr-pGr | Cyclic phosphorylation | 28 | -0.08 | 100 | 1.2 | 40.95 | 896.117 | -10 | 8968.2178 | 8972.26 | 8968.2185 | 581938.19 |
| 2060-2084 | Ar-pAr-pUr-pCr-pUr-pGr-pCr-pGr-pCr-pCr-pAr-pGr-pCr-pUr-pAr | Cyclic phosphorylation | 25 | 2.03 | 100 | 1 | 41.02 | 890.562 | -9 | 8021.1211 | 8024.56 | 8021.1048 | 636498.81 |
| 2808-2831 | Ar-pCr-pAr-pCr-pAr-pGr-pAr-pGr-pCr-pUr-pGr-pAr-pUr-pCr-pC | Cyclic phosphorylation | 24 | 1.9 | 99.2 | 1.3 | 41.02 | 773.203 | -10 | 7739.1055 | 7742.74 | 7739.0907 | 597770.5 |
| 2060-2084 | Ar-pAr-pUr-pCr-pUr-pGr-pCr-pGr-pCr-pCr-pAr-pGr-pCr-pUr-pAr | Cyclic phosphorylation | 25 | 2.03 | 100 | 1 | 41.02 | 801.305 | -10 | 8021.1211 | 8024.63 | 8021.1048 | 1016321.38 |
| 2808-2831 | Ar-pCr-pAr-pCr-pAr-pGr-pAr-pGr-pCr-pUr-pGr-pAr-pUr-pCr-pC | Cyclic phosphorylation | 24 | 2.22 | 99.2 | 1 | 41.03 | 859.339 | -9 | 7739.1079 | 7742.74 | 7739.0907 | 618276.81 |
| 233-261 | Cr-pAr-pAr-pCr-pGr-pUr-pGr-pAr-pCr-pCr-pUr-pGr-pGr-pUr-pU | Cyclic phosphorylation | 29 | 0.11 | 99.8 | 1.1 | 41.05 | 768.929 | -12 | 9235.2227 | 9239.47 | 9235.2217 | 658734.12 |
| 233-261 | Cr-pAr-pAr-pCr-pGr-pUr-pGr-pAr-pCr-pCr-pUr-pGr-pGr-pUr-pU | Cyclic phosphorylation | 29 | 0.95 | 99.8 | 1 | 41.05 | 838.924 | -11 | 9235.2305 | 9239.28 | 9235.2217 | 728249.38 |
| 233-261 | Cr-pAr-pAr-pCr-pGr-pUr-pGr-pAr-pCr-pCr-pUr-pGr-pGr-pUr-pU | Cyclic phosphorylation | 29 | 1.16 | 99.8 | 1 | 41.07 | 922.817 | -10 | 9235.2324 | 9239.31 | 9235.2217 | 478456.5 |
| 2492-2519 | Cr-pAr-pGr-pCr-pGr-pGr-pAr-pGr-pCr-pUr-pUr-pCr-pAr-pU | Cyclic phosphorylation | 28 | 0.91 | 99.2 | 1.1 | 41.09 | 822.103 | -11 | 9050.207 | 9054.37 | 9050.1988 | 647362.56 |
| 2492-2519 | Cr-pAr-pGr-pCr-pGr-pGr-pAr-pGr-pCr-pUr-pUr-pCr-pAr-pU | Cyclic phosphorylation | 28 | -0.06 | 99.2 | 1.2 | 41.09 | 904.315 | -10 | 9050.1982 | 9054.28 | 9050.1988 | 423823.72 |
| 2191-2219 | Cr-pUr-pAr-pUr-pCr-pCr-pCr-pCr-pAr-pCr-pCr-pAr-pAr-pCr-pUr | Cyclic phosphorylation | 29 | 2.75 | 100 | 1.4 | 41.23 | 761.011 | -12 | 9140.2236 | 9144.31 | 9140.1985 | 594723.62 |
| 2191-2219 | Cr-pUr-pAr-pUr-pCr-pCr-pCr-pCr-pAr-pCr-pCr-pAr-pAr-pCr-pUr | Cyclic phosphorylation | 29 | -2.59 | 100 | 1.9 | 41.24 | 913.315 | -10 | 9140.1748 | 9143.06 | 9140.1985 | 314573.56 |
| 2191-2219 | Cr-pUr-pAr-pUr-pCr-pCr-pCr-pCr-pAr-pCr-pCr-pAr-pAr-pCr-pUr | Cyclic phosphorylation | 29 | 3.82 | 100 | 1.5 | 41.24 | 830.195 | -11 | 9140.2334 | 9144.38 | 9140.1985 | 449707.81 |
| 3687-3717 | Ur-pGr-pGr-pCr-pCr-pCr-pUr-pGr-pGr-pUr-pGr-pAr-pUr-pC | Cyclic phosphorylation | 31 | 4.29 | 90.7 | 1.2 | 41.49 | 827.596 | -12 | 9938.2578 | 9942.66 | 9938.2152 | 469341.91 |
| 3687-3717 | Ur-pGr-pGr-pCr-pCr-pCr-pUr-pGr-pGr-pUr-pGr-pAr-pUr-pC | Cyclic phosphorylation | 31 | 2.81 | 90.7 | 1.1 | 41.54 | 902.832 | -11 | 9938.2432 | 9942.56 | 9938.2152 | 496841.12 |
| 531-558 | Ur-pAr-pCr-pAr-pGr-pCr-pAr-pGr-pCr-pCr-pAr-pCr-pAr-pCr | Cyclic phosphorylation | 28 | 0.57 | 100 | 1 | 41.61 | 892.915 | -10 | 8937.2178 | 8941.13 | 8937.2127 | 634223.81 |
| 531-558 | Ur-pAr-pCr-pAr-pGr-pCr-pAr-pGr-pCr-pCr-pAr-pCr-pAr-pCr | Cyclic phosphorylation | 28 | 1.01 | 100 | 1 | 41.63 | 811.832 | -11 | 8937.2217 | 8941.14 | 8937.2127 | 791685.5 |
| 3078-3104 | Ar-pCr-pCr-pCr-pAr-pGr-pCr-pAr-pGr-pCr-pUr-pGr-pAr-pUr-pCr | Cyclic phosphorylation | 27 | -0.52 | 100 | 1.2 | 41.87 | 969.018 | -9 | 8726.2061 | 8730.16 | 8726.2106 | 485936.16 |
| 3078-3104 | Ar-pCr-pCr-pCr-pAr-pGr-pCr-pAr-pGr-pCr-pUr-pGr-pAr-pUr-pCr | Cyclic phosphorylation | 27 | 1.27 | 100 | 1.1 | 41.87 | 792.65 | -11 | 8726.2217 | 8730.15 | 8726.2106 | 889766.06 |
| 3078-3104 | Ar-pCr-pCr-pCr-pAr-pGr-pCr-pAr-pGr-pCr-pUr-pGr-pAr-pUr-pCr | Cyclic phosphorylation | 27 | 2.61 | 100 | 1 | 41.89 | 872.016 | -10 | 8726.2334 | 8730.05 | 8726.2106 | 773842.12 |
| 948-975 | Ar-pCr-pAr-pAr-pAr-pGr-pUr-pGr-pCr-pAr-pCr-pCr-pCr-pUr-pGr | Cyclic phosphorylation | 28 | 2.68 | 100 | 1.5 | 42.24 | 744.093 | -12 | 8938.2207 | 8942.27 | 8938.1967 | 1545480.25 |
| 948-975 | Ar-pCr-pAr-pAr-pAr-pGr-pUr-pGr-pCr-pAr-pCr-pCr-pCr-pUr-pGr | Cyclic phosphorylation | 28 | 1.16 | 100 | 1 | 42.27 | 811.83 | -11 | 8938.207 | 8942.19 | 8938.1967 | 3376480.5 |
| 948-975 | Ar-pCr-pAr-pAr-pAr-pGr-pUr-pGr-pCr-pAr-pCr-pCr-pCr-pUr-pGr | Cyclic phosphorylation | 28 | 1.48 | 100 | 1 | 42.29 | 893.214 | -10 | 8938.21 | 8942.26 | 8938.1967 | 2393531 |

| | | | | | | | | | | | | | |
|---|---|---|---|---|---|---|---|---|---|---|---|---|---|
| 948-975 | Ar-pCr-pAr-pAr-pAr-pGr-pUr-pGr-pCr-pAr-pCr-pCr-pCr-pUr-pGr | Cyclic phosphorylation | 28 | 1.37 | 100 | 1 | 42.3 | 992.572 | -9 | 8938.209 | 8942.25 | 8938.1967 | 1231002.88 |
| 948-975 | Ar-pCr-pAr-pAr-pAr-pGr-pUr-pGr-pCr-pAr-pCr-pCr-pCr-pUr-pGr | Cyclic phosphorylation | 28 | 1.26 | 100 | 1.3 | 42.3 | 1116.769 | -8 | 8938.208 | 8942.35 | 8938.1967 | 564566 |
| 4097-4119 | Cr-pAr-pAr-pUr-pAr-pAr-pAr-pCr-pGr-pAr-pAr-pAr-pGr-pUr-pU | Cyclic phosphorylation | 23 | 1.39 | 100 | 1 | 42.53 | 824.774 | -9 | 7429.0332 | 7432.33 | 7429.0229 | 1920754 |
| 4097-4119 | Cr-pAr-pAr-pUr-pAr-pAr-pAr-pCr-pGr-pAr-pAr-pAr-pGr-pUr-pU | Cyclic phosphorylation | 23 | 1.85 | 100 | 1 | 42.55 | 927.872 | -8 | 7429.0366 | 7432.42 | 7429.0229 | 1239658 |
| 4097-4119 | Cr-pAr-pAr-pUr-pAr-pAr-pAr-pCr-pGr-pAr-pAr-pAr-pGr-pUr-pU | Cyclic phosphorylation | 23 | 1.39 | 100 | 1.5 | 42.55 | 742.197 | -10 | 7429.0332 | 7432.25 | 7429.0229 | 1018354.69 |
| 4097-4119 | Cr-pAr-pAr-pUr-pAr-pAr-pAr-pCr-pGr-pAr-pAr-pAr-pGr-pUr-pU | Cyclic phosphorylation | 23 | 0.73 | 100 | 1.2 | 42.57 | 1060.713 | -7 | 7429.0283 | 7432.54 | 7429.0229 | 431067.53 |
| 2085-2110 | Ar-pCr-pAr-pAr-pAr-pCr-pAr-pGr-pCr-pCr-pCr-pUr-pCr-pGr-pGr | Cyclic phosphorylation | 26 | 0.24 | 100 | 1 | 42.8 | 844.516 | -10 | 8452.2148 | 8455.96 | 8452.2128 | 749989.62 |
| 2085-2110 | Ar-pCr-pAr-pAr-pAr-pCr-pAr-pGr-pCr-pCr-pCr-pUr-pCr-pGr-pGr | Cyclic phosphorylation | 26 | 0.93 | 100 | 1.2 | 42.81 | 938.686 | -9 | 8452.2207 | 8456.17 | 8452.2128 | 434991.41 |
| 596-622 | Cr-pAr-pAr-pGr-pCr-pAr-pGr-pGr-pGr-pCr-pAr-pAr-pCr-pUr-pU | Cyclic phosphorylation | 27 | 1.46 | 100 | 1 | 42.87 | 969.907 | -9 | 8735.2236 | 8739.14 | 8735.2109 | 741558.5 |
| 596-622 | Cr-pAr-pAr-pGr-pCr-pAr-pGr-pGr-pGr-pCr-pAr-pAr-pCr-pUr-pU | Cyclic phosphorylation | 27 | 0.67 | 100 | 1 | 42.88 | 872.915 | -10 | 8735.2168 | 8739.09 | 8735.2109 | 1291137.88 |
| 596-622 | Cr-pAr-pAr-pGr-pCr-pAr-pGr-pGr-pGr-pCr-pAr-pAr-pCr-pUr-pU | Cyclic phosphorylation | 27 | 1.9 | 100 | 1.6 | 42.92 | 793.378 | -11 | 8735.2275 | 8739.16 | 8735.2109 | 950542.75 |
| 3042-3068 | Ar-pUr-pCr-pAr-pCr-pAr-pGr-pGr-pCr-pAr-pGr-pAr-pCr-pUr-pG | Cyclic phosphorylation | 27 | -0.95 | 99.7 | 1.2 | 42.99 | 791.194 | -11 | 8711.1914 | 8715.26 | 8711.1997 | 936317.88 |
| 3042-3068 | Ar-pUr-pCr-pAr-pCr-pAr-pGr-pGr-pCr-pAr-pGr-pAr-pCr-pUr-pG | Cyclic phosphorylation | 27 | 3.09 | 99.7 | 1 | 43.04 | 870.414 | -10 | 8711.2266 | 8714.95 | 8711.1997 | 858557.38 |
| 3145-3171 | Ar-pGr-pUr-pGr-pUr-pGr-pUr-pGr-pCr-pUr-pGr-pGr-pGr-pCr-p( | Cyclic phosphorylation | 27 | 1.2 | 100 | 1.3 | 43.18 | 987.129 | -9 | 8888.2139 | 8892.28 | 8888.2032 | 583394 |
| 3145-3171 | Ar-pGr-pUr-pGr-pUr-pGr-pUr-pGr-pCr-pUr-pGr-pGr-pGr-pCr-p( | Cyclic phosphorylation | 27 | 1.31 | 100 | 1 | 43.18 | 888.215 | -10 | 8888.2148 | 8892.25 | 8888.2032 | 919197.12 |
| 495-517 | Ar-pAr-pCr-pAr-pAr-pCr-pAr-pAr-pGr-pAr-pGr-pCr-pUr-pGr-pG | Cyclic phosphorylation | 23 | 1.71 | 99.9 | 1 | 43.3 | 839.559 | -9 | 7562.0972 | 7565.57 | 7562.0842 | 458460.31 |
| 4054-4082 | Cr-pCr-pUr-pAr-pGr-pCr-pCr-pAr-pCr-pAr-pCr-pCr-pCr-pCr-pCr- | Cyclic phosphorylation | 29 | -9.97 | 95.4 | 1.9 | 43.4 | 774.688 | -12 | 9303.2266 | 9307.21 | 9303.3193 | 691279.44 |
| 4054-4082 | Cr-pCr-pUr-pAr-pGr-pCr-pCr-pAr-pCr-pAr-pCr-pCr-pCr-pCr-pCr- | Cyclic phosphorylation | 29 | -8.81 | 95.4 | 1.5 | 43.42 | 845.115 | -11 | 9303.2373 | 9307.15 | 9303.3193 | 716587.25 |
| 1305-1332 | Ar-pUr-pCr-pGr-pCr-pCr-pGr-pAr-pCr-pUr-pAr-pCr-pAr-pAr-pCr | Cyclic phosphorylation | 28 | 1.33 | 96.6 | 1.3 | 43.88 | 811.832 | -11 | 8937.2246 | 8941.25 | 8937.2127 | 512339.5 |
| 3237-3267 | Ur-pUr-pUr-pCr-pUr-pGr-pCr-pAr-pCr-pGr-pUr-pGr-pAr-pCr-pA | Cyclic phosphorylation | 31 | 1.35 | 100 | 1.1 | 44.24 | 988.32 | -10 | 9889.2598 | 9893.76 | 9889.2465 | 502264.81 |
| 3237-3267 | Ur-pUr-pUr-pCr-pUr-pGr-pCr-pAr-pCr-pGr-pUr-pGr-pAr-pCr-pA | Cyclic phosphorylation | 31 | 1.74 | 100 | 1.3 | 44.25 | 760.013 | -13 | 9889.2637 | 9893.62 | 9889.2465 | 858065.12 |
| 3237-3267 | Ur-pUr-pUr-pCr-pUr-pGr-pCr-pAr-pCr-pGr-pUr-pGr-pAr-pCr-pA | Cyclic phosphorylation | 31 | 1.64 | 100 | 1.1 | 44.26 | 823.431 | -12 | 9889.2627 | 9893.67 | 9889.2465 | 1074509.25 |
| 3237-3267 | Ur-pUr-pUr-pCr-pUr-pGr-pCr-pAr-pCr-pGr-pUr-pGr-pAr-pCr-pA | Cyclic phosphorylation | 31 | 1.35 | 100 | 1.1 | 44.26 | 898.381 | -11 | 9889.2598 | 9893.69 | 9889.2465 | 954616.5 |
| 3273-3303 | Ar-pAr-pUr-pUr-pUr-pCr-pAr-pCr-pCr-pAr-pCr-pCr-pGr-pCr-pUr | Cyclic phosphorylation | 31 | 2.78 | 100 | 1.1 | 44.58 | 815.185 | -12 | 9790.3145 | 9794.5 | 9790.2872 | 2019066.12 |
| 3273-3303 | Ar-pAr-pUr-pUr-pUr-pCr-pAr-pCr-pCr-pAr-pCr-pCr-pGr-pCr-pUr | Cyclic phosphorylation | 31 | 2.88 | 100 | 1.6 | 44.59 | 752.324 | -13 | 9790.3154 | 9794.4 | 9790.2872 | 1076824.75 |
| 3273-3303 | Ar-pAr-pUr-pUr-pUr-pCr-pAr-pCr-pCr-pAr-pCr-pCr-pGr-pCr-pUr | Cyclic phosphorylation | 31 | 2.98 | 100 | 1 | 44.59 | 889.384 | -11 | 9790.3164 | 9794.88 | 9790.2872 | 1621833.88 |
| 3273-3303 | Ar-pAr-pUr-pUr-pUr-pCr-pAr-pCr-pCr-pAr-pCr-pCr-pGr-pCr-pUr | Cyclic phosphorylation | 31 | 2.19 | 100 | 1.1 | 44.61 | 978.423 | -10 | 9790.3086 | 9795.11 | 9790.2872 | 861487.94 |
| 1001-1032 | Cr-pAr-pAr-pCr-pUr-pUr-pCr-pCr-pGr-pGr-pGr-pUr-pGr-pCr-pA | Cyclic phosphorylation | 32 | 1.18 | 98.3 | 1.4 | 44.92 | 785.023 | -13 | 10214.375 | 10218.93 | 10214.3629 | 1087518.38 |
| 1001-1032 | Cr-pAr-pAr-pCr-pUr-pUr-pCr-pCr-pGr-pGr-pGr-pUr-pGr-pCr-pA | Cyclic phosphorylation | 32 | 1.66 | 98.3 | 1 | 44.92 | 927.845 | -11 | 10214.3799 | 10218.92 | 10214.3629 | 881709.38 |
| 1001-1032 | Cr-pAr-pAr-pCr-pUr-pUr-pCr-pCr-pGr-pGr-pGr-pUr-pGr-pCr-pA | Cyclic phosphorylation | 32 | 0.51 | 98.3 | 1 | 44.94 | 850.525 | -12 | 10214.3682 | 10218.9 | 10214.3629 | 1248787.62 |
| 1001-1032 | Cr-pAr-pAr-pCr-pUr-pUr-pCr-pCr-pGr-pGr-pGr-pUr-pGr-pCr-pA | Cyclic phosphorylation | 32 | 0.61 | 98.3 | 1.2 | 44.94 | 1020.831 | -10 | 10214.3691 | 10218.95 | 10214.3629 | 463563.91 |
| 596-624 | Cr-pAr-pAr-pGr-pCr-pAr-pGr-pGr-pGr-pCr-pAr-pAr-pCr-pUr-pU | Cyclic phosphorylation | 29 | 1.79 | 100 | 1.3 | 45.2 | 781.519 | -12 | 9385.3164 | 9389.86 | 9385.2996 | 1743794.88 |
| 596-624 | Cr-pAr-pAr-pGr-pCr-pAr-pGr-pGr-pGr-pCr-pAr-pAr-pCr-pUr-pU | Cyclic phosphorylation | 29 | -0.29 | 100 | 1 | 45.24 | 937.924 | -10 | 9385.2969 | 9389.68 | 9385.2996 | 2545449.25 |
| 596-624 | Cr-pAr-pAr-pGr-pCr-pAr-pGr-pGr-pGr-pCr-pAr-pAr-pCr-pUr-pU | Cyclic phosphorylation | 29 | -0.09 | 100 | 1 | 45.24 | 1042.361 | -9 | 9385.2988 | 9389.73 | 9385.2996 | 1093979.12 |
| 596-624 | Cr-pAr-pAr-pGr-pCr-pAr-pGr-pGr-pGr-pCr-pAr-pAr-pCr-pUr-pU | Cyclic phosphorylation | 29 | 5.12 | 100 | 1 | 45.25 | 852.658 | -11 | 9385.3477 | 9389.62 | 9385.2996 | 3693520 |
| 198-232 | Cr-pAr-pCr-pUr-pCr-pUr-pAr-pCr-pCr-pCr-pAr-pGr-pGr-pAr-pCr | Cyclic phosphorylation | 35 | 2.43 | 100 | 1 | 45.32 | 846.021 | -13 | 11007.3828 | 11012.33 | 11007.3561 | 4395774 |
| 198-232 | Cr-pAr-pCr-pUr-pCr-pUr-pAr-pCr-pCr-pCr-pAr-pGr-pGr-pAr-pCr | Cyclic phosphorylation | 35 | 2.78 | 100 | 1 | 45.34 | 785.519 | -14 | 11007.3867 | 11012.27 | 11007.3561 | 1529863.88 |
| 198-232 | Cr-pAr-pCr-pUr-pCr-pUr-pAr-pCr-pCr-pCr-pAr-pGr-pGr-pAr-pCr | Cyclic phosphorylation | 35 | 0.56 | 100 | 1 | 45.34 | 1000.118 | -11 | 11007.3623 | 11012.2 | 11007.3561 | 3311526.5 |
| 198-232 | Cr-pAr-pCr-pUr-pCr-pUr-pAr-pCr-pCr-pCr-pAr-pGr-pGr-pAr-pCr | Cyclic phosphorylation | 35 | 2.6 | 100 | 1 | 45.34 | 916.691 | -12 | 11007.3848 | 11012.44 | 11007.3561 | 4973651 |
| 3328-3358 | Ar-pAr-pGr-pGr-pCr-pGr-pUr-pGr-pUr-pUr-pCr-pGr-pUr-pGr-pU | Cyclic phosphorylation | 31 | -4.18 | 99.9 | 1 | 45.36 | 905.384 | -11 | 9967.249 | 9971.81 | 9967.2907 | 1803506.75 |
| 198-232 | Cr-pAr-pCr-pUr-pCr-pUr-pAr-pCr-pCr-pCr-pAr-pGr-pGr-pAr-pCr | Cyclic phosphorylation | 35 | 1.89 | 100 | 1.2 | 45.36 | 1100.231 | -10 | 11007.377 | 11012.14 | 11007.3561 | 1421784.25 |
| 3328-3358 | Ar-pAr-pGr-pGr-pCr-pGr-pUr-pGr-pUr-pUr-pCr-pGr-pUr-pGr-pU | Cyclic phosphorylation | 31 | 1.4 | 99.9 | 1 | 45.37 | 766.017 | -13 | 9967.3047 | 9971.91 | 9967.2907 | 2698729.25 |
| 3328-3358 | Ar-pAr-pGr-pGr-pCr-pGr-pUr-pGr-pUr-pUr-pCr-pGr-pUr-pGr-pU | Cyclic phosphorylation | 31 | -0.56 | 99.9 | 1 | 45.37 | 829.935 | -12 | 9967.2852 | 9971.66 | 9967.2907 | 2552742 |
| 3328-3358 | Ar-pAr-pGr-pGr-pCr-pGr-pUr-pGr-pUr-pUr-pCr-pGr-pUr-pGr-pU | Cyclic phosphorylation | 31 | -0.07 | 99.9 | 1.2 | 45.37 | 996.124 | -10 | 9967.29 | 9971.71 | 9967.2907 | 940309.69 |
| 1743-1773 | Cr-pAr-pGr-pUr-pUr-pUr-pGr-pGr-pCr-pCr-pGr-pGr-pGr-pAr-pU | Cyclic phosphorylation | 31 | 4.44 | 99.8 | 1.6 | 45.62 | 998.526 | -10 | 9990.3623 | 9994.69 | 9990.3179 | 488803.5 |
| 1743-1773 | Cr-pAr-pGr-pUr-pUr-pUr-pGr-pGr-pCr-pCr-pGr-pGr-pGr-pAr-pU | Cyclic phosphorylation | 31 | 0.43 | 99.8 | 1.2 | 45.62 | 767.788 | -13 | 9990.3223 | 9994.6 | 9990.3179 | 922946.19 |
| 1743-1773 | Cr-pAr-pGr-pUr-pUr-pUr-pGr-pGr-pCr-pCr-pGr-pGr-pGr-pAr-pU | Cyclic phosphorylation | 31 | 1.22 | 99.8 | 1 | 45.63 | 831.937 | -12 | 9990.3301 | 9994.87 | 9990.3179 | 1487913.62 |
| 1743-1773 | Cr-pAr-pGr-pUr-pUr-pUr-pGr-pGr-pCr-pCr-pGr-pGr-pGr-pAr-pU | Cyclic phosphorylation | 31 | 0.53 | 99.8 | 1 | 45.65 | 907.569 | -11 | 9990.3232 | 9994.83 | 9990.3179 | 911207.94 |
| 3145-3173 | Ar-pGr-pUr-pGr-pUr-pGr-pUr-pGr-pCr-pUr-pGr-pGr-pGr-pCr-p( | Cyclic phosphorylation | 29 | -7.74 | 99.6 | 1 | 45.69 | 953.322 | -10 | 9539.2021 | 9543.38 | 9539.2759 | 580422.25 |
| 3145-3173 | Ar-pGr-pUr-pGr-pUr-pGr-pUr-pGr-pCr-pUr-pGr-pGr-pGr-pCr-p( | Cyclic phosphorylation | 29 | -1.29 | 99.6 | 1 | 45.7 | 866.474 | -11 | 9539.2637 | 9543.65 | 9539.2759 | 791227.81 |
| 4054-4084 | Cr-pCr-pUr-pAr-pGr-pCr-pCr-pAr-pCr-pAr-pCr-pCr-pCr-pCr-pCr- | Cyclic phosphorylation | 31 | 2.02 | 99.2 | 1.5 | 45.91 | 828.778 | -12 | 9954.4121 | 9958.63 | 9954.392 | 397808 |
| 3141-3171 | Ur-pCr-pUr-pGr-pAr-pGr-pUr-pGr-pUr-pGr-pUr-pGr-pCr-pUr-p( | Cyclic phosphorylation | 31 | 0.7 | 99.9 | 1 | 46.24 | 922.208 | -11 | 10150.3496 | 10154.86 | 10150.3425 | 941698.38 |
| 1346-1376 | Cr-pUr-pGr-pUr-pGr-pUr-pGr-pAr-pUr-pUr-pGr-pCr-pCr-pUr-p( | Cyclic phosphorylation | 31 | 2.31 | 99.8 | 1.1 | 46.24 | 830.52 | -12 | 9975.3301 | 9979.76 | 9975.307 | 993553.38 |
| 1346-1376 | Cr-pUr-pGr-pUr-pGr-pUr-pGr-pAr-pUr-pUr-pGr-pCr-pCr-pUr-p( | Cyclic phosphorylation | 31 | 1.92 | 99.8 | 1 | 46.26 | 766.71 | -13 | 9975.3262 | 9979.82 | 9975.307 | 985342.88 |
| 116-145 | Ar-pAr-pCr-pAr-pCr-pAr-pGr-pCr-pUr-pGr-pCr-pCr-pUr-pCr-pCr | Cyclic phosphorylation | 30 | 2 | 100 | 1 | 46.36 | 954.828 | -10 | 9554.3467 | 9558.81 | 9554.3276 | 1293795.25 |
| 116-145 | Ar-pAr-pCr-pAr-pCr-pAr-pGr-pCr-pUr-pGr-pCr-pCr-pUr-pCr-pCr | Cyclic phosphorylation | 30 | 2.92 | 100 | 1 | 46.38 | 867.842 | -11 | 9554.3555 | 9558.49 | 9554.3276 | 1365208.12 |
| 116-145 | Ar-pAr-pCr-pAr-pCr-pAr-pGr-pCr-pUr-pGr-pCr-pCr-pUr-pCr-pCr | Cyclic phosphorylation | 30 | 1.08 | 100 | 1.2 | 46.38 | 1061.031 | -9 | 9554.3379 | 9558.78 | 9554.3276 | 683270.25 |
| 116-145 | Ar-pAr-pCr-pAr-pCr-pAr-pGr-pCr-pUr-pGr-pCr-pCr-pUr-pCr-pCr | Cyclic phosphorylation | 30 | 1.29 | 100 | 1.3 | 46.41 | 795.438 | -12 | 9554.3398 | 9558.63 | 9554.3276 | 668236.06 |
| 3393-3423 | Ar-pUr-pCr-pAr-pCr-pUr-pAr-pCr-pCr-pAr-pCr-pCr-pGr-pAr-pCr | Cyclic phosphorylation | 31 | 1.27 | 100 | 1.2 | 46.62 | 817.352 | -12 | 9815.2949 | 9819.74 | 9815.2824 | 493466.31 |
| 3453-3483 | Ar-pAr-pCr-pAr-pAr-pUr-pAr-pCr-pCr-pGr-pUr-pGr-pUr-pAr-pCr | Cyclic phosphorylation | 31 | 2.41 | 100 | 1 | 46.87 | 823.856 | -12 | 9893.3506 | 9897.91 | 9893.3267 | 1091432 |
| 3453-3483 | Ar-pAr-pCr-pAr-pAr-pUr-pAr-pCr-pCr-pGr-pUr-pGr-pUr-pAr-pCr | Cyclic phosphorylation | 31 | 2.91 | 100 | 1 | 46.9 | 898.752 | -11 | 9893.3555 | 9897.7 | 9893.3267 | 1388193.12 |
| 3453-3483 | Ar-pAr-pCr-pAr-pAr-pUr-pAr-pCr-pCr-pGr-pUr-pGr-pUr-pAr-pCr | Cyclic phosphorylation | 31 | 2.41 | 100 | 1 | 46.92 | 988.627 | -10 | 9893.3506 | 9897.62 | 9893.3267 | 916172.75 |
| 461-494 | Cr-pAr-pAr-pCr-pGr-pAr-pCr-pCr-pCr-pCr-pUr-pUr-pCr-pCr-pUr | Cyclic phosphorylation | 34 | 1.81 | 99 | 1.1 | 47.04 | 771.454 | -14 | 10809.4541 | 10814.36 | 10809.4346 | 3787548.25 |
| 461-494 | Cr-pAr-pAr-pCr-pGr-pAr-pCr-pCr-pCr-pCr-pUr-pUr-pCr-pCr-pUr | Cyclic phosphorylation | 34 | 1.17 | 99 | 1 | 47.04 | 900.114 | -12 | 10809.4473 | 10814.29 | 10809.4346 | 2709876.25 |
| 461-494 | Cr-pAr-pAr-pCr-pGr-pAr-pCr-pCr-pCr-pCr-pUr-pUr-pCr-pCr-pUr | Cyclic phosphorylation | 34 | -10.12 | 99 | 1.2 | 47.04 | 1080.238 | -10 | 10809.3252 | 10814.28 | 10809.4346 | 1218524.5 |
| 461-494 | Cr-pAr-pAr-pCr-pGr-pAr-pCr-pCr-pCr-pCr-pUr-pUr-pCr-pCr-pUr | Cyclic phosphorylation | 34 | 1.72 | 99 | 1 | 47.04 | 982.034 | -11 | 10809.4531 | 10814.02 | 10809.4346 | 2374411 |
| 461-494 | Cr-pAr-pAr-pCr-pGr-pAr-pCr-pCr-pCr-pCr-pUr-pUr-pCr-pCr-pUr | Cyclic phosphorylation | 34 | 1.17 | 99 | 1 | 47.06 | 830.797 | -13 | 10809.4473 | 10814.22 | 10809.4346 | 4823511.5 |
| 1671-1701 | Ar-pAr-pCr-pUr-pUr-pCr-pAr-pAr-pCr-pUr-pUr-pCr-pAr-pAr-pCr | Cyclic phosphorylation | 31 | 3.3 | 100 | 1.1 | 47.23 | 1098.698 | -9 | 9893.3594 | 9897.65 | 9893.3267 | 794780.94 |
| 1671-1701 | Ar-pAr-pCr-pUr-pUr-pCr-pAr-pAr-pCr-pUr-pUr-pCr-pAr-pAr-pCr | Cyclic phosphorylation | 31 | 2.12 | 100 | 1 | 47.25 | 988.728 | -10 | 9893.3477 | 9897.7 | 9893.3267 | 1663696.75 |
| 1671-1701 | Ar-pAr-pCr-pUr-pUr-pCr-pAr-pAr-pCr-pUr-pUr-pCr-pAr-pAr-pCr | Cyclic phosphorylation | 31 | 0.83 | 100 | 1 | 47.26 | 898.752 | -11 | 9893.335 | 9897.83 | 9893.3267 | 2405697.75 |
| 233-267 | Cr-pAr-pAr-pCr-pGr-pUr-pGr-pAr-pCr-pCr-pUr-pGr-pGr-pUr-pU | Cyclic phosphorylation | 35 | -0.22 | 91.7 | 1.4 | 47.26 | 928.282 | -12 | 11147.4473 | 11152.12 | 11147.4497 | 686007.25 |
| 233-267 | Cr-pAr-pAr-pCr-pGr-pUr-pGr-pAr-pCr-pCr-pUr-pGr-pGr-pUr-pU | Cyclic phosphorylation | 35 | 1.18 | 91.7 | 1.1 | 47.26 | 856.798 | -13 | 11147.4629 | 11152.12 | 11147.4497 | 1029505.62 |
| 1671-1701 | Ar-pAr-pCr-pUr-pUr-pCr-pAr-pAr-pCr-pUr-pUr-pCr-pAr-pAr-pCr | Cyclic phosphorylation | 31 | 1.03 | 100 | 1 | 47.27 | 823.772 | -12 | 9893.3369 | 9897.66 | 9893.3267 | 2129130.75 |
| 233-267 | Cr-pAr-pAr-pCr-pGr-pUr-pGr-pAr-pCr-pCr-pUr-pGr-pGr-pUr-pU | Cyclic phosphorylation | 35 | 1.8 | 91.7 | 1.1 | 47.27 | 795.67 | -14 | 11147.4697 | 11152.46 | 11147.4497 | 1206467.75 |
| 414-441 | Ar-pAr-pAr-pAr-pAr-pGr-pCr-pGr-pCr-pCr-pAr-pCr-pAr-pAr-pCr- | Cyclic phosphorylation | 28 | 1.99 | 100 | 1.1 | 47.46 | 1125.403 | -8 | 9008.2803 | 9012.17 | 9008.2624 | 1439472 |
| 414-441 | Ar-pAr-pAr-pAr-pAr-pGr-pCr-pGr-pCr-pCr-pAr-pCr-pAr-pAr-pCr- | Cyclic phosphorylation | 28 | 0.79 | 100 | 1 | 47.46 | 900.121 | -10 | 9008.2695 | 9012.29 | 9008.2624 | 4998068 |
| 414-441 | Ar-pAr-pAr-pAr-pAr-pGr-pCr-pGr-pCr-pCr-pAr-pCr-pAr-pAr-pCr- | Cyclic phosphorylation | 28 | 1.45 | 100 | 1 | 47.48 | 1000.246 | -9 | 9008.2754 | 9012.33 | 9008.2624 | 3201948.5 |
| 414-441 | Ar-pAr-pAr-pAr-pAr-pGr-pCr-pGr-pCr-pCr-pAr-pCr-pAr-pAr-pCr- | Cyclic phosphorylation | 28 | 1.12 | 100 | 1 | 47.48 | 818.2 | -11 | 9008.2725 | 9012.31 | 9008.2624 | 3964722.75 |
| 2160-2190 | Ar-pAr-pCr-pAr-pGr-pGr-pCr-pGr-pUr-pGr-pGr-pCr-pCr-pUr-pAr-pCr | Cyclic phosphorylation | 31 | -1.42 | 100 | 1.2 | 47.6 | 1097.03 | -9 | 9878.3018 | 9882.77 | 9878.3158 | 626301.88 |
| 2160-2190 | Ar-pAr-pCr-pAr-pGr-pGr-pCr-pGr-pUr-pGr-pGr-pCr-pCr-pUr-pAr-pCr | Cyclic phosphorylation | 31 | 1.25 | 100 | 1 | 47.6 | 897.387 | -11 | 9878.3281 | 9882.7 | 9878.3158 | 2115127.5 |
| 2160-2190 | Ar-pAr-pCr-pAr-pGr-pGr-pCr-pGr-pUr-pGr-pGr-pCr-pCr-pUr-pAr-pCr | Cyclic phosphorylation | 31 | 1.84 | 100 | 1 | 47.6 | 987.227 | -10 | 9878.334 | 9882.56 | 9878.3158 | 1399017 |
| 1305-1335 | Ar-pUr-pCr-pGr-pCr-pCr-pGr-pAr-pCr-pUr-pAr-pCr-pAr-pAr-pCr | Cyclic phosphorylation | 31 | 1.44 | 99.8 | 1.4 | 47.77 | 825.858 | -12 | 9916.3682 | 9920.74 | 9916.3539 | 504645.72 |
| 1305-1335 | Ar-pUr-pCr-pGr-pCr-pCr-pGr-pAr-pCr-pUr-pAr-pCr-pAr-pAr-pCr | Cyclic phosphorylation | 31 | 1.83 | 99.8 | 1.1 | 47.79 | 900.937 | -11 | 9916.3721 | 9920.81 | 9916.3539 | 347337.84 |
| 596-626 | Cr-pAr-pAr-pGr-pCr-pAr-pGr-pGr-pGr-pCr-pAr-pAr-pCr-pUr-pU | Cyclic phosphorylation | 31 | -2.93 | 99.2 | 1 | 48.44 | 913.849 | -11 | 10059.3701 | 10063.91 | 10059.3996 | 1338273.38 |
| 596-626 | Cr-pAr-pAr-pGr-pCr-pAr-pGr-pGr-pGr-pCr-pAr-pAr-pCr-pUr-pU | Cyclic phosphorylation | 31 | 3.09 | 99.2 | 1.1 | 48.45 | 837.527 | -12 | 10059.4307 | 10063.91 | 10059.3996 | 1747013.75 |
| 596-626 | Cr-pAr-pAr-pGr-pCr-pAr-pGr-pGr-pGr-pCr-pAr-pAr-pCr-pUr-pU | Cyclic phosphorylation | 31 | -1.57 | 99.2 | 1.3 | 48.49 | 1005.235 | -10 | 10059.3838 | 10063.97 | 10059.3996 | 738076.69 |
| 1671-1702 | Ar-pAr-pCr-pUr-pUr-pCr-pAr-pAr-pCr-pUr-pUr-pCr-pAr-pAr-pCr | Cyclic phosphorylation | 32 | 2.75 | 100 | 1.1 | 48.63 | 1023.133 | -10 | 10238.4023 | 10242.69 | 10238.3741 | 663135.88 |
| 1671-1702 | Ar-pAr-pCr-pUr-pUr-pCr-pAr-pAr-pCr-pUr-pUr-pCr-pAr-pAr-pCr | Cyclic phosphorylation | 32 | 2.09 | 100 | 1.1 | 48.63 | 786.946 | -13 | 10238.3955 | 10243.18 | 10238.3741 | 908963.94 |
| 1671-1702 | Ar-pAr-pCr-pUr-pUr-pCr-pAr-pAr-pCr-pUr-pUr-pCr-pAr-pAr-pCr | Cyclic phosphorylation | 32 | 1.13 | 100 | 1 | 48.64 | 852.526 | -12 | 10238.3857 | 10243.21 | 10238.3741 | 1258917.12 |
| 1671-1702 | Ar-pAr-pCr-pUr-pUr-pCr-pAr-pAr-pCr-pUr-pUr-pCr-pAr-pAr-pCr | Cyclic phosphorylation | 32 | 2.09 | 100 | 1 | 48.65 | 930.12 | -11 | 10238.3955 | 10242.92 | 10238.3741 | 1015755.88 |
| 2377-2406 | Ar-pCr-pAr-pAr-pGr-pAr-pAr-pGr-pAr-pCr-pCr-pCr-pAr-pAr-pGr | Cyclic phosphorylation | 30 | 1.23 | 100 | 1.2 | 49.22 | 1077.034 | -9 | 9698.3691 | 9702.64 | 9698.3572 | 664105.12 |
| 2377-2406 | Ar-pCr-pAr-pAr-pGr-pAr-pAr-pGr-pAr-pCr-pCr-pCr-pAr-pAr-pGr | Cyclic phosphorylation | 30 | 0.72 | 100 | 1 | 49.22 | 807.524 | -12 | 9698.3643 | 9702.63 | 9698.3572 | 2213847.25 |
| 2377-2406 | Ar-pCr-pAr-pAr-pGr-pAr-pAr-pGr-pAr-pCr-pCr-pCr-pAr-pAr-pGr | Cyclic phosphorylation | 30 | 1.43 | 100 | 1 | 49.22 | 969.23 | -10 | 9698.3711 | 9702.83 | 9698.3572 | 1248796.12 |
| 2377-2406 | Ar-pCr-pAr-pAr-pGr-pAr-pAr-pGr-pAr-pCr-pCr-pCr-pAr-pAr-pGr | Cyclic phosphorylation | 30 | 1.53 | 100 | 1 | 49.22 | 881.027 | -11 | 9698.3721 | 9702.66 | 9698.3572 | 1824709.88 |
| 2377-2406 | Ar-pCr-pAr-pAr-pGr-pAr-pAr-pGr-pAr-pCr-pCr-pCr-pAr-pAr-pGr | Cyclic phosphorylation | 30 | 2.23 | 100 | 1.2 | 49.22 | 745.329 | -13 | 9698.3789 | 9702.55 | 9698.3572 | 1188984.88 |
| 3078-3109 | Ar-pCr-pCr-pCr-pAr-pGr-pCr-pAr-pGr-pCr-pUr-pGr-pAr-pUr-pCr | Cyclic phosphorylation | 32 | 1.55 | 99.8 | 1.1 | 49.27 | 861.029 | -12 | 10341.4297 | 10346.11 | 10341.4137 | 1074906.75 |
| 882-912 | Cr-pUr-pGr-pAr-pAr-pGr-pUr-pAr-pCr-pAr-pAr-pCr-pGr-pAr-pG | Cyclic phosphorylation | 31 | -5.87 | 98.8 | 1 | 49.29 | 832.944 | -12 | 10003.3398 | 10007.77 | 10003.3985 | 690725.06 |
| 3078-3109 | Ar-pCr-pCr-pCr-pAr-pGr-pCr-pAr-pGr-pCr-pUr-pGr-pAr-pUr-pCr | Cyclic phosphorylation | 32 | -0.81 | 99.8 | 1.1 | 49.29 | 939.487 | -11 | 10341.4053 | 10345.93 | 10341.4137 | 733518.38 |
| 3078-3109 | Ar-pCr-pCr-pCr-pAr-pGr-pCr-pAr-pGr-pCr-pUr-pGr-pAr-pUr-pCr | Cyclic phosphorylation | 32 | 0.79 | 99.8 | 1.1 | 49.31 | 794.795 | -13 | 10341.4219 | 10346.09 | 10341.4137 | 1134909.88 |
| 1296-1325 | Ar-pCr-pAr-pGr-pGr-pCr-pAr-pAr-pGr-pAr-pUr-pCr-pGr-pCr-pCr | Cyclic phosphorylation | 30 | 4.23 | 100 | 1.2 | 50.2 | 879.574 | -11 | 9682.4033 | 9686.99 | 9682.3623 | 488432.22 |
| 1296-1325 | Ar-pCr-pAr-pGr-pGr-pCr-pAr-pAr-pGr-pAr-pUr-pCr-pGr-pCr-pCr | Cyclic phosphorylation | 30 | 2.32 | 100 | 1.2 | 50.22 | 967.632 | -10 | 9682.3848 | 9686.86 | 9682.3623 | 370560.72 |
| 1296-1325 | Ar-pCr-pAr-pGr-pGr-pCr-pAr-pAr-pGr-pAr-pUr-pCr-pGr-pCr-pCr | Cyclic phosphorylation | 30 | 3.12 | 100 | 1.4 | 50.22 | 806.275 | -12 | 9682.3926 | 9686.68 | 9682.3623 | 621543.06 |
| 2191-2226 | Cr-pUr-pAr-pUr-pCr-pCr-pCr-pAr-pCr-pCr-pAr-pAr-pCr-pUr | Cyclic phosphorylation | 36 | 1.89 | 100 | 1 | 50.53 | 1034.679 | -11 | 11387.5488 | 11392.72 | 11387.5273 | 1300109.75 |
| 2191-2226 | Cr-pUr-pAr-pUr-pCr-pCr-pCr-pAr-pCr-pCr-pAr-pAr-pCr-pUr | Cyclic phosphorylation | 36 | 0.69 | 100 | 1 | 50.54 | 948.372 | -12 | 11387.5352 | 11392.75 | 11387.5273 | 2226657.5 |
| 2191-2226 | Cr-pUr-pAr-pUr-pCr-pCr-pCr-pAr-pCr-pCr-pAr-pAr-pCr-pUr | Cyclic phosphorylation | 36 | 1.12 | 100 | 1 | 50.55 | 812.674 | -14 | 11387.54 | 11392.7 | 11387.5273 | 5429381.5 |
| 2191-2226 | Cr-pUr-pAr-pUr-pCr-pCr-pCr-pAr-pCr-pCr-pAr-pAr-pCr-pUr | Cyclic phosphorylation | 36 | 2.32 | 100 | 1 | 50.55 | 875.343 | -13 | 11387.5537 | 11392.68 | 11387.5273 | 3984054.5 |
| 2191-2226 | Cr-pUr-pAr-pUr-pCr-pCr-pCr-pAr-pCr-pCr-pAr-pAr-pCr-pUr | Cyclic phosphorylation | 36 | 1.46 | 100 | 1.3 | 50.55 | 758.43 | -15 | 11387.5439 | 11392.61 | 11387.5273 | 3232030 |
| 2191-2226 | Cr-pUr-pAr-pUr-pCr-pCr-pCr-pAr-pCr-pCr-pAr-pAr-pCr-pUr | Cyclic phosphorylation | 36 | -1.97 | 100 | 1.5 | 50.55 | 1138.148 | -10 | 11387.5049 | 11392.54 | 11387.5273 | 786983.19 |
| 2041-2078 | Ar-pCr-pAr-pUr-pCr-pCr-pCr-pCr-pAr-pUr-pCr-pGr-pCr-pGr-pGr | Cyclic phosphorylation | 38 | -0.08 | 99.8 | 1.2 | 51.83 | 867.109 | -14 | 12149.6172 | 12155.16 | 12149.6182 | 783118.19 |
| 2041-2078 | Ar-pCr-pAr-pUr-pCr-pCr-pCr-pCr-pAr-pUr-pCr-pGr-pCr-pGr-pGr | Cyclic phosphorylation | 38 | -1.45 | 99.8 | 1.3 | 51.83 | 933.965 | -13 | 12149.6006 | 12155.07 | 12149.6182 | 499070.28 |
| 2041-2078 | Ar-pCr-pAr-pUr-pCr-pCr-pCr-pCr-pAr-pUr-pCr-pGr-pCr-pGr-pGr | Cyclic phosphorylation | 38 | 0.96 | 99.8 | 1.2 | 51.84 | 809.236 | -15 | 12149.6299 | 12155.07 | 12149.6182 | 940533.25 |
| 2191-2228 | Cr-pUr-pAr-pUr-pCr-pCr-pCr-pAr-pCr-pCr-pAr-pAr-pCr-pUr | Cyclic phosphorylation | 38 | -7.23 | 96.2 | 1.4 | 52.8 | 860.968 | -14 | 12061.5615 | 12067.22 | 12061.6273 | 530275.44 |
| 2191-2228 | Cr-pUr-pAr-pUr-pCr-pCr-pCr-pAr-pCr-pCr-pAr-pAr-pCr-pUr | Cyclic phosphorylation | 38 | 2.48 | 96.2 | 1.4 | 52.82 | 803.37 | -15 | 12061.6572 | 12067.19 | 12061.6273 | 704437.81 |
| 49-95 | Cr-pCr-pAr-pCr-pCr-pAr-pUr-pGr-pCr-pUr-pCr-pGr-pUr-pGr-pU | Cyclic phosphorylation | 47 | 1.91 | 99.7 | 1.6 | 53.2 | 879.809 | -17 | 14966.8701 | 14973.62 | 14966.8415 | 471986.19 |
| 1559-1599 | Cr-pGr-pUr-pGr-pGr-pGr-pCr-pUr-pAr-pUr-pCr-pAr-pGr-pCr-pC | Cyclic phosphorylation | 41 | 0.87 | 100 | 1.4 | 53.43 | 883.374 | -15 | 13260.7041 | 13266.5 | 13260.6925 | 1185411.5 |
| 1559-1599 | Cr-pGr-pUr-pGr-pGr-pGr-pCr-pUr-pAr-pUr-pCr-pAr-pGr-pCr-pC | Cyclic phosphorylation | 41 | -1.34 | 100 | 1.5 | 53.44 | 1019.51 | -13 | 13260.6748 | 13266.53 | 13260.6925 | 527210.12 |
| 2060-2092 | Ar-pAr-pUr-pCr-pUr-pGr-pCr-pGr-pCr-pCr-pAr-pGr-pCr-pUr-pA | Cyclic phosphorylation | 33 | -0.12 | 99.4 | 1.1 | 53.71 | 884.453 | -12 | 10621.4961 | 10626.29 | 10621.4974 | 737044.06 |

| | | | | | | | | | | | | | |
|---|---|---|---|---|---|---|---|---|---|---|---|---|---|
| 2060-2092 | Ar-pAr-pUr-pCr-pUr-pGr-pCr-pGr-pCr-pCr-pAr-pGr-pCr-pUr-pA | Cyclic phosphorylation | 33 | 0.7 | 99.4 | 1.2 | 53.74 | 816.34 | -13 | 10621.5049 | 10626.32 | 10621.4974 | 836303.81 |
| 1558-1599 | Gr-pCr-pGr-pUr-pGr-pGr-pGr-pCr-pUr-pAr-pUr-pCr-pAr-pGr-pC | Cyclic phosphorylation | 42 | -0.12 | 100 | 1.7 | 54.07 | 849.728 | -16 | 13605.7383 | 13612.2 | 13605.74 | 330017.56 |
| 1558-1599 | Gr-pCr-pGr-pUr-pGr-pGr-pGr-pCr-pUr-pAr-pUr-pCr-pAr-pGr-pC | Cyclic phosphorylation | 42 | 0.09 | 100 | 1.2 | 54.1 | 906.377 | -15 | 13605.7412 | 13611.74 | 13605.74 | 737211 |
| 3038-3075 | Ar-pCr-pUr-pGr-pAr-pUr-pCr-pAr-pCr-pAr-pGr-pGr-pCr-pAr-pG | Cyclic phosphorylation | 38 | -1.31 | 95.6 | 1.6 | 55.35 | 873.972 | -14 | 12244.6631 | 12250.09 | 12244.6791 | 355957.72 |
| 3038-3075 | Ar-pCr-pUr-pGr-pAr-pUr-pCr-pAr-pCr-pAr-pGr-pGr-pCr-pAr-pG | Cyclic phosphorylation | 38 | -3.22 | 95.6 | 1.7 | 55.37 | 815.707 | -15 | 12244.6396 | 12250.34 | 12244.6791 | 388012.94 |
| 1113-1152 | Ar-pAr-pCr-pCr-pGr-pGr-pAr-pAr-pGr-pCr-pGr-pGr-pAr-pUr-pC | Cyclic phosphorylation | 40 | -9.7 | 100 | 1.1 | 55.86 | 807.284 | -16 | 12927.6162 | 12932.18 | 12927.7417 | 1006289.81 |
| 1113-1152 | Ar-pAr-pCr-pCr-pGr-pGr-pAr-pAr-pGr-pCr-pGr-pGr-pAr-pUr-pC | Cyclic phosphorylation | 40 | -3.43 | 100 | 1 | 55.96 | 861.177 | -15 | 12927.6973 | 12932.9 | 12927.7417 | 2074526.62 |

| Positions | Oligo Sequence | 3' Termini | Seq Length | Δ ppm | Conf. Score | Best ASR | RT | M/Z | Charge St. | Mono Mass Exp. | Avg Mass Exp. | Mono Mass Theo. | MS Area |
|---|---|---|---|---|---|---|---|---|---|---|---|---|---|
| 314-323 | Cr-pGr-pAr-pCr-pGr-pGr-pGr-pGr-pUr-pGr | Cyclic phosphorylation | 10 | -0.57 | 99.3 | 1 | 5.43 | 662.282 | -5 | 3315.4431 | 3316.86 | 3315.445 | 11090002 |
| 2634-2645 | Cr-pUr-pGr-pCr-pCr-pUr-pCr-pCr-pUr-pCr-pUr-pGr | Cyclic phosphorylation | 12 | -0.57 | 97.2 | 1 | 5.89 | 623.233 | -6 | 3744.4417 | 3746.01 | 3744.4438 | 10172427 |
| 1990-1999 | Gr-pAr-pGr-pCr-pCr-pGr-pGr-pCr-pUr-pGr | Cyclic phosphorylation | 10 | -0.94 | 97.3 | 1 | 6.04 | 654.28 | -5 | 3275.4358 | 3276.7 | 3275.4389 | 13566792 |
| 314-323 | Cr-pGr-pAr-pCr-pGr-pGr-pGr-pGr-pUr-pGr | Cyclic phosphorylation | 10 | -1.08 | 99.5 | 1 | 6.08 | 662.281 | -5 | 3315.4414 | 3316.85 | 3315.445 | 16387818 |
| 3094-3103 | Gr-pAr-pGr-pCr-pCr-pGr-pCr-pCr-pGr-pAr | Cyclic phosphorylation | 10 | -0.74 | 99.2 | 1 | 6.36 | 813.856 | -4 | 3258.4575 | 3260.07 | 3258.4599 | 7617890 |
| 3094-3103 | Gr-pAr-pGr-pCr-pCr-pGr-pCr-pCr-pGr-pAr | Cyclic phosphorylation | 10 | -0.36 | 99.2 | 1 | 6.36 | 1085.477 | -3 | 3258.4587 | 3260.17 | 3258.4599 | 7464381.5 |
| 3094-3103 | Gr-pAr-pGr-pCr-pCr-pGr-pCr-pCr-pGr-pAr | Cyclic phosphorylation | 10 | -1.11 | 99.2 | 1 | 6.36 | 650.883 | -5 | 3258.4563 | 3260.12 | 3258.4599 | 29266484 |
| 42-51 | Gr-pAr-pAr-pCr-pCr-pCr-pGr-pCr-pCr-pAr | Cyclic phosphorylation | 10 | -0.57 | 93.2 | 1 | 6.73 | 799.857 | -4 | 3202.457 | 3203.9 | 3202.4589 | 5589773 |
| 1961-1971 | Cr-pUr-pCr-pCr-pAr-pCr-pCr-pGr-pGr-pCr-pAr | Cyclic phosphorylation | 11 | -0.5 | 94.5 | 1 | 7.01 | 1160.815 | -3 | 3484.4712 | 3486.46 | 3484.4729 | 11670685 |
| 1961-1971 | Cr-pUr-pCr-pCr-pAr-pCr-pCr-pGr-pGr-pCr-pAr | Cyclic phosphorylation | 11 | -0.57 | 94.5 | 1 | 7.02 | 496.917 | -7 | 3484.4709 | 3486.1 | 3484.4729 | 18800562 |
| 832-843 | Cr-pUr-pGr-pGr-pUr-pGr-pCr-pCr-pGr-pCr-pCr-pGr | Cyclic phosphorylation | 12 | -0.59 | 100 | 1 | 7.02 | 550.92 | -7 | 3862.4919 | 3864.08 | 3862.4942 | 5237625.5 |
| 832-843 | Cr-pUr-pGr-pGr-pUr-pGr-pCr-pCr-pGr-pCr-pCr-pGr | Cyclic phosphorylation | 12 | -0.84 | 100 | 1 | 7.04 | 642.908 | -6 | 3862.491 | 3864.06 | 3862.4942 | 17637888 |
| 1092-1103 | Ur-pUr-pCr-pGr-pCr-pCr-pCr-pUr-pCr-pUr-pGr-pUr-pGr | Cyclic phosphorylation | 12 | -0.48 | 95.1 | 1 | 7.06 | 630.065 | -6 | 3785.4321 | 3786.96 | 3785.434 | 9924528 |
| 151-161 | Cr-pCr-pAr-pGr-pAr-pGr-pGr-pCr-pGr-pUr-pGr | Cyclic phosphorylation | 11 | -1.47 | 99.1 | 1 | 7.34 | 599.907 | -6 | 3604.4861 | 3606 | 3604.4914 | 42949264 |
| 151-161 | Cr-pCr-pAr-pGr-pAr-pGr-pGr-pCr-pGr-pUr-pGr | Cyclic phosphorylation | 11 | -0.72 | 99.1 | 1 | 7.36 | 1200.823 | -3 | 3604.4888 | 3605.98 | 3604.4914 | 9276050 |
| 151-161 | Cr-pCr-pAr-pGr-pAr-pGr-pGr-pCr-pGr-pUr-pGr | Cyclic phosphorylation | 11 | -0.93 | 99.1 | 1 | 7.39 | 720.091 | -5 | 3604.488 | 3606.01 | 3604.4914 | 23584758 |
| 814-824 | Gr-pCr-pAr-pGr-pCr-pAr-pGr-pCr-pGr-pGr-pAr | Cyclic phosphorylation | 11 | -0.75 | 94.9 | 1 | 7.97 | 724.696 | -5 | 3627.5159 | 3629.01 | 3627.5186 | 6801343.5 |
| 814-824 | Gr-pCr-pAr-pGr-pCr-pAr-pGr-pCr-pGr-pGr-pAr | Cyclic phosphorylation | 11 | -0.62 | 94.9 | 1 | 7.99 | 603.746 | -6 | 3627.5164 | 3629.03 | 3627.5186 | 9076859 |
| 1961-1972 | Cr-pUr-pCr-pCr-pAr-pCr-pCr-pGr-pGr-pCr-pAr-pGr | Cyclic phosphorylation | 12 | -0.54 | 97.8 | 1 | 8.06 | 637.412 | -6 | 3829.5183 | 3831.49 | 3829.5204 | 4242058.5 |
| 1556-1567 | Ur-pGr-pGr-pCr-pGr-pUr-pGr-pGr-pGr-pCr-pUr-pAr | Cyclic phosphorylation | 12 | -0.62 | 98.7 | 1 | 8.22 | 560.206 | -7 | 3927.4932 | 3929.18 | 3927.4956 | 11014133 |
| 1556-1567 | Ur-pGr-pGr-pCr-pGr-pUr-pGr-pGr-pGr-pCr-pUr-pAr | Cyclic phosphorylation | 12 | 0 | 98.7 | 1 | 8.22 | 653.742 | -6 | 3927.4956 | 3929.12 | 3927.4956 | 14610892 |
| 1556-1567 | Ur-pGr-pGr-pCr-pGr-pUr-pGr-pGr-pGr-pCr-pUr-pAr | Cyclic phosphorylation | 12 | 0.12 | 98.7 | 1 | 8.22 | 1308.491 | -3 | 3927.4961 | 3929.22 | 3927.4956 | 4604466.5 |
| 686-697 | Cr-pCr-pUr-pCr-pGr-pUr-pGr-pCr-pGr-pGr-pGr-pAr | Cyclic phosphorylation | 12 | -0.71 | 98.5 | 1 | 8.34 | 647.075 | -6 | 3886.5027 | 3888.97 | 3886.5054 | 15034379 |
| 686-697 | Cr-pCr-pUr-pCr-pGr-pUr-pGr-pCr-pGr-pGr-pGr-pAr | Cyclic phosphorylation | 12 | -0.33 | 98.5 | 1 | 8.34 | 1295.157 | -3 | 3886.5042 | 3889.07 | 3886.5054 | 3645402 |
| 686-697 | Cr-pCr-pUr-pCr-pGr-pUr-pGr-pCr-pGr-pGr-pGr-pAr | Cyclic phosphorylation | 12 | -0.58 | 98.5 | 1 | 8.34 | 554.492 | -7 | 3886.5032 | 3888.9 | 3886.5054 | 7100537 |
| 3911-3924 | Cr-pUr-pGr-pCr-pCr-pCr-pCr-pUr-pUr-pCr-pCr-pCr-pGr | Cyclic phosphorylation | 14 | -0.79 | 100 | 1.1 | 8.36 | 724.914 | -6 | 4354.5229 | 4356.19 | 4354.5264 | 573134.12 |
| 2637-2649 | Cr-pCr-pUr-pCr-pCr-pUr-pCr-pUr-pGr-pCr-pUr-pGr-pAr | Cyclic phosphorylation | 13 | -0.17 | 95.4 | 1 | 8.43 | 581.064 | -7 | 4073.4956 | 4075.08 | 4073.4963 | 3564010.5 |
| 831-843 | Gr-pCr-pUr-pGr-pGr-pUr-pGr-pCr-pCr-pGr-pCr-pCr-pGr | Cyclic phosphorylation | 13 | -0.38 | 100 | 1 | 8.43 | 700.416 | -6 | 4207.54 | 4209.38 | 4207.5416 | 4262774 |
| 3911-3924 | Cr-pUr-pGr-pCr-pCr-pCr-pCr-pUr-pUr-pCr-pCr-pCr-pGr | Cyclic phosphorylation | 14 | -0.45 | 100 | 1 | 8.48 | 543.434 | -8 | 4354.5244 | 4356.27 | 4354.5264 | 2010231.88 |
| 3911-3924 | Cr-pUr-pGr-pCr-pCr-pCr-pCr-pUr-pUr-pCr-pCr-pCr-pGr | Cyclic phosphorylation | 14 | -0.45 | 100 | 1 | 8.48 | 621.211 | -7 | 4354.5244 | 4356.26 | 4354.5264 | 4600544 |
| 3911-3924 | Cr-pUr-pGr-pCr-pCr-pCr-pCr-pUr-pUr-pCr-pCr-pCr-pGr | Cyclic phosphorylation | 14 | -0.45 | 100 | 1.1 | 8.5 | 724.914 | -6 | 4354.5244 | 4356.25 | 4354.5264 | 1697512.25 |
| 1160-1171 | Cr-pAr-pAr-pCr-pUr-pCr-pCr-pGr-pCr-pCr-pAr-pGr | Cyclic phosphorylation | 12 | -0.4 | 98.4 | 1 | 8.57 | 543.925 | -7 | 3813.5239 | 3815.42 | 3813.5255 | 2213539 |
| 1160-1171 | Cr-pAr-pAr-pCr-pUr-pCr-pCr-pGr-pCr-pCr-pAr-pGr | Cyclic phosphorylation | 12 | -0.46 | 98.4 | 1 | 8.57 | 634.747 | -6 | 3813.5237 | 3815.21 | 3813.5255 | 5384630.5 |
| 1322-1333 | Cr-pAr-pAr-pGr-pCr-pUr-pGr-pCr-pCr-pCr-pGr-pAr | Cyclic phosphorylation | 12 | -0.6 | 99.9 | 1 | 8.86 | 641.414 | -6 | 3853.5293 | 3855.14 | 3853.5316 | 28663876 |
| 1322-1333 | Cr-pAr-pAr-pGr-pCr-pUr-pGr-pCr-pCr-pCr-pGr-pAr | Cyclic phosphorylation | 12 | -0.47 | 99.9 | 1 | 8.86 | 769.899 | -5 | 3853.5298 | 3855.14 | 3853.5316 | 8009602 |
| 1322-1333 | Cr-pAr-pAr-pGr-pCr-pUr-pGr-pCr-pCr-pCr-pGr-pAr | Cyclic phosphorylation | 12 | -0.15 | 99.9 | 1 | 8.86 | 1283.837 | -3 | 3853.531 | 3855.18 | 3853.5316 | 8123332.5 |
| 1322-1333 | Cr-pAr-pAr-pGr-pCr-pUr-pGr-pCr-pCr-pCr-pGr-pAr | Cyclic phosphorylation | 12 | -0.41 | 99.9 | 1 | 8.86 | 549.64 | -7 | 3853.53 | 3855.16 | 3853.5316 | 16823666 |
| 1911-1923 | Gr-pUr-pGr-pCr-pCr-pCr-pGr-pUr-pGr-pGr-pCr-pCr-pAr | Cyclic phosphorylation | 13 | -0.43 | 99.8 | 1 | 8.88 | 597.928 | -7 | 4191.5449 | 4193.24 | 4191.5467 | 12911681 |
| 1911-1923 | Gr-pUr-pGr-pCr-pCr-pCr-pGr-pUr-pGr-pGr-pCr-pCr-pAr | Cyclic phosphorylation | 13 | -0.66 | 99.8 | 1 | 8.88 | 697.75 | -6 | 4191.5439 | 4193.18 | 4191.5467 | 9697895 |
| 1406-1418 | Cr-pCr-pUr-pGr-pUr-pAr-pCr-pCr-pGr-pGr-pCr-pUr-pGr | Cyclic phosphorylation | 13 | 0.31 | 100 | 1 | 9.3 | 592.496 | -7 | 4152.5259 | 4154.31 | 4152.5246 | 17354634 |
| 1406-1418 | Cr-pCr-pUr-pGr-pUr-pAr-pCr-pCr-pGr-pGr-pCr-pUr-pGr | Cyclic phosphorylation | 13 | -0.16 | 100 | 1 | 9.3 | 691.414 | -6 | 4152.5239 | 4154.39 | 4152.5246 | 12969069 |
| 1623-1635 | Gr-pUr-pGr-pUr-pGr-pCr-pGr-pGr-pCr-pCr-pCr-pUr-pAr | Cyclic phosphorylation | 13 | 0.47 | 96.7 | 1 | 9.39 | 598.069 | -7 | 4192.5327 | 4194.41 | 4192.5307 | 5111100 |
| 1623-1635 | Gr-pUr-pGr-pUr-pGr-pCr-pGr-pGr-pCr-pCr-pCr-pUr-pAr | Cyclic phosphorylation | 13 | 0.59 | 96.7 | 1 | 9.39 | 697.915 | -6 | 4192.5332 | 4194.36 | 4192.5307 | 5906635.5 |
| 314-325 | Cr-pGr-pAr-pCr-pGr-pGr-pGr-pGr-pUr-pGr-pUr-pAr | Cyclic phosphorylation | 12 | 0.59 | 100 | 1 | 9.51 | 657.58 | -6 | 3950.5251 | 3952.08 | 3950.5228 | 13997593 |
| 3614-3625 | Cr-pGr-pAr-pGr-pGr-pUr-pGr-pGr-pCr-pCr-pAr-pAr | Cyclic phosphorylation | 12 | -0.11 | 90.7 | 1 | 9.71 | 785.902 | -5 | 3933.5435 | 3935.13 | 3933.5439 | 10773823 |
| 3614-3625 | Cr-pGr-pAr-pGr-pGr-pUr-pGr-pGr-pCr-pCr-pAr-pAr | Cyclic phosphorylation | 12 | -0.67 | 90.7 | 1 | 9.74 | 654.75 | -6 | 3933.5413 | 3935.18 | 3933.5439 | 30908918 |
| 3614-3625 | Cr-pGr-pAr-pGr-pGr-pUr-pGr-pGr-pCr-pCr-pAr-pAr | Cyclic phosphorylation | 12 | -0.17 | 90.7 | 1 | 9.75 | 1310.508 | -3 | 3933.5432 | 3935.17 | 3933.5439 | 9331216 |
| 3614-3625 | Cr-pGr-pAr-pGr-pGr-pUr-pGr-pGr-pCr-pCr-pAr-pAr | Cyclic phosphorylation | 12 | -0.11 | 90.7 | 1 | 9.83 | 561.07 | -7 | 3933.5435 | 3935.19 | 3933.5439 | 14805064 |
| 1992-2004 | Gr-pCr-pCr-pGr-pGr-pCr-pUr-pGr-pUr-pCr-pUr-pGr-pAr | Cyclic phosphorylation | 13 | -0.46 | 93.1 | 1 | 9.92 | 697.915 | -6 | 4192.5288 | 4194.22 | 4192.5307 | 6616081 |
| 1992-2004 | Gr-pCr-pCr-pGr-pGr-pCr-pUr-pGr-pUr-pCr-pUr-pGr-pAr | Cyclic phosphorylation | 13 | 0.24 | 93.1 | 1 | 9.92 | 598.069 | -7 | 4192.5317 | 4194.21 | 4192.5307 | 5117761.5 |
| 2722-2735 | Cr-pAr-pGr-pGr-pCr-pGr-pCr-pCr-pGr-pCr-pUr-pCr-pUr-pGr | Cyclic phosphorylation | 14 | -0.25 | 99.8 | 1 | 10.19 | 641.505 | -7 | 4496.5869 | 4498.56 | 4496.588 | 7207279 |
| 910-922 | Cr-pCr-pGr-pAr-pCr-pGr-pCr-pCr-pGr-pUr-pGr-pGr-pAr | Cyclic phosphorylation | 13 | -0.28 | 100 | 1 | 10.21 | 1404.517 | -3 | 4214.5728 | 4216.62 | 4214.5739 | 10941869 |
| 910-922 | Cr-pCr-pGr-pAr-pCr-pGr-pCr-pCr-pGr-pUr-pGr-pGr-pAr | Cyclic phosphorylation | 13 | 0.06 | 100 | 1 | 10.21 | 1053.136 | -4 | 4214.5742 | 4216.6 | 4214.5739 | 4617632.5 |
| 910-922 | Cr-pCr-pGr-pAr-pCr-pGr-pCr-pCr-pGr-pUr-pGr-pGr-pAr | Cyclic phosphorylation | 13 | -0.17 | 100 | 1 | 10.21 | 842.107 | -5 | 4214.5732 | 4216.57 | 4214.5739 | 6422309 |
| 910-922 | Cr-pCr-pGr-pAr-pCr-pGr-pCr-pCr-pGr-pUr-pGr-pGr-pAr | Cyclic phosphorylation | 13 | -0.4 | 100 | 1 | 10.21 | 701.588 | -6 | 4214.5723 | 4216.57 | 4214.5739 | 23244198 |
| 910-922 | Cr-pCr-pGr-pAr-pCr-pGr-pCr-pCr-pGr-pUr-pGr-pGr-pAr | Cyclic phosphorylation | 13 | -0.05 | 100 | 1 | 10.21 | 601.359 | -7 | 4214.5737 | 4216.61 | 4214.5739 | 37180528 |
| 1193-1206 | Cr-pGr-pGr-pCr-pGr-pUr-pGr-pUr-pCr-pCr-pCr-pCr-pUr-pAr | Cyclic phosphorylation | 14 | -0.32 | 99.9 | 1 | 10.33 | 742.087 | -6 | 4457.5645 | 4459.33 | 4457.5659 | 14836176 |
| 1193-1206 | Cr-pGr-pGr-pCr-pGr-pUr-pGr-pUr-pCr-pCr-pCr-pCr-pUr-pAr | Cyclic phosphorylation | 14 | 0.01 | 99.9 | 1 | 10.34 | 635.931 | -7 | 4457.5659 | 4459.47 | 4457.5659 | 25345276 |
| 1193-1206 | Cr-pGr-pGr-pCr-pGr-pUr-pGr-pUr-pCr-pCr-pCr-pCr-pUr-pAr | Cyclic phosphorylation | 14 | -0.21 | 99.9 | 1 | 10.34 | 1485.182 | -3 | 4457.5649 | 4459.52 | 4457.5659 | 7132931.5 |
| 1538-1551 | Cr-pGr-pGr-pCr-pUr-pUr-pUr-pCr-pAr-pGr-pCr-pCr-pCr-pAr | Cyclic phosphorylation | 14 | 0.62 | 99.4 | 1.1 | 10.36 | 554.315 | -8 | 4441.5737 | 4443.49 | 4441.571 | 4656149.5 |
| 1193-1206 | Cr-pGr-pGr-pCr-pGr-pUr-pGr-pUr-pCr-pCr-pCr-pCr-pUr-pAr | Cyclic phosphorylation | 14 | 0.12 | 99.9 | 1 | 10.38 | 556.439 | -8 | 4457.5664 | 4459.46 | 4457.5659 | 5974220.5 |
| 1538-1551 | Cr-pGr-pGr-pCr-pUr-pUr-pUr-pCr-pAr-pGr-pCr-pCr-pCr-pAr | Cyclic phosphorylation | 14 | 0.29 | 99.4 | 1 | 10.41 | 633.646 | -7 | 4441.5723 | 4443.55 | 4441.571 | 9841554 |
| 1538-1551 | Cr-pGr-pGr-pCr-pUr-pUr-pUr-pCr-pAr-pGr-pCr-pCr-pCr-pAr | Cyclic phosphorylation | 14 | -0.04 | 99.4 | 1 | 10.43 | 739.422 | -6 | 4441.5708 | 4443.51 | 4441.571 | 7982691.5 |
| 4062-4074 | Cr-pAr-pCr-pCr-pCr-pCr-pCr-pAr-pCr-pGr-pGr-pGr-pAr | Cyclic phosphorylation | 13 | 0.12 | 99.9 | 1 | 10.46 | 692.091 | -6 | 4157.5894 | 4159.53 | 4157.5889 | 4204112 |
| 4062-4074 | Cr-pAr-pCr-pCr-pCr-pCr-pCr-pAr-pCr-pGr-pGr-pGr-pAr | Cyclic phosphorylation | 13 | 0.35 | 99.9 | 1 | 10.48 | 593.077 | -7 | 4157.5903 | 4159.59 | 4157.5889 | 3877605.75 |
| 1581-1593 | Gr-pUr-pGr-pGr-pUr-pGr-pGr-pUr-pGr-pCr-pUr-pGr-pAr | Cyclic phosphorylation | 13 | -0.28 | 98.1 | 1 | 10.65 | 711.414 | -6 | 4273.5259 | 4275.23 | 4273.5271 | 3225663 |
| 3091-3103 | Ur-pCr-pAr-pGr-pAr-pGr-pCr-pCr-pGr-pCr-pCr-pGr-pAr | Cyclic phosphorylation | 13 | 0.13 | 99.3 | 1 | 10.71 | 523.94 | -8 | 4198.5796 | 4200.28 | 4198.579 | 3553722.75 |
| 3091-3103 | Ur-pCr-pAr-pGr-pAr-pGr-pCr-pCr-pGr-pCr-pCr-pGr-pAr | Cyclic phosphorylation | 13 | -0.33 | 99.3 | 1 | 10.72 | 1048.888 | -4 | 4198.5776 | 4200.46 | 4198.579 | 4397861 |
| 3091-3103 | Ur-pCr-pAr-pGr-pAr-pGr-pCr-pCr-pGr-pCr-pCr-pGr-pAr | Cyclic phosphorylation | 13 | -0.45 | 99.3 | 1 | 10.72 | 598.932 | -7 | 4198.5771 | 4200.3 | 4198.579 | 30365508 |
| 3091-3103 | Ur-pCr-pAr-pGr-pAr-pGr-pCr-pCr-pGr-pCr-pCr-pGr-pAr | Cyclic phosphorylation | 13 | -0.33 | 99.3 | 1 | 10.74 | 839.109 | -5 | 4198.5776 | 4200.34 | 4198.579 | 8214214 |
| 3091-3103 | Ur-pCr-pAr-pGr-pAr-pGr-pCr-pCr-pGr-pCr-pCr-pGr-pAr | Cyclic phosphorylation | 13 | -0.33 | 99.3 | 1 | 10.74 | 698.922 | -6 | 4198.5776 | 4200.48 | 4198.579 | 19519592 |
| 3091-3103 | Ur-pCr-pAr-pGr-pAr-pGr-pCr-pCr-pGr-pCr-pCr-pGr-pAr | Cyclic phosphorylation | 13 | -0.33 | 99.3 | 1 | 10.74 | 1398.853 | -3 | 4198.5776 | 4200.48 | 4198.579 | 9353747 |
| 1693-1706 | Cr-pCr-pGr-pCr-pAr-pCr-pCr-pGr-pGr-pCr-pGr-pCr-pUr-pGr | Cyclic phosphorylation | 14 | -0.28 | 99.8 | 1 | 10.98 | 647.223 | -7 | 4535.6089 | 4537.56 | 4535.6101 | 13564842 |
| 2602-2614 | Ur-pUr-pUr-pGr-pCr-pGr-pCr-pCr-pCr-pAr-pGr-pAr-pAr | Cyclic phosphorylation | 13 | 1.2 | 99.7 | 1 | 11.03 | 519.186 | -8 | 4160.5459 | 4162.19 | 4160.5409 | 4863406.5 |
| 2145-2158 | Ur-pCr-pUr-pCr-pUr-pGr-pGr-pGr-pCr-pGr-pCr-pCr-pGr-pAr | Cyclic phosphorylation | 14 | -0.27 | 99.9 | 1 | 11.3 | 1498.517 | -3 | 4497.5708 | 4499.53 | 4497.572 | 10198667 |
| 2145-2158 | Ur-pCr-pUr-pCr-pUr-pGr-pGr-pGr-pCr-pGr-pCr-pCr-pGr-pAr | Cyclic phosphorylation | 14 | -0.38 | 99.9 | 1 | 11.32 | 561.314 | -8 | 4497.5703 | 4499.49 | 4497.572 | 17317010 |
| 2145-2158 | Ur-pCr-pUr-pCr-pUr-pGr-pGr-pGr-pCr-pGr-pCr-pCr-pGr-pAr | Cyclic phosphorylation | 14 | -0.6 | 99.9 | 1 | 11.32 | 641.789 | -7 | 4497.5693 | 4499.51 | 4497.572 | 31294434 |
| 2145-2158 | Ur-pCr-pUr-pCr-pUr-pGr-pGr-pGr-pCr-pGr-pCr-pCr-pGr-pAr | Cyclic phosphorylation | 14 | -0.38 | 99.9 | 1 | 11.32 | 748.755 | -6 | 4497.5703 | 4499.51 | 4497.572 | 14861367 |
| 3601-3613 | Ur-pCr-pGr-pAr-pCr-pCr-pGr-pGr-pCr-pUr-pGr-pAr-pAr | Cyclic phosphorylation | 13 | 0.45 | 99.7 | 1 | 11.47 | 699.253 | -6 | 4199.5649 | 4201.55 | 4199.563 | 12283314 |
| 2441-2454 | Cr-pUr-pUr-pCr-pGr-pGr-pCr-pGr-pGr-pCr-pUr-pUr-pCr-pAr | Cyclic phosphorylation | 14 | -0.13 | 100 | 1 | 11.49 | 636.071 | -7 | 4458.5493 | 4460.46 | 4458.5499 | 22299508 |
| 2441-2454 | Cr-pUr-pUr-pCr-pGr-pGr-pCr-pGr-pGr-pCr-pUr-pUr-pCr-pAr | Cyclic phosphorylation | 14 | -0.13 | 100 | 1 | 11.51 | 1485.51 | -3 | 4458.5493 | 4460.52 | 4458.5499 | 8455149 |
| 2441-2454 | Cr-pUr-pUr-pCr-pGr-pGr-pCr-pGr-pGr-pCr-pUr-pUr-pCr-pAr | Cyclic phosphorylation | 14 | -0.13 | 100 | 1 | 11.51 | 742.418 | -6 | 4458.5493 | 4460.51 | 4458.5499 | 5856164 |
| 2441-2454 | Cr-pUr-pUr-pCr-pGr-pGr-pCr-pGr-pGr-pCr-pUr-pUr-pCr-pAr | Cyclic phosphorylation | 14 | -0.35 | 100 | 1 | 11.51 | 556.436 | -8 | 4458.5483 | 4460.47 | 4458.5499 | 24516108 |
| 3601-3613 | Ur-pCr-pGr-pAr-pCr-pCr-pGr-pGr-pCr-pUr-pGr-pAr-pAr | Cyclic phosphorylation | 13 | 0.33 | 99.7 | 1 | 11.59 | 599.073 | -7 | 4199.5645 | 4201.42 | 4199.563 | 24362662 |
| 3601-3613 | Ur-pCr-pGr-pAr-pCr-pCr-pGr-pGr-pCr-pUr-pGr-pAr-pAr | Cyclic phosphorylation | 13 | 0.8 | 99.7 | 1 | 11.61 | 1399.181 | -3 | 4199.5664 | 4201.45 | 4199.563 | 7919372.5 |
| 3975-3989 | Cr-pCr-pUr-pCr-pCr-pAr-pCr-pCr-pUr-pGr-pCr-pCr-pCr-pCr-pAr | Cyclic phosphorylation | 15 | -0.15 | 99 | 1 | 11.75 | 665.796 | -7 | 4665.6152 | 4667.63 | 4665.6159 | 9000338 |
| 3975-3989 | Cr-pCr-pUr-pCr-pCr-pAr-pCr-pCr-pUr-pGr-pCr-pCr-pCr-pCr-pAr | Cyclic phosphorylation | 15 | 0.27 | 99 | 1 | 11.77 | 582.32 | -8 | 4665.6172 | 4667.58 | 4665.6159 | 12745062 |
| 151-163 | Cr-pCr-pAr-pGr-pAr-pGr-pGr-pCr-pGr-pUr-pGr-pUr-pAr | Cyclic phosphorylation | 13 | 0.61 | 100 | 1 | 12.09 | 1412.516 | -3 | 4239.5718 | 4241.47 | 4239.5692 | 3813357.5 |
| 151-163 | Cr-pCr-pAr-pGr-pAr-pGr-pGr-pCr-pGr-pUr-pGr-pUr-pAr | Cyclic phosphorylation | 13 | 1.07 | 100 | 1 | 12.09 | 705.755 | -6 | 4239.5737 | 4241.43 | 4239.5692 | 4872299.5 |
| 151-163 | Cr-pCr-pAr-pGr-pAr-pGr-pGr-pCr-pGr-pUr-pGr-pUr-pAr | Cyclic phosphorylation | 13 | 0.61 | 100 | 1 | 12.11 | 604.789 | -7 | 4239.5718 | 4241.47 | 4239.5692 | 9055537 |
| 3846-3858 | Cr-pUr-pGr-pAr-pAr-pGr-pGr-pGr-pCr-pGr-pUr-pGr-pAr | Cyclic phosphorylation | 13 | 0.42 | 99.3 | 1 | 12.16 | 712.422 | -6 | 4279.5771 | 4281.24 | 4279.5753 | 3691480.25 |
| 3846-3858 | Cr-pUr-pGr-pAr-pAr-pGr-pGr-pGr-pCr-pGr-pUr-pGr-pAr | Cyclic phosphorylation | 13 | 0.19 | 99.3 | 1 | 12.16 | 610.647 | -7 | 4279.5762 | 4281.28 | 4279.5753 | 6555739 |
| 3054-3067 | Cr-pUr-pGr-pCr-pAr-pGr-pAr-pGr-pCr-pGr-pCr-pCr-pCr-pAr | Cyclic phosphorylation | 14 | 1.41 | 91.5 | 1 | 12.49 | 636.936 | -7 | 4464.6045 | 4466.3 | 4464.5982 | 6888129.5 |
| 15-29 | Ur-pUr-pCr-pUr-pUr-pCr-pUr-pGr-pGr-pUr-pCr-pCr-pCr-pCr-pA | Cyclic phosphorylation | 15 | -0.11 | 100 | 1 | 12.66 | 519.832 | -9 | 4685.5464 | 4687.55 | 4685.5469 | 5666966 |
| 15-29 | Ur-pUr-pCr-pUr-pUr-pCr-pUr-pGr-pGr-pUr-pCr-pCr-pCr-pCr-pA | Cyclic phosphorylation | 15 | -0.11 | 100 | 1 | 12.66 | 1561.51 | -3 | 4685.5464 | 4687.58 | 4685.5469 | 7411382.5 |
| 15-29 | Ur-pUr-pCr-pUr-pUr-pCr-pUr-pGr-pGr-pUr-pCr-pCr-pCr-pCr-pA | Cyclic phosphorylation | 15 | -0.11 | 100 | 1 | 12.66 | 668.499 | -7 | 4685.5464 | 4687.56 | 4685.5469 | 14933829 |
| 15-29 | Ur-pUr-pCr-pUr-pUr-pCr-pUr-pGr-pGr-pUr-pCr-pCr-pCr-pCr-pA | Cyclic phosphorylation | 15 | -0.32 | 100 | 1 | 12.66 | 584.936 | -8 | 4685.5454 | 4687.53 | 4685.5469 | 29450080 |
| 2420-2433 | Cr-pAr-pAr-pGr-pCr-pAr-pCr-pCr-pCr-pUr-pCr-pCr-pUr-pAr | Cyclic phosphorylation | 14 | -0.1 | 100 | 1 | 12.69 | 628.935 | -7 | 4408.5967 | 4410.35 | 4408.5971 | 12227350 |
| 2420-2433 | Cr-pAr-pAr-pGr-pCr-pAr-pCr-pCr-pCr-pUr-pCr-pCr-pUr-pAr | Cyclic phosphorylation | 14 | 0.01 | 100 | 1 | 12.69 | 550.193 | -8 | 4408.5972 | 4410.51 | 4408.5971 | 8934748 |
| 2420-2433 | Cr-pAr-pAr-pGr-pCr-pAr-pCr-pCr-pCr-pUr-pCr-pCr-pUr-pAr | Cyclic phosphorylation | 14 | -0.21 | 100 | 1 | 12.71 | 1469.193 | -3 | 4408.5962 | 4410.52 | 4408.5971 | 4406891 |
| 829-843 | Cr-pAr-pGr-pCr-pUr-pGr-pGr-pUr-pGr-pCr-pCr-pGr-pCr-pCr-pGr | Cyclic phosphorylation | 15 | -0.34 | 99.8 | 1 | 12.71 | 690.941 | -7 | 4841.6338 | 4843.67 | 4841.6355 | 10348006 |
| 2356-2369 | Cr-pAr-pGr-pCr-pGr-pGr-pAr-pUr-pGr-pCr-pCr-pGr-pUr-pGr | Cyclic phosphorylation | 14 | -0.2 | 99.8 | 1 | 12.74 | 650.794 | -7 | 4560.6045 | 4562.61 | 4560.6054 | 4575945.5 |
| 1030-1044 | Ur-pCr-pGr-pUr-pGr-pCr-pGr-pGr-pUr-pGr-pCr-pCr-pCr-pCr-pA | Cyclic phosphorylation | 15 | 0.33 | 94.5 | 1 | 12.83 | 679.792 | -7 | 4763.5928 | 4765.62 | 4763.5912 | 4695447 |
| 15-29 | Ur-pUr-pCr-pUr-pUr-pCr-pUr-pGr-pGr-pUr-pCr-pCr-pCr-pCr-pA | Cyclic phosphorylation | 15 | -0.22 | 100 | 1 | 12.85 | 584.936 | -8 | 4685.5459 | 4687.55 | 4685.5469 | 5156864 |
| 1030-1044 | Ur-pCr-pGr-pUr-pGr-pCr-pGr-pGr-pUr-pGr-pCr-pCr-pCr-pCr-pA | Cyclic phosphorylation | 15 | 0.95 | 94.5 | 1 | 12.85 | 594.567 | -8 | 4763.5957 | 4765.59 | 4763.5912 | 7165116.5 |
| 2230-2244 | Ur-pCr-pUr-pGr-pCr-pCr-pCr-pUr-pGr-pUr-pGr-pUr-pCr-pCr-pA | Cyclic phosphorylation | 15 | -0.04 | 100 | 1 | 13.06 | 1574.184 | -3 | 4724.5688 | 4726.54 | 4724.5691 | 5851354.5 |
| 2230-2244 | Ur-pCr-pUr-pGr-pCr-pCr-pCr-pUr-pGr-pUr-pGr-pUr-pCr-pCr-pA | Cyclic phosphorylation | 15 | 0.16 | 100 | 1 | 13.06 | 589.814 | -8 | 4724.5698 | 4726.6 | 4724.5691 | 21065330 |
| 2230-2244 | Ur-pCr-pUr-pGr-pCr-pCr-pCr-pUr-pGr-pUr-pGr-pUr-pCr-pCr-pA | Cyclic phosphorylation | 15 | -0.15 | 100 | 1 | 13.07 | 674.074 | -7 | 4724.5684 | 4726.51 | 4724.5691 | 11855180 |
| 3470-3484 | Cr-pCr-pCr-pUr-pCr-pUr-pCr-pGr-pCr-pAr-pGr-pCr-pCr-pGr-pAr | Cyclic phosphorylation | 15 | -0.17 | 90.1 | 1 | 13.25 | 677.225 | -7 | 4745.6274 | 4747.83 | 4745.6282 | 8518538 |
| 3470-3484 | Cr-pCr-pCr-pUr-pCr-pUr-pCr-pGr-pCr-pAr-pGr-pCr-pCr-pGr-pAr | Cyclic phosphorylation | 15 | -0.07 | 90.1 | 1 | 13.25 | 592.446 | -8 | 4745.6279 | 4747.84 | 4745.6282 | 5187860 |
| 3800-3814 | Cr-pUr-pGr-pUr-pGr-pGr-pCr-pAr-pGr-pCr-pCr-pGr-pCr-pUr-pG | Cyclic phosphorylation | 15 | 0.31 | 99 | 1 | 13.43 | 691.222 | -7 | 4843.605 | 4845.66 | 4843.6035 | 3127429.25 |
| 1679-1692 | Cr-pUr-pUr-pCr-pAr-pAr-pCr-pGr-pGr-pCr-pCr-pUr-pGr-pAr | Cyclic phosphorylation | 14 | 0.29 | 100 | 1 | 13.49 | 557.316 | -8 | 4465.5835 | 4467.48 | 4465.5822 | 2429857.25 |
| 1679-1692 | Cr-pUr-pUr-pCr-pAr-pAr-pCr-pGr-pGr-pCr-pCr-pUr-pGr-pAr | Cyclic phosphorylation | 14 | 0.62 | 100 | 1 | 13.5 | 1487.854 | -3 | 4465.585 | 4467.54 | 4465.5822 | 3733025.75 |
| 1679-1692 | Cr-pUr-pUr-pCr-pAr-pAr-pCr-pGr-pGr-pCr-pCr-pUr-pGr-pAr | Cyclic phosphorylation | 14 | 0.84 | 100 | 1 | 13.5 | 743.424 | -6 | 4465.5859 | 4467.46 | 4465.5822 | 4082272.5 |
| 1679-1692 | Cr-pUr-pUr-pCr-pAr-pAr-pCr-pGr-pGr-pCr-pCr-pUr-pGr-pAr | Cyclic phosphorylation | 14 | 0.4 | 100 | 1 | 13.52 | 637.076 | -7 | 4465.584 | 4467.47 | 4465.5822 | 12683010 |
| 3280-3294 | Cr-pCr-pAr-pCr-pCr-pGr-pCr-pUr-pCr-pCr-pAr-pGr-pCr-pGr-pAr | Cyclic phosphorylation | 15 | 0.23 | 99.4 | 1 | 13.74 | 674.8 | -7 | 4728.6504 | 4730.73 | 4728.6493 | 5194264.5 |

| | | | | | | | | | | | | | |
|---|---|---|---|---|---|---|---|---|---|---|---|---|---|
| 3280-3294 | Cr-pCr-pAr-pCr-pCr-pGr-pCr-pUr-pCr-pCr-pAr-pGr-pCr-pCr-pAr | Cyclic phosphorylation | 15 | 0.44 | 99.4 | 1 | 13.76 | 590.325 | -8 | 4728.6514 | 4730.77 | 4728.6493 | 6029585 |
| 1106-1119 | Cr-pGr-pCr-pCr-pUr-pGr-pGr-pAr-pAr-pCr-pCr-pGr-pGr-pAr | Cyclic phosphorylation | 14 | -0.22 | 92.9 | 1 | 13.85 | 648.368 | -7 | 4543.6255 | 4545.64 | 4543.6265 | 9099448 |
| 1106-1119 | Cr-pGr-pCr-pCr-pUr-pGr-pGr-pAr-pAr-pCr-pCr-pGr-pGr-pAr | Cyclic phosphorylation | 14 | -0.22 | 92.9 | 1 | 13.86 | 756.431 | -6 | 4543.6255 | 4545.65 | 4543.6265 | 7912701 |
| 2634-2649 | Cr-pUr-pGr-pCr-pCr-pUr-pCr-pCr-pUr-pCr-pUr-pGr-pCr-pUr-pG | Cyclic phosphorylation | 16 | 0.29 | 98.3 | 1 | 13.86 | 558.061 | -9 | 5029.6118 | 5031.75 | 5029.6103 | 2311317 |
| 2634-2649 | Cr-pUr-pGr-pCr-pCr-pUr-pCr-pCr-pUr-pCr-pUr-pGr-pCr-pUr-pG | Cyclic phosphorylation | 16 | 0.2 | 98.3 | 1 | 13.88 | 627.945 | -8 | 5029.6113 | 5031.7 | 5029.6103 | 5680254 |
| 241-255 | Cr-pCr-pUr-pGr-pGr-pUr-pUr-pCr-pCr-pAr-pCr-pGr-pCr-pCr-pAr | Cyclic phosphorylation | 15 | 0.42 | 99.9 | 1 | 14.12 | 592.57 | -8 | 4746.6143 | 4748.62 | 4746.6123 | 8168803 |
| 241-255 | Cr-pCr-pUr-pGr-pGr-pUr-pUr-pCr-pCr-pAr-pCr-pGr-pCr-pCr-pAr | Cyclic phosphorylation | 15 | -0.2 | 99.9 | 1 | 14.12 | 677.367 | -7 | 4746.6113 | 4748.66 | 4746.6123 | 5706655 |
| 4062-4075 | Cr-pAr-pCr-pCr-pCr-pCr-pCr-pAr-pCr-pGr-pGr-pGr-pAr-pAr | Cyclic phosphorylation | 14 | 0.7 | 93.1 | 1 | 14.16 | 640.085 | -7 | 4486.6445 | 4488.69 | 4486.6414 | 4611754.5 |
| 3700-3714 | Ur-pCr-pUr-pGr-pGr-pCr-pUr-pGr-pGr-pGr-pCr-pUr-pUr-pUr-pA | Cyclic phosphorylation | 15 | 0.01 | 100 | 1 | 14.26 | 1601.516 | -3 | 4805.5654 | 4807.72 | 4805.5654 | 4779425 |
| 3700-3714 | Ur-pCr-pUr-pGr-pGr-pCr-pUr-pGr-pGr-pGr-pCr-pUr-pUr-pUr-pA | Cyclic phosphorylation | 15 | -0.29 | 100 | 1 | 14.26 | 685.645 | -7 | 4805.564 | 4807.59 | 4805.5654 | 10633691 |
| 3700-3714 | Ur-pCr-pUr-pGr-pGr-pCr-pUr-pGr-pGr-pGr-pCr-pUr-pUr-pUr-pA | Cyclic phosphorylation | 15 | -0.09 | 100 | 1 | 14.26 | 599.939 | -8 | 4805.5649 | 4807.59 | 4805.5654 | 11651434 |
| 2278-2292 | Ur-pCr-pUr-pGr-pCr-pGr-pGr-pCr-pGr-pAr-pUr-pUr-pCr-pCr-pA | Cyclic phosphorylation | 15 | 0.74 | 100 | 1 | 14.89 | 1595.529 | -3 | 4787.606 | 4789.65 | 4787.6024 | 22578052 |
| 2278-2292 | Ur-pCr-pUr-pGr-pCr-pGr-pGr-pCr-pGr-pAr-pUr-pUr-pCr-pCr-pA | Cyclic phosphorylation | 15 | 0.54 | 100 | 1 | 14.89 | 683.079 | -7 | 4787.605 | 4789.6 | 4787.6024 | 41019544 |
| 2278-2292 | Ur-pCr-pUr-pGr-pCr-pGr-pGr-pCr-pGr-pAr-pUr-pUr-pCr-pCr-pA | Cyclic phosphorylation | 15 | 0.23 | 100 | 1 | 14.89 | 597.693 | -8 | 4787.6035 | 4789.6 | 4787.6024 | 65998544 |
| 2278-2292 | Ur-pCr-pUr-pGr-pCr-pGr-pGr-pCr-pGr-pAr-pUr-pUr-pCr-pCr-pA | Cyclic phosphorylation | 15 | 0.74 | 100 | 1 | 14.89 | 797.094 | -6 | 4787.606 | 4789.6 | 4787.6024 | 9916185 |
| 702-717 | Cr-pCr-pUr-pCr-pAr-pGr-pGr-pGr-pCr-pUr-pUr-pCr-pUr-pCr-pU | Cyclic phosphorylation | 16 | 0.33 | 100 | 1 | 14.91 | 632.945 | -8 | 5069.6182 | 5071.8 | 5069.6165 | 11097491 |
| 702-717 | Cr-pCr-pUr-pCr-pAr-pGr-pGr-pGr-pCr-pUr-pUr-pCr-pUr-pCr-pU | Cyclic phosphorylation | 16 | 0.14 | 100 | 1 | 14.91 | 723.367 | -7 | 5069.6172 | 5071.78 | 5069.6165 | 8483678 |
| 2278-2292 | Ur-pCr-pUr-pGr-pCr-pGr-pGr-pCr-pGr-pAr-pUr-pUr-pCr-pCr-pA | Cyclic phosphorylation | 15 | 0.23 | 100 | 1 | 14.92 | 531.171 | -9 | 4787.6035 | 4789.61 | 4787.6024 | 12726306 |
| 1477-1492 | Cr-pCr-pGr-pGr-pCr-pAr-pGr-pCr-pAr-pCr-pCr-pCr-pCr-pUr-pUr | Cyclic phosphorylation | 16 | -0.17 | 100 | 1 | 14.98 | 635.578 | -8 | 5090.6748 | 5092.81 | 5090.6757 | 5637429 |
| 2945-2959 | Gr-pCr-pAr-pGr-pCr-pUr-pGr-pUr-pCr-pUr-pCr-pCr-pAr-pAr | Cyclic phosphorylation | 15 | 1.55 | 93.5 | 1 | 15.05 | 595.571 | -8 | 4770.6309 | 4772.97 | 4770.6235 | 12781343 |
| 2945-2959 | Gr-pCr-pAr-pGr-pCr-pUr-pGr-pUr-pCr-pUr-pCr-pCr-pAr-pAr | Cyclic phosphorylation | 15 | 1.03 | 93.5 | 1 | 15.07 | 680.796 | -7 | 4770.6284 | 4772.87 | 4770.6235 | 21715860 |
| 2945-2959 | Gr-pCr-pAr-pGr-pCr-pUr-pGr-pUr-pCr-pUr-pCr-pCr-pAr-pAr | Cyclic phosphorylation | 15 | 0.62 | 93.5 | 1 | 15.09 | 794.43 | -6 | 4770.6265 | 4772.81 | 4770.6235 | 8185963 |
| 2945-2959 | Gr-pCr-pAr-pGr-pCr-pUr-pGr-pUr-pCr-pUr-pCr-pCr-pAr-pAr | Cyclic phosphorylation | 15 | 1.34 | 93.5 | 1 | 15.12 | 1589.867 | -3 | 4770.6299 | 4772.89 | 4770.6235 | 8719264 |
| 1412-1426 | Cr-pCr-pGr-pGr-pCr-pUr-pGr-pUr-pUr-pCr-pCr-pGr-pGr-pAr-pA | Cyclic phosphorylation | 15 | 0.3 | 95.4 | 1 | 15.32 | 602.571 | -8 | 4826.626 | 4828.84 | 4826.6246 | 6772975 |
| 1412-1426 | Cr-pCr-pGr-pGr-pCr-pUr-pGr-pUr-pUr-pCr-pCr-pGr-pGr-pAr-pA | Cyclic phosphorylation | 15 | -0.01 | 95.4 | 1 | 15.32 | 688.796 | -7 | 4826.6245 | 4828.86 | 4826.6246 | 7359256 |
| 1439-1453 | Gr-pCr-pCr-pCr-pUr-pUr-pCr-pGr-pAr-pGr-pCr-pGr-pGr-pGr-pA | Cyclic phosphorylation | 15 | -0.14 | 98.1 | 1 | 15.65 | 694.371 | -7 | 4865.646 | 4868.08 | 4865.6467 | 6453388 |
| 567-582 | Ur-pCr-pCr-pCr-pAr-pGr-pCr-pCr-pUr-pUr-pCr-pCr-pUr-pG | Cyclic phosphorylation | 16 | -0.33 | 99.7 | 1 | 15.67 | 715.51 | -7 | 5013.6138 | 5015.73 | 5013.6154 | 4437953.5 |
| 567-582 | Ur-pCr-pCr-pCr-pAr-pGr-pCr-pCr-pUr-pUr-pCr-pCr-pUr-pG | Cyclic phosphorylation | 16 | -0.04 | 99.7 | 1 | 15.68 | 625.945 | -8 | 5013.6152 | 5015.72 | 5013.6154 | 11525148 |
| 2676-2692 | Ur-pCr-pUr-pGr-pCr-pCr-pCr-pUr-pGr-pCr-pUr-pGr-pGr-pCr-pC | Cyclic phosphorylation | 17 | 0.27 | 100 | 1 | 15.84 | 602.513 | -9 | 5429.6763 | 5431.95 | 5429.6748 | 5655837.5 |
| 2143-2158 | Ur-pGr-pUr-pCr-pUr-pCr-pUr-pGr-pGr-pCr-pGr-pCr-pCr-pG | Cyclic phosphorylation | 16 | 0.33 | 99.9 | 1 | 15.86 | 734.8 | -7 | 5148.6465 | 5150.81 | 5148.6448 | 6134033.5 |
| 2143-2158 | Ur-pGr-pUr-pCr-pUr-pCr-pUr-pGr-pGr-pCr-pGr-pCr-pCr-pG | Cyclic phosphorylation | 16 | 1.09 | 99.9 | 1 | 15.86 | 642.824 | -8 | 5148.6504 | 5150.8 | 5148.6448 | 6745940 |
| 2676-2692 | Ur-pCr-pUr-pGr-pCr-pCr-pCr-pUr-pGr-pCr-pUr-pGr-pGr-pCr-pC | Cyclic phosphorylation | 17 | 0.18 | 100 | 1 | 15.86 | 677.953 | -8 | 5429.6758 | 5431.94 | 5429.6748 | 10804844 |
| 3542-3556 | Cr-pGr-pUr-pGr-pGr-pAr-pCr-pCr-pUr-pGr-pGr-pCr-pGr-pA | Cyclic phosphorylation | 15 | -0.3 | 100 | 1 | 15.87 | 700.086 | -7 | 4905.6514 | 4907.73 | 4905.6528 | 26550658 |
| 3542-3556 | Cr-pGr-pUr-pGr-pGr-pAr-pCr-pCr-pUr-pGr-pGr-pCr-pGr-pA | Cyclic phosphorylation | 15 | 0.2 | 100 | 1 | 15.88 | 1634.879 | -3 | 4905.6538 | 4907.78 | 4905.6528 | 7839586.5 |
| 3542-3556 | Cr-pGr-pUr-pGr-pGr-pAr-pCr-pCr-pUr-pGr-pGr-pCr-pGr-pA | Cyclic phosphorylation | 15 | 0 | 100 | 1 | 15.89 | 816.936 | -6 | 4905.6528 | 4907.76 | 4905.6528 | 8264307 |
| 3542-3556 | Cr-pGr-pUr-pGr-pGr-pAr-pCr-pCr-pUr-pGr-pGr-pCr-pGr-pA | Cyclic phosphorylation | 15 | 0.3 | 100 | 1 | 15.89 | 612.45 | -8 | 4905.6543 | 4907.74 | 4905.6528 | 12109028 |
| 464-480 | Cr-pGr-pAr-pCr-pCr-pCr-pCr-pUr-pUr-pCr-pCr-pUr-pGr-pGr-pG | Cyclic phosphorylation | 17 | 0.34 | 100 | 1 | 16.29 | 675.83 | -8 | 5412.6978 | 5414.95 | 5412.6959 | 7702838.5 |
| 464-480 | Cr-pGr-pAr-pCr-pCr-pCr-pCr-pUr-pUr-pCr-pCr-pUr-pGr-pGr-pG | Cyclic phosphorylation | 17 | 0.16 | 100 | 1 | 16.29 | 772.521 | -7 | 5412.6968 | 5415.09 | 5412.6959 | 10494575 |
| 464-480 | Cr-pGr-pAr-pCr-pCr-pCr-pCr-pUr-pUr-pCr-pCr-pUr-pGr-pGr-pG | Cyclic phosphorylation | 17 | 0.52 | 100 | 1 | 16.31 | 600.515 | -9 | 5412.6987 | 5414.46 | 5412.6959 | 702825.75 |
| 3275-3290 | Ur-pUr-pUr-pCr-pAr-pCr-pCr-pAr-pCr-pGr-pCr-pUr-pCr-pCr | Cyclic phosphorylation | 16 | 0.04 | 100 | 1 | 16.48 | 713.084 | -7 | 4996.6367 | 4998.77 | 4996.6365 | 4648415 |
| 1001-1015 | Cr-pAr-pAr-pCr-pUr-pUr-pCr-pGr-pGr-pUr-pGr-pCr-pCr-pA | Cyclic phosphorylation | 15 | 0.36 | 95 | 1 | 16.49 | 686.512 | -7 | 4810.6313 | 4812.78 | 4810.6296 | 3897483.75 |
| 3275-3290 | Ur-pUr-pUr-pCr-pAr-pCr-pCr-pAr-pCr-pGr-pCr-pUr-pCr-pCr | Cyclic phosphorylation | 16 | 0.14 | 100 | 1 | 16.49 | 623.823 | -8 | 4996.6372 | 4998.77 | 4996.6365 | 12921336 |
| 1001-1015 | Cr-pAr-pAr-pCr-pUr-pUr-pCr-pGr-pGr-pUr-pGr-pCr-pCr-pA | Cyclic phosphorylation | 15 | 0.46 | 95 | 1 | 16.51 | 600.572 | -8 | 4810.6318 | 4812.72 | 4810.6296 | 5733656 |
| 3275-3290 | Ur-pUr-pUr-pCr-pAr-pCr-pCr-pAr-pCr-pGr-pCr-pUr-pCr-pCr | Cyclic phosphorylation | 16 | -0.05 | 100 | 1 | 16.51 | 1665.206 | -3 | 4996.6362 | 4998.78 | 4996.6365 | 4728551 |
| 3275-3290 | Ur-pUr-pUr-pCr-pAr-pCr-pCr-pAr-pCr-pGr-pCr-pUr-pCr-pCr | Cyclic phosphorylation | 16 | 0.63 | 100 | 1 | 16.51 | 554.398 | -9 | 4996.6396 | 4998.77 | 4996.6365 | 9668712 |
| 3527-3541 | Cr-pCr-pAr-pCr-pAr-pCr-pAr-pAr-pGr-pCr-pCr-pCr-pGr-pAr | Cyclic phosphorylation | 15 | 0.66 | 100 | 1 | 16.62 | 1591.557 | -3 | 4775.6909 | 4777.8 | 4775.6878 | 3482646.75 |
| 4054-4069 | Cr-pCr-pUr-pAr-pGr-pCr-pCr-pAr-pCr-pAr-pCr-pCr-pCr-pCr- | Cyclic phosphorylation | 16 | 0.22 | 95.6 | 1.1 | 16.63 | 626.456 | -8 | 5017.6968 | 5019.84 | 5017.6957 | 2844315.5 |
| 3527-3541 | Cr-pCr-pAr-pCr-pAr-pCr-pAr-pAr-pGr-pCr-pCr-pCr-pGr-pAr | Cyclic phosphorylation | 15 | 1.58 | 100 | 1 | 16.63 | 596.204 | -8 | 4775.6953 | 4777.8 | 4775.6878 | 5847341 |
| 3527-3541 | Cr-pCr-pAr-pCr-pAr-pCr-pAr-pAr-pGr-pCr-pCr-pCr-pGr-pAr | Cyclic phosphorylation | 15 | 0.97 | 100 | 1 | 16.63 | 681.52 | -7 | 4775.6924 | 4777.84 | 4775.6878 | 7713719.5 |
| 3467-3483 | Cr-pGr-pAr-pCr-pCr-pCr-pUr-pCr-pUr-pGr-pCr-pAr-pGr-pCr-pC | Cyclic phosphorylation | 17 | 0.06 | 100 | 1 | 16.84 | 770.096 | -7 | 5395.7173 | 5398.18 | 5395.717 | 2030506.25 |
| 3091-3105 | Ur-pCr-pAr-pGr-pAr-pGr-pCr-pCr-pGr-pCr-pGr-pAr-pGr-pA | Cyclic phosphorylation | 15 | 0.54 | 94.2 | 1 | 17.07 | 1623.888 | -3 | 4872.6816 | 4874.86 | 4872.679 | 2088479.75 |
| 3091-3105 | Ur-pCr-pAr-pGr-pAr-pGr-pCr-pCr-pGr-pCr-pGr-pAr-pGr-pA | Cyclic phosphorylation | 15 | 0.64 | 94.2 | 1 | 17.07 | 608.328 | -8 | 4872.6821 | 4874.8 | 4872.679 | 4891455 |
| 3091-3105 | Ur-pCr-pAr-pGr-pAr-pGr-pCr-pCr-pGr-pCr-pGr-pAr-pGr-pA | Cyclic phosphorylation | 15 | 0.24 | 94.2 | 1 | 17.07 | 695.376 | -7 | 4872.6802 | 4874.81 | 4872.679 | 3959276.75 |
| 2358-2372 | Gr-pGr-pGr-pAr-pUr-pCr-pGr-pCr-pCr-pGr-pUr-pGr-pGr-pAr-pA | Cyclic phosphorylation | 15 | 0.4 | 96.6 | 1 | 17.34 | 703.517 | -7 | 4929.666 | 4931.81 | 4929.6641 | 1939242 |
| 2715-2730 | Ur-pUr-pUr-pGr-pGr-pAr-pGr-pCr-pAr-pGr-pGr-pCr-pGr-pCr-pC | Cyclic phosphorylation | 16 | 0.39 | 100 | 1 | 17.36 | 743.805 | -7 | 5211.6802 | 5213.84 | 5211.6781 | 6063096 |
| 2715-2730 | Ur-pUr-pUr-pGr-pGr-pAr-pGr-pCr-pAr-pGr-pGr-pCr-pGr-pCr-pC | Cyclic phosphorylation | 16 | 0.58 | 100 | 1 | 17.36 | 650.703 | -8 | 5211.6812 | 5213.89 | 5211.6781 | 8664463 |
| 2715-2730 | Ur-pUr-pUr-pGr-pGr-pAr-pGr-pCr-pAr-pGr-pGr-pCr-pGr-pCr-pC | Cyclic phosphorylation | 16 | -0.17 | 100 | 1.3 | 17.41 | 578.18 | -9 | 5211.6772 | 5213.78 | 5211.6781 | 350448.19 |
| 200-215 | Cr-pUr-pCr-pUr-pAr-pCr-pCr-pCr-pAr-pGr-pGr-pAr-pCr-pCr-pUr | Cyclic phosphorylation | 16 | 0.05 | 100 | 1 | 17.46 | 563.178 | -9 | 5075.665 | 5077.88 | 5075.6648 | 5239363 |
| 2854-2868 | Ur-pCr-pCr-pAr-pGr-pGr-pAr-pCr-pAr-pGr-pCr-pCr-pUr-pGr-pA | Cyclic phosphorylation | 15 | 0.18 | 100 | 1 | 17.48 | 689.801 | -7 | 4833.6577 | 4835.81 | 4833.6569 | 12323253 |
| 2854-2868 | Ur-pCr-pCr-pAr-pGr-pGr-pAr-pCr-pAr-pGr-pCr-pCr-pUr-pGr-pA | Cyclic phosphorylation | 15 | 0.48 | 100 | 1 | 17.48 | 603.45 | -8 | 4833.6592 | 4835.79 | 4833.6569 | 13534530 |
| 2854-2868 | Ur-pCr-pCr-pAr-pGr-pGr-pAr-pCr-pAr-pGr-pCr-pCr-pUr-pGr-pA | Cyclic phosphorylation | 15 | 0.38 | 100 | 1 | 17.48 | 1610.88 | -3 | 4833.6587 | 4835.86 | 4833.6569 | 5453567.5 |
| 2854-2868 | Ur-pCr-pCr-pAr-pGr-pGr-pAr-pCr-pAr-pGr-pCr-pCr-pUr-pGr-pA | Cyclic phosphorylation | 15 | 0.48 | 100 | 1 | 17.48 | 804.77 | -6 | 4833.6592 | 4835.9 | 4833.6569 | 2401525 |
| 3907-3924 | Cr-pUr-pAr-pGr-pCr-pUr-pGr-pCr-pCr-pCr-pCr-pUr-pUr-pCr | Cyclic phosphorylation | 18 | 0.17 | 99.6 | 1 | 17.52 | 704.33 | -8 | 5639.6938 | 5642.19 | 5639.6929 | 1271858.75 |
| 200-215 | Cr-pUr-pCr-pUr-pAr-pCr-pCr-pCr-pAr-pGr-pGr-pAr-pCr-pCr-pUr | Cyclic phosphorylation | 16 | 0.44 | 100 | 1 | 17.57 | 1691.549 | -3 | 5075.667 | 5078.09 | 5075.6648 | 4427307 |
| 200-215 | Cr-pUr-pCr-pUr-pAr-pCr-pCr-pCr-pAr-pGr-pGr-pAr-pCr-pCr-pUr | Cyclic phosphorylation | 16 | 0.24 | 100 | 1 | 17.57 | 724.374 | -7 | 5075.666 | 5077.93 | 5075.6648 | 6008283.5 |
| 200-215 | Cr-pUr-pCr-pUr-pAr-pCr-pCr-pCr-pAr-pGr-pGr-pAr-pCr-pCr-pUr | Cyclic phosphorylation | 16 | 0.63 | 100 | 1 | 17.57 | 633.701 | -8 | 5075.668 | 5077.88 | 5075.6648 | 14036081 |
| 4062-4076 | Cr-pAr-pCr-pCr-pCr-pCr-pCr-pAr-pCr-pGr-pGr-pGr-pAr-pAr-pAr | Cyclic phosphorylation | 15 | 0.9 | 100 | 1 | 17.91 | 601.205 | -8 | 4815.6982 | 4818.15 | 4815.6939 | 2564114.25 |
| 4062-4076 | Cr-pAr-pCr-pCr-pCr-pCr-pCr-pAr-pCr-pGr-pGr-pGr-pAr-pAr-pAr | Cyclic phosphorylation | 15 | 0.49 | 100 | 1 | 17.95 | 1604.892 | -3 | 4815.6963 | 4817.95 | 4815.6939 | 2079909.38 |
| 4062-4076 | Cr-pAr-pCr-pCr-pCr-pCr-pCr-pAr-pCr-pGr-pGr-pGr-pAr-pAr-pAr | Cyclic phosphorylation | 15 | -0.01 | 100 | 1 | 17.95 | 687.235 | -7 | 4815.6938 | 4817.84 | 4815.6939 | 5872897 |
| 3951-3966 | Cr-pCr-pUr-pCr-pGr-pGr-pGr-pUr-pCr-pCr-pCr-pAr-pGr-pGr-pU | Cyclic phosphorylation | 16 | 0.22 | 99.4 | 1 | 18.04 | 732.374 | -7 | 5131.667 | 5133.87 | 5131.6658 | 3242547.5 |
| 3951-3966 | Cr-pCr-pUr-pCr-pGr-pGr-pGr-pUr-pCr-pCr-pCr-pAr-pGr-pGr-pU | Cyclic phosphorylation | 16 | 0.51 | 99.4 | 1 | 18.04 | 640.701 | -8 | 5131.6685 | 5133.89 | 5131.6658 | 8112439.5 |
| 3951-3966 | Cr-pCr-pUr-pCr-pGr-pGr-pGr-pUr-pCr-pCr-pCr-pAr-pGr-pGr-pU | Cyclic phosphorylation | 16 | 0.51 | 99.4 | 1 | 18.05 | 569.401 | -9 | 5131.6685 | 5133.89 | 5131.6658 | 2410171.25 |
| 120-136 | Cr-pAr-pGr-pCr-pUr-pGr-pCr-pCr-pUr-pCr-pCr-pAr-pGr-pCr-pC | Cyclic phosphorylation | 17 | 0.36 | 99.9 | 1 | 18.31 | 671.832 | -8 | 5380.708 | 5382.97 | 5380.7061 | 6671738.5 |
| 120-136 | Cr-pAr-pGr-pCr-pUr-pGr-pCr-pCr-pUr-pCr-pCr-pAr-pGr-pCr-pC | Cyclic phosphorylation | 17 | 0.36 | 99.9 | 1 | 18.31 | 597.072 | -9 | 5380.708 | 5382.99 | 5380.7061 | 6896100.5 |
| 120-136 | Cr-pAr-pGr-pCr-pUr-pGr-pCr-pCr-pUr-pCr-pCr-pAr-pGr-pCr-pC | Cyclic phosphorylation | 17 | 0.36 | 99.9 | 1.1 | 18.31 | 1793.23 | -3 | 5380.708 | 5382.98 | 5380.7061 | 2628230.5 |
| 205-221 | Cr-pCr-pCr-pAr-pGr-pGr-pAr-pCr-pCr-pUr-pGr-pUr-pCr-pCr | Cyclic phosphorylation | 17 | -0.08 | 96.1 | 1 | 18.6 | 673.954 | -8 | 5397.6846 | 5400.1 | 5397.685 | 11466443 |
| 205-221 | Cr-pCr-pCr-pAr-pGr-pGr-pAr-pCr-pCr-pUr-pGr-pUr-pCr-pCr | Cyclic phosphorylation | 17 | 0.19 | 96.1 | 1 | 18.61 | 598.958 | -9 | 5397.686 | 5399.95 | 5397.685 | 11621513 |
| 682-697 | Ur-pCr-pAr-pAr-pCr-pCr-pUr-pCr-pGr-pUr-pGr-pCr-pGr-pGr-pG | Cyclic phosphorylation | 16 | 1.55 | 100 | 1 | 18.69 | 572.069 | -9 | 5155.6851 | 5157.83 | 5155.6771 | 7852328 |
| 682-697 | Ur-pCr-pAr-pAr-pCr-pCr-pUr-pCr-pGr-pUr-pGr-pCr-pGr-pGr-pG | Cyclic phosphorylation | 16 | 0.89 | 100 | 1 | 18.7 | 1718.221 | -3 | 5155.6816 | 5157.87 | 5155.6771 | 14030589 |
| 682-697 | Ur-pCr-pAr-pAr-pCr-pCr-pUr-pCr-pGr-pUr-pGr-pCr-pGr-pGr-pG | Cyclic phosphorylation | 16 | 0.03 | 100 | 1 | 18.7 | 643.702 | -8 | 5155.6772 | 5157.84 | 5155.6771 | 47746436 |
| 682-697 | Ur-pCr-pAr-pAr-pCr-pCr-pUr-pCr-pGr-pUr-pGr-pCr-pGr-pGr-pG | Cyclic phosphorylation | 16 | 0.51 | 100 | 1 | 18.71 | 1288.413 | -4 | 5155.6797 | 5157.88 | 5155.6771 | 6555524.5 |
| 682-697 | Ur-pCr-pAr-pAr-pCr-pCr-pUr-pCr-pGr-pUr-pGr-pCr-pGr-pGr-pG | Cyclic phosphorylation | 16 | 0.51 | 100 | 1 | 18.71 | 858.607 | -6 | 5155.6797 | 5157.86 | 5155.6771 | 7085054.5 |
| 682-697 | Ur-pCr-pAr-pAr-pCr-pCr-pUr-pCr-pGr-pUr-pGr-pCr-pGr-pGr-pG | Cyclic phosphorylation | 16 | 0.22 | 100 | 1 | 18.71 | 735.804 | -7 | 5155.6782 | 5157.83 | 5155.6771 | 28244104 |
| 1055-1072 | Ur-pCr-pUr-pGr-pUr-pGr-pCr-pCr-pCr-pCr-pUr-pUr-pCr-pGr-pG | Cyclic phosphorylation | 18 | 0.92 | 100 | 1 | 18.96 | 634.738 | -9 | 5719.7104 | 5722.16 | 5719.7052 | 52564572 |
| 1055-1072 | Ur-pCr-pUr-pGr-pUr-pGr-pCr-pCr-pCr-pCr-pUr-pUr-pCr-pGr-pG | Cyclic phosphorylation | 18 | 1.26 | 100 | 1.1 | 18.98 | 1906.231 | -3 | 5719.7124 | 5722.17 | 5719.7052 | 9835516 |
| 1055-1072 | Ur-pCr-pUr-pGr-pUr-pGr-pCr-pCr-pCr-pCr-pUr-pUr-pCr-pGr-pG | Cyclic phosphorylation | 18 | 0.66 | 100 | 1 | 18.98 | 1429.42 | -4 | 5719.709 | 5722.17 | 5719.7052 | 8074039.5 |
| 1055-1072 | Ur-pCr-pUr-pGr-pUr-pGr-pCr-pCr-pCr-pCr-pUr-pUr-pCr-pGr-pG | Cyclic phosphorylation | 18 | 0.75 | 100 | 1 | 18.98 | 571.164 | -10 | 5719.7095 | 5722.21 | 5719.7052 | 9486761 |
| 1055-1072 | Ur-pCr-pUr-pGr-pUr-pGr-pCr-pCr-pCr-pCr-pUr-pUr-pCr-pGr-pG | Cyclic phosphorylation | 18 | 0.66 | 100 | 1 | 18.98 | 714.206 | -8 | 5719.709 | 5722.15 | 5719.7052 | 32774416 |
| 1055-1072 | Ur-pCr-pUr-pGr-pUr-pGr-pCr-pCr-pCr-pCr-pUr-pUr-pCr-pGr-pG | Cyclic phosphorylation | 18 | 0.32 | 100 | 1 | 18.98 | 816.38 | -7 | 5719.707 | 5722.17 | 5719.7052 | 8523829 |
| 2971-2986 | Ur-pCr-pAr-pGr-pCr-pUr-pCr-pUr-pGr-pUr-pGr-pCr-pUr-pGr-pA | Cyclic phosphorylation | 16 | 0.42 | 100 | 1 | 19.17 | 1705.542 | -3 | 5117.6411 | 5119.83 | 5117.639 | 6024453.5 |
| 2971-2986 | Ur-pCr-pAr-pGr-pCr-pUr-pCr-pUr-pGr-pUr-pGr-pCr-pUr-pGr-pA | Cyclic phosphorylation | 16 | 0.04 | 100 | 1 | 19.17 | 638.948 | -8 | 5117.6392 | 5119.81 | 5117.639 | 21698596 |
| 2971-2986 | Ur-pCr-pAr-pGr-pCr-pUr-pCr-pUr-pGr-pUr-pGr-pCr-pUr-pGr-pA | Cyclic phosphorylation | 16 | 0.61 | 100 | 1.1 | 19.17 | 567.731 | -9 | 5117.6421 | 5119.77 | 5117.639 | 5734545.5 |
| 2971-2986 | Ur-pCr-pAr-pGr-pCr-pUr-pCr-pUr-pGr-pUr-pGr-pCr-pUr-pGr-pA | Cyclic phosphorylation | 16 | 0.14 | 100 | 1 | 19.17 | 730.37 | -7 | 5117.6396 | 5119.77 | 5117.639 | 6261229.5 |
| 2356-2371 | Cr-pAr-pGr-pGr-pGr-pAr-pUr-pCr-pGr-pCr-pCr-pGr-pUr-pGr-pG | Cyclic phosphorylation | 16 | -0.05 | 100 | 1 | 19.2 | 747.094 | -7 | 5234.7051 | 5236.9 | 5234.7054 | 5399441 |
| 2356-2371 | Cr-pAr-pGr-pGr-pGr-pAr-pUr-pCr-pGr-pCr-pCr-pGr-pUr-pGr-pG | Cyclic phosphorylation | 16 | 0.04 | 100 | 1 | 19.22 | 653.581 | -8 | 5234.7056 | 5236.93 | 5234.7054 | 11285134 |
| 2356-2371 | Cr-pAr-pGr-pGr-pGr-pAr-pUr-pCr-pGr-pCr-pCr-pGr-pUr-pGr-pG | Cyclic phosphorylation | 16 | 0.41 | 100 | 1 | 19.24 | 580.85 | -9 | 5234.7075 | 5236.89 | 5234.7054 | 5109361 |
| 973-987 | Cr-pCr-pGr-pUr-pGr-pGr-pAr-pAr-pAr-pAr-pGr-pGr-pCr-pCr-pA | Cyclic phosphorylation | 15 | 0.58 | 99.7 | 1 | 19.39 | 616.33 | -8 | 4936.6992 | 4938.81 | 4936.6964 | 3484325.5 |
| 973-987 | Cr-pCr-pGr-pUr-pGr-pGr-pAr-pAr-pAr-pAr-pGr-pGr-pCr-pCr-pA | Cyclic phosphorylation | 15 | 0.18 | 99.7 | 1 | 19.39 | 704.521 | -7 | 4936.6973 | 4938.82 | 4936.6964 | 3833395.75 |
| 1337-1353 | Cr-pUr-pCr-pCr-pAr-pCr-pGr-pCr-pUr-pCr-pGr-pUr-pGr-pU | Cyclic phosphorylation | 17 | 0.74 | 95.3 | 1 | 19.65 | 679.078 | -8 | 5438.6792 | 5440.98 | 5438.6752 | 2060028.38 |
| 317-333 | Cr-pGr-pGr-pGr-pUr-pGr-pUr-pAr-pCr-pUr-pUr-pUr-pGr-p( | Cyclic phosphorylation | 17 | 1.04 | 99 | 1 | 19.96 | 684.079 | -8 | 5478.687 | 5480.86 | 5478.6813 | 3816186.75 |
| 2731-2748 | Cr-pUr-pCr-pUr-pGr-pGr-pCr-pAr-pGr-pAr-pUr-pCr-pCr-pCr-pUr | Cyclic phosphorylation | 18 | 0.31 | 99.9 | 1 | 20.04 | 707.329 | -8 | 5664.6899 | 5667.03 | 5664.6882 | 5028826 |
| 2731-2748 | Cr-pUr-pCr-pUr-pGr-pGr-pCr-pAr-pGr-pAr-pUr-pCr-pCr-pCr-pUr | Cyclic phosphorylation | 18 | 0.57 | 99.9 | 1 | 20.05 | 628.625 | -9 | 5664.6914 | 5667.11 | 5664.6882 | 4561642.5 |
| 3467-3484 | Cr-pGr-pAr-pCr-pCr-pCr-pUr-pCr-pUr-pGr-pCr-pAr-pGr-pCr-pCr | Cyclic phosphorylation | 18 | 0.86 | 100 | 1 | 20.34 | 635.411 | -9 | 5724.7744 | 5727.71 | 5724.7695 | 2456951.25 |
| 3467-3484 | Cr-pGr-pAr-pCr-pCr-pCr-pUr-pCr-pUr-pGr-pCr-pAr-pGr-pCr-pCr | Cyclic phosphorylation | 18 | 0.01 | 100 | 1 | 20.37 | 714.839 | -8 | 5724.7695 | 5727.44 | 5724.7695 | 11838501 |
| 3467-3484 | Cr-pGr-pAr-pCr-pCr-pCr-pUr-pCr-pUr-pGr-pCr-pAr-pGr-pCr-pCr | Cyclic phosphorylation | 18 | 0.95 | 100 | 1 | 20.37 | 1907.918 | -3 | 5724.7749 | 5727.47 | 5724.7695 | 3148505.5 |
| 3467-3484 | Cr-pGr-pAr-pCr-pCr-pCr-pUr-pCr-pUr-pGr-pCr-pAr-pGr-pCr-pCr | Cyclic phosphorylation | 18 | -0.08 | 100 | 1 | 20.37 | 817.103 | -7 | 5724.769 | 5727.35 | 5724.7695 | 8987161 |
| 1475-1492 | Gr-pGr-pCr-pCr-pGr-pGr-pCr-pAr-pGr-pCr-pAr-pCr-pCr-pCr | Cyclic phosphorylation | 18 | -0.68 | 100 | 1 | 20.39 | 825.247 | -7 | 5780.7666 | 5783.35 | 5780.7705 | 3120158.5 |
| 1475-1492 | Gr-pGr-pCr-pCr-pGr-pGr-pCr-pAr-pGr-pCr-pAr-pCr-pCr-pCr | Cyclic phosphorylation | 18 | -0.26 | 100 | 1 | 20.4 | 721.84 | -8 | 5780.769 | 5783.28 | 5780.7705 | 3622825 |
| 3997-4013 | Cr-pCr-pUr-pCr-pUr-pGr-pCr-pUr-pAr-pGr-pUr-pCr-pCr-pA | Cyclic phosphorylation | 17 | 0.13 | 99.1 | 1 | 20.42 | 672.078 | -8 | 5382.6748 | 5384.92 | 5382.6741 | 3919864.75 |
| 3997-4013 | Cr-pCr-pUr-pCr-pUr-pGr-pCr-pUr-pAr-pGr-pUr-pCr-pCr-pA | Cyclic phosphorylation | 17 | -0.14 | 99.1 | 1 | 20.42 | 597.291 | -9 | 5382.6733 | 5384.95 | 5382.6741 | 2775330.25 |
| 1879-1894 | Cr-pAr-pGr-pUr-pGr-pCr-pUr-pGr-pUr-pAr-pCr-pCr-pAr-pGr-pG | Cyclic phosphorylation | 16 | 0.79 | 99.4 | 1 | 20.46 | 646.705 | -8 | 5179.6924 | 5181.83 | 5179.6883 | 4765599 |
| 1879-1894 | Cr-pAr-pGr-pUr-pGr-pCr-pUr-pGr-pUr-pAr-pCr-pCr-pAr-pGr-pG | Cyclic phosphorylation | 16 | 0.69 | 99.4 | 1 | 20.46 | 739.235 | -7 | 5179.6919 | 5181.82 | 5179.6883 | 3343722 |
| 463-480 | Ar-pCr-pGr-pAr-pCr-pCr-pCr-pCr-pUr-pUr-pCr-pCr-pUr-pGr-pGr | Cyclic phosphorylation | 18 | 0.36 | 99.9 | 1 | 20.76 | 637.188 | -9 | 5741.7505 | 5744.33 | 5741.7484 | 4556206 |
| 463-480 | Ar-pCr-pGr-pAr-pCr-pCr-pCr-pCr-pUr-pUr-pCr-pCr-pUr-pGr-pGr | Cyclic phosphorylation | 18 | -0.06 | 99.9 | 1 | 20.76 | 716.962 | -8 | 5741.748 | 5744.28 | 5741.7484 | 4602528 |
| 2090-2106 | Cr-pAr-pGr-pCr-pCr-pCr-pUr-pCr-pGr-pGr-pAr-pGr-pAr-pGr-pCr | Cyclic phosphorylation | 17 | 0.03 | 100 | 1 | 20.79 | 608.411 | -9 | 5482.7617 | 5485.09 | 5482.7616 | 11381752 |
| 2090-2106 | Cr-pAr-pGr-pCr-pCr-pCr-pUr-pCr-pGr-pGr-pAr-pGr-pAr-pGr-pCr | Cyclic phosphorylation | 17 | -0.15 | 100 | 1 | 20.79 | 684.588 | -8 | 5482.7607 | 5485.24 | 5482.7616 | 22243324 |
| 2090-2106 | Cr-pAr-pGr-pCr-pCr-pCr-pUr-pCr-pGr-pGr-pAr-pGr-pAr-pGr-pCr | Cyclic phosphorylation | 17 | 0.39 | 100 | 1 | 20.79 | 782.53 | -7 | 5482.7637 | 5485.25 | 5482.7616 | 9417850 |

| | | | | | | | | | | | | | |
|---|---|---|---|---|---|---|---|---|---|---|---|---|---|
| 2090-2106 | Cr-pAr-pGr-pCr-pCr-pCr-pUr-pCr-pGr-pGr-pAr-pGr-pAr-pGr-pCr | Cyclic phosphorylation | 17 | 0.65 | 100 | 1 | 20.81 | 1827.249 | -3 | 5482.7651 | 5485.25 | 5482.7616 | 6152451.5 |
| 1000-1016 | Gr-pCr-pAr-pAr-pCr-pUr-pUr-pCr-pCr-pGr-pGr-pGr-pUr-pGr-pC | Cyclic phosphorylation | 17 | 0.91 | 100 | 1 | 20.84 | 686.834 | -8 | 5500.7295 | 5503.19 | 5500.7245 | 9145309 |
| 1000-1016 | Gr-pCr-pAr-pAr-pCr-pUr-pUr-pCr-pCr-pGr-pGr-pGr-pUr-pGr-pC | Cyclic phosphorylation | 17 | 0.2 | 100 | 1 | 20.84 | 785.097 | -7 | 5500.7256 | 5503.16 | 5500.7245 | 4998770 |
| 1693-1710 | Cr-pCr-pGr-pGr-pCr-pAr-pCr-pCr-pGr-pGr-pCr-pGr-pUr-pGr-pCr | Cyclic phosphorylation | 18 | 0.11 | 99.2 | 1 | 20.9 | 645.969 | -9 | 5820.7773 | 5823.38 | 5820.7767 | 6456175 |
| 1693-1710 | Cr-pCr-pGr-pGr-pCr-pAr-pCr-pCr-pGr-pGr-pCr-pGr-pUr-pGr-pCr | Cyclic phosphorylation | 18 | 0.53 | 99.2 | 1 | 20.91 | 581.271 | -10 | 5820.7798 | 5823.2 | 5820.7767 | 3113179.25 |
| 1693-1710 | Cr-pCr-pGr-pGr-pCr-pAr-pCr-pCr-pGr-pGr-pCr-pGr-pUr-pGr-pCr | Cyclic phosphorylation | 18 | 0.03 | 99.2 | 1 | 20.91 | 726.84 | -8 | 5820.7769 | 5823.27 | 5820.7767 | 6924168 |
| 419-435 | Cr-pGr-pCr-pCr-pAr-pCr-pCr-pAr-pAr-pCr-pGr-pUr-pGr-pGr-pUr | Cyclic phosphorylation | 17 | -0.12 | 100 | 1 | 20.95 | 679.711 | -8 | 5443.7388 | 5446.23 | 5443.7394 | 8193638 |
| 419-435 | Cr-pGr-pCr-pCr-pAr-pCr-pCr-pAr-pAr-pCr-pGr-pUr-pGr-pGr-pUr | Cyclic phosphorylation | 17 | -0.21 | 100 | 1 | 20.96 | 776.956 | -7 | 5443.7383 | 5446.02 | 5443.7394 | 6731188.5 |
| 3216-3234 | Ur-pCr-pUr-pGr-pCr-pCr-pCr-pCr-pUr-pCr-pAr-pCr-pGr-pGr-pCr | Cyclic phosphorylation | 19 | -0.01 | 98.4 | 1 | 21.02 | 757.214 | -8 | 6063.7686 | 6066.52 | 6063.7686 | 12446242 |
| 3216-3234 | Ur-pCr-pUr-pGr-pCr-pCr-pCr-pCr-pUr-pCr-pAr-pCr-pGr-pGr-pCr | Cyclic phosphorylation | 19 | -0.01 | 98.4 | 1 | 21.03 | 1515.435 | -4 | 6063.7686 | 6066.57 | 6063.7686 | 4682287.5 |
| 3216-3234 | Ur-pCr-pUr-pGr-pCr-pCr-pCr-pCr-pUr-pCr-pAr-pCr-pGr-pGr-pCr | Cyclic phosphorylation | 19 | 0.39 | 98.4 | 1 | 21.03 | 672.967 | -9 | 6063.771 | 6066.56 | 6063.7686 | 22496074 |
| 3216-3234 | Ur-pCr-pUr-pGr-pCr-pCr-pCr-pCr-pUr-pCr-pAr-pCr-pGr-pGr-pCr | Cyclic phosphorylation | 19 | 0.55 | 98.4 | 1 | 21.07 | 605.57 | -10 | 6063.772 | 6066.69 | 6063.7686 | 14070961 |
| 1602-1620 | Cr-pUr-pGr-pCr-pUr-pGr-pCr-pAr-pUr-pGr-pCr-pCr-pCr-pCr-pUr | Cyclic phosphorylation | 19 | 0.82 | 100 | 1 | 21.15 | 599.971 | -10 | 6007.7725 | 6010.36 | 6007.7676 | 6109739 |
| 1602-1620 | Cr-pUr-pGr-pCr-pUr-pGr-pCr-pAr-pUr-pGr-pCr-pCr-pCr-pCr-pUr | Cyclic phosphorylation | 19 | 1.3 | 100 | 1 | 21.17 | 666.746 | -9 | 6007.7754 | 6010.35 | 6007.7676 | 8461960 |
| 1602-1620 | Cr-pUr-pGr-pCr-pUr-pGr-pCr-pAr-pUr-pGr-pCr-pCr-pCr-pCr-pUr | Cyclic phosphorylation | 19 | 0.9 | 100 | 1 | 21.2 | 750.215 | -8 | 6007.7729 | 6010.33 | 6007.7676 | 3986514.75 |
| 3003-3021 | Cr-pUr-pGr-pGr-pAr-pCr-pCr-pCr-pUr-pCr-pCr-pUr-pGr-pAr-pGr | Cyclic phosphorylation | 19 | 0.41 | 100 | 1 | 21.65 | 675.526 | -9 | 6086.7983 | 6089.4 | 6086.7958 | 4375160 |
| 3003-3021 | Cr-pUr-pGr-pGr-pAr-pCr-pCr-pCr-pUr-pCr-pCr-pUr-pGr-pAr-pGr | Cyclic phosphorylation | 19 | 0.73 | 100 | 1.1 | 21.66 | 607.973 | -10 | 6086.8003 | 6089.46 | 6086.7958 | 922130.69 |
| 3003-3021 | Cr-pUr-pGr-pGr-pAr-pCr-pCr-pCr-pUr-pCr-pCr-pUr-pGr-pAr-pGr | Cyclic phosphorylation | 19 | 0.25 | 100 | 1 | 21.67 | 760.218 | -8 | 6086.7974 | 6089.47 | 6086.7958 | 1476596.38 |
| 2111-2127 | Cr-pGr-pUr-pGr-pGr-pCr-pCr-pAr-pGr-pCr-pCr-pAr-pGr-pAr-pGr | Cyclic phosphorylation | 17 | 0.06 | 98.8 | 1 | 21.67 | 689.589 | -8 | 5522.7681 | 5525.23 | 5522.7677 | 2045970.62 |
| 1906-1923 | Cr-pCr-pGr-pAr-pAr-pGr-pUr-pGr-pCr-pCr-pCr-pGr-pUr-pGr-pG | Cyclic phosphorylation | 18 | 0.41 | 95.2 | 1 | 21.74 | 724.841 | -8 | 5804.7842 | 5807.4 | 5804.7818 | 4807893 |
| 4123-4139 | Ur-pAr-pCr-pUr-pAr-pAr-pCr-pCr-pCr-pCr-pAr-pGr-pGr-pGr-pUr | Cyclic phosphorylation | 17 | 1.63 | 97.9 | 1 | 21.74 | 679.957 | -8 | 5445.7163 | 5447.86 | 5445.7075 | 3855903.75 |
| 1906-1923 | Cr-pCr-pGr-pAr-pAr-pGr-pUr-pGr-pCr-pCr-pCr-pGr-pUr-pGr-pG | Cyclic phosphorylation | 18 | 0.5 | 95.2 | 1 | 21.75 | 644.191 | -9 | 5804.7847 | 5807.37 | 5804.7818 | 3108537.75 |
| 392-408 | Gr-pAr-pCr-pCr-pCr-pAr-pGr-pAr-pGr-pCr-pCr-pUr-pGr-pCr-pUr | Cyclic phosphorylation | 17 | 0.36 | 99.9 | 1 | 21.88 | 608.521 | -9 | 5483.7476 | 5486.2 | 5483.7456 | 3163371 |
| 3407-3424 | Cr-pAr-pAr-pCr-pAr-pCr-pCr-pUr-pUr-pCr-pGr-pUr-pGr-pUr-pCr | Cyclic phosphorylation | 18 | 1.9 | 100 | 1 | 22.09 | 715.209 | -8 | 5727.7324 | 5730.13 | 5727.7215 | 4177306.25 |
| 392-408 | Gr-pAr-pCr-pCr-pCr-pAr-pGr-pAr-pGr-pCr-pCr-pUr-pGr-pCr-pUr | Cyclic phosphorylation | 17 | 0.63 | 99.9 | 1 | 22.12 | 684.711 | -8 | 5483.749 | 5486.34 | 5483.7456 | 4641626.5 |
| 392-408 | Gr-pAr-pCr-pCr-pCr-pAr-pGr-pAr-pGr-pCr-pCr-pUr-pGr-pCr-pUr | Cyclic phosphorylation | 17 | 0.54 | 99.9 | 1 | 22.17 | 782.671 | -7 | 5483.7485 | 5486.27 | 5483.7456 | 2941643.5 |
| 2075-2089 | Cr-pCr-pAr-pGr-pAr-pCr-pAr-pCr-pAr-pGr-pAr-pCr-pAr-pAr-pAr | Cyclic phosphorylation | 15 | 0.35 | 98.3 | 1.1 | 22.31 | 605.084 | -8 | 4847.7231 | 4849.83 | 4847.7215 | 3050855.25 |
| 2075-2089 | Cr-pCr-pAr-pGr-pAr-pCr-pAr-pCr-pAr-pGr-pAr-pCr-pAr-pAr-pAr | Cyclic phosphorylation | 15 | 0.05 | 98.3 | 1.1 | 22.31 | 691.811 | -7 | 4847.7217 | 4849.82 | 4847.7215 | 2208024.25 |
| 2928-2944 | Cr-pUr-pGr-pAr-pAr-pCr-pAr-pCr-pCr-pCr-pUr-pGr-pGr-pUr-pCr | Cyclic phosphorylation | 17 | 0.09 | 99.9 | 1 | 22.48 | 677.834 | -8 | 5428.729 | 5431.01 | 5428.7285 | 4381246.5 |
| 2928-2944 | Cr-pUr-pGr-pAr-pAr-pCr-pAr-pCr-pCr-pCr-pUr-pGr-pGr-pUr-pCr | Cyclic phosphorylation | 17 | 1.08 | 99.9 | 1 | 22.48 | 602.408 | -9 | 5428.7344 | 5430.97 | 5428.7285 | 3705927.5 |
| 825-843 | Ur-pGr-pGr-pAr-pCr-pAr-pGr-pCr-pUr-pGr-pGr-pUr-pGr-pCr-pC | Cyclic phosphorylation | 19 | 0.71 | 96.6 | 1.1 | 22.55 | 615.974 | -10 | 6166.8125 | 6169.51 | 6166.8081 | 673829.88 |
| 3467-3485 | Cr-pGr-pAr-pCr-pCr-pCr-pUr-pCr-pUr-pGr-pCr-pAr-pGr-pCr-pCr | Cyclic phosphorylation | 19 | -0.41 | 100 | 1 | 22.55 | 757.97 | -8 | 6069.8145 | 6072.61 | 6069.8169 | 5101206.5 |
| 825-843 | Ur-pGr-pGr-pAr-pCr-pAr-pGr-pCr-pUr-pGr-pGr-pUr-pGr-pCr-pC | Cyclic phosphorylation | 19 | 0.55 | 96.6 | 1 | 22.55 | 684.417 | -9 | 6166.8115 | 6169.41 | 6166.8081 | 3902184.75 |
| 2356-2372 | Cr-pAr-pGr-pGr-pGr-pAr-pUr-pCr-pGr-pCr-pCr-pGr-pUr-pGr-pG | Cyclic phosphorylation | 17 | 0.08 | 100 | 1 | 22.72 | 1390.433 | -4 | 5563.7583 | 5566.26 | 5563.7579 | 2925443 |
| 2356-2372 | Cr-pAr-pGr-pGr-pGr-pAr-pUr-pCr-pGr-pCr-pCr-pGr-pUr-pGr-pG | Cyclic phosphorylation | 17 | 0.08 | 100 | 1 | 22.72 | 694.713 | -8 | 5563.7583 | 5566.05 | 5563.7579 | 17713764 |
| 2356-2372 | Cr-pAr-pGr-pGr-pGr-pAr-pUr-pCr-pGr-pCr-pCr-pGr-pUr-pGr-pG | Cyclic phosphorylation | 17 | 0.6 | 100 | 1 | 22.72 | 1854.248 | -3 | 5563.7612 | 5566.23 | 5563.7579 | 4585697 |
| 2356-2372 | Cr-pAr-pGr-pGr-pGr-pAr-pUr-pCr-pGr-pCr-pCr-pGr-pUr-pGr-pG | Cyclic phosphorylation | 17 | -0.01 | 100 | 1 | 22.72 | 794.102 | -7 | 5563.7578 | 5566.04 | 5563.7579 | 4895491 |
| 2356-2372 | Cr-pAr-pGr-pGr-pGr-pAr-pUr-pCr-pGr-pCr-pCr-pGr-pUr-pGr-pG | Cyclic phosphorylation | 17 | 0.52 | 100 | 1 | 22.74 | 617.411 | -9 | 5563.7607 | 5566.09 | 5563.7579 | 10179262 |
| 1533-1551 | Ur-pCr-pCr-pUr-pAr-pCr-pGr-pGr-pCr-pUr-pUr-pUr-pCr-pAr-pG | Cyclic phosphorylation | 19 | 0.03 | 100 | 1 | 23.01 | 748.338 | -8 | 5992.7568 | 5995.34 | 5992.7567 | 8543525 |
| 1533-1551 | Ur-pCr-pCr-pUr-pAr-pCr-pGr-pGr-pCr-pUr-pUr-pUr-pCr-pAr-pG | Cyclic phosphorylation | 19 | 0.92 | 100 | 1 | 23.02 | 598.469 | -10 | 5992.7622 | 5995.34 | 5992.7567 | 15268191 |
| 1533-1551 | Ur-pCr-pCr-pUr-pAr-pCr-pGr-pGr-pCr-pUr-pUr-pUr-pCr-pAr-pG | Cyclic phosphorylation | 19 | 0.44 | 100 | 1 | 23.03 | 1497.684 | -4 | 5992.7593 | 5995.34 | 5992.7567 | 4058173.75 |
| 1533-1551 | Ur-pCr-pCr-pUr-pAr-pCr-pGr-pGr-pCr-pUr-pUr-pUr-pCr-pAr-pG | Cyclic phosphorylation | 19 | 0.92 | 100 | 1.1 | 23.03 | 1997.248 | -3 | 5992.7622 | 5995.37 | 5992.7567 | 4329710.5 |
| 1533-1551 | Ur-pCr-pCr-pUr-pAr-pCr-pGr-pGr-pCr-pUr-pUr-pUr-pCr-pAr-pG | Cyclic phosphorylation | 19 | 0.68 | 100 | 1 | 23.03 | 665.077 | -9 | 5992.7607 | 5995.31 | 5992.7567 | 20873154 |
| 2634-2653 | Cr-pUr-pGr-pCr-pCr-pUr-pCr-pCr-pUr-pCr-pUr-pGr-pCr-pUr-pG | Cyclic phosphorylation | 20 | -0.14 | 97.4 | 1 | 23.23 | 700.859 | -9 | 6313.792 | 6316.52 | 6313.7929 | 3701507.75 |
| 1849-1866 | Cr-pCr-pCr-pCr-pUr-pGr-pGr-pCr-pAr-pCr-pCr-pAr-pAr-pCr-pAr- | Cyclic phosphorylation | 18 | 0.65 | 98.5 | 1.1 | 23.26 | 631.637 | -9 | 5691.7993 | 5694.44 | 5691.7956 | 3411882 |
| 2634-2653 | Cr-pUr-pGr-pCr-pCr-pUr-pCr-pCr-pUr-pCr-pUr-pGr-pCr-pUr-pG | Cyclic phosphorylation | 20 | 0.33 | 97.4 | 1.1 | 23.27 | 630.572 | -10 | 6313.7949 | 6316.67 | 6313.7929 | 2916898.5 |
| 1849-1866 | Cr-pCr-pCr-pCr-pUr-pGr-pGr-pCr-pAr-pCr-pCr-pAr-pAr-pCr-pAr- | Cyclic phosphorylation | 18 | -0.38 | 98.5 | 1 | 23.27 | 710.843 | -8 | 5691.7935 | 5694.4 | 5691.7956 | 3798179.5 |
| 118-136 | Cr-pAr-pCr-pAr-pGr-pCr-pUr-pGr-pCr-pCr-pUr-pCr-pCr-pAr-pGr | Cyclic phosphorylation | 19 | 0.4 | 100 | 1.1 | 23.67 | 2004.595 | -3 | 6014.8022 | 6017.47 | 6014.7999 | 2499723.5 |
| 118-136 | Cr-pAr-pCr-pAr-pGr-pCr-pUr-pGr-pCr-pCr-pUr-pCr-pCr-pAr-pGr | Cyclic phosphorylation | 19 | 0.8 | 100 | 1.1 | 23.67 | 600.673 | -10 | 6014.8047 | 6017.39 | 6014.7999 | 3356507.75 |
| 118-136 | Cr-pAr-pCr-pAr-pGr-pCr-pUr-pGr-pCr-pCr-pUr-pCr-pCr-pAr-pGr | Cyclic phosphorylation | 19 | 0.4 | 100 | 1 | 23.67 | 667.527 | -9 | 6014.8022 | 6017.41 | 6014.7999 | 8679748 |
| 118-136 | Cr-pAr-pCr-pAr-pGr-pCr-pUr-pGr-pCr-pCr-pUr-pCr-pCr-pAr-pGr | Cyclic phosphorylation | 19 | -0.09 | 100 | 1 | 23.67 | 751.093 | -8 | 6014.7993 | 6017.4 | 6014.7999 | 6232109 |
| 1433-1451 | Ur-pCr-pUr-pGr-pAr-pAr-pGr-pCr-pCr-pCr-pUr-pUr-pCr-pGr-pA | Cyclic phosphorylation | 19 | 0.39 | 99.9 | 1 | 23.86 | 678.304 | -9 | 6111.7935 | 6114.36 | 6111.7911 | 2589085.25 |
| 1433-1451 | Ur-pCr-pUr-pGr-pAr-pAr-pGr-pCr-pCr-pCr-pUr-pUr-pCr-pGr-pA | Cyclic phosphorylation | 19 | 0.95 | 99.9 | 1.1 | 23.86 | 610.373 | -10 | 6111.7969 | 6114.22 | 6111.7911 | 726499 |
| 2761-2778 | Cr-pCr-pUr-pAr-pCr-pCr-pGr-pGr-pUr-pUr-pCr-pAr-pAr-pCr-pGr | Cyclic phosphorylation | 18 | 0.33 | 100 | 1 | 23.86 | 717.964 | -8 | 5749.7666 | 5752.38 | 5749.7647 | 12122471 |
| 2761-2778 | Cr-pCr-pUr-pAr-pCr-pCr-pGr-pGr-pUr-pUr-pCr-pAr-pAr-pCr-pGr | Cyclic phosphorylation | 18 | 0.5 | 100 | 1 | 23.88 | 820.674 | -7 | 5749.7676 | 5752.39 | 5749.7647 | 3867015 |
| 2761-2778 | Cr-pCr-pUr-pAr-pCr-pCr-pGr-pGr-pUr-pUr-pCr-pAr-pAr-pCr-pGr | Cyclic phosphorylation | 18 | 0.5 | 100 | 1 | 23.88 | 638.078 | -9 | 5749.7676 | 5752.37 | 5749.7647 | 17464200 |
| 2761-2778 | Cr-pCr-pUr-pAr-pCr-pCr-pGr-pGr-pUr-pUr-pCr-pAr-pAr-pCr-pGr | Cyclic phosphorylation | 18 | 0.84 | 100 | 1.1 | 23.89 | 1916.25 | -3 | 5749.7695 | 5752.43 | 5749.7647 | 4712860.5 |
| 2761-2778 | Cr-pCr-pUr-pAr-pCr-pCr-pGr-pGr-pUr-pUr-pCr-pAr-pAr-pCr-pGr | Cyclic phosphorylation | 18 | 0.33 | 100 | 1 | 23.89 | 1436.935 | -4 | 5749.7666 | 5752.43 | 5749.7647 | 3637155.75 |
| 2761-2778 | Cr-pCr-pUr-pAr-pCr-pCr-pGr-pGr-pUr-pUr-pCr-pAr-pAr-pCr-pGr | Cyclic phosphorylation | 18 | 0.58 | 100 | 1 | 23.89 | 574.17 | -10 | 5749.7681 | 5752.32 | 5749.7647 | 6008385.5 |
| 63-83 | Ur-pUr-pCr-pCr-pUr-pGr-pGr-pUr-pGr-pCr-pUr-pGr-pCr-pUr-pG | Cyclic phosphorylation | 21 | 0.71 | 100 | 1 | 24.1 | 738.636 | -9 | 6653.7808 | 6656.73 | 6653.776 | 2255774.75 |
| 563-582 | Cr-pGr-pUr-pGr-pUr-pCr-pCr-pCr-pAr-pGr-pCr-pCr-pUr-pUr-pU | Cyclic phosphorylation | 20 | 1.31 | 99.9 | 1 | 24.1 | 630.671 | -10 | 6314.7852 | 6317.44 | 6314.7769 | 1625839.12 |
| 63-83 | Ur-pUr-pCr-pCr-pUr-pGr-pGr-pUr-pGr-pCr-pUr-pGr-pCr-pUr-pG | Cyclic phosphorylation | 21 | 0.49 | 100 | 1 | 24.12 | 664.571 | -10 | 6653.7793 | 6656.56 | 6653.776 | 4493065.5 |
| 563-582 | Cr-pGr-pUr-pGr-pUr-pCr-pCr-pCr-pAr-pGr-pCr-pCr-pUr-pUr-pU | Cyclic phosphorylation | 20 | 0.38 | 99.9 | 1 | 24.14 | 700.858 | -9 | 6314.7793 | 6317.5 | 6314.7769 | 3109526.75 |
| 4123-4140 | Ur-pAr-pCr-pUr-pAr-pAr-pCr-pCr-pCr-pCr-pAr-pGr-pGr-pGr-pUr | Cyclic phosphorylation | 18 | 1.35 | 100 | 1 | 24.24 | 642.633 | -9 | 5790.7627 | 5793.2 | 5790.7549 | 5261409 |
| 4123-4140 | Ur-pAr-pCr-pUr-pAr-pAr-pCr-pCr-pCr-pCr-pAr-pGr-pGr-pGr-pUr | Cyclic phosphorylation | 18 | 0.76 | 100 | 1 | 24.24 | 723.088 | -8 | 5790.7593 | 5793.24 | 5790.7549 | 7665209 |
| 4123-4140 | Ur-pAr-pCr-pUr-pAr-pAr-pCr-pCr-pCr-pCr-pAr-pGr-pGr-pGr-pUr | Cyclic phosphorylation | 18 | 1.68 | 100 | 1 | 24.26 | 826.53 | -7 | 5790.7646 | 5793.29 | 5790.7549 | 2575841.75 |
| 1454-1471 | Cr-pAr-pUr-pCr-pUr-pCr-pCr-pAr-pCr-pCr-pGr-pAr-pGr-pAr-pUr | Cyclic phosphorylation | 18 | 0.32 | 97.6 | 1 | 24.57 | 631.966 | -9 | 5694.7495 | 5697.16 | 5694.7477 | 4946352.5 |
| 1454-1471 | Cr-pAr-pUr-pCr-pUr-pCr-pCr-pAr-pCr-pCr-pGr-pAr-pGr-pAr-pUr | Cyclic phosphorylation | 18 | 0.58 | 97.6 | 1 | 24.58 | 711.087 | -8 | 5694.751 | 5697.15 | 5694.7477 | 2957739.75 |
| 3206-3226 | Cr-pUr-pUr-pCr-pCr-pCr-pUr-pCr-pAr-pGr-pUr-pCr-pUr-pGr-pCr | Cyclic phosphorylation | 21 | 0.57 | 98.1 | 1 | 24.62 | 653.174 | -10 | 6539.8096 | 6542.58 | 6539.8059 | 8892935 |
| 3206-3226 | Cr-pUr-pUr-pCr-pCr-pCr-pUr-pCr-pAr-pGr-pUr-pCr-pUr-pGr-pCr | Cyclic phosphorylation | 21 | 0.34 | 98.1 | 1 | 24.62 | 593.703 | -11 | 6539.8081 | 6542.55 | 6539.8059 | 7555090 |
| 461-480 | Cr-pAr-pAr-pCr-pGr-pAr-pCr-pCr-pCr-pCr-pUr-pUr-pCr-pCr-pUr | Cyclic phosphorylation | 20 | 0.39 | 100 | 1 | 24.81 | 636.778 | -10 | 6375.8447 | 6378.58 | 6375.8422 | 3123182.5 |
| 461-480 | Cr-pAr-pAr-pCr-pGr-pAr-pCr-pCr-pCr-pCr-pUr-pUr-pCr-pCr-pUr | Cyclic phosphorylation | 20 | -0.14 | 100 | 1 | 24.81 | 707.642 | -9 | 6375.8413 | 6378.61 | 6375.8422 | 4850416.5 |
| 2926-2943 | Cr-pAr-pCr-pUr-pGr-pAr-pAr-pCr-pAr-pCr-pCr-pCr-pUr-pGr-pGr | Cyclic phosphorylation | 18 | -0.13 | 92 | 1 | 24.86 | 715.964 | -8 | 5733.769 | 5736.21 | 5733.7698 | 2704752.75 |
| 2926-2943 | Cr-pAr-pCr-pUr-pGr-pAr-pAr-pCr-pAr-pCr-pCr-pCr-pUr-pGr-pGr | Cyclic phosphorylation | 18 | -0.82 | 92 | 1.1 | 24.91 | 636.3 | -9 | 5733.7651 | 5736.62 | 5733.7698 | 964055.75 |
| 3003-3022 | Cr-pUr-pGr-pGr-pAr-pCr-pCr-pCr-pUr-pCr-pCr-pUr-pGr-pAr-pGr | Cyclic phosphorylation | 20 | 0.5 | 100 | 1.1 | 25.1 | 2138.278 | -3 | 6415.8516 | 6418.67 | 6415.8484 | 4089333.75 |
| 3003-3022 | Cr-pUr-pGr-pGr-pAr-pCr-pCr-pCr-pUr-pCr-pCr-pUr-pGr-pAr-pGr | Cyclic phosphorylation | 20 | -0.19 | 100 | 1 | 25.1 | 1282.563 | -5 | 6415.8472 | 6418.59 | 6415.8484 | 2747115.5 |
| 3003-3022 | Cr-pUr-pGr-pGr-pAr-pCr-pCr-pCr-pUr-pCr-pCr-pUr-pGr-pAr-pGr | Cyclic phosphorylation | 20 | 0.5 | 100 | 1.2 | 25.1 | 582.434 | -11 | 6415.8516 | 6418.55 | 6415.8484 | 2355874 |
| 3003-3022 | Cr-pUr-pGr-pGr-pAr-pCr-pCr-pCr-pUr-pCr-pCr-pUr-pGr-pAr-pGr | Cyclic phosphorylation | 20 | 0.5 | 100 | 1 | 25.1 | 640.778 | -10 | 6415.8516 | 6418.6 | 6415.8484 | 24543404 |
| 3003-3022 | Cr-pUr-pGr-pGr-pAr-pCr-pCr-pCr-pUr-pCr-pCr-pUr-pGr-pAr-pGr | Cyclic phosphorylation | 20 | -0.19 | 100 | 1 | 25.1 | 801.349 | -8 | 6415.8472 | 6418.69 | 6415.8484 | 7512612.5 |
| 3003-3022 | Cr-pUr-pGr-pGr-pAr-pCr-pCr-pCr-pUr-pCr-pCr-pUr-pGr-pAr-pGr | Cyclic phosphorylation | 20 | 0.19 | 100 | 1 | 25.12 | 1603.456 | -4 | 6415.8496 | 6418.65 | 6415.8484 | 5761341 |
| 3003-3022 | Cr-pUr-pGr-pGr-pAr-pCr-pCr-pCr-pUr-pCr-pCr-pUr-pGr-pAr-pGr | Cyclic phosphorylation | 20 | -0.03 | 100 | 1 | 25.12 | 712.087 | -9 | 6415.8481 | 6418.65 | 6415.8484 | 21424690 |
| 3003-3022 | Cr-pUr-pGr-pGr-pAr-pCr-pCr-pCr-pUr-pCr-pCr-pUr-pGr-pAr-pGr | Cyclic phosphorylation | 20 | -0.11 | 100 | 1 | 25.12 | 915.829 | -7 | 6415.8477 | 6418.67 | 6415.8484 | 3171513.5 |
| 464-484 | Cr-pGr-pAr-pCr-pCr-pCr-pCr-pUr-pUr-pCr-pCr-pUr-pGr-pGr-pGr | Cyclic phosphorylation | 21 | 0.08 | 99.8 | 1 | 25.54 | 739.198 | -9 | 6658.8408 | 6661.92 | 6658.8403 | 18344054 |
| 851-870 | Ur-pGr-pUr-pGr-pGr-pGr-pCr-pUr-pAr-pCr-pCr-pUr-pGr-pCr-pA | Cyclic phosphorylation | 20 | 2.44 | 99.9 | 1 | 25.58 | 801.596 | -8 | 6417.832 | 6420.48 | 6417.8164 | 3334180.25 |
| 464-484 | Cr-pGr-pAr-pCr-pCr-pCr-pCr-pUr-pUr-pCr-pCr-pUr-pGr-pGr-pGr | Cyclic phosphorylation | 21 | 0.37 | 99.8 | 1 | 25.63 | 831.598 | -8 | 6658.8428 | 6661.72 | 6658.8403 | 8445437 |
| 3275-3294 | Ur-pUr-pUr-pCr-pAr-pCr-pCr-pAr-pCr-pCr-pGr-pCr-pUr-pCr-pCr | Cyclic phosphorylation | 20 | 0.21 | 92.6 | 1 | 25.63 | 627.275 | -10 | 6280.8203 | 6283.48 | 6280.819 | 9416239 |
| 3275-3294 | Ur-pUr-pUr-pCr-pAr-pCr-pCr-pAr-pCr-pCr-pGr-pCr-pUr-pCr-pCr | Cyclic phosphorylation | 20 | 0.44 | 92.6 | 1 | 25.65 | 697.084 | -9 | 6280.8218 | 6283.56 | 6280.819 | 4379897 |
| 1532-1551 | Gr-pUr-pCr-pCr-pUr-pAr-pCr-pGr-pGr-pCr-pUr-pUr-pUr-pCr-pA | Cyclic phosphorylation | 20 | -0.06 | 95.8 | 1 | 25.74 | 703.415 | -9 | 6337.8037 | 6340.61 | 6337.8041 | 12604569 |
| 1532-1551 | Gr-pUr-pCr-pCr-pUr-pAr-pCr-pGr-pGr-pCr-pUr-pUr-pUr-pCr-pA | Cyclic phosphorylation | 20 | 0.56 | 95.8 | 1 | 25.75 | 632.974 | -10 | 6337.8076 | 6340.57 | 6337.8041 | 12259045 |
| 236-255 | Cr-pGr-pUr-pGr-pAr-pCr-pCr-pUr-pGr-pGr-pUr-pUr-pCr-pCr-pA | Cyclic phosphorylation | 20 | 1.6 | 99.1 | 1 | 25.86 | 707.752 | -9 | 6376.8364 | 6379.35 | 6376.8262 | 8114586.5 |
| 236-255 | Cr-pGr-pUr-pGr-pAr-pCr-pCr-pUr-pGr-pGr-pUr-pUr-pCr-pCr-pA | Cyclic phosphorylation | 20 | 1.52 | 99.1 | 1 | 25.87 | 796.348 | -8 | 6376.8359 | 6379.3 | 6376.8262 | 3577073.25 |
| 63-84 | Ur-pUr-pCr-pCr-pUr-pGr-pGr-pUr-pGr-pCr-pUr-pGr-pCr-pUr-pG | Cyclic phosphorylation | 22 | 0.46 | 100 | 1 | 26.03 | 699.176 | -10 | 6998.8267 | 7001.92 | 6998.8235 | 4469357 |
| 63-84 | Ur-pUr-pCr-pCr-pUr-pGr-pGr-pUr-pGr-pCr-pUr-pGr-pCr-pUr-pG | Cyclic phosphorylation | 22 | 1.71 | 100 | 1 | 26.03 | 635.432 | -11 | 6998.8354 | 7001.89 | 6998.8235 | 3701701 |
| 2715-2735 | Ur-pUr-pUr-pGr-pGr-pAr-pGr-pCr-pAr-pGr-pGr-pCr-pGr-pCr-pC | Cyclic phosphorylation | 21 | 0.17 | 100 | 1 | 26.46 | 752.533 | -9 | 6778.8599 | 6781.9 | 6778.8587 | 5413221.5 |
| 2715-2735 | Ur-pUr-pUr-pGr-pGr-pAr-pGr-pCr-pAr-pGr-pGr-pCr-pGr-pCr-pC | Cyclic phosphorylation | 21 | 0.09 | 100 | 1 | 26.46 | 615.435 | -11 | 6778.8594 | 6781.91 | 6778.8587 | 4208890 |
| 2715-2735 | Ur-pUr-pUr-pGr-pGr-pAr-pGr-pCr-pAr-pGr-pGr-pCr-pGr-pCr-pC | Cyclic phosphorylation | 21 | -0.05 | 100 | 1 | 26.48 | 677.079 | -10 | 6778.8584 | 6781.73 | 6778.8587 | 11278642 |
| 1412-1431 | Cr-pCr-pGr-pGr-pCr-pUr-pGr-pUr-pUr-pCr-pCr-pGr-pGr-pAr-pA | Cyclic phosphorylation | 20 | 0.1 | 94.3 | 1 | 26.51 | 712.308 | -9 | 6416.833 | 6419.86 | 6416.8324 | 5299069 |
| 1412-1431 | Cr-pCr-pGr-pGr-pCr-pUr-pGr-pUr-pUr-pCr-pCr-pGr-pGr-pAr-pA | Cyclic phosphorylation | 20 | 0.33 | 94.3 | 1 | 26.53 | 640.977 | -10 | 6416.8345 | 6419.86 | 6416.8324 | 6767583 |
| 1472-1492 | Ur-pCr-pAr-pGr-pGr-pCr-pCr-pGr-pGr-pCr-pAr-pGr-pCr-pAr-pCr | Cyclic phosphorylation | 21 | 0.44 | 100 | 1 | 26.63 | 671.282 | -10 | 6720.8926 | 6723.75 | 6720.8897 | 23345556 |
| 1472-1492 | Ur-pCr-pAr-pGr-pGr-pCr-pCr-pGr-pGr-pCr-pAr-pGr-pCr-pAr-pCr | Cyclic phosphorylation | 21 | 0.15 | 100 | 1 | 26.63 | 1679.717 | -4 | 6720.8906 | 6723.91 | 6720.8897 | 3988327 |
| 1472-1492 | Ur-pCr-pAr-pGr-pGr-pCr-pCr-pGr-pGr-pCr-pAr-pGr-pCr-pAr-pCr | Cyclic phosphorylation | 21 | 0.36 | 100 | 1.2 | 26.63 | 610.165 | -11 | 6720.8921 | 6723.78 | 6720.8897 | 2441929.75 |
| 3994-4013 | Cr-pCr-pAr-pCr-pCr-pUr-pCr-pUr-pGr-pCr-pUr-pAr-pGr-pUr-pU | Cyclic phosphorylation | 20 | -0.48 | 99.5 | 1 | 26.69 | 631.374 | -10 | 6321.8062 | 6324.56 | 6321.8092 | 3219180.25 |
| 3994-4013 | Cr-pCr-pAr-pCr-pCr-pUr-pCr-pUr-pGr-pCr-pUr-pAr-pGr-pUr-pU | Cyclic phosphorylation | 20 | -0.09 | 99.5 | 1 | 26.7 | 701.639 | -9 | 6321.8086 | 6324.48 | 6321.8092 | 3973042.25 |
| 953-972 | Gr-pUr-pGr-pCr-pAr-pCr-pCr-pCr-pUr-pGr-pAr-pAr-pGr-pUr-pC | Cyclic phosphorylation | 20 | 2.39 | 100 | 1.1 | 26.82 | 577.523 | -11 | 6361.8306 | 6364.39 | 6361.8153 | 10786599 |
| 953-972 | Gr-pUr-pGr-pCr-pAr-pCr-pCr-pCr-pUr-pGr-pAr-pAr-pGr-pUr-pC | Cyclic phosphorylation | 20 | 2.09 | 100 | 1.1 | 26.84 | 2120.269 | -3 | 6361.8286 | 6364.5 | 6361.8153 | 6772766.5 |
| 953-972 | Gr-pUr-pGr-pCr-pAr-pCr-pCr-pCr-pUr-pGr-pAr-pAr-pGr-pUr-pC | Cyclic phosphorylation | 20 | 1.4 | 100 | 1 | 26.84 | 706.084 | -9 | 6361.8242 | 6364.44 | 6361.8153 | 23574872 |
| 953-972 | Gr-pUr-pGr-pCr-pAr-pCr-pCr-pCr-pUr-pGr-pAr-pAr-pGr-pUr-pC | Cyclic phosphorylation | 20 | 0.71 | 100 | 1 | 26.84 | 908.11 | -7 | 6361.8198 | 6364.44 | 6361.8153 | 4176138.75 |
| 953-972 | Gr-pUr-pGr-pCr-pAr-pCr-pCr-pCr-pUr-pGr-pAr-pAr-pGr-pUr-pC | Cyclic phosphorylation | 20 | 0.78 | 100 | 1 | 26.84 | 794.47 | -8 | 6361.8203 | 6364.52 | 6361.8153 | 11530397 |
| 953-972 | Gr-pUr-pGr-pCr-pAr-pCr-pCr-pCr-pUr-pGr-pAr-pAr-pGr-pUr-pC | Cyclic phosphorylation | 20 | 1.63 | 100 | 1 | 26.84 | 1589.949 | -4 | 6361.8257 | 6364.42 | 6361.8153 | 8090510 |
| 2533-2553 | Cr-pAr-pCr-pUr-pGr-pGr-pCr-pCr-pGr-pAr-pCr-pGr-pCr-pCr-pGr | Cyclic phosphorylation | 21 | 0.22 | 100 | 1 | 27.22 | 671.481 | -10 | 6720.8911 | 6724.4 | 6720.8897 | 8797542 |
| 2533-2553 | Cr-pAr-pCr-pUr-pGr-pGr-pCr-pCr-pGr-pAr-pCr-pGr-pCr-pCr-pGr | Cyclic phosphorylation | 21 | 0.87 | 100 | 1 | 27.25 | 1679.966 | -4 | 6720.8955 | 6724.15 | 6720.8897 | 3747442.25 |
| 2533-2553 | Cr-pAr-pCr-pUr-pGr-pGr-pCr-pCr-pGr-pAr-pCr-pGr-pCr-pCr-pGr | Cyclic phosphorylation | 21 | -0.22 | 100 | 1 | 27.3 | 839.354 | -8 | 6720.8882 | 6724.01 | 6720.8897 | 3566141.5 |
| 1322-1341 | Cr-pAr-pAr-pGr-pCr-pUr-pGr-pCr-pCr-pCr-pGr-pAr-pCr-pGr-pAr | Cyclic phosphorylation | 20 | 0.28 | 96.1 | 1 | 27.41 | 708.533 | -9 | 6383.8604 | 6386.59 | 6383.8585 | 8023071.5 |
| 1322-1341 | Cr-pAr-pAr-pGr-pCr-pUr-pGr-pCr-pCr-pCr-pGr-pAr-pCr-pGr-pAr | Cyclic phosphorylation | 20 | 0.21 | 96.1 | 1 | 27.42 | 637.579 | -10 | 6383.8599 | 6386.57 | 6383.8585 | 8634848 |
| 1322-1341 | Cr-pAr-pAr-pGr-pCr-pUr-pGr-pCr-pCr-pCr-pGr-pAr-pCr-pGr-pAr | Cyclic phosphorylation | 20 | 0.21 | 96.1 | 1.1 | 27.42 | 797.351 | -8 | 6383.8599 | 6386.57 | 6383.8585 | 3497721.75 |
| 3911-3933 | Cr-pUr-pGr-pCr-pCr-pCr-pCr-pUr-pUr-pUr-pCr-pCr-pCr-pGr-pUr | Cyclic phosphorylation | 23 | 0.77 | 100 | 1 | 27.63 | 658.073 | -11 | 7246.8853 | 7250.03 | 7246.8797 | 8623961 |
| 3911-3933 | Cr-pUr-pGr-pCr-pCr-pCr-pCr-pUr-pUr-pUr-pCr-pCr-pCr-pGr-pUr | Cyclic phosphorylation | 23 | 0.57 | 100 | 1.4 | 27.63 | 603.15 | -12 | 7246.8838 | 7250.05 | 7246.8797 | 3108525 |

| | | | | | | | | | | | | |
|---|---|---|---|---|---|---|---|---|---|---|---|---|
| 3911-3933 | Cr-pUr-pGr-pCr-pCr-pCr-pCr-pUr-pUr-pUr-pCr-pCr-pCr-pGr-pU | Cyclic phosphorylation | 23 | -0.11 | 100 | 1 | 27.64 | 723.982 | -10 | 7246.8789 | 7250.07 | 7246.8797 | 3926816.5 |
| 4049-4069 | Cr-pUr-pUr-pAr-pGr-pCr-pCr-pUr-pAr-pGr-pCr-pCr-pAr-pCr-pA | Cyclic phosphorylation | 21 | -0.2 | 99.1 | 1 | 27.75 | 660.082 | -10 | 6608.8862 | 6613.1 | 6608.8875 | 3205277.75 |
| 4049-4069 | Cr-pUr-pUr-pAr-pGr-pCr-pCr-pUr-pAr-pGr-pCr-pCr-pAr-pCr-pA | Cyclic phosphorylation | 21 | 0.99 | 99.1 | 1 | 27.75 | 600.074 | -11 | 6608.894 | 6611.95 | 6608.8875 | 2878225.75 |
| 2926-2944 | Cr-pAr-pCr-pUr-pGr-pAr-pAr-pCr-pAr-pCr-pCr-pCr-pUr-pGr-pG | Cyclic phosphorylation | 19 | -0.25 | 100 | 1 | 27.8 | 865.396 | -7 | 6062.8208 | 6065.47 | 6062.8223 | 1109686 |
| 2926-2944 | Cr-pAr-pCr-pUr-pGr-pAr-pAr-pCr-pAr-pCr-pCr-pCr-pUr-pGr-pG | Cyclic phosphorylation | 19 | 0.79 | 100 | 1 | 27.82 | 672.862 | -9 | 6062.8271 | 6065.41 | 6062.8223 | 597314 |
| 2926-2944 | Cr-pAr-pCr-pUr-pGr-pAr-pAr-pCr-pAr-pCr-pCr-pCr-pUr-pGr-pG | Cyclic phosphorylation | 19 | -0.33 | 100 | 1 | 27.82 | 757.096 | -8 | 6062.8203 | 6065.48 | 6062.8223 | 2377975 |
| 3048-3067 | Gr-pGr-pCr-pAr-pGr-pAr-pCr-pUr-pGr-pCr-pAr-pGr-pAr-pGr-pC | Cyclic phosphorylation | 20 | -2.43 | 96 | 1.1 | 27.94 | 807.104 | -8 | 6462.8711 | 6465.67 | 6462.8868 | 1954810.75 |
| 3048-3067 | Gr-pGr-pCr-pAr-pGr-pAr-pCr-pUr-pGr-pCr-pAr-pGr-pAr-pGr-pC | Cyclic phosphorylation | 20 | 0.29 | 96 | 1 | 27.94 | 717.314 | -9 | 6462.8887 | 6465.61 | 6462.8868 | 2295143.25 |
| 2949-2970 | Cr-pUr-pGr-pUr-pCr-pCr-pUr-pCr-pCr-pAr-pAr-pCr-pUr-pUr-pC | Cyclic phosphorylation | 22 | 0.78 | 100 | 1 | 27.96 | 694.082 | -10 | 6947.8921 | 6951 | 6947.8867 | 3147150.75 |
| 4155-4174 | Gr-pCr-pCr-pAr-pCr-pAr-pCr-pCr-pCr-pUr-pGr-pGr-pAr-pGr-pC | Cyclic phosphorylation | 20 | 0.56 | 98.9 | 1 | 27.97 | 641.481 | -10 | 6422.8843 | 6425.86 | 6422.8807 | 1852452.12 |
| 2949-2970 | Cr-pUr-pGr-pUr-pCr-pCr-pUr-pCr-pCr-pAr-pAr-pCr-pUr-pUr-pC | Cyclic phosphorylation | 22 | 0.01 | 100 | 1 | 27.97 | 630.984 | -11 | 6947.8867 | 6950.93 | 6947.8867 | 1481576.75 |
| 2949-2970 | Cr-pUr-pGr-pUr-pCr-pCr-pUr-pCr-pCr-pAr-pAr-pCr-pUr-pUr-pC | Cyclic phosphorylation | 22 | 0.01 | 100 | 1 | 27.98 | 771.314 | -9 | 6947.8867 | 6950.96 | 6947.8867 | 1515988.38 |
| 4155-4174 | Gr-pCr-pCr-pAr-pCr-pAr-pCr-pCr-pCr-pUr-pGr-pGr-pAr-pGr-pC | Cyclic phosphorylation | 20 | 0.33 | 98.9 | 1 | 27.98 | 712.98 | -9 | 6422.8828 | 6425.87 | 6422.8807 | 2140379 |
| 2204-2223 | Cr-pUr-pUr-pCr-pAr-pCr-pCr-pAr-pUr-pCr-pAr-pGr-pCr-pGr-pU | Cyclic phosphorylation | 20 | 2.08 | 96.4 | 1 | 28.08 | 633.677 | -10 | 6344.8496 | 6347.52 | 6344.8364 | 3358602.25 |
| 2204-2223 | Cr-pUr-pUr-pCr-pAr-pCr-pCr-pAr-pUr-pCr-pAr-pGr-pCr-pGr-pU | Cyclic phosphorylation | 20 | 0.85 | 96.4 | 1 | 28.11 | 575.979 | -11 | 6344.8418 | 6347.52 | 6344.8364 | 1843202.88 |
| 2726-2748 | Cr-pGr-pCr-pCr-pGr-pCr-pUr-pCr-pUr-pGr-pCr-pAr-pGr-pAr-pU | Cyclic phosphorylation | 23 | 1.19 | 97.4 | 1 | 28.2 | 807.206 | -9 | 7269.9155 | 7272.77 | 7269.9069 | 728170.56 |
| 1406-1426 | Cr-pCr-pUr-pGr-pUr-pAr-pCr-pCr-pGr-pGr-pCr-pUr-pGr-pUr-pU | Cyclic phosphorylation | 21 | 0.62 | 99.9 | 1 | 28.34 | 610.344 | -11 | 6722.8618 | 6725.67 | 6722.8577 | 1841390.62 |
| 1406-1426 | Cr-pCr-pUr-pGr-pUr-pAr-pCr-pCr-pGr-pGr-pCr-pUr-pGr-pUr-pU | Cyclic phosphorylation | 21 | 0.91 | 99.9 | 1 | 28.34 | 671.479 | -10 | 6722.8638 | 6725.72 | 6722.8577 | 3964452.5 |
| 1406-1426 | Cr-pCr-pUr-pGr-pUr-pAr-pCr-pCr-pGr-pGr-pCr-pUr-pGr-pUr-pU | Cyclic phosphorylation | 21 | 0.62 | 99.9 | 1 | 28.35 | 746.311 | -9 | 6722.8618 | 6725.94 | 6722.8577 | 1435827.75 |
| 1000-1020 | Gr-pCr-pAr-pAr-pCr-pUr-pUr-pCr-pCr-pGr-pGr-pGr-pUr-pGr-pC | Cyclic phosphorylation | 21 | -0.22 | 100 | 1 | 28.36 | 842.356 | -8 | 6744.8994 | 6747.97 | 6744.9009 | 2580554.75 |
| 1000-1020 | Gr-pCr-pAr-pAr-pCr-pUr-pUr-pCr-pCr-pGr-pGr-pGr-pUr-pGr-pC | Cyclic phosphorylation | 21 | -0.07 | 100 | 1 | 28.39 | 748.648 | -9 | 6744.9004 | 6748.06 | 6744.9009 | 6140793.5 |
| 1000-1020 | Gr-pCr-pAr-pAr-pCr-pUr-pUr-pCr-pCr-pGr-pGr-pGr-pUr-pGr-pC | Cyclic phosphorylation | 21 | 0.58 | 100 | 1 | 28.41 | 1685.718 | -4 | 6744.9048 | 6748.1 | 6744.9009 | 2049362.5 |
| 1433-1453 | Ur-pCr-pUr-pGr-pAr-pAr-pGr-pCr-pCr-pCr-pUr-pUr-pCr-pGr-pA | Cyclic phosphorylation | 21 | -0.06 | 100 | 1 | 28.72 | 753.203 | -9 | 6785.8906 | 6788.85 | 6785.891 | 4681566.5 |
| 1433-1453 | Ur-pCr-pUr-pGr-pAr-pAr-pGr-pCr-pCr-pCr-pUr-pUr-pCr-pGr-pA | Cyclic phosphorylation | 21 | 0.3 | 100 | 1 | 28.73 | 677.782 | -10 | 6785.8931 | 6788.75 | 6785.891 | 8666970 |
| 2758-2778 | Ur-pGr-pGr-pCr-pCr-pUr-pAr-pCr-pCr-pGr-pGr-pUr-pUr-pCr-pA | Cyclic phosphorylation | 21 | 0.78 | 100 | 1 | 28.81 | 1686.216 | -4 | 6745.8901 | 6748.97 | 6745.8849 | 3863420.25 |
| 2758-2778 | Ur-pGr-pGr-pCr-pCr-pUr-pAr-pCr-pCr-pGr-pGr-pUr-pUr-pCr-pA | Cyclic phosphorylation | 21 | 0.05 | 100 | 1 | 28.83 | 612.528 | -11 | 6745.8853 | 6748.78 | 6745.8849 | 13293344 |
| 2758-2778 | Ur-pGr-pGr-pCr-pCr-pUr-pAr-pCr-pCr-pGr-pGr-pUr-pUr-pCr-pA | Cyclic phosphorylation | 21 | -0.09 | 100 | 1 | 28.83 | 748.758 | -9 | 6745.8843 | 6748.97 | 6745.8849 | 7707714 |
| 2758-2778 | Ur-pGr-pGr-pCr-pCr-pUr-pAr-pCr-pCr-pGr-pGr-pUr-pUr-pCr-pA | Cyclic phosphorylation | 21 | 0.12 | 100 | 1 | 28.83 | 673.781 | -10 | 6745.8857 | 6748.95 | 6745.8849 | 18022866 |
| 416-435 | Cr-pAr-pAr-pCr-pGr-pCr-pCr-pAr-pCr-pCr-pAr-pAr-pCr-pGr-pUr | Cyclic phosphorylation | 20 | -0.31 | 100 | 1 | 28.86 | 581.619 | -11 | 6406.8838 | 6409.69 | 6406.8858 | 3918224 |
| 416-435 | Cr-pAr-pAr-pCr-pGr-pCr-pCr-pAr-pCr-pCr-pAr-pAr-pCr-pGr-pUr | Cyclic phosphorylation | 20 | -0.23 | 100 | 1 | 28.86 | 711.091 | -9 | 6406.8843 | 6409.63 | 6406.8858 | 10435602 |
| 416-435 | Cr-pAr-pAr-pCr-pGr-pCr-pCr-pAr-pCr-pCr-pAr-pAr-pCr-pGr-pUr | Cyclic phosphorylation | 20 | 0.23 | 100 | 1 | 28.86 | 800.104 | -8 | 6406.8872 | 6409.69 | 6406.8858 | 3784301.25 |
| 416-435 | Cr-pAr-pAr-pCr-pGr-pCr-pCr-pAr-pCr-pCr-pAr-pAr-pCr-pGr-pUr | Cyclic phosphorylation | 20 | 0.38 | 100 | 1 | 28.86 | 639.882 | -10 | 6406.8882 | 6409.68 | 6406.8858 | 14758224 |
| 1846-1866 | Ur-pCr-pAr-pCr-pCr-pCr-pCr-pUr-pGr-pGr-pCr-pAr-pCr-pCr-pAr | Cyclic phosphorylation | 21 | 0.71 | 100 | 1 | 29.07 | 602.077 | -11 | 6631.9194 | 6634.72 | 6631.9147 | 8989608 |
| 1846-1866 | Ur-pCr-pAr-pCr-pCr-pCr-pCr-pUr-pGr-pGr-pCr-pAr-pCr-pCr-pAr | Cyclic phosphorylation | 21 | 0.27 | 100 | 1 | 29.07 | 662.385 | -10 | 6631.9165 | 6634.75 | 6631.9147 | 16811876 |
| 1846-1866 | Ur-pCr-pAr-pCr-pCr-pCr-pCr-pUr-pGr-pGr-pCr-pAr-pCr-pCr-pAr | Cyclic phosphorylation | 21 | 0.27 | 100 | 1 | 29.07 | 736.206 | -9 | 6631.9165 | 6634.74 | 6631.9147 | 12600067 |
| 1846-1866 | Ur-pCr-pAr-pCr-pCr-pCr-pCr-pUr-pGr-pGr-pCr-pAr-pCr-pCr-pAr | Cyclic phosphorylation | 21 | 0.04 | 100 | 1 | 29.07 | 1657.473 | -4 | 6631.915 | 6634.77 | 6631.9147 | 4561971 |
| 1846-1866 | Ur-pCr-pAr-pCr-pCr-pCr-pCr-pUr-pGr-pGr-pCr-pAr-pCr-pCr-pAr | Cyclic phosphorylation | 21 | -0.03 | 100 | 1 | 29.07 | 828.358 | -8 | 6631.9146 | 6634.84 | 6631.9147 | 4294249.5 |
| 1172-1192 | Cr-pUr-pUr-pCr-pAr-pGr-pCr-pAr-pCr-pCr-pUr-pUr-pCr-pAr-pA | Cyclic phosphorylation | 21 | 0.58 | 100 | 1.9 | 29.2 | 2217.279 | -3 | 6651.8496 | 6654.77 | 6651.8457 | 3136444.25 |
| 1172-1192 | Cr-pUr-pUr-pCr-pAr-pGr-pCr-pAr-pCr-pCr-pUr-pUr-pCr-pAr-pA | Cyclic phosphorylation | 21 | 0.51 | 100 | 1 | 29.22 | 738.421 | -9 | 6651.8491 | 6654.66 | 6651.8457 | 5642536.5 |
| 1172-1192 | Cr-pUr-pUr-pCr-pAr-pGr-pCr-pAr-pCr-pCr-pUr-pUr-pCr-pAr-pA | Cyclic phosphorylation | 21 | 0.29 | 100 | 1.2 | 29.22 | 553.48 | -12 | 6651.8477 | 6654.75 | 6651.8457 | 2356070.5 |
| 1172-1192 | Cr-pUr-pUr-pCr-pAr-pGr-pCr-pAr-pCr-pCr-pUr-pUr-pCr-pAr-pA | Cyclic phosphorylation | 21 | 0.66 | 100 | 1 | 29.22 | 664.478 | -10 | 6651.8501 | 6654.68 | 6651.8457 | 20880676 |
| 1172-1192 | Cr-pUr-pUr-pCr-pAr-pGr-pCr-pAr-pCr-pCr-pUr-pUr-pCr-pAr-pA | Cyclic phosphorylation | 21 | 0.66 | 100 | 1 | 29.22 | 1662.456 | -4 | 6651.8501 | 6654.85 | 6651.8457 | 4217742 |
| 533-552 | Cr-pAr-pGr-pCr-pAr-pGr-pCr-pGr-pCr-pCr-pAr-pAr-pCr-pAr-pAr | Cyclic phosphorylation | 20 | 0.78 | 100 | 1 | 29.22 | 642.185 | -10 | 6429.918 | 6432.67 | 6429.913 | 9435599 |
| 533-552 | Cr-pAr-pGr-pCr-pAr-pGr-pCr-pGr-pCr-pCr-pAr-pAr-pCr-pAr-pAr | Cyclic phosphorylation | 20 | -0.13 | 100 | 1 | 29.22 | 803.108 | -8 | 6429.9121 | 6432.65 | 6429.913 | 2288240 |
| 1172-1192 | Cr-pUr-pUr-pCr-pAr-pGr-pCr-pAr-pCr-pCr-pUr-pUr-pCr-pAr-pA | Cyclic phosphorylation | 21 | 0.51 | 100 | 1 | 29.24 | 603.979 | -11 | 6651.8491 | 6654.69 | 6651.8457 | 9025226 |
| 533-552 | Cr-pAr-pGr-pCr-pAr-pGr-pCr-pGr-pCr-pCr-pAr-pAr-pCr-pAr-pAr | Cyclic phosphorylation | 20 | 0.25 | 100 | 1.2 | 29.24 | 1607.223 | -4 | 6429.9146 | 6432.69 | 6429.913 | 2421416.25 |
| 533-552 | Cr-pAr-pGr-pCr-pAr-pGr-pCr-pGr-pCr-pCr-pAr-pAr-pCr-pAr-pAr | Cyclic phosphorylation | 20 | 0.17 | 100 | 1 | 29.24 | 713.762 | -9 | 6429.9141 | 6432.69 | 6429.913 | 5844679.5 |
| 1595-1617 | Cr-pUr-pUr-pCr-pGr-pAr-pAr-pCr-pUr-pGr-pCr-pUr-pGr-pCr-pA | Cyclic phosphorylation | 23 | 1.25 | 99.2 | 1 | 29.67 | 662.26 | -11 | 7293.9272 | 7297.04 | 7293.9181 | 1236582.88 |
| 1595-1617 | Cr-pUr-pUr-pCr-pGr-pAr-pAr-pCr-pUr-pGr-pCr-pUr-pGr-pCr-pA | Cyclic phosphorylation | 23 | 3.06 | 99.2 | 1 | 29.71 | 728.586 | -10 | 7293.9404 | 7296.91 | 7293.9181 | 1778941.88 |
| 3403-3424 | Cr-pCr-pGr-pAr-pCr-pAr-pAr-pCr-pAr-pCr-pCr-pUr-pUr-pCr-pGr | Cyclic phosphorylation | 22 | 2.47 | 99.8 | 1 | 29.72 | 778.428 | -9 | 7011.9214 | 7014.88 | 7011.9041 | 1792675.5 |
| 4120-4140 | Cr-pUr-pAr-pUr-pAr-pCr-pUr-pAr-pAr-pCr-pCr-pCr-pCr-pAr-pGr | Cyclic phosphorylation | 21 | 2.11 | 100 | 1 | 30.04 | 840.604 | -8 | 6730.8882 | 6733.6 | 6730.874 | 941363.88 |
| 4120-4140 | Cr-pUr-pAr-pUr-pAr-pCr-pUr-pAr-pAr-pCr-pCr-pCr-pCr-pAr-pGr | Cyclic phosphorylation | 21 | 2.03 | 100 | 1 | 30.05 | 747.091 | -9 | 6730.8877 | 6733.66 | 6730.874 | 3061744.5 |
| 4123-4143 | Ur-pAr-pCr-pUr-pAr-pAr-pCr-pCr-pCr-pCr-pAr-pGr-pGr-pGr-pU | Cyclic phosphorylation | 21 | 0.73 | 98.8 | 1 | 30.54 | 747.202 | -9 | 6730.8789 | 6734.09 | 6730.874 | 3150916.25 |
| 4123-4143 | Ur-pAr-pCr-pUr-pAr-pAr-pCr-pCr-pCr-pCr-pAr-pGr-pGr-pGr-pU | Cyclic phosphorylation | 21 | 1.6 | 98.8 | 1 | 30.54 | 611.073 | -11 | 6730.8848 | 6733.81 | 6730.874 | 4866705 |
| 4123-4143 | Ur-pAr-pCr-pUr-pAr-pAr-pCr-pCr-pCr-pCr-pAr-pGr-pGr-pGr-pU | Cyclic phosphorylation | 21 | 0.95 | 98.8 | 1 | 30.54 | 672.281 | -10 | 6730.8804 | 6733.82 | 6730.874 | 11501463 |
| 4123-4143 | Ur-pAr-pCr-pUr-pAr-pAr-pCr-pCr-pCr-pCr-pAr-pGr-pGr-pGr-pU | Cyclic phosphorylation | 21 | 1.31 | 98.8 | 1 | 30.54 | 1682.464 | -4 | 6730.8828 | 6733.99 | 6730.874 | 1858932.25 |
| 388-408 | Gr-pCr-pAr-pAr-pGr-pAr-pCr-pCr-pCr-pAr-pGr-pAr-pGr-pCr-pCr | Cyclic phosphorylation | 21 | 2.25 | 95 | 1 | 30.65 | 753.987 | -9 | 6791.9546 | 6795.26 | 6791.9393 | 3659053.5 |
| 15-37 | Ur-pUr-pCr-pUr-pUr-pCr-pUr-pGr-pGr-pUr-pCr-pCr-pCr-pCr-pA | Cyclic phosphorylation | 23 | 1.19 | 100 | 1 | 30.85 | 1809.221 | -4 | 7238.9097 | 7242.1 | 7238.9011 | 2520776 |
| 15-37 | Ur-pUr-pCr-pUr-pUr-pCr-pUr-pGr-pGr-pUr-pCr-pCr-pCr-pCr-pA | Cyclic phosphorylation | 23 | 1.32 | 100 | 1 | 30.86 | 657.348 | -11 | 7238.9106 | 7242.11 | 7238.9011 | 9672647 |
| 15-37 | Ur-pUr-pCr-pUr-pUr-pCr-pUr-pGr-pGr-pUr-pCr-pCr-pCr-pCr-pA | Cyclic phosphorylation | 23 | 1.86 | 100 | 1 | 30.86 | 602.486 | -12 | 7238.9146 | 7241.92 | 7238.9011 | 2835335.25 |
| 15-37 | Ur-pUr-pCr-pUr-pUr-pCr-pUr-pGr-pGr-pUr-pCr-pCr-pCr-pCr-pA | Cyclic phosphorylation | 23 | 1.05 | 100 | 1 | 30.86 | 723.184 | -10 | 7238.9087 | 7242.09 | 7238.9011 | 5469374 |
| 388-408 | Gr-pCr-pAr-pAr-pGr-pAr-pCr-pCr-pCr-pAr-pGr-pAr-pGr-pCr-pCr | Cyclic phosphorylation | 21 | 1.24 | 95 | 1.1 | 30.95 | 616.716 | -11 | 6791.9478 | 6794.89 | 6791.9393 | 947024 |
| 1879-1899 | Cr-pAr-pGr-pUr-pGr-pCr-pUr-pGr-pUr-pAr-pCr-pCr-pAr-pGr-pG | Cyclic phosphorylation | 21 | 1.3 | 100 | 1 | 31.04 | 755.872 | -9 | 6809.9111 | 6812.95 | 6809.9023 | 1719930.5 |
| 3202-3226 | Ur-pGr-pAr-pGr-pCr-pUr-pUr-pCr-pCr-pCr-pUr-pCr-pAr-pGr-pU | Cyclic phosphorylation | 25 | 0.68 | 96.9 | 1 | 31.05 | 785.792 | -10 | 7864.9839 | 7868.5 | 7864.9786 | 2207125.75 |
| 1879-1899 | Cr-pAr-pGr-pUr-pGr-pCr-pUr-pGr-pUr-pAr-pCr-pCr-pAr-pGr-pG | Cyclic phosphorylation | 21 | 0.73 | 100 | 1 | 31.06 | 680.284 | -10 | 6809.9072 | 6813 | 6809.9023 | 2651622.75 |
| 1879-1899 | Cr-pAr-pGr-pUr-pGr-pCr-pUr-pGr-pUr-pAr-pCr-pCr-pAr-pGr-pG | Cyclic phosphorylation | 21 | 1.59 | 100 | 1 | 31.07 | 618.258 | -11 | 6809.9131 | 6812.85 | 6809.9023 | 1560540.12 |
| 3202-3226 | Ur-pGr-pAr-pGr-pCr-pUr-pUr-pCr-pCr-pCr-pUr-pCr-pAr-pGr-pU | Cyclic phosphorylation | 25 | 4.03 | 96.9 | 1.2 | 31.07 | 714.356 | -11 | 7865.0103 | 7868.58 | 7864.9786 | 1593413.5 |
| 151-172 | Cr-pCr-pAr-pGr-pAr-pGr-pGr-pCr-pGr-pUr-pGr-pUr-pAr-pCr-pU | Cyclic phosphorylation | 22 | 1.27 | 97.5 | 1 | 31.3 | 785.322 | -9 | 7073.9624 | 7077.43 | 7073.9534 | 2041118.12 |
| 151-172 | Cr-pCr-pAr-pGr-pAr-pGr-pGr-pCr-pGr-pUr-pGr-pUr-pAr-pCr-pU | Cyclic phosphorylation | 22 | 1.27 | 97.5 | 1 | 31.31 | 706.689 | -10 | 7073.9624 | 7077.26 | 7073.9534 | 4048064.75 |
| 151-172 | Cr-pCr-pAr-pGr-pAr-pGr-pGr-pCr-pGr-pUr-pGr-pUr-pAr-pCr-pU | Cyclic phosphorylation | 22 | 1.27 | 97.5 | 1 | 31.33 | 642.353 | -11 | 7073.9624 | 7077.71 | 7073.9534 | 3266082.25 |
| 2724-2748 | Gr-pGr-pCr-pGr-pCr-pCr-pGr-pCr-pUr-pCr-pUr-pGr-pCr-pAr-pG | Cyclic phosphorylation | 25 | 2.17 | 99.3 | 1 | 31.43 | 795.394 | -10 | 7960.019 | 7963.91 | 7960.0018 | 1558027.38 |
| 461-484 | Cr-pAr-pAr-pCr-pGr-pAr-pCr-pCr-pCr-pCr-pUr-pUr-pCr-pCr-pUr | Cyclic phosphorylation | 24 | 1.05 | 100 | 1 | 31.73 | 1088.135 | -7 | 7621.9946 | 7625.33 | 7621.9866 | 1784156.25 |
| 461-484 | Cr-pAr-pAr-pCr-pGr-pAr-pCr-pCr-pCr-pCr-pUr-pUr-pCr-pCr-pUr | Cyclic phosphorylation | 24 | 0.92 | 100 | 1.4 | 31.75 | 585.532 | -13 | 7621.9937 | 7625.3 | 7621.9866 | 1840586.5 |
| 461-484 | Cr-pAr-pAr-pCr-pGr-pAr-pCr-pCr-pCr-pCr-pUr-pUr-pCr-pCr-pUr | Cyclic phosphorylation | 24 | 0.41 | 100 | 1 | 31.75 | 846.215 | -9 | 7621.9897 | 7625.31 | 7621.9866 | 4013733.75 |
| 461-484 | Cr-pAr-pAr-pCr-pGr-pAr-pCr-pCr-pCr-pCr-pUr-pUr-pCr-pCr-pUr | Cyclic phosphorylation | 24 | 1.63 | 100 | 1 | 31.75 | 634.409 | -12 | 7621.999 | 7625.3 | 7621.9866 | 20901388 |
| 461-484 | Cr-pAr-pAr-pCr-pGr-pAr-pCr-pCr-pCr-pCr-pUr-pUr-pCr-pCr-pUr | Cyclic phosphorylation | 24 | 1.11 | 100 | 1 | 31.75 | 951.992 | -8 | 7621.9951 | 7625.31 | 7621.9866 | 2348245.5 |
| 461-484 | Cr-pAr-pAr-pCr-pGr-pAr-pCr-pCr-pCr-pCr-pUr-pUr-pCr-pCr-pUr | Cyclic phosphorylation | 24 | 0.79 | 100 | 1 | 31.75 | 1523.792 | -5 | 7621.9927 | 7625.32 | 7621.9866 | 2829993.25 |
| 461-484 | Cr-pAr-pAr-pCr-pGr-pAr-pCr-pCr-pCr-pCr-pUr-pUr-pCr-pCr-pUr | Cyclic phosphorylation | 24 | 1.5 | 100 | 1.1 | 31.75 | 1905.244 | -4 | 7621.998 | 7625.3 | 7621.9866 | 6734571 |
| 461-484 | Cr-pAr-pAr-pCr-pGr-pAr-pCr-pCr-pCr-pCr-pUr-pUr-pCr-pCr-pUr | Cyclic phosphorylation | 24 | 1.11 | 100 | 1 | 31.75 | 692.174 | -11 | 7621.9951 | 7625.28 | 7621.9866 | 26578942 |
| 461-484 | Cr-pAr-pAr-pCr-pGr-pAr-pCr-pCr-pCr-pCr-pUr-pUr-pCr-pCr-pUr | Cyclic phosphorylation | 24 | 0.73 | 100 | 1 | 31.76 | 761.492 | -10 | 7621.9922 | 7625.29 | 7621.9866 | 12741299 |
| 461-484 | Cr-pAr-pAr-pCr-pGr-pAr-pCr-pCr-pCr-pCr-pUr-pUr-pCr-pCr-pUr | Cyclic phosphorylation | 24 | 0.99 | 100 | 1 | 31.76 | 1269.826 | -6 | 7621.9941 | 7625.35 | 7621.9866 | 2022017.75 |
| 2621-2645 | Cr-pGr-pGr-pAr-pCr-pUr-pGr-pAr-pCr-pAr-pGr-pUr-pGr-pCr-pU | Cyclic phosphorylation | 25 | 2.1 | 100 | 1 | 32.07 | 725.086 | -11 | 7984.0298 | 7987.47 | 7984.013 | 2001811 |
| 2621-2645 | Cr-pGr-pGr-pAr-pCr-pUr-pGr-pAr-pCr-pAr-pGr-pUr-pGr-pCr-pU | Cyclic phosphorylation | 25 | 1.13 | 100 | 1 | 32.08 | 797.695 | -10 | 7984.022 | 7987.53 | 7984.013 | 1668097.75 |
| 456-480 | Ur-pUr-pCr-pUr-pGr-pCr-pAr-pAr-pCr-pGr-pAr-pCr-pCr-pCr-pCr | Cyclic phosphorylation | 25 | 1.41 | 99.9 | 1 | 32.25 | 793.694 | -10 | 7944.0181 | 7948.05 | 7944.0068 | 1289193.75 |
| 456-480 | Ur-pUr-pCr-pUr-pGr-pCr-pAr-pAr-pCr-pGr-pAr-pCr-pCr-pCr-pCr | Cyclic phosphorylation | 25 | 0 | 99.9 | 1 | 32.25 | 721.449 | -11 | 7944.0068 | 7947.53 | 7944.0068 | 2992705.25 |
| 456-480 | Ur-pUr-pCr-pUr-pGr-pCr-pAr-pAr-pCr-pGr-pAr-pCr-pCr-pCr-pCr | Cyclic phosphorylation | 25 | 1.47 | 99.9 | 1 | 32.26 | 661.328 | -12 | 7944.0186 | 7948.01 | 7944.0068 | 2754439 |
| 3313-3335 | Cr-pCr-pCr-pAr-pCr-pUr-pUr-pUr-pCr-pCr-pUr-pAr-pGr-pAr-pG | Cyclic phosphorylation | 23 | 1.08 | 99.8 | 1.1 | 32.42 | 614.324 | -12 | 7380.9707 | 7383.92 | 7380.9627 | 500582.28 |
| 233-255 | Cr-pAr-pAr-pCr-pGr-pUr-pGr-pAr-pCr-pCr-pUr-pGr-pGr-pUr-pU | Cyclic phosphorylation | 23 | 0.88 | 96.8 | 1 | 32.42 | 666.537 | -11 | 7339.979 | 7343.29 | 7339.9726 | 3968717 |
| 3313-3335 | Cr-pCr-pCr-pAr-pCr-pUr-pUr-pUr-pCr-pCr-pUr-pAr-pGr-pAr-pG | Cyclic phosphorylation | 23 | 3 | 99.8 | 1 | 32.42 | 737.291 | -10 | 7380.9849 | 7384 | 7380.9627 | 3950194.25 |
| 233-255 | Cr-pAr-pAr-pCr-pGr-pUr-pGr-pAr-pCr-pCr-pUr-pGr-pGr-pUr-pU | Cyclic phosphorylation | 23 | 0.61 | 96.8 | 1 | 32.42 | 733.291 | -10 | 7339.9771 | 7343.26 | 7339.9726 | 3702740.75 |
| 3313-3335 | Cr-pCr-pCr-pAr-pCr-pUr-pUr-pUr-pCr-pCr-pUr-pAr-pGr-pAr-pG | Cyclic phosphorylation | 23 | 1.88 | 99.8 | 1 | 32.42 | 670.263 | -11 | 7380.9766 | 7384.16 | 7380.9627 | 4002845.25 |
| 2128-2150 | Ur-pCr-pAr-pUr-pUr-pGr-pCr-pCr-pUr-pAr-pCr-pAr-pCr-pAr-pA | Cyclic phosphorylation | 23 | -0.45 | 94.7 | 1 | 32.46 | 725.684 | -10 | 7263.8931 | 7267 | 7263.8963 | 305721.81 |
| 3914-3939 | Cr-pCr-pCr-pCr-pUr-pUr-pUr-pCr-pCr-pCr-pGr-pUr-pCr-pCr-pUr | Cyclic phosphorylation | 26 | 0.12 | 100 | 1 | 32.58 | 817.797 | -10 | 8185.0317 | 8188.69 | 8185.0307 | 1831387.38 |
| 3914-3939 | Cr-pCr-pCr-pCr-pUr-pUr-pUr-pCr-pCr-pCr-pGr-pUr-pCr-pCr-pUr | Cyclic phosphorylation | 26 | -0.36 | 100 | 1 | 32.58 | 681.33 | -12 | 8185.0278 | 8188.78 | 8185.0307 | 1604825.38 |
| 3914-3939 | Cr-pCr-pCr-pCr-pUr-pUr-pUr-pCr-pCr-pCr-pGr-pUr-pCr-pCr-pUr | Cyclic phosphorylation | 26 | 3.94 | 100 | 1 | 32.61 | 743.452 | -11 | 8185.063 | 8188.64 | 8185.0307 | 3437448.75 |
| 4152-4174 | Cr-pCr-pAr-pGr-pCr-pCr-pAr-pCr-pAr-pCr-pCr-pCr-pUr-pGr-pGr | Cyclic phosphorylation | 23 | 1.04 | 99.8 | 1 | 32.64 | 668.541 | -11 | 7362.0234 | 7365.41 | 7362.0158 | 5579124.5 |
| 4152-4174 | Cr-pCr-pAr-pGr-pCr-pCr-pAr-pCr-pAr-pCr-pCr-pCr-pUr-pGr-pGr | Cyclic phosphorylation | 23 | 1.44 | 99.8 | 1.1 | 32.64 | 612.745 | -12 | 7362.0264 | 7365.34 | 7362.0158 | 2725520.25 |
| 4152-4174 | Cr-pCr-pAr-pGr-pCr-pCr-pAr-pCr-pAr-pCr-pCr-pCr-pUr-pGr-pGr | Cyclic phosphorylation | 23 | 0.98 | 99.8 | 1 | 32.66 | 735.495 | -10 | 7362.0229 | 7365.28 | 7362.0158 | 2597174.5 |
| 3332-3355 | Cr-pGr-pUr-pGr-pUr-pUr-pCr-pGr-pUr-pGr-pUr-pCr-pCr-pAr-pA | Cyclic phosphorylation | 24 | 1.07 | 96.3 | 1 | 32.7 | 695.72 | -11 | 7662.001 | 7665.27 | 7661.9928 | 1133880.62 |
| 997-1020 | Cr-pCr-pAr-pGr-pCr-pAr-pAr-pCr-pUr-pUr-pCr-pCr-pGr-pGr-pG | Cyclic phosphorylation | 24 | 2.18 | 99.2 | 1 | 33.18 | 639.58 | -12 | 7684.0527 | 7687.54 | 7684.036 | 4782152.5 |
| 997-1020 | Cr-pCr-pAr-pGr-pCr-pAr-pAr-pCr-pUr-pUr-pCr-pCr-pGr-pGr-pG | Cyclic phosphorylation | 24 | 1.29 | 99.2 | 1 | 33.2 | 697.906 | -11 | 7684.0459 | 7687.95 | 7684.036 | 9942212 |
| 997-1020 | Cr-pCr-pAr-pGr-pCr-pAr-pAr-pCr-pUr-pUr-pCr-pCr-pGr-pGr-pG | Cyclic phosphorylation | 24 | 1.1 | 99.2 | 1 | 33.2 | 767.697 | -10 | 7684.0444 | 7687.93 | 7684.036 | 3785782 |
| 997-1020 | Cr-pCr-pAr-pGr-pCr-pAr-pAr-pCr-pUr-pUr-pCr-pCr-pGr-pGr-pG | Cyclic phosphorylation | 24 | 2.18 | 99.2 | 1.7 | 33.22 | 1920.755 | -4 | 7684.0527 | 7688.04 | 7684.036 | 2432480.25 |
| 997-1020 | Cr-pCr-pAr-pGr-pCr-pAr-pAr-pCr-pUr-pUr-pCr-pCr-pGr-pGr-pG | Cyclic phosphorylation | 24 | 0.47 | 99.2 | 1.1 | 33.23 | 853.109 | -9 | 7684.0396 | 7687.73 | 7684.036 | 1330191.88 |
| 3460-3484 | Cr-pCr-pGr-pUr-pGr-pUr-pAr-pCr-pGr-pAr-pCr-pCr-pCr-pUr-pC | Cyclic phosphorylation | 25 | 0.89 | 99.5 | 1.1 | 33.27 | 795.799 | -10 | 7966.0571 | 7969.82 | 7966.05 | 2017339.75 |
| 3460-3484 | Cr-pCr-pGr-pUr-pGr-pUr-pAr-pCr-pGr-pAr-pCr-pCr-pCr-pUr-pC | Cyclic phosphorylation | 25 | 1.13 | 99.5 | 1 | 33.3 | 723.453 | -11 | 7966.0591 | 7970.09 | 7966.05 | 4229816.5 |
| 109-131 | Cr-pCr-pAr-pCr-pCr-pAr-pGr-pAr-pAr-pCr-pAr-pCr-pAr-pGr-pCr | Cyclic phosphorylation | 23 | 1.46 | 95.5 | 1.4 | 33.32 | 608.079 | -12 | 7306.0254 | 7309.22 | 7306.0147 | 1167719.75 |
| 109-131 | Cr-pCr-pAr-pCr-pCr-pAr-pGr-pAr-pAr-pCr-pAr-pCr-pAr-pGr-pCr | Cyclic phosphorylation | 23 | 0.93 | 95.5 | 1 | 33.32 | 729.895 | -10 | 7306.0215 | 7309.31 | 7306.0147 | 2227897.25 |
| 2380-2400 | Ar-pGr-pAr-pAr-pCr-pAr-pCr-pCr-pCr-pAr-pAr-pGr-pAr-pGr-pG | Cyclic phosphorylation | 21 | 0.57 | 97.9 | 1 | 33.58 | 756.654 | -9 | 6816.9385 | 6820.03 | 6816.9346 | 1866306.62 |
| 1879-1900 | Cr-pAr-pGr-pUr-pGr-pCr-pUr-pGr-pUr-pAr-pCr-pCr-pAr-pGr-pG | Cyclic phosphorylation | 22 | 0.93 | 100 | 1.1 | 33.67 | 1784.485 | -4 | 7138.9614 | 7142.08 | 7138.9548 | 1961316.88 |
| 1879-1900 | Cr-pAr-pGr-pUr-pGr-pCr-pUr-pGr-pUr-pAr-pCr-pCr-pAr-pGr-pG | Cyclic phosphorylation | 22 | 0.38 | 100 | 1 | 33.67 | 713.189 | -10 | 7138.9575 | 7142.13 | 7138.9548 | 5984548.5 |
| 1879-1900 | Cr-pAr-pGr-pUr-pGr-pCr-pUr-pGr-pUr-pAr-pCr-pCr-pAr-pGr-pG | Cyclic phosphorylation | 22 | 1.68 | 100 | 1 | 33.67 | 648.262 | -11 | 7138.9668 | 7142.12 | 7138.9548 | 7522855 |
| 1879-1900 | Cr-pAr-pGr-pUr-pGr-pCr-pUr-pGr-pUr-pAr-pCr-pCr-pAr-pGr-pG | Cyclic phosphorylation | 22 | 1.27 | 100 | 1 | 33.68 | 792.545 | -9 | 7138.9639 | 7142.13 | 7138.9548 | 2188150.5 |
| 232-255 | Gr-pCr-pAr-pAr-pCr-pGr-pUr-pGr-pAr-pCr-pCr-pUr-pGr-pGr-pU | Cyclic phosphorylation | 24 | 1.72 | 100 | 1.4 | 33.79 | 1921.002 | -4 | 7685.0332 | 7688.56 | 7685.02 | 1495161.12 |
| 232-255 | Gr-pCr-pAr-pAr-pCr-pGr-pUr-pGr-pAr-pCr-pCr-pUr-pGr-pGr-pU | Cyclic phosphorylation | 24 | 0.96 | 100 | 1 | 33.79 | 697.905 | -11 | 7685.0273 | 7688.42 | 7685.02 | 4298506 |
| 232-255 | Gr-pCr-pAr-pAr-pCr-pGr-pUr-pGr-pAr-pCr-pCr-pUr-pGr-pGr-pU | Cyclic phosphorylation | 24 | 0.83 | 100 | 1 | 33.79 | 767.796 | -10 | 7685.0264 | 7688.4 | 7685.02 | 6593540.5 |
| 232-255 | Gr-pCr-pAr-pAr-pCr-pGr-pUr-pGr-pAr-pCr-pCr-pUr-pGr-pGr-pU | Cyclic phosphorylation | 24 | 1.02 | 100 | 1 | 33.8 | 853.218 | -9 | 7685.0278 | 7688.4 | 7685.02 | 2332068 |

| | | | | | | | | | | | | | |
|---|---|---|---|---|---|---|---|---|---|---|---|---|---|
| 1742-1765 | Gr-pCr-pAr-pGr-pUr-pUr-pUr-pGr-pGr-pCr-pCr-pGr-pGr-pGr-pA | Cyclic phosphorylation | 24 | 0.38 | 99.4 | 1 | 34.03 | 706.813 | -11 | 7782.0142 | 7785.39 | 7782.0112 | 1051802.88 |
| 386-408 | Cr-pAr-pGr-pCr-pAr-pAr-pGr-pAr-pCr-pCr-pCr-pAr-pGr-pAr-pGr | Cyclic phosphorylation | 23 | 1.52 | 100 | 1 | 34.03 | 741.897 | -10 | 7426.0444 | 7429.31 | 7426.0331 | 7539031 |
| 1742-1765 | Gr-pCr-pAr-pGr-pUr-pUr-pUr-pGr-pGr-pCr-pCr-pGr-pGr-pGr-pA | Cyclic phosphorylation | 24 | 1.57 | 99.4 | 1 | 34.04 | 777.495 | -10 | 7782.0234 | 7785.6 | 7782.0112 | 2596446.25 |
| 386-408 | Cr-pAr-pGr-pCr-pAr-pAr-pGr-pAr-pCr-pCr-pCr-pAr-pGr-pAr-pGr | Cyclic phosphorylation | 23 | 0.86 | 100 | 1 | 34.05 | 674.361 | -11 | 7426.0396 | 7429.33 | 7426.0331 | 7586463 |
| 386-408 | Cr-pAr-pGr-pCr-pAr-pAr-pGr-pAr-pCr-pCr-pCr-pAr-pGr-pAr-pGr | Cyclic phosphorylation | 23 | 1.26 | 100 | 1.1 | 34.06 | 617.997 | -12 | 7426.0425 | 7429.32 | 7426.0331 | 2208408 |
| 1742-1765 | Gr-pCr-pAr-pGr-pUr-pUr-pUr-pGr-pGr-pCr-pCr-pGr-pGr-pGr-pA | Cyclic phosphorylation | 24 | 0.25 | 99.4 | 1.1 | 34.07 | 647.661 | -12 | 7782.0132 | 7785.81 | 7782.0112 | 234583.91 |
| 3312-3335 | Gr-pCr-pCr-pCr-pAr-pCr-pUr-pUr-pUr-pCr-pCr-pUr-pAr-pGr-pA | Cyclic phosphorylation | 24 | 0.9 | 100 | 1 | 34.1 | 857.773 | -9 | 7726.0171 | 7729.3 | 7726.0102 | 2628356.75 |
| 3312-3335 | Gr-pCr-pCr-pCr-pAr-pCr-pUr-pUr-pUr-pCr-pCr-pUr-pAr-pGr-pA | Cyclic phosphorylation | 24 | 1.53 | 100 | 1 | 34.1 | 771.895 | -10 | 7726.022 | 7729.3 | 7726.0102 | 5780800 |
| 3312-3335 | Gr-pCr-pCr-pCr-pAr-pCr-pUr-pUr-pUr-pCr-pCr-pUr-pAr-pGr-pA | Cyclic phosphorylation | 24 | 1.72 | 100 | 1.1 | 34.11 | 643.245 | -12 | 7726.0234 | 7729.36 | 7726.0102 | 847450.81 |
| 3312-3335 | Gr-pCr-pCr-pCr-pAr-pCr-pUr-pUr-pUr-pCr-pCr-pUr-pAr-pGr-pA | Cyclic phosphorylation | 24 | 1.85 | 100 | 1 | 34.12 | 701.632 | -11 | 7726.0244 | 7729.23 | 7726.0102 | 5263080 |
| 1843-1866 | Ur-pGr-pAr-pUr-pCr-pAr-pCr-pCr-pCr-pCr-pUr-pGr-pGr-pCr-pA | Cyclic phosphorylation | 24 | 0.78 | 95.1 | 1 | 34.22 | 760.398 | -10 | 7612.0459 | 7615.36 | 7612.04 | 1696099.62 |
| 1843-1866 | Ur-pGr-pAr-pUr-pCr-pAr-pCr-pCr-pCr-pCr-pUr-pGr-pGr-pCr-pA | Cyclic phosphorylation | 24 | -5.13 | 95.1 | 1 | 34.48 | 691.271 | -11 | 7612.001 | 7614.99 | 7612.04 | 1305825.75 |
| 760-785 | Cr-pCr-pCr-pGr-pGr-pUr-pUr-pUr-pCr-pAr-pGr-pAr-pCr-pAr-pC | Cyclic phosphorylation | 26 | 0.87 | 100 | 1.1 | 34.48 | 636.921 | -13 | 8289.0615 | 8292.97 | 8289.0543 | 1854372 |
| 205-231 | Cr-pCr-pCr-pAr-pGr-pGr-pAr-pCr-pCr-pUr-pGr-pUr-pUr-pCr-pC | Cyclic phosphorylation | 27 | 0.48 | 99.4 | 1 | 34.49 | 769.996 | -11 | 8477.0332 | 8480.79 | 8477.0292 | 7518774 |
| 205-231 | Cr-pCr-pCr-pAr-pGr-pGr-pAr-pCr-pCr-pUr-pGr-pUr-pUr-pCr-pC | Cyclic phosphorylation | 27 | 1.28 | 99.4 | 1.2 | 34.49 | 2119.005 | -4 | 8477.04 | 8480.92 | 8477.0292 | 4180142.75 |
| 205-231 | Cr-pCr-pCr-pAr-pGr-pGr-pAr-pCr-pCr-pUr-pGr-pUr-pUr-pCr-pC | Cyclic phosphorylation | 27 | 0.71 | 99.4 | 1 | 34.49 | 705.662 | -12 | 8477.0352 | 8480.78 | 8477.0292 | 19203670 |
| 760-785 | Cr-pCr-pCr-pGr-pGr-pUr-pUr-pUr-pCr-pAr-pGr-pAr-pCr-pAr-pC | Cyclic phosphorylation | 26 | 0.99 | 100 | 1 | 34.5 | 689.998 | -12 | 8289.0625 | 8292.77 | 8289.0543 | 13843035 |
| 760-785 | Cr-pCr-pCr-pGr-pGr-pUr-pUr-pUr-pCr-pAr-pGr-pAr-pCr-pAr-pC | Cyclic phosphorylation | 26 | 0.4 | 100 | 1 | 34.5 | 752.817 | -11 | 8289.0576 | 8292.73 | 8289.0543 | 9530580 |
| 760-785 | Cr-pCr-pCr-pGr-pGr-pUr-pUr-pUr-pCr-pAr-pGr-pAr-pCr-pAr-pC | Cyclic phosphorylation | 26 | 0.99 | 100 | 1.4 | 34.5 | 2072.261 | -4 | 8289.0625 | 8292.75 | 8289.0543 | 2420395 |
| 205-231 | Cr-pCr-pCr-pAr-pGr-pGr-pAr-pCr-pCr-pUr-pGr-pUr-pUr-pCr-pC | Cyclic phosphorylation | 27 | 0.94 | 99.4 | 1 | 34.5 | 604.782 | -14 | 8477.0371 | 8480.91 | 8477.0292 | 4160585.25 |
| 205-231 | Cr-pCr-pCr-pAr-pGr-pGr-pAr-pCr-pCr-pUr-pGr-pUr-pUr-pCr-pC | Cyclic phosphorylation | 27 | 1.28 | 99.4 | 1 | 34.51 | 651.304 | -13 | 8477.04 | 8480.88 | 8477.0292 | 17263472 |
| 205-231 | Cr-pCr-pCr-pAr-pGr-pGr-pAr-pCr-pCr-pUr-pGr-pUr-pUr-pCr-pC | Cyclic phosphorylation | 27 | 0.13 | 99.4 | 1 | 34.51 | 846.997 | -10 | 8477.0303 | 8480.68 | 8477.0292 | 2585881.5 |
| 760-785 | Cr-pCr-pCr-pGr-pGr-pUr-pUr-pUr-pCr-pAr-pGr-pAr-pCr-pAr-pC | Cyclic phosphorylation | 26 | 0.52 | 100 | 1 | 34.51 | 828.2 | -10 | 8289.0586 | 8292.79 | 8289.0543 | 2767613.5 |
| 15-39 | Ur-pUr-pCr-pUr-pUr-pCr-pUr-pGr-pGr-pUr-pCr-pCr-pCr-pCr-pA | Cyclic phosphorylation | 25 | 0.12 | 96.1 | 1 | 34.79 | 658.66 | -12 | 7913.002 | 7916.41 | 7913.001 | 3086402 |
| 1241-1265 | Cr-pGr-pCr-pCr-pGr-pAr-pCr-pAr-pGr-pCr-pUr-pUr-pCr-pGr-pU | Cyclic phosphorylation | 25 | -0.38 | 100 | 1 | 34.94 | 734.454 | -11 | 8086.0654 | 8089.68 | 8086.0685 | 3815035.5 |
| 1241-1265 | Cr-pGr-pCr-pCr-pGr-pAr-pCr-pAr-pGr-pCr-pUr-pUr-pCr-pGr-pU | Cyclic phosphorylation | 25 | 0.83 | 100 | 1 | 34.94 | 808 | -10 | 8086.0752 | 8089.71 | 8086.0685 | 5961125 |
| 1241-1265 | Cr-pGr-pCr-pCr-pGr-pAr-pCr-pAr-pGr-pCr-pUr-pUr-pCr-pGr-pU | Cyclic phosphorylation | 25 | -0.32 | 100 | 1 | 34.96 | 897.89 | -9 | 8086.0659 | 8089.92 | 8086.0685 | 2258684.25 |
| 1370-1393 | Cr-pAr-pAr-pCr-pCr-pUr-pGr-pGr-pAr-pCr-pUr-pCr-pCr-pAr-pA | Cyclic phosphorylation | 24 | 0.68 | 99.8 | 1 | 35.17 | 703.635 | -11 | 7748.0586 | 7751.42 | 7748.0534 | 2486980.75 |
| 1370-1393 | Cr-pAr-pAr-pCr-pCr-pUr-pGr-pGr-pAr-pCr-pUr-pCr-pCr-pAr-pA | Cyclic phosphorylation | 24 | 0.42 | 99.8 | 1 | 35.17 | 774.099 | -10 | 7748.0566 | 7751.63 | 7748.0534 | 1458734 |
| 4120-4143 | Cr-pUr-pAr-pUr-pAr-pCr-pUr-pAr-pAr-pCr-pCr-pCr-pCr-pAr-pG | Cyclic phosphorylation | 24 | 0.39 | 100 | 1 | 35.46 | 766.393 | -10 | 7670.9961 | 7674.45 | 7670.9931 | 1893096.88 |
| 4120-4143 | Cr-pUr-pAr-pUr-pAr-pCr-pUr-pAr-pAr-pCr-pCr-pCr-pCr-pAr-pG | Cyclic phosphorylation | 24 | 2.04 | 100 | 1.6 | 35.46 | 1917.494 | -4 | 7671.0088 | 7674.64 | 7670.9931 | 1694618.38 |
| 4120-4143 | Cr-pUr-pAr-pUr-pAr-pCr-pUr-pAr-pAr-pCr-pCr-pCr-pCr-pAr-pG | Cyclic phosphorylation | 24 | 0.96 | 100 | 1 | 35.46 | 696.629 | -11 | 7671.0005 | 7674.47 | 7670.9931 | 5466983.5 |
| 4120-4143 | Cr-pUr-pAr-pUr-pAr-pCr-pUr-pAr-pAr-pCr-pCr-pCr-pCr-pAr-pG | Cyclic phosphorylation | 24 | 0.9 | 100 | 1 | 35.46 | 851.659 | -9 | 7671 | 7674.45 | 7670.9931 | 755707.12 |
| 4120-4143 | Cr-pUr-pAr-pUr-pAr-pCr-pUr-pAr-pAr-pCr-pCr-pCr-pCr-pAr-pG | Cyclic phosphorylation | 24 | 1.28 | 100 | 1 | 35.48 | 638.493 | -12 | 7671.0029 | 7674.44 | 7670.9931 | 7317955.5 |
| 4120-4143 | Cr-pUr-pAr-pUr-pAr-pCr-pUr-pAr-pAr-pCr-pCr-pCr-pCr-pAr-pG | Cyclic phosphorylation | 24 | 0.96 | 100 | 1.2 | 35.48 | 589.224 | -13 | 7671.0005 | 7674.43 | 7670.9931 | 1727476.75 |
| 3911-3939 | Cr-pUr-pGr-pCr-pCr-pCr-pCr-pUr-pUr-pCr-pCr-pCr-pCr-pGr-pU | Cyclic phosphorylation | 29 | 1.36 | 92.3 | 1.2 | 35.64 | 702.389 | -13 | 9141.1572 | 9145.19 | 9141.1448 | 161520.72 |
| 205-232 | Cr-pCr-pCr-pAr-pGr-pGr-pAr-pCr-pCr-pUr-pGr-pUr-pUr-pCr-pC | Cyclic phosphorylation | 28 | 0.73 | 99.9 | 1 | 35.84 | 801.364 | -11 | 8822.083 | 8826.02 | 8822.0766 | 1559616 |
| 205-232 | Cr-pCr-pCr-pAr-pGr-pGr-pAr-pCr-pCr-pUr-pGr-pUr-pUr-pCr-pC | Cyclic phosphorylation | 28 | -0.05 | 99.9 | 1 | 35.84 | 734.5 | -12 | 8822.0762 | 8826.11 | 8822.0766 | 3069625.5 |
| 205-232 | Cr-pCr-pCr-pAr-pGr-pGr-pAr-pCr-pCr-pUr-pGr-pUr-pUr-pCr-pC | Cyclic phosphorylation | 28 | 0.28 | 99.9 | 1.1 | 35.86 | 677.846 | -13 | 8822.0791 | 8826.04 | 8822.0766 | 2012166.12 |
| 2306-2332 | Cr-pCr-pUr-pGr-pCr-pUr-pGr-pCr-pUr-pGr-pCr-pAr-pGr-pUr-pA | Cyclic phosphorylation | 27 | 0.48 | 98.7 | 1 | 36.1 | 718.834 | -12 | 8635.0898 | 8639.22 | 8635.0857 | 3628721.5 |
| 2306-2332 | Cr-pCr-pUr-pGr-pCr-pUr-pGr-pCr-pUr-pGr-pCr-pAr-pGr-pUr-pA | Cyclic phosphorylation | 27 | 0.7 | 98.7 | 1.1 | 36.12 | 663.539 | -13 | 8635.0918 | 8639.08 | 8635.0857 | 2191872.25 |
| 563-590 | Cr-pGr-pUr-pGr-pUr-pCr-pCr-pCr-pAr-pGr-pCr-pCr-pUr-pUr-pU | Cyclic phosphorylation | 28 | 0.72 | 100 | 1 | 36.31 | 684.002 | -13 | 8901.1113 | 8905.14 | 8901.1049 | 3677315 |
| 563-590 | Cr-pGr-pUr-pGr-pUr-pCr-pCr-pCr-pAr-pGr-pCr-pCr-pUr-pUr-pU | Cyclic phosphorylation | 28 | 1.05 | 100 | 1 | 36.31 | 808.549 | -11 | 8901.1143 | 8905.12 | 8901.1049 | 2711704.25 |
| 563-590 | Cr-pGr-pUr-pGr-pUr-pCr-pCr-pCr-pAr-pGr-pCr-pCr-pUr-pUr-pU | Cyclic phosphorylation | 28 | 0.83 | 100 | 1 | 36.31 | 741.002 | -12 | 8901.1123 | 8905.1 | 8901.1049 | 6270847.5 |
| 563-590 | Cr-pGr-pUr-pGr-pUr-pCr-pCr-pCr-pAr-pGr-pCr-pCr-pUr-pUr-pU | Cyclic phosphorylation | 28 | 0.61 | 100 | 1.2 | 36.33 | 635.073 | -14 | 8901.1104 | 8904.92 | 8901.1049 | 1290067.25 |
| 3934-3962 | Cr-pCr-pCr-pCr-pGr-pAr-pGr-pUr-pCr-pUr-pCr-pCr-pCr-pCr-pC | Cyclic phosphorylation | 29 | 0.98 | 100 | 1 | 36.62 | 762.761 | -12 | 9162.2129 | 9166.29 | 9162.204 | 3284687 |
| 3934-3962 | Cr-pCr-pCr-pCr-pGr-pAr-pGr-pUr-pCr-pUr-pCr-pCr-pCr-pCr-pC | Cyclic phosphorylation | 29 | 1.08 | 100 | 1 | 36.62 | 704.01 | -13 | 9162.2139 | 9166.34 | 9162.204 | 3950697 |
| 3934-3962 | Cr-pCr-pCr-pCr-pGr-pAr-pGr-pUr-pCr-pUr-pCr-pCr-pCr-pCr-pC | Cyclic phosphorylation | 29 | 1.19 | 100 | 1 | 36.64 | 832.285 | -11 | 9162.2148 | 9166.39 | 9162.204 | 1041659.31 |
| 2758-2783 | Ur-pGr-pGr-pCr-pCr-pUr-pAr-pCr-pCr-pGr-pGr-pUr-pUr-pCr-pA | Cyclic phosphorylation | 26 | -0.03 | 91.4 | 1 | 37.14 | 697.335 | -12 | 8376.0986 | 8379.75 | 8376.0989 | 2491147.5 |
| 2758-2783 | Ur-pGr-pGr-pCr-pCr-pUr-pAr-pCr-pCr-pGr-pGr-pUr-pUr-pCr-pA | Cyclic phosphorylation | 26 | 0.67 | 91.4 | 1.2 | 37.16 | 643.54 | -13 | 8376.1045 | 8379.9 | 8376.0989 | 1660136.5 |
| 2758-2783 | Ur-pGr-pGr-pCr-pCr-pUr-pAr-pCr-pCr-pGr-pGr-pUr-pUr-pCr-pA | Cyclic phosphorylation | 26 | -0.73 | 91.4 | 1 | 37.16 | 760.73 | -11 | 8376.0928 | 8379.79 | 8376.0989 | 1955230.62 |
| 502-521 | Ar-pGr-pAr-pGr-pCr-pUr-pGr-pGr-pAr-pUr-pGr-pGr-pAr-pAr-pA | Cyclic phosphorylation | 20 | 5.52 | 93.4 | 1.1 | 37.21 | 946.56 | -7 | 6630.9644 | 6634 | 6630.9277 | 757524.75 |
| 502-521 | Ar-pGr-pAr-pGr-pCr-pUr-pGr-pGr-pAr-pUr-pGr-pGr-pAr-pAr-pA | Cyclic phosphorylation | 20 | 5.67 | 93.4 | 1.1 | 37.23 | 828.239 | -8 | 6630.9653 | 6633.8 | 6630.9277 | 1887853.12 |
| 502-521 | Ar-pGr-pAr-pGr-pCr-pUr-pGr-pGr-pAr-pUr-pGr-pGr-pAr-pAr-pA | Cyclic phosphorylation | 20 | 6.33 | 93.4 | 1.1 | 37.23 | 1657.236 | -4 | 6630.9697 | 6633.97 | 6630.9277 | 1972089.75 |
| 502-521 | Ar-pGr-pAr-pGr-pCr-pUr-pGr-pGr-pAr-pUr-pGr-pGr-pAr-pAr-pA | Cyclic phosphorylation | 20 | 6.48 | 93.4 | 1 | 37.23 | 662.39 | -10 | 6630.9707 | 6633.83 | 6630.9277 | 9514599 |
| 502-521 | Ar-pGr-pAr-pGr-pCr-pUr-pGr-pGr-pAr-pUr-pGr-pGr-pAr-pAr-pA | Cyclic phosphorylation | 20 | 5.67 | 93.4 | 1.1 | 37.23 | 735.989 | -9 | 6630.9653 | 6633.8 | 6630.9277 | 5681199 |
| 502-521 | Ar-pGr-pAr-pGr-pCr-pUr-pGr-pGr-pAr-pUr-pGr-pGr-pAr-pAr-pA | Cyclic phosphorylation | 20 | 6.33 | 93.4 | 1.3 | 37.23 | 2210.318 | -3 | 6630.9697 | 6633.99 | 6630.9277 | 2100001.25 |
| 518-543 | Cr-pGr-pAr-pGr-pUr-pUr-pCr-pCr-pGr-pGr-pUr-pGr-pUr-pA | Cyclic phosphorylation | 26 | -2.66 | 100 | 1 | 37.3 | 840.805 | -10 | 8415.0986 | 8418.94 | 8415.121 | 1338059.12 |
| 518-543 | Cr-pGr-pAr-pGr-pUr-pUr-pCr-pCr-pGr-pGr-pUr-pGr-pUr-pA | Cyclic phosphorylation | 26 | 0.71 | 100 | 1 | 37.3 | 764.277 | -11 | 8415.127 | 8418.89 | 8415.121 | 2308637 |
| 4212-4229 | Cr-pUr-pAr-pAr-pAr-pAr-pAr-pAr-pAr-pAr-pAr-pAr-pAr-pAr-pA | Cyclic phosphorylation | 18 | 1.05 | 95.5 | 1.4 | 37.4 | 652.206 | -9 | 5875.9131 | 5878.47 | 5875.9069 | 593632.88 |
| 4212-4229 | Cr-pUr-pAr-pAr-pAr-pAr-pAr-pAr-pAr-pAr-pAr-pAr-pAr-pAr-pA | Cyclic phosphorylation | 18 | -1.44 | 95.5 | 2 | 37.42 | 838.694 | -7 | 5875.8984 | 5878.26 | 5875.9069 | 110746.23 |
| 3311-3335 | Ar-pGr-pCr-pCr-pCr-pAr-pCr-pUr-pUr-pUr-pCr-pCr-pUr-pAr-pG | Cyclic phosphorylation | 25 | 0.65 | 93.9 | 1 | 37.61 | 731.545 | -11 | 8055.0679 | 8058.73 | 8055.0627 | 1233843.62 |
| 563-591 | Cr-pGr-pUr-pGr-pUr-pCr-pCr-pCr-pAr-pGr-pCr-pCr-pUr-pUr-pU | Cyclic phosphorylation | 29 | -0.52 | 100 | 1 | 37.64 | 710.621 | -13 | 9246.1475 | 9250.28 | 9246.1523 | 1762017.62 |
| 563-591 | Cr-pGr-pUr-pGr-pUr-pCr-pCr-pCr-pAr-pGr-pCr-pCr-pUr-pUr-pU | Cyclic phosphorylation | 29 | 0.95 | 100 | 1 | 37.64 | 839.917 | -11 | 9246.1611 | 9250.3 | 9246.1523 | 1300724.5 |
| 563-591 | Cr-pGr-pUr-pGr-pUr-pCr-pCr-pCr-pAr-pGr-pCr-pCr-pUr-pUr-pU | Cyclic phosphorylation | 29 | 0.43 | 100 | 1 | 37.66 | 769.839 | -12 | 9246.1562 | 9250.32 | 9246.1523 | 3324564.25 |
| 1876-1900 | Ur-pGr-pGr-pCr-pAr-pGr-pUr-pGr-pCr-pUr-pGr-pUr-pAr-pGr-pC | Cyclic phosphorylation | 25 | 0.33 | 91.8 | 1.1 | 37.66 | 738.818 | -11 | 8135.0776 | 8138.61 | 8135.075 | 3300825.5 |
| 563-591 | Cr-pGr-pUr-pGr-pUr-pCr-pCr-pCr-pAr-pGr-pCr-pCr-pUr-pUr-pU | Cyclic phosphorylation | 29 | 1.06 | 100 | 1.2 | 37.68 | 659.647 | -14 | 9246.1621 | 9249.22 | 9246.1523 | 256296.72 |
| 1876-1900 | Ur-pGr-pGr-pCr-pAr-pGr-pUr-pGr-pCr-pUr-pGr-pUr-pAr-pGr-pC | Cyclic phosphorylation | 25 | 0.39 | 91.8 | 1 | 37.68 | 677.167 | -12 | 8135.0781 | 8138.75 | 8135.075 | 3252657.75 |
| 4120-4144 | Cr-pUr-pAr-pUr-pAr-pCr-pUr-pAr-pAr-pCr-pCr-pCr-pCr-pAr-pG | Cyclic phosphorylation | 25 | 0.89 | 94.4 | 1.6 | 37.9 | 614.612 | -13 | 8000.0527 | 8003.51 | 8000.0456 | 905281.12 |
| 4120-4144 | Cr-pUr-pAr-pUr-pAr-pCr-pUr-pAr-pAr-pCr-pCr-pCr-pCr-pAr-pG | Cyclic phosphorylation | 25 | 1.74 | 94.4 | 1 | 37.9 | 726.543 | -11 | 8000.0596 | 8003.45 | 8000.0456 | 4001359 |
| 4120-4144 | Cr-pUr-pAr-pUr-pAr-pCr-pUr-pAr-pAr-pCr-pCr-pCr-pCr-pAr-pG | Cyclic phosphorylation | 25 | 1.01 | 94.4 | 1 | 37.9 | 665.914 | -12 | 8000.0537 | 8003.46 | 8000.0456 | 4269499.5 |
| 702-731 | Cr-pCr-pUr-pCr-pAr-pGr-pGr-pGr-pCr-pUr-pUr-pCr-pUr-pCr-pU | Cyclic phosphorylation | 30 | 2.47 | 100 | 1 | 37.9 | 731.629 | -13 | 9511.2109 | 9518.22 | 9511.1875 | 4343095.5 |
| 702-731 | Cr-pCr-pUr-pCr-pAr-pGr-pGr-pGr-pCr-pUr-pUr-pCr-pUr-pCr-pU | Cyclic phosphorylation | 30 | 1.55 | 100 | 1.4 | 37.92 | 633.273 | -15 | 9511.2021 | 9515.64 | 9511.1875 | 1993640.75 |
| 702-731 | Cr-pCr-pUr-pCr-pAr-pGr-pGr-pGr-pCr-pUr-pUr-pCr-pUr-pCr-pU | Cyclic phosphorylation | 30 | 1.85 | 100 | 1 | 37.92 | 730.931 | -13 | 9511.2051 | 9515.57 | 9511.1875 | 6467640 |
| 702-731 | Cr-pCr-pUr-pCr-pAr-pGr-pGr-pGr-pCr-pUr-pUr-pCr-pUr-pCr-pU | Cyclic phosphorylation | 30 | 2.47 | 100 | 1 | 37.93 | 792.009 | -12 | 9511.2109 | 9515.27 | 9511.1875 | 4922467.5 |
| 702-731 | Cr-pCr-pUr-pCr-pAr-pGr-pGr-pGr-pCr-pUr-pUr-pCr-pUr-pCr-pU | Cyclic phosphorylation | 30 | 0.31 | 100 | 1 | 37.94 | 678.65 | -14 | 9511.1904 | 9515.45 | 9511.1875 | 4794416 |
| 1679-1706 | Cr-pUr-pUr-pCr-pAr-pAr-pCr-pGr-pGr-pCr-pCr-pUr-pGr-pAr-pC | Cyclic phosphorylation | 28 | 0.44 | 100 | 1 | 37.96 | 691.7 | -13 | 9001.1963 | 9005.19 | 9001.1923 | 4116217.5 |
| 1679-1706 | Cr-pUr-pUr-pCr-pAr-pAr-pCr-pGr-pGr-pCr-pCr-pUr-pGr-pAr-pC | Cyclic phosphorylation | 28 | -0.21 | 100 | 1 | 37.97 | 749.426 | -12 | 9001.1904 | 9005.32 | 9001.1923 | 2449986 |
| 2212-2238 | Ur-pCr-pAr-pGr-pCr-pGr-pUr-pGr-pAr-pCr-pCr-pAr-pCr-pAr-pG | Cyclic phosphorylation | 27 | 2.06 | 99.8 | 1 | 38.51 | 665.851 | -13 | 8665.1631 | 8668.96 | 8665.1452 | 1406928.5 |
| 2212-2238 | Ur-pCr-pAr-pGr-pCr-pGr-pUr-pGr-pAr-pCr-pCr-pAr-pCr-pAr-pG | Cyclic phosphorylation | 27 | 0.48 | 99.8 | 1 | 38.65 | 721.339 | -12 | 8665.1494 | 8668.94 | 8665.1452 | 2036235.12 |
| 702-732 | Cr-pCr-pUr-pCr-pAr-pGr-pGr-pGr-pCr-pUr-pUr-pCr-pUr-pCr-pU | Cyclic phosphorylation | 31 | 2.72 | 100 | 1 | 39.02 | 820.68 | -12 | 9856.2617 | 9860.7 | 9856.2349 | 6384784.5 |
| 702-732 | Cr-pCr-pUr-pCr-pAr-pGr-pGr-pGr-pCr-pUr-pUr-pCr-pUr-pCr-pU | Cyclic phosphorylation | 31 | -7.09 | 100 | 1 | 39.02 | 895.471 | -11 | 9856.165 | 9860.5 | 9856.2349 | 1240278 |
| 702-732 | Cr-pCr-pUr-pCr-pAr-pGr-pGr-pGr-pCr-pUr-pUr-pCr-pUr-pCr-pU | Cyclic phosphorylation | 31 | 1.34 | 100 | 1 | 39.03 | 757.473 | -13 | 9856.248 | 9860.59 | 9856.2349 | 13225843 |
| 702-732 | Cr-pCr-pUr-pCr-pAr-pGr-pGr-pGr-pCr-pUr-pUr-pCr-pUr-pCr-pU | Cyclic phosphorylation | 31 | 1.63 | 100 | 1 | 39.04 | 703.296 | -14 | 9856.251 | 9860.74 | 9856.2349 | 5844590.5 |
| 533-559 | Cr-pAr-pGr-pCr-pGr-pCr-pGr-pCr-pCr-pAr-pAr-pCr-pAr-pAr-pA | Cyclic phosphorylation | 27 | -1.12 | 95.4 | 1 | 39.11 | 784.011 | -11 | 8631.1777 | 8634.91 | 8631.1874 | 1319078.25 |
| 563-592 | Cr-pGr-pUr-pGr-pUr-pCr-pCr-pCr-pAr-pGr-pCr-pCr-pUr-pUr-pU | Cyclic phosphorylation | 30 | -0.08 | 100 | 1 | 39.61 | 683.223 | -14 | 9575.2041 | 9579.45 | 9575.2048 | 1401591.88 |
| 563-592 | Cr-pGr-pUr-pGr-pUr-pCr-pCr-pCr-pAr-pGr-pCr-pCr-pUr-pUr-pU | Cyclic phosphorylation | 30 | 1.66 | 100 | 1 | 39.61 | 735.856 | -13 | 9575.2207 | 9579.64 | 9575.2048 | 2766543 |
| 563-592 | Cr-pGr-pUr-pGr-pUr-pCr-pCr-pCr-pAr-pGr-pCr-pCr-pUr-pUr-pU | Cyclic phosphorylation | 30 | -0.79 | 100 | 1 | 39.61 | 797.261 | -12 | 9575.1973 | 9579.51 | 9575.2048 | 1683935 |
| 1826-1855 | Cr-pUr-pUr-pCr-pGr-pGr-pCr-pGr-pGr-pAr-pGr-pUr-pGr-pUr-pC | Cyclic phosphorylation | 30 | -1.81 | 100 | 1.1 | 39.77 | 687.223 | -14 | 9631.1885 | 9635.68 | 9631.2059 | 600645.69 |
| 1826-1855 | Cr-pUr-pUr-pCr-pGr-pGr-pCr-pGr-pGr-pAr-pGr-pUr-pGr-pUr-pC | Cyclic phosphorylation | 30 | 0.22 | 100 | 1 | 39.77 | 740.086 | -13 | 9631.208 | 9635.32 | 9631.2059 | 2025426.38 |
| 186-215 | Ur-pCr-pCr-pAr-pGr-pCr-pGr-pUr-pGr-pCr-pUr-pGr-pCr-pAr-pC | Cyclic phosphorylation | 30 | 0.13 | 100 | 1 | 40.15 | 795.764 | -12 | 9557.2432 | 9561.53 | 9557.2419 | 1830331.12 |
| 186-215 | Ur-pCr-pCr-pAr-pGr-pCr-pGr-pUr-pGr-pCr-pUr-pGr-pCr-pAr-pC | Cyclic phosphorylation | 30 | 0.64 | 100 | 1 | 40.17 | 734.474 | -13 | 9557.248 | 9561.45 | 9557.2419 | 3837861.75 |
| 186-215 | Ur-pCr-pCr-pAr-pGr-pCr-pGr-pUr-pGr-pCr-pUr-pGr-pCr-pAr-pC | Cyclic phosphorylation | 30 | 1.05 | 100 | 1 | 40.17 | 681.94 | -14 | 9557.252 | 9561.49 | 9557.2419 | 2381546.5 |
| 186-215 | Ur-pCr-pCr-pAr-pGr-pCr-pGr-pUr-pGr-pCr-pUr-pGr-pCr-pAr-pC | Cyclic phosphorylation | 30 | 0.03 | 100 | 1.2 | 40.17 | 868.198 | -11 | 9557.2422 | 9561.52 | 9557.2419 | 513725.31 |
| 1370-1396 | Cr-pAr-pAr-pCr-pCr-pUr-pGr-pGr-pAr-pCr-pUr-pCr-pCr-pAr-pA | Cyclic phosphorylation | 27 | -0.39 | 100 | 1 | 40.69 | 791.285 | -11 | 8711.1963 | 8715.36 | 8711.1997 | 2754202 |
| 1370-1396 | Cr-pAr-pAr-pCr-pCr-pUr-pGr-pGr-pAr-pCr-pUr-pCr-pCr-pAr-pA | Cyclic phosphorylation | 27 | 1.4 | 100 | 1 | 40.69 | 725.26 | -12 | 8711.2119 | 8715.23 | 8711.1997 | 5169799 |
| 1370-1396 | Cr-pAr-pAr-pCr-pCr-pUr-pGr-pGr-pAr-pCr-pUr-pCr-pCr-pAr-pA | Cyclic phosphorylation | 27 | 1.07 | 100 | 1.1 | 40.69 | 669.317 | -13 | 8711.209 | 8715.26 | 8711.1997 | 3215138.75 |
| 200-231 | Cr-pUr-pCr-pUr-pAr-pCr-pCr-pCr-pAr-pGr-pGr-pAr-pCr-pCr-pU | Cyclic phosphorylation | 32 | 1.36 | 100 | 1.1 | 40.9 | 835.012 | -12 | 10028.2285 | 10032.78 | 10028.2149 | 1435109.75 |
| 200-231 | Cr-pUr-pCr-pUr-pAr-pCr-pCr-pCr-pAr-pGr-pGr-pAr-pCr-pCr-pU | Cyclic phosphorylation | 32 | 0.68 | 100 | 1 | 40.91 | 667.808 | -15 | 10028.2217 | 10032.76 | 10028.2149 | 12945042 |
| 200-231 | Cr-pUr-pCr-pUr-pAr-pCr-pCr-pCr-pAr-pGr-pGr-pAr-pCr-pCr-pU | Cyclic phosphorylation | 32 | 0.87 | 100 | 1.1 | 40.91 | 626.007 | -16 | 10028.2236 | 10032.71 | 10028.2149 | 6546049 |
| 200-231 | Cr-pUr-pCr-pUr-pAr-pCr-pCr-pCr-pAr-pGr-pGr-pAr-pCr-pCr-pU | Cyclic phosphorylation | 32 | 0.29 | 100 | 1 | 40.91 | 715.58 | -14 | 10028.2178 | 10032.79 | 10028.2149 | 10059151 |
| 200-231 | Cr-pUr-pCr-pUr-pAr-pCr-pCr-pCr-pAr-pGr-pGr-pAr-pCr-pCr-pU | Cyclic phosphorylation | 32 | 0.29 | 100 | 1 | 40.91 | 770.702 | -13 | 10028.2178 | 10032.64 | 10028.2149 | 3759915.25 |
| 200-231 | Cr-pUr-pCr-pUr-pAr-pCr-pCr-pCr-pAr-pGr-pGr-pAr-pCr-pCr-pU | Cyclic phosphorylation | 32 | 0.49 | 100 | 1.3 | 40.91 | 589.124 | -17 | 10028.2197 | 10032.75 | 10028.2149 | 1894745 |
| 293-323 | Cr-pAr-pAr-pCr-pCr-pCr-pGr-pUr-pGr-pCr-pUr-pGr-pCr-pCr-pC | Cyclic phosphorylation | 31 | 0.11 | 100 | 1 | 41.13 | 764.018 | -13 | 9941.3125 | 9945.8 | 9941.3115 | 4574732 |
| 293-323 | Cr-pAr-pAr-pCr-pCr-pCr-pGr-pUr-pGr-pCr-pUr-pGr-pCr-pCr-pC | Cyclic phosphorylation | 31 | 1.19 | 100 | 1 | 41.14 | 709.373 | -14 | 9941.3232 | 9945.64 | 9941.3115 | 2235725 |
| 293-323 | Cr-pAr-pAr-pCr-pCr-pCr-pGr-pUr-pGr-pCr-pUr-pGr-pCr-pCr-pC | Cyclic phosphorylation | 31 | 0.99 | 100 | 1 | 41.15 | 827.687 | -12 | 9941.3213 | 9945.77 | 9941.3115 | 1837144.38 |
| 2345-2372 | Ur-pAr-pGr-pAr-pGr-pCr-pCr-pCr-pUr-pGr-pAr-pCr-pAr-pGr-pG | Cyclic phosphorylation | 28 | 0.56 | 90.4 | 1 | 41.41 | 758.68 | -12 | 9113.2373 | 9117.33 | 9113.2322 | 3407766.25 |
| 2345-2372 | Ur-pAr-pGr-pAr-pGr-pCr-pCr-pCr-pUr-pGr-pAr-pCr-pAr-pGr-pG | Cyclic phosphorylation | 28 | 1.31 | 90.4 | 1 | 41.41 | 700.242 | -13 | 9113.2441 | 9117.21 | 9113.2322 | 5271076.5 |
| 2345-2372 | Ur-pAr-pGr-pAr-pGr-pCr-pCr-pCr-pUr-pGr-pAr-pCr-pAr-pGr-pG | Cyclic phosphorylation | 28 | -2.55 | 90.4 | 1 | 41.43 | 827.742 | -11 | 9113.209 | 9117.54 | 9113.2322 | 812853.5 |
| 381-408 | Cr-pUr-pGr-pAr-pCr-pAr-pGr-pCr-pAr-pAr-pGr-pAr-pCr-pCr-pC | Cyclic phosphorylation | 28 | -0.01 | 100 | 1 | 41.47 | 695.859 | -13 | 9056.2471 | 9060.32 | 9056.2471 | 2586376.5 |
| 381-408 | Cr-pUr-pGr-pAr-pCr-pAr-pGr-pCr-pAr-pAr-pGr-pAr-pCr-pCr-pC | Cyclic phosphorylation | 28 | 0.1 | 100 | 1 | 41.5 | 753.931 | -12 | 9056.248 | 9060.31 | 9056.2471 | 3453360.5 |
| 104-132 | Cr-pCr-pUr-pGr-pCr-pAr-pCr-pCr-pAr-pGr-pAr-pAr-pCr-pC | Cyclic phosphorylation | 29 | -0.57 | 99.1 | 1.5 | 41.67 | 839.381 | -11 | 9241.2646 | 9245.08 | 9241.27 | 218129.2 |
| 104-132 | Cr-pCr-pUr-pGr-pCr-pAr-pCr-pCr-pAr-pGr-pAr-pAr-pCr-pC | Cyclic phosphorylation | 29 | -0.26 | 99.1 | 1.8 | 41.69 | 710.169 | -13 | 9241.2676 | 9245.43 | 9241.27 | 853238.56 |
| 104-132 | Cr-pCr-pUr-pGr-pCr-pAr-pCr-pCr-pAr-pGr-pAr-pAr-pCr-pC | Cyclic phosphorylation | 29 | -0.05 | 99.1 | 1.2 | 41.69 | 769.35 | -12 | 9241.2695 | 9245.57 | 9241.27 | 776142.06 |
| 563-593 | Cr-pGr-pUr-pGr-pUr-pCr-pCr-pCr-pAr-pGr-pCr-pCr-pUr-pUr-pU | Cyclic phosphorylation | 31 | 2.81 | 97.8 | 1.1 | 41.73 | 706.798 | -14 | 9904.2852 | 9908.44 | 9904.2574 | 1372822.62 |
| 563-593 | Cr-pGr-pUr-pGr-pUr-pCr-pCr-pCr-pAr-pGr-pCr-pCr-pUr-pUr-pU | Cyclic phosphorylation | 31 | 1.53 | 97.8 | 1.1 | 41.74 | 761.168 | -13 | 9904.2725 | 9908.71 | 9904.2574 | 1784030.62 |
| 755-785 | Cr-pAr-pUr-pCr-pAr-pCr-pCr-pCr-pGr-pGr-pUr-pUr-pUr-pCr-pA | Cyclic phosphorylation | 31 | 1.82 | 99 | 1.1 | 41.76 | 656.812 | -15 | 9863.2852 | 9867.67 | 9863.2672 | 1610716.88 |

| | | | | | | | | | | | | | |
|---|---|---|---|---|---|---|---|---|---|---|---|---|---|
| 755-785 | Cr-pAr-pUr-pCr-pAr-pCr-pCr-pCr-pGr-pGr-pUr-pUr-pUr-pCr-pA | Cyclic phosphorylation | 31 | 1.03 | 99 | 1.1 | 41.76 | 703.799 | -14 | 9863.2773 | 9867.81 | 9863.2672 | 2229597.75 |
| 755-785 | Cr-pAr-pUr-pCr-pAr-pCr-pCr-pCr-pGr-pGr-pUr-pUr-pUr-pCr-pA | Cyclic phosphorylation | 31 | 0.63 | 99 | 1 | 41.76 | 757.938 | -13 | 9863.2734 | 9867.63 | 9863.2672 | 1624362.25 |
| 200-232 | Cr-pUr-pCr-pUr-pAr-pCr-pCr-pCr-pAr-pGr-pGr-pAr-pCr-pCr-pUr | Cyclic phosphorylation | 33 | 0.79 | 100 | 1.1 | 41.93 | 797.245 | -13 | 10373.2705 | 10377.95 | 10373.2623 | 916819.69 |
| 200-232 | Cr-pUr-pCr-pUr-pAr-pCr-pCr-pCr-pAr-pGr-pGr-pAr-pCr-pCr-pUr | Cyclic phosphorylation | 33 | 1.64 | 100 | 1.2 | 41.95 | 690.812 | -15 | 10373.2793 | 10377.74 | 10373.2623 | 1289133.38 |
| 200-232 | Cr-pUr-pCr-pUr-pAr-pCr-pCr-pCr-pAr-pGr-pGr-pAr-pCr-pCr-pUr | Cyclic phosphorylation | 33 | -0.15 | 100 | 1.2 | 41.97 | 740.299 | -14 | 10373.2607 | 10378 | 10373.2623 | 1630993 |
| 2992-3022 | Ur-pCr-pCr-pUr-pGr-pAr-pGr-pCr-pAr-pGr-pAr-pCr-pUr-pGr-pG | Cyclic phosphorylation | 31 | 1.04 | 99.9 | 1 | 42.44 | 765.865 | -13 | 9965.333 | 9969.61 | 9965.3227 | 1515360.88 |
| 2992-3022 | Ur-pCr-pCr-pUr-pGr-pAr-pGr-pCr-pAr-pGr-pAr-pCr-pUr-pGr-pG | Cyclic phosphorylation | 31 | 1.72 | 99.9 | 1.1 | 42.45 | 711.16 | -14 | 9965.3398 | 9969.91 | 9965.3227 | 1030898.69 |
| 4212-4231 | Cr-pUr-pAr-pAr-pAr-pAr-pAr-pAr-pAr-pAr-pAr-pAr-pAr-pAr-pA | Cyclic phosphorylation | 20 | 0.64 | 96.6 | 1.2 | 42.45 | 725.329 | -9 | 6534.0161 | 6536.86 | 6534.0119 | 1320350.5 |
| 4212-4231 | Cr-pUr-pAr-pAr-pAr-pAr-pAr-pAr-pAr-pAr-pAr-pAr-pAr-pAr-pA | Cyclic phosphorylation | 20 | 1.31 | 96.6 | 1.2 | 42.46 | 652.695 | -10 | 6534.0205 | 6536.96 | 6534.0119 | 851639.12 |
| 2715-2748 | Ur-pUr-pUr-pGr-pGr-pAr-pGr-pCr-pAr-pGr-pGr-pCr-pGr-pCr-pC | Cyclic phosphorylation | 34 | -0.55 | 97 | 1.1 | 42.96 | 679.017 | -16 | 10876.3604 | 10881.21 | 10876.3663 | 831882.75 |
| 2715-2748 | Ur-pUr-pUr-pGr-pGr-pAr-pGr-pCr-pAr-pGr-pGr-pCr-pGr-pCr-pC | Cyclic phosphorylation | 34 | 1.34 | 97 | 1.1 | 42.97 | 724.352 | -15 | 10876.3809 | 10881.31 | 10876.3663 | 1617700.88 |
| 2715-2748 | Ur-pUr-pUr-pGr-pGr-pAr-pGr-pCr-pAr-pGr-pGr-pCr-pGr-pCr-pC | Cyclic phosphorylation | 34 | -8.54 | 97 | 1.1 | 42.97 | 776.163 | -14 | 10876.2734 | 10881.31 | 10876.3663 | 378649.03 |
| 2015-2041 | Gr-pCr-pAr-pCr-pGr-pUr-pGr-pAr-pAr-pCr-pAr-pAr-pUr-pAr-pG | Cyclic phosphorylation | 27 | 0.23 | 99.9 | 1.2 | 42.99 | 797.194 | -11 | 8776.2031 | 8780.14 | 8776.2011 | 642406.75 |
| 55-90 | Ur-pGr-pUr-pUr-pCr-pGr-pUr-pGr-pUr-pUr-pCr-pCr-pUr-pGr-p | Cyclic phosphorylation | 36 | 1.48 | 96.2 | 1.2 | 43.08 | 763.283 | -15 | 11459.3584 | 11464.43 | 11459.3414 | 1271512.88 |
| 698-732 | Ur-pCr-pUr-pGr-pCr-pCr-pUr-pCr-pAr-pGr-pGr-pGr-pCr-pUr-pU | Cyclic phosphorylation | 35 | -0.98 | 100 | 1.5 | 43.1 | 793.521 | -14 | 11118.3633 | 11123.45 | 11118.3742 | 771331.38 |
| 55-90 | Ur-pGr-pUr-pUr-pCr-pGr-pUr-pGr-pUr-pUr-pCr-pCr-pUr-pGr-p | Cyclic phosphorylation | 36 | 2.51 | 96.2 | 1.6 | 43.1 | 817.876 | -14 | 11459.3701 | 11464.48 | 11459.3414 | 927561.06 |
| 698-732 | Ur-pCr-pUr-pGr-pCr-pCr-pUr-pCr-pAr-pGr-pGr-pGr-pCr-pUr-pU | Cyclic phosphorylation | 35 | -0.63 | 100 | 1.2 | 43.1 | 740.553 | -15 | 11118.3672 | 11123.37 | 11118.3742 | 1031928.69 |
| 2378-2405 | Cr-pAr-pAr-pGr-pAr-pAr-pCr-pAr-pCr-pCr-pCr-pAr-pAr-pGr-pAr | Cyclic phosphorylation | 28 | 1.25 | 99.9 | 1.2 | 43.41 | 819.745 | -11 | 9024.2686 | 9028.36 | 9024.2573 | 593689.25 |
| 2378-2405 | Cr-pAr-pAr-pGr-pAr-pAr-pCr-pAr-pCr-pCr-pCr-pAr-pAr-pGr-pAr | Cyclic phosphorylation | 28 | 0.92 | 99.9 | 1.1 | 43.43 | 693.398 | -13 | 9024.2656 | 9028.14 | 9024.2573 | 2899886.5 |
| 2378-2405 | Cr-pAr-pAr-pGr-pAr-pAr-pCr-pAr-pCr-pCr-pCr-pAr-pAr-pGr-pAr | Cyclic phosphorylation | 28 | 1.68 | 99.9 | 1.1 | 43.44 | 751.349 | -12 | 9024.2725 | 9028.25 | 9024.2573 | 1722442.62 |
| 702-736 | Cr-pCr-pUr-pCr-pAr-pGr-pGr-pGr-pCr-pUr-pUr-pCr-pUr-pCr-pU | Cyclic phosphorylation | 35 | 0.58 | 100 | 1.1 | 44.31 | 657.077 | -17 | 11181.4141 | 11186.55 | 11181.4076 | 1109831.5 |
| 702-736 | Cr-pCr-pUr-pCr-pAr-pGr-pGr-pGr-pCr-pUr-pUr-pCr-pUr-pCr-pU | Cyclic phosphorylation | 35 | 0.67 | 100 | 1 | 44.32 | 744.688 | -15 | 11181.415 | 11186.44 | 11181.4076 | 5212895 |
| 702-736 | Cr-pCr-pUr-pCr-pAr-pGr-pGr-pGr-pCr-pUr-pUr-pCr-pUr-pCr-pU | Cyclic phosphorylation | 35 | 0.32 | 100 | 1 | 44.32 | 798.023 | -14 | 11181.4111 | 11186.31 | 11181.4076 | 3294164.25 |
| 702-736 | Cr-pCr-pUr-pCr-pAr-pGr-pGr-pGr-pCr-pUr-pUr-pCr-pUr-pCr-pU | Cyclic phosphorylation | 35 | -0.29 | 100 | 1.1 | 44.33 | 859.41 | -13 | 11181.4043 | 11186.55 | 11181.4076 | 981227.62 |
| 702-736 | Cr-pCr-pUr-pCr-pAr-pGr-pGr-pGr-pCr-pUr-pUr-pCr-pUr-pCr-pU | Cyclic phosphorylation | 35 | 0.32 | 100 | 1.1 | 44.33 | 698.082 | -16 | 11181.4111 | 11186.51 | 11181.4076 | 4098378.5 |
| 4212-4232 | Cr-pUr-pAr-pAr-pAr-pAr-pAr-pAr-pAr-pAr-pAr-pAr-pAr-pAr-pA | Cyclic phosphorylation | 21 | 1.28 | 95.7 | 1.5 | 44.98 | 685.5 | -10 | 6863.0732 | 6865.91 | 6863.0645 | 1144973.25 |
| 4212-4232 | Cr-pUr-pAr-pAr-pAr-pAr-pAr-pAr-pAr-pAr-pAr-pAr-pAr-pAr-pA | Cyclic phosphorylation | 21 | 1.07 | 95.7 | 1.3 | 44.98 | 761.89 | -9 | 6863.0718 | 6866.1 | 6863.0645 | 837878.5 |
| 2375-2404 | Gr-pGr-pAr-pCr-pAr-pAr-pGr-pAr-pAr-pCr-pAr-pCr-pCr-pCr-pAr | Cyclic phosphorylation | 30 | -0.36 | 90.1 | 1.3 | 45.66 | 882.481 | -11 | 9714.3486 | 9718.58 | 9714.3522 | 401731.91 |
| 2375-2404 | Gr-pGr-pAr-pCr-pAr-pAr-pGr-pAr-pAr-pCr-pAr-pCr-pCr-pCr-pAr | Cyclic phosphorylation | 30 | 0.94 | 90.1 | 1.4 | 45.66 | 808.856 | -12 | 9714.3613 | 9719 | 9714.3522 | 444995.22 |
| 104-136 | Cr-pCr-pUr-pGr-pAr-pCr-pCr-pAr-pCr-pCr-pAr-pGr-pAr-pAr-pCr | Cyclic phosphorylation | 33 | 0.96 | 98.8 | 1.2 | 45.85 | 654.646 | -16 | 10486.4404 | 10491.19 | 10486.4304 | 3808773.75 |
| 104-136 | Cr-pCr-pUr-pGr-pAr-pCr-pCr-pAr-pCr-pCr-pAr-pGr-pAr-pAr-pCr | Cyclic phosphorylation | 33 | 0.22 | 98.8 | 1 | 45.85 | 748.381 | -14 | 10486.4326 | 10490.97 | 10486.4304 | 2258171.5 |
| 104-136 | Cr-pCr-pUr-pGr-pAr-pCr-pCr-pAr-pCr-pCr-pAr-pGr-pAr-pAr-pCr | Cyclic phosphorylation | 33 | 0.12 | 98.8 | 1.1 | 45.85 | 698.356 | -15 | 10486.4316 | 10491.2 | 10486.4304 | 3283260 |
| 104-136 | Cr-pCr-pUr-pGr-pAr-pCr-pCr-pAr-pCr-pCr-pAr-pGr-pAr-pAr-pCr | Cyclic phosphorylation | 33 | 1.24 | 98.8 | 1.7 | 45.85 | 873.196 | -12 | 10486.4434 | 10491.08 | 10486.4304 | 459096.03 |
| 104-136 | Cr-pCr-pUr-pGr-pAr-pCr-pCr-pAr-pCr-pCr-pAr-pGr-pAr-pAr-pCr | Cyclic phosphorylation | 33 | 4.59 | 98.8 | 1.1 | 45.88 | 805.95 | -13 | 10486.4785 | 10491.16 | 10486.4304 | 1189528.62 |
| 186-221 | Ur-pCr-pCr-pAr-pGr-pCr-pGr-pUr-pGr-pCr-pUr-pGr-pCr-pAr-pC | Cyclic phosphorylation | 36 | 2.77 | 97.8 | 1.1 | 45.88 | 761.357 | -15 | 11430.4795 | 11435.71 | 11430.4478 | 984859.94 |
| 186-221 | Ur-pCr-pCr-pAr-pGr-pCr-pGr-pUr-pGr-pCr-pUr-pGr-pCr-pAr-pC | Cyclic phosphorylation | 36 | 1.92 | 97.8 | 1.7 | 45.88 | 671.667 | -17 | 11430.4697 | 11435.67 | 11430.4478 | 474968.84 |
| 186-221 | Ur-pCr-pCr-pAr-pGr-pCr-pGr-pUr-pGr-pCr-pUr-pGr-pCr-pAr-pC | Cyclic phosphorylation | 36 | 0.38 | 97.8 | 1.2 | 45.88 | 713.646 | -16 | 11430.4521 | 11435.69 | 11430.4478 | 991207.56 |
| 288-323 | Ur-pUr-pCr-pGr-pAr-pCr-pAr-pAr-pCr-pCr-pCr-pCr-pGr-pUr-pG | Cyclic phosphorylation | 36 | 2.51 | 98.7 | 1.4 | 46.27 | 823.102 | -14 | 11532.5322 | 11537.53 | 11532.5033 | 775743.81 |
| 288-323 | Ur-pUr-pCr-pGr-pAr-pCr-pAr-pAr-pCr-pCr-pCr-pCr-pGr-pUr-pG | Cyclic phosphorylation | 36 | 1.07 | 98.7 | 1.1 | 46.28 | 720.088 | -16 | 11532.5156 | 11537.51 | 11532.5033 | 1085687.38 |
| 288-323 | Ur-pUr-pCr-pGr-pAr-pCr-pAr-pAr-pCr-pCr-pCr-pCr-pGr-pUr-pG | Cyclic phosphorylation | 36 | -0.37 | 98.7 | 1.2 | 46.28 | 768.094 | -15 | 11532.499 | 11537.87 | 11532.5033 | 1350614.5 |
| 751-785 | Ur-pCr-pAr-pAr-pCr-pAr-pUr-pCr-pAr-pCr-pCr-pCr-pGr-pGr-pU | Cyclic phosphorylation | 35 | 1.55 | 99.9 | 1.1 | 46.75 | 695.084 | -16 | 11132.4561 | 11137.6 | 11132.4388 | 679394.5 |
| 751-785 | Ur-pCr-pAr-pAr-pCr-pAr-pUr-pCr-pAr-pCr-pCr-pCr-pGr-pGr-pU | Cyclic phosphorylation | 35 | 2.07 | 99.9 | 1 | 46.75 | 741.49 | -15 | 11132.4619 | 11137.64 | 11132.4388 | 1104292.62 |
| 4212-4233 | Cr-pUr-pAr-pAr-pAr-pAr-pAr-pAr-pAr-pAr-pAr-pAr-pAr-pAr-pA | Cyclic phosphorylation | 22 | 1.11 | 99.7 | 1.4 | 47.07 | 718.506 | -10 | 7192.125 | 7195.15 | 7192.117 | 1039065.06 |
| 4212-4233 | Cr-pUr-pAr-pAr-pAr-pAr-pAr-pAr-pAr-pAr-pAr-pAr-pAr-pAr-pA | Cyclic phosphorylation | 22 | 1.18 | 99.7 | 1.6 | 47.08 | 798.451 | -9 | 7192.1255 | 7195.32 | 7192.117 | 495365.56 |
| 4212-4233 | Cr-pUr-pAr-pAr-pAr-pAr-pAr-pAr-pAr-pAr-pAr-pAr-pAr-pAr-pA | Cyclic phosphorylation | 22 | 0.37 | 99.7 | 2 | 47.08 | 653.096 | -11 | 7192.1196 | 7195.51 | 7192.117 | 413451.84 |
| 3524-3556 | Gr-pAr-pAr-pCr-pCr-pAr-pCr-pAr-pCr-pAr-pAr-pGr-pCr-pCr-pCr | Cyclic phosphorylation | 33 | 1.65 | 93.1 | 1.6 | 47.27 | 821.185 | -13 | 10684.5107 | 10689.34 | 10684.4931 | 667096.94 |
| 2375-2405 | Gr-pGr-pAr-pCr-pAr-pAr-pGr-pAr-pAr-pCr-pAr-pCr-pCr-pCr-pAr | Cyclic phosphorylation | 31 | -0.72 | 100 | 1.1 | 47.31 | 912.394 | -11 | 10043.3975 | 10047.91 | 10043.4047 | 825750.31 |
| 2375-2405 | Gr-pGr-pAr-pCr-pAr-pAr-pGr-pAr-pAr-pCr-pAr-pCr-pCr-pCr-pAr | Cyclic phosphorylation | 31 | 3.56 | 100 | 1 | 47.31 | 836.194 | -12 | 10043.4404 | 10047.97 | 10043.4047 | 987617.56 |
| 1163-1199 | Cr-pUr-pCr-pCr-pGr-pCr-pCr-pAr-pGr-pCr-pUr-pUr-pCr-pAr-pG | Cyclic phosphorylation | 37 | -2.07 | 97 | 1.3 | 47.59 | 693.319 | -17 | 11798.498 | 11803.71 | 11798.5225 | 986614.44 |
| 1163-1199 | Cr-pUr-pCr-pCr-pGr-pCr-pCr-pAr-pGr-pCr-pUr-pUr-pCr-pAr-pG | Cyclic phosphorylation | 37 | 0.17 | 97 | 1.2 | 47.61 | 736.714 | -16 | 11798.5244 | 11803.74 | 11798.5225 | 999914.5 |
| 698-736 | Ur-pCr-pUr-pGr-pCr-pCr-pUr-pCr-pAr-pGr-pGr-pGr-pCr-pUr-pU | Cyclic phosphorylation | 39 | 3.37 | 94.6 | 1.6 | 47.71 | 690.524 | -18 | 12443.5889 | 12449.17 | 12443.5469 | 871643.88 |
| 698-736 | Ur-pCr-pUr-pGr-pCr-pCr-pUr-pCr-pAr-pGr-pGr-pGr-pCr-pUr-pU | Cyclic phosphorylation | 39 | -0.08 | 94.6 | 1.1 | 47.71 | 777.028 | -16 | 12443.5459 | 12449.02 | 12443.5469 | 2053482.62 |
| 698-736 | Ur-pCr-pUr-pGr-pCr-pCr-pUr-pCr-pAr-pGr-pGr-pGr-pCr-pUr-pU | Cyclic phosphorylation | 39 | 4 | 94.6 | 1.2 | 47.71 | 828.898 | -15 | 12443.5967 | 12449.22 | 12443.5469 | 906373.06 |
| 698-736 | Ur-pCr-pUr-pGr-pCr-pCr-pUr-pCr-pAr-pGr-pGr-pGr-pCr-pUr-pU | Cyclic phosphorylation | 39 | 0.86 | 94.6 | 1.2 | 47.73 | 731.32 | -17 | 12443.5576 | 12449.24 | 12443.5469 | 2317225.75 |
| 2075-2106 | Cr-pCr-pAr-pGr-pAr-pCr-pAr-pCr-pAr-pGr-pAr-pCr-pAr-pAr-pAr | Cyclic phosphorylation | 32 | 1.08 | 99.6 | 2 | 48.3 | 793.954 | -13 | 10330.4941 | 10335 | 10330.483 | 444742.62 |
| 2075-2106 | Cr-pCr-pAr-pGr-pAr-pCr-pAr-pCr-pAr-pGr-pAr-pCr-pAr-pAr-pAr | Cyclic phosphorylation | 32 | -1.19 | 99.6 | 1.1 | 48.3 | 737.1 | -14 | 10330.4707 | 10335.02 | 10330.483 | 990052.44 |
| 2075-2106 | Cr-pCr-pAr-pGr-pAr-pCr-pAr-pCr-pAr-pGr-pAr-pCr-pAr-pAr-pAr | Cyclic phosphorylation | 32 | -0.44 | 99.6 | 2 | 48.3 | 688.026 | -15 | 10330.4785 | 10335.36 | 10330.483 | 349110.38 |
| 2306-2343 | Cr-pCr-pUr-pGr-pCr-pUr-pGr-pCr-pUr-pGr-pCr-pAr-pGr-pUr-pA | Cyclic phosphorylation | 38 | 1.64 | 98.1 | 1 | 48.51 | 758.342 | -16 | 12143.5898 | 12149.11 | 12143.5699 | 3897453.25 |
| 2306-2343 | Cr-pCr-pUr-pGr-pCr-pUr-pGr-pCr-pUr-pGr-pCr-pAr-pGr-pUr-pA | Cyclic phosphorylation | 38 | 0.68 | 98.1 | 1.1 | 48.51 | 808.965 | -15 | 12143.5781 | 12149.19 | 12143.5699 | 2012077.38 |
| 2306-2343 | Cr-pCr-pUr-pGr-pCr-pUr-pGr-pCr-pUr-pGr-pCr-pAr-pGr-pUr-pA | Cyclic phosphorylation | 38 | -0.37 | 98.1 | 1.2 | 48.53 | 673.97 | -18 | 12143.5654 | 12149.1 | 12143.5699 | 1178008.5 |
| 1196-1232 | Cr-pGr-pUr-pGr-pUr-pCr-pCr-pCr-pCr-pUr-pAr-pCr-pCr-pAr-pAr | Cyclic phosphorylation | 37 | 2.62 | 93 | 1.4 | 49.29 | 734.777 | -16 | 11766.5635 | 11771.9 | 11766.5326 | 2054287.62 |
| 1196-1232 | Cr-pGr-pUr-pGr-pUr-pCr-pCr-pCr-pCr-pUr-pAr-pCr-pCr-pAr-pAr | Cyclic phosphorylation | 37 | -1.36 | 93 | 1.5 | 49.31 | 691.437 | -17 | 11766.5166 | 11771.71 | 11766.5326 | 1884679.88 |
| 1196-1232 | Cr-pGr-pUr-pGr-pUr-pCr-pCr-pCr-pCr-pUr-pAr-pCr-pCr-pAr-pAr | Cyclic phosphorylation | 37 | -0.86 | 93 | 1.2 | 49.31 | 783.763 | -15 | 11766.5225 | 11771.75 | 11766.5326 | 1114055.88 |
| 1196-1232 | Cr-pGr-pUr-pGr-pUr-pCr-pCr-pCr-pCr-pUr-pAr-pCr-pCr-pAr-pAr | Cyclic phosphorylation | 37 | 0.46 | 93 | 1.4 | 49.34 | 839.676 | -14 | 11766.5381 | 11771.19 | 11766.5326 | 284662.5 |
| 4212-4234 | Cr-pUr-pAr-pAr-pAr-pAr-pAr-pAr-pAr-pAr-pAr-pAr-pAr-pAr-pA | Cyclic phosphorylation | 23 | 0.83 | 99 | 1.6 | 49.54 | 751.411 | -10 | 7521.1758 | 7524.48 | 7521.1695 | 771497.75 |
| 4212-4234 | Cr-pUr-pAr-pAr-pAr-pAr-pAr-pAr-pAr-pAr-pAr-pAr-pAr-pAr-pA | Cyclic phosphorylation | 23 | -1.96 | 99 | 1.9 | 49.54 | 683.009 | -11 | 7521.1548 | 7524.33 | 7521.1695 | 607540.12 |
| 104-141 | Cr-pCr-pUr-pGr-pAr-pCr-pCr-pAr-pCr-pCr-pAr-pGr-pAr-pAr-pCr | Cyclic phosphorylation | 38 | 1.13 | 99.8 | 1.8 | 51.22 | 669.253 | -18 | 12059.6729 | 12064.94 | 12059.6593 | 1596929.25 |
| 104-141 | Cr-pCr-pUr-pGr-pAr-pCr-pCr-pAr-pCr-pCr-pAr-pGr-pAr-pAr-pCr | Cyclic phosphorylation | 38 | 1.29 | 99.8 | 1.4 | 51.22 | 753.035 | -16 | 12059.6748 | 12065.45 | 12059.6593 | 1008900.5 |
| 104-141 | Cr-pCr-pUr-pGr-pAr-pCr-pCr-pAr-pCr-pCr-pAr-pGr-pAr-pAr-pCr | Cyclic phosphorylation | 38 | 2.1 | 99.8 | 1.5 | 51.23 | 708.739 | -17 | 12059.6846 | 12065.07 | 12059.6593 | 1373296.25 |
| 104-141 | Cr-pCr-pUr-pGr-pAr-pCr-pCr-pAr-pCr-pCr-pAr-pGr-pAr-pAr-pCr | Cyclic phosphorylation | 38 | 1.69 | 99.8 | 1.6 | 51.24 | 803.371 | -15 | 12059.6797 | 12065.13 | 12059.6593 | 539561.81 |
| 1136-1177 | Cr-pGr-pUr-pGr-pGr-pCr-pCr-pGr-pAr-pCr-pUr-pAr-pCr-pUr-pC | Cyclic phosphorylation | 42 | -0.86 | 91.1 | 1.8 | 51.45 | 703.98 | -19 | 13388.7188 | 13394.63 | 13388.7303 | 429101.38 |
| 4212-4235 | Cr-pUr-pAr-pAr-pAr-pAr-pAr-pAr-pAr-pAr-pAr-pAr-pAr-pAr-pA | Cyclic phosphorylation | 24 | 0.14 | 96.2 | 1.7 | 51.64 | 712.923 | -11 | 7850.2231 | 7853.65 | 7850.222 | 925909.06 |
| 4212-4235 | Cr-pUr-pAr-pAr-pAr-pAr-pAr-pAr-pAr-pAr-pAr-pAr-pAr-pAr-pA | Cyclic phosphorylation | 24 | -0.67 | 96.2 | 1.5 | 51.64 | 784.316 | -10 | 7850.2168 | 7853.82 | 7850.222 | 662670.06 |
| 1514-1555 | Cr-pUr-pGr-pCr-pUr-pAr-pCr-pUr-pUr-pCr-pCr-pCr-pAr-pCr-pU | Cyclic phosphorylation | 42 | -0.02 | 92.8 | 1.2 | 52.34 | 737.034 | -18 | 13277.7119 | 13283.78 | 13277.7122 | 1385755 |
| 1514-1555 | Cr-pUr-pGr-pCr-pUr-pAr-pCr-pUr-pUr-pCr-pCr-pCr-pAr-pCr-pU | Cyclic phosphorylation | 42 | -1.78 | 92.8 | 1.3 | 52.36 | 698.137 | -19 | 13277.6885 | 13283.58 | 13277.7122 | 626303.44 |
| 104-142 | Cr-pCr-pUr-pGr-pAr-pCr-pCr-pAr-pCr-pCr-pAr-pGr-pAr-pAr-pCr | Cyclic phosphorylation | 39 | -1.72 | 99.8 | 1.1 | 52.52 | 728.036 | -17 | 12388.6904 | 12394.49 | 12388.7118 | 5521631.5 |
| 104-142 | Cr-pCr-pUr-pGr-pAr-pCr-pCr-pAr-pCr-pCr-pAr-pGr-pAr-pAr-pCr | Cyclic phosphorylation | 39 | -7.71 | 99.8 | 1.8 | 52.53 | 825.308 | -15 | 12388.6162 | 12394.25 | 12388.7118 | 832784.88 |
| 104-142 | Cr-pCr-pUr-pGr-pAr-pCr-pCr-pAr-pCr-pCr-pAr-pGr-pAr-pAr-pCr | Cyclic phosphorylation | 39 | 3.56 | 99.8 | 1.2 | 52.53 | 773.601 | -16 | 12388.7559 | 12394.32 | 12388.7118 | 2522050.25 |
| 4212-4236 | Cr-pUr-pAr-pAr-pAr-pAr-pAr-pAr-pAr-pAr-pAr-pAr-pAr-pAr-pA | Cyclic phosphorylation | 25 | -0.97 | 99.8 | 1.8 | 53.42 | 817.321 | -10 | 8179.2666 | 8183.14 | 8179.2745 | 431122.75 |
| 4212-4236 | Cr-pUr-pAr-pAr-pAr-pAr-pAr-pAr-pAr-pAr-pAr-pAr-pAr-pAr-pA | Cyclic phosphorylation | 25 | 1.18 | 99.8 | 1.9 | 53.42 | 742.837 | -11 | 8179.2842 | 8182.85 | 8179.2745 | 914457.75 |
| 745-787 | Ur-pCr-pGr-pGr-pCr-pAr-pUr-pCr-pAr-pAr-pCr-pAr-pUr-pCr-pA | Cyclic phosphorylation | 43 | 1.37 | 91.8 | 1.3 | 53.92 | 684.333 | -20 | 13701.8066 | 13707.83 | 13701.7879 | 1570059.25 |
| 745-787 | Ur-pCr-pGr-pGr-pCr-pAr-pUr-pCr-pAr-pAr-pCr-pAr-pUr-pCr-pA | Cyclic phosphorylation | 43 | 0.8 | 91.8 | 1.1 | 53.92 | 760.482 | -18 | 13701.7988 | 13707.75 | 13701.7879 | 1064473.12 |
| 745-787 | Ur-pCr-pGr-pGr-pCr-pAr-pUr-pCr-pAr-pAr-pCr-pAr-pUr-pCr-pA | Cyclic phosphorylation | 43 | 0.73 | 91.8 | 1.1 | 53.92 | 720.456 | -19 | 13701.7979 | 13707.96 | 13701.7879 | 1853561.75 |
| 745-787 | Ur-pCr-pGr-pGr-pCr-pAr-pUr-pCr-pAr-pAr-pCr-pAr-pUr-pCr-pA | Cyclic phosphorylation | 43 | -2.77 | 91.8 | 1.1 | 53.94 | 805.393 | -17 | 13701.75 | 13707.97 | 13701.7879 | 581234.81 |
| 2521-2566 | Ur-pCr-pGr-pUr-pGr-pCr-pUr-pGr-pCr-pGr-pCr-pGr-pGr-pAr-pG | Cyclic phosphorylation | 46 | -2.35 | 97 | 1.5 | 55.26 | 745.441 | -20 | 14922.8613 | 14929.47 | 14922.8963 | 838992.56 |
| 3914-3966 | Cr-pCr-pCr-pCr-pUr-pUr-pUr-pCr-pCr-pCr-pGr-pUr-pCr-pCr-pUr | Cyclic phosphorylation | 53 | -8.25 | 100 | 1.3 | 55.37 | 838.251 | -20 | 16778.0039 | 16785.68 | 16778.1423 | 4885569.5 |
| 3914-3966 | Cr-pCr-pCr-pCr-pUr-pUr-pUr-pCr-pCr-pCr-pGr-pUr-pCr-pCr-pUr | Cyclic phosphorylation | 53 | -3.01 | 100 | 1.4 | 55.37 | 882.422 | -19 | 16778.0918 | 16785.81 | 16778.1423 | 2373104 |
| 3914-3966 | Cr-pCr-pCr-pCr-pUr-pUr-pUr-pCr-pCr-pCr-pGr-pUr-pCr-pCr-pUr | Cyclic phosphorylation | 53 | 1.53 | 100 | 1.6 | 55.37 | 931.557 | -18 | 16778.168 | 16785.58 | 16778.1423 | 1080794.12 |
| 3914-3966 | Cr-pCr-pCr-pCr-pUr-pUr-pUr-pCr-pCr-pCr-pGr-pUr-pCr-pCr-pUr | Cyclic phosphorylation | 53 | 0.6 | 100 | 1.5 | 55.37 | 798.286 | -21 | 16778.1523 | 16785.72 | 16778.1423 | 8087408 |
| 3914-3966 | Cr-pCr-pCr-pCr-pUr-pUr-pUr-pCr-pCr-pCr-pGr-pUr-pCr-pCr-pUr | Cyclic phosphorylation | 53 | -5.45 | 100 | 2 | 55.38 | 728.826 | -23 | 16778.0508 | 16784.87 | 16778.1423 | 408803.72 |
